# Supplementary material for: Use of trifluoroacetaldehyde N-tfsylhydrazone as a trifluorodiazoethane surrogate and its synthetic applications
Source: Nat Commun. 2019 Jan 17;10:284. doi: 10.1038/s41467-018-08253-z (PMC6336877; doi:10.1038/s41467-018-08253-z)
Supplement: Supplementary file 1 — Supplementary Information [file 41467_2018_8253_MOESM1_ESM.pdf]

Supplementary Information

Use of trifluoroacetaldehyde N-tfsylhydrazone as trifluorodiazoethane surrogate and its synthetic applications

Zhang et al

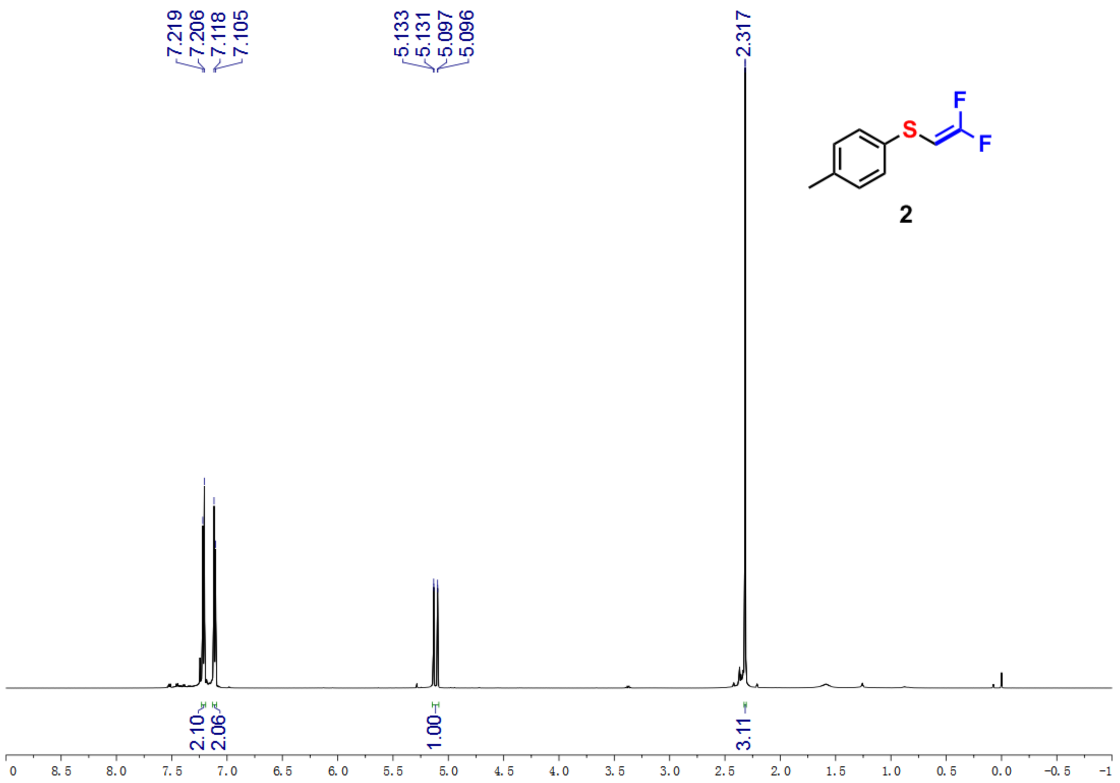

Supplementary Figure 1. ^{1}H NMR of 2

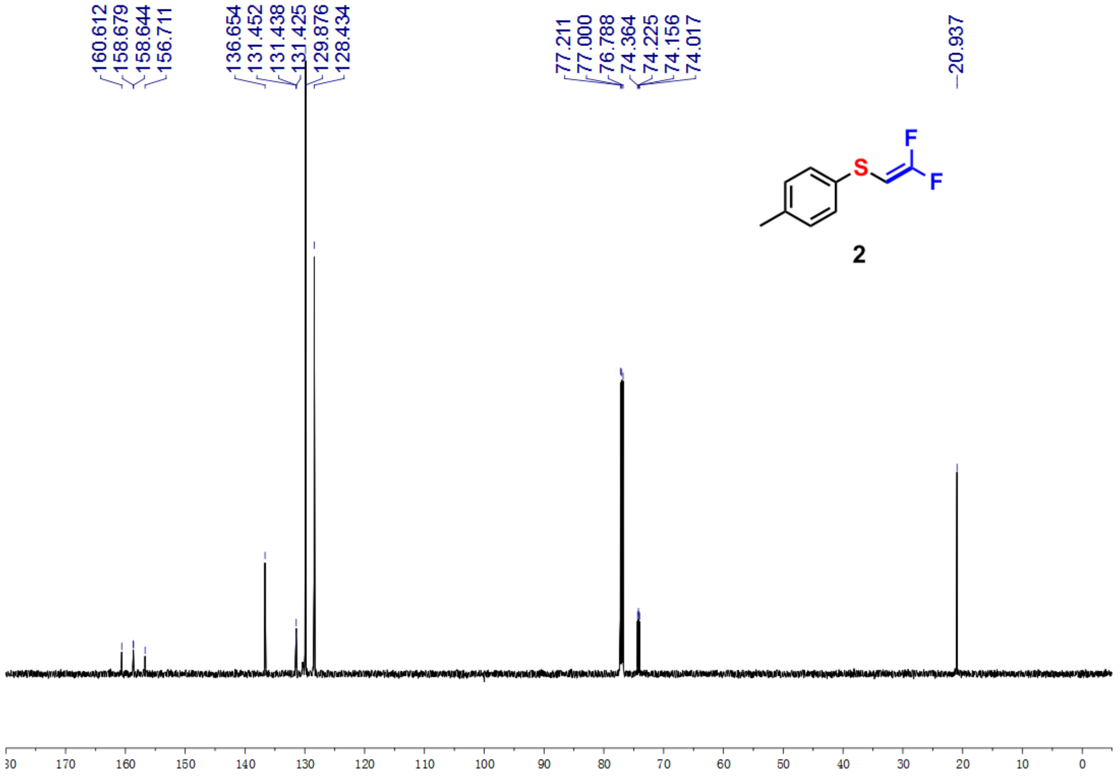

Supplementary Figure 2. ^{13}C NMR of 2

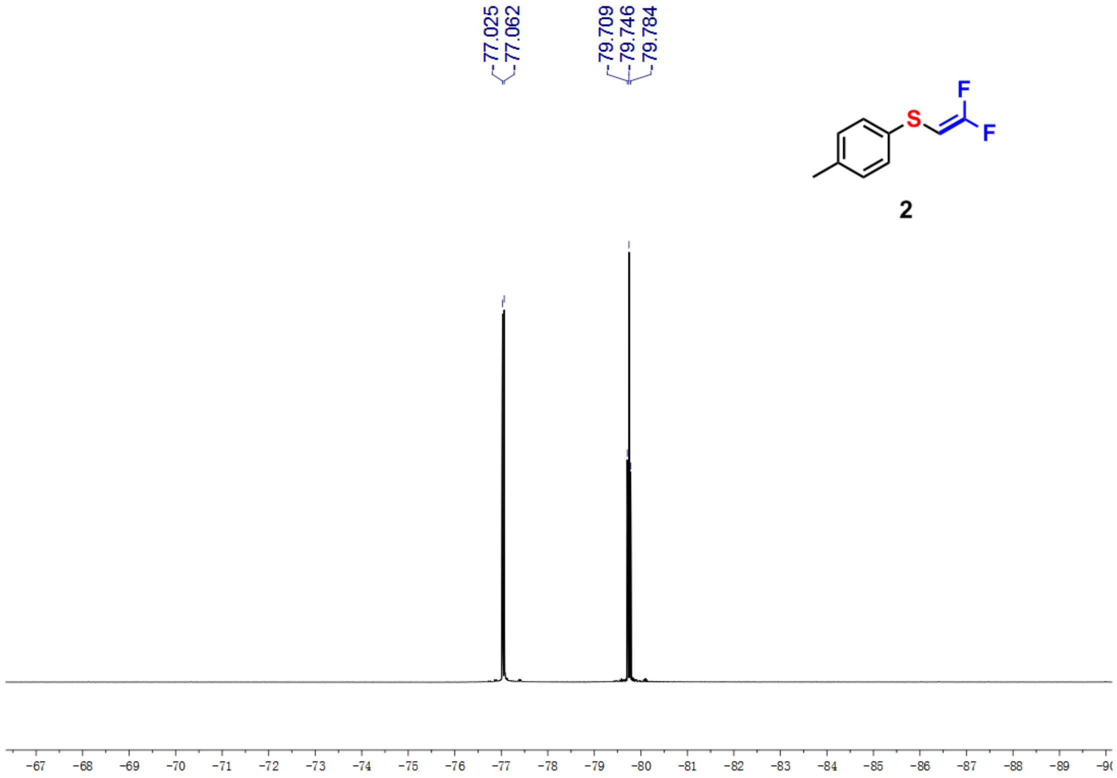

Supplementary Figure 3. ^{19}F NMR of 2

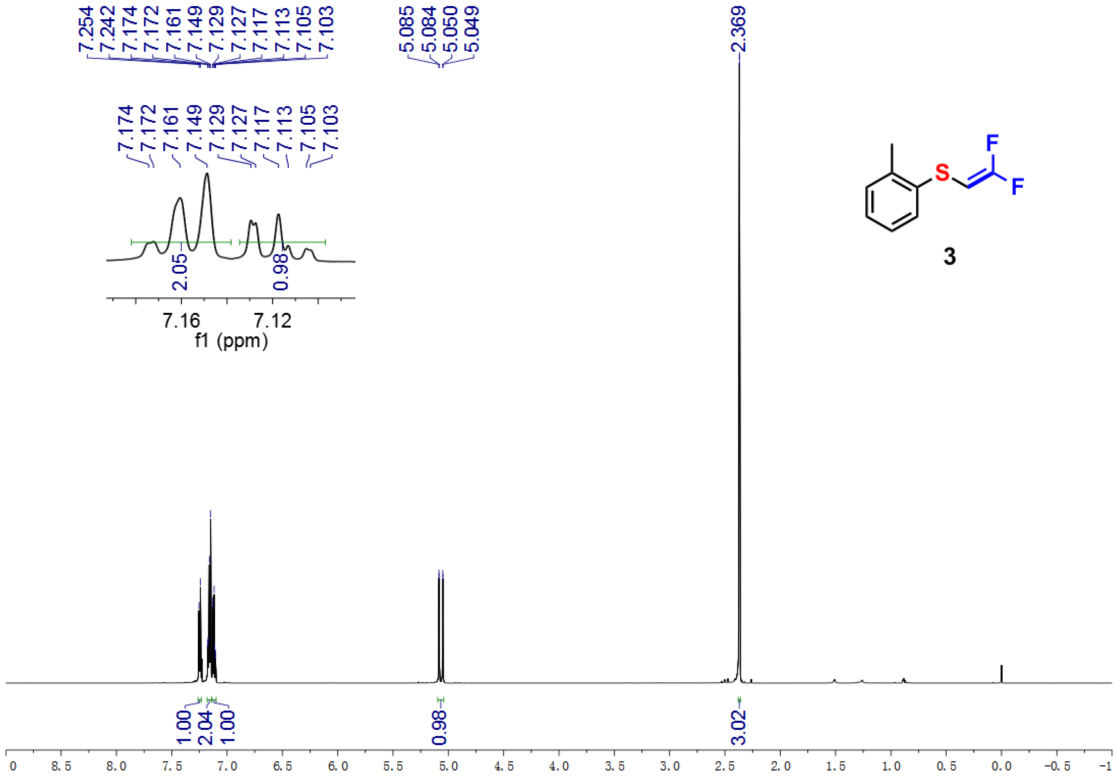

Supplementary Figure 4. ^{1}H NMR of 3

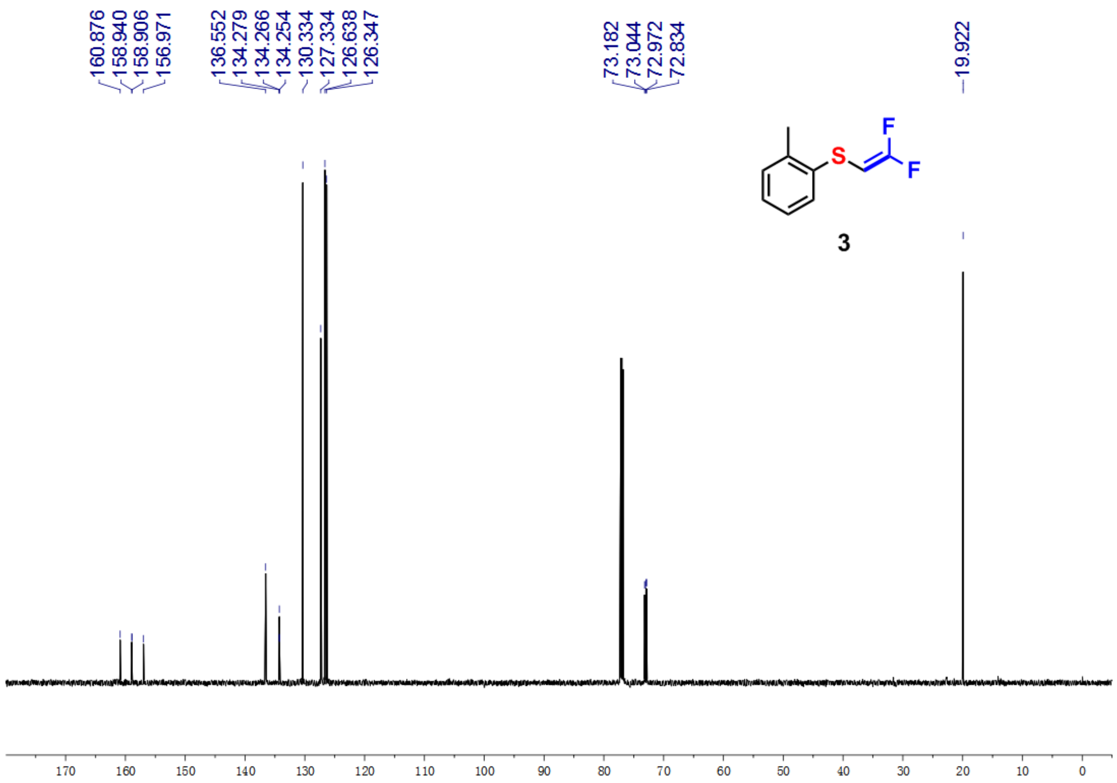

Supplementary Figure 5. ^{13}C NMR of 3

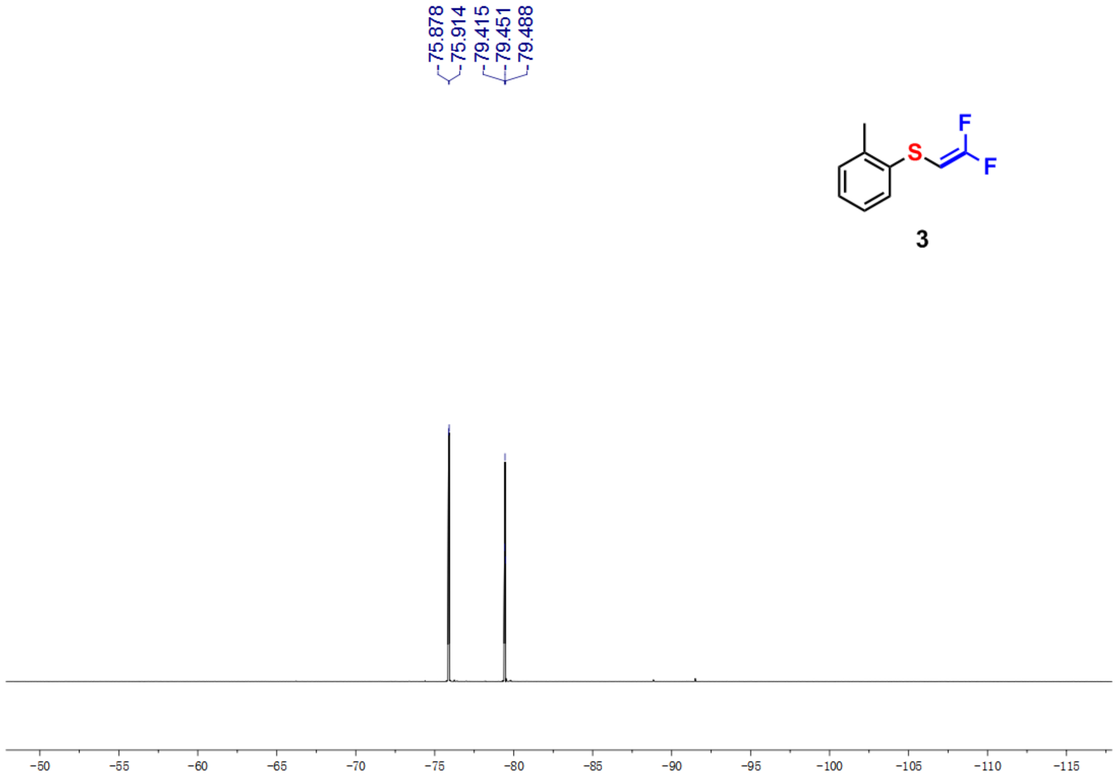

Supplementary Figure 6. ^{19}F NMR of 3

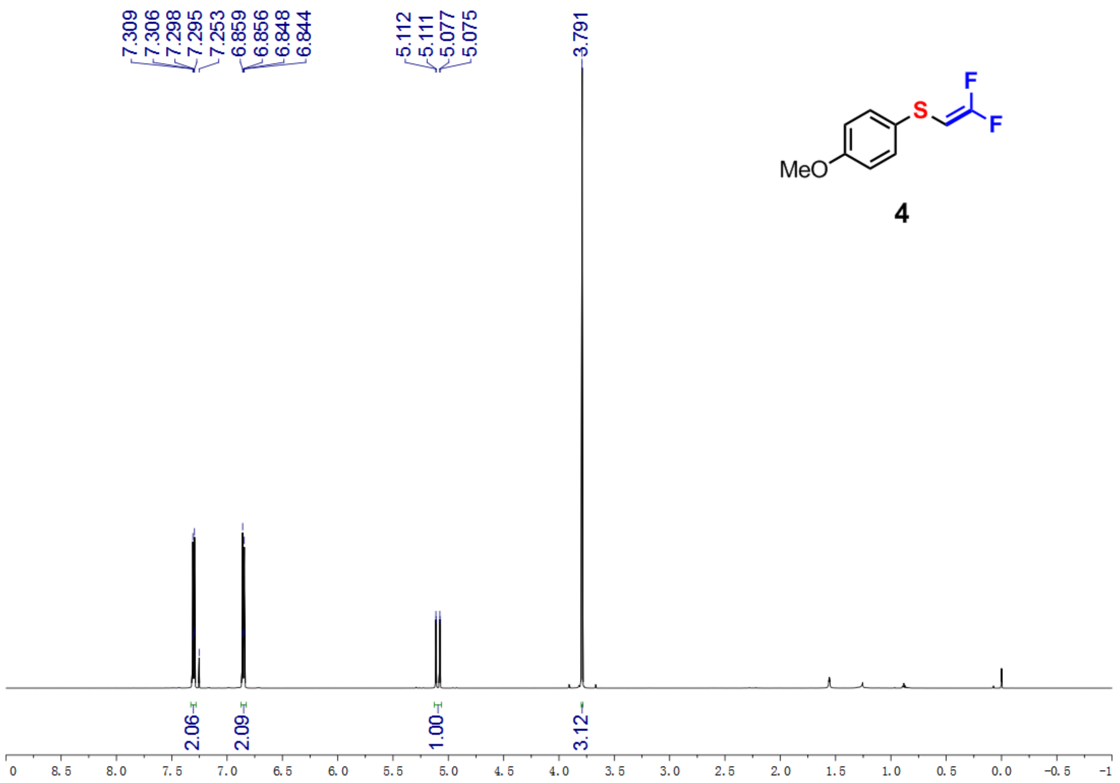

Supplementary Figure 7. ^{1}H NMR of 4

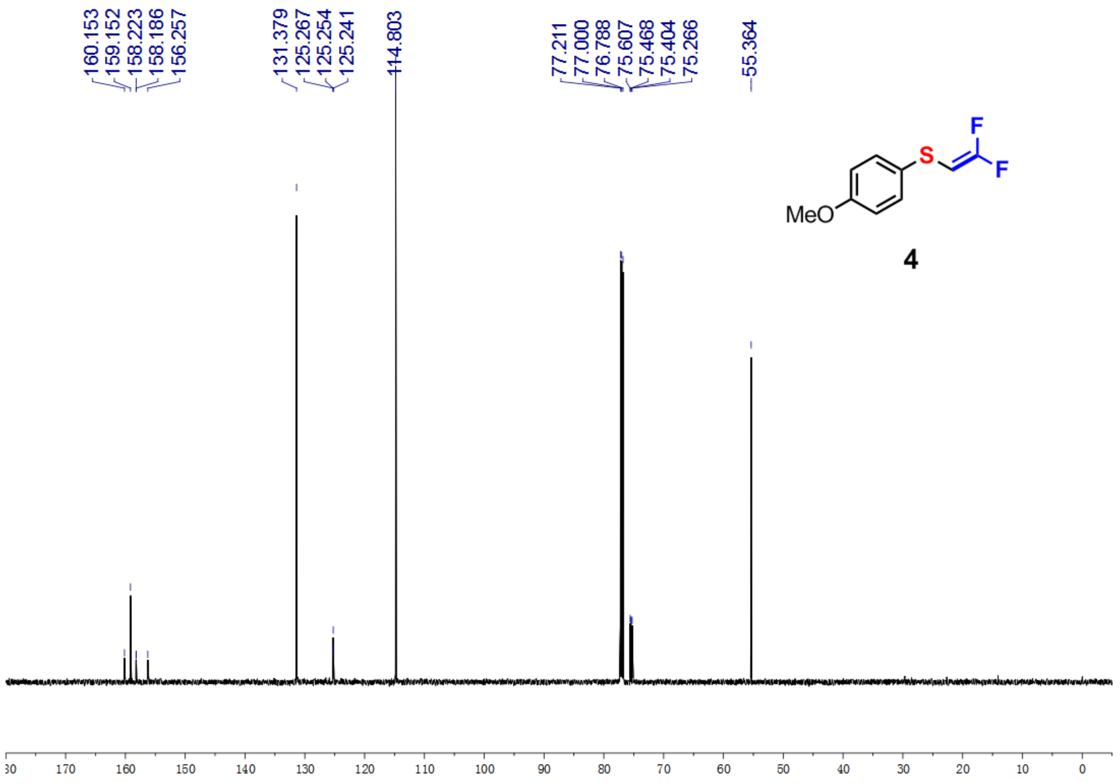

Supplementary Figure 8. ^{13}C NMR of 4

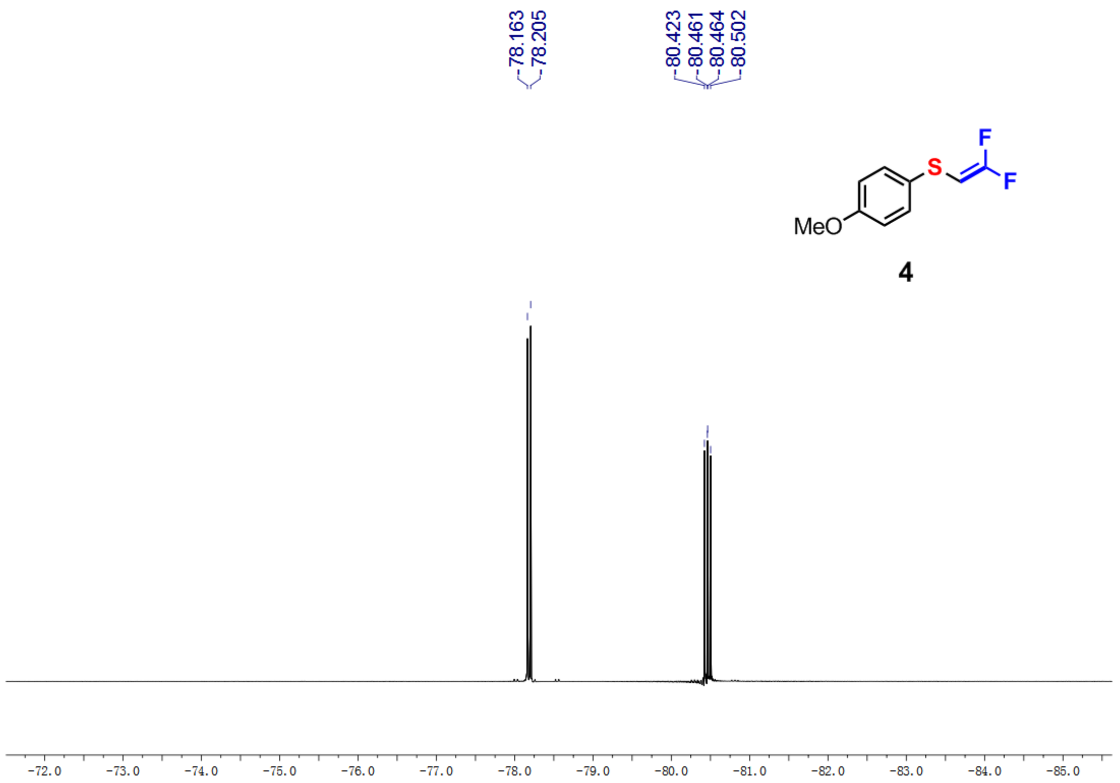

Supplementary Figure 9. ^{19}F NMR of 4

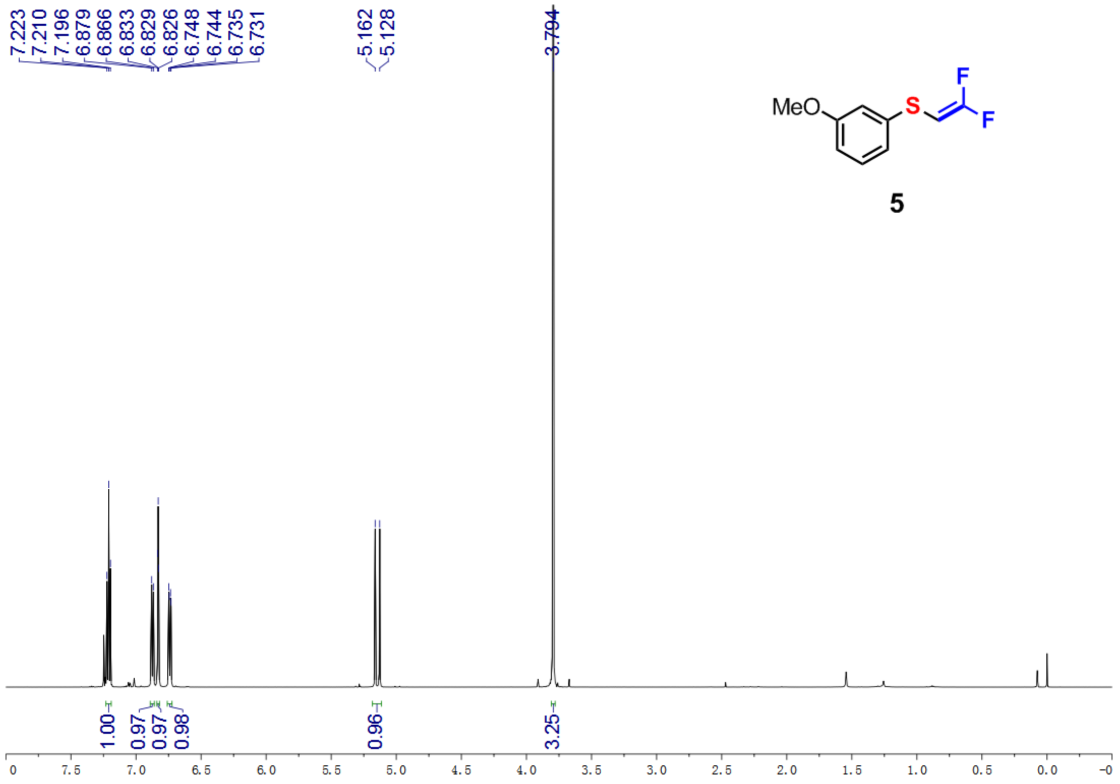

Supplementary Figure 10. ^{1}H NMR of 5

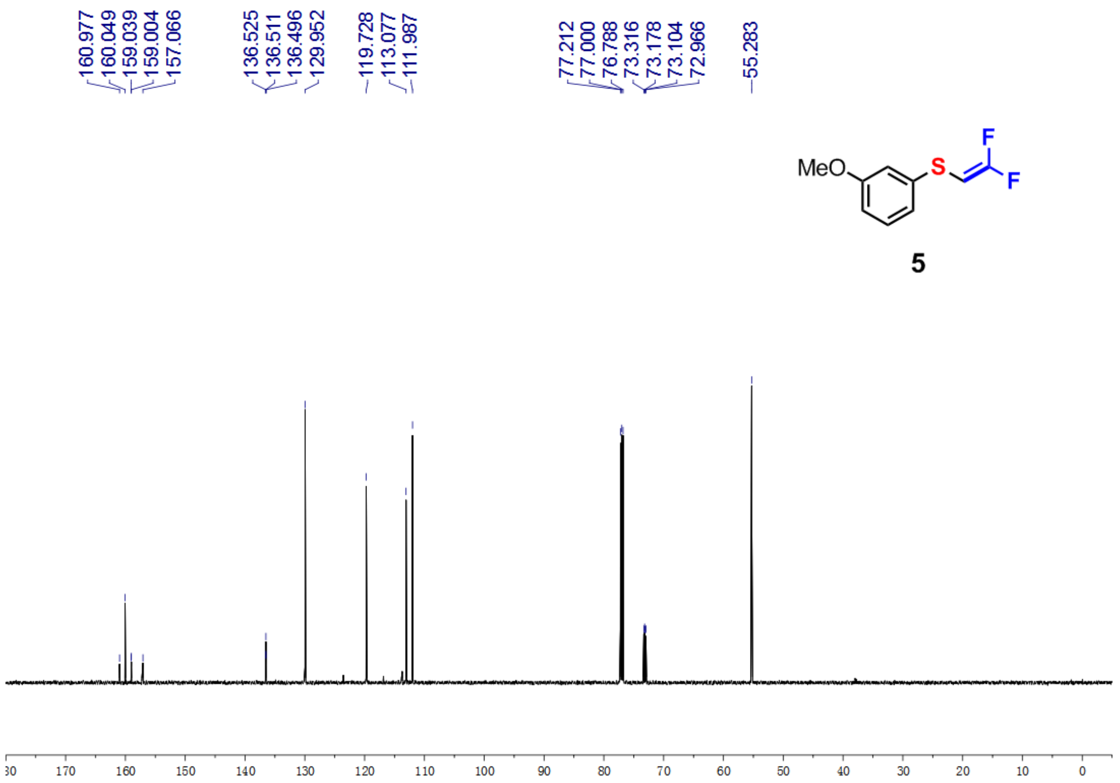

Supplementary Figure 11. ^{13}C NMR of 5

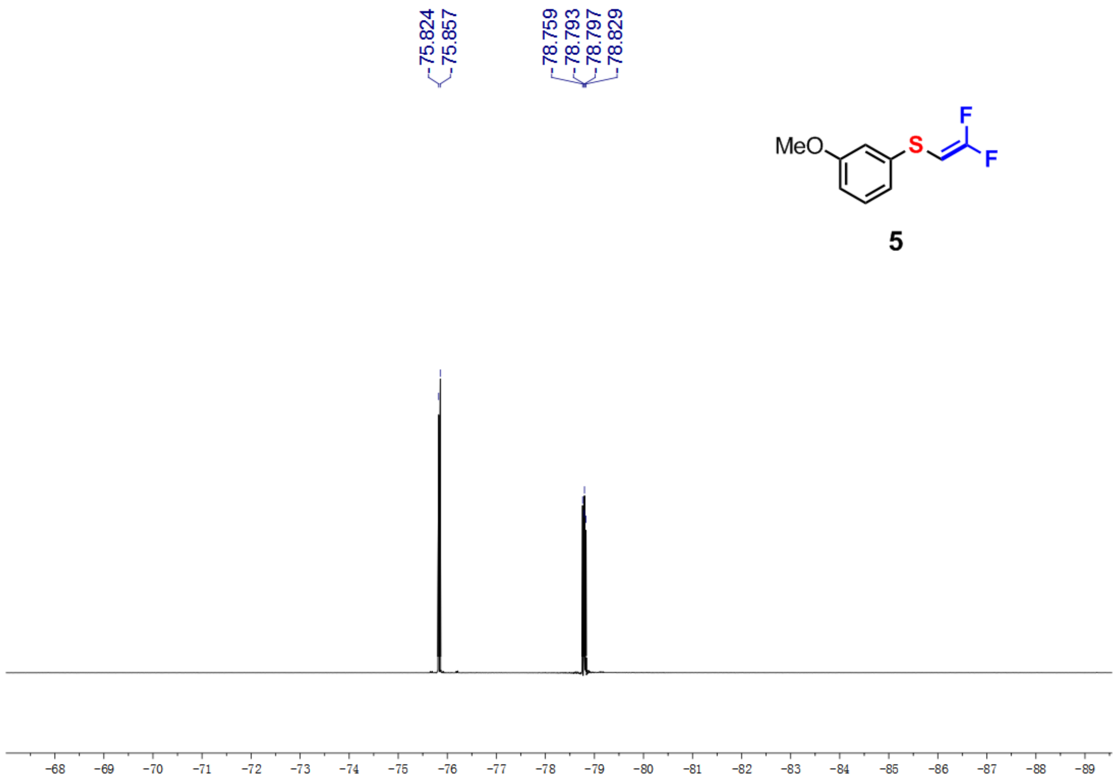

Supplementary Figure 12. ^{19}F NMR of 5

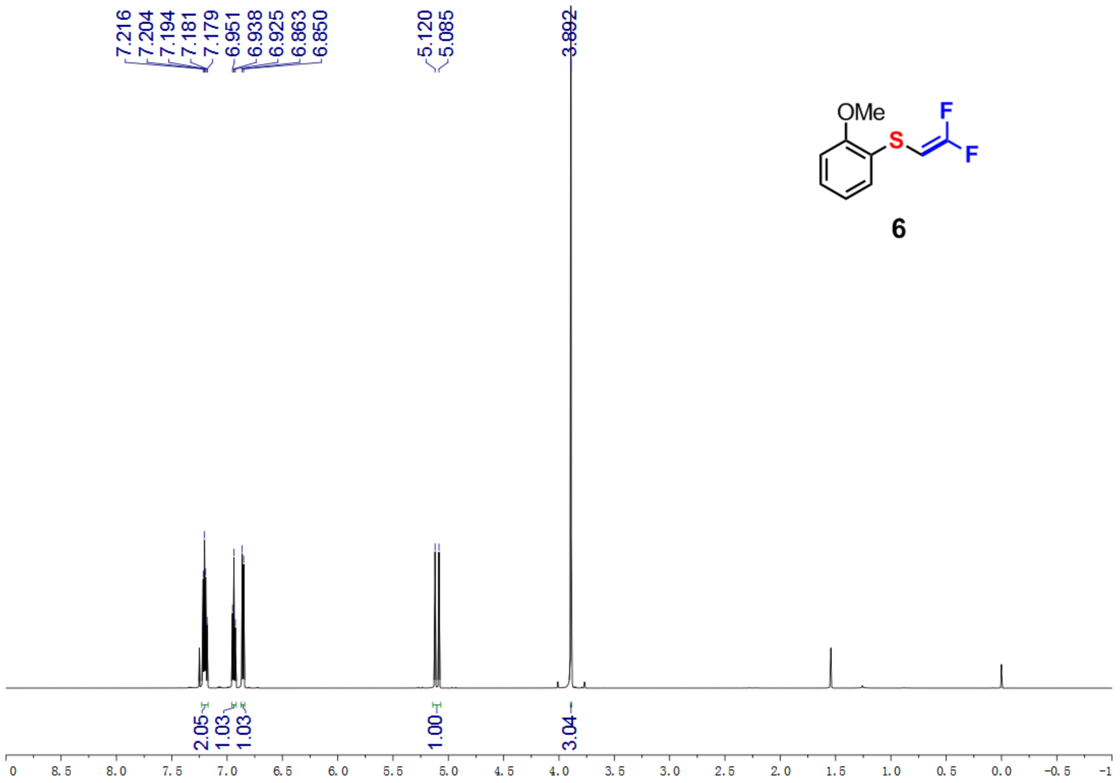

Supplementary Figure 13. ^{1}H NMR of 6

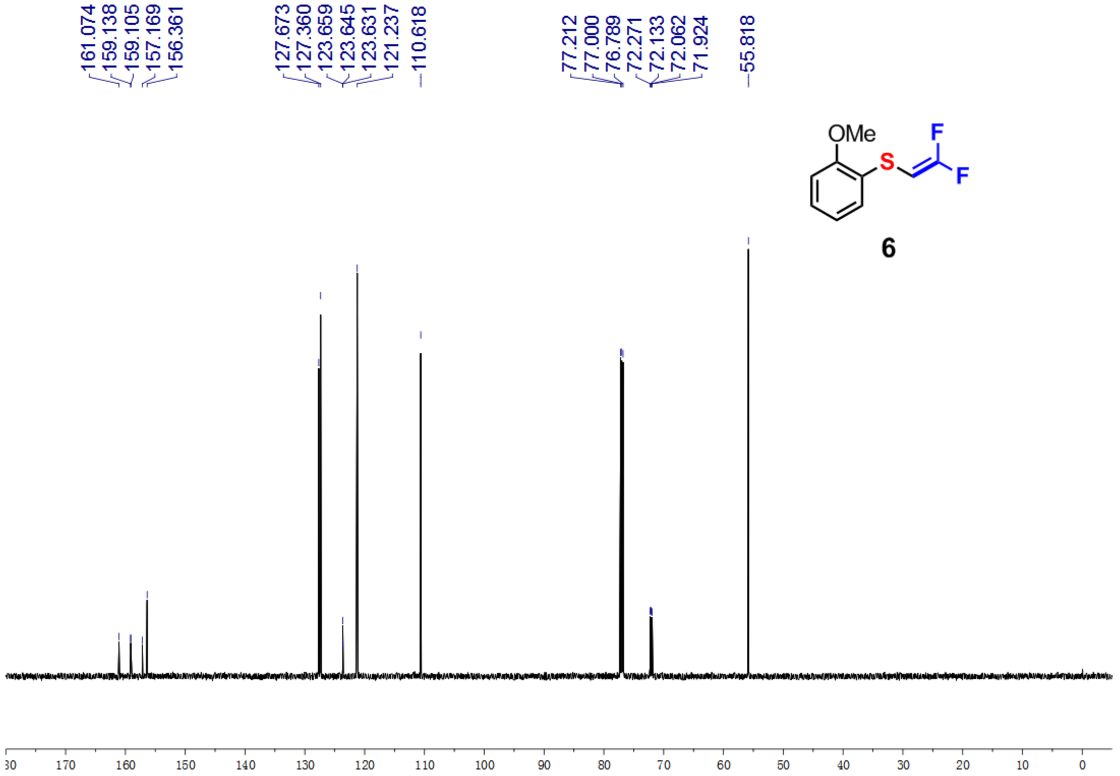

Supplementary Figure 14. ^{13}C NMR of 6

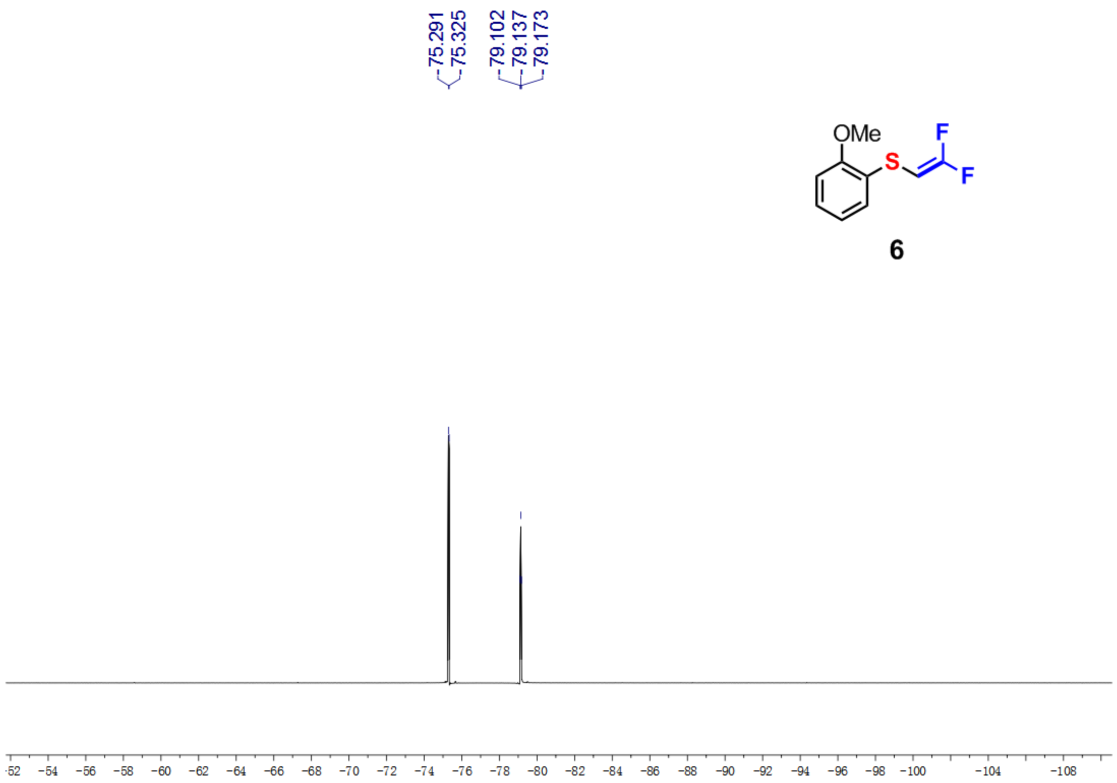

Supplementary Figure 15. ^{19}F NMR of 6

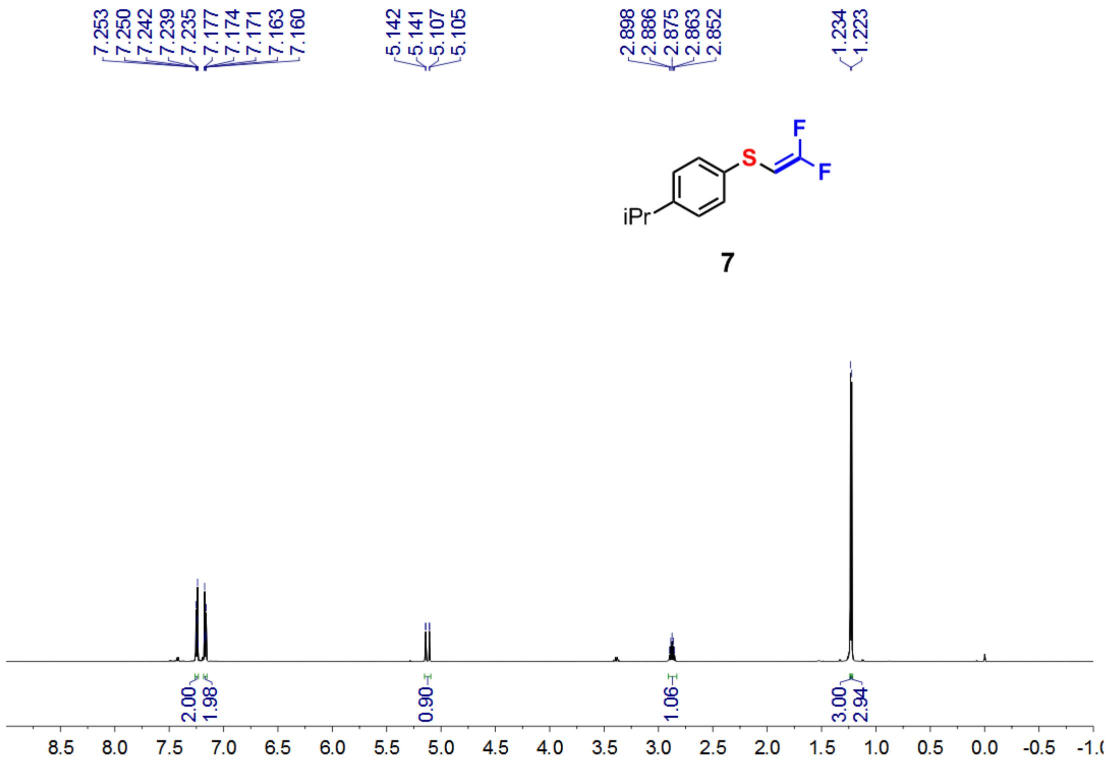

Supplementary Figure 16. ^{1}H NMR of 7

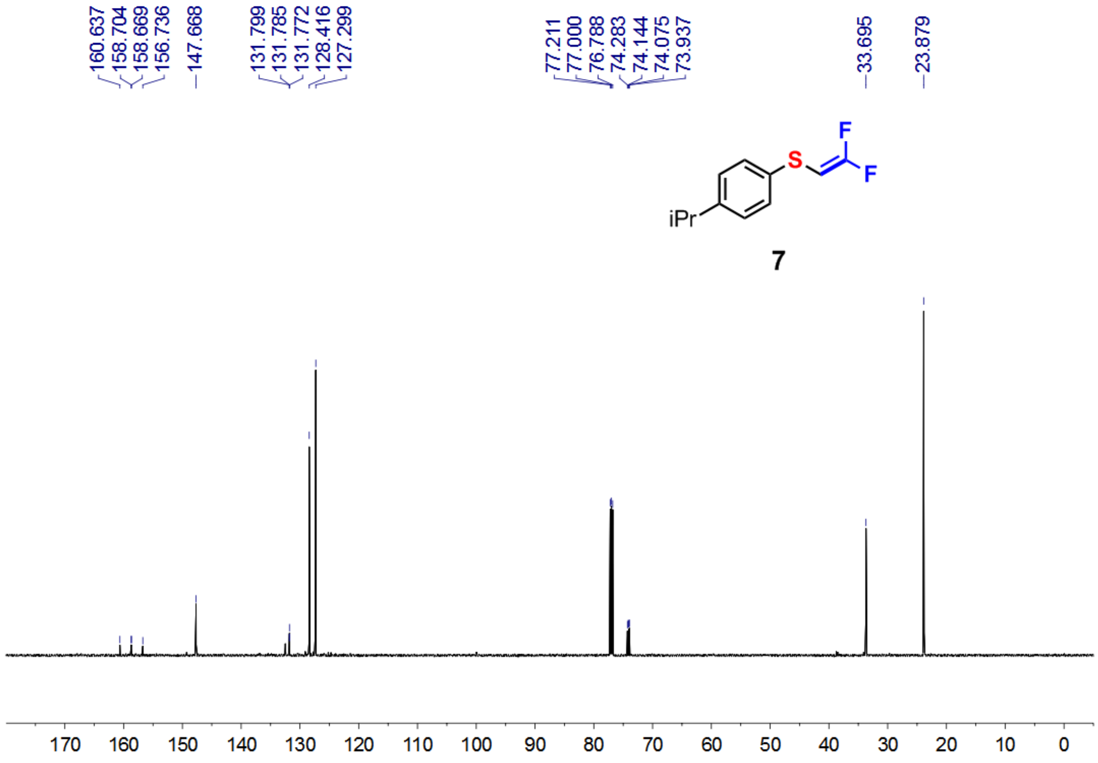

Supplementary Figure 17. ^{13}C NMR of 7

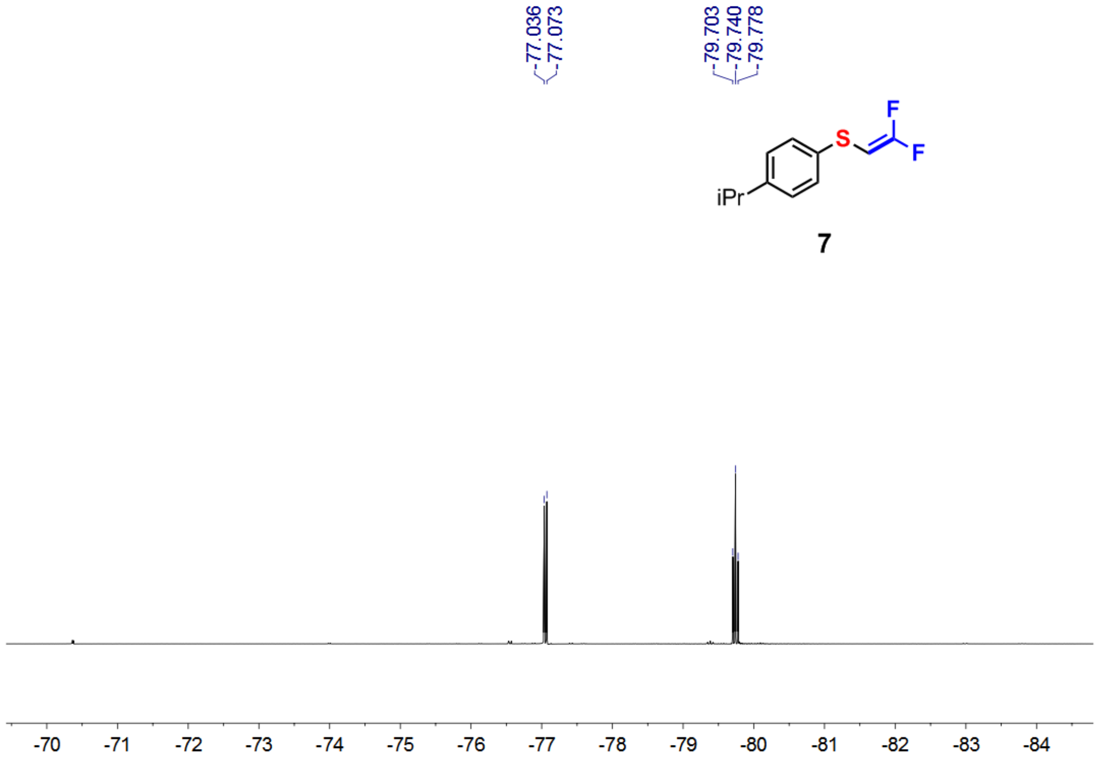

Supplementary Figure 18. ^{19}F NMR of 7

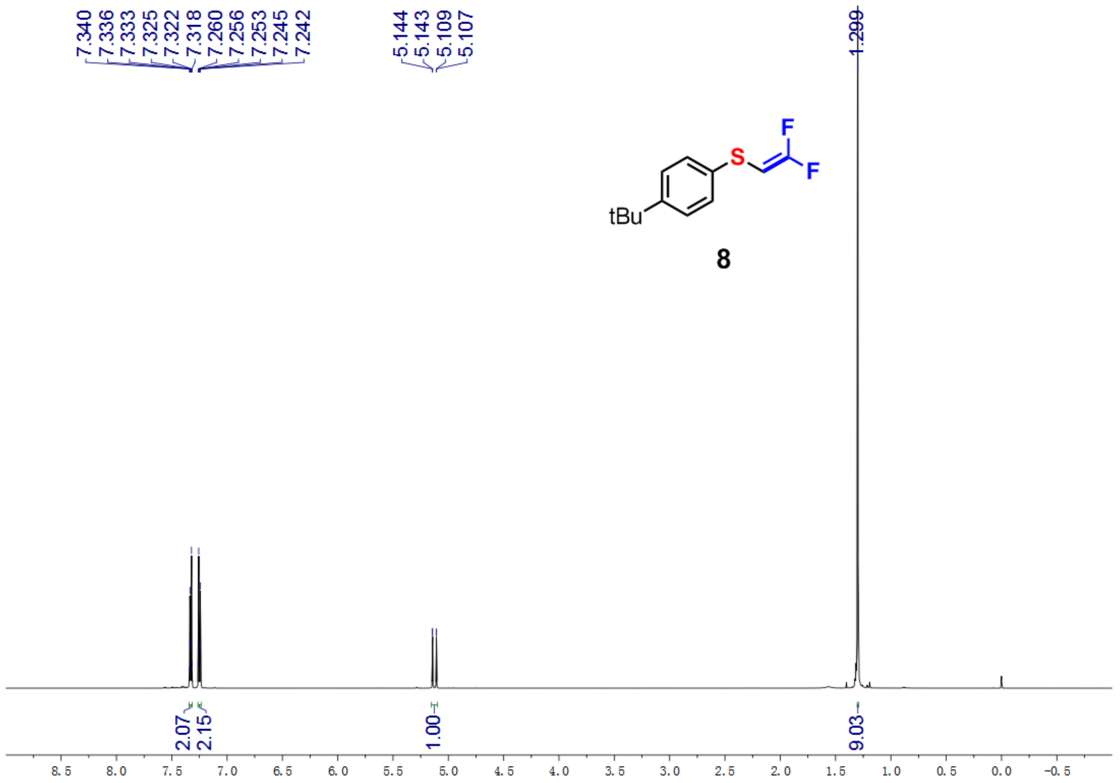

Supplementary Figure 19. ^{1}H NMR of 8

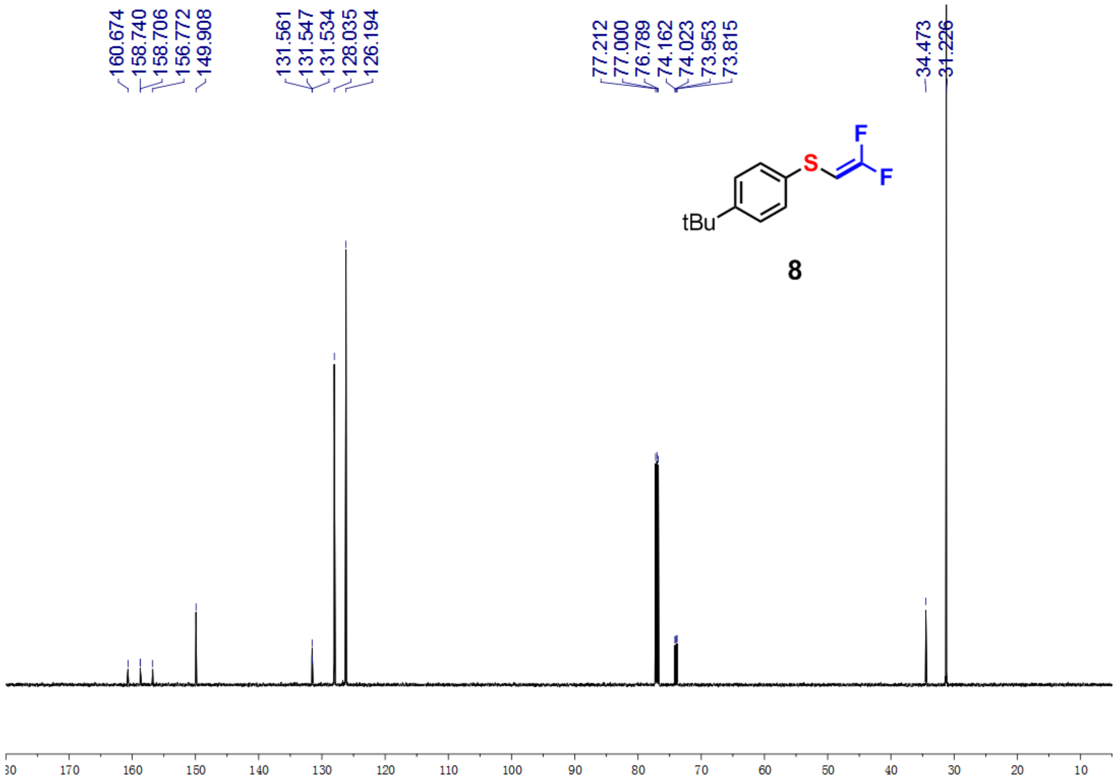

Supplementary Figure 20. ^{13}C NMR of 8

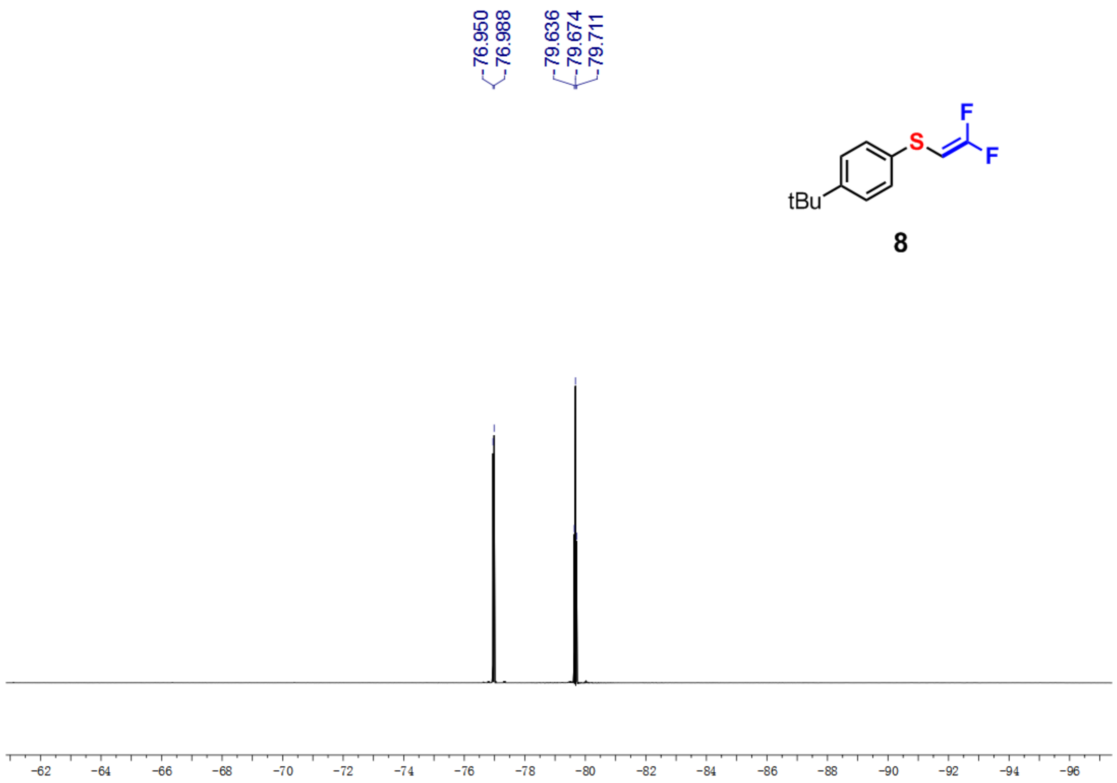

Supplementary Figure 21. ^{19}F NMR of 8

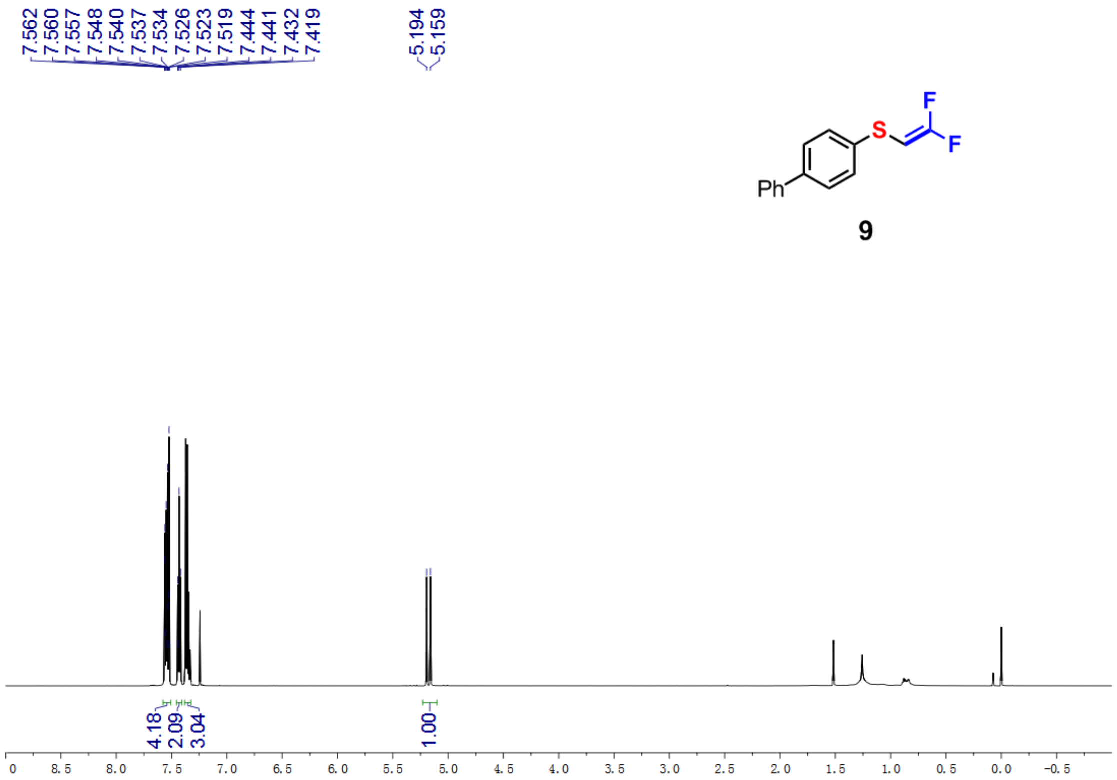

Supplementary Figure 22. ^{1}H NMR of 9

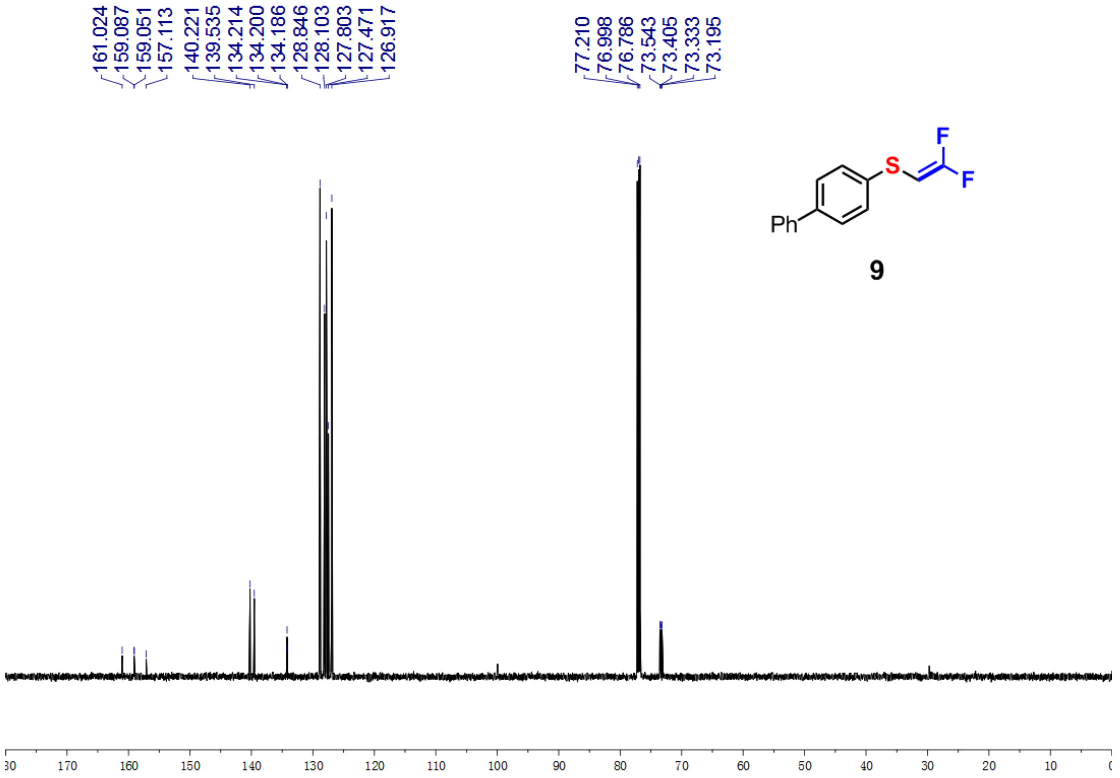

Supplementary Figure 23. ^{13}C NMR of 9

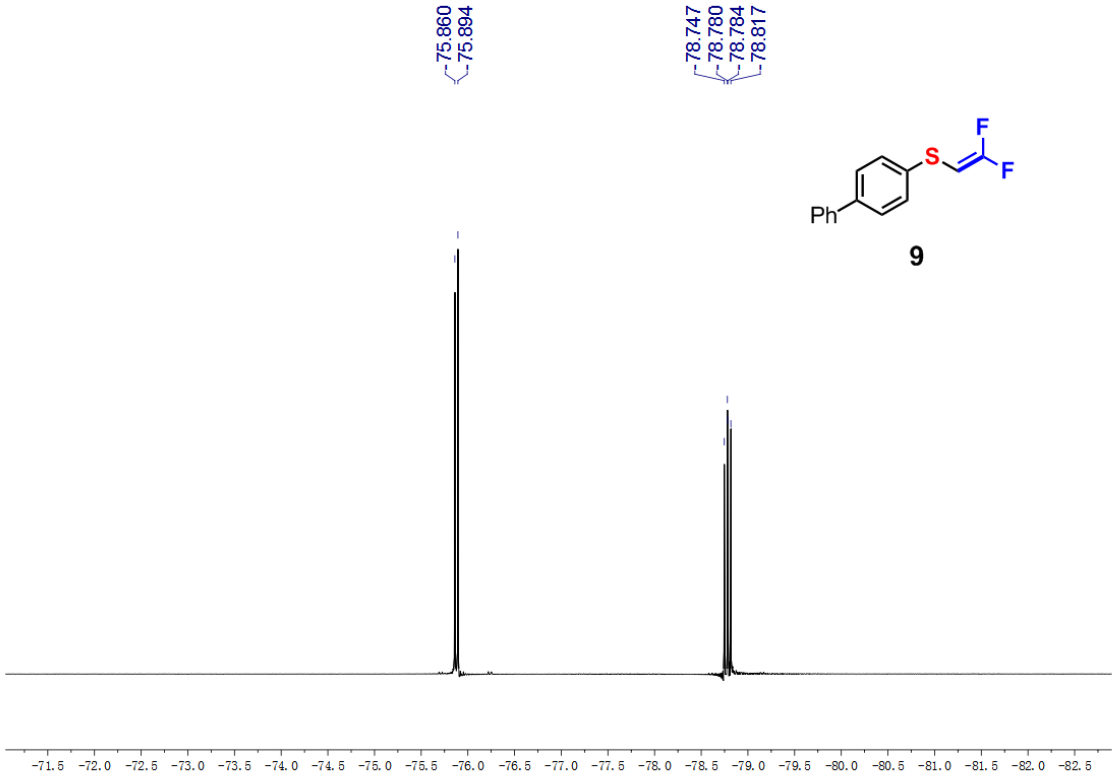

Supplementary Figure 24. ^{19}F NMR of 9

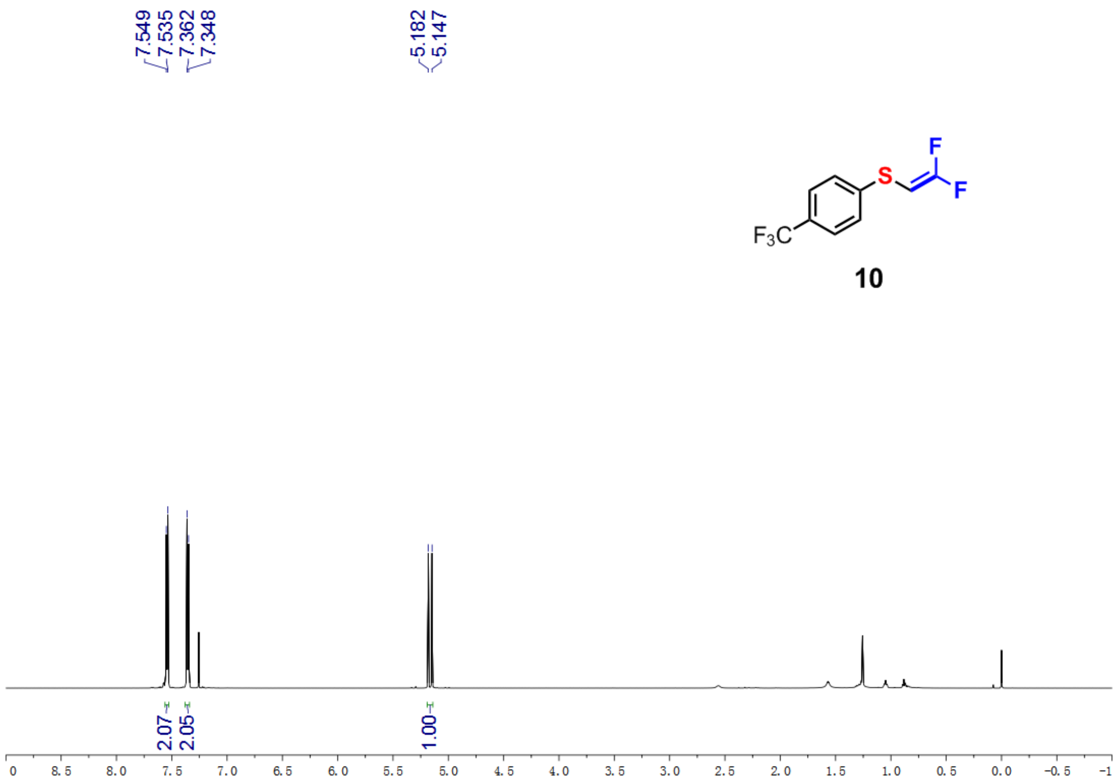

Supplementary Figure 25. ^{1}H NMR of 10

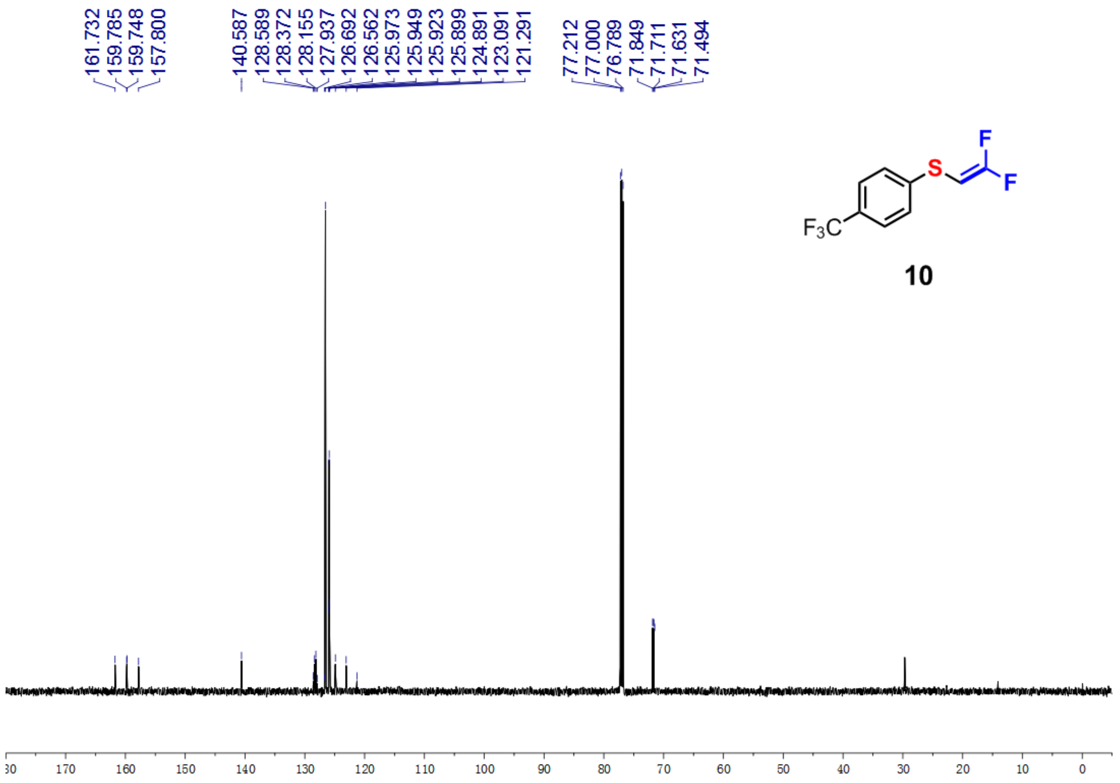

Supplementary Figure 26. ^{13}C NMR of 10

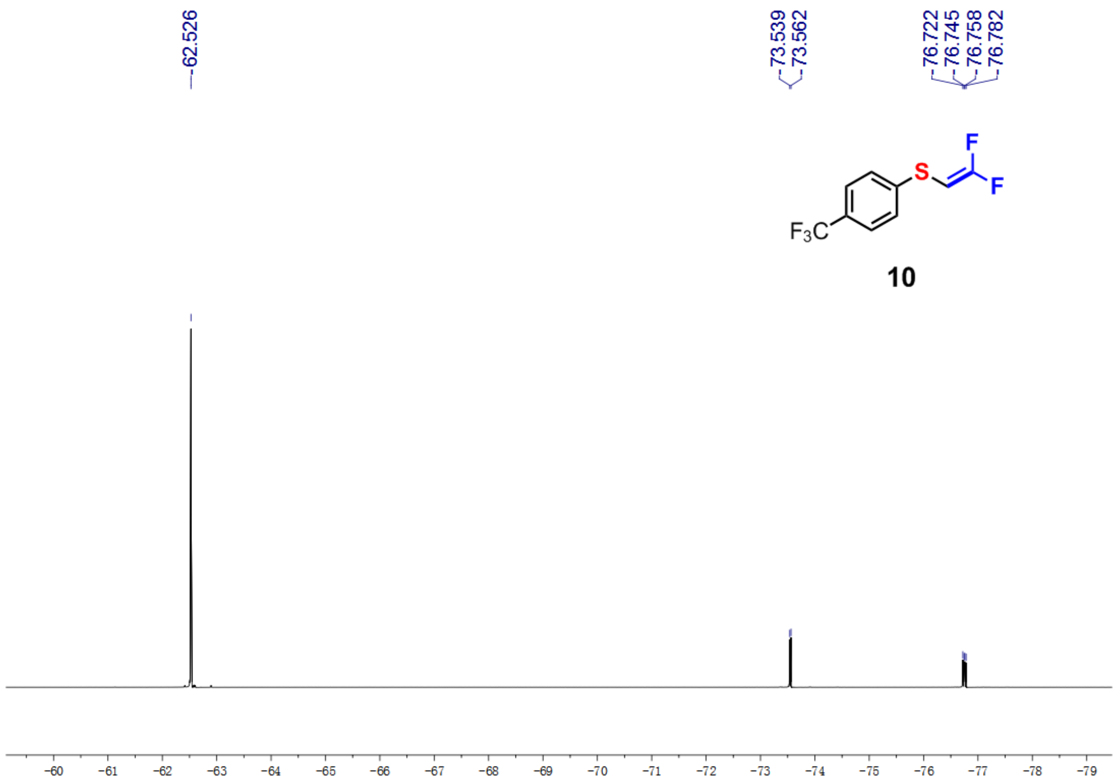

Supplementary Figure 27. ^{19}F NMR of 10

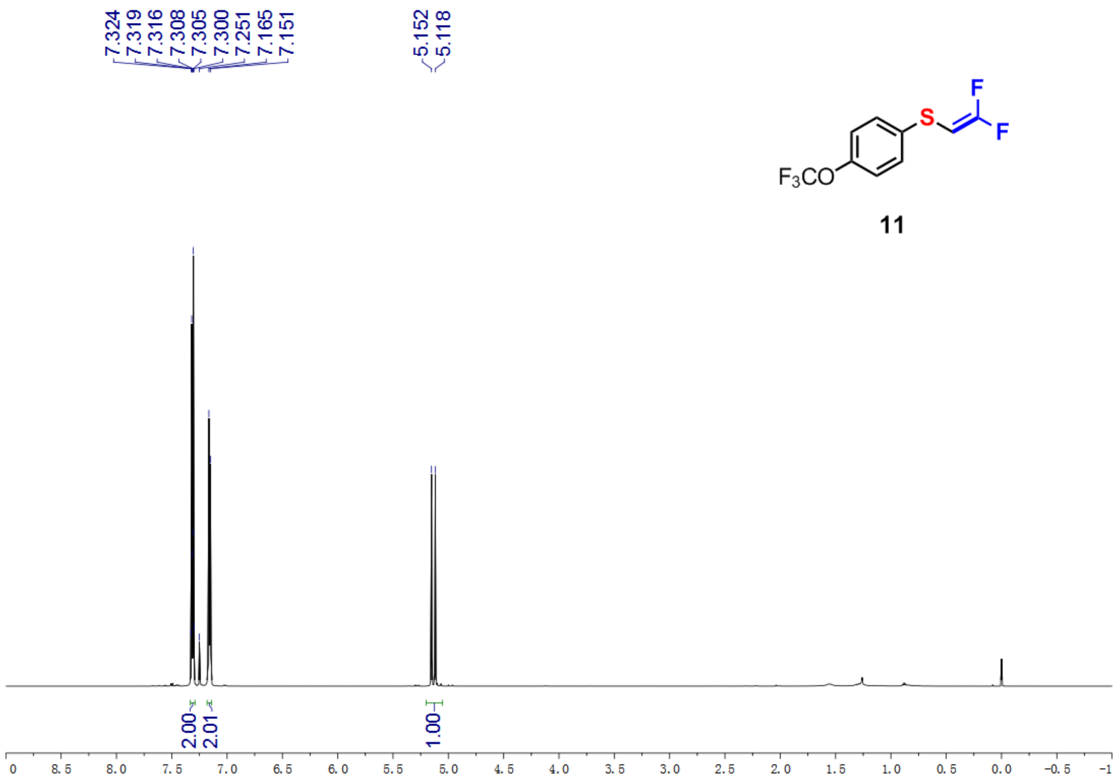

Supplementary Figure 28. ^{1}H NMR of 11

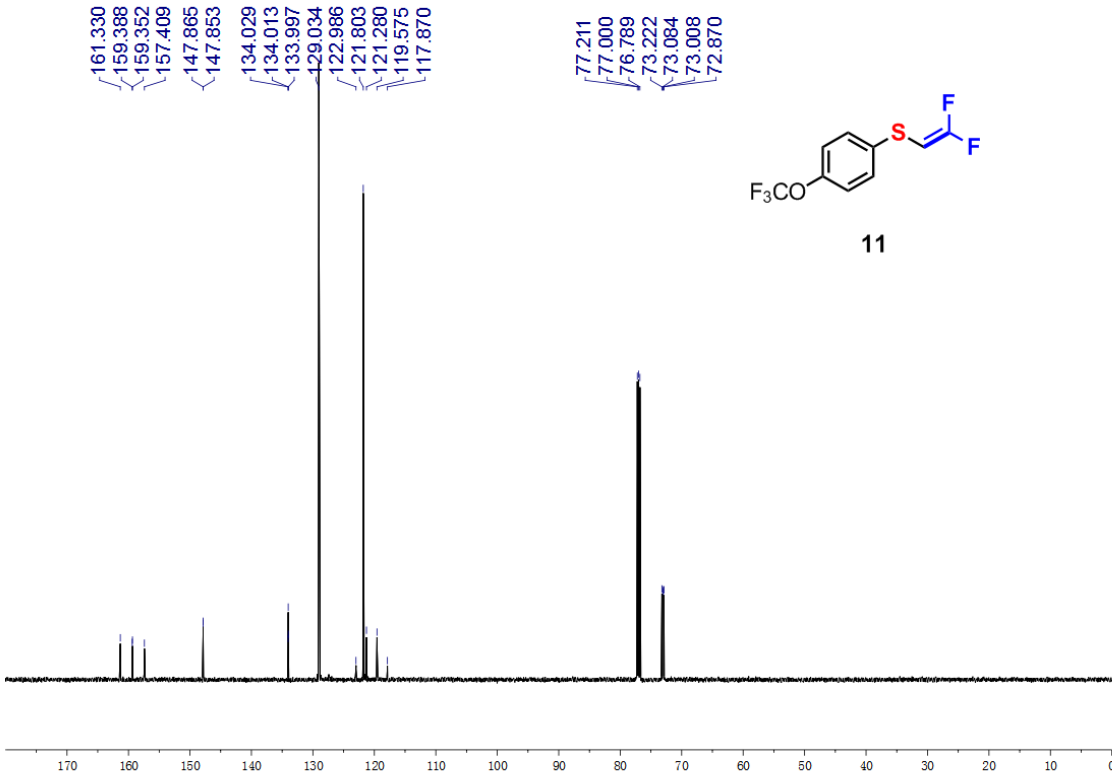

Supplementary Figure 29. ^{13}C NMR of 11

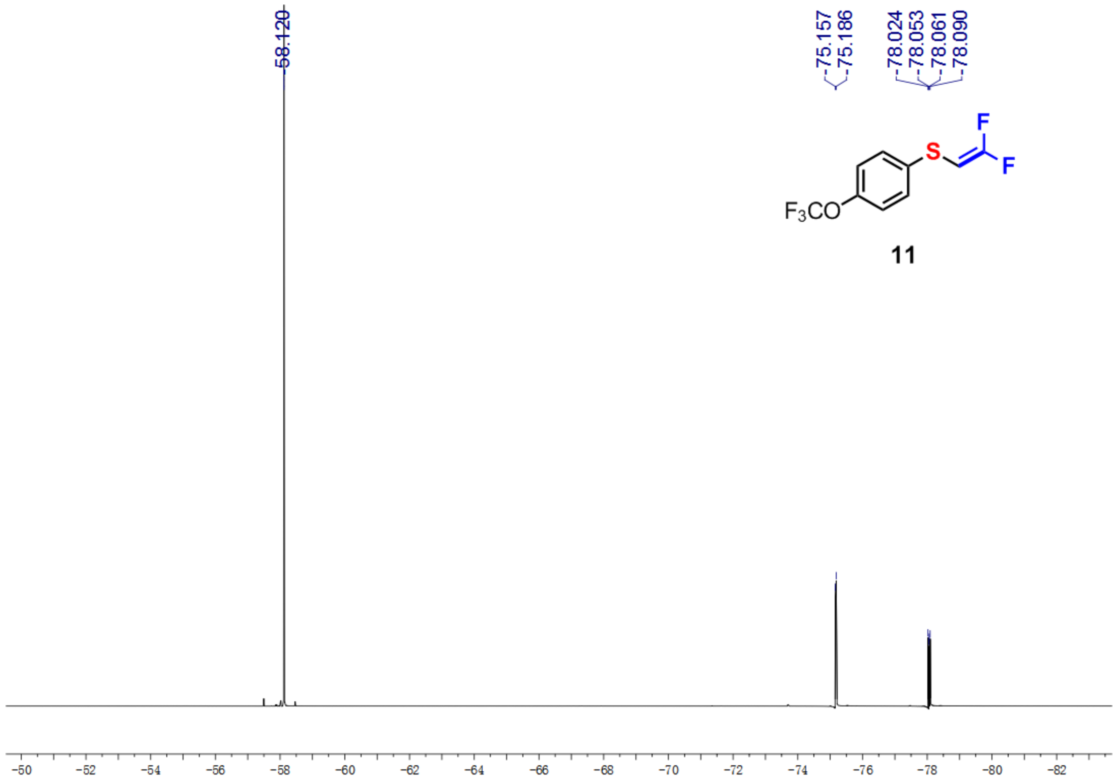

Supplementary Figure 30. ^{19}F NMR of 11

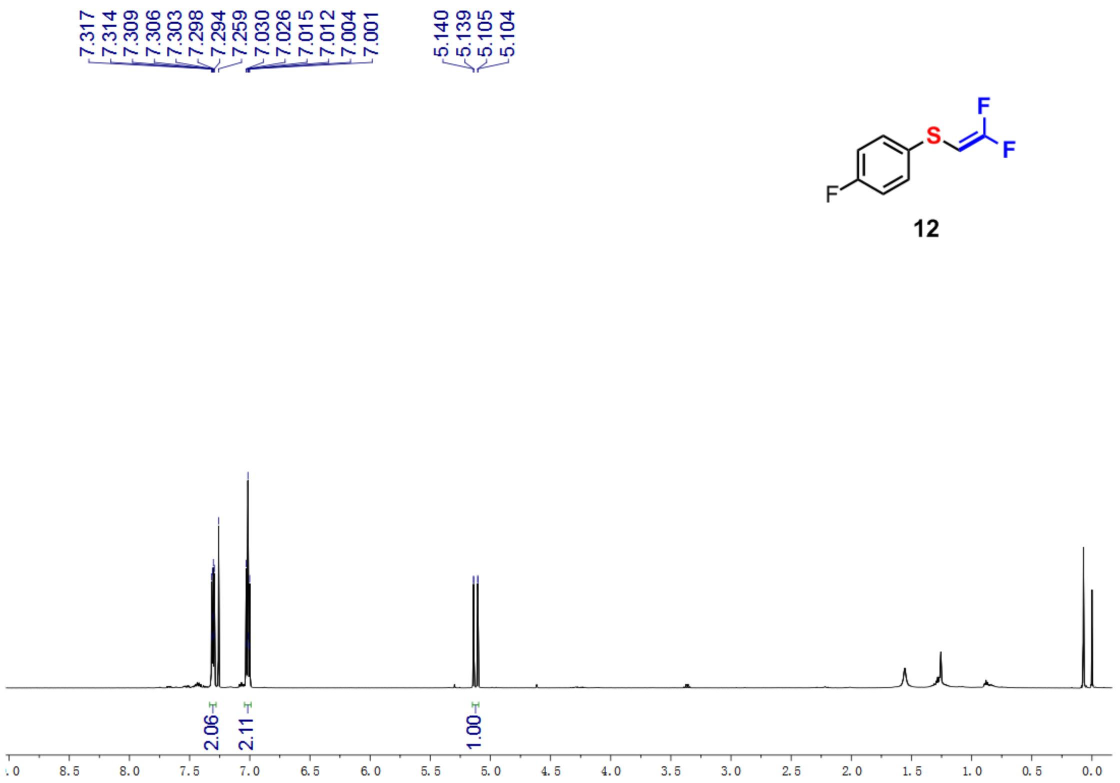

Supplementary Figure 31. ^{1}H NMR of 12

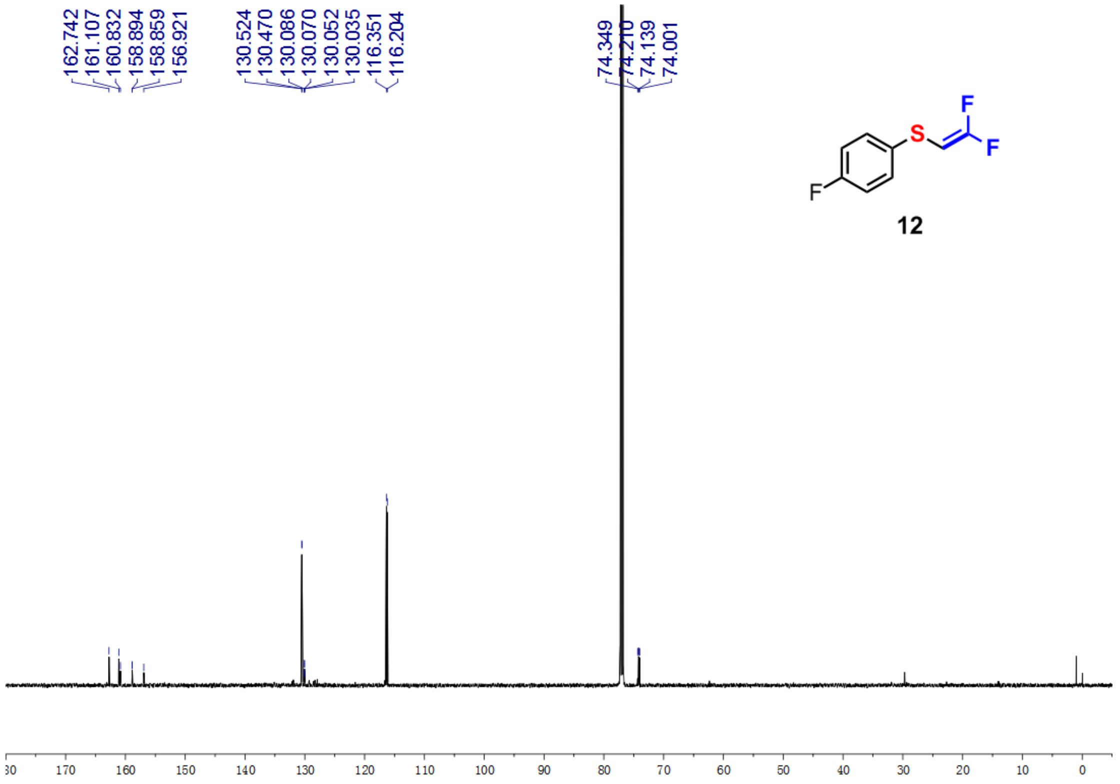

Supplementary Figure 32. ^{13}C NMR of 12

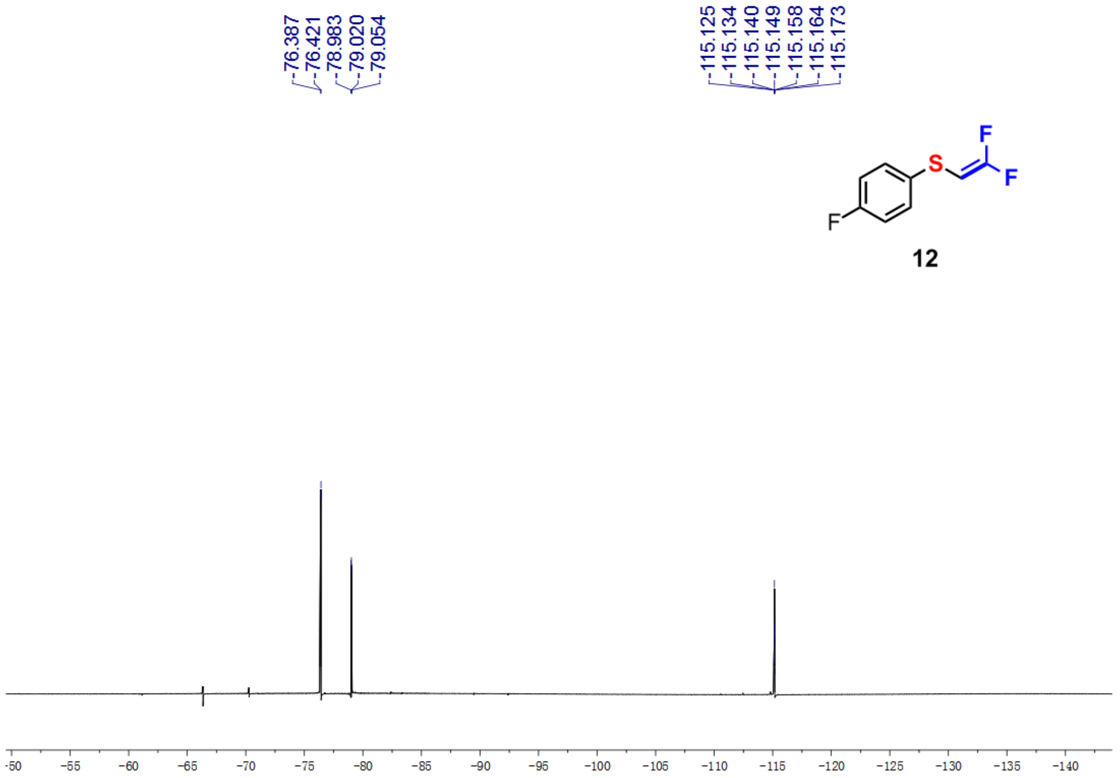

Supplementary Figure 33. ^{19}F NMR of 12

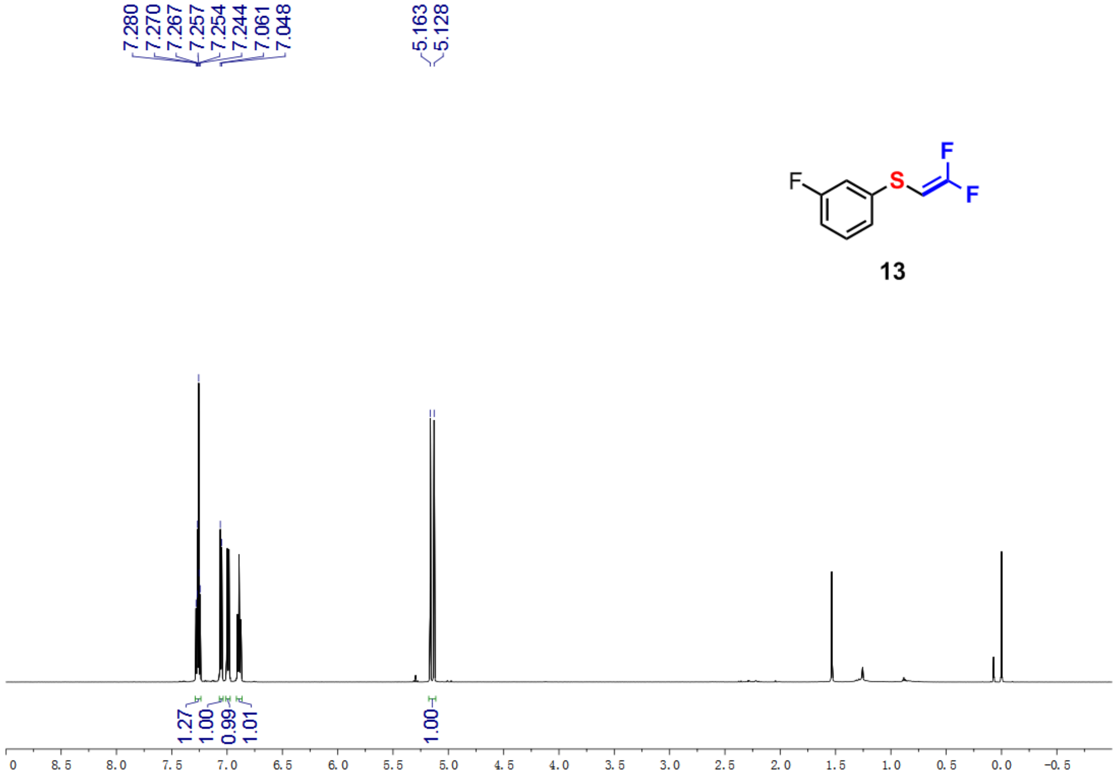

Supplementary Figure 34. ^{1}H NMR of 13

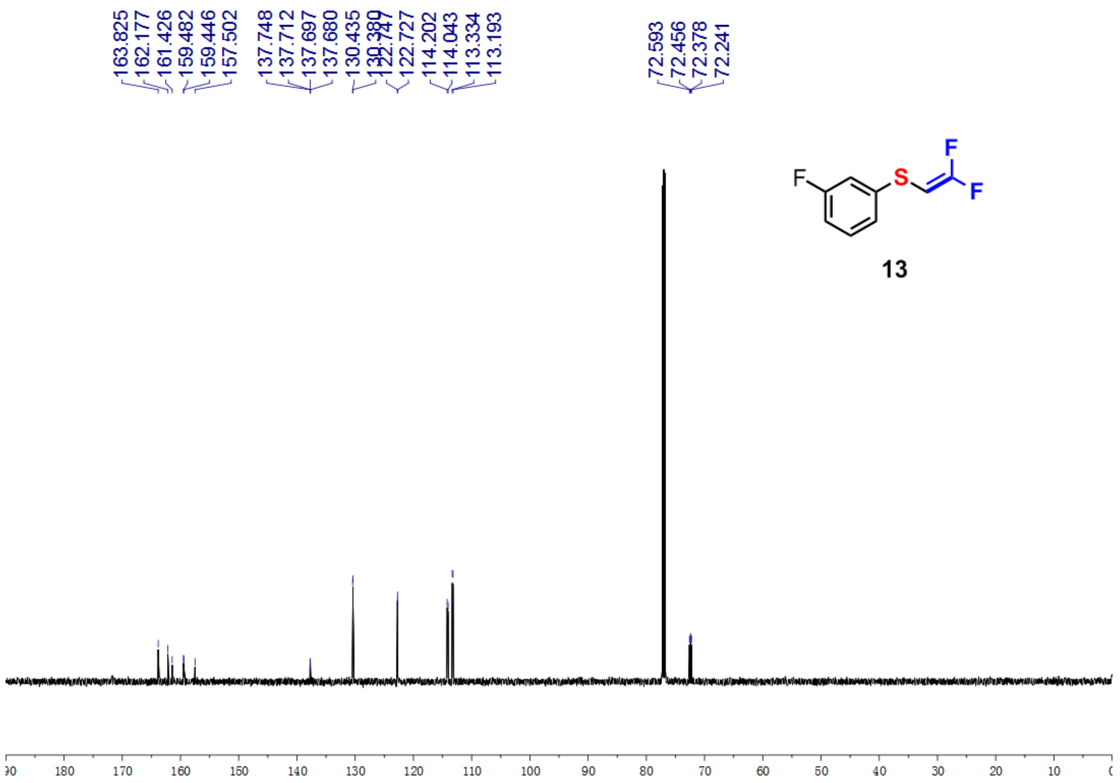

Supplementary Figure 35. ^{13}C NMR of 13

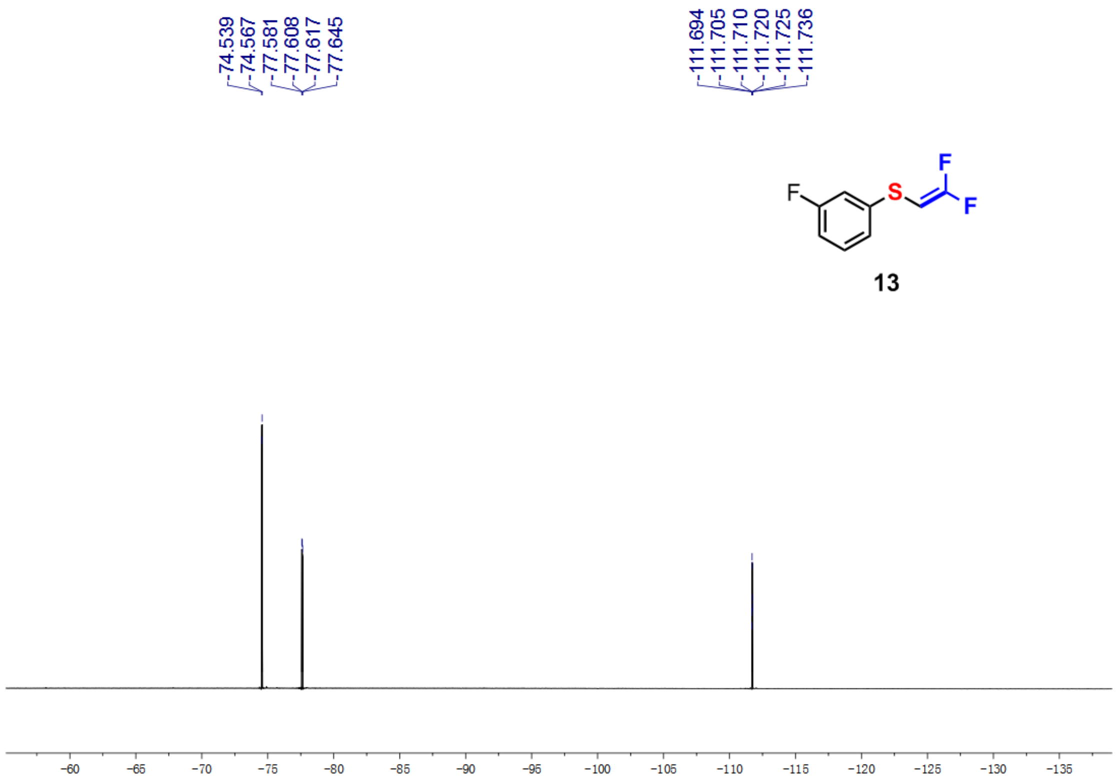

Supplementary Figure 36. ^{19}F NMR of 13

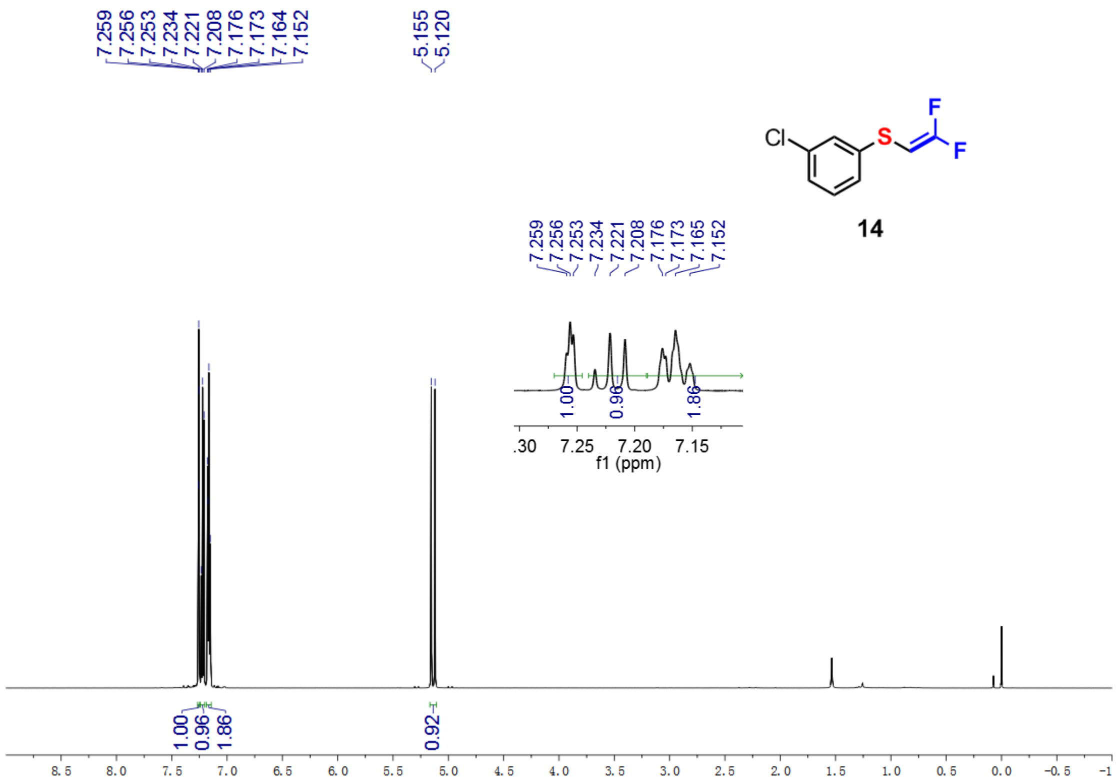

Supplementary Figure 37. ^{1}H NMR of 14

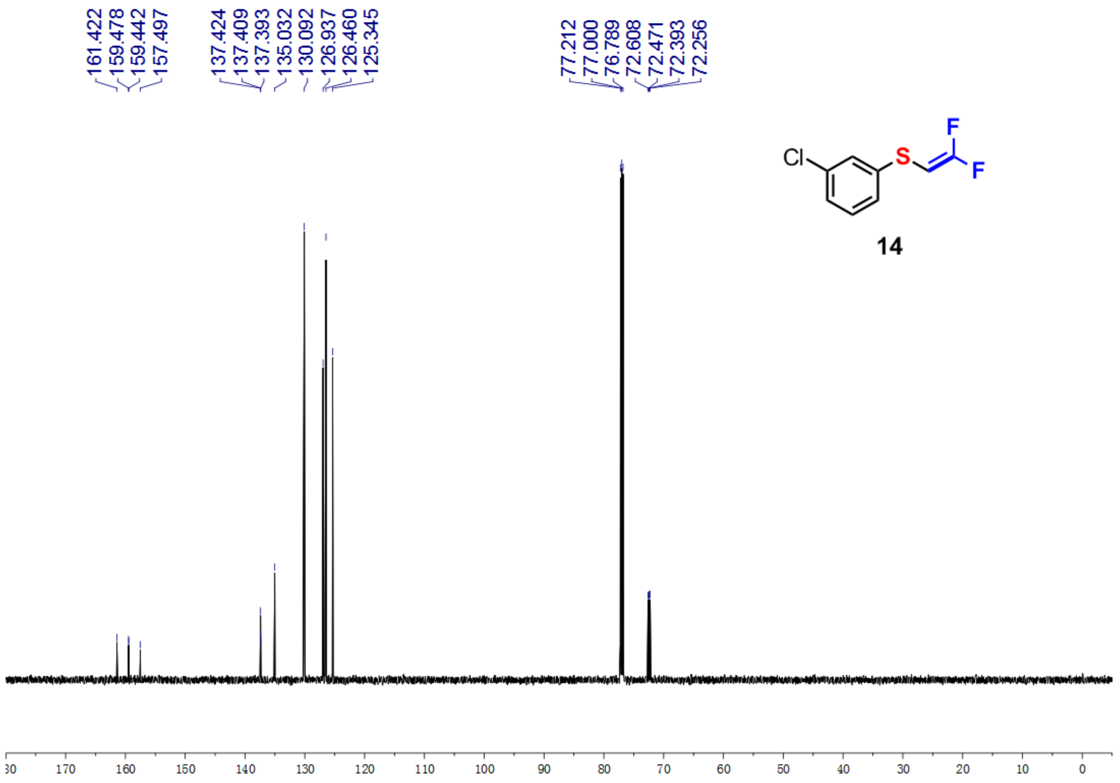

Supplementary Figure 38. ^{13}C NMR of 14

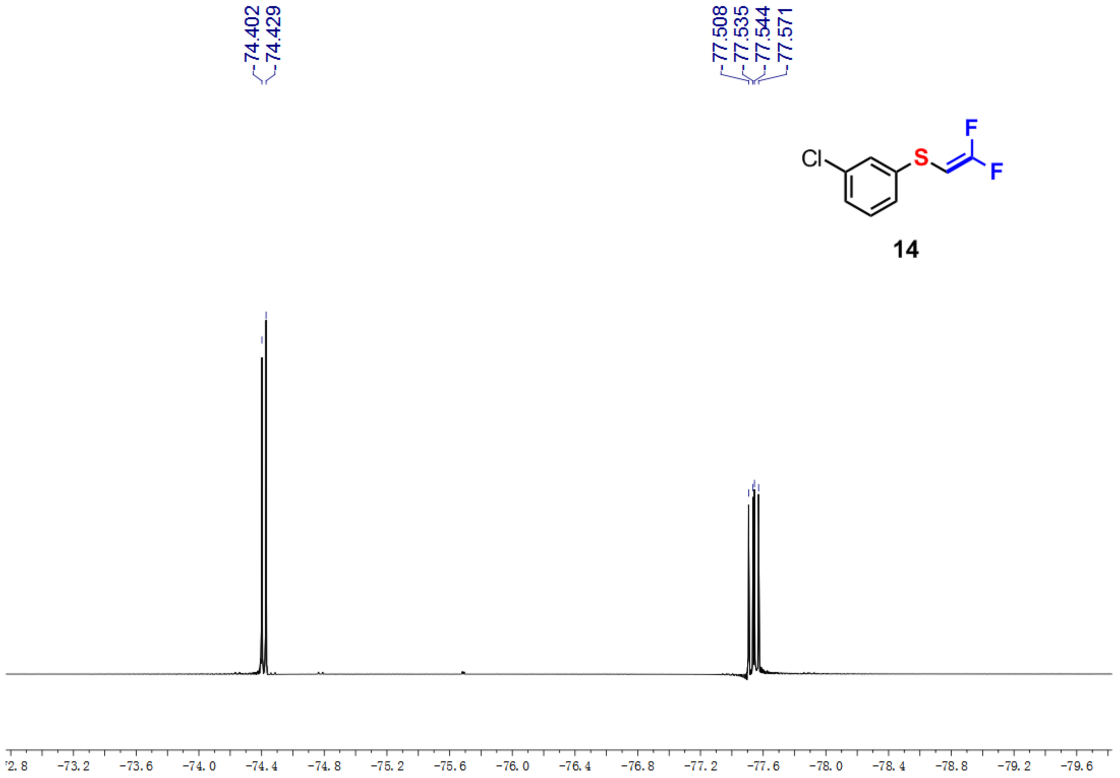

Supplementary Figure 39. ^{19}F NMR of 14

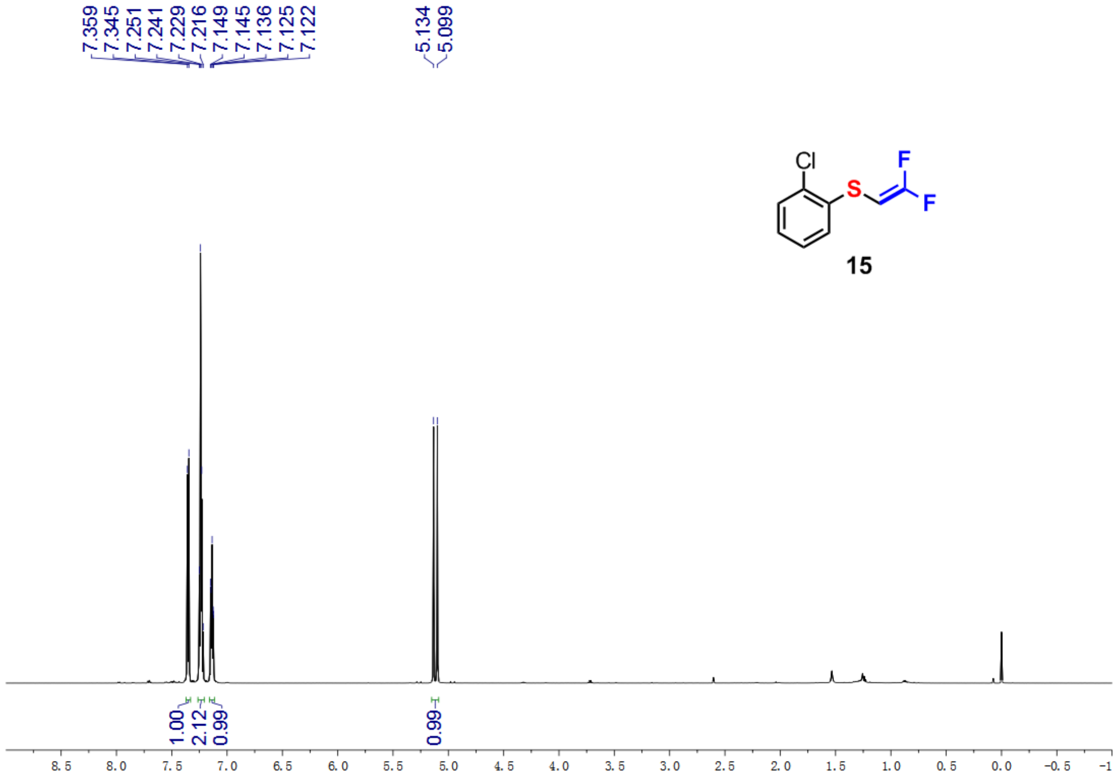

Supplementary Figure 40. ^{1}H NMR of 15

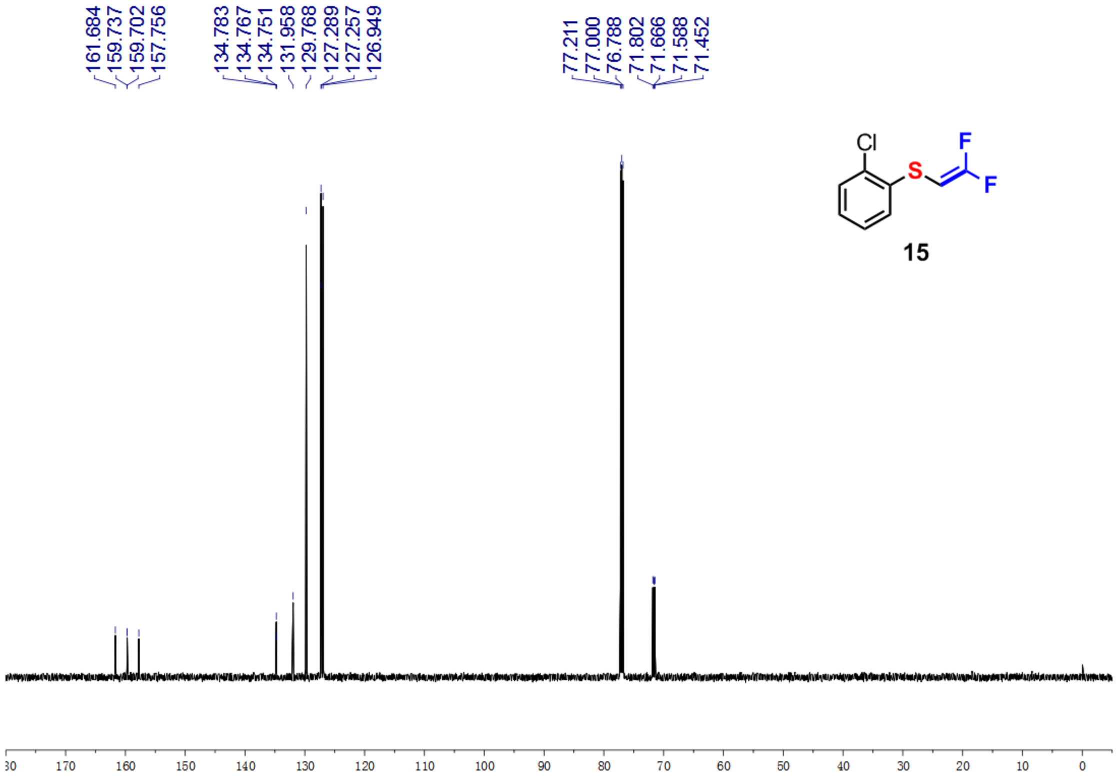

Supplementary Figure 41. ^{13}C NMR of 15

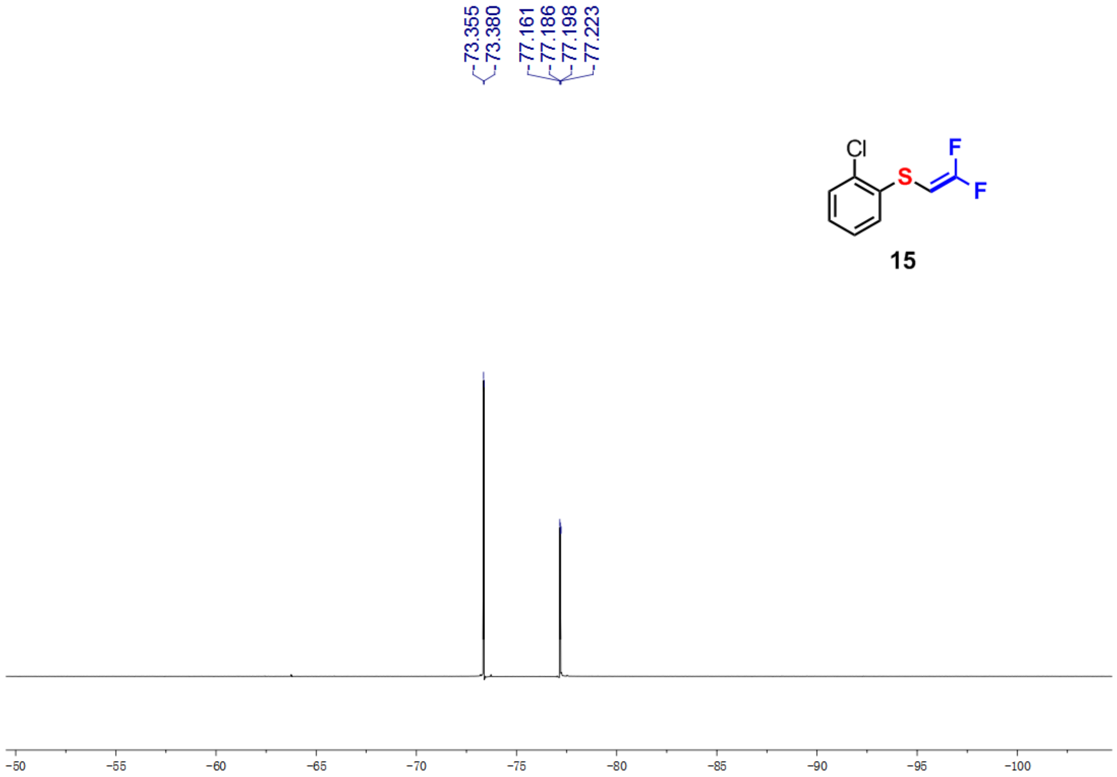

Supplementary Figure 42. ^{19}F NMR of 15

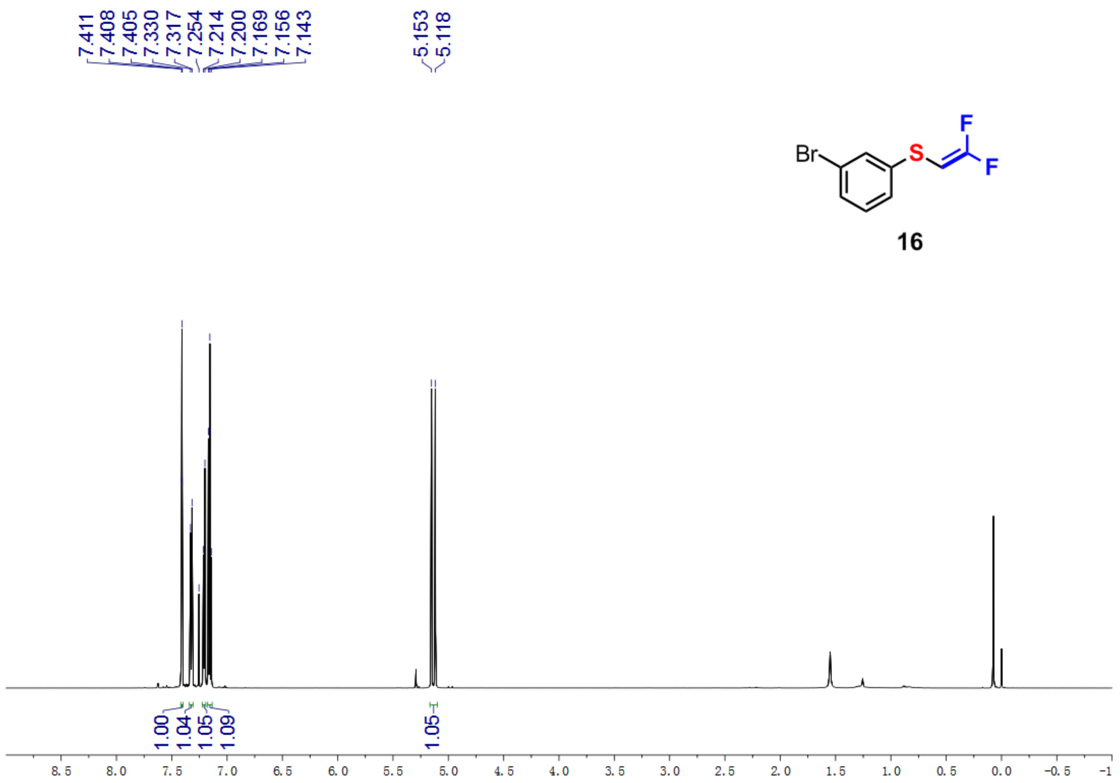

Supplementary Figure 43. ^{1}H NMR of 16

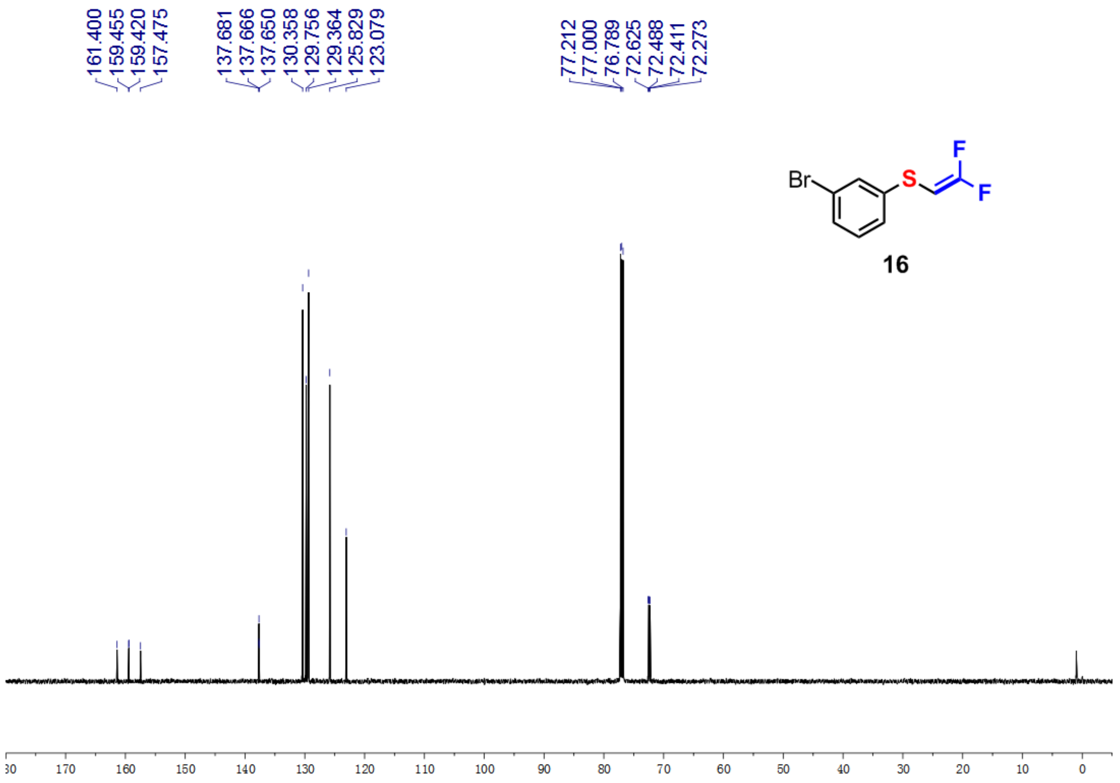

Supplementary Figure 44. ^{13}C NMR of 16

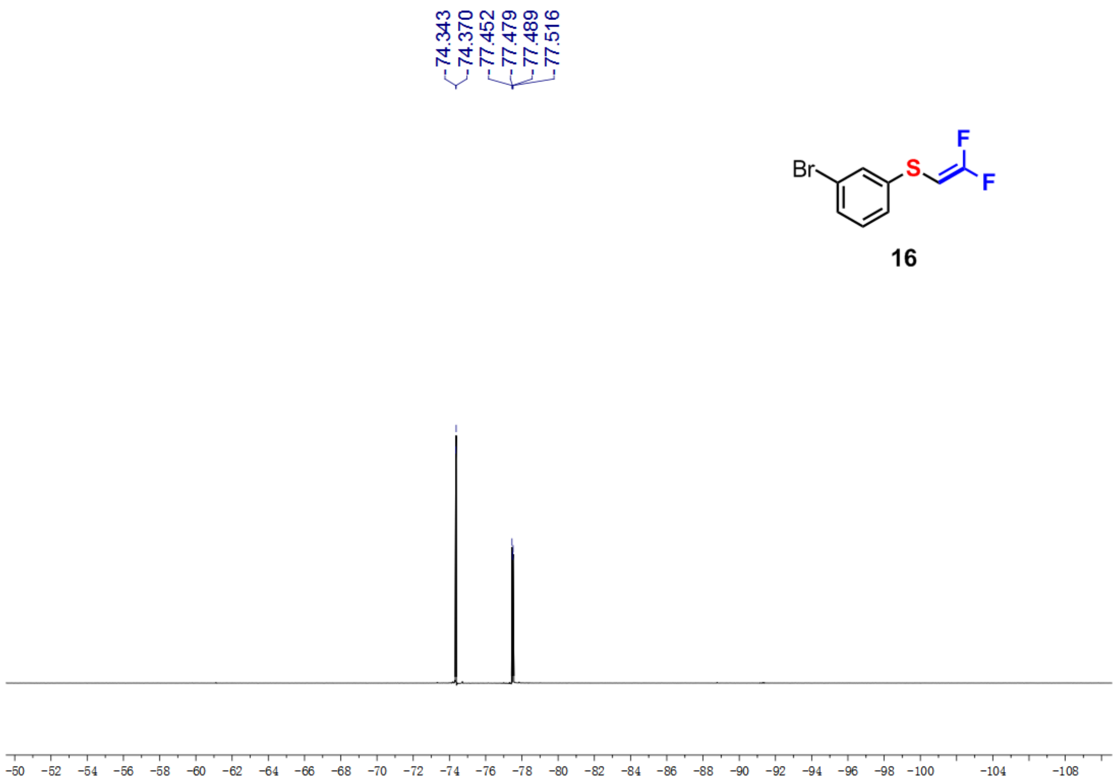

Supplementary Figure 45. ^{19}F NMR of 16

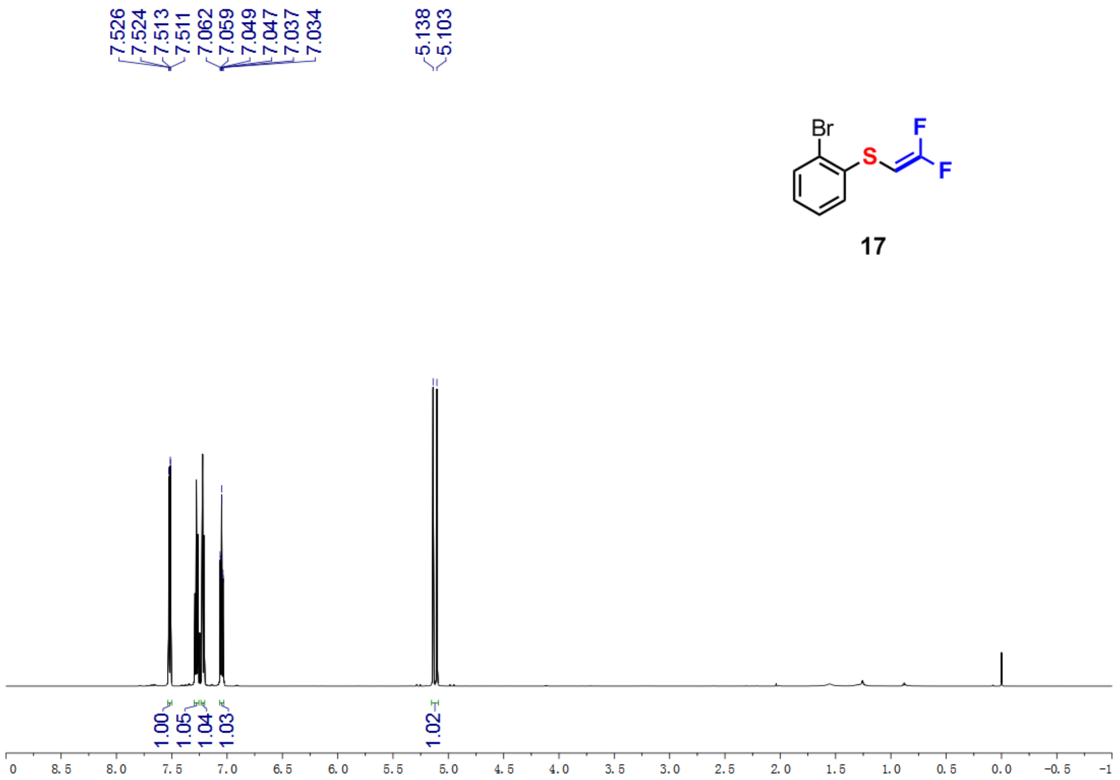

Supplementary Figure 46. ^{1}H NMR of 17

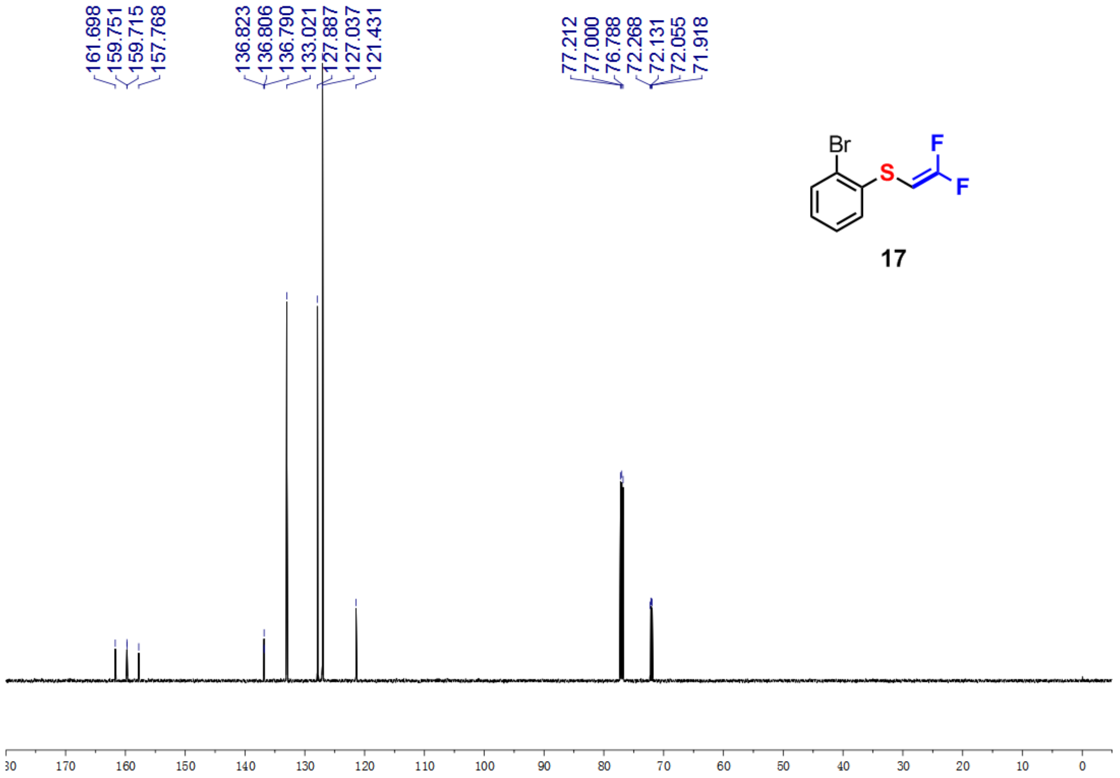

Supplementary Figure 47. ^{13}C NMR of 17

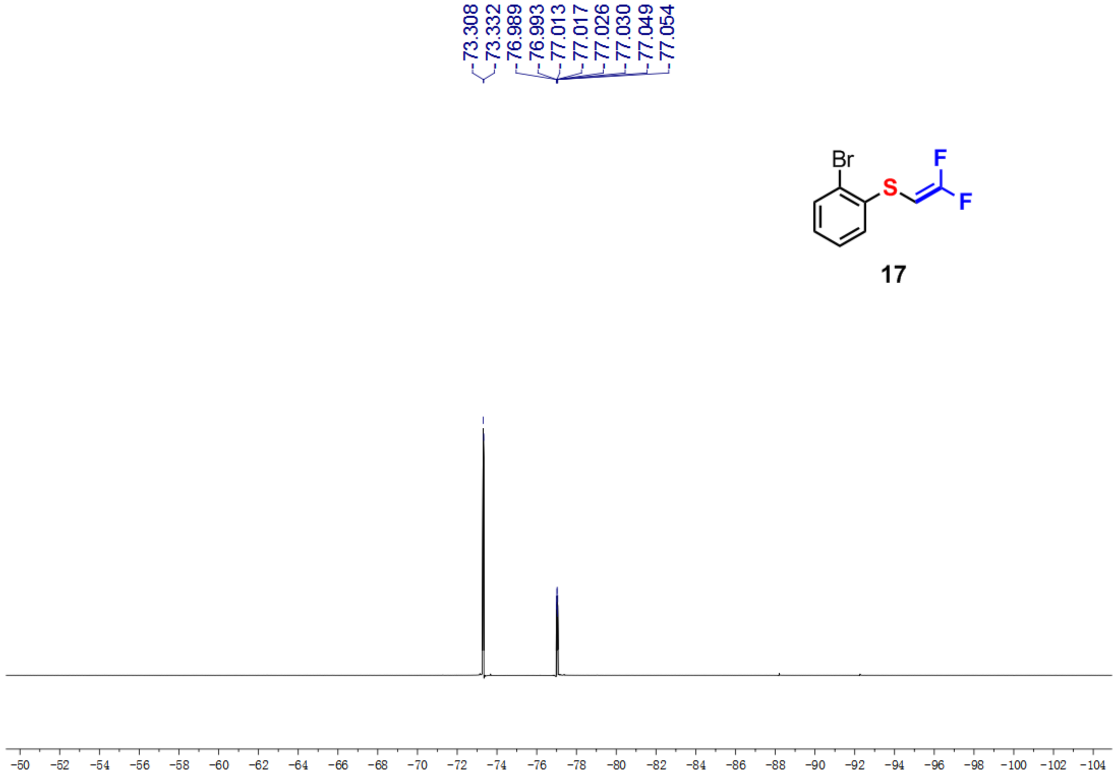

Supplementary Figure 48. ^{19}F NMR of 17

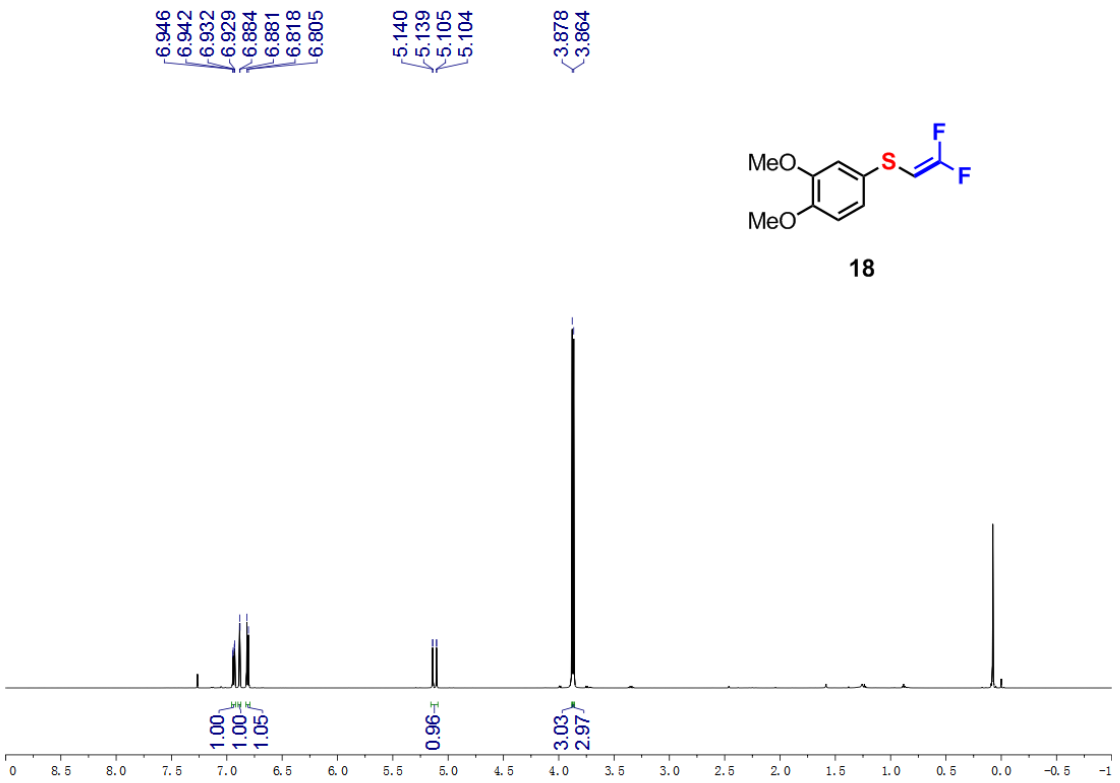

Supplementary Figure 49. ^{1}H NMR of 18

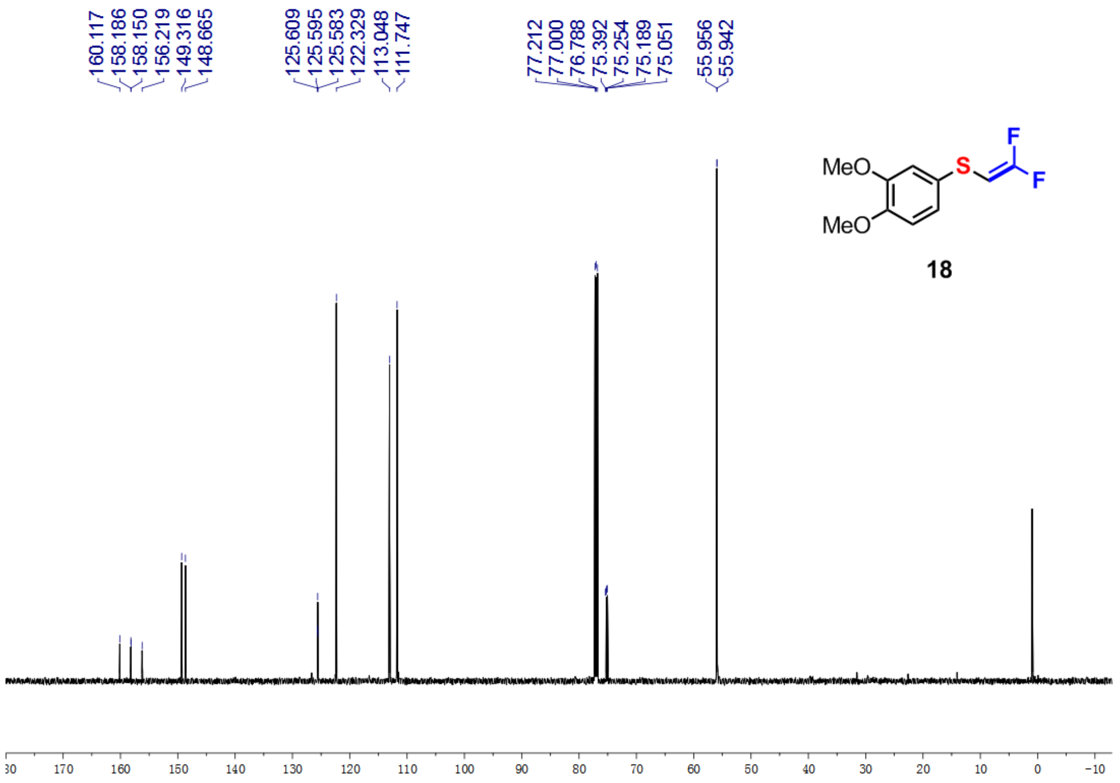

Supplementary Figure 50. ^{13}C NMR of 18

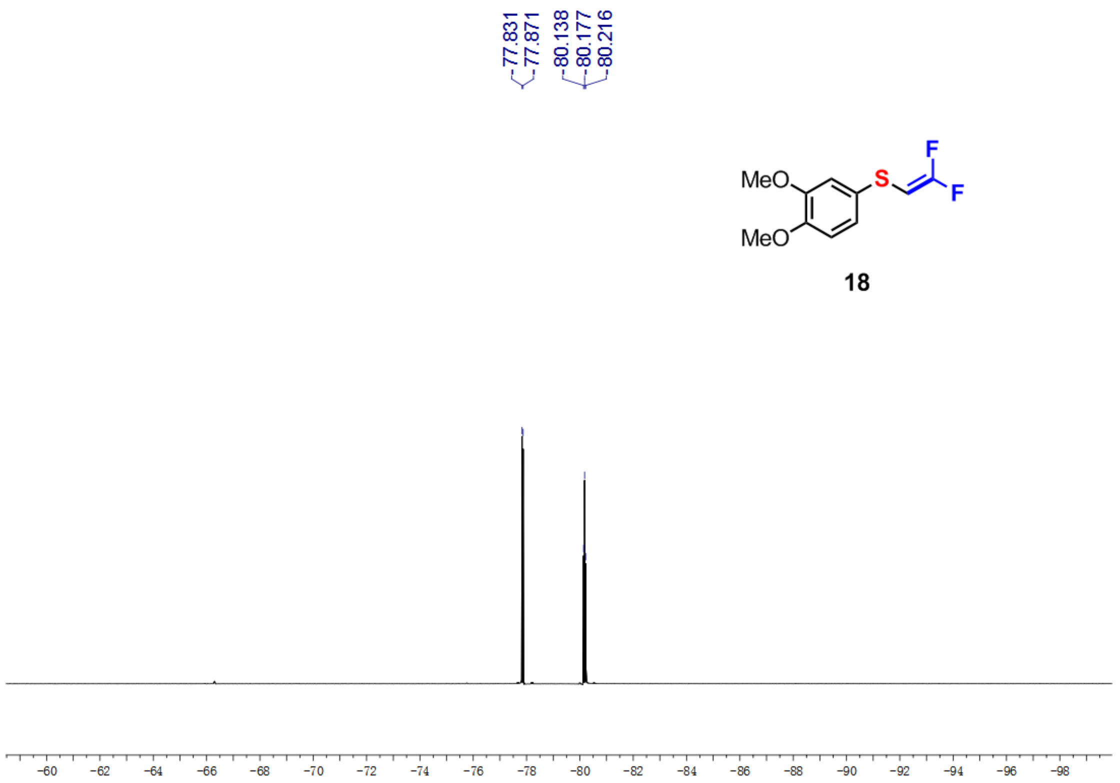

Supplementary Figure 51. ^{19}F NMR of 18

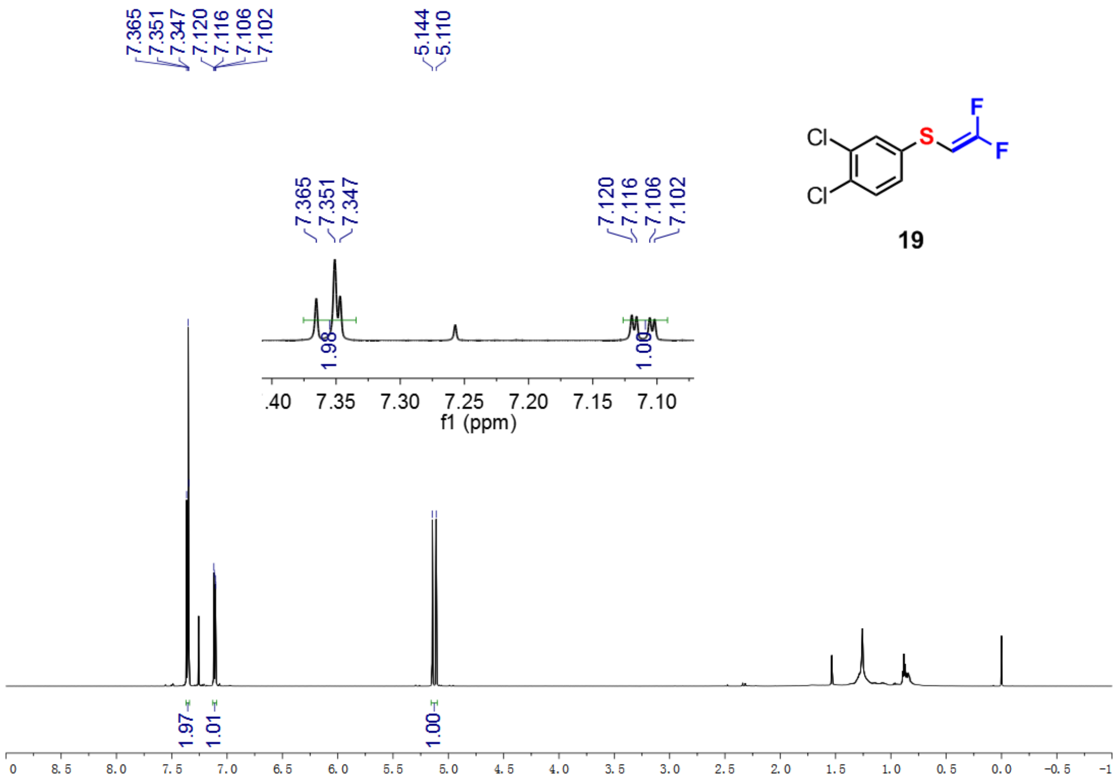

Supplementary Figure 52. ^{1}H NMR of 19

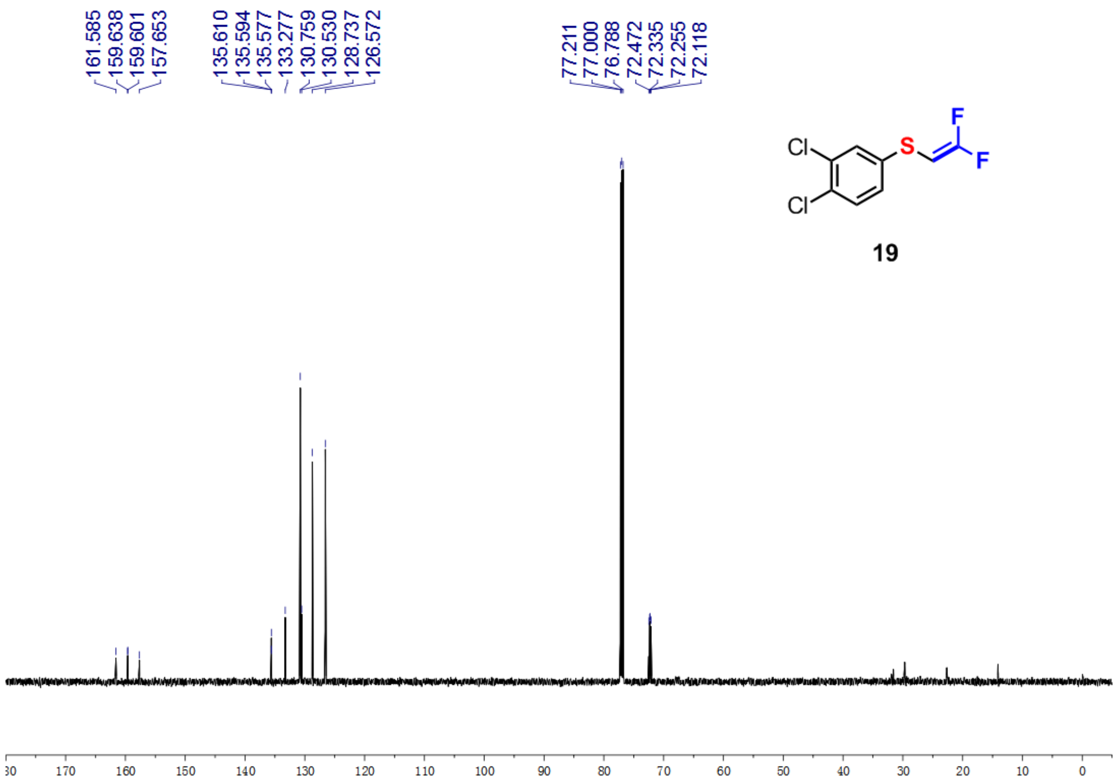

Supplementary Figure 53. ^{13}C NMR of 19

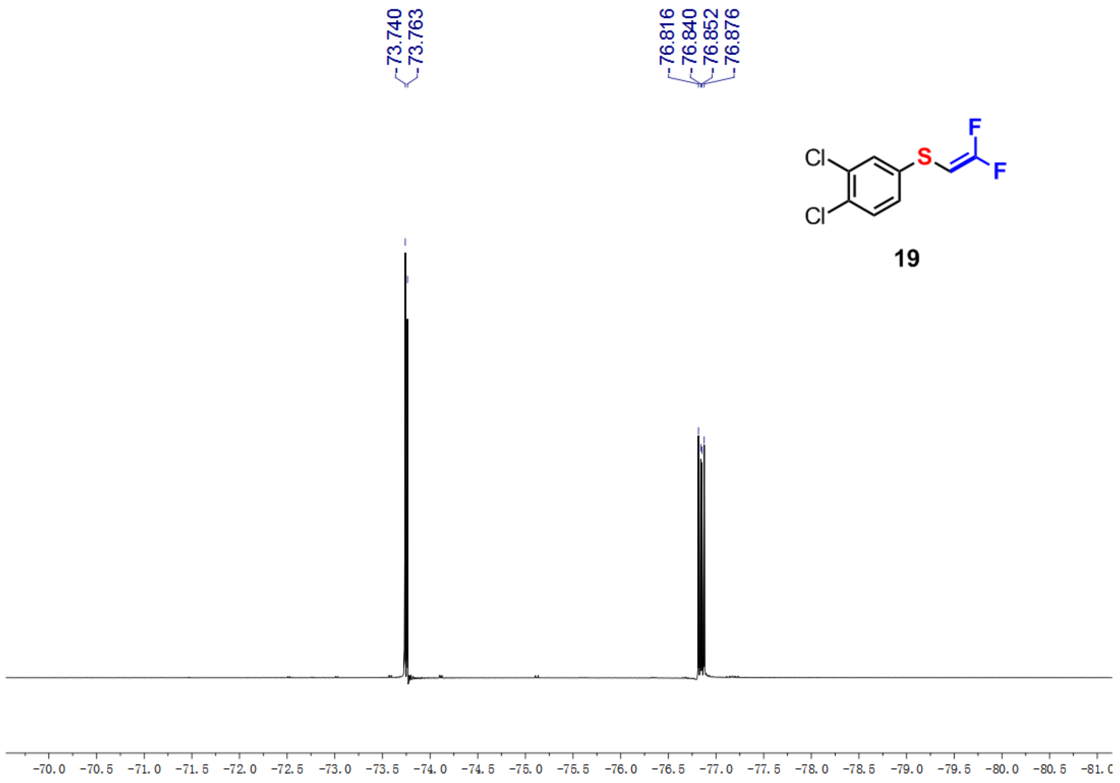

Supplementary Figure 54. ^{19}F NMR of 19

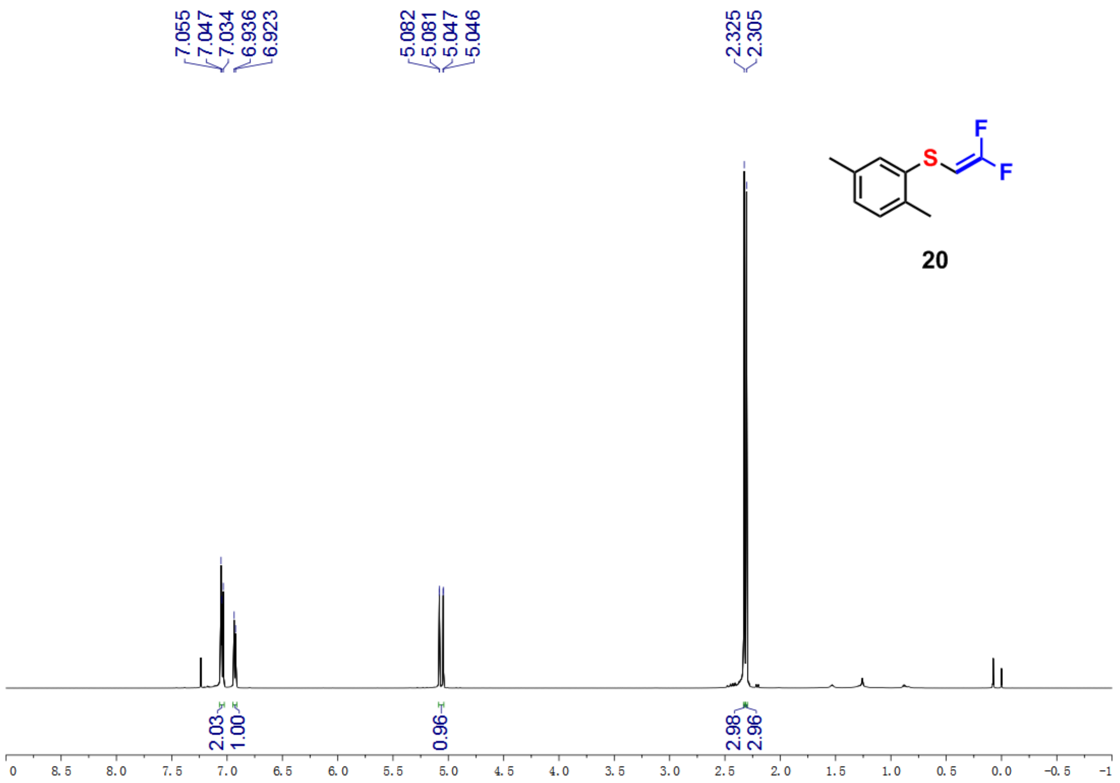

Supplementary Figure 55. ^{1}H NMR of 20

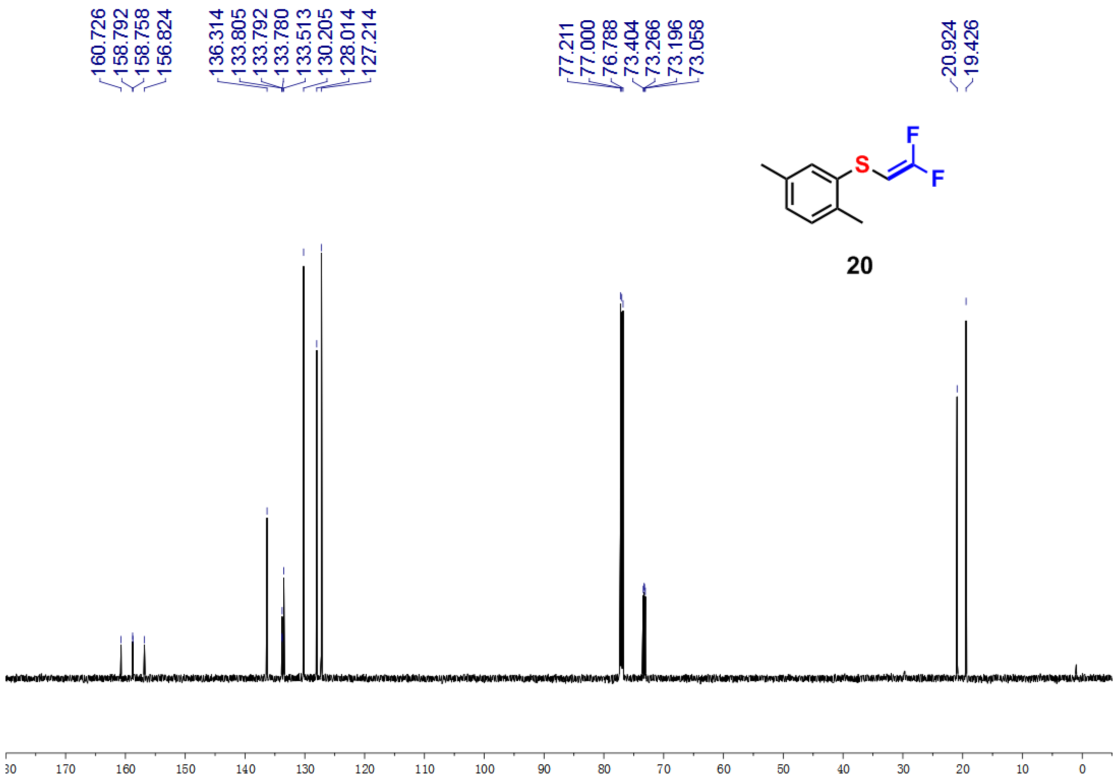

Supplementary Figure 56. ^{13}C NMR of 20

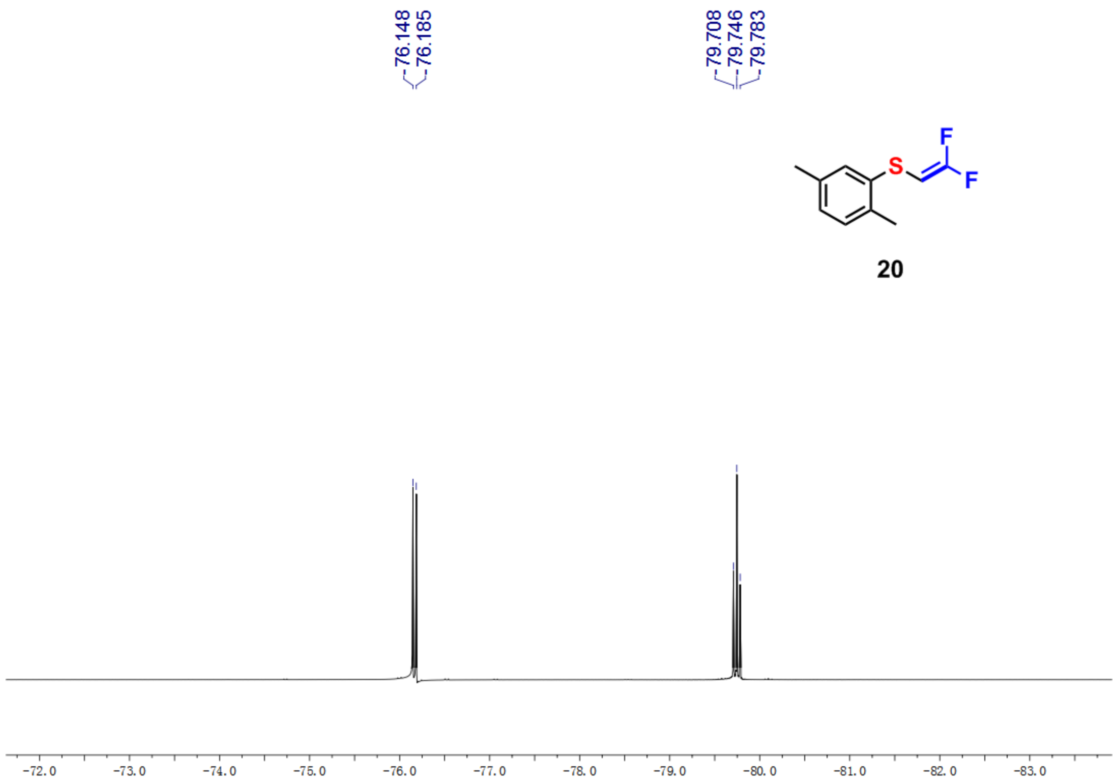

Supplementary Figure 57. ^{19}F NMR of 20

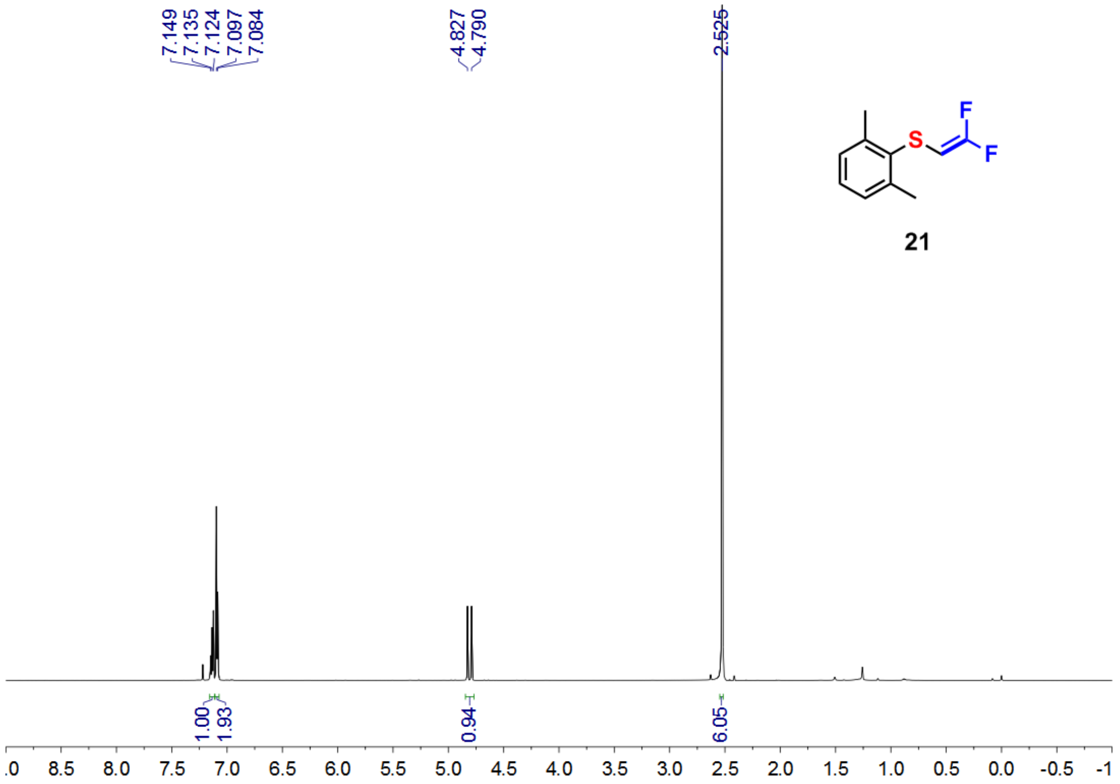

Supplementary Figure 58. ^{1}H NMR of 21

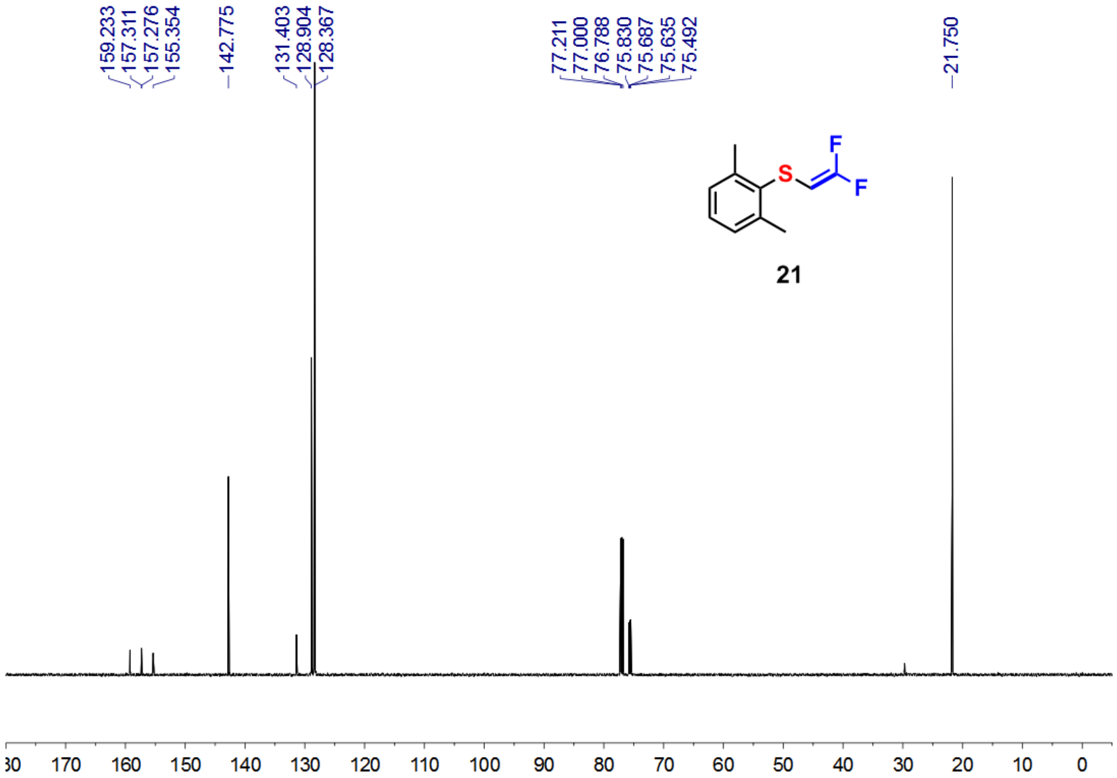

Supplementary Figure 59. ^{13}C NMR of 21

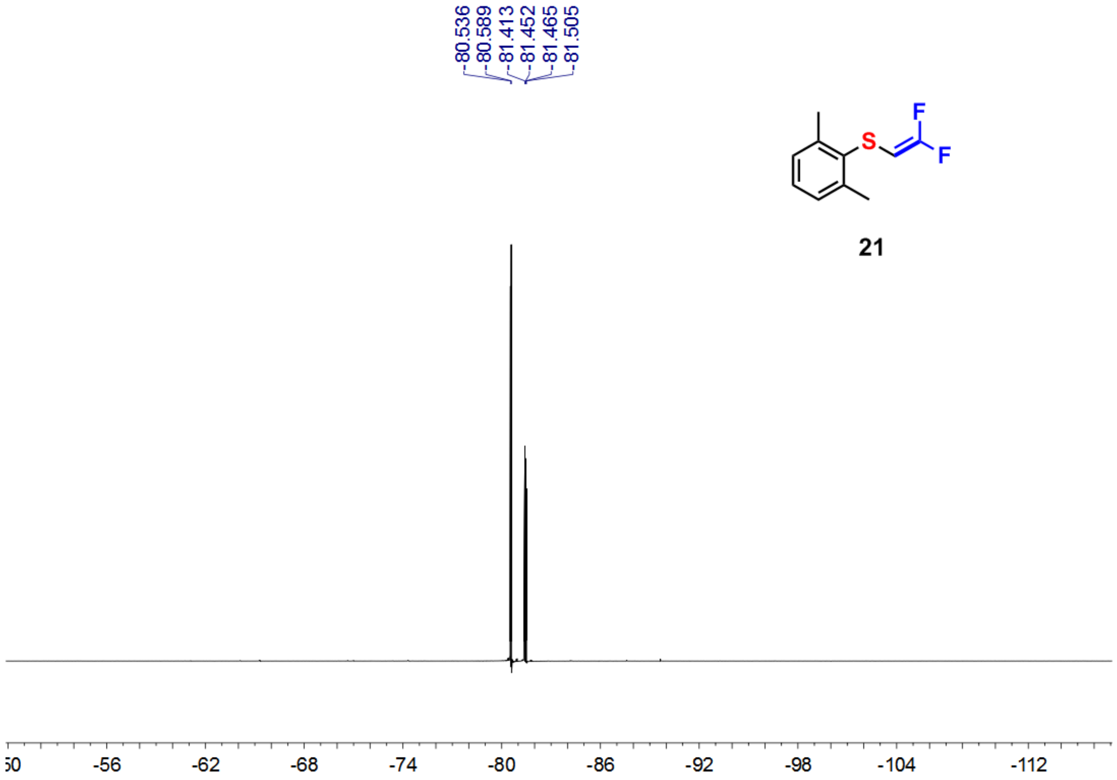

Supplementary Figure 60. ^{19}F NMR of 21

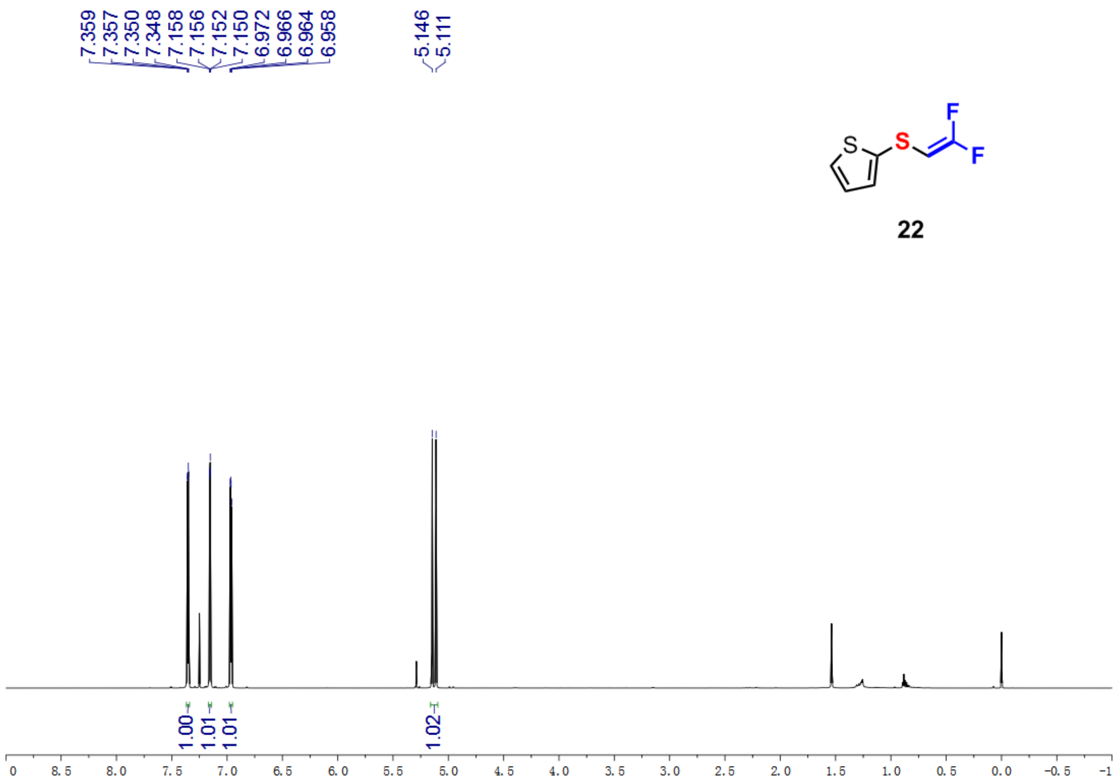

Supplementary Figure 61. ^{1}H NMR of 22

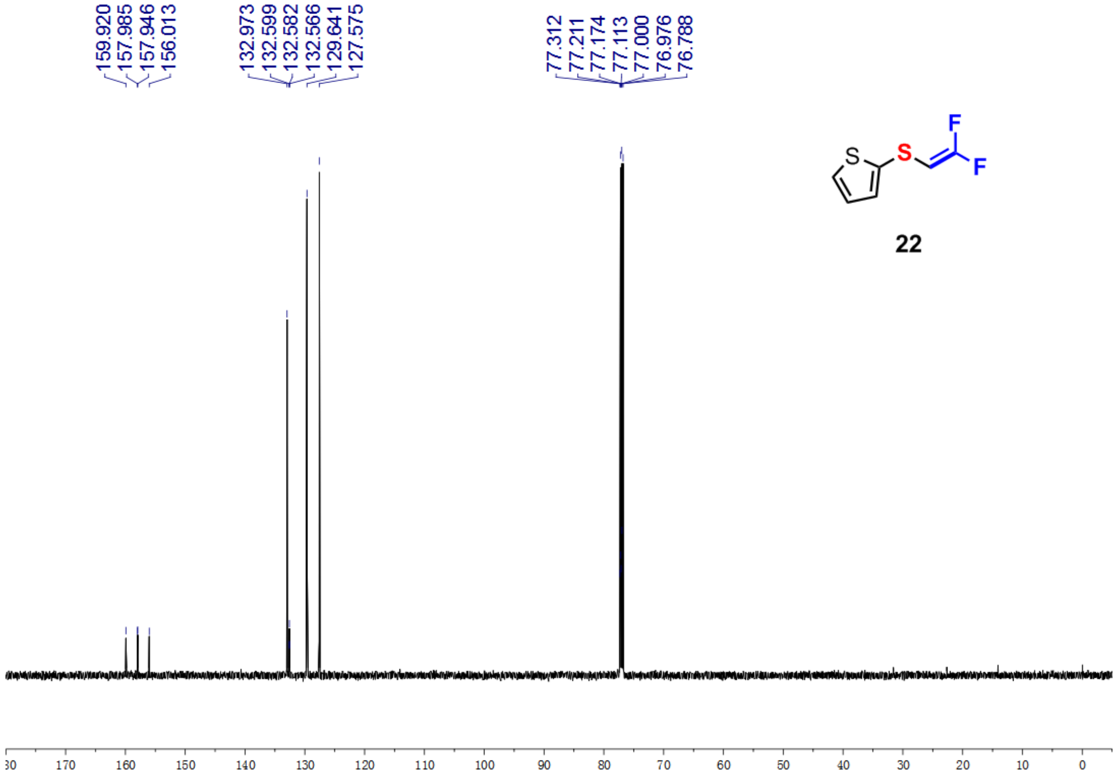

Supplementary Figure 62. ^{13}C NMR of 22

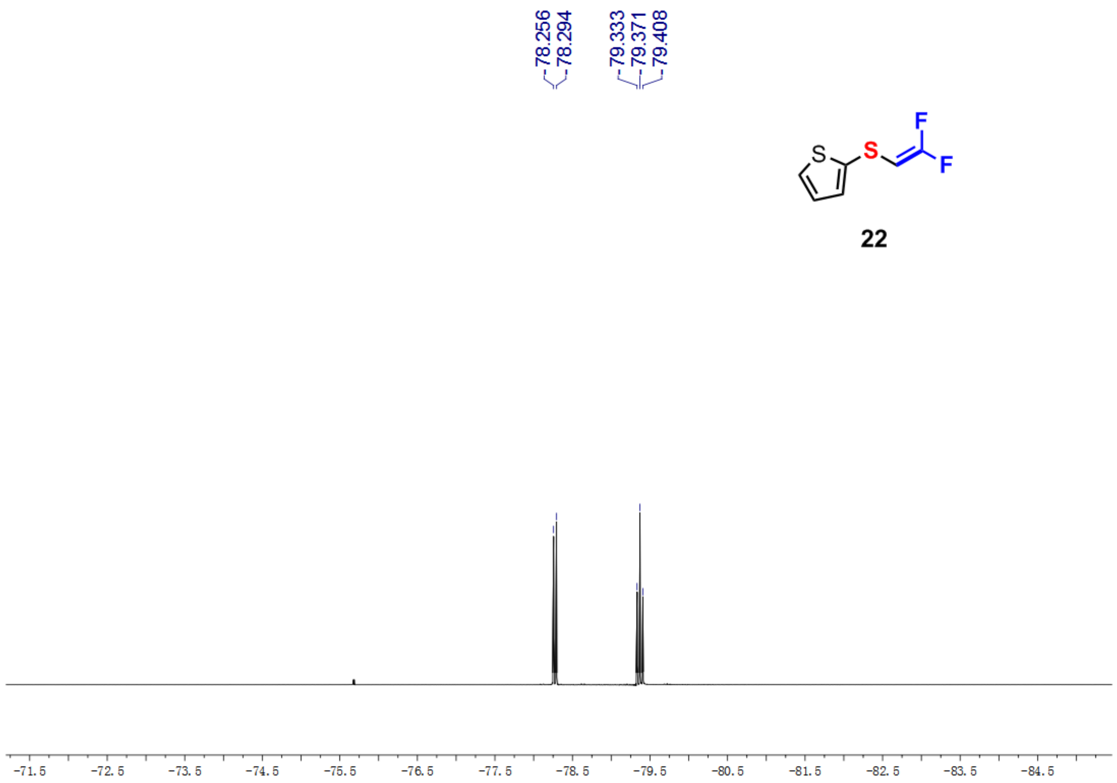

Supplementary Figure 63. ^{19}F NMR of 22

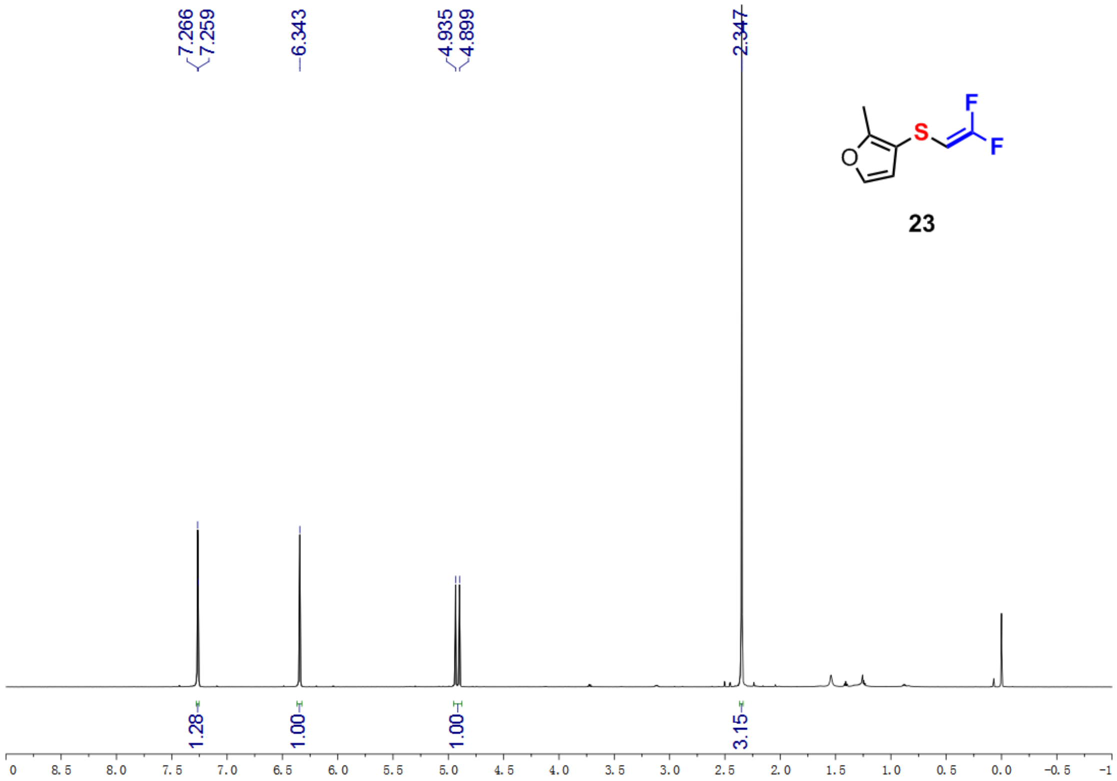

Supplementary Figure 64. ^{1}H NMR of 23

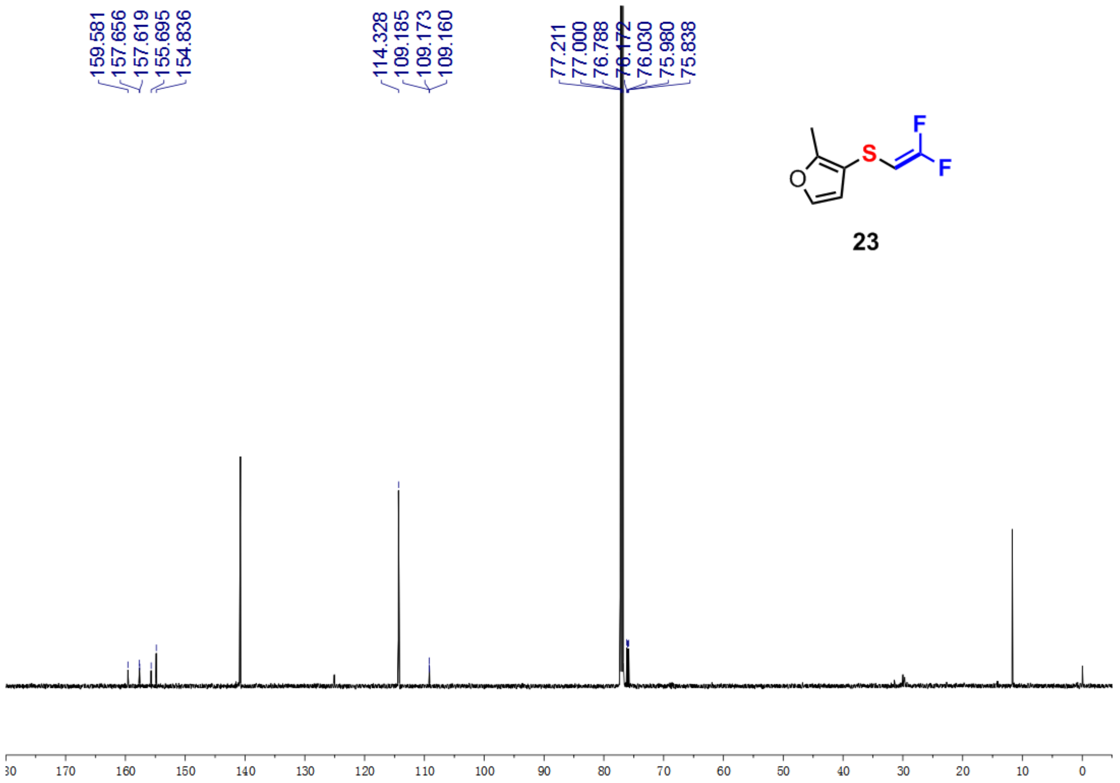

Supplementary Figure 65. ^{13}C NMR of 23

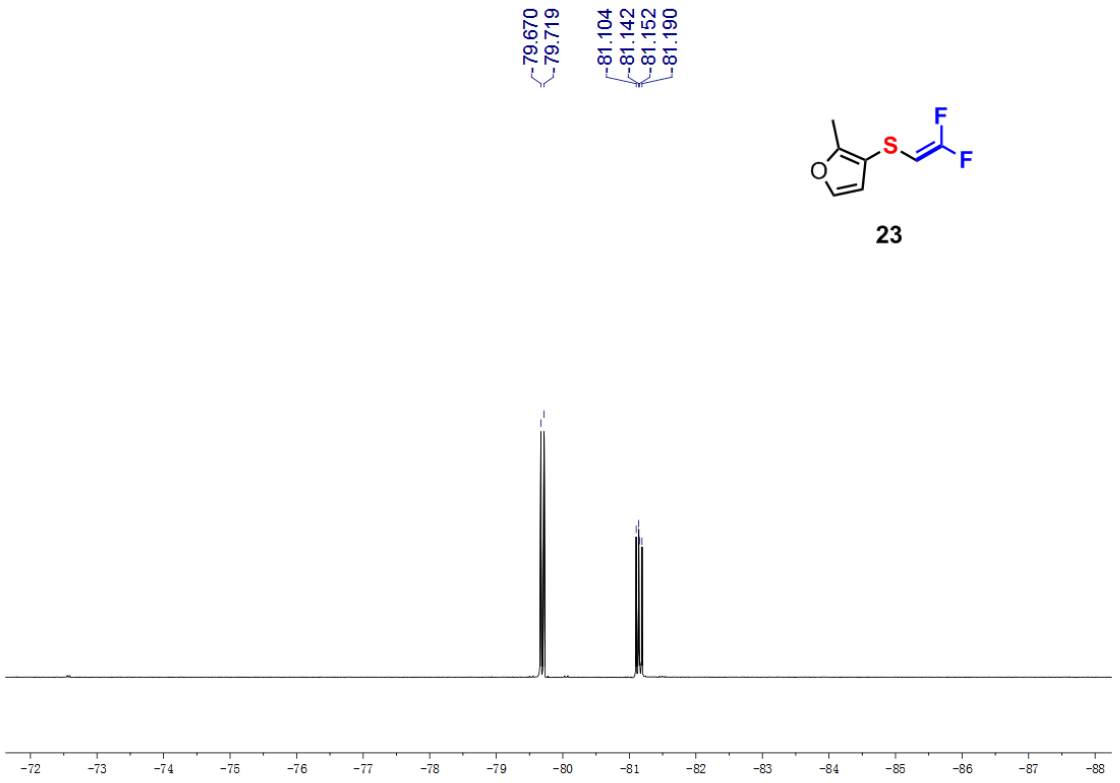

Supplementary Figure 66. ^{19}F NMR of 23

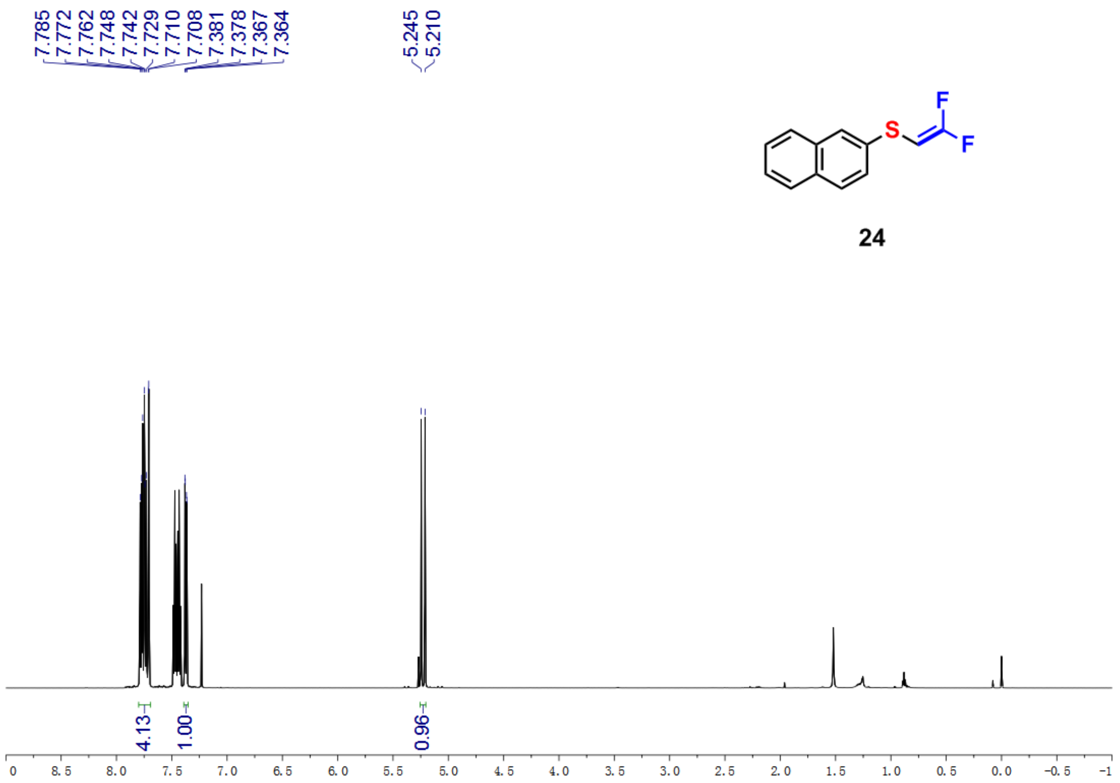

Supplementary Figure 67. ^{1}H NMR of 24

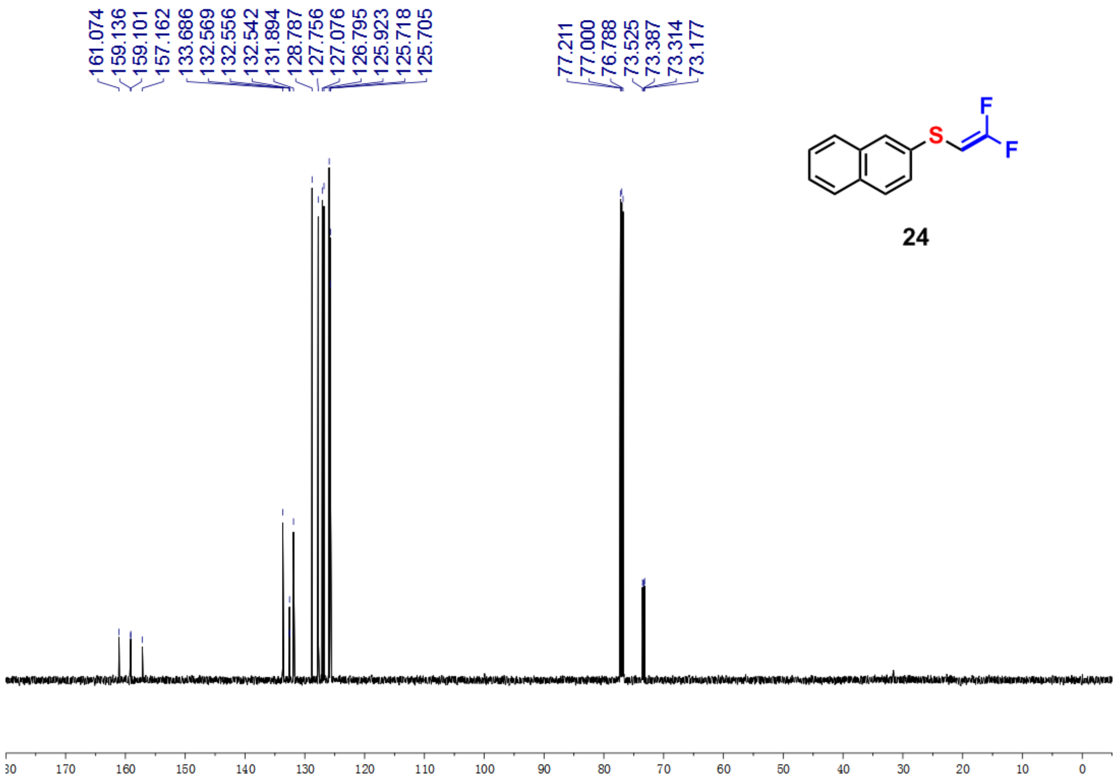

Supplementary Figure 68. ^{13}C NMR of 24

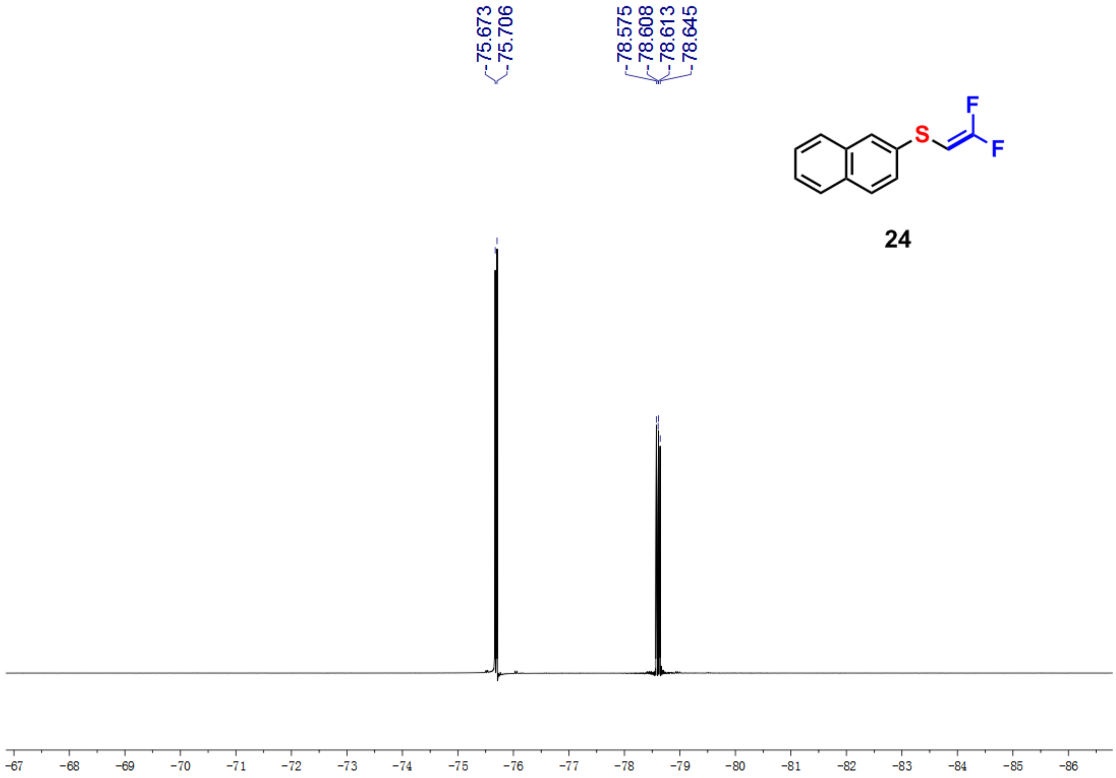

Supplementary Figure 69. ^{19}F NMR of 24

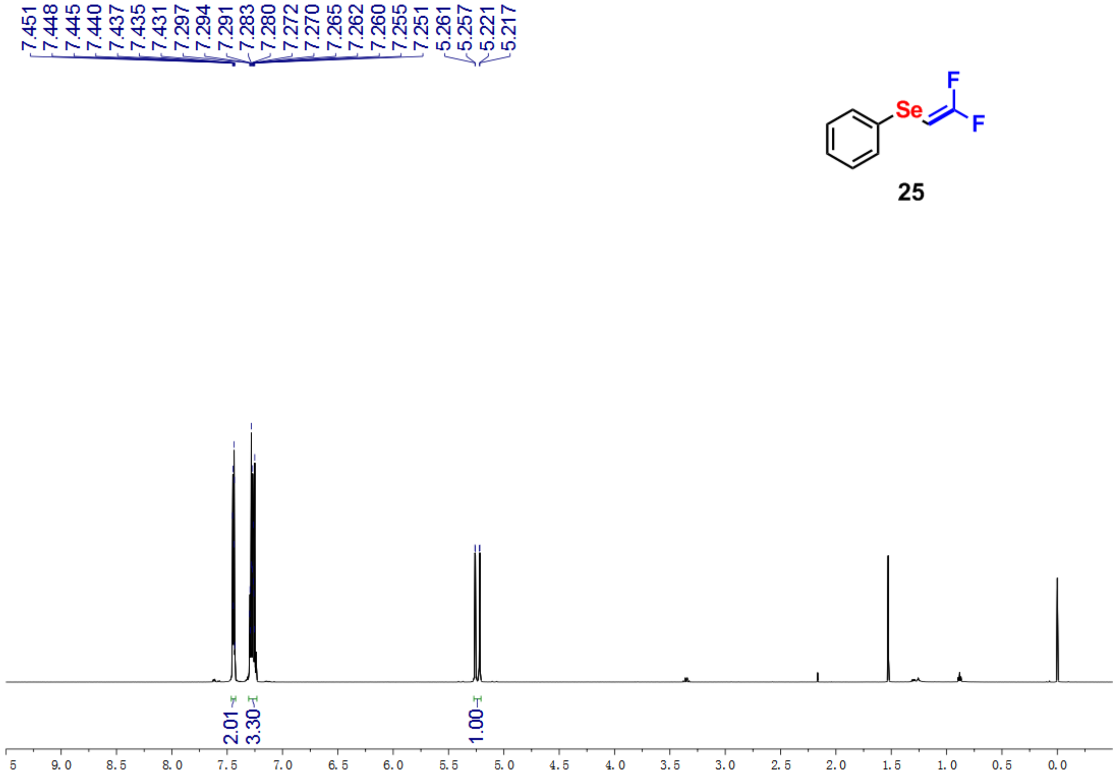

Supplementary Figure 70. ^{1}H NMR of 25

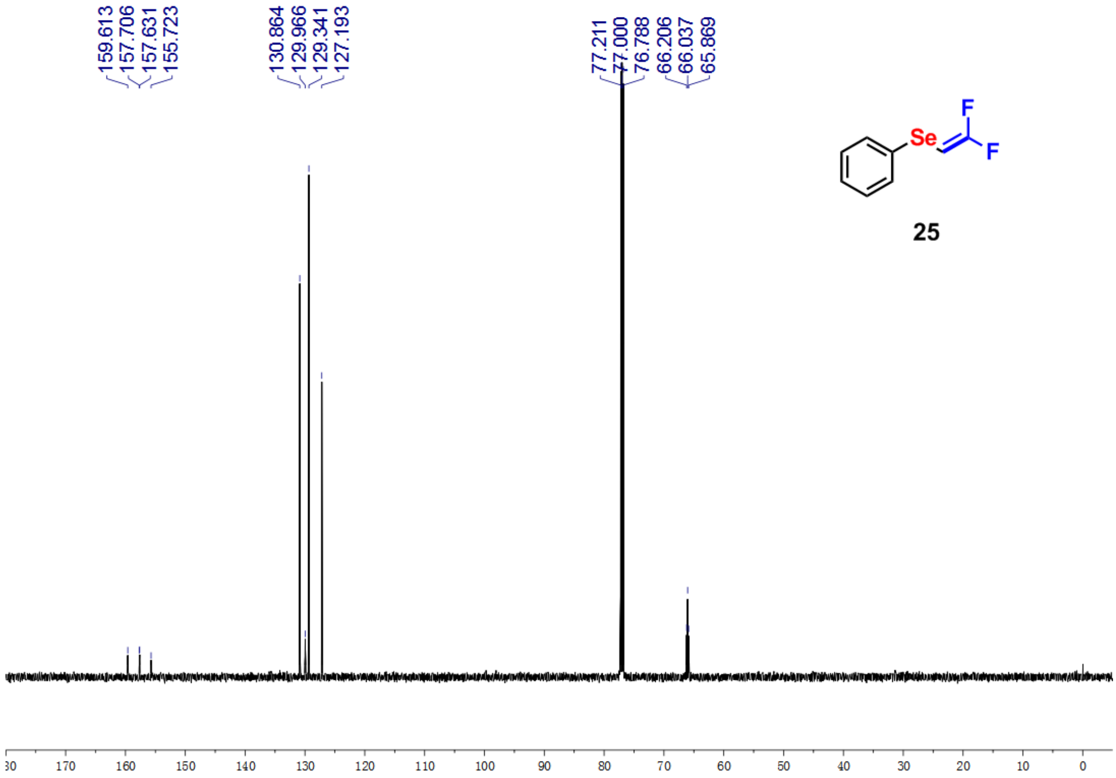

Supplementary Figure 71. ^{13}C NMR of 25

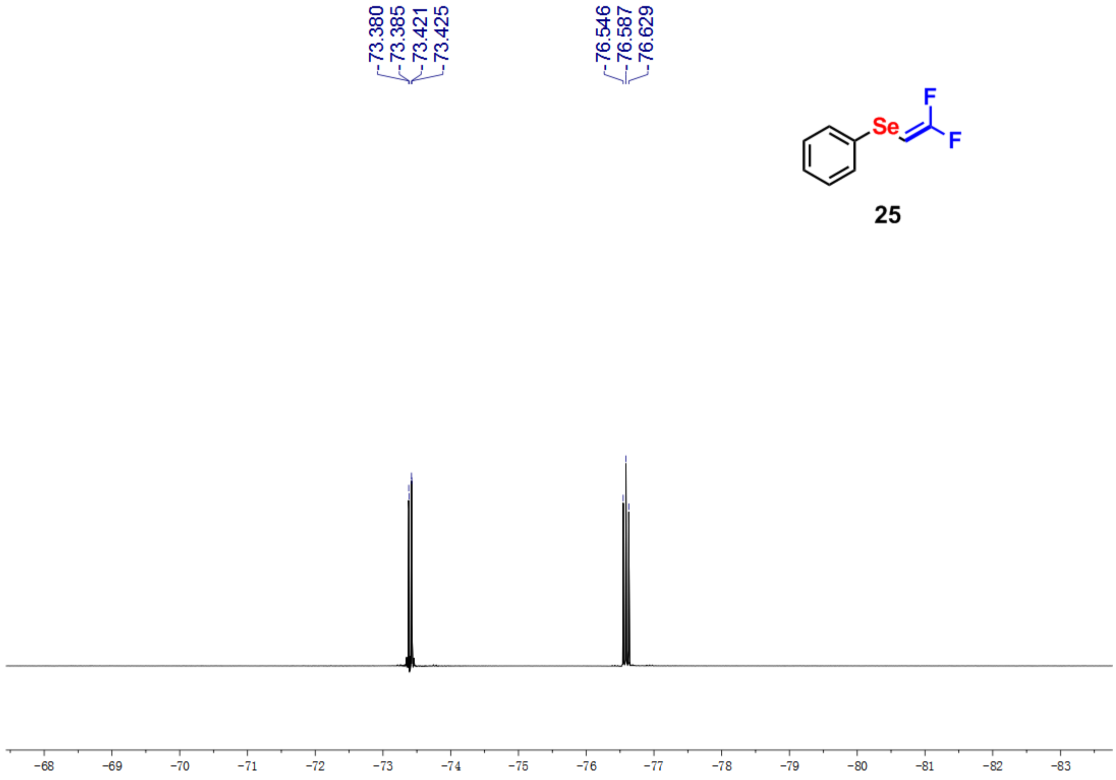

Supplementary Figure 72. ^{19}F NMR of 25

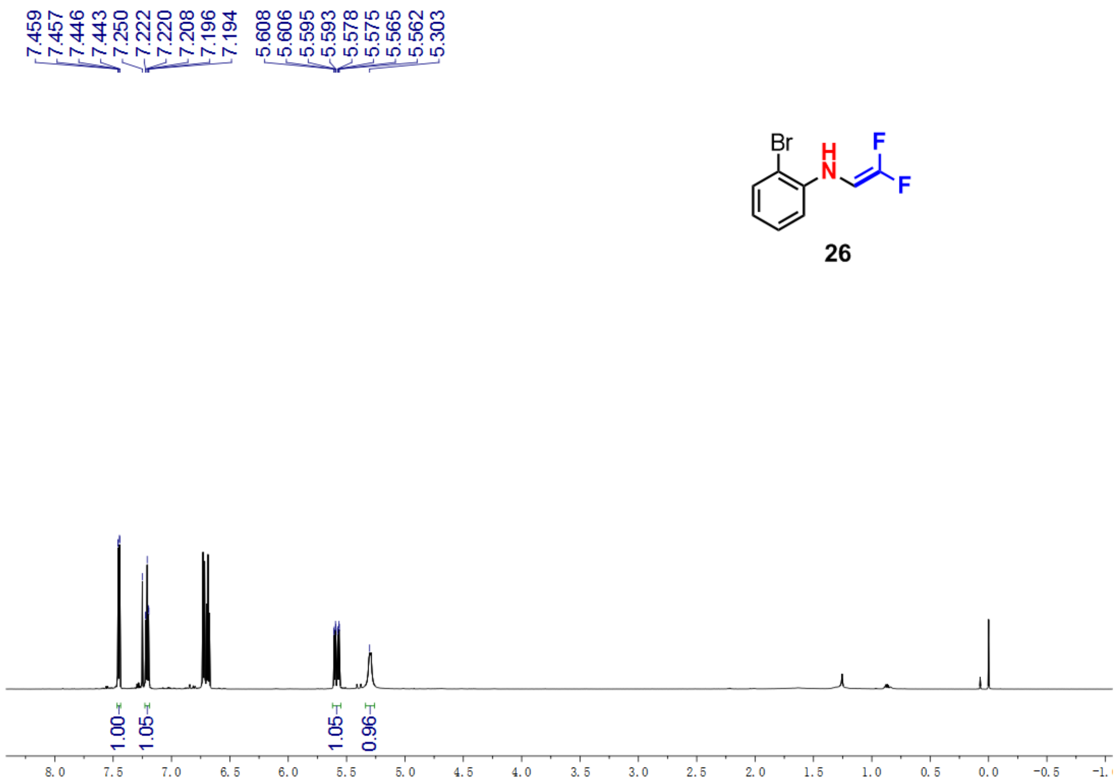

Supplementary Figure 73. ^{1}H NMR of 26

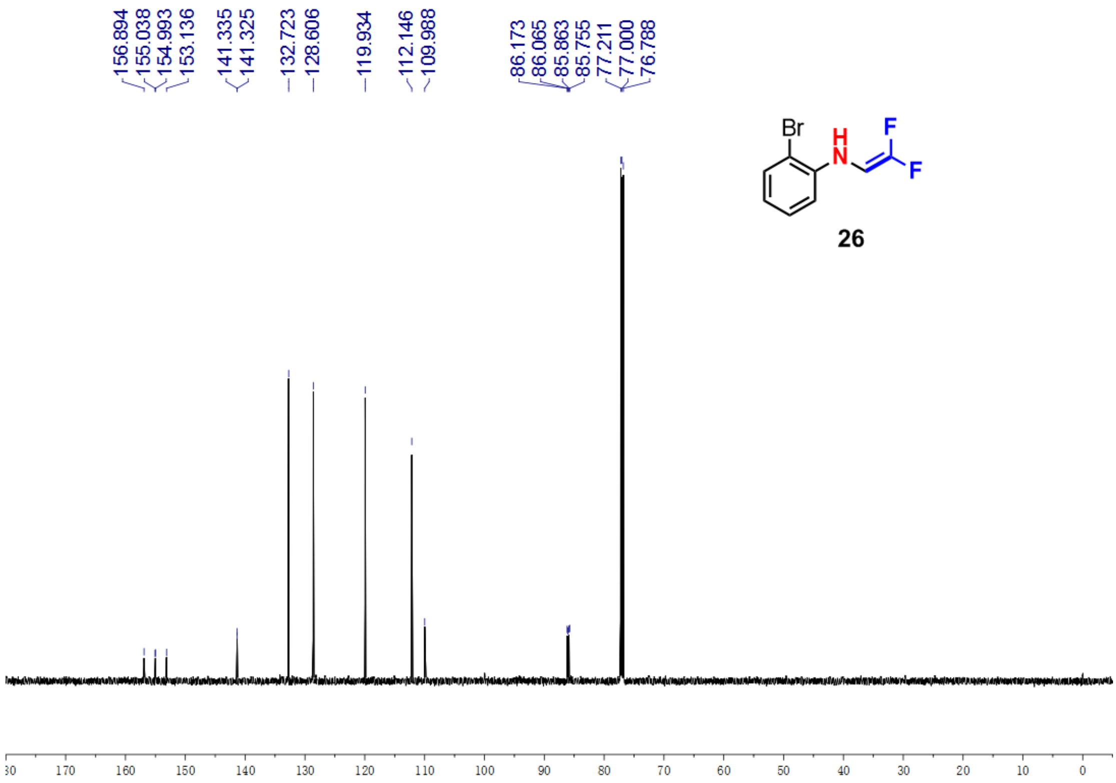

Supplementary Figure 74. ^{13}C NMR of 26

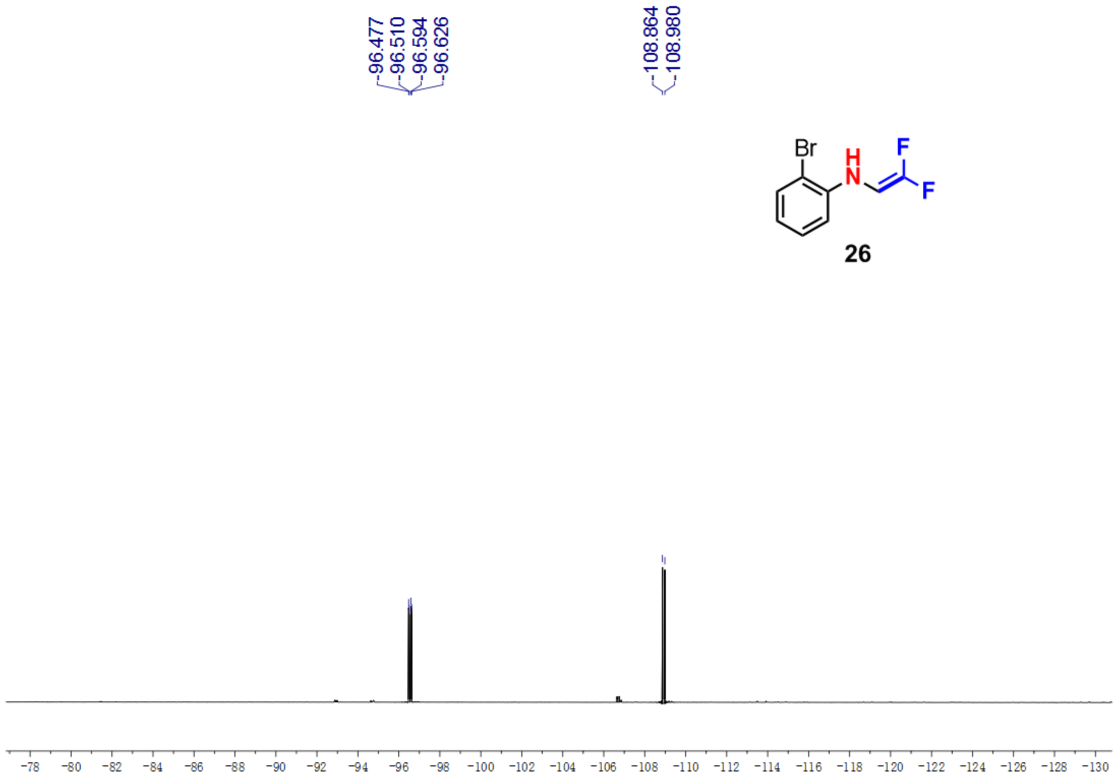

Supplementary Figure 75. ^{19}F NMR of 26

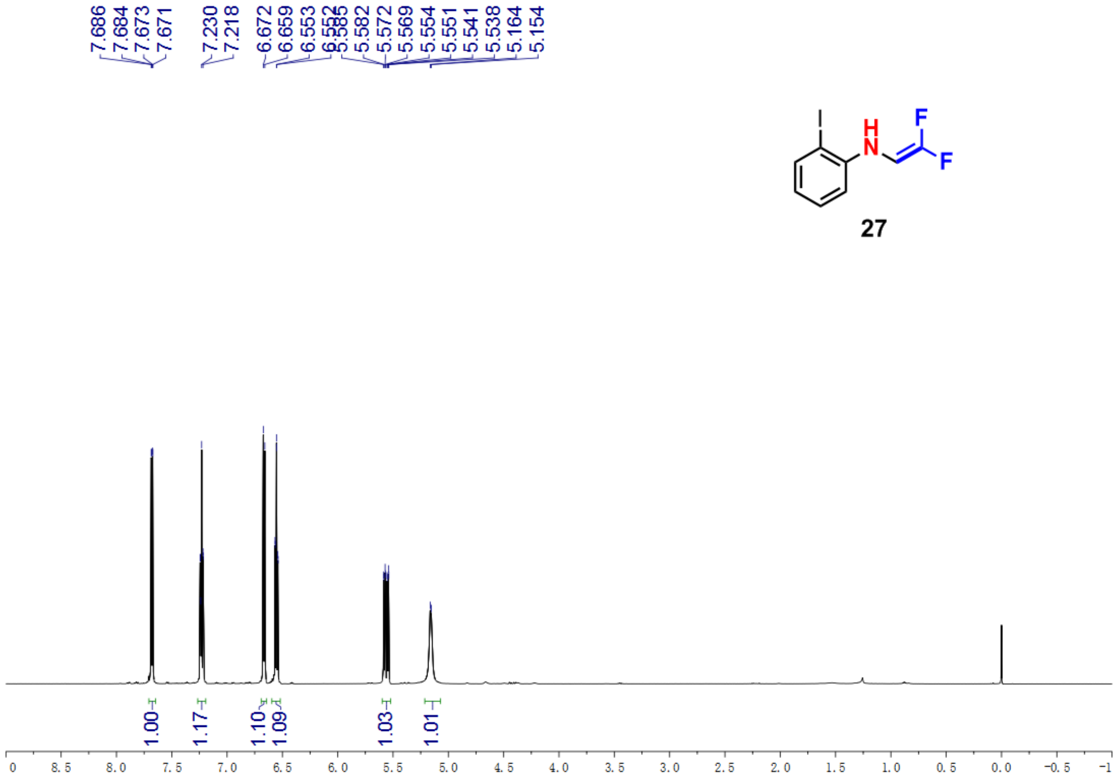

Supplementary Figure 76. ^{1}H NMR of 27

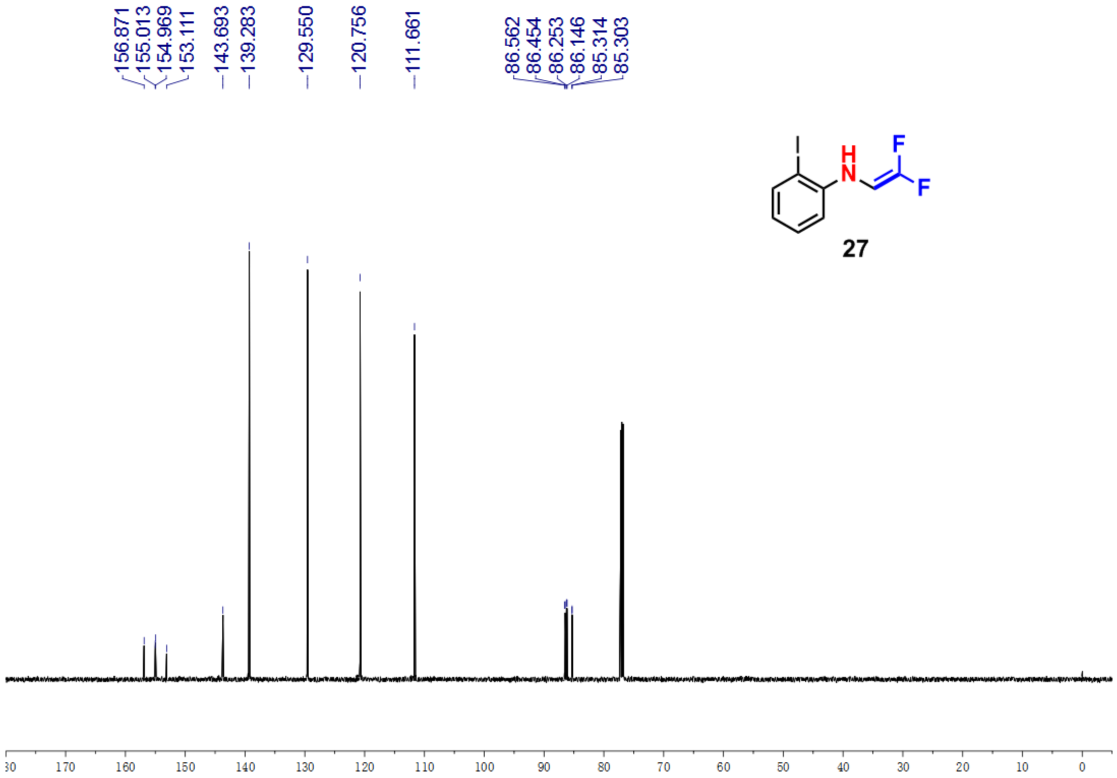

Supplementary Figure 77. ^{13}C NMR of 27

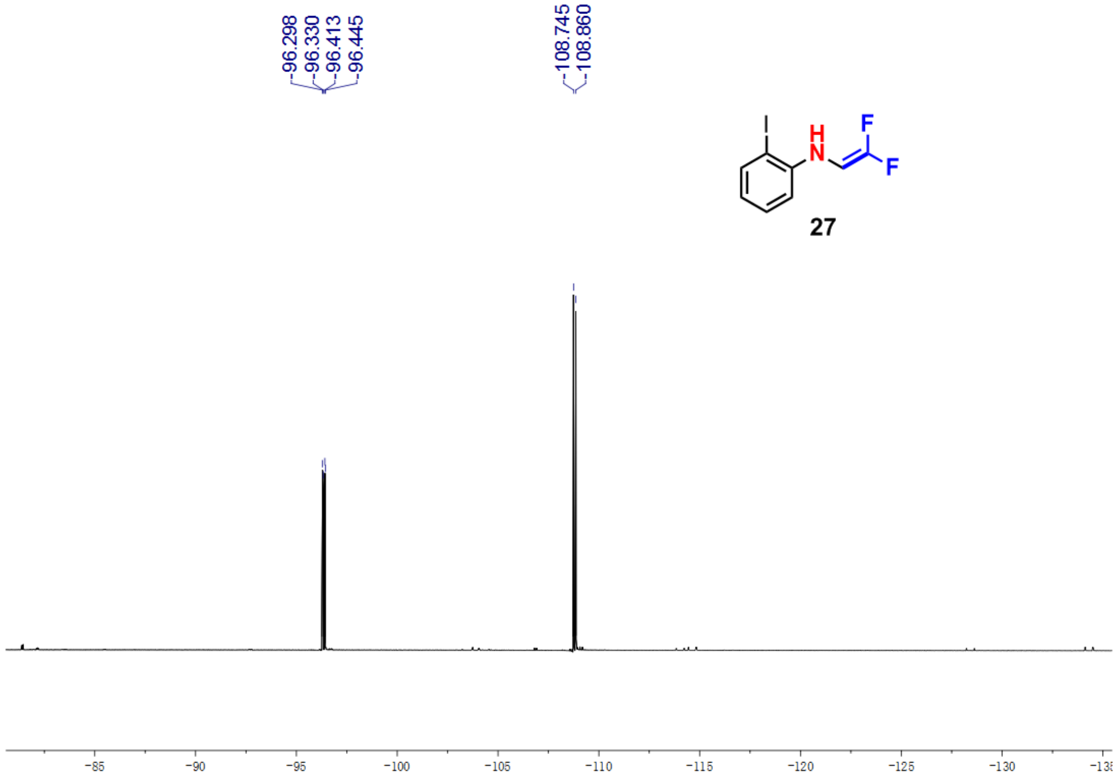

Supplementary Figure 78. ^{19}H NMR of 27

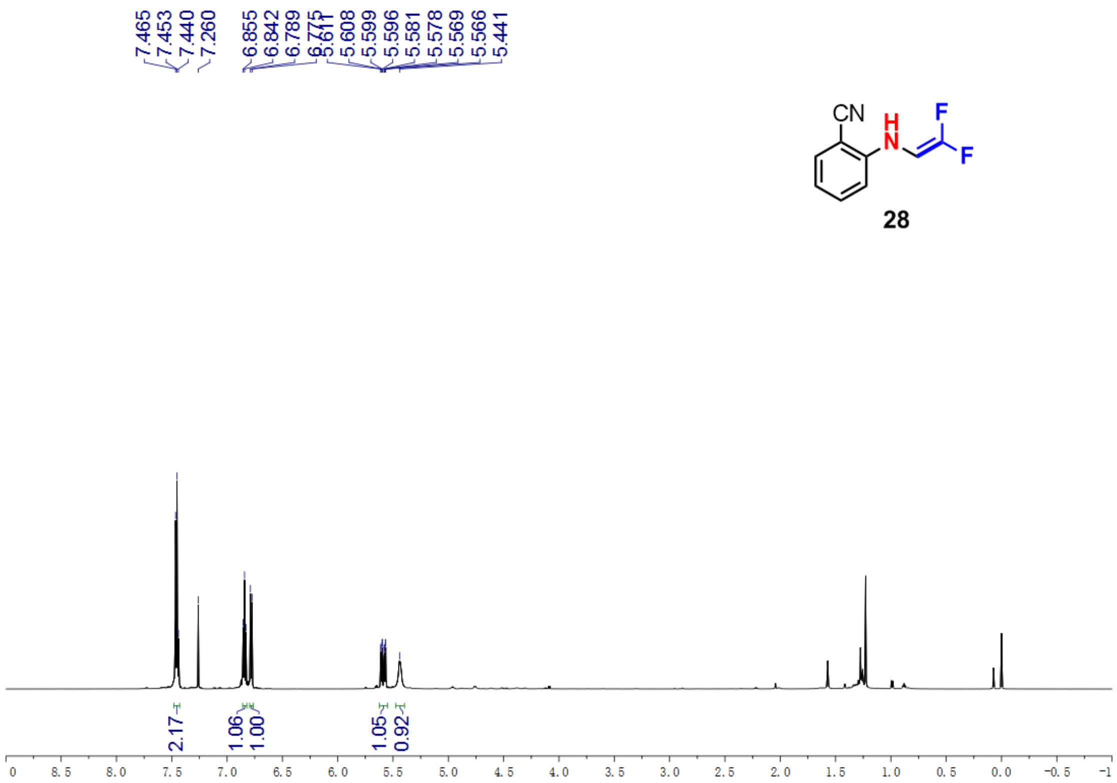

Supplementary Figure 79. ^{1}H NMR of 28

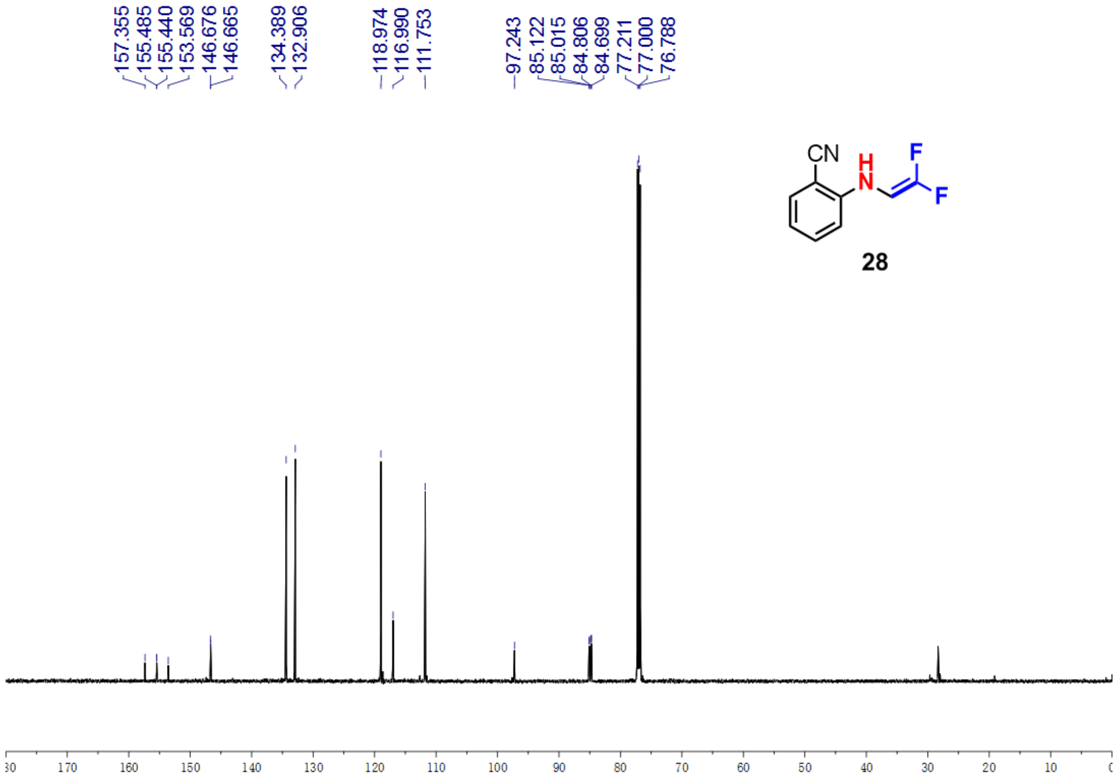

Supplementary Figure 80. ^{13}C NMR of 28

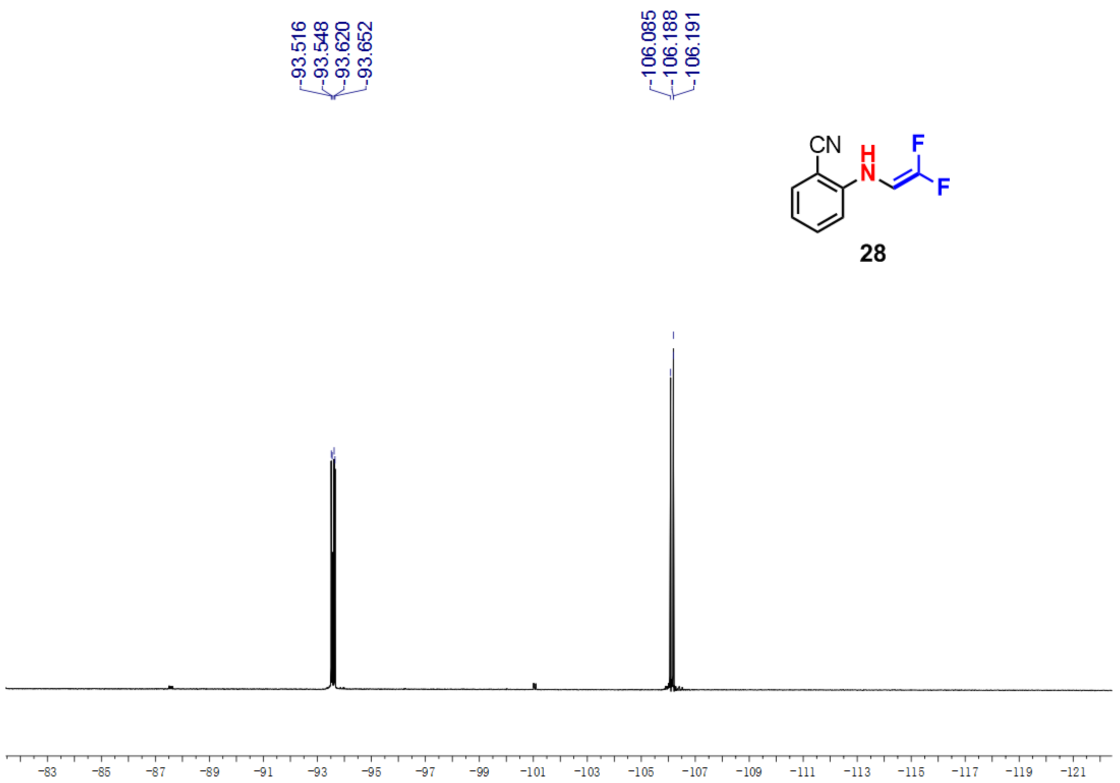

Supplementary Figure 81. ^{19}F NMR of 28

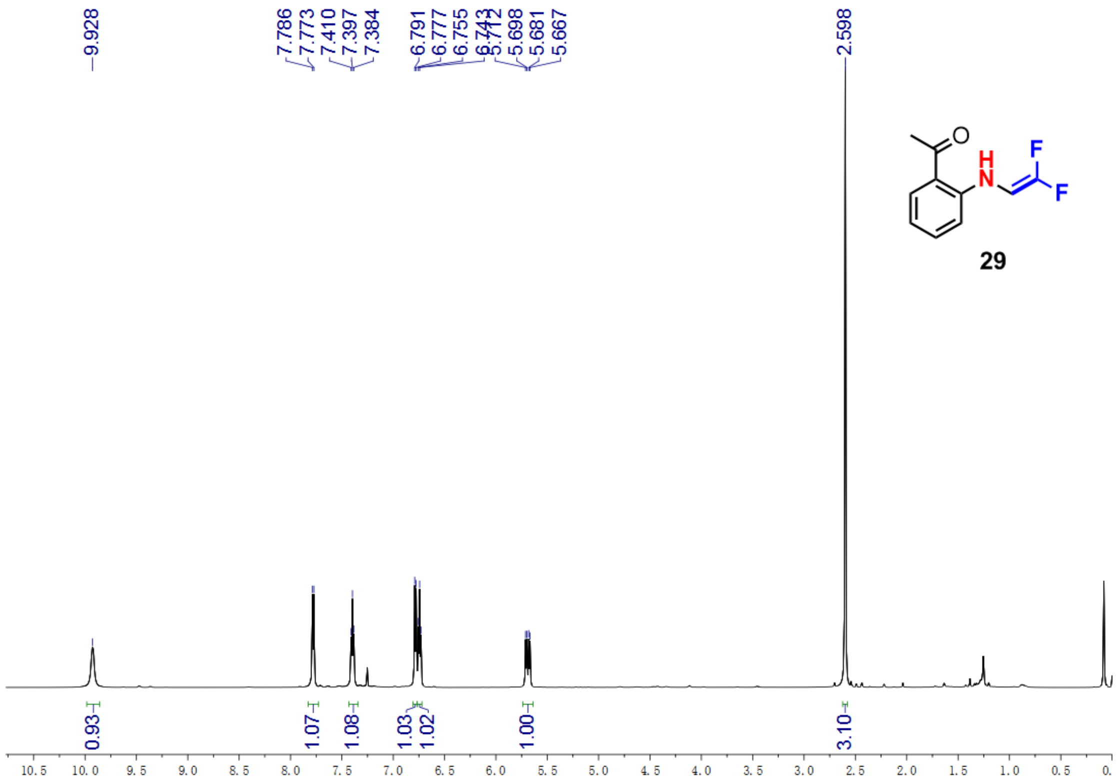

Supplementary Figure 82. ^{1}H NMR of 29

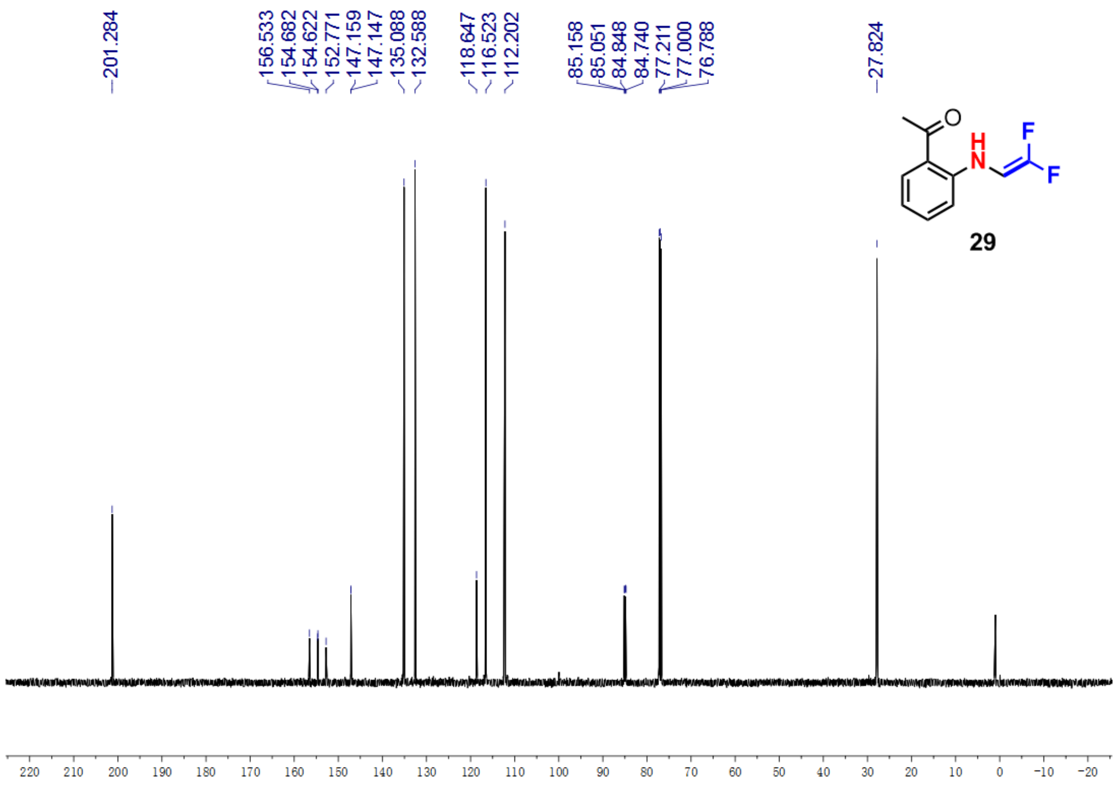

Supplementary Figure 83. ^{13}C NMR of 29

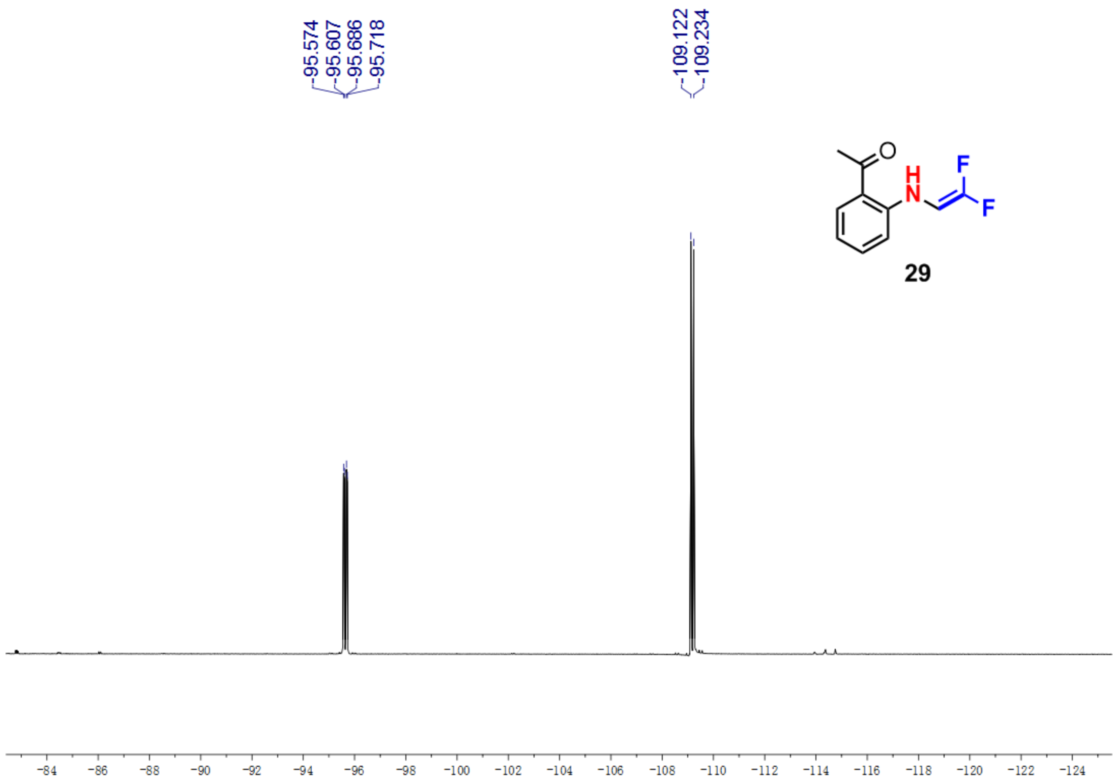

Supplementary Figure 84. ^{19}F NMR of 29

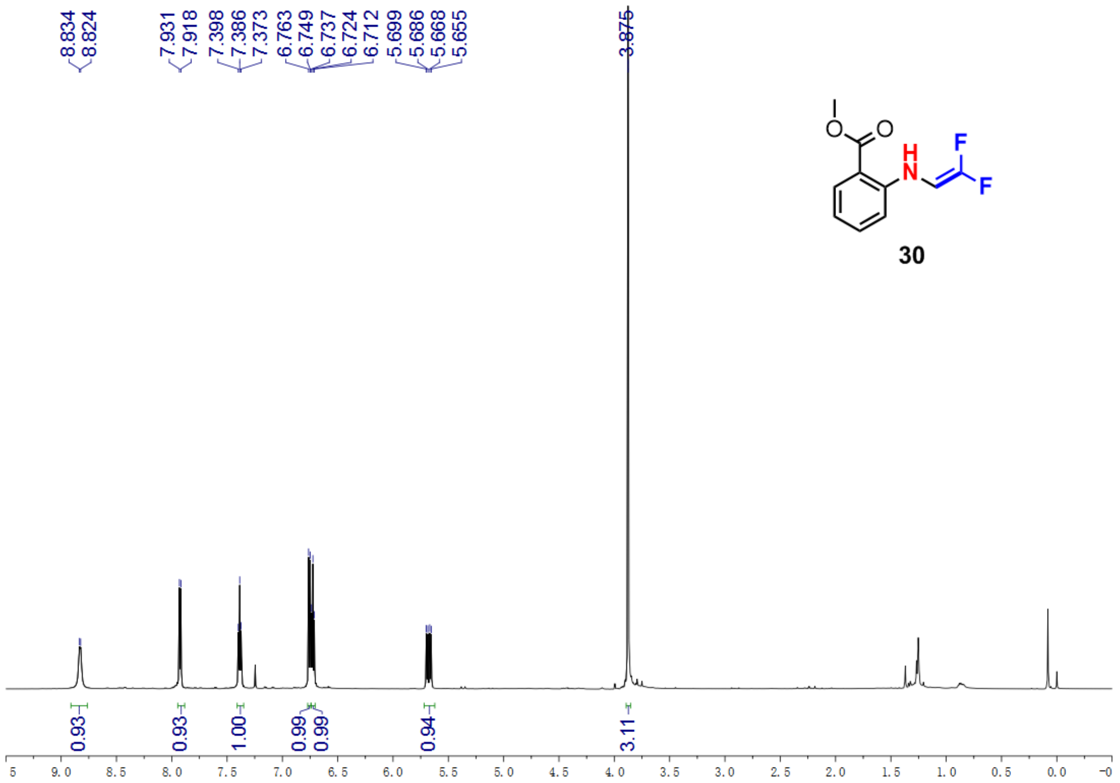

Supplementary Figure 85. ^{1}H NMR of 30

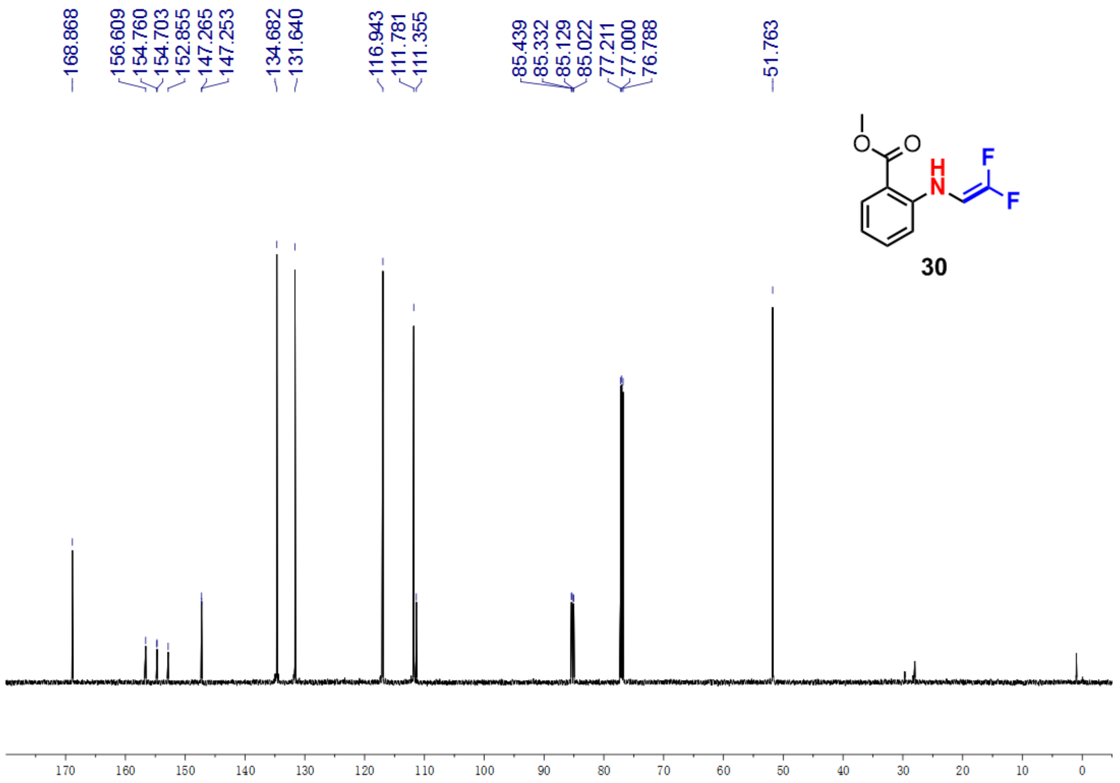

Supplementary Figure 86. ^{13}C NMR of 30

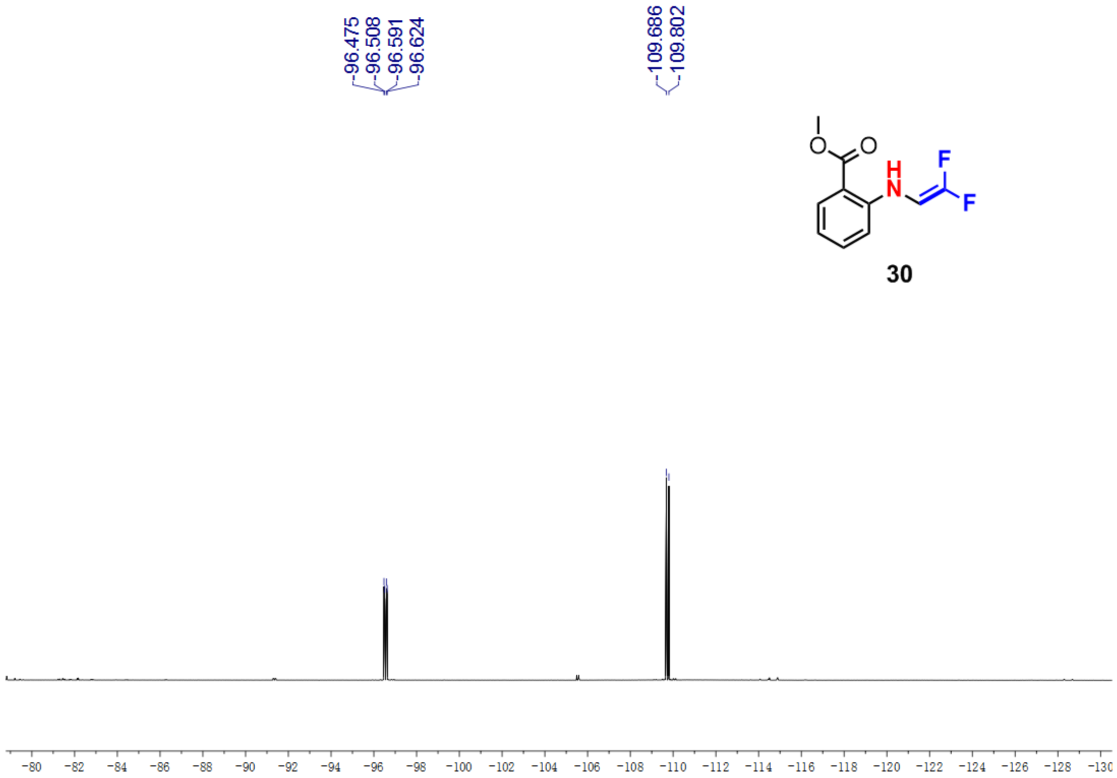

Supplementary Figure 87. ^{19}F NMR of 30

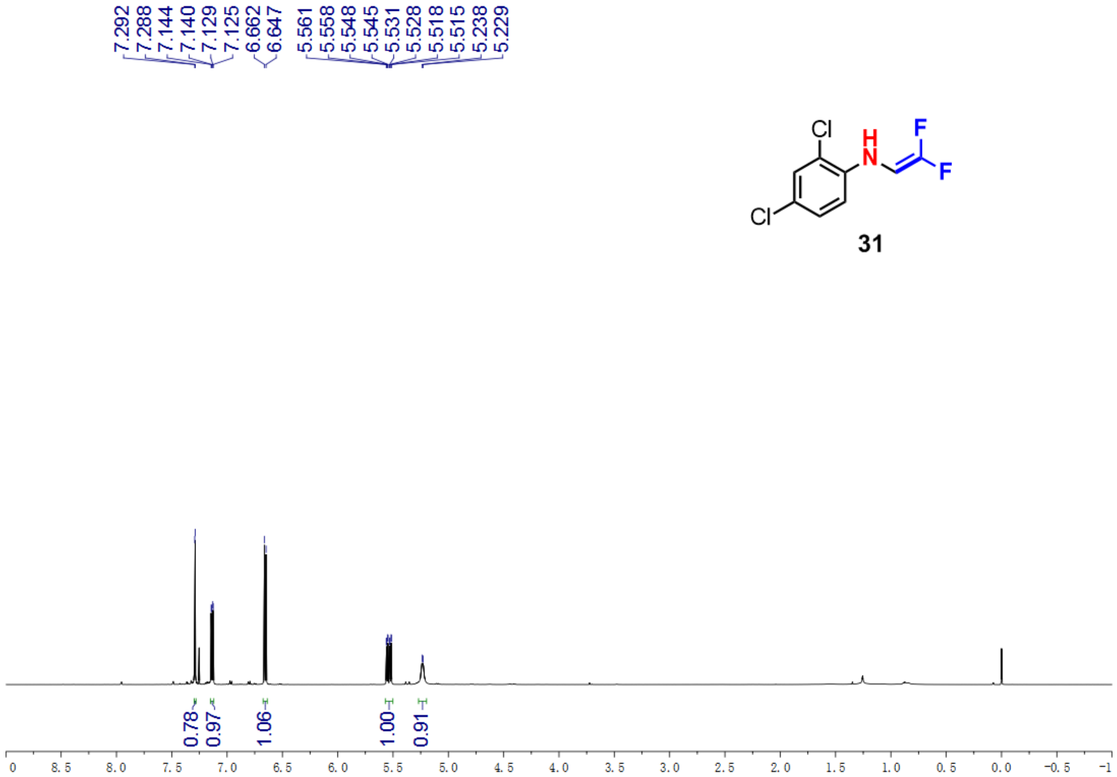

Supplementary Figure 88. ^{1}H NMR of 31

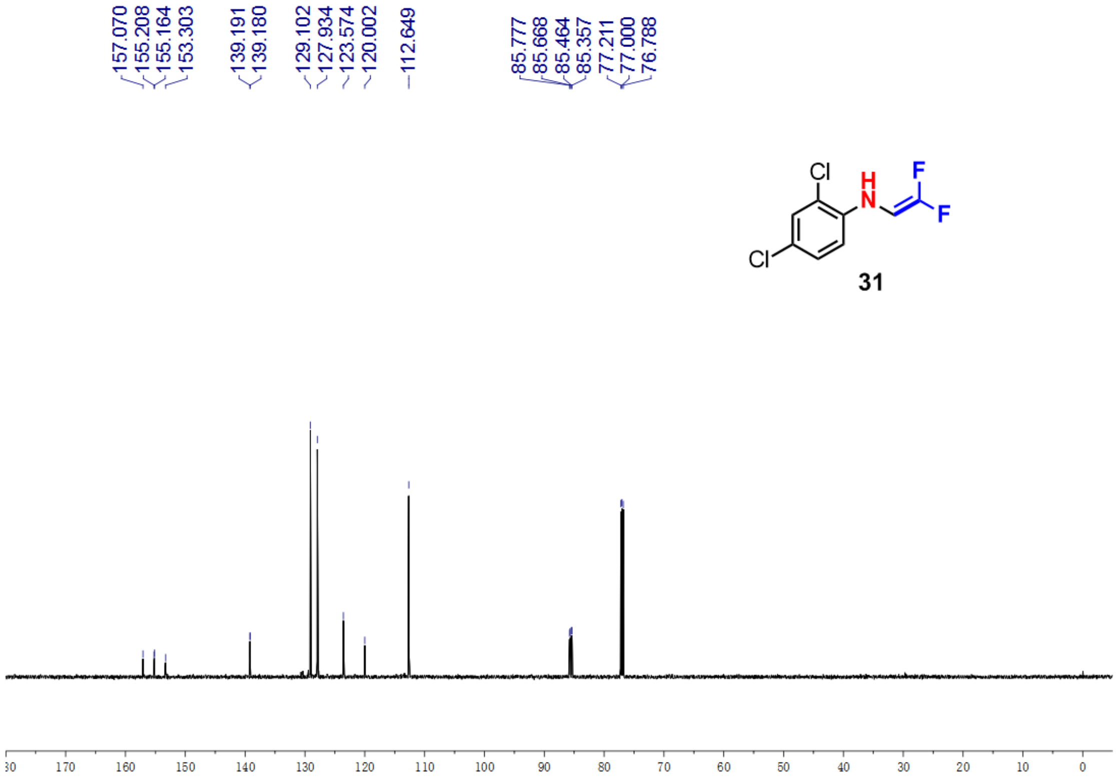

Supplementary Figure 89. ^{13}H NMR of 31

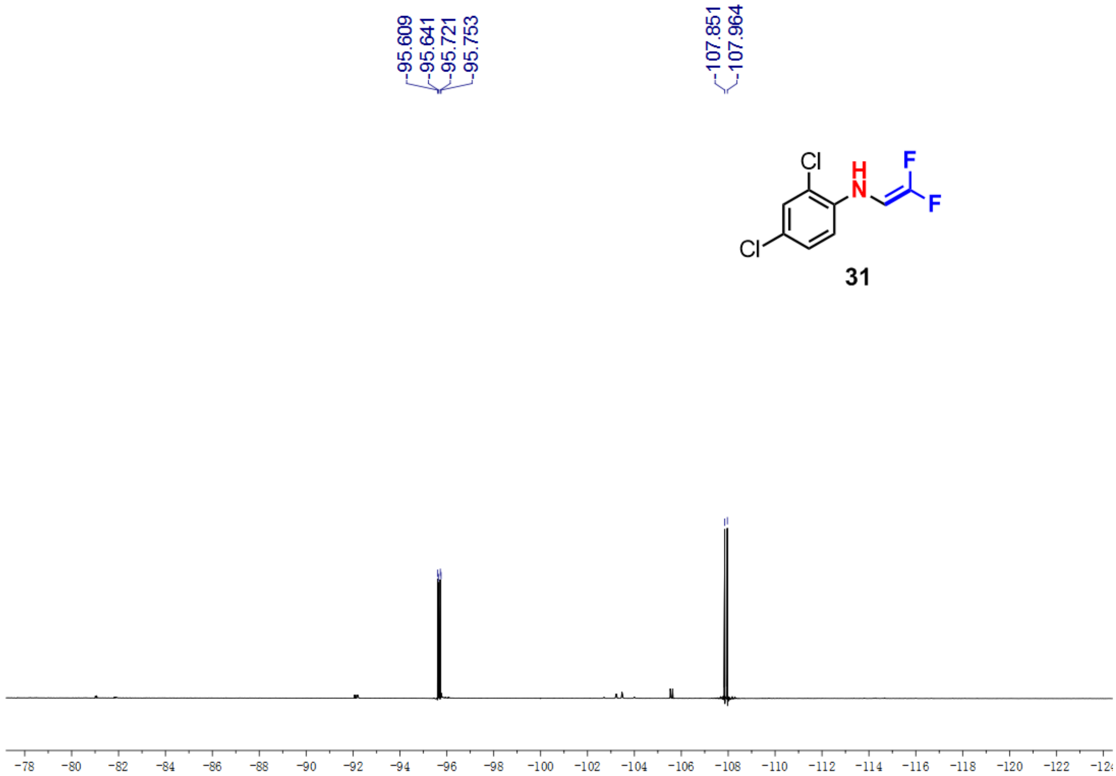

Supplementary Figure 90. ^{19}H NMR of 31

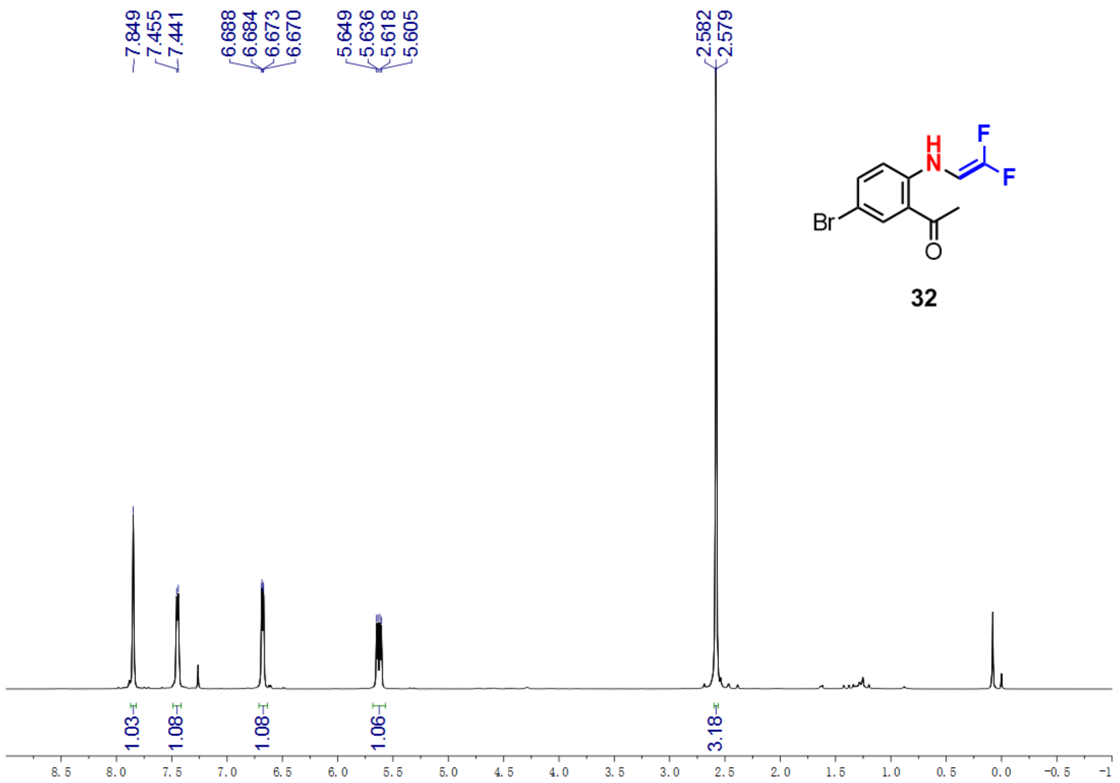

Supplementary Figure 91. ^{1}H NMR of 32

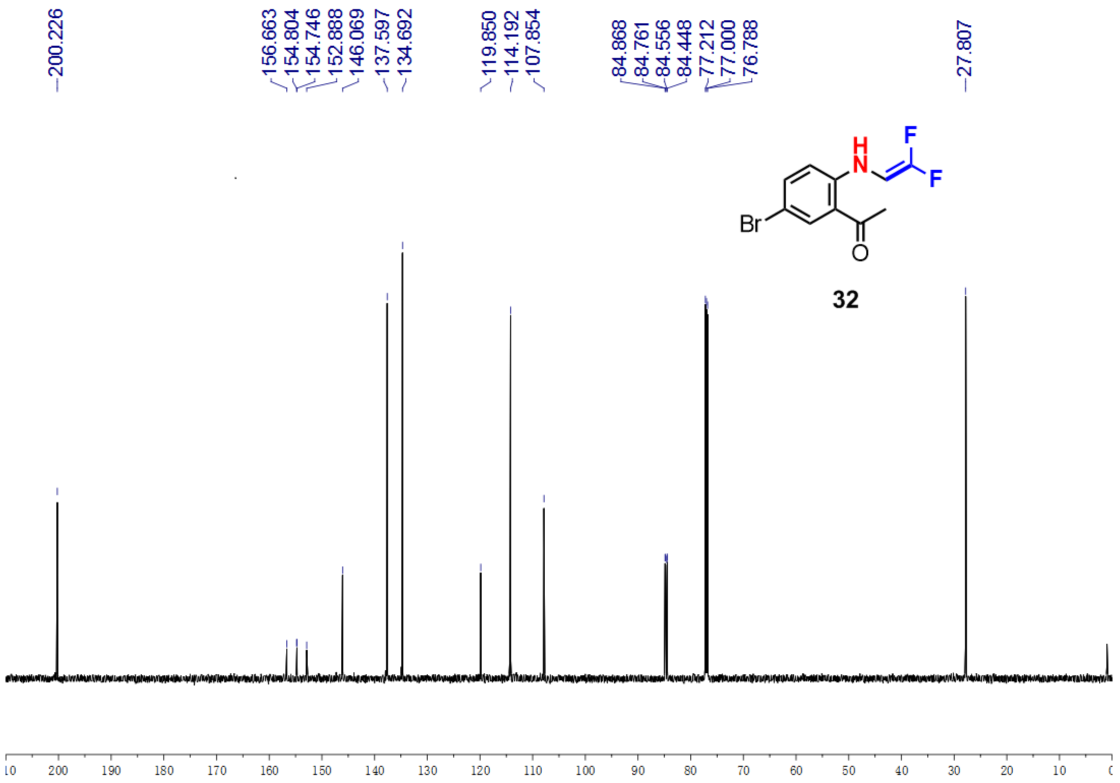

Supplementary Figure 92. ^{13}C NMR of 32

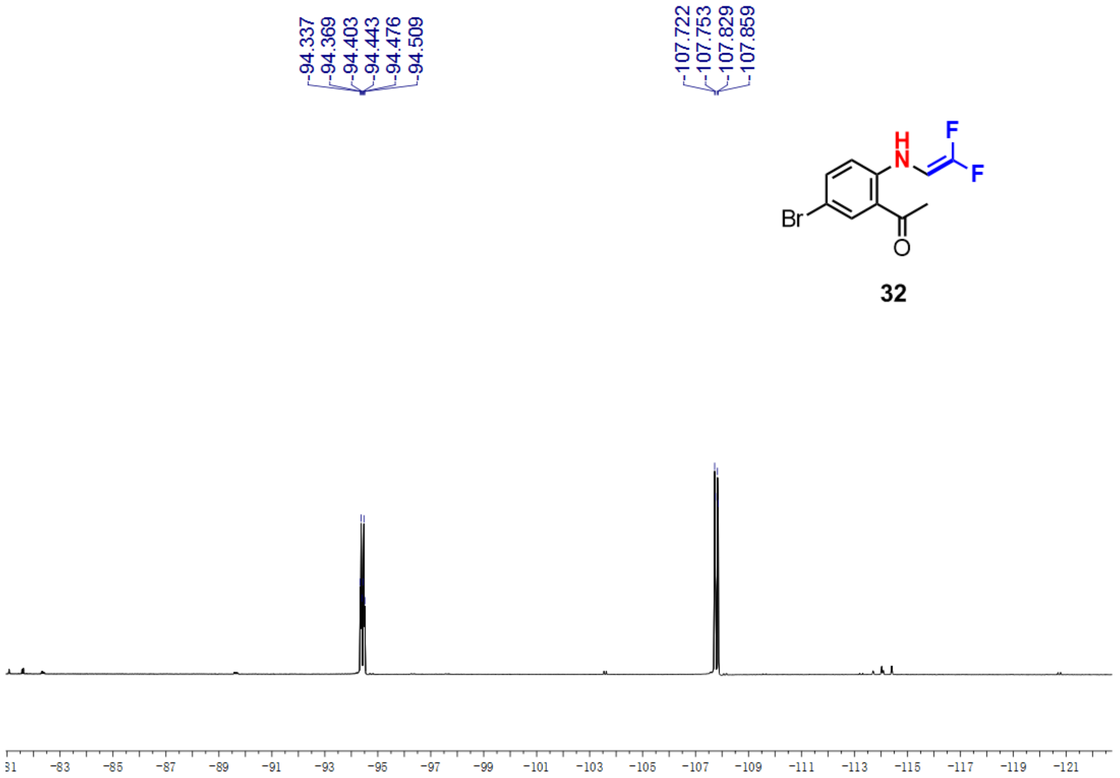

Supplementary Figure 93. ^{19}F NMR of 32

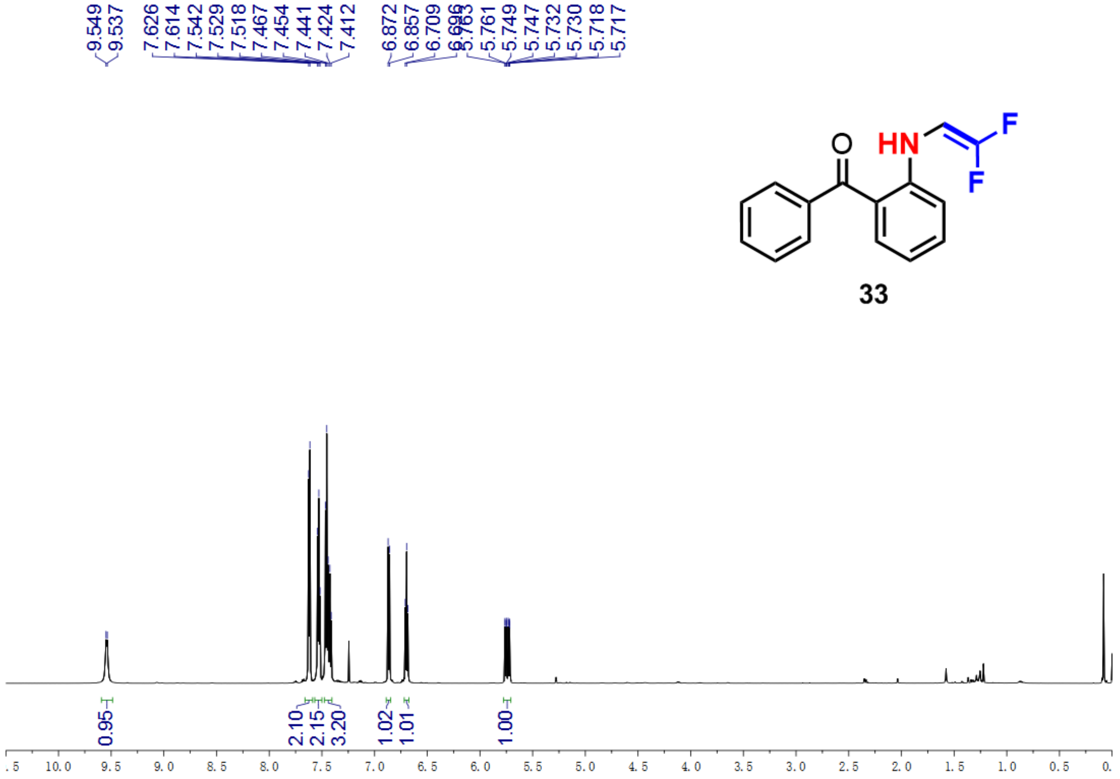

Supplementary Figure 94. ^{1}H NMR of 33

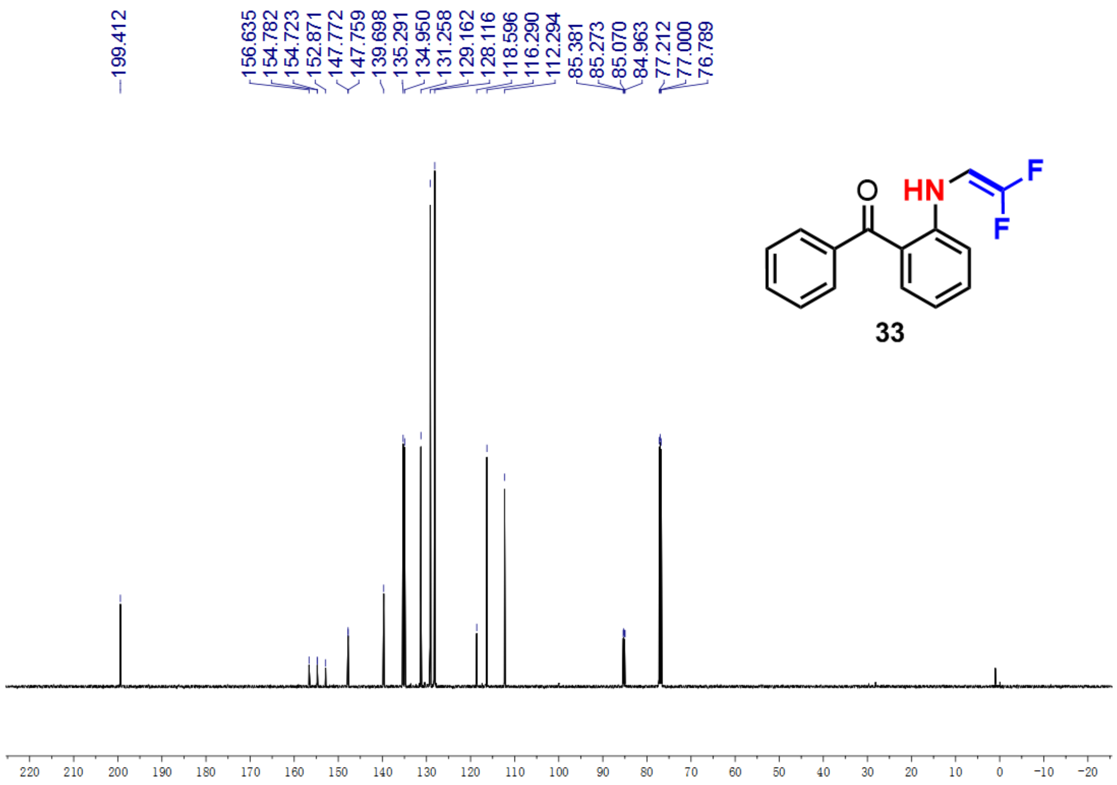

Supplementary Figure 95. ^{13}C NMR of 33

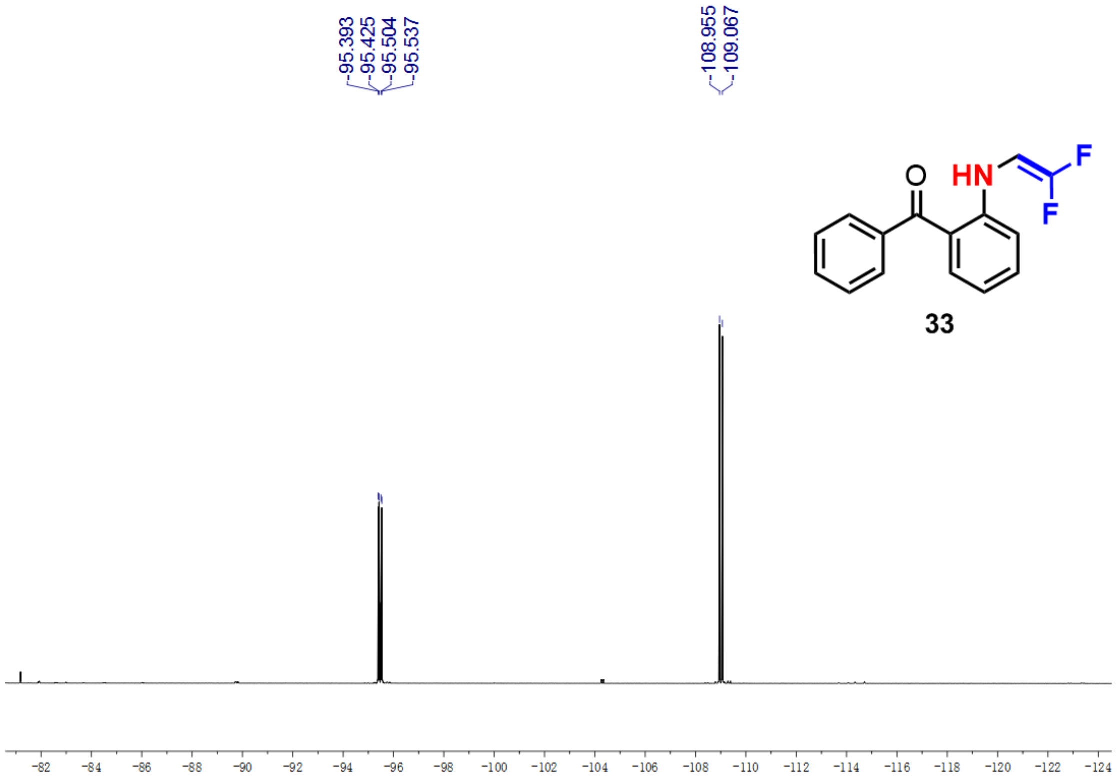

Supplementary Figure 96. ^{19}F NMR of 33

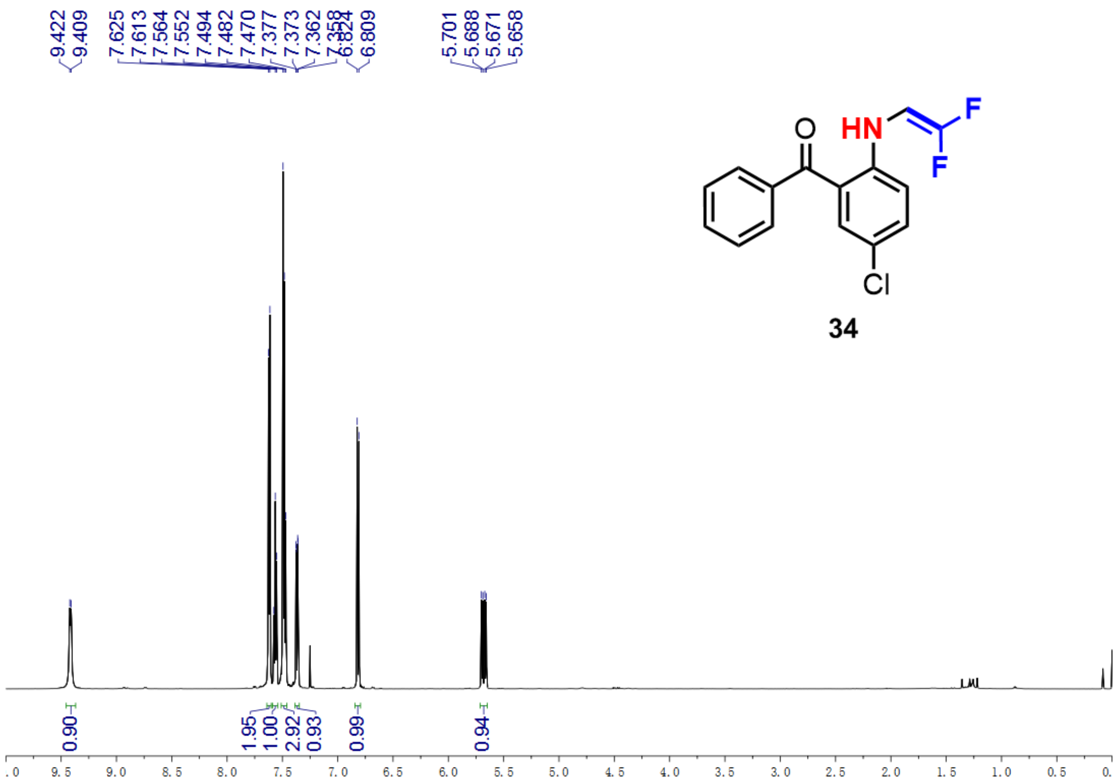

Supplementary Figure 97. ^{1}H NMR of 34

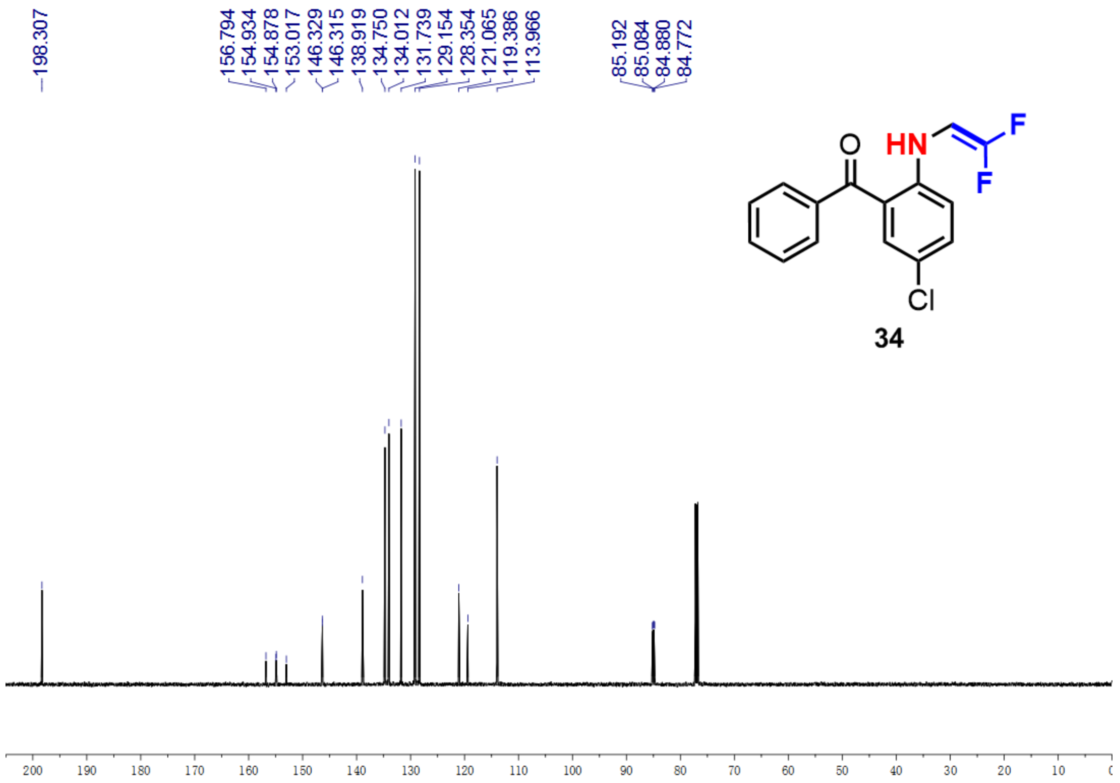

Supplementary Figure 98. ^{13}C NMR of 34

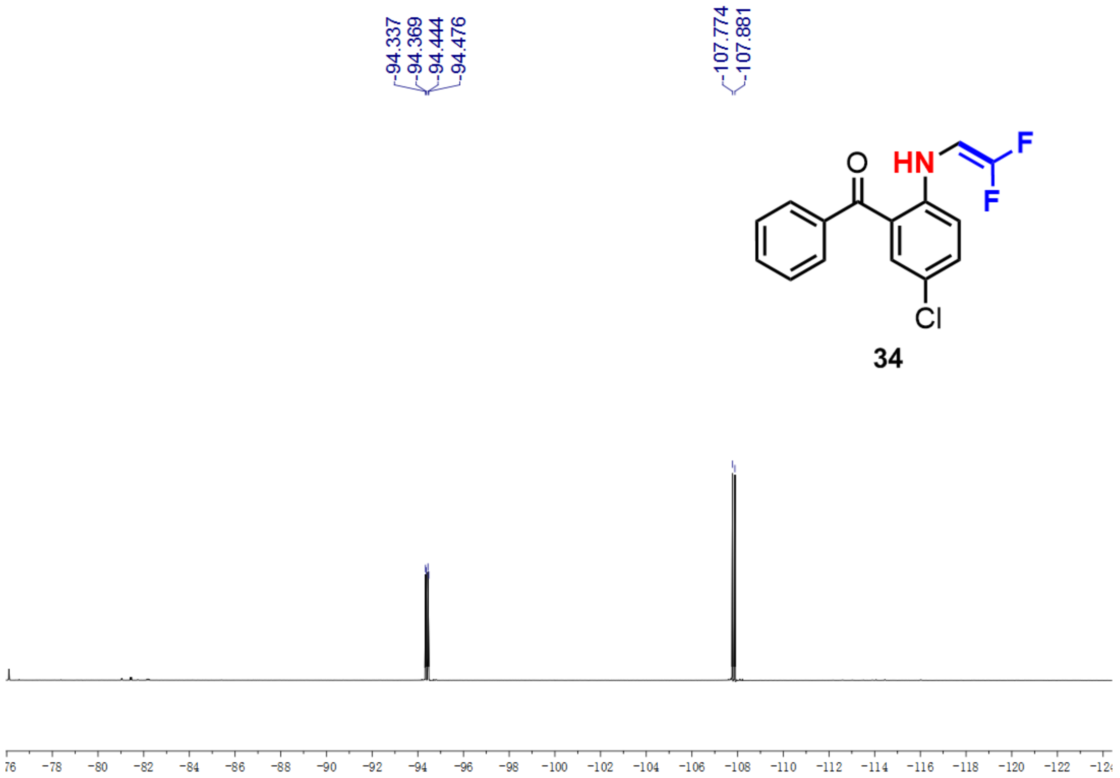

Supplementary Figure 99. ^{19}F NMR of 34

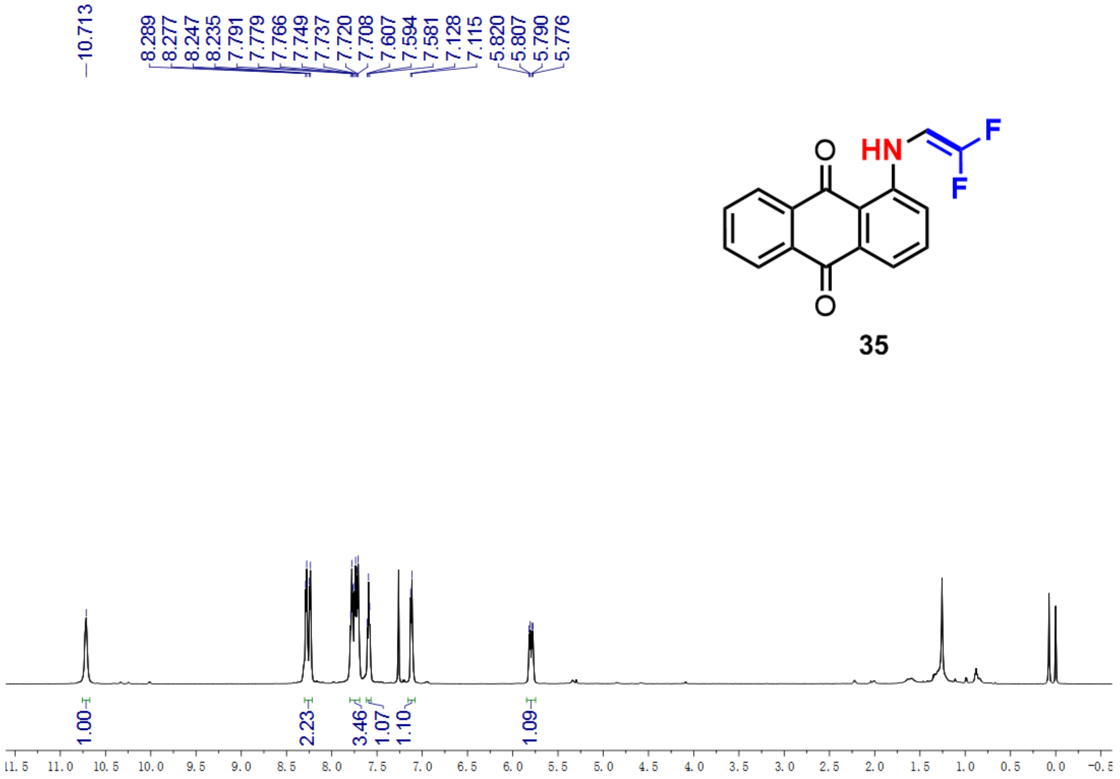

Supplementary Figure 100. ^{1}H NMR of 35

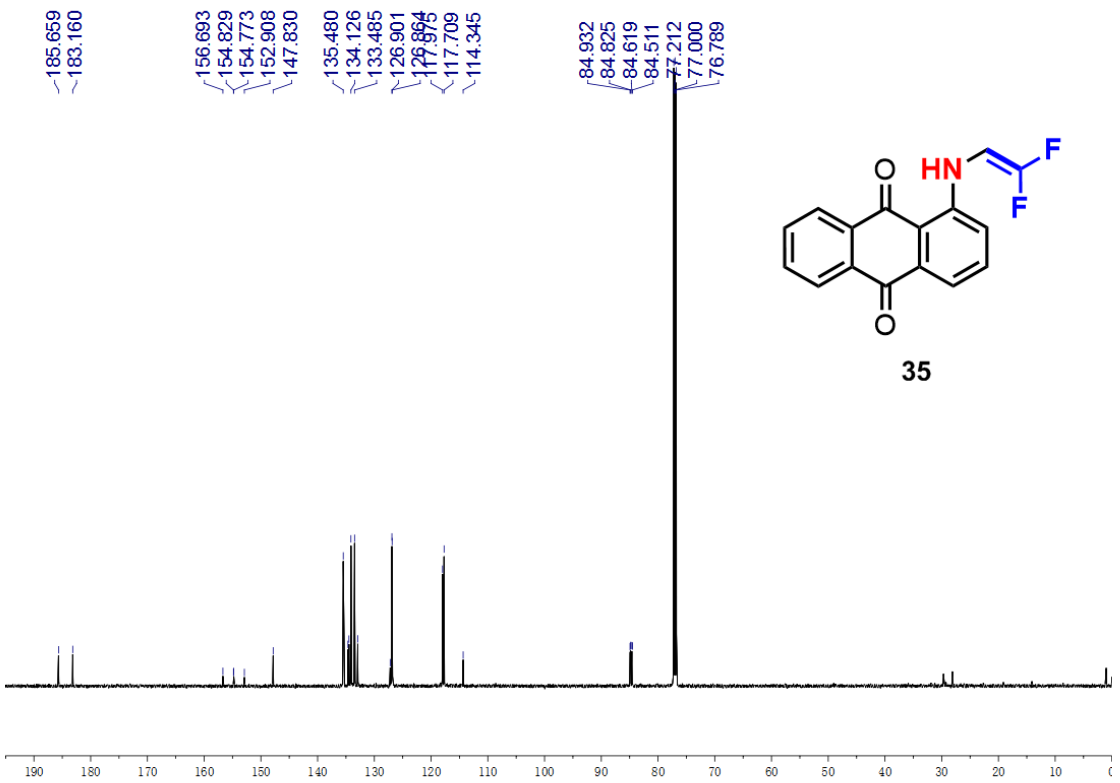

Supplementary Figure 101. ^{13}C NMR of 35

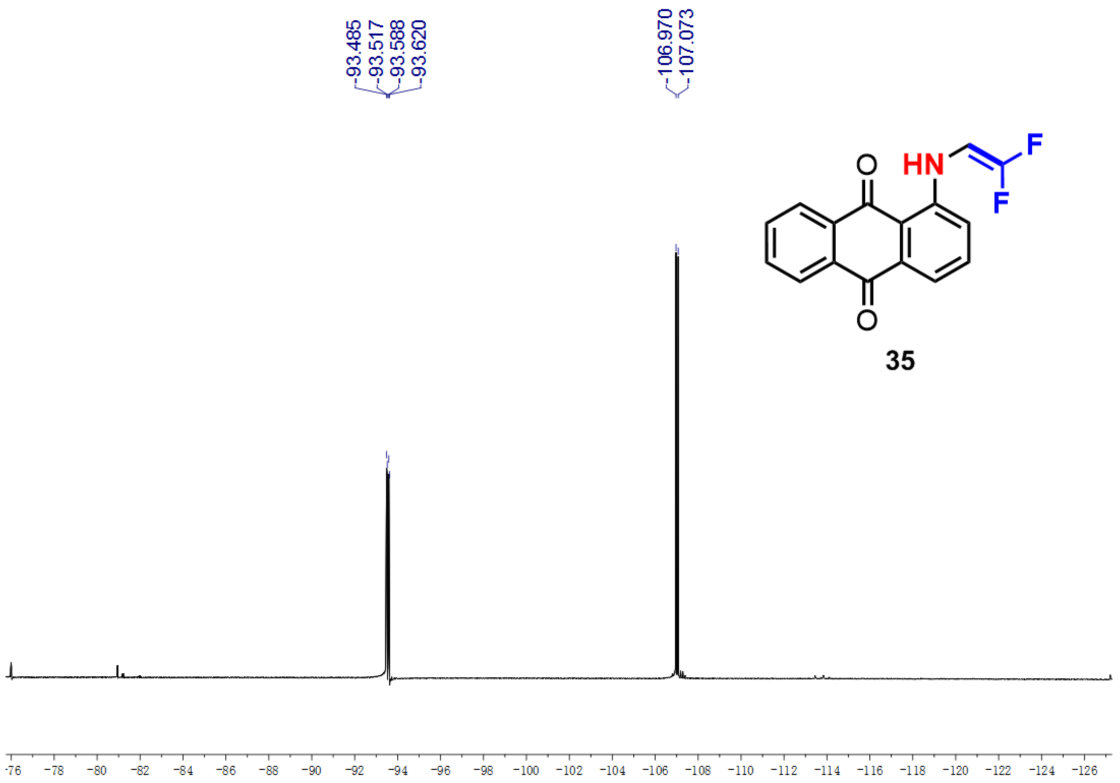

Supplementary Figure 102. ^{19}F NMR of 35

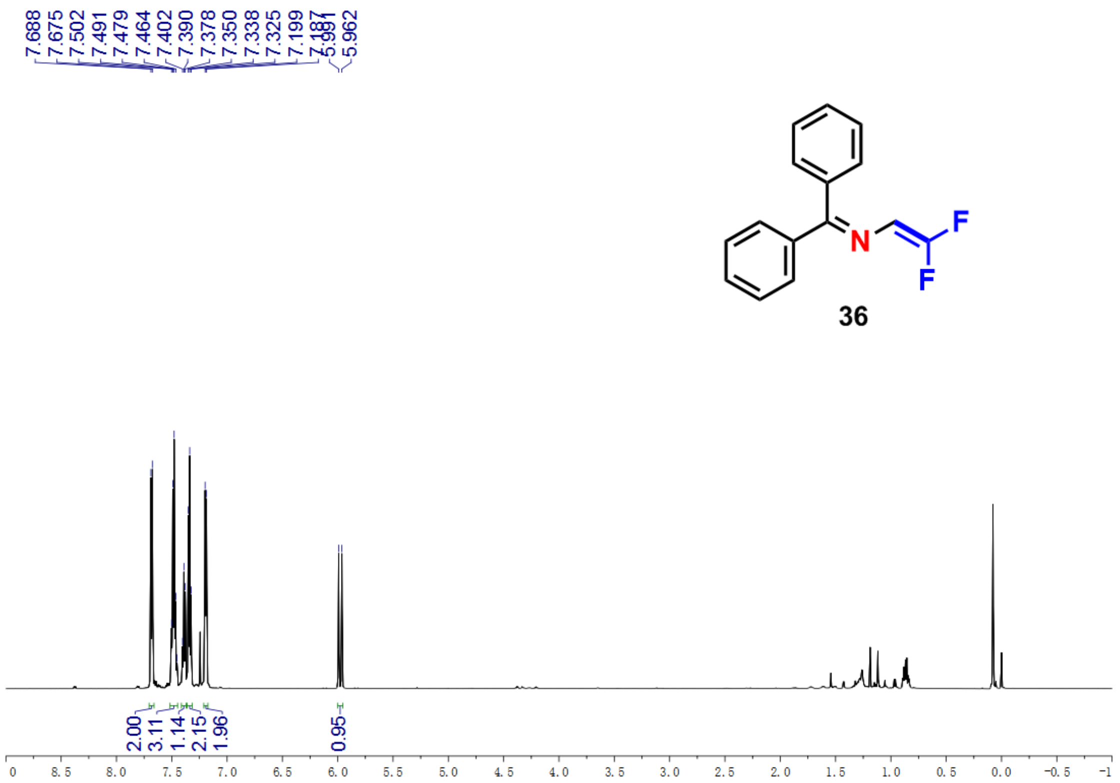

Supplementary Figure 103. ^{1}H NMR of 36

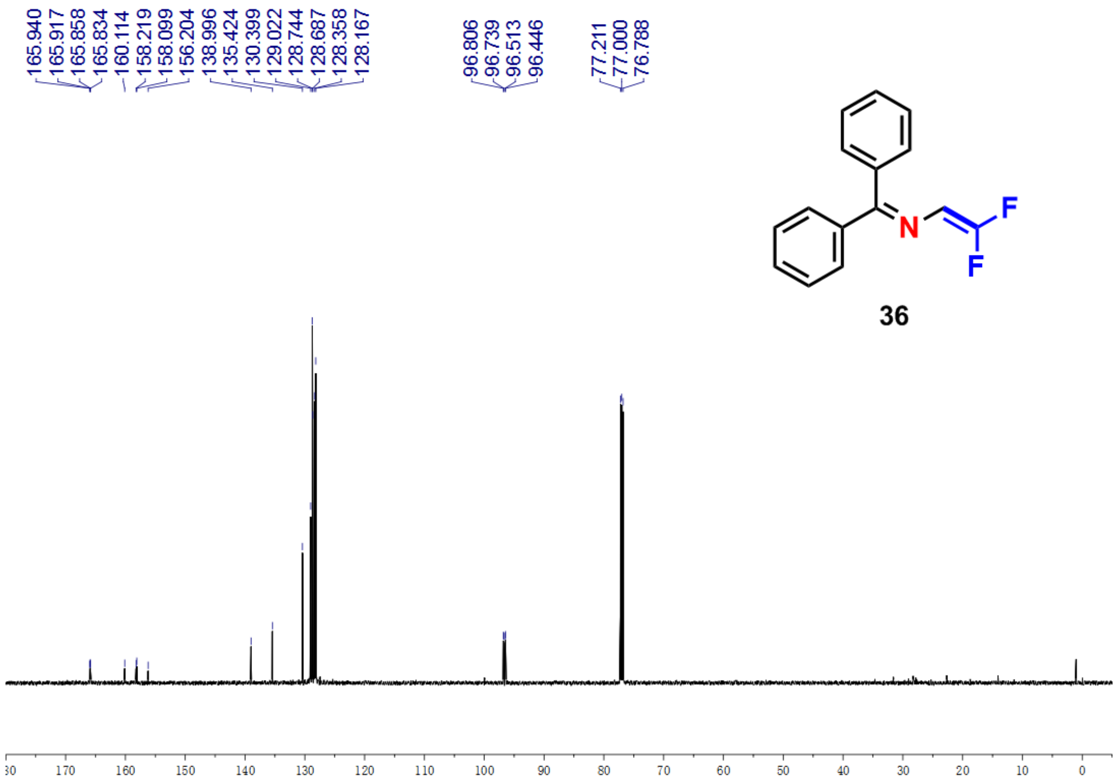

Supplementary Figure 104. ^{13}C NMR of 36

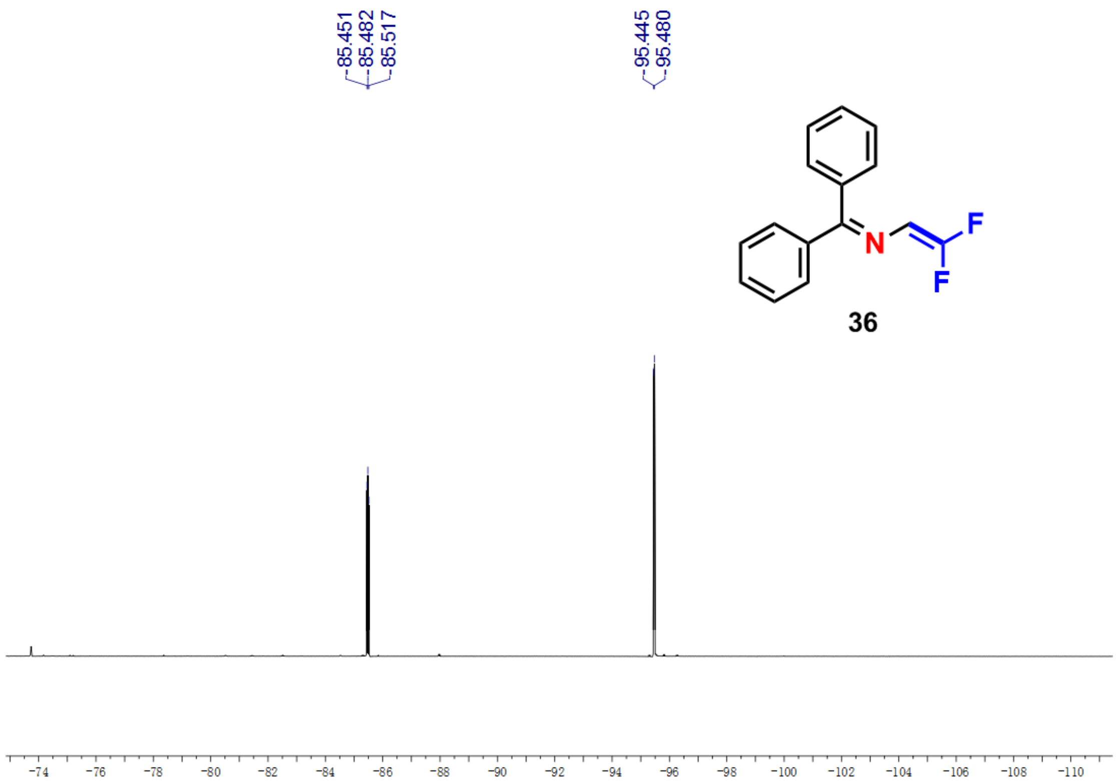

Supplementary Figure 105. ^{19}F NMR of 36

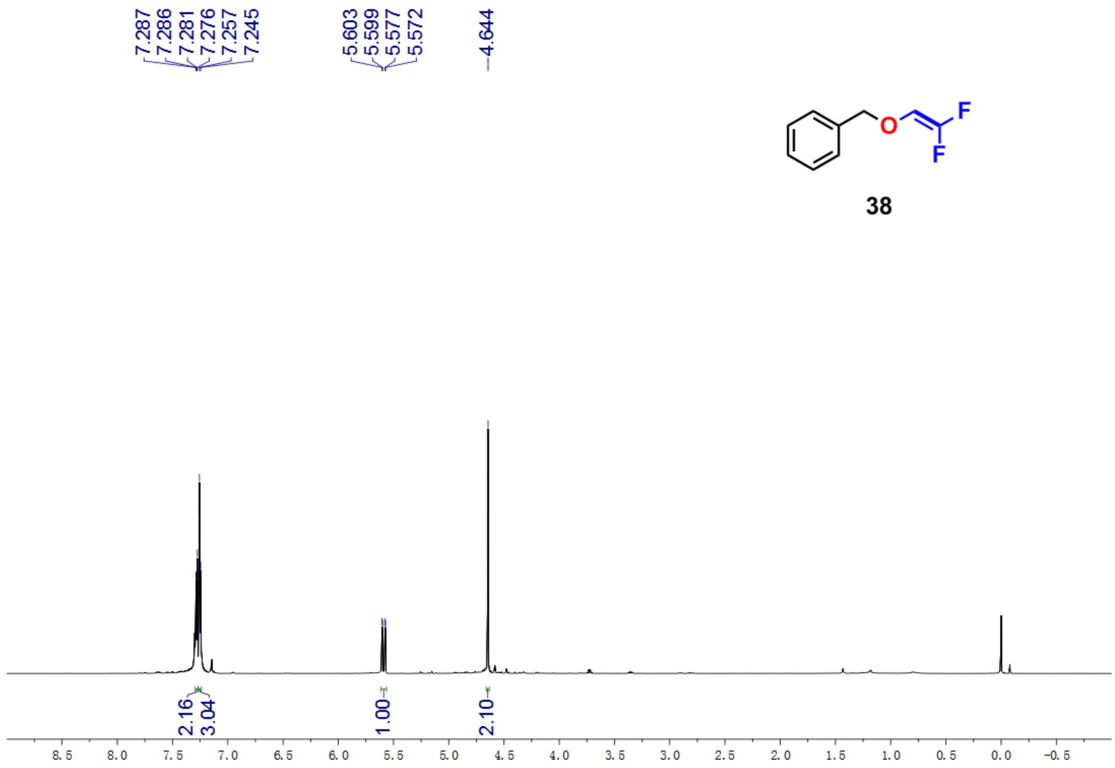

Supplementary Figure 106. ^{1}H NMR of 38

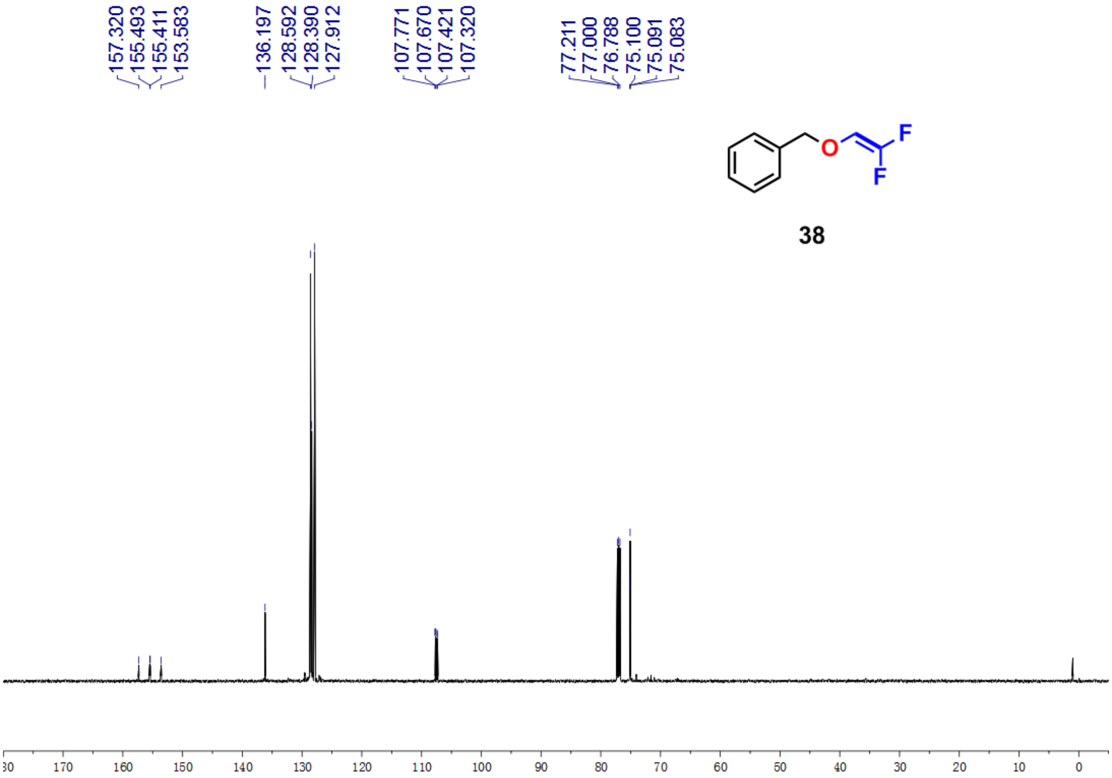

Supplementary Figure 107. ^{13}C NMR of 38

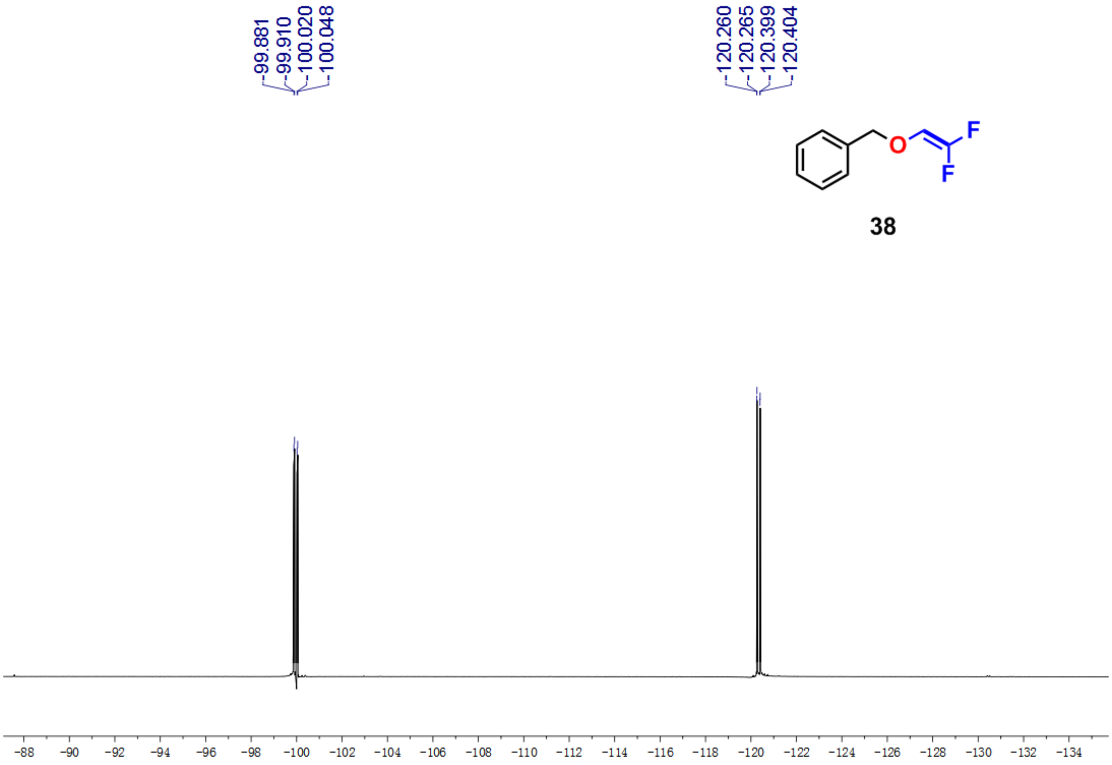

Supplementary Figure 108. ^{19}F NMR of 38

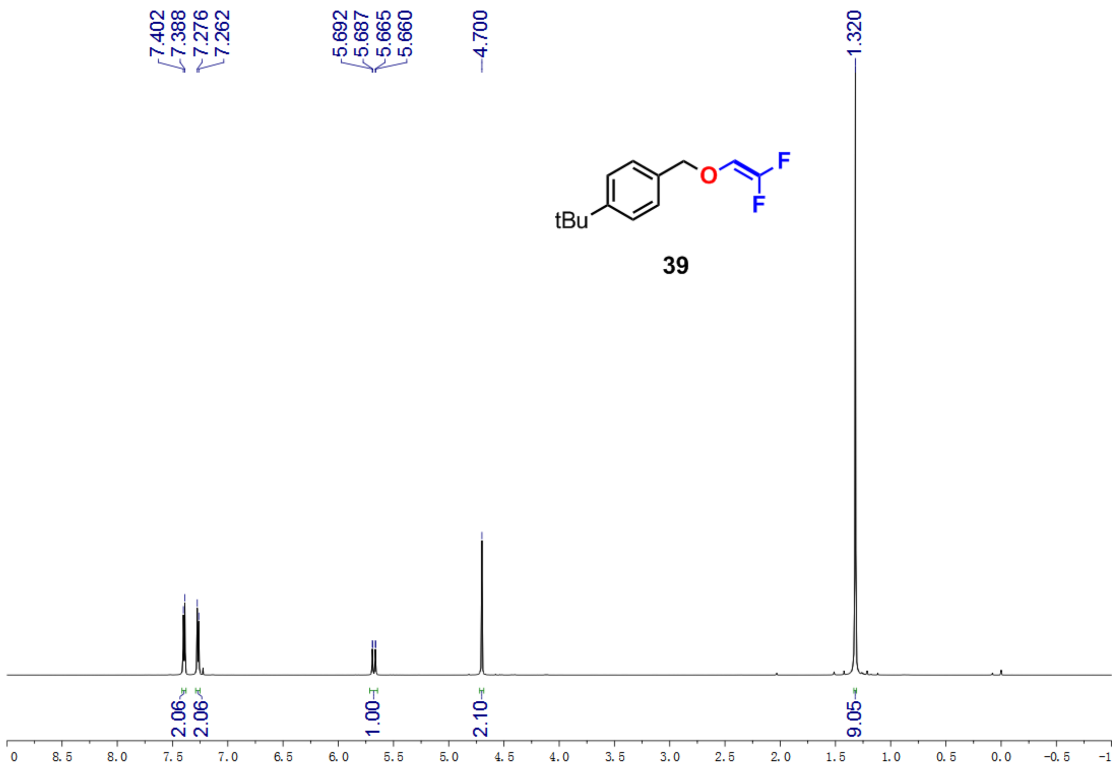

Supplementary Figure 109. ^{1}H NMR of 39

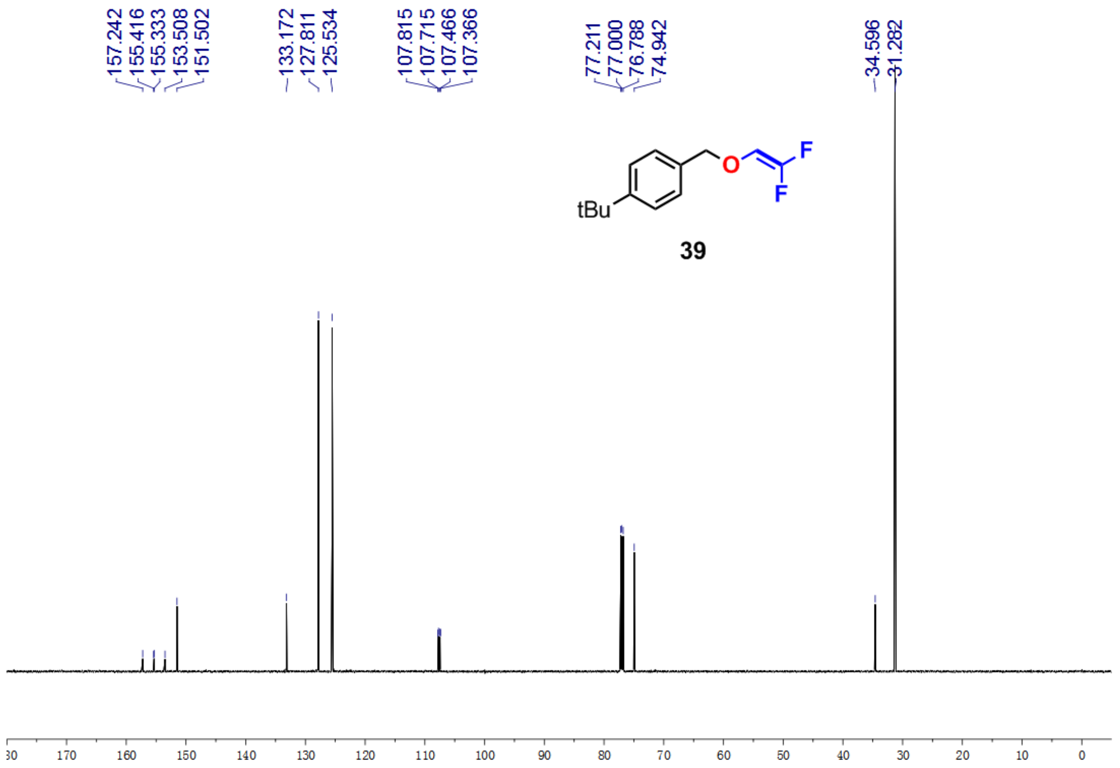

Supplementary Figure 110. ^{13}C NMR of 39

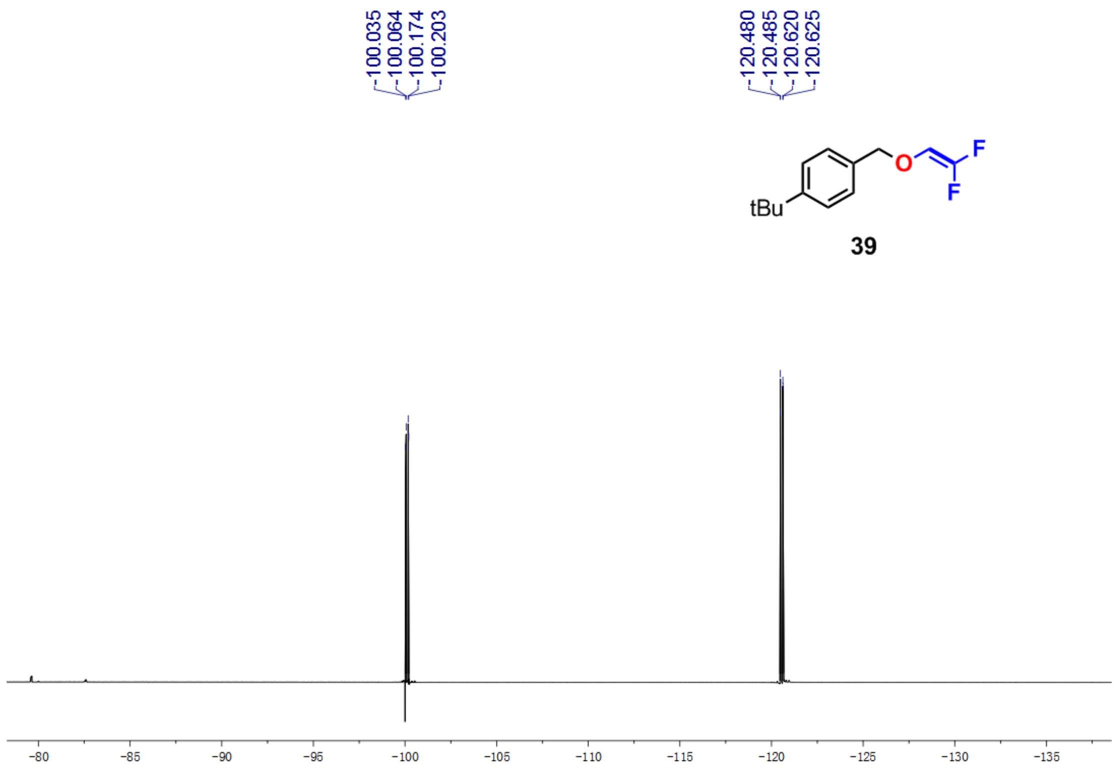

Supplementary Figure 111. ^{19}F NMR of 39

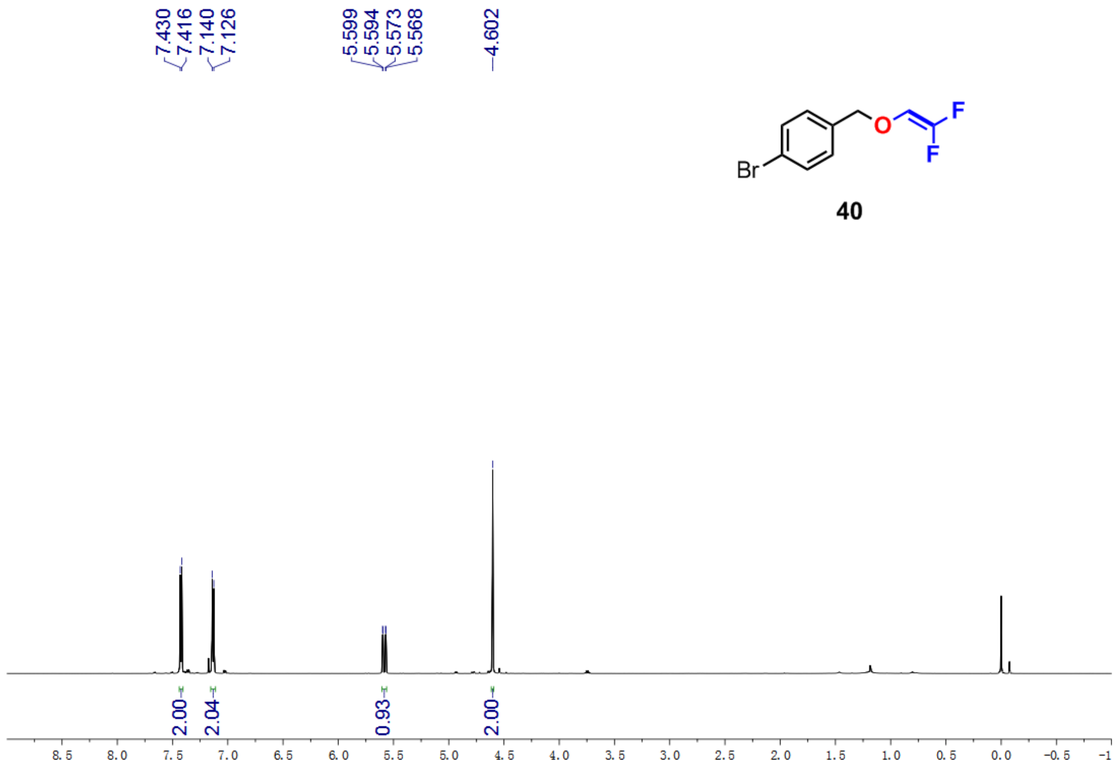

Supplementary Figure 112. ^{1}H NMR of 40

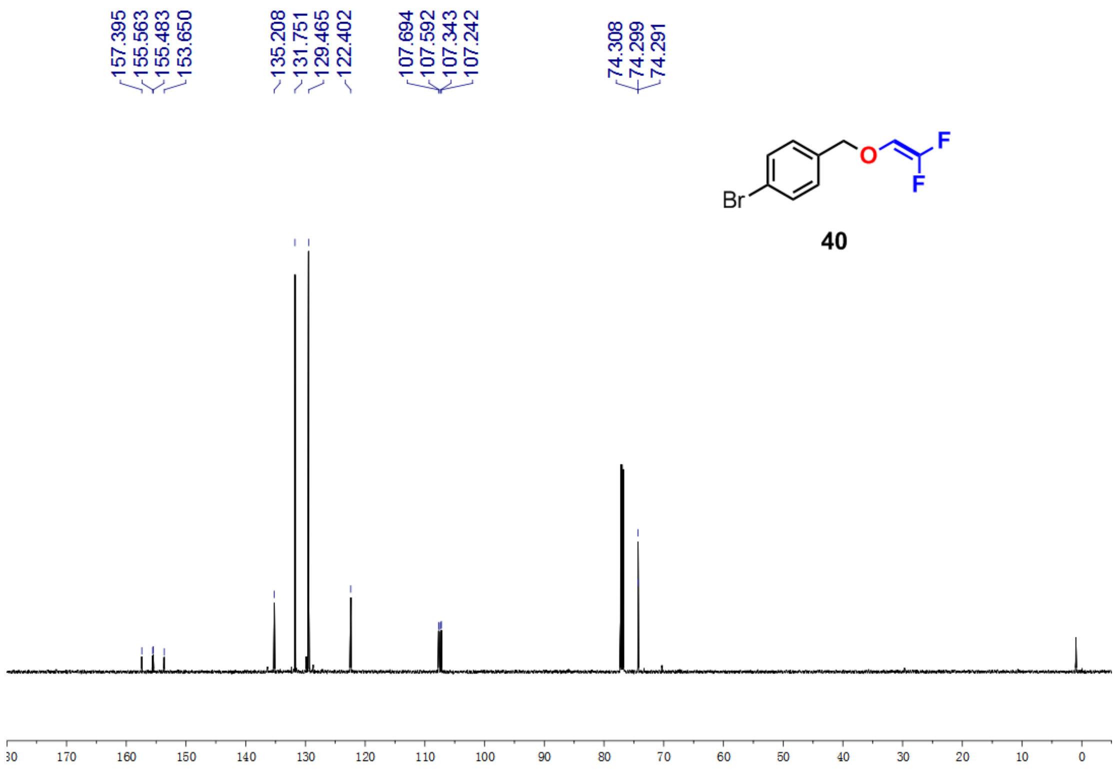

Supplementary Figure 113. ^{13}C NMR of 40

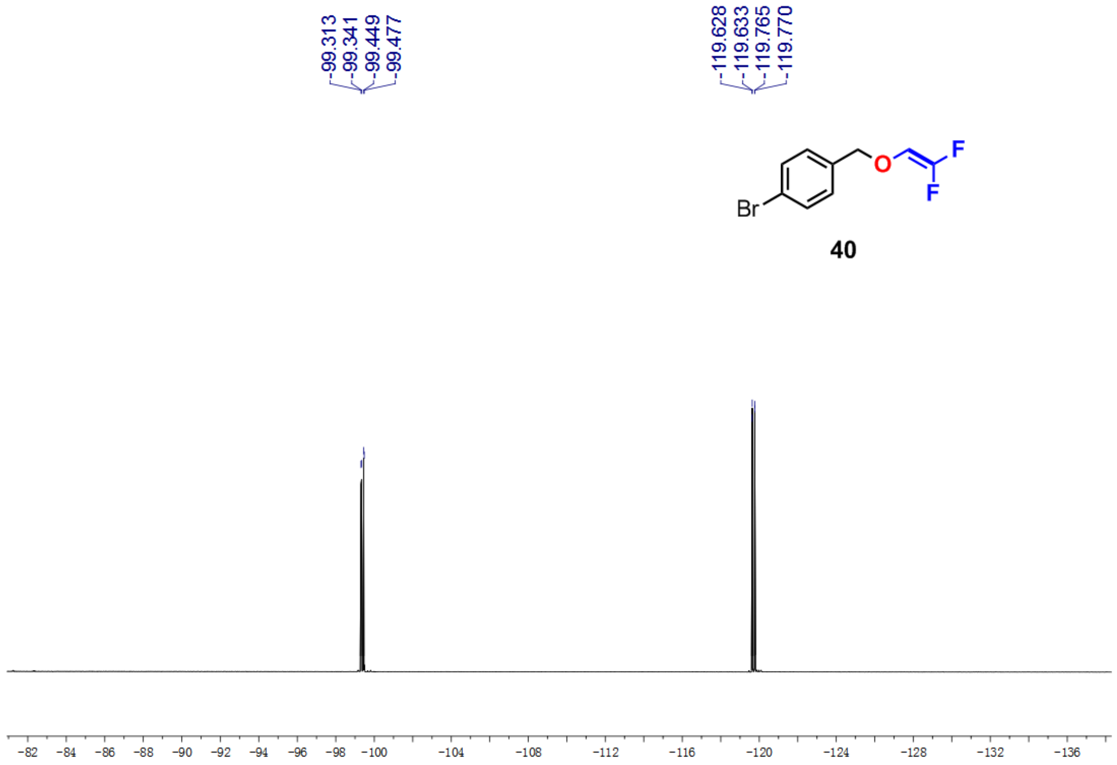

Supplementary Figure 114. ^{19}F NMR of 40

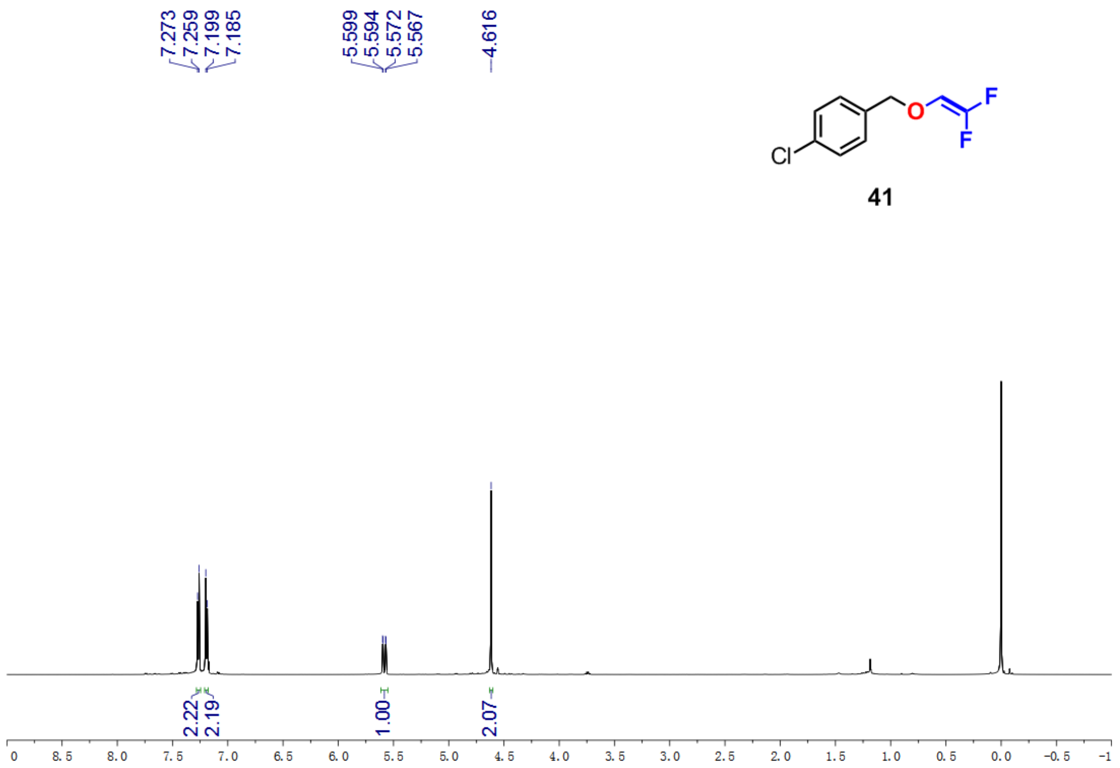

Supplementary Figure 115. ^{1}H NMR of 41

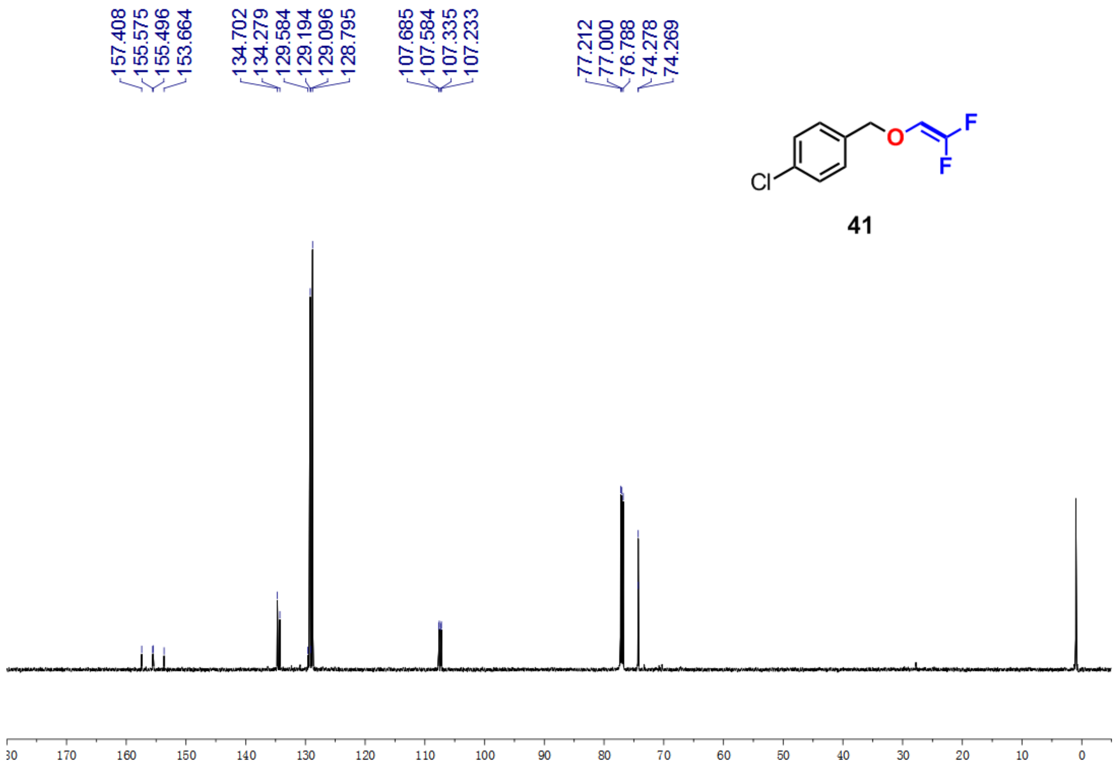

Supplementary Figure 116. ^{13}C NMR of 41

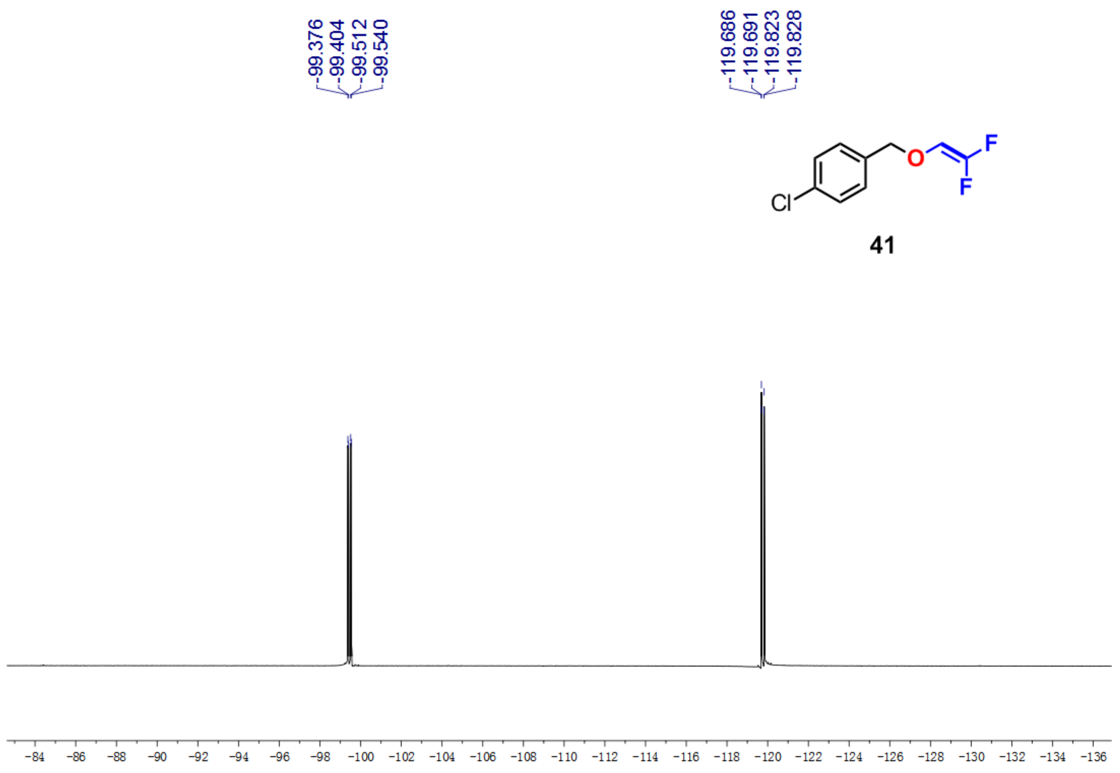

Supplementary Figure 117. ^{19}F NMR of 41

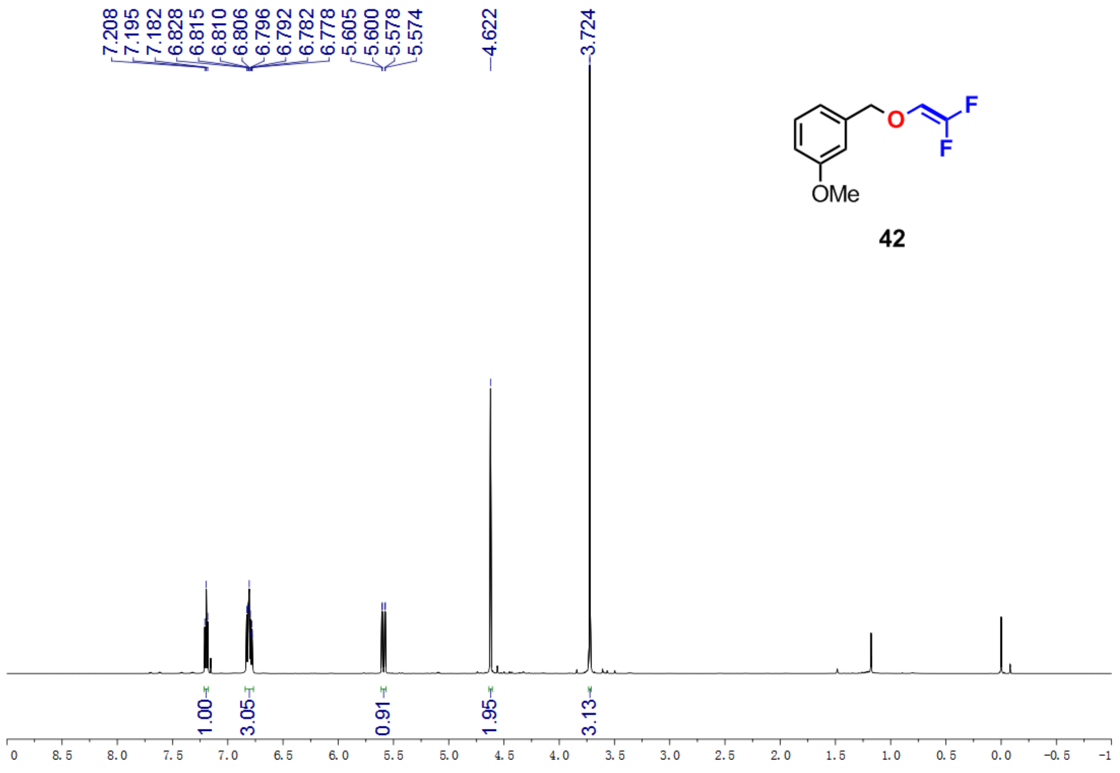

Supplementary Figure 118. ^{1}H NMR of 42

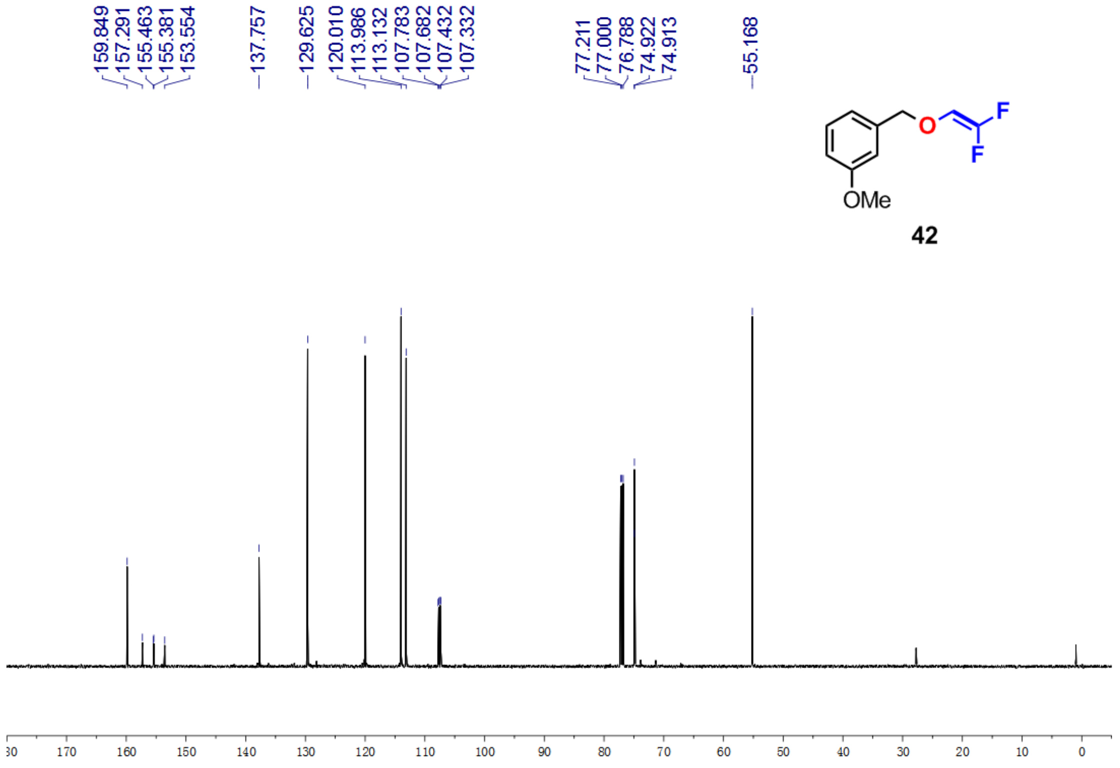

Supplementary Figure 119. ^{13}C NMR of 42

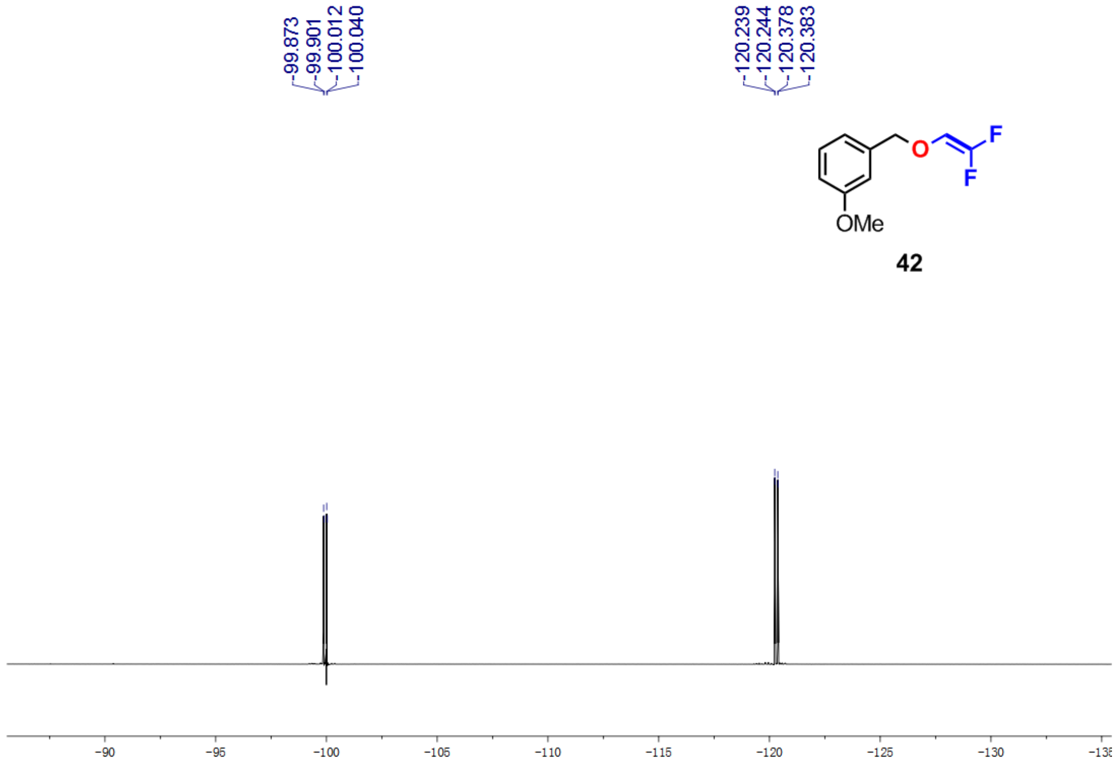

Supplementary Figure 120. ^{19}F NMR of 42

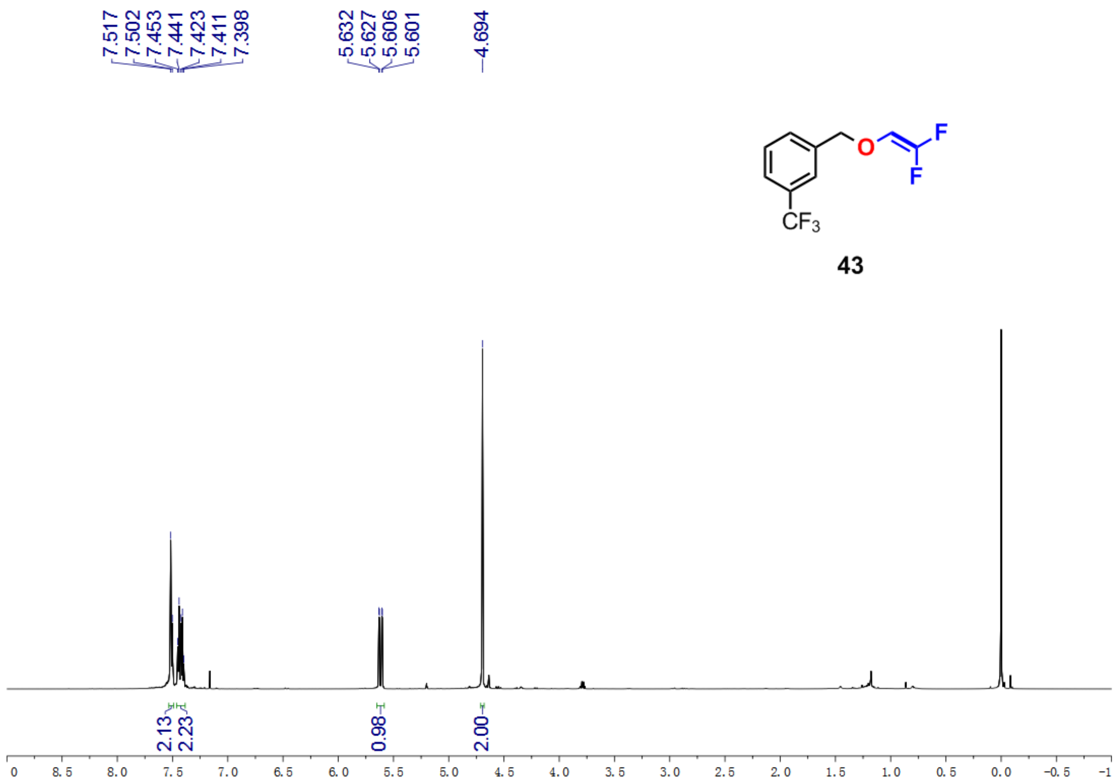

Supplementary Figure 121. ^{1}H NMR of 43

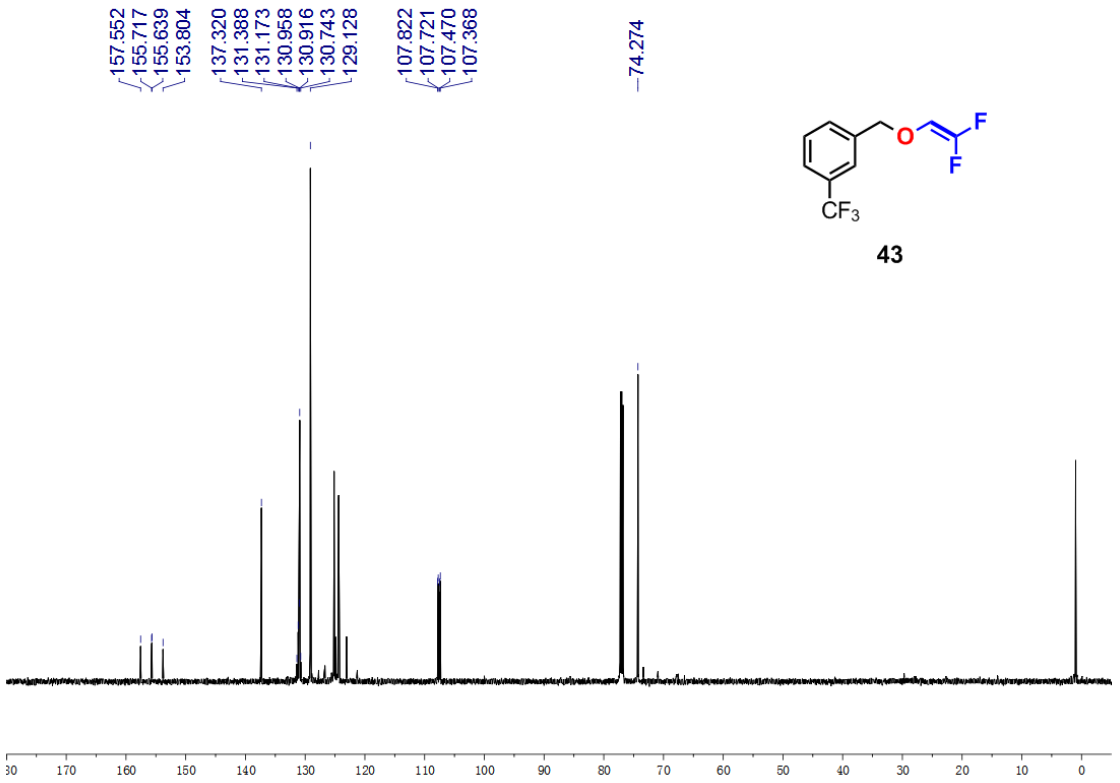

Supplementary Figure 122. ^{13}C NMR of 43

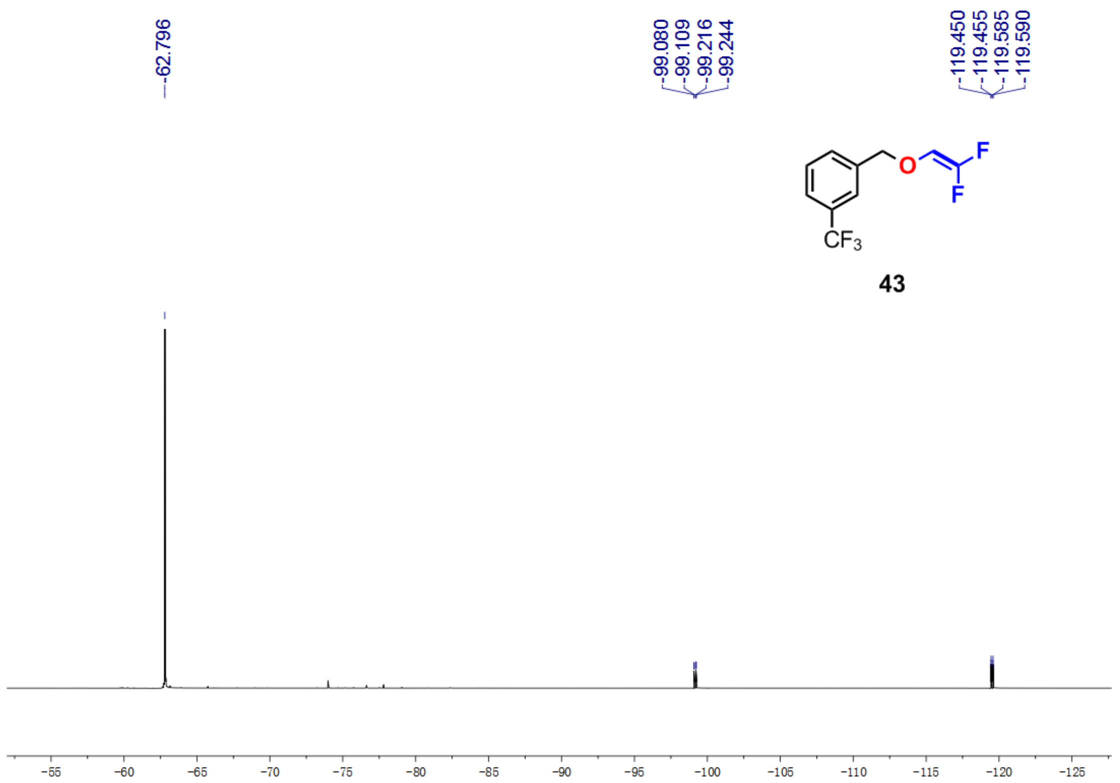

Supplementary Figure 123. ^{19}F NMR of 43

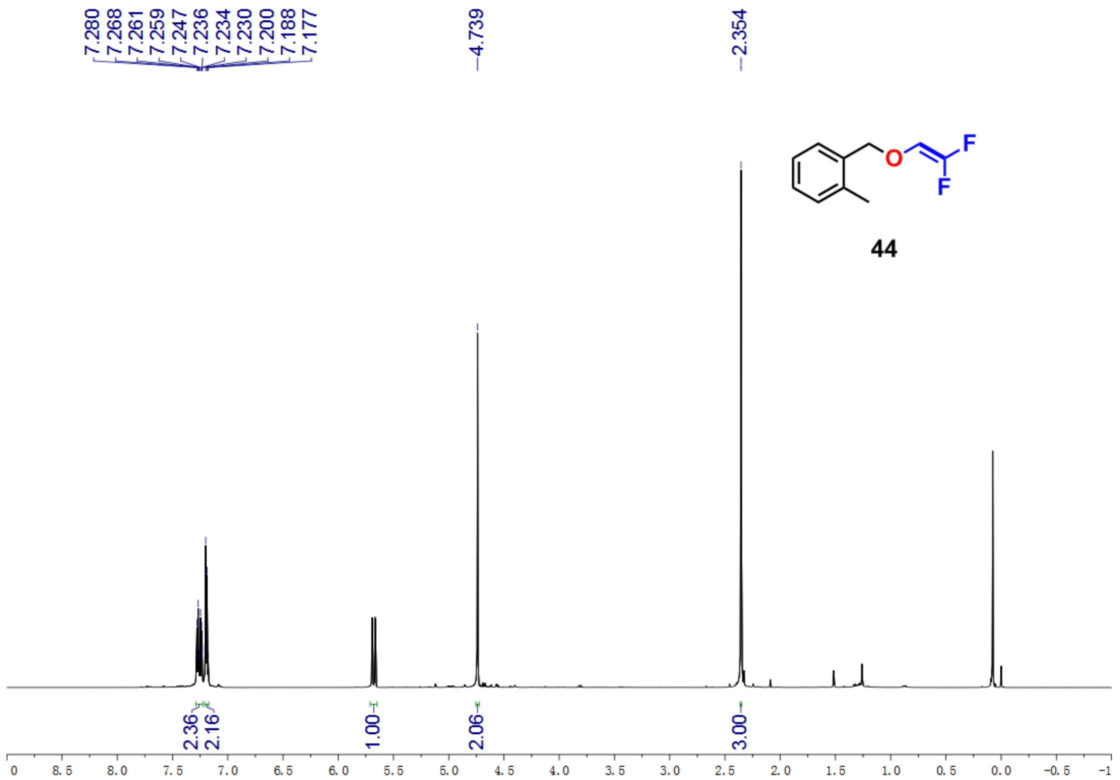

Supplementary Figure 124. ^{1}H NMR of 44

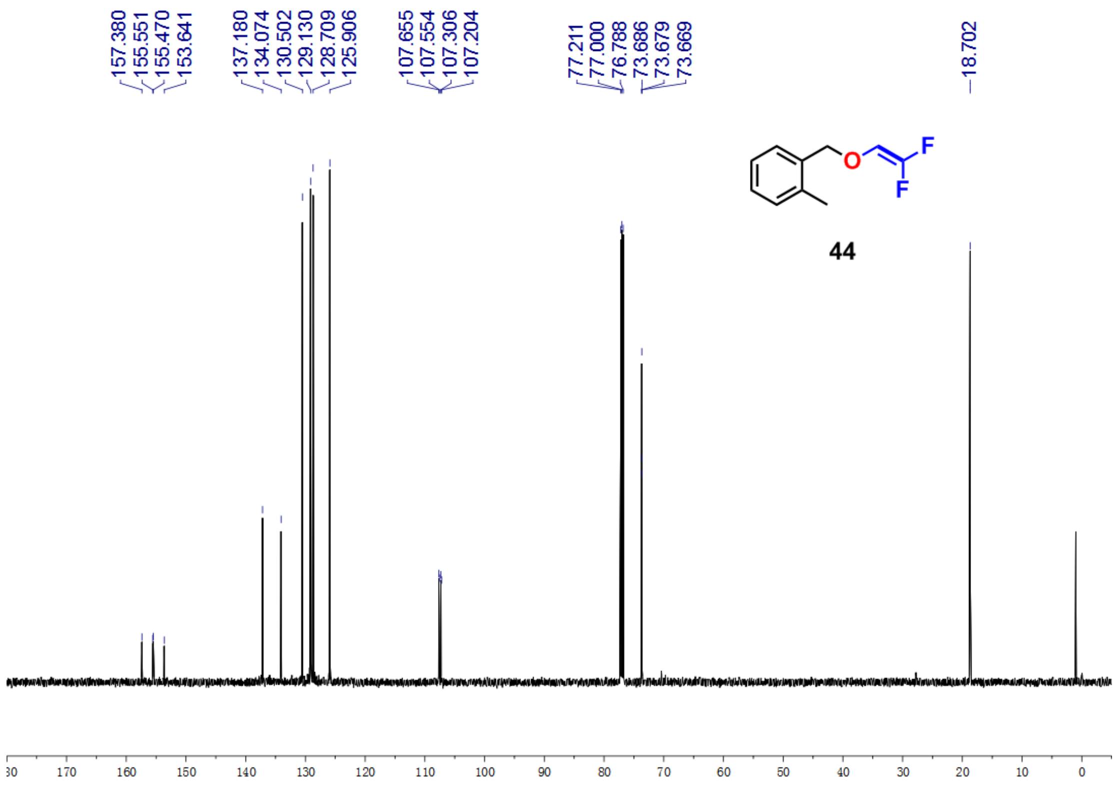

Supplementary Figure 125. ^{13}C NMR of 44

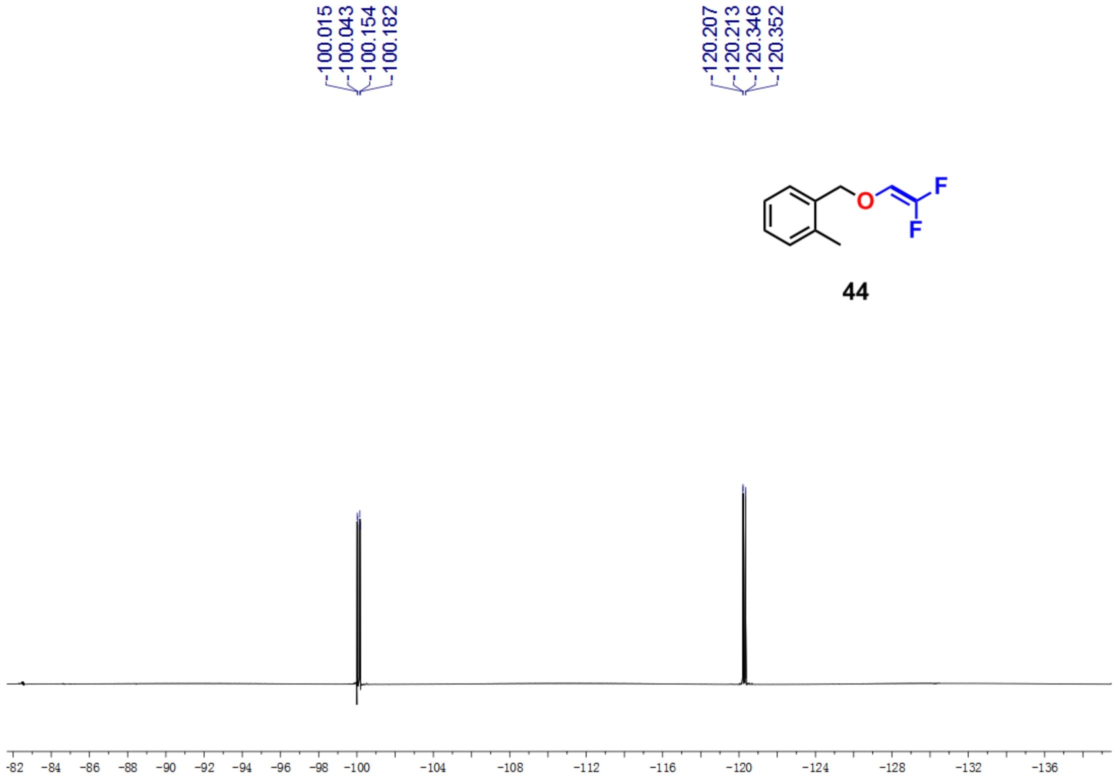

Supplementary Figure 126. ^{19}F NMR of 44

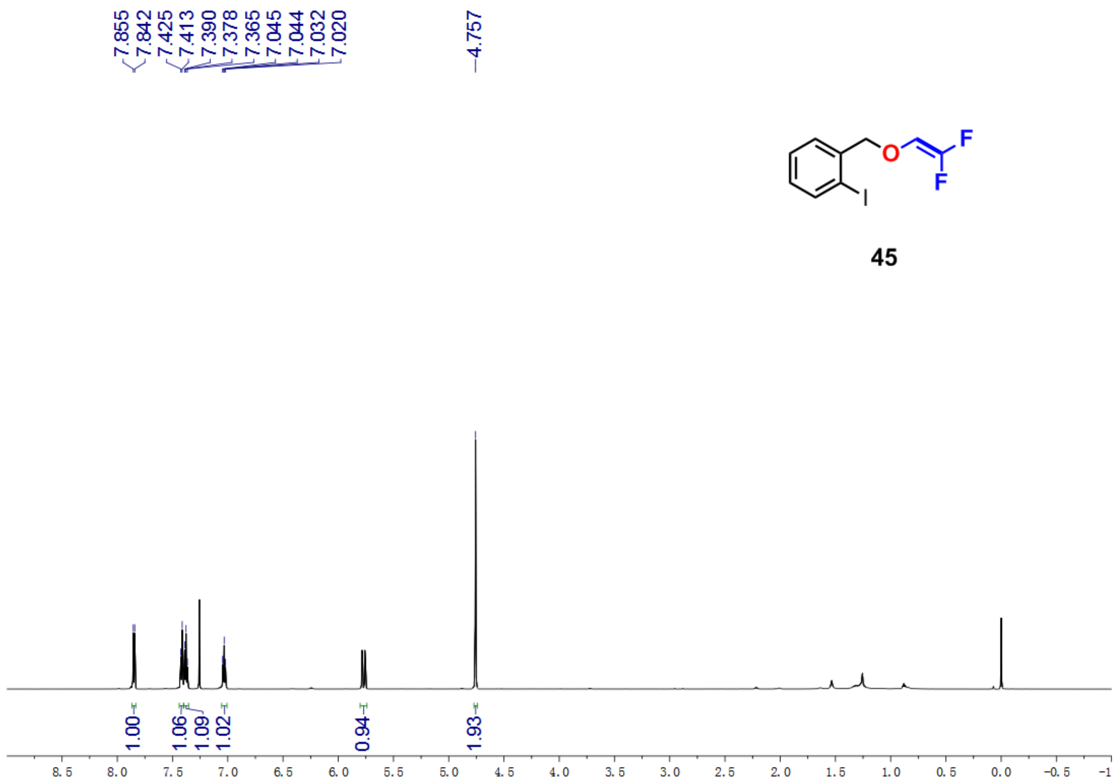

Supplementary Figure 127. ^{1}H NMR of 45

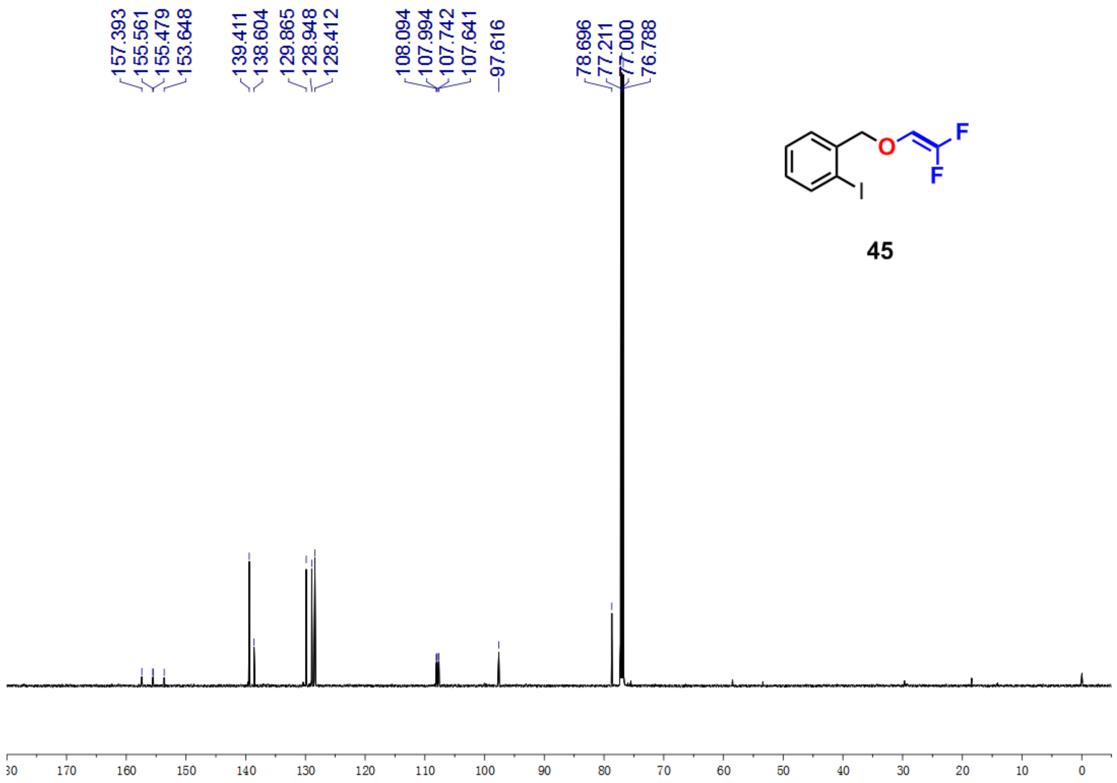

Supplementary Figure 128. ^{13}C NMR of 45

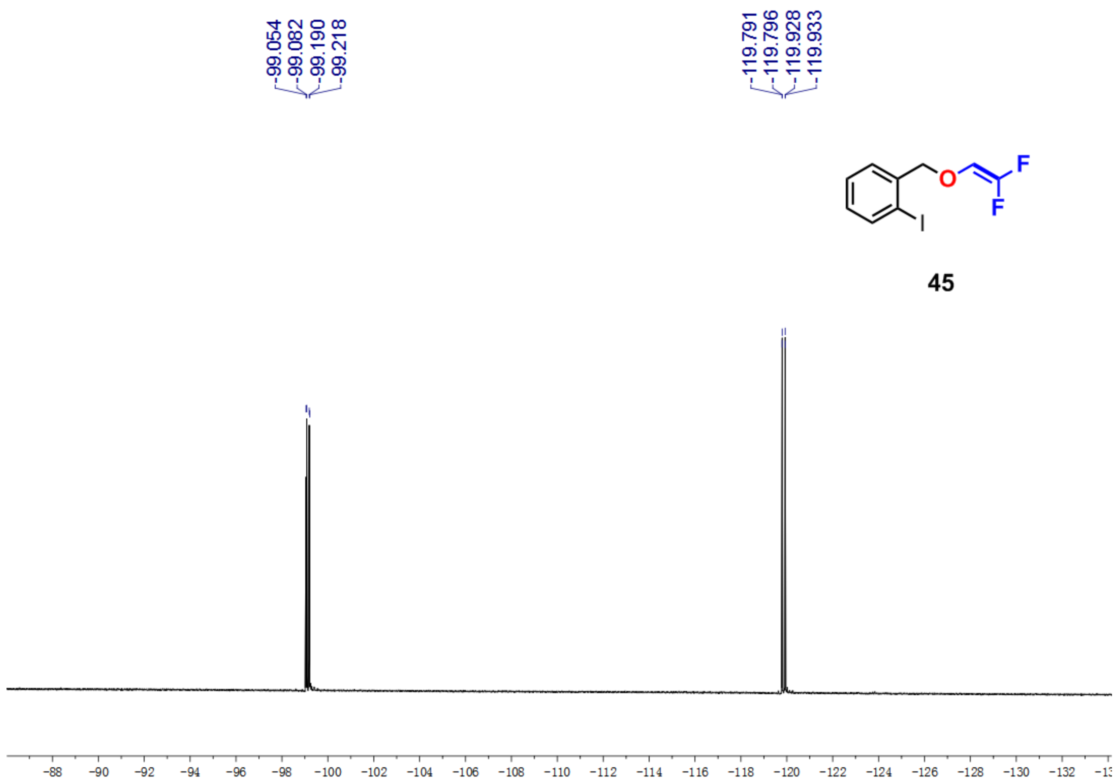

Supplementary Figure 129. ^{19}F NMR of 45

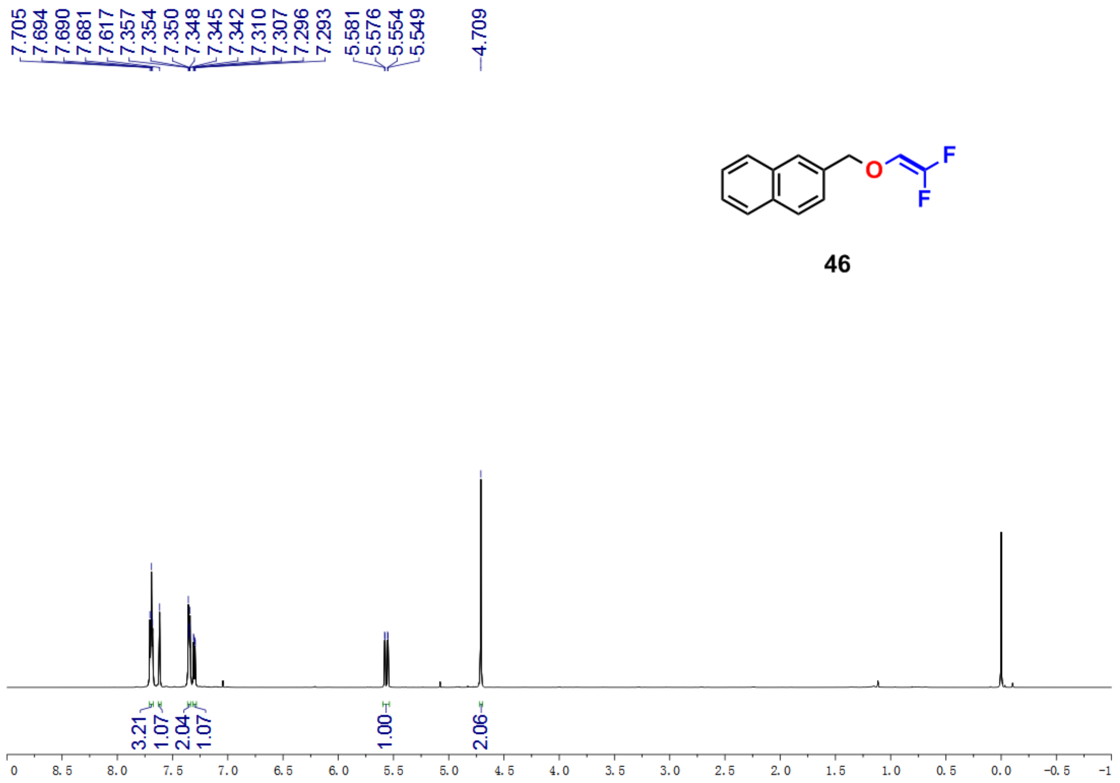

Supplementary Figure 130. ^{1}H NMR of 46

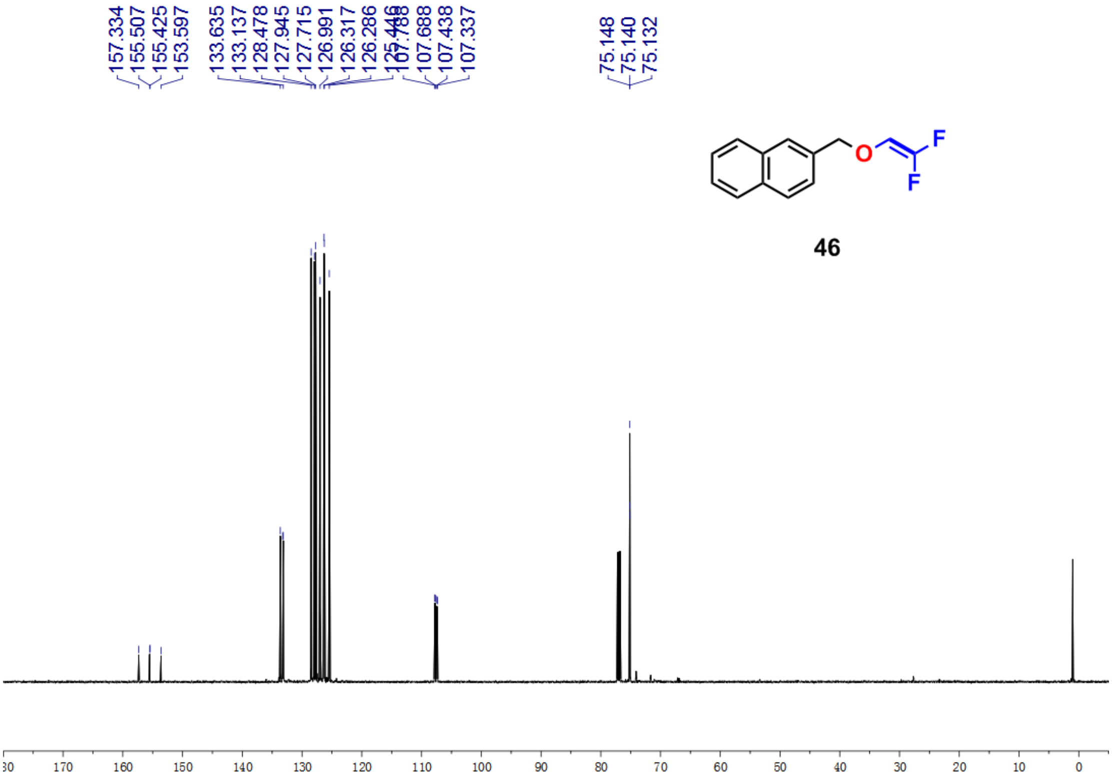

Supplementary Figure 131. ^{13}C NMR of 46

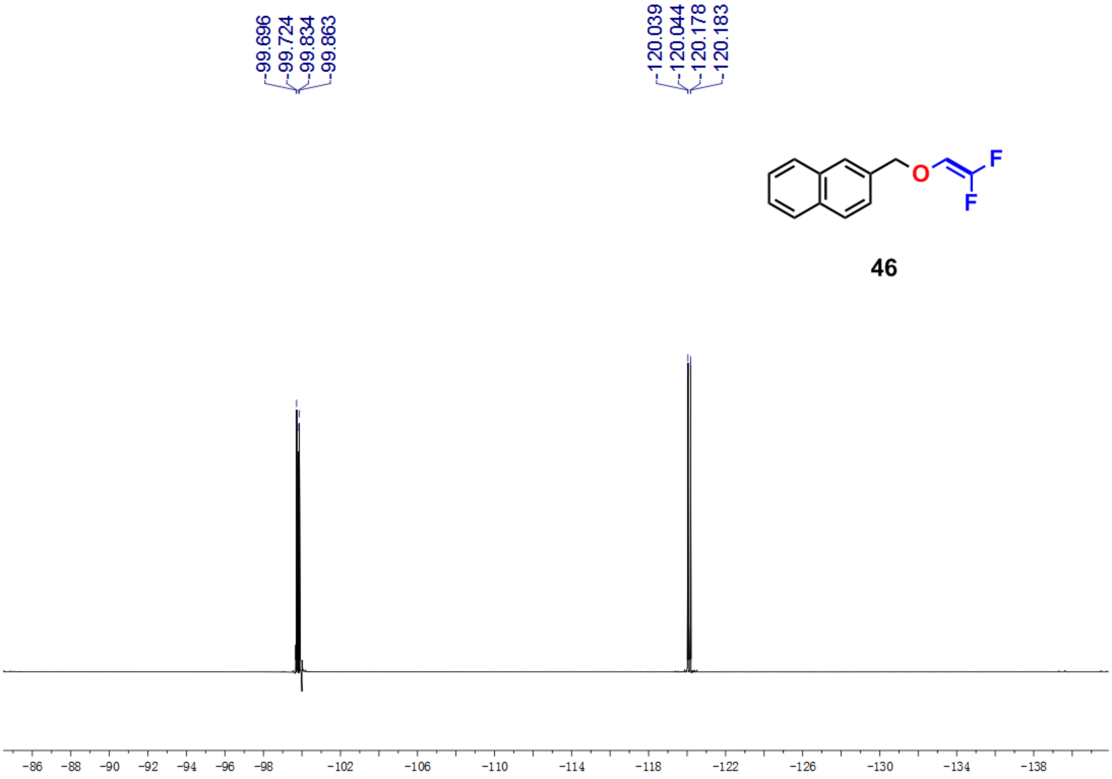

Supplementary Figure 132. ^{19}F NMR of 46

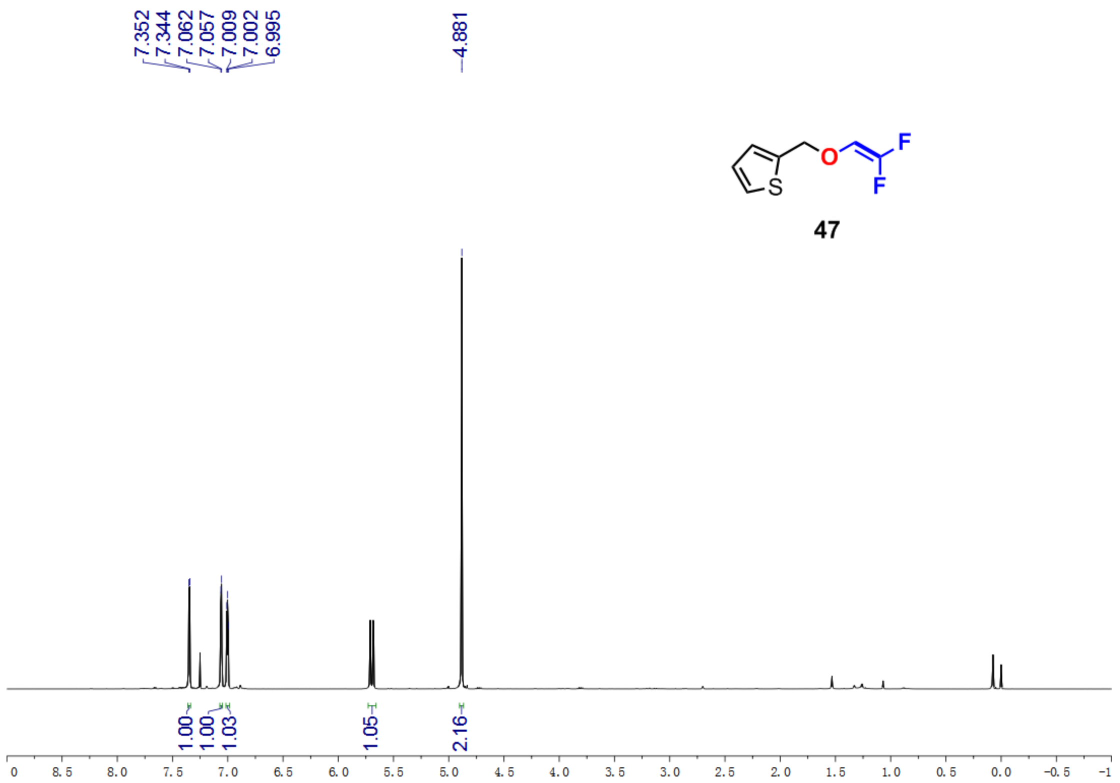

Supplementary Figure 133. ^{1}H NMR of 47

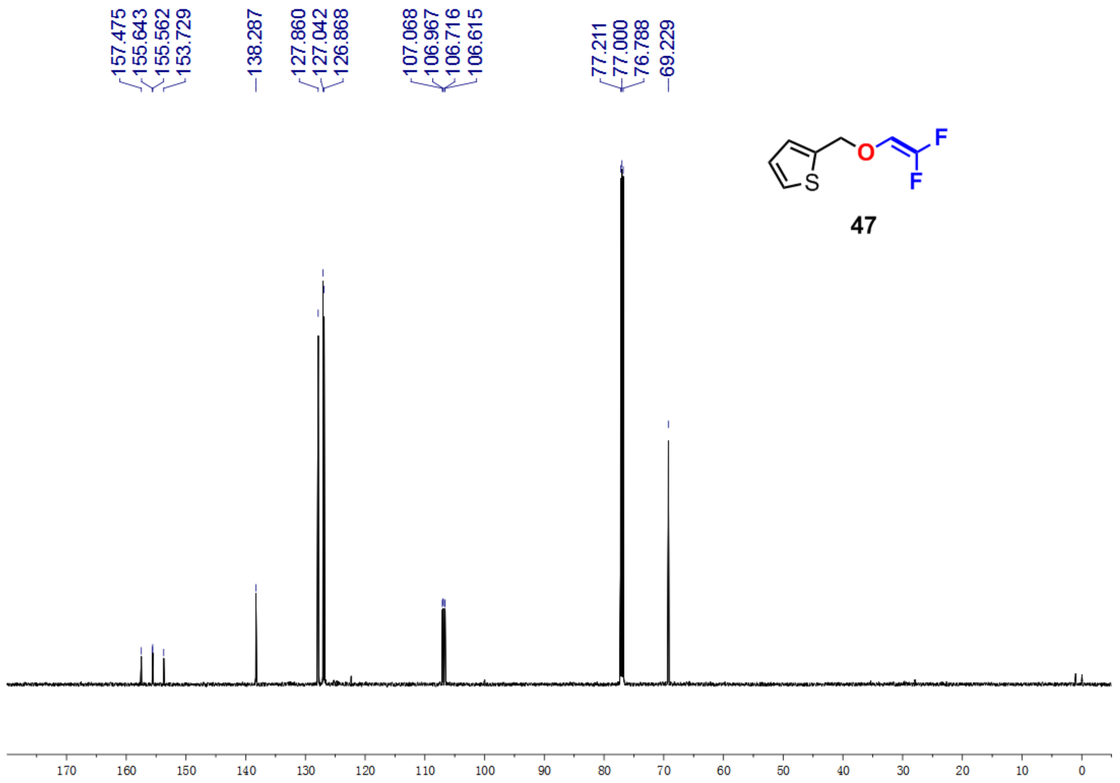

Supplementary Figure 134. ^{13}C NMR of 47

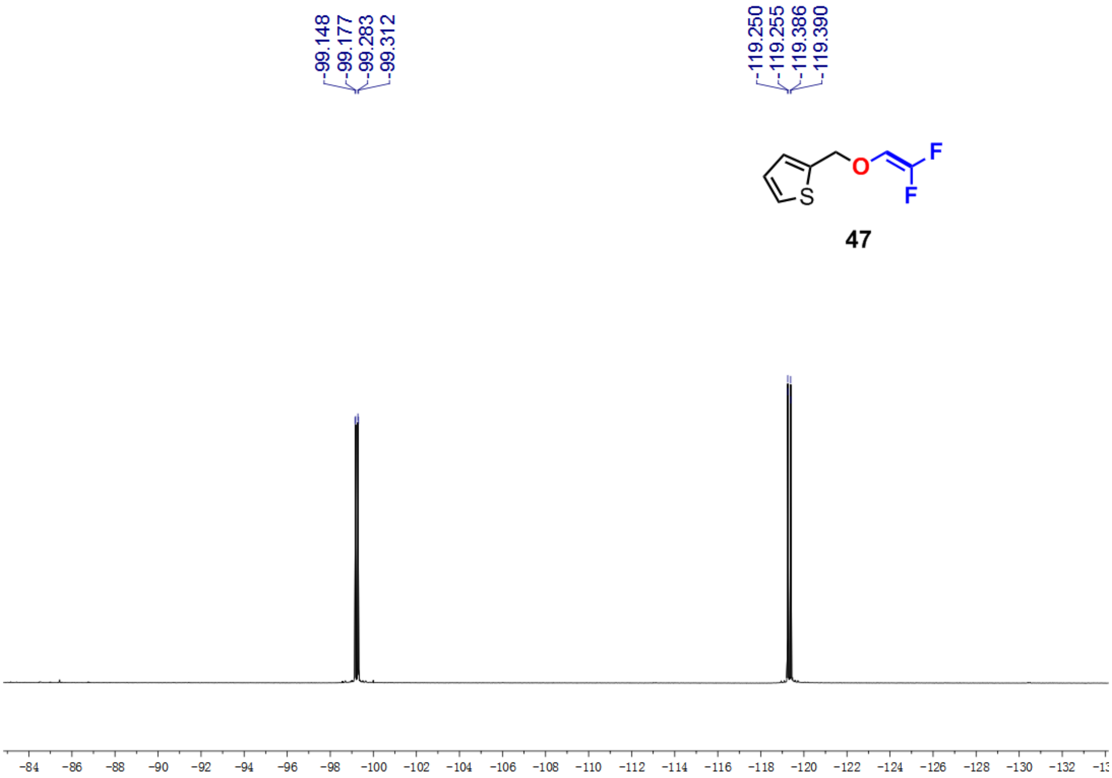

Supplementary Figure 135. ^{19}F NMR of 47

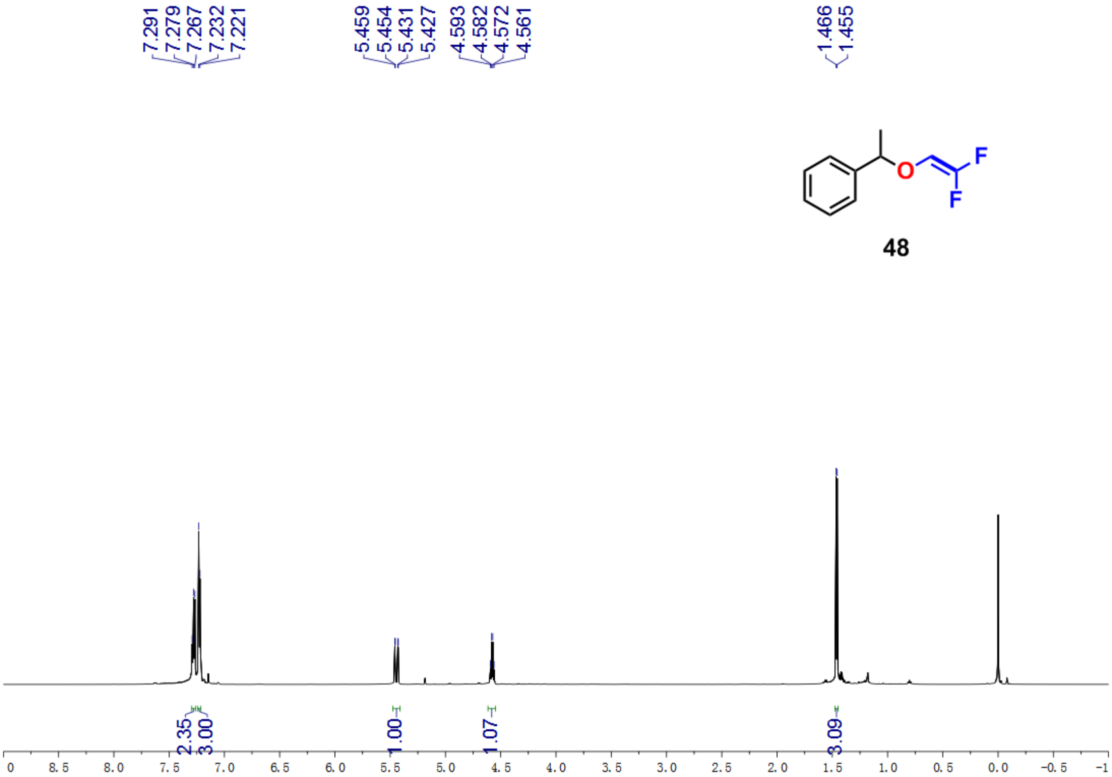

Supplementary Figure 136. ^{1}H NMR of 48

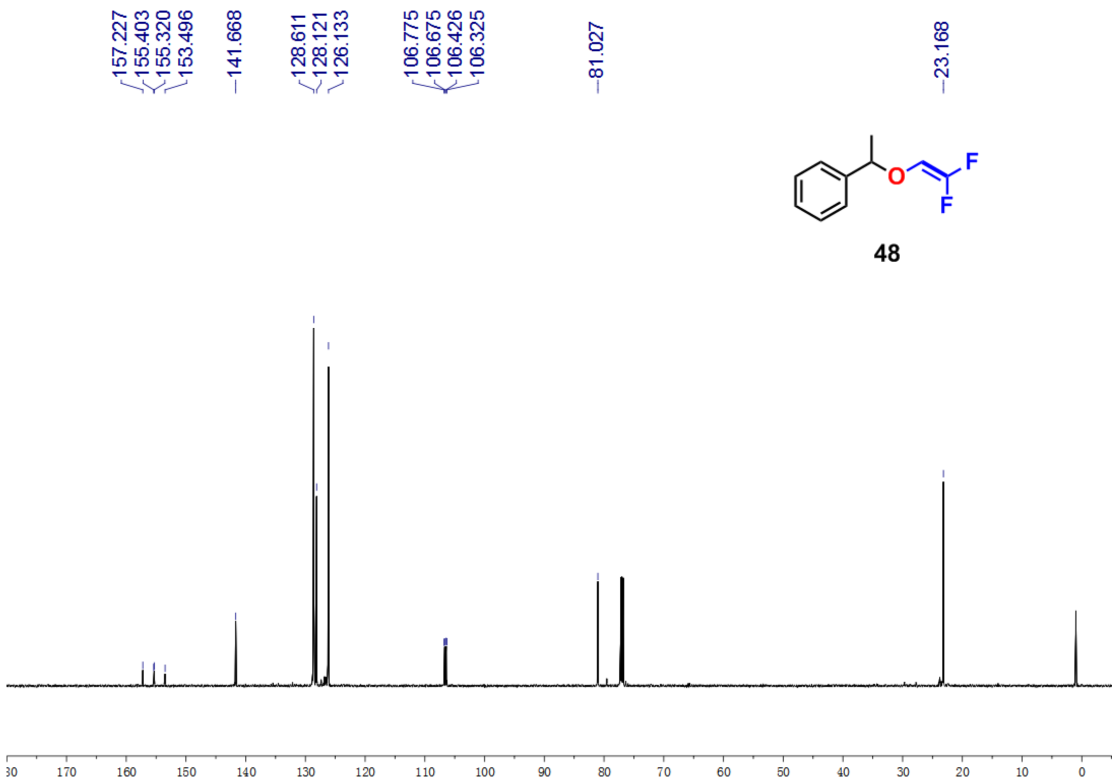

Supplementary Figure 137. ^{13}C NMR of 48

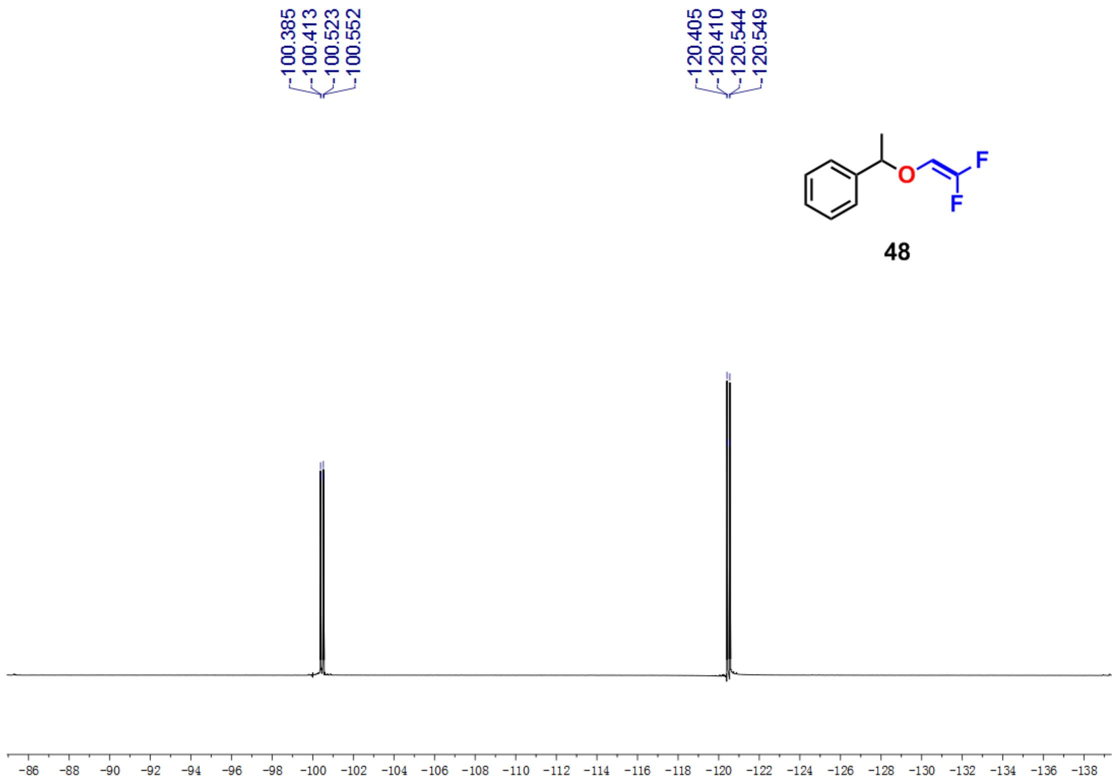

Supplementary Figure 138. ^{19}F NMR of 48

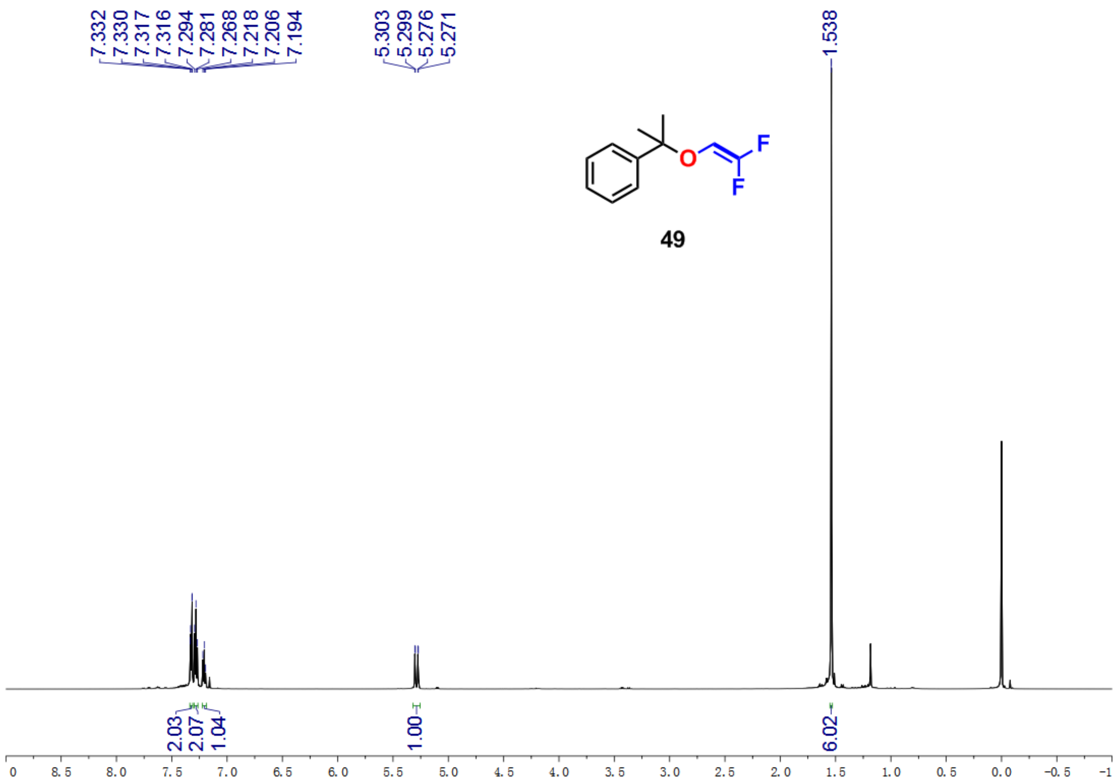

Supplementary Figure 139. ^{1}H NMR of 49

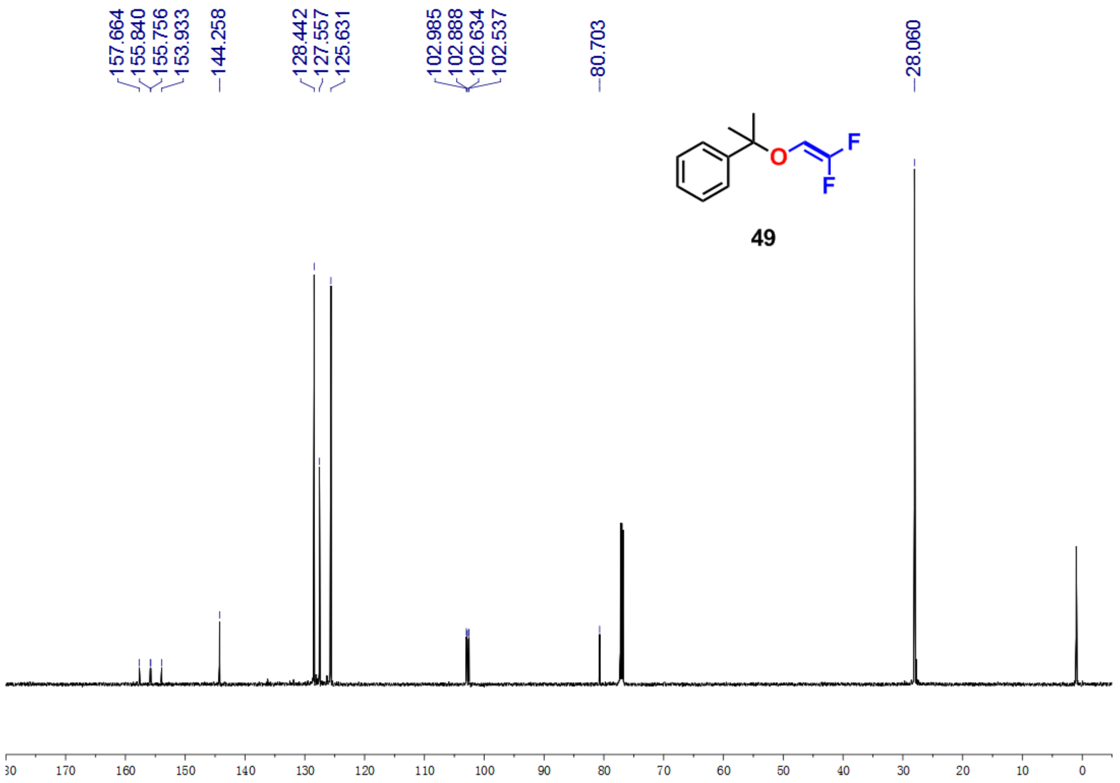

Supplementary Figure 140. ^{13}C NMR of 49

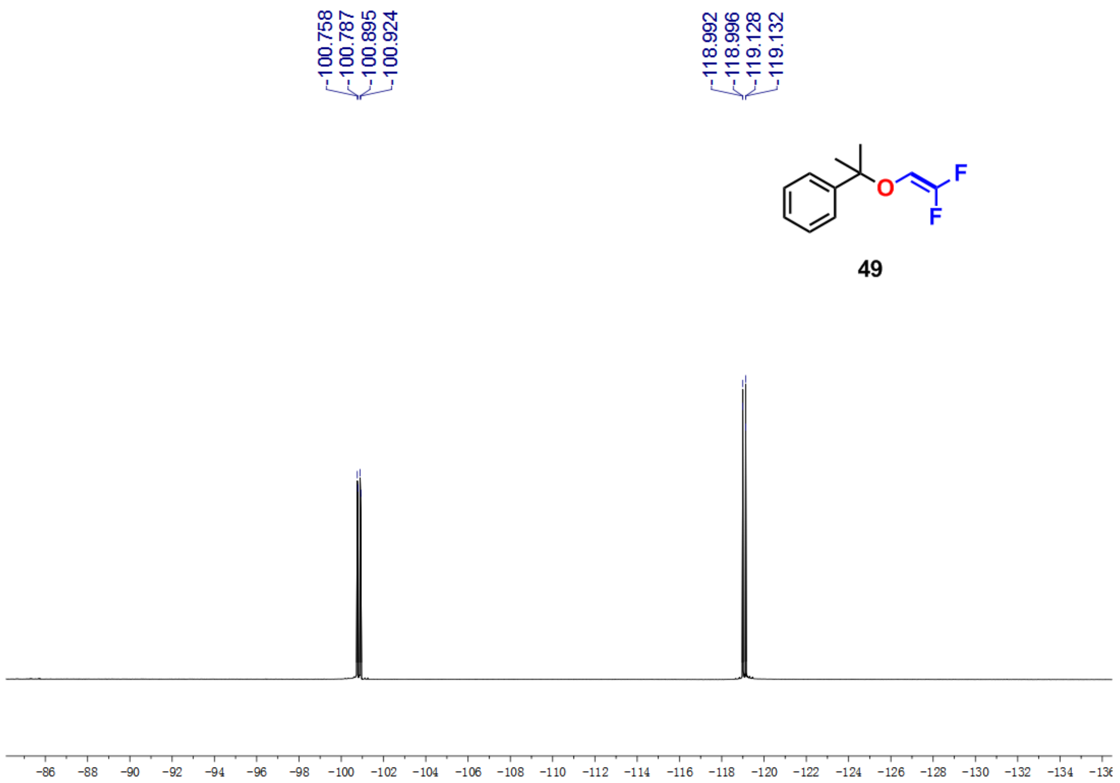

Supplementary Figure 141. ^{19}F NMR of 49

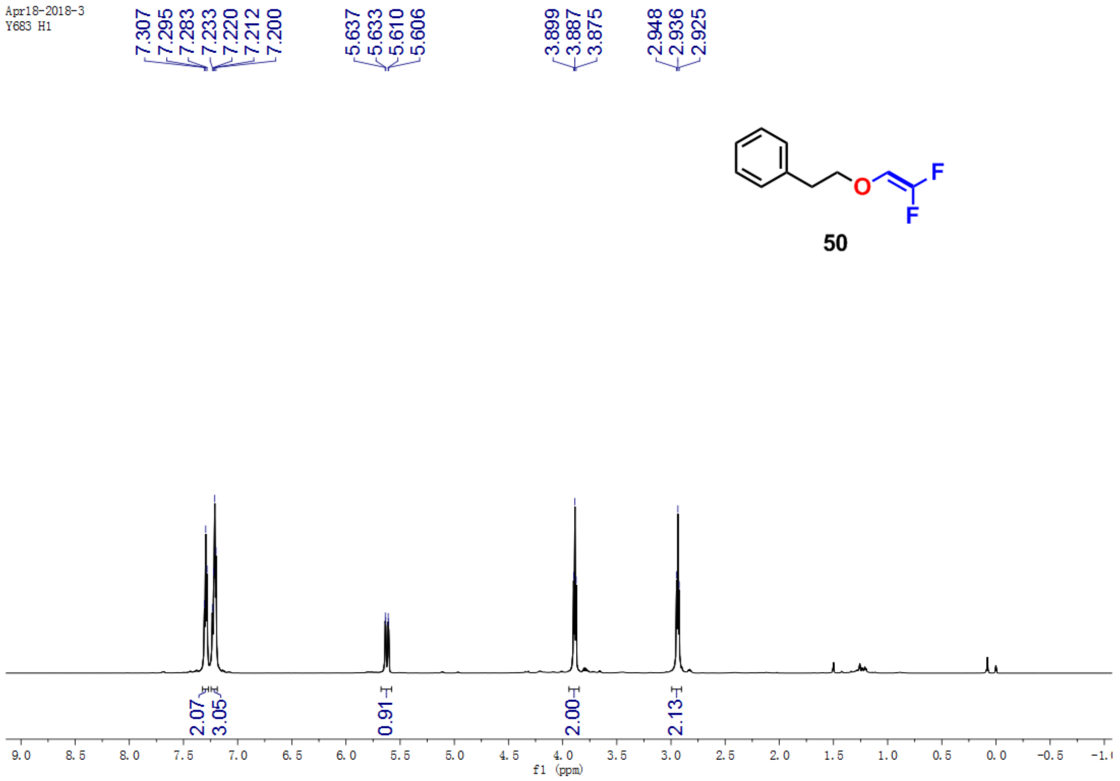

Supplementary Figure 142. ^{1}H NMR of 50

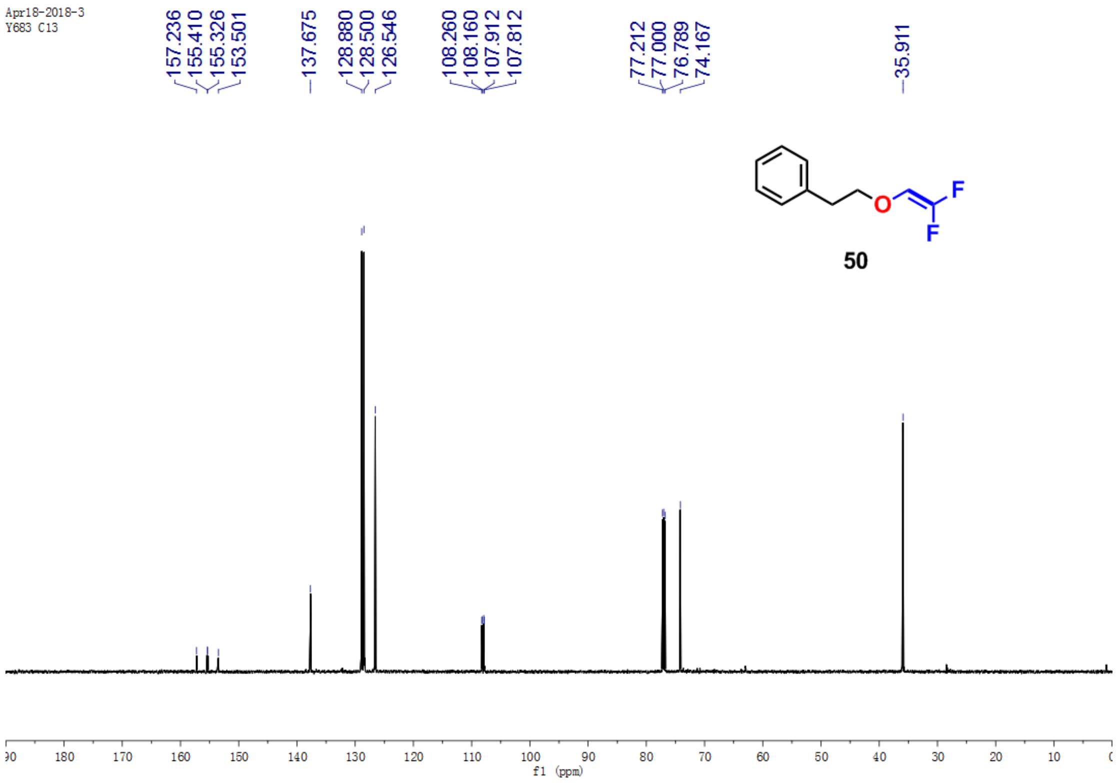

Supplementary Figure 143. ^{13}C NMR of 50

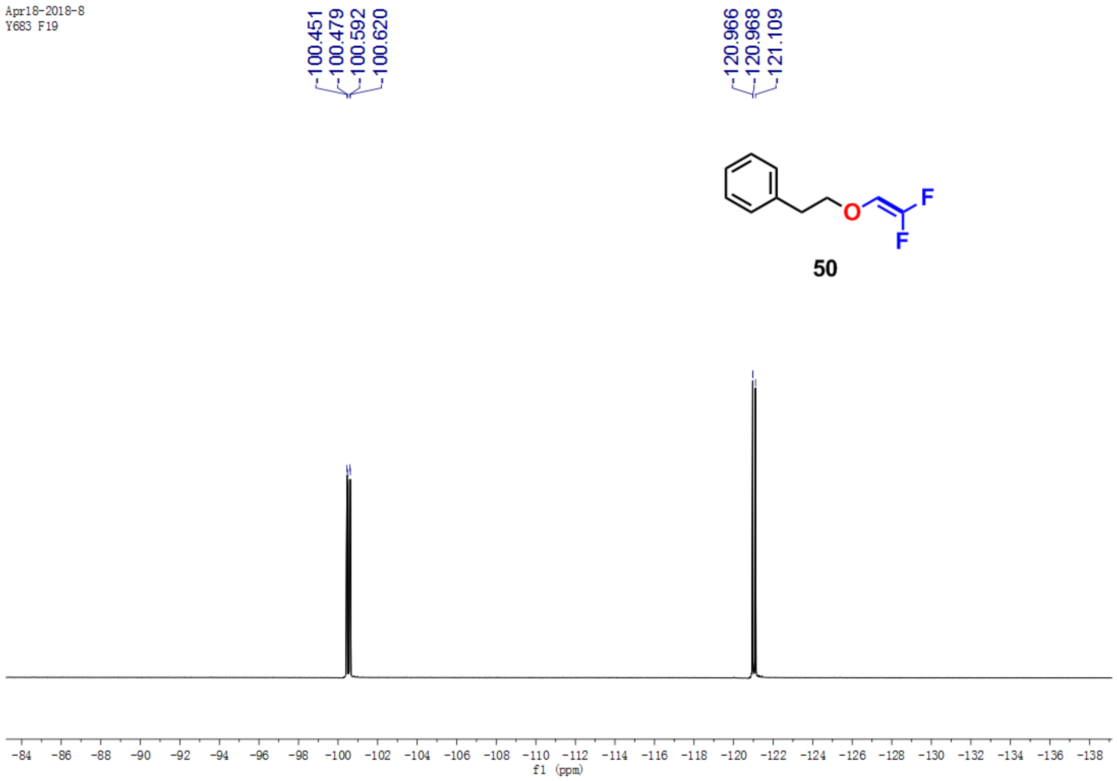

Supplementary Figure 144. ^{19}F NMR of 50

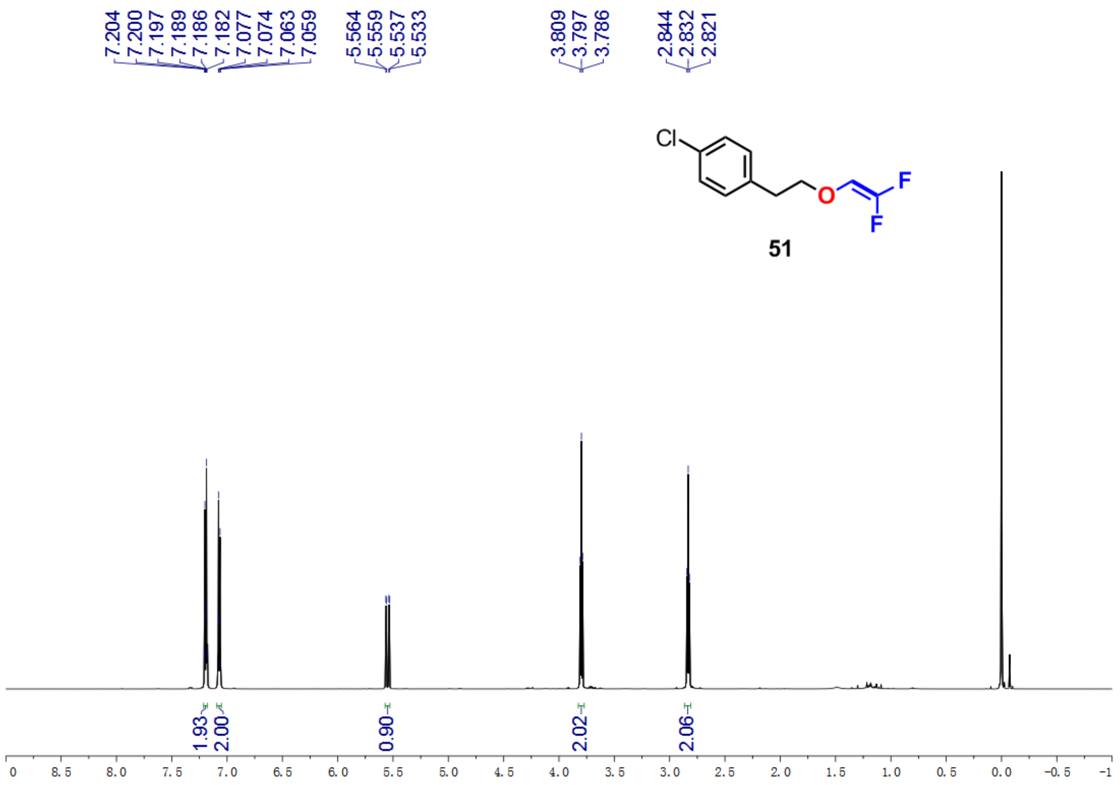

Supplementary Figure 145. ^{1}H NMR of 51

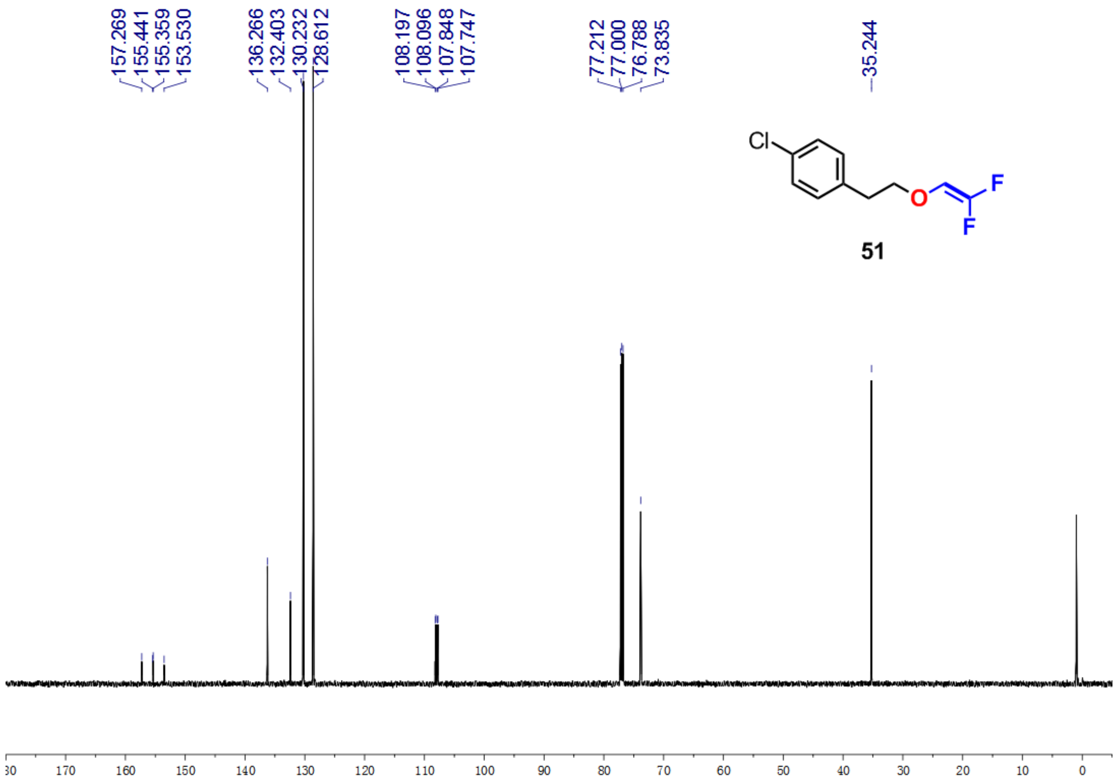

Supplementary Figure 146. ^{13}C NMR of 51

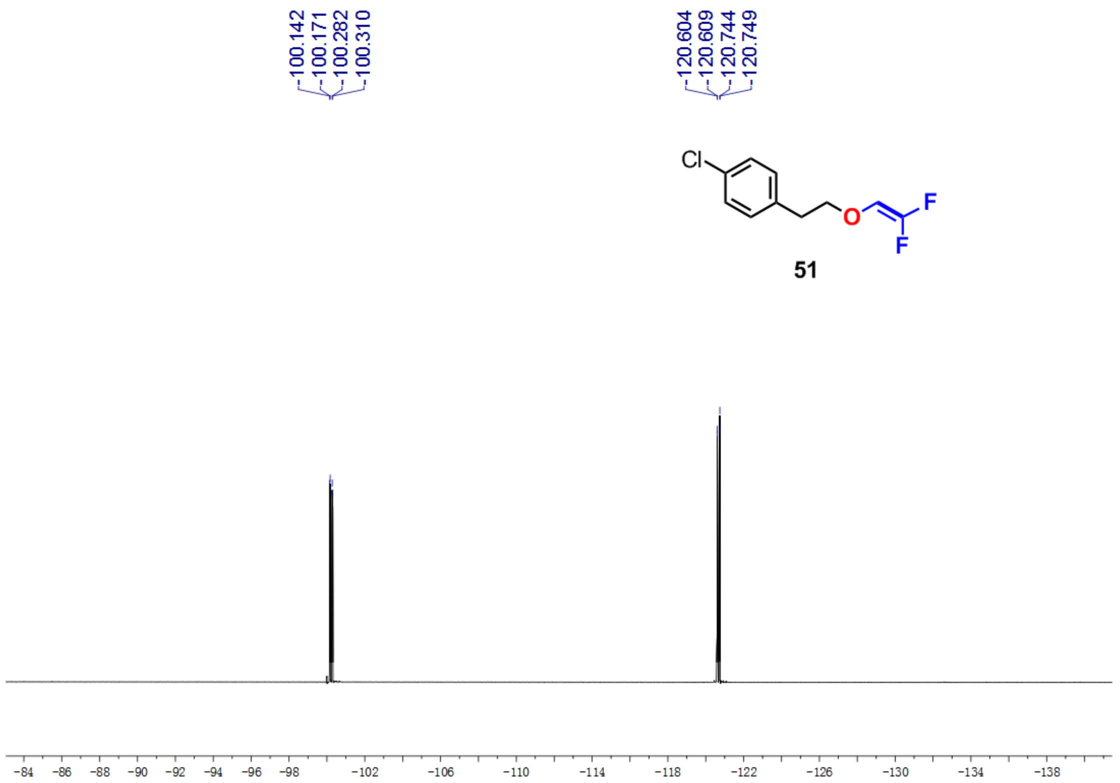

Supplementary Figure 147. ^{19}F NMR of 51

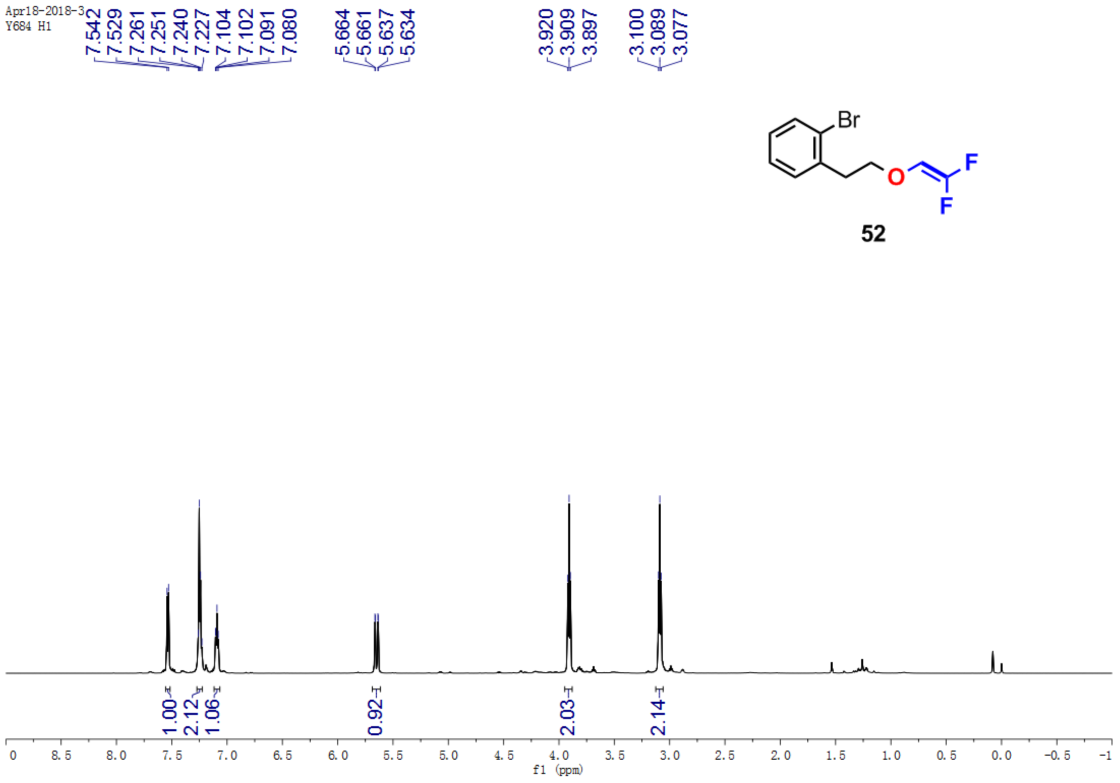

Supplementary Figure 148. ^{1}H NMR of 52

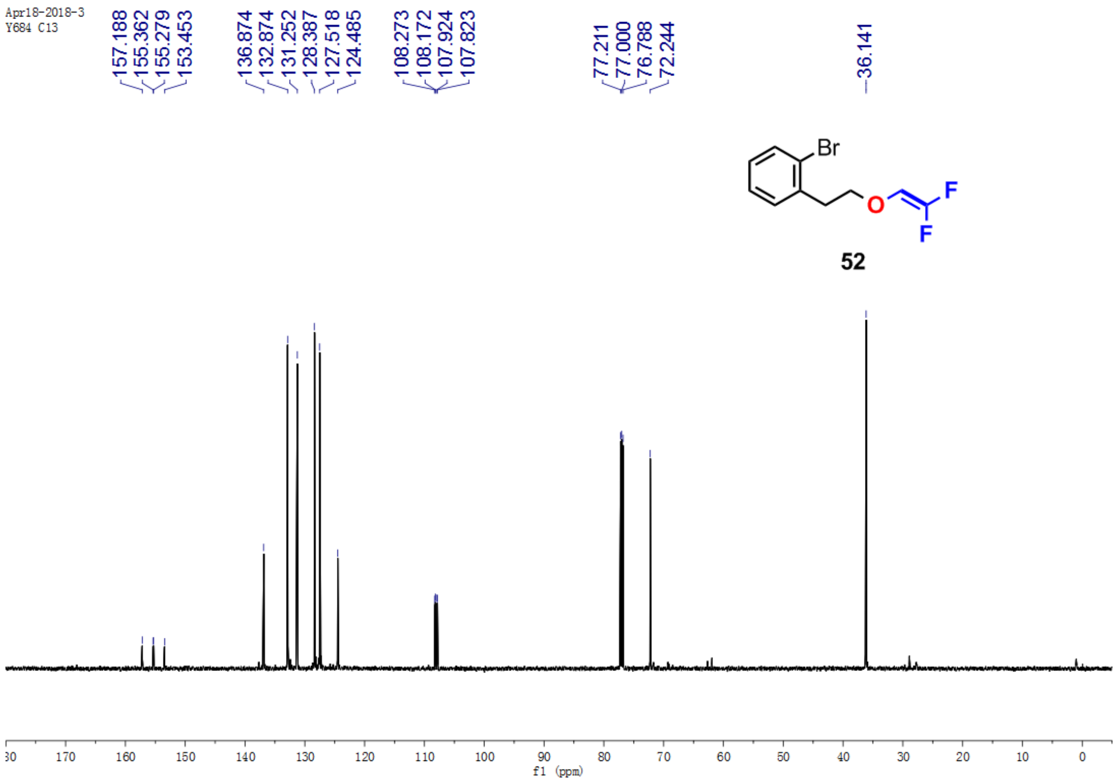

Supplementary Figure 149. ^{13}C NMR of 52

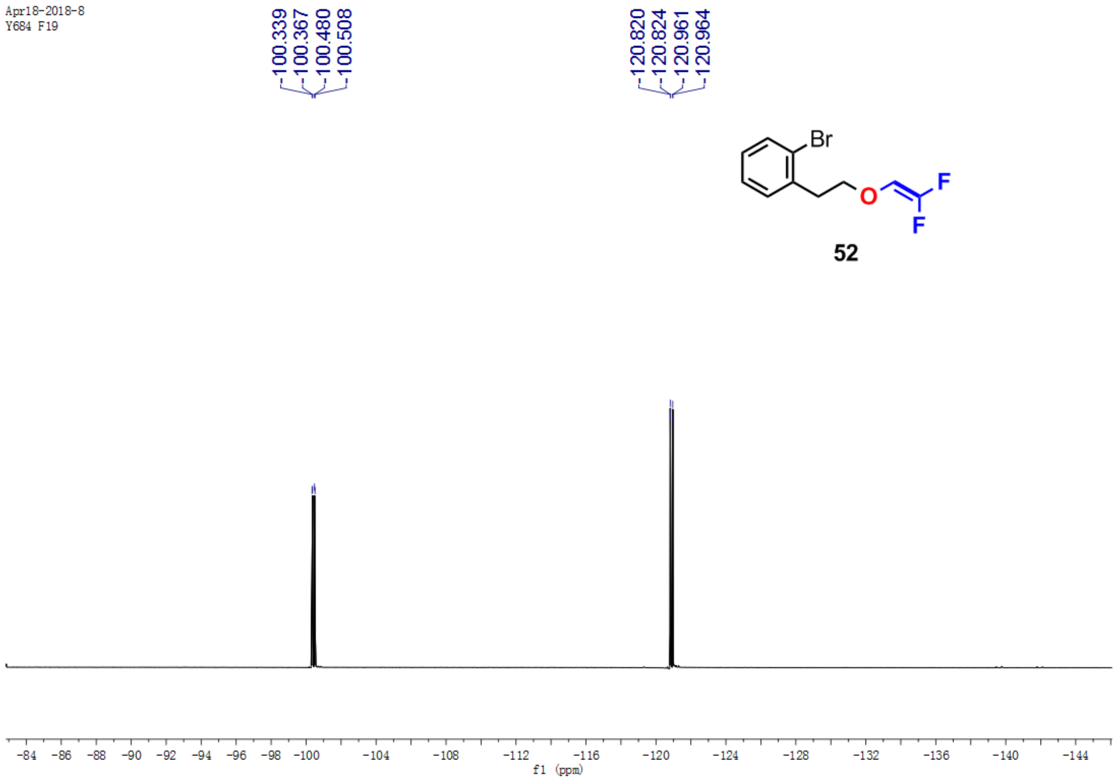

Supplementary Figure 150. ^{19}F NMR of 52

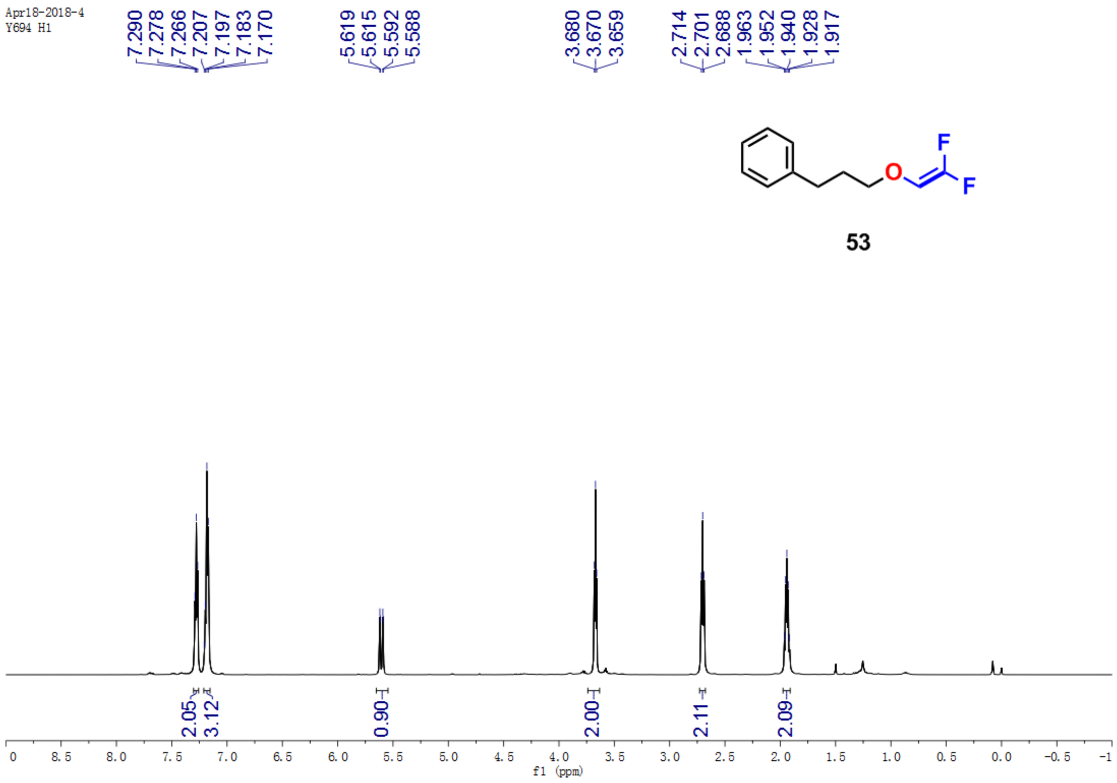

Supplementary Figure 151. ^{1}H NMR of 53

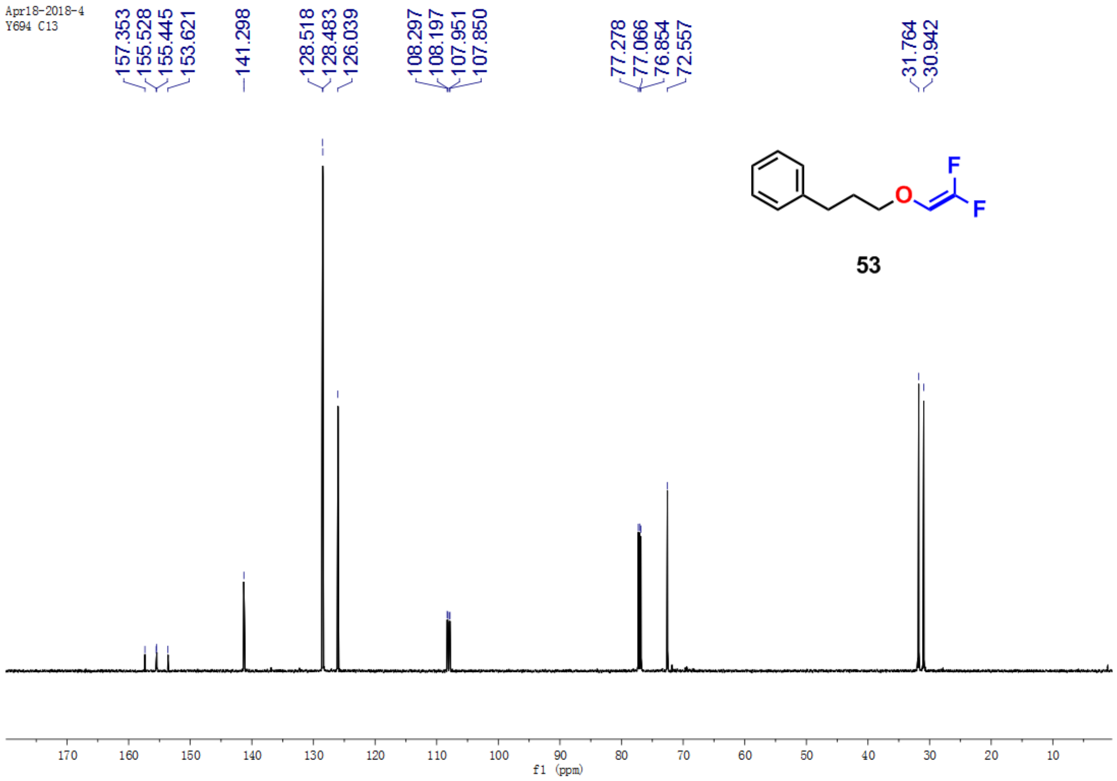

Supplementary Figure 152. ^{13}C NMR of 53

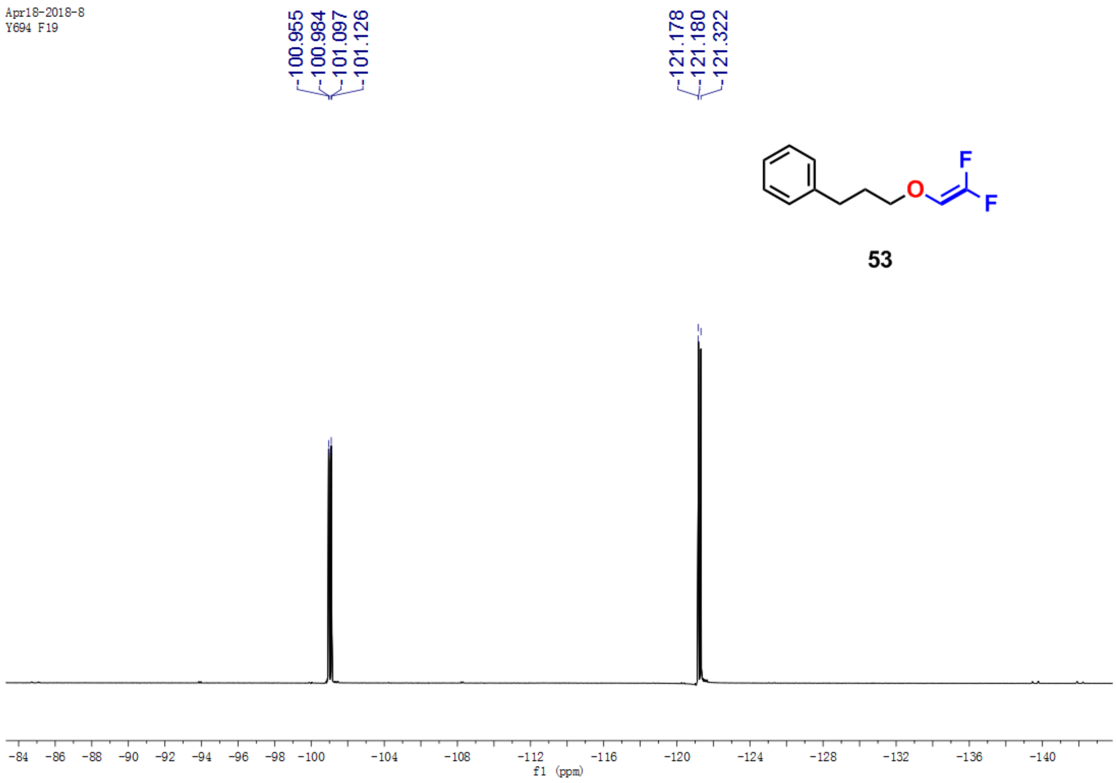

Supplementary Figure 153. ^{19}F NMR of 53

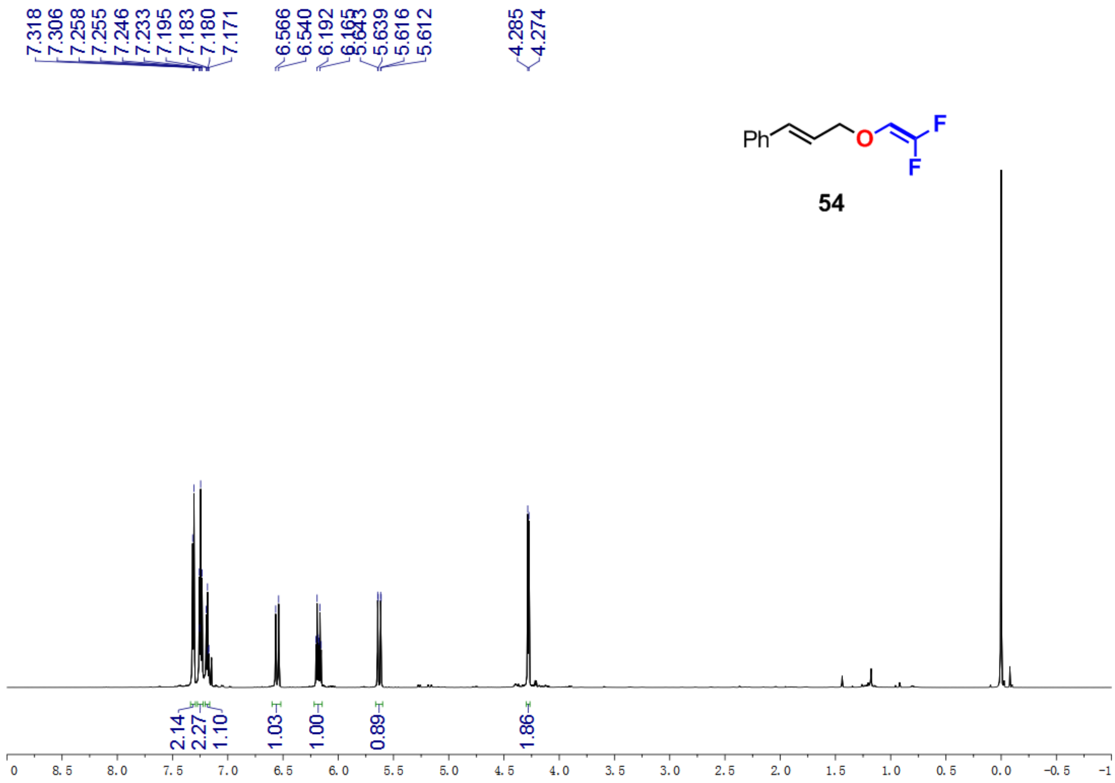

Supplementary Figure 154. ^{1}H NMR of 54

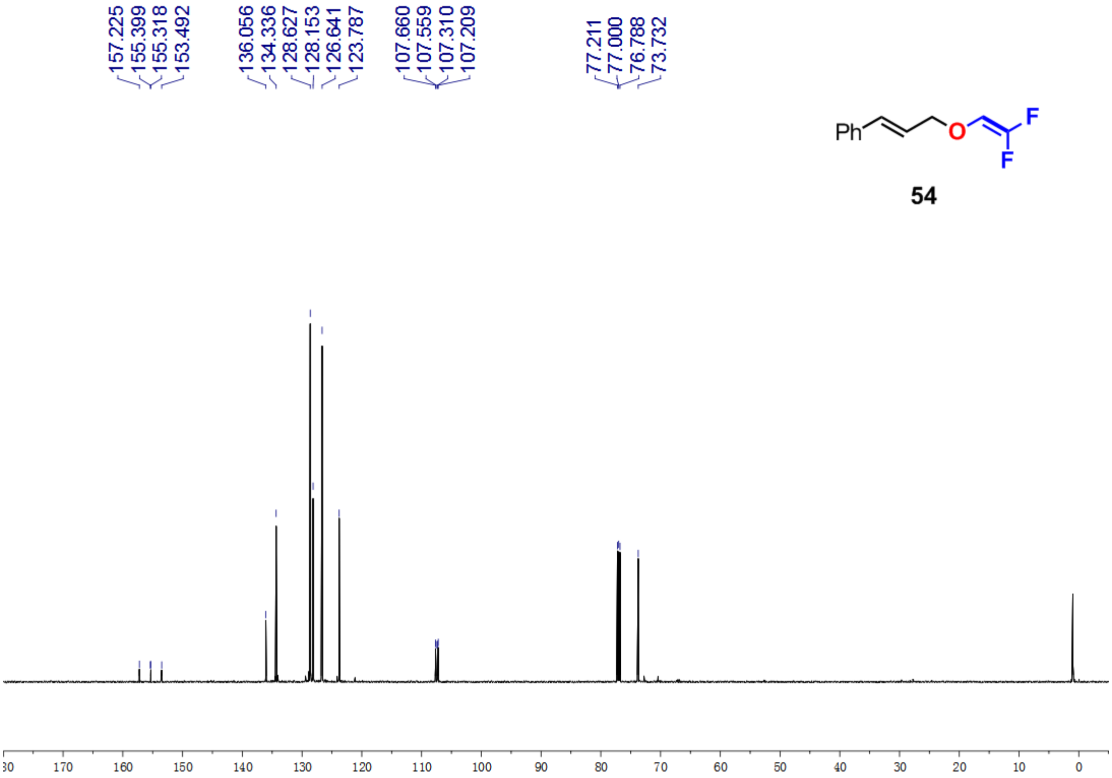

Supplementary Figure 155. ^{13}C NMR of 54

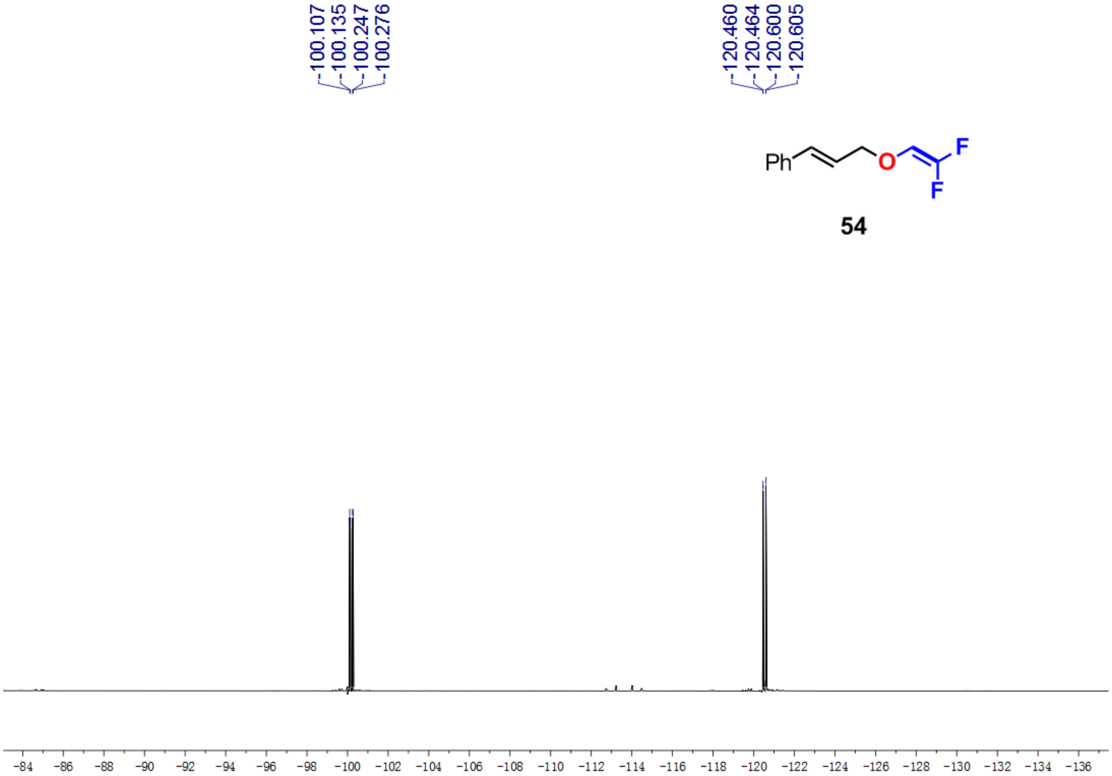

Supplementary Figure 156. ^{19}F NMR of 54

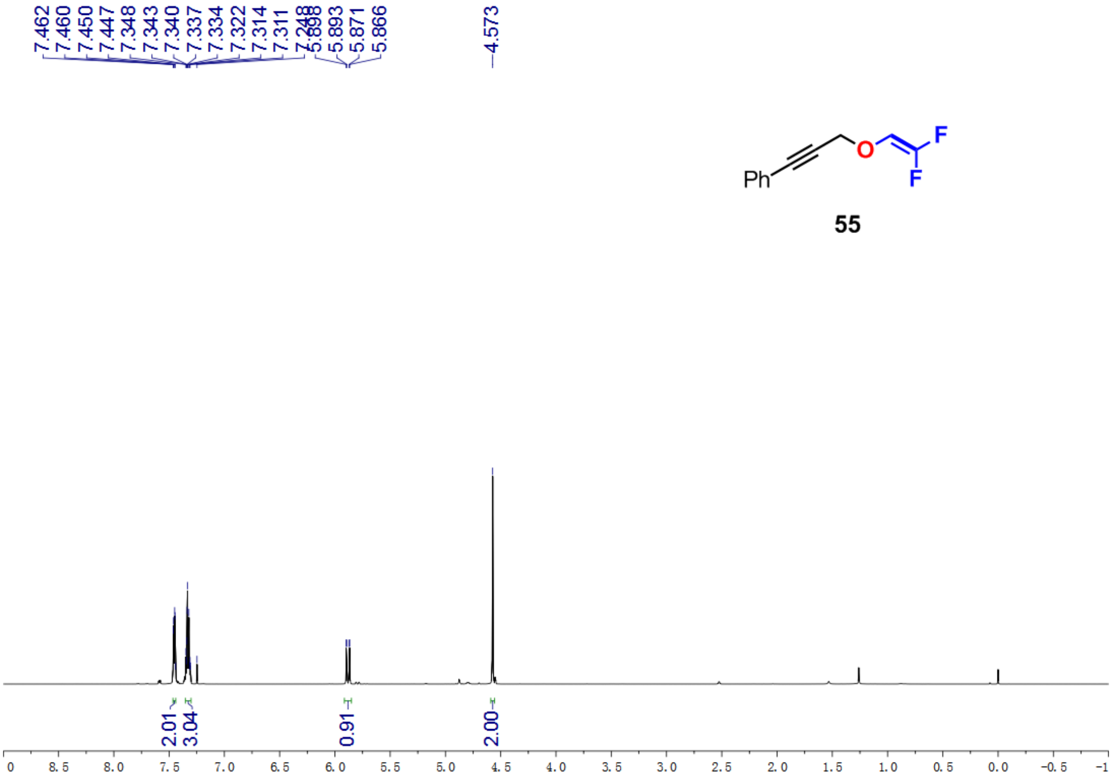

Supplementary Figure 157. ^{1}H NMR of 55

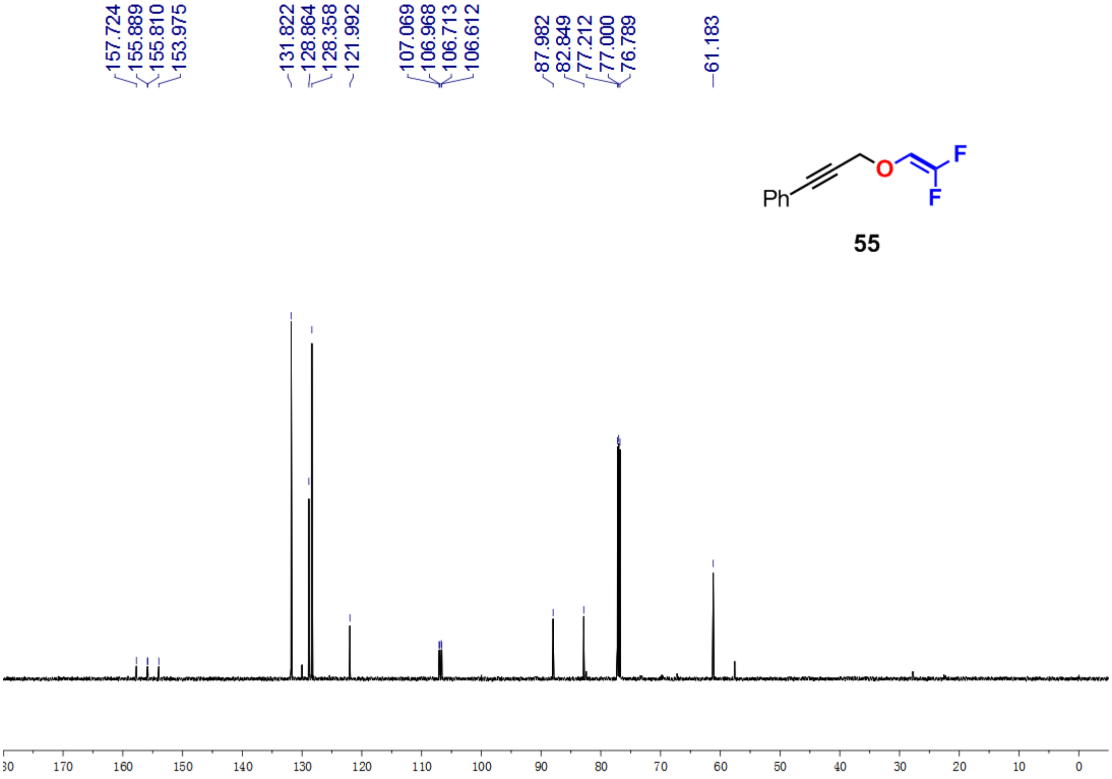

Supplementary Figure 158. ^{13}C NMR of 55

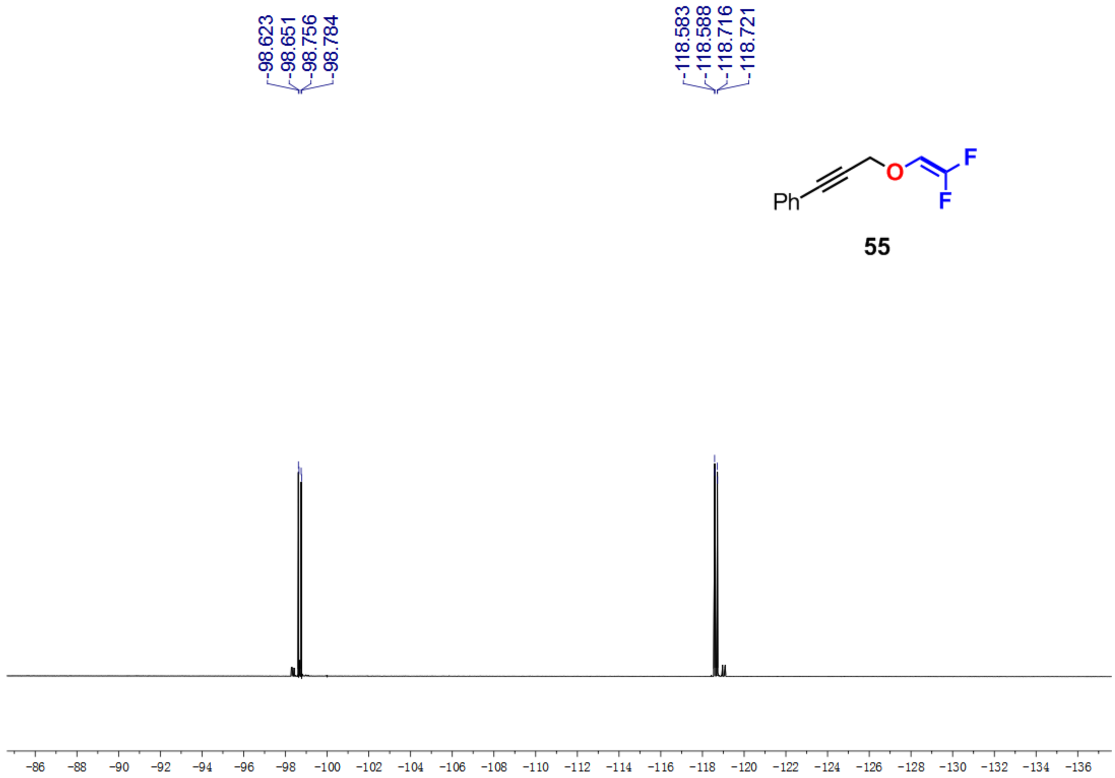

Supplementary Figure 159. ^{19}F NMR of 55

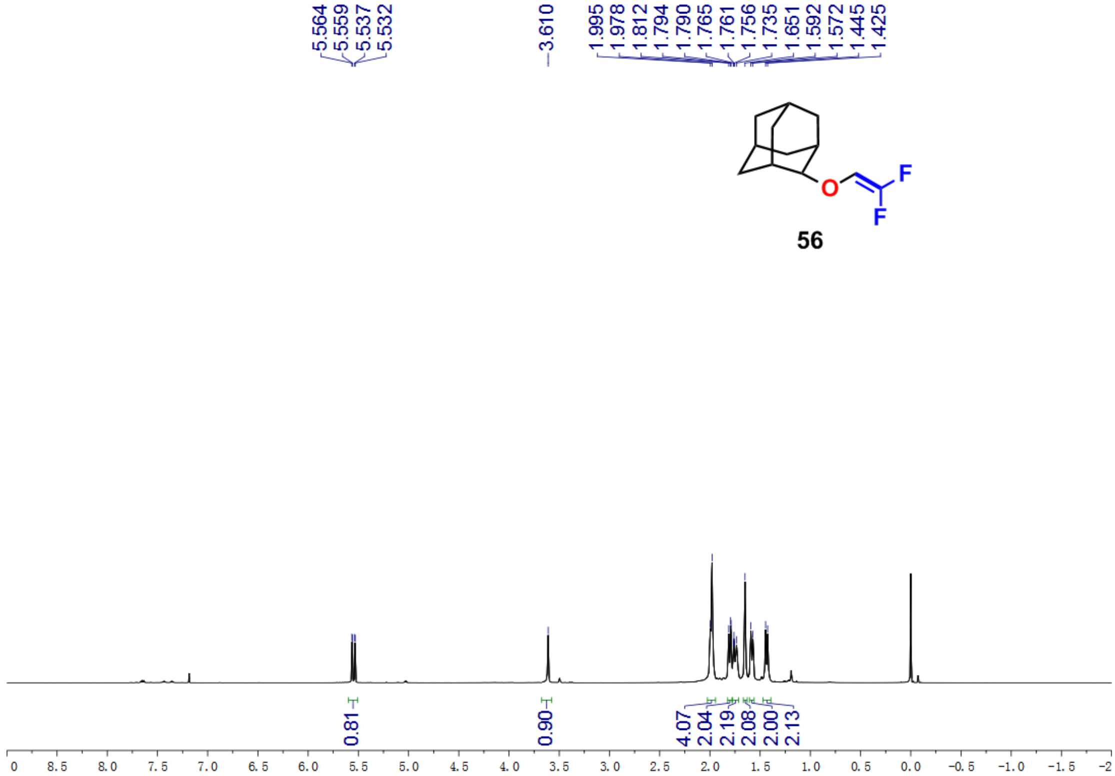

Supplementary Figure 160. ^{1}H NMR of 56

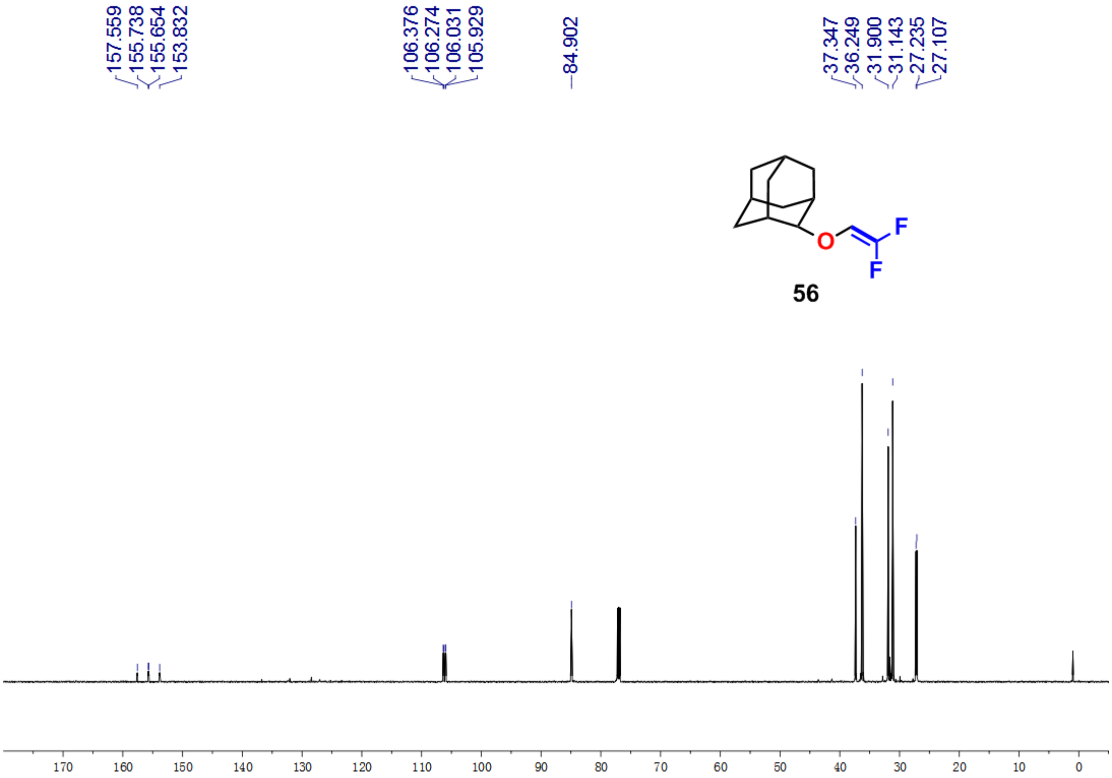

Supplementary Figure 161. ^{13}C NMR of 56

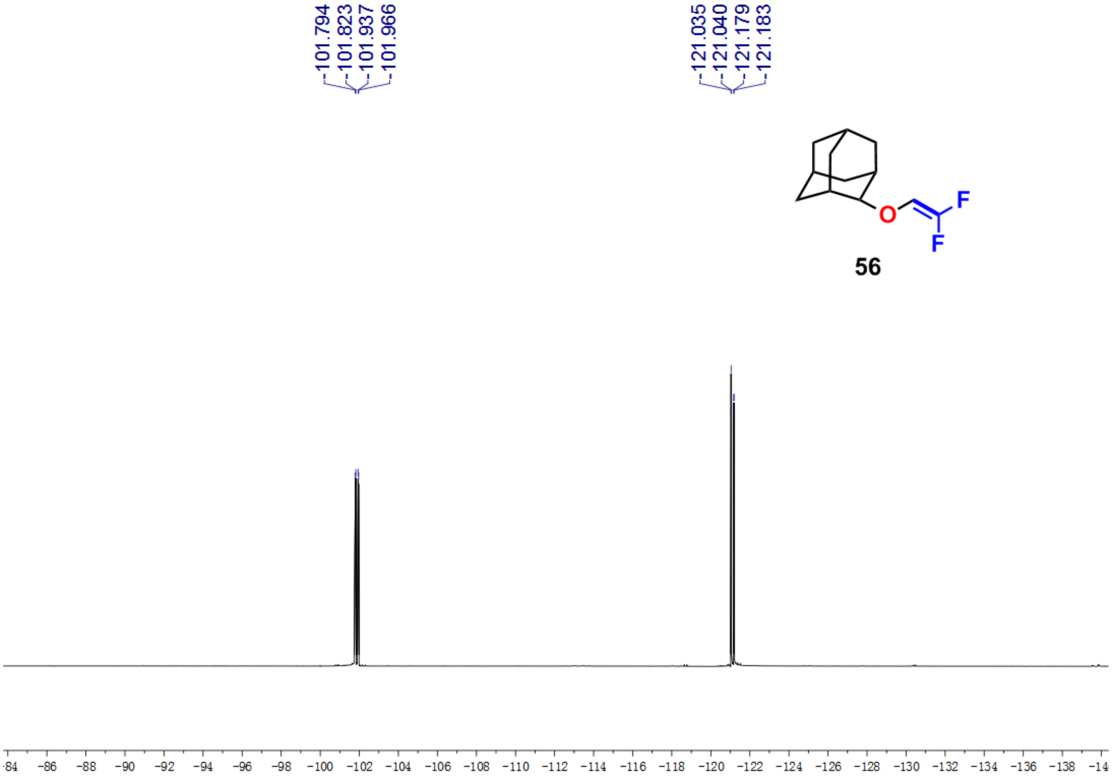

Supplementary Figure 162. ^{19}F NMR of 56

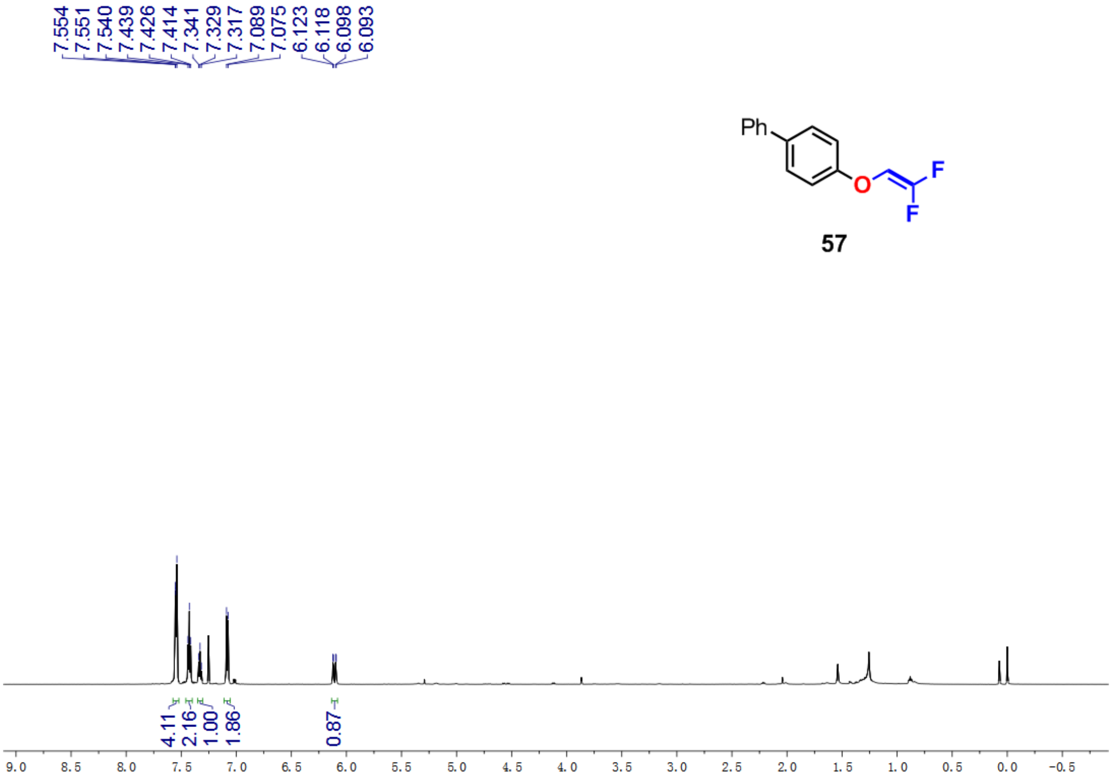

Supplementary Figure 163. ^{1}H NMR of 57

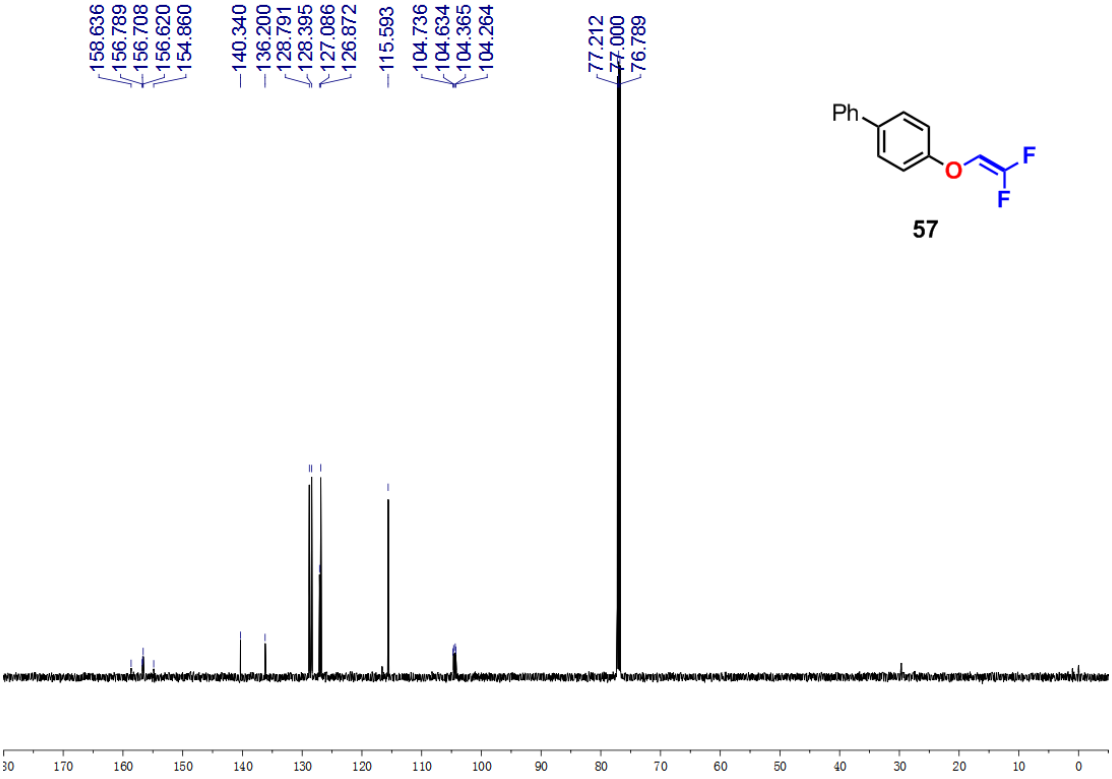

Supplementary Figure 164. ^{13}C NMR of 57

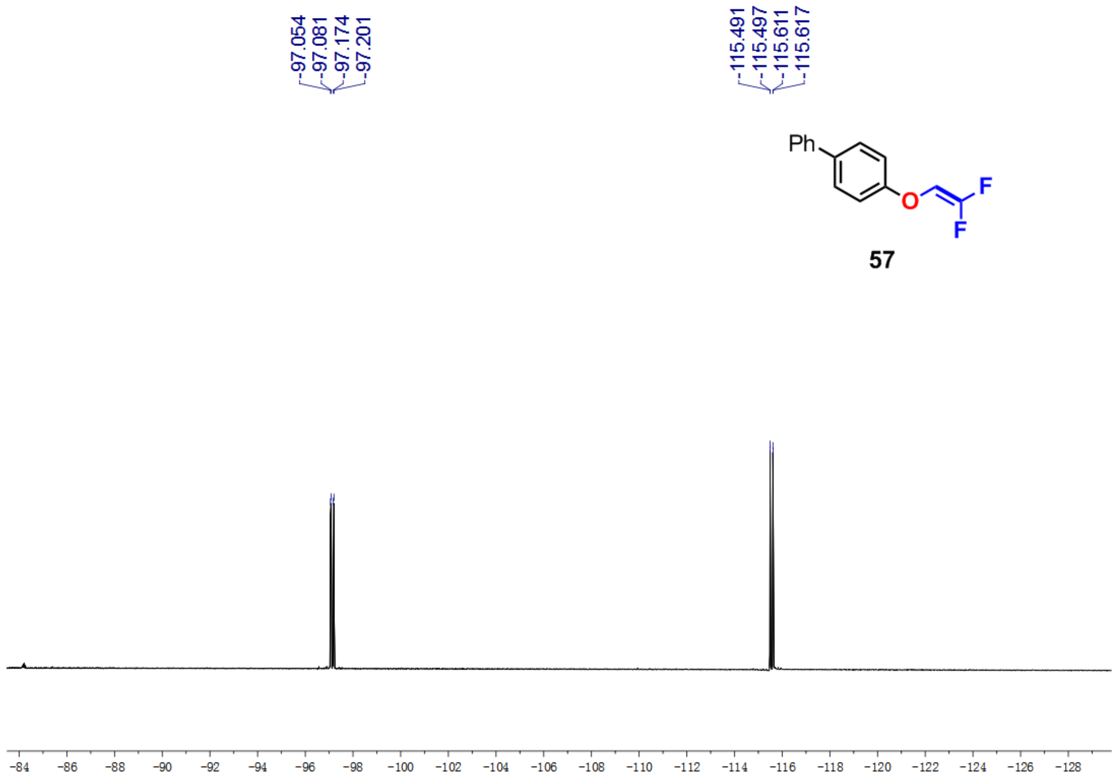

Supplementary Figure 165. ^{19}F NMR of 57

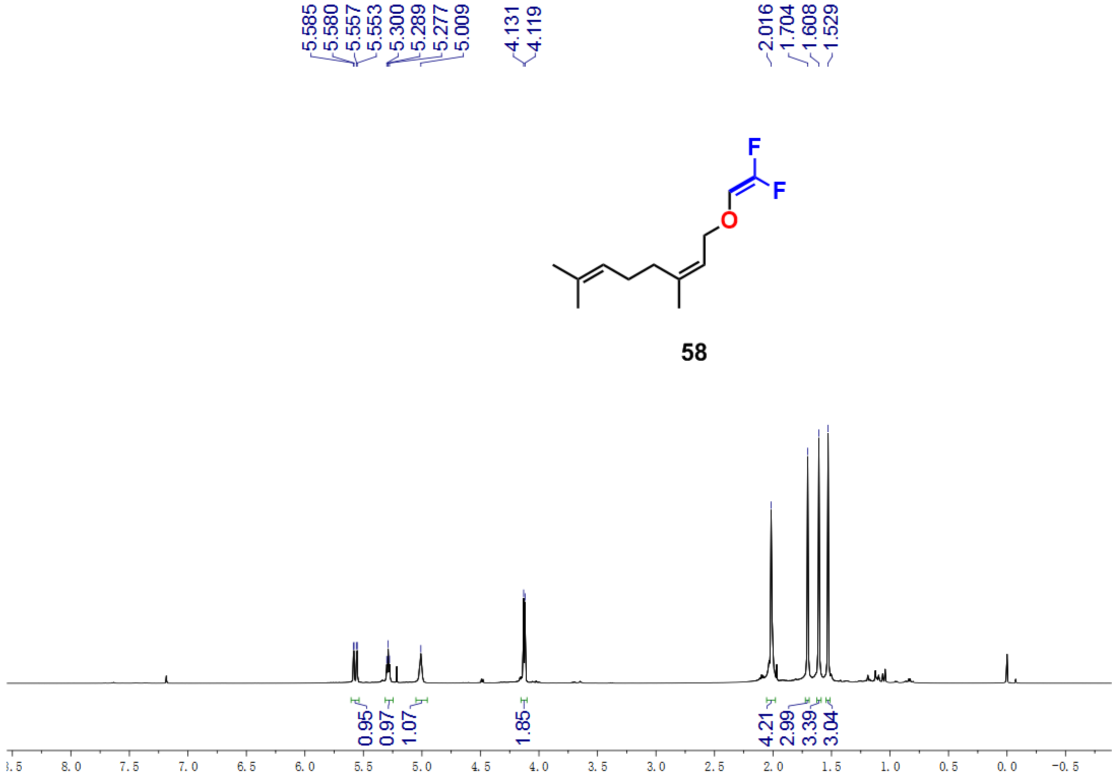

Supplementary Figure 166. ^{1}H NMR of 58

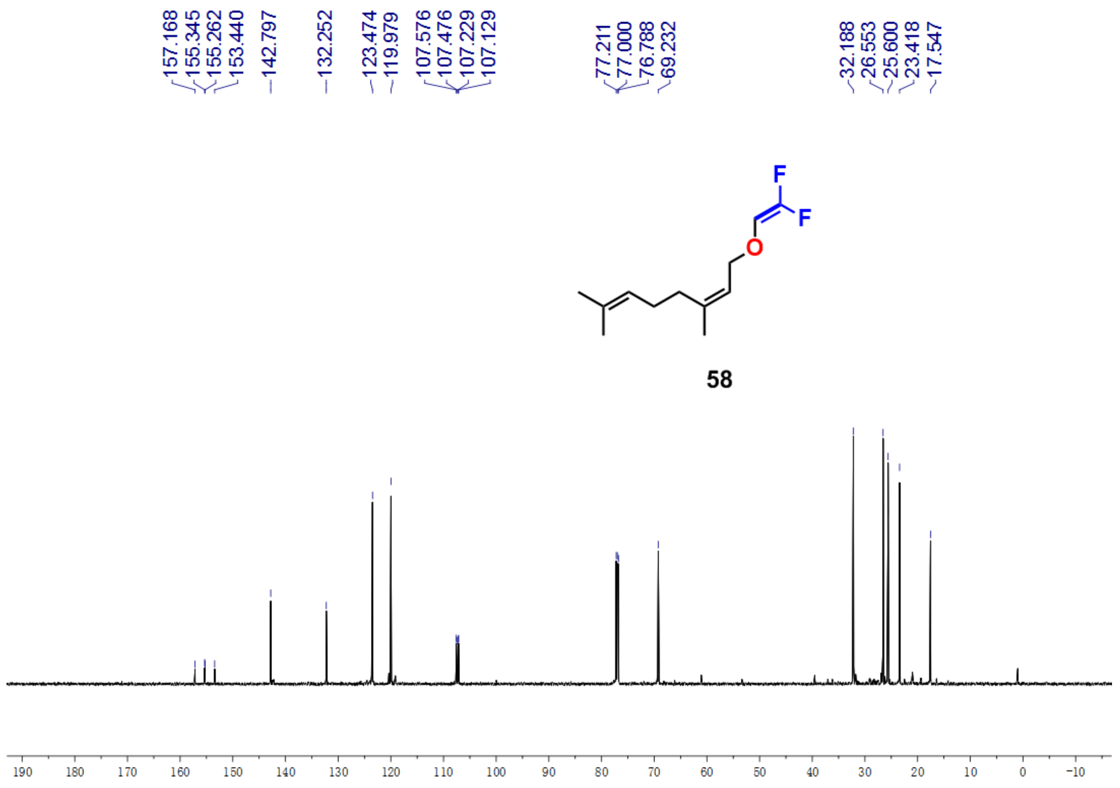

Supplementary Figure 167. ^{13}C NMR of 58

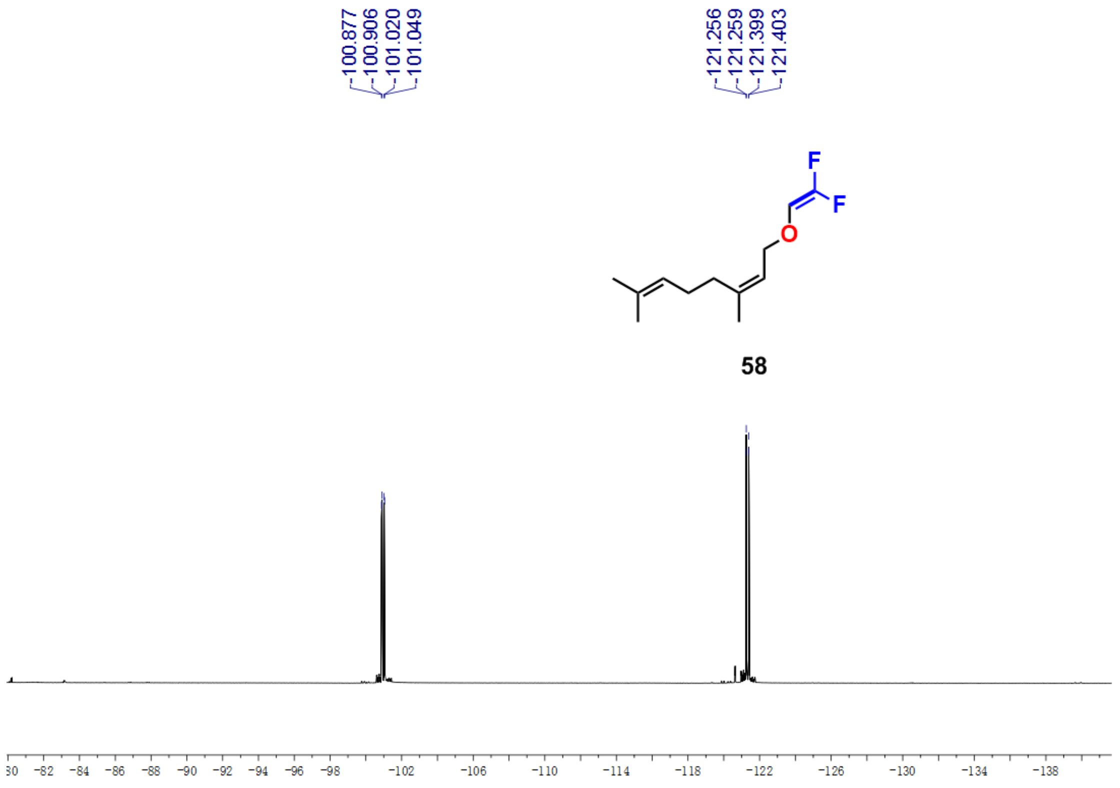

Supplementary Figure 168. ^{19}F NMR of 58

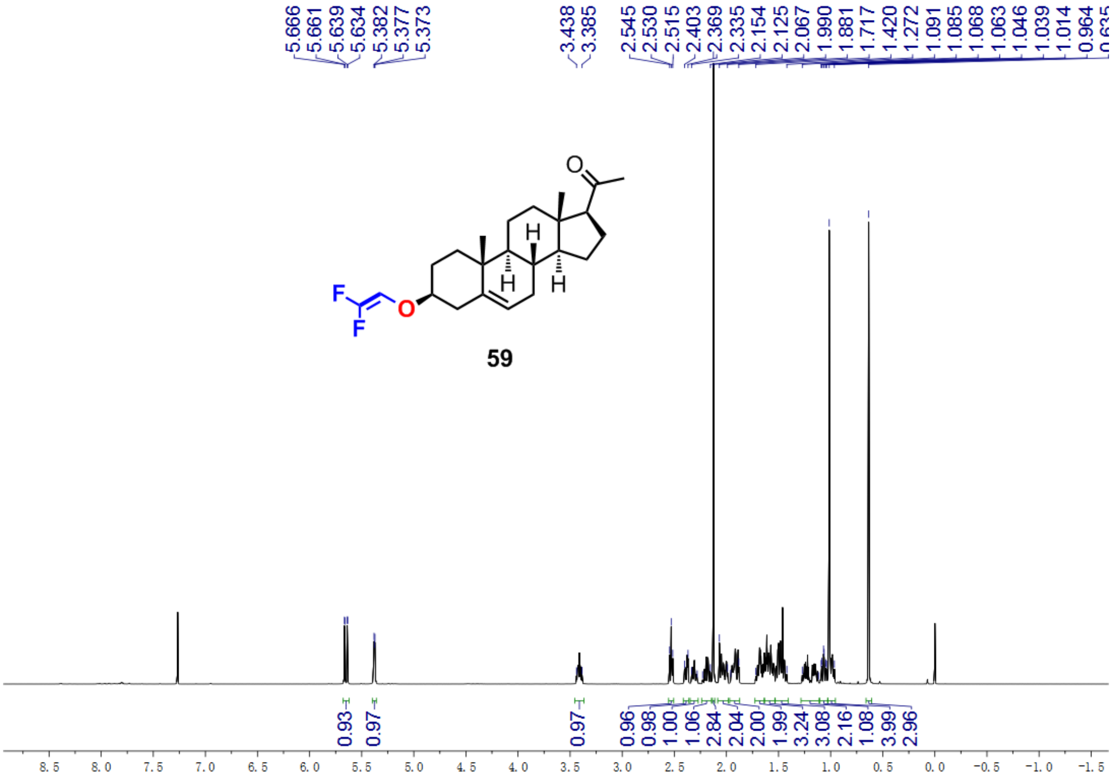

Supplementary Figure 169. ^{1}H NMR of 59

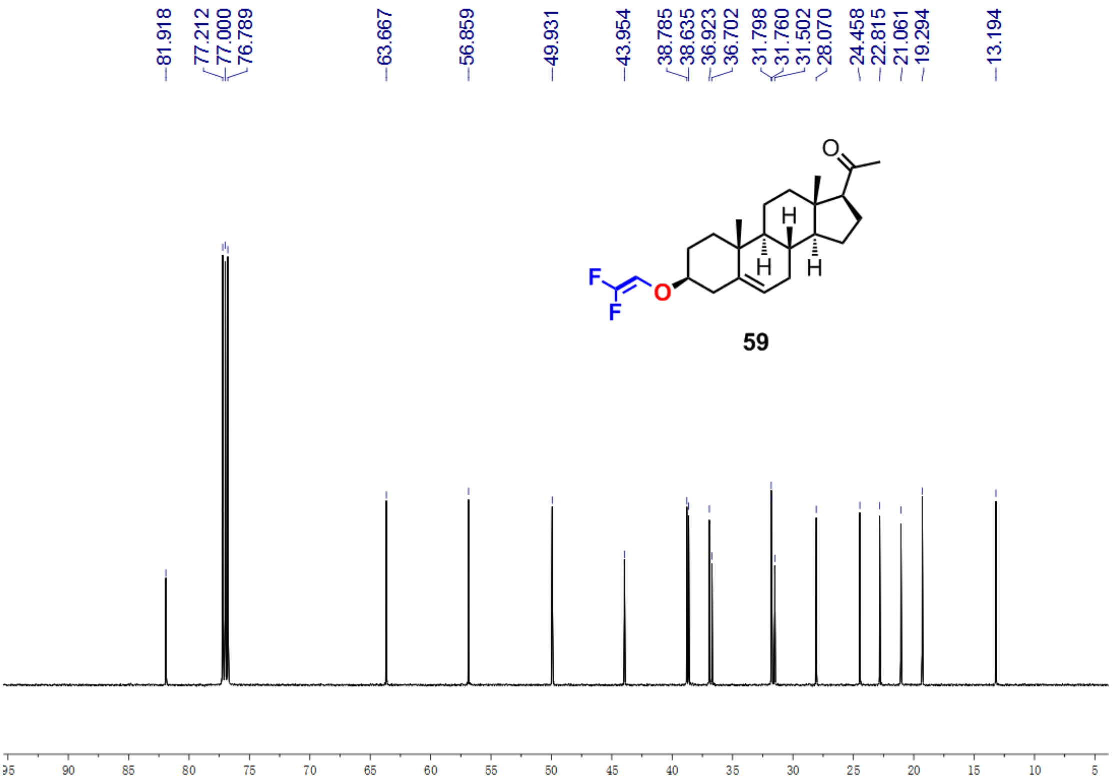

Supplementary Figure 170. ^{13}C NMR of 59

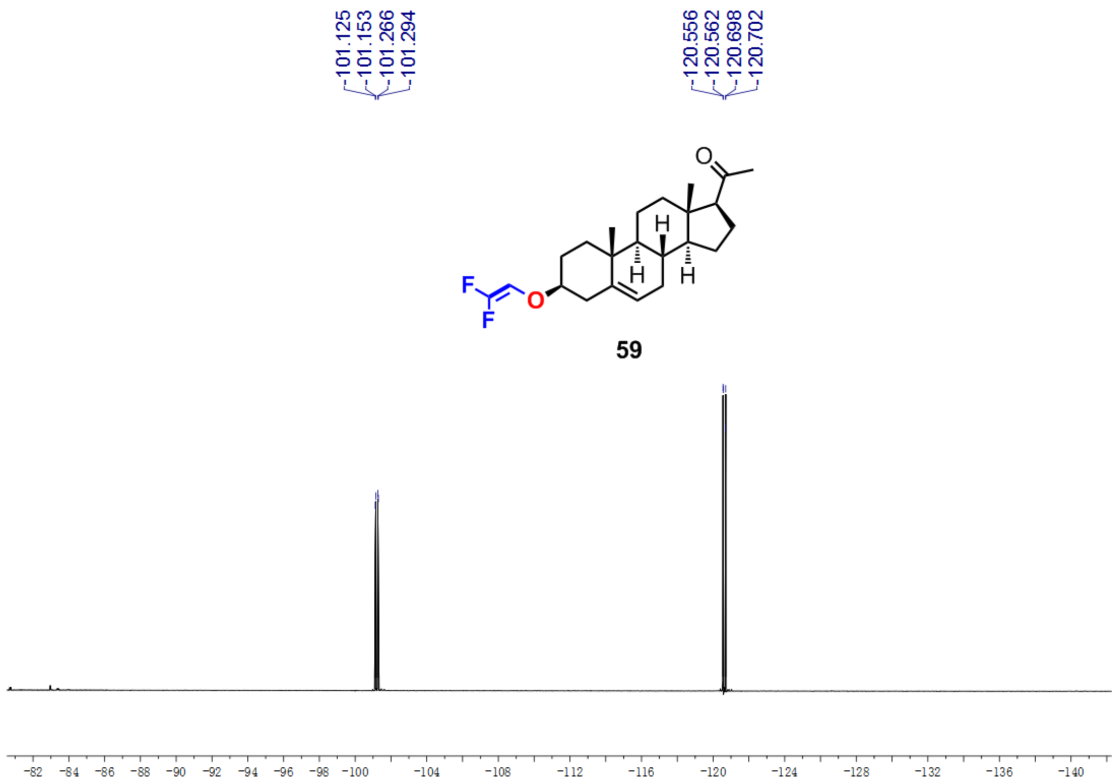

Supplementary Figure 171. ^{19}F NMR of 59

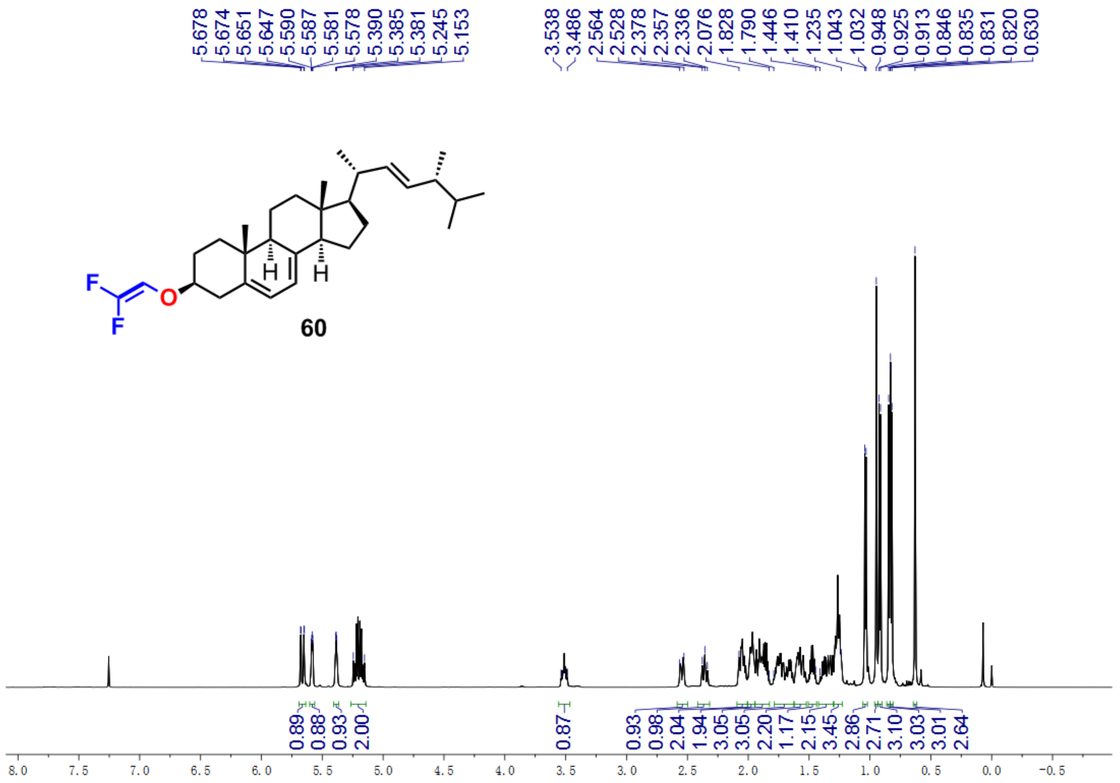

Supplementary Figure 172. ^{1}H NMR of 60

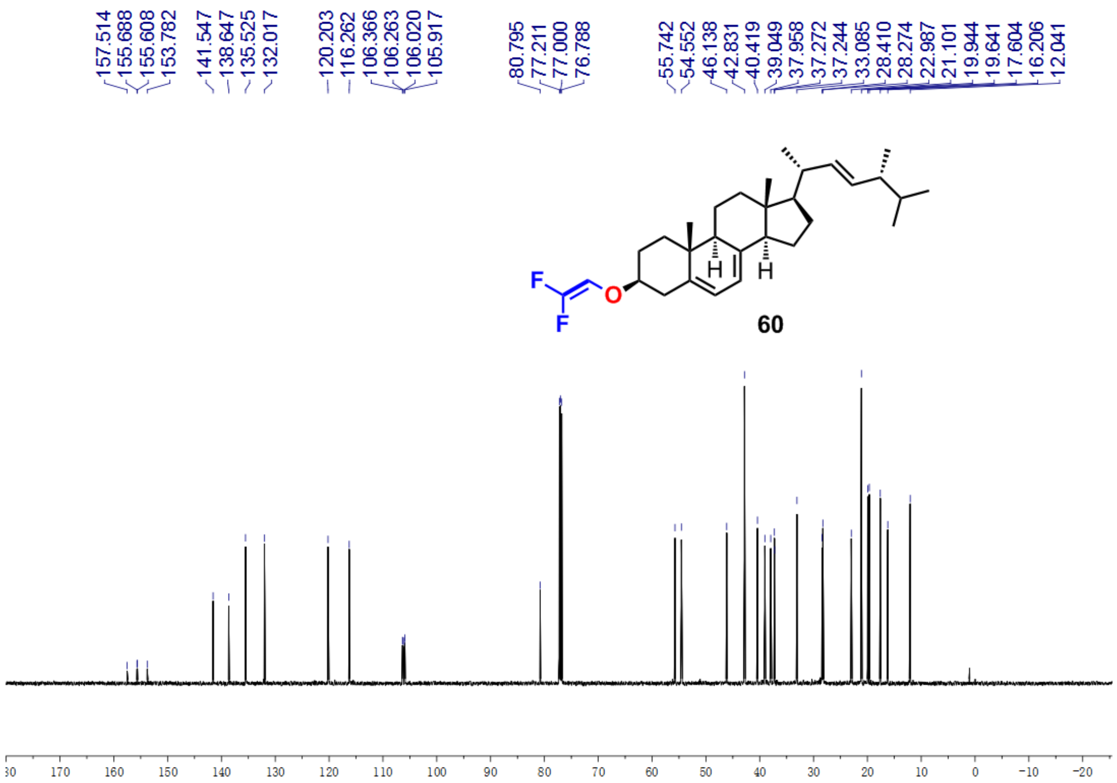

Supplementary Figure 173. ^{13}C NMR of 60

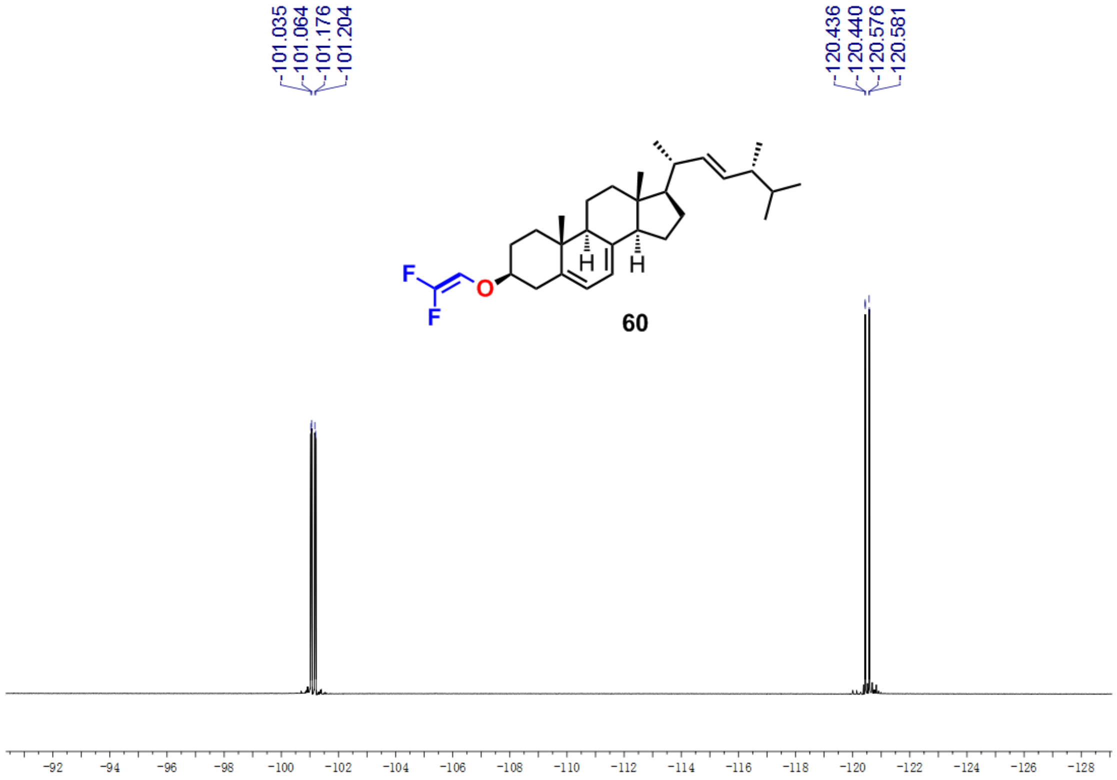

Supplementary Figure 174. ^{19}F NMR of 60

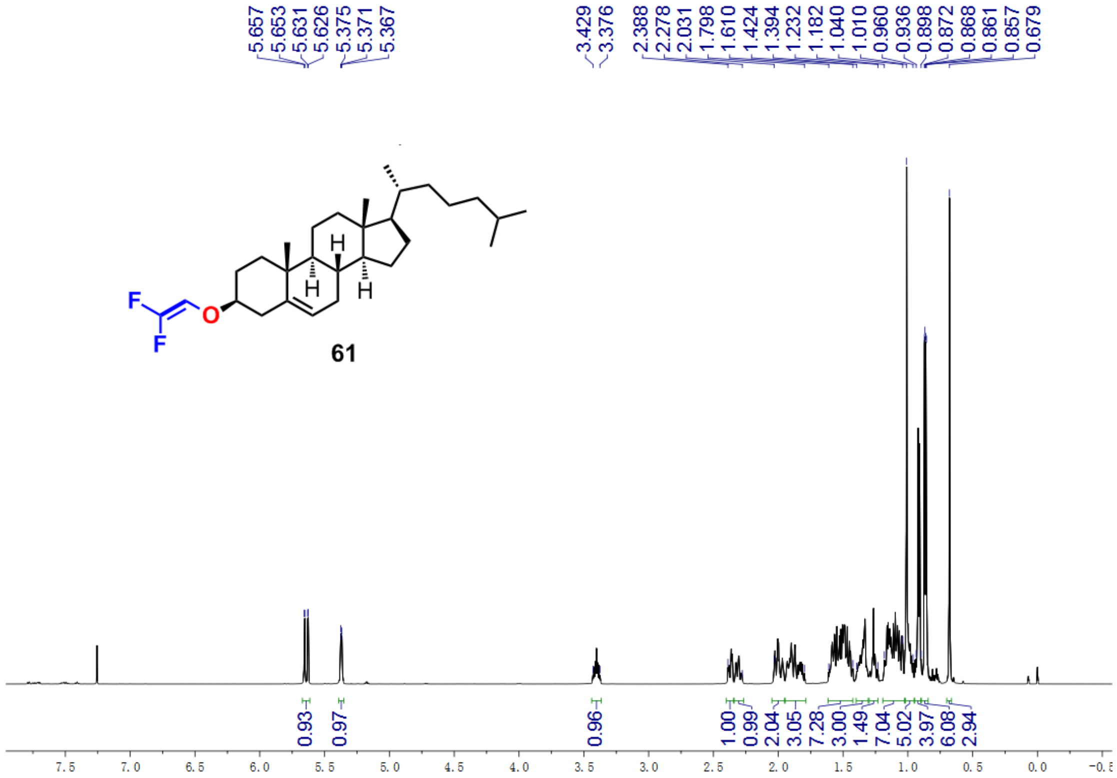

Supplementary Figure 175. ^{1}H NMR of 61

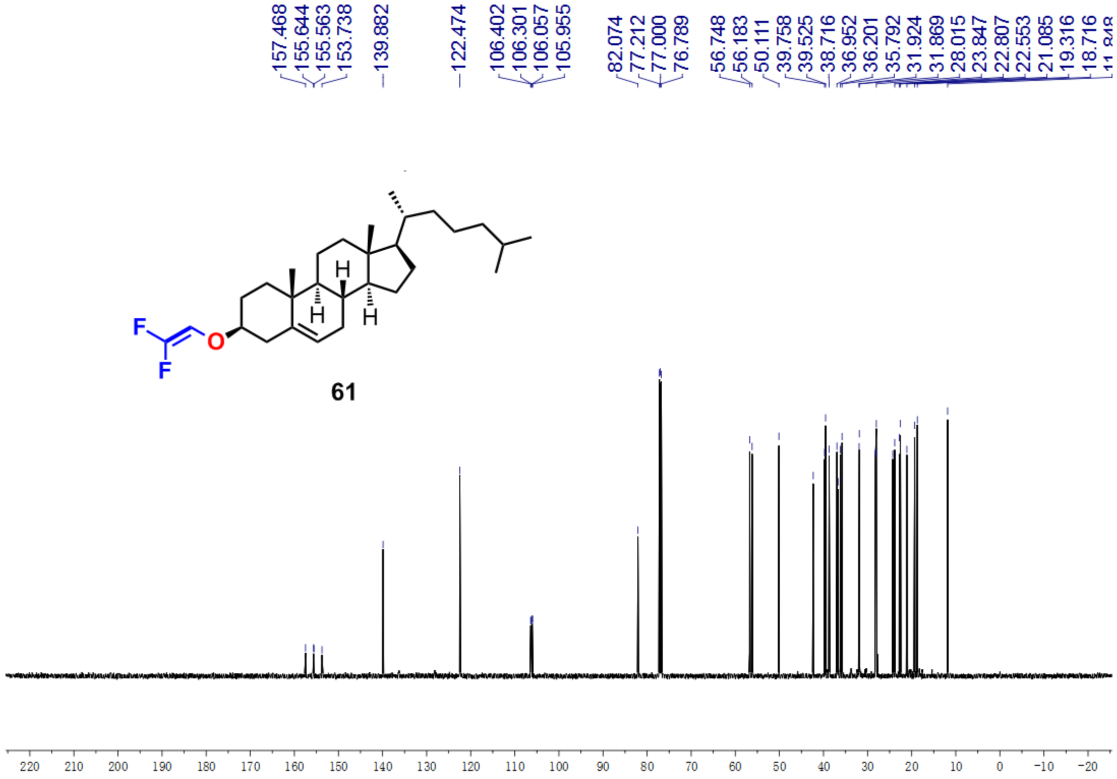

Supplementary Figure 176. ^{13}C NMR of 61

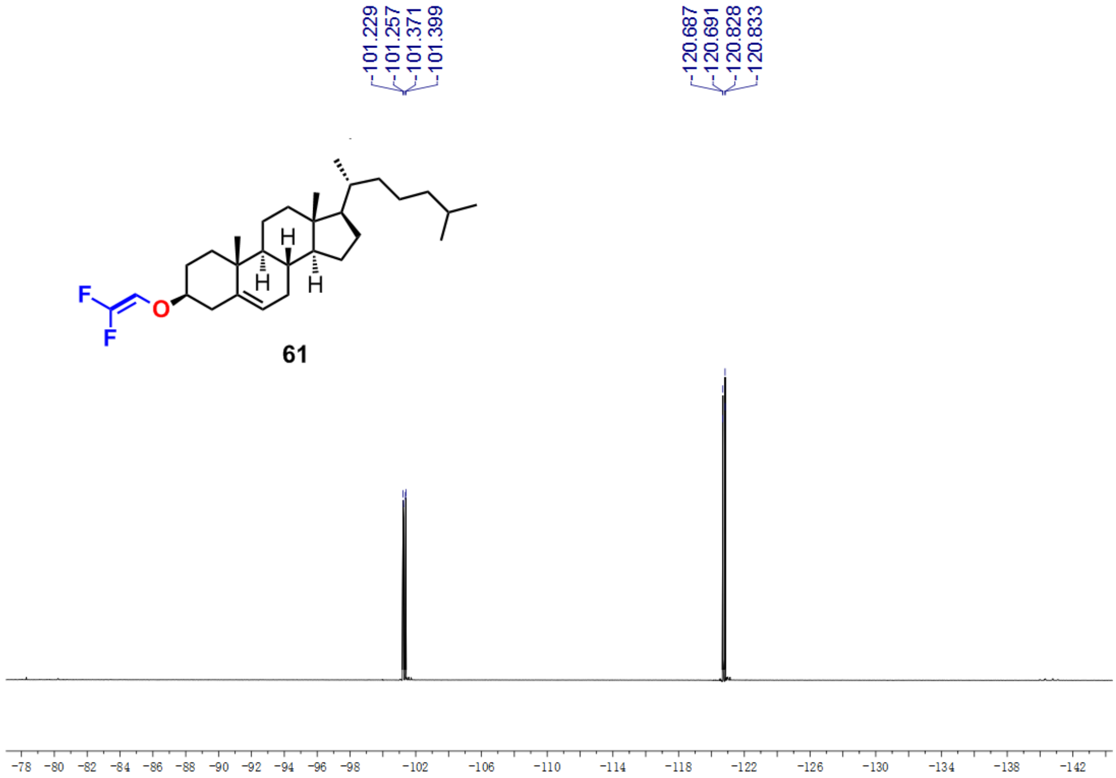

Supplementary Figure 177. ^{19}F NMR of 61

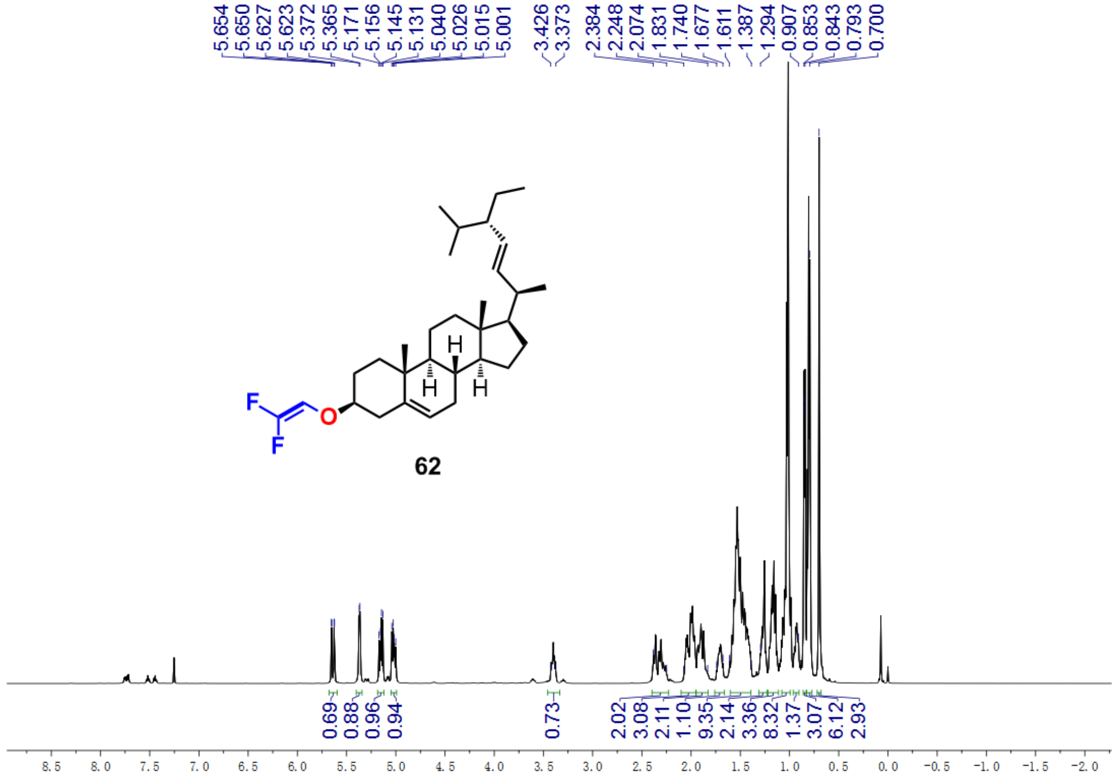

Supplementary Figure 178. ^{1}H NMR of 62

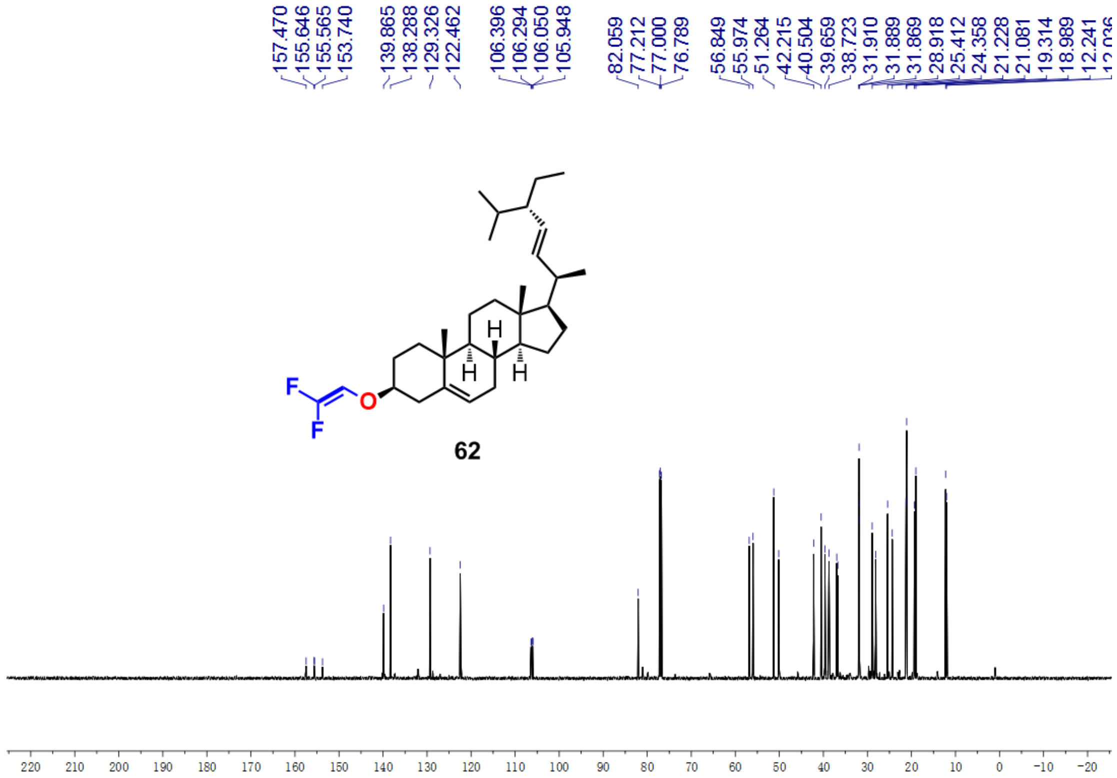

Supplementary Figure 179. ^{13}C NMR of 62

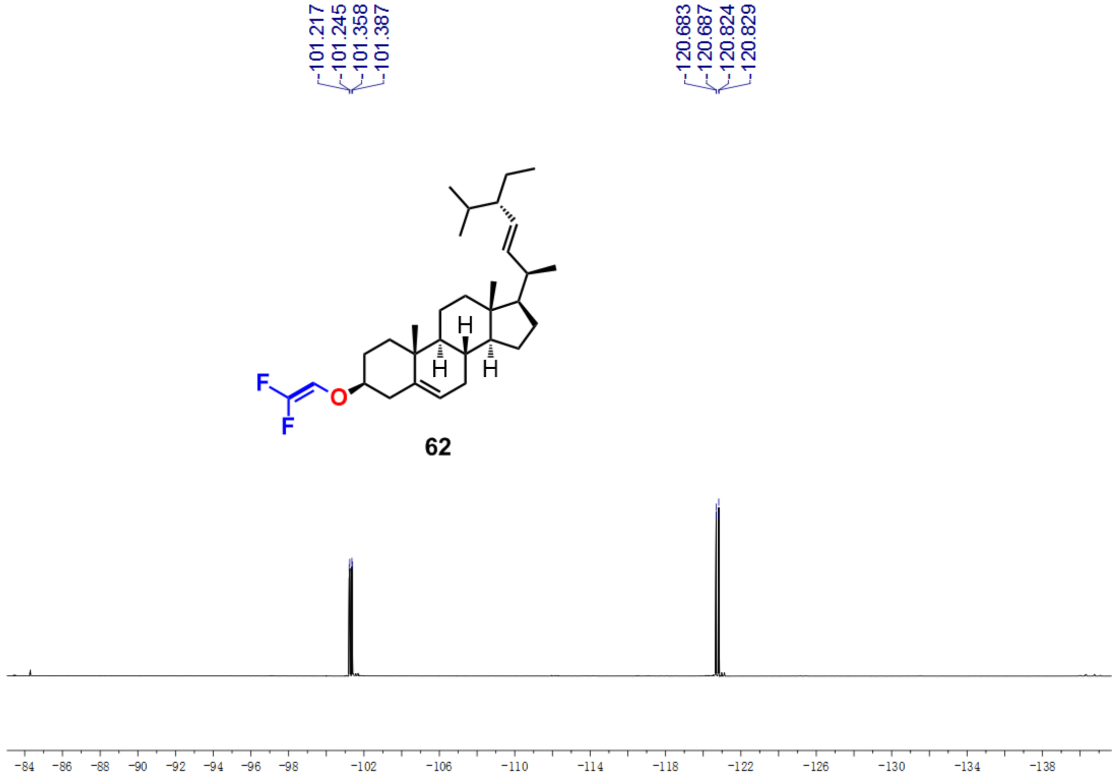

Supplementary Figure 180. ^{19}F NMR of 62

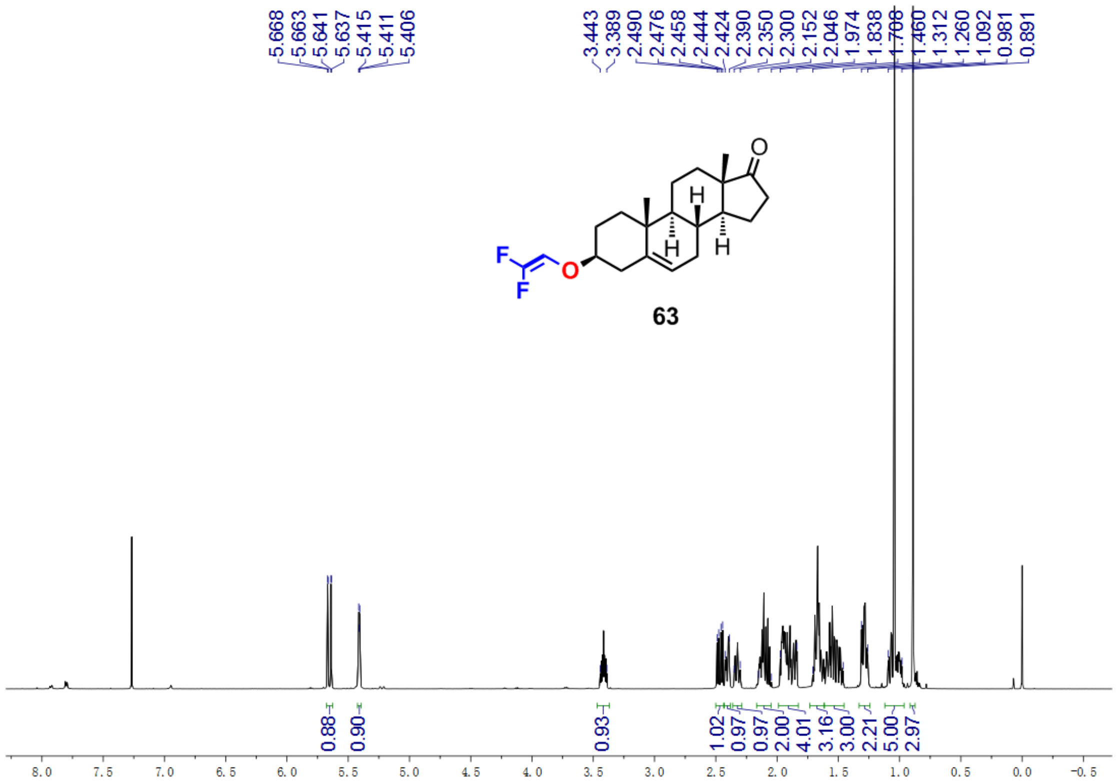

Supplementary Figure 181. ^{1}H NMR of 63

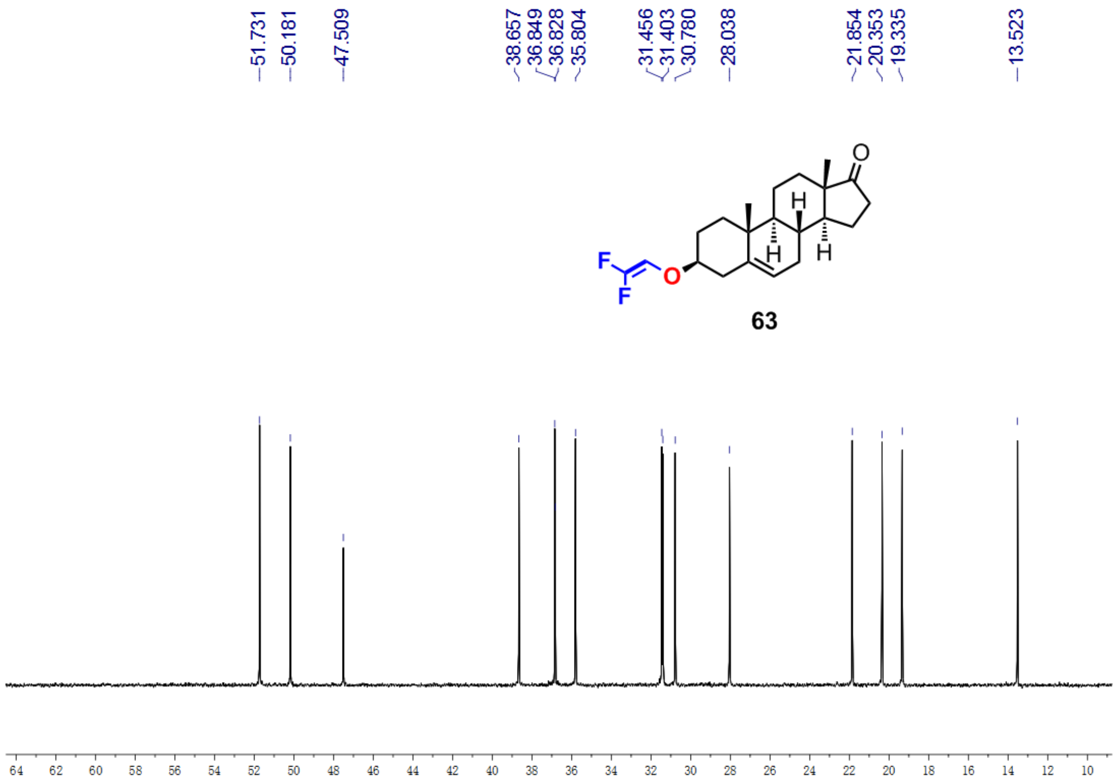

Supplementary Figure 182. ^{13}C NMR of 63

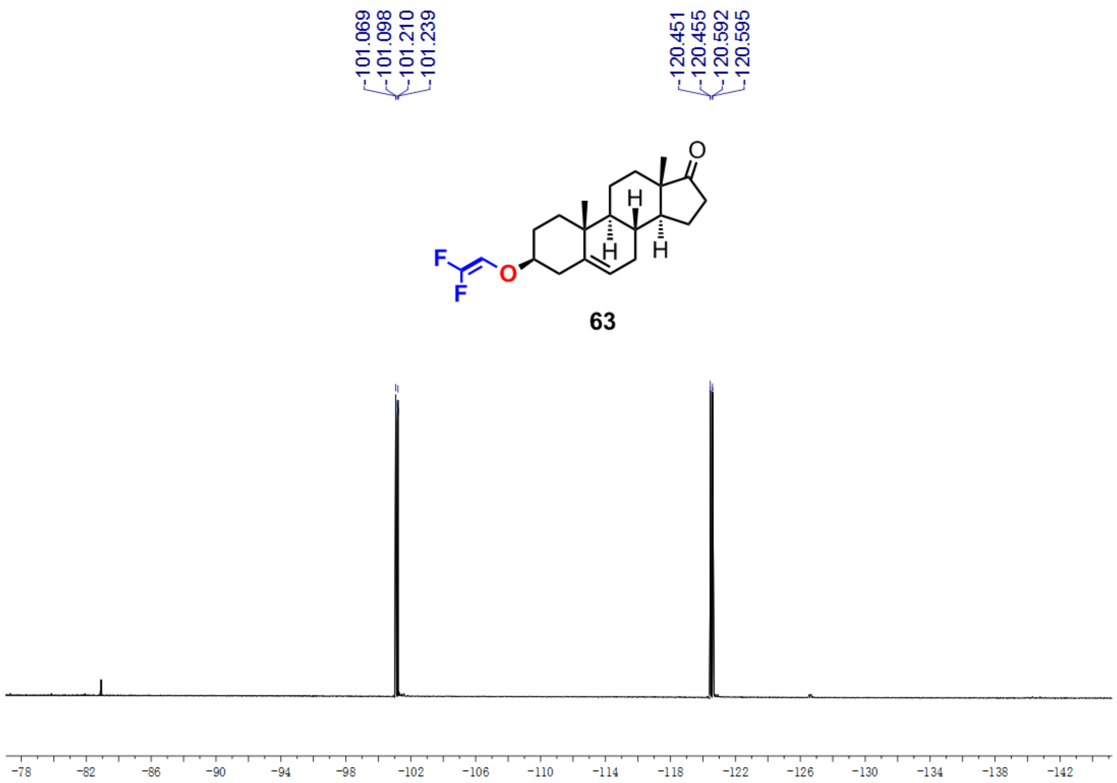

Supplementary Figure 183. ^{19}F NMR of 63

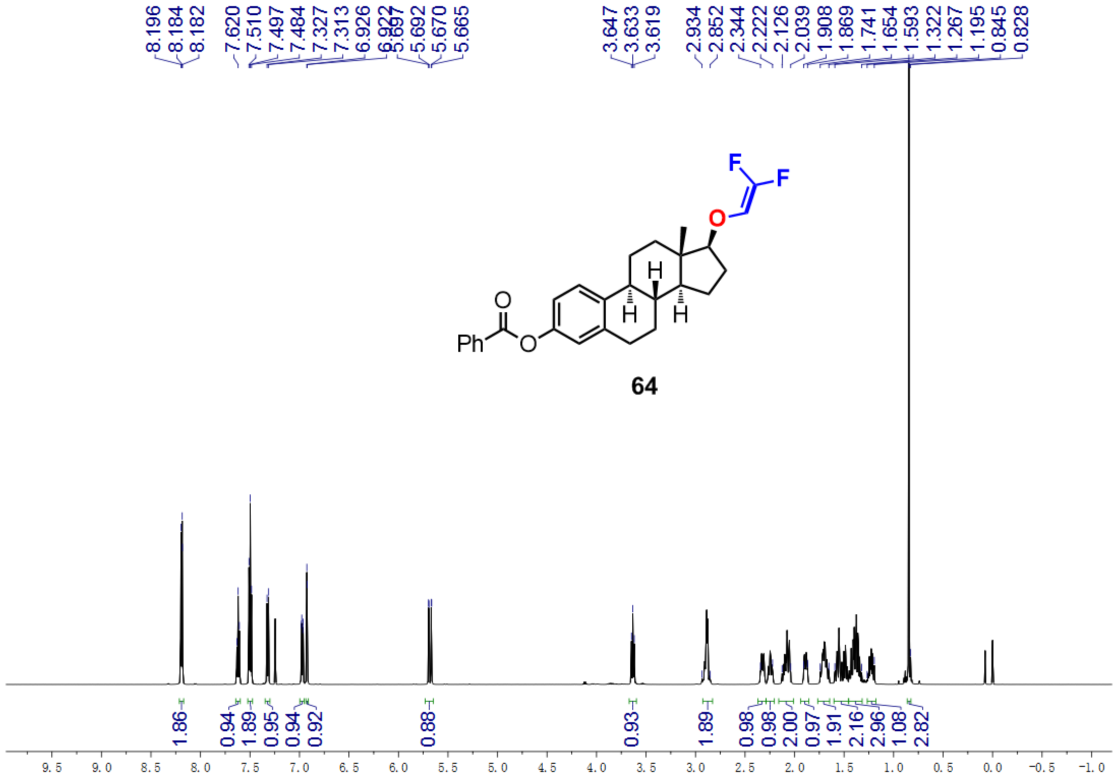

Supplementary Figure 184. ^{1}H NMR of 64

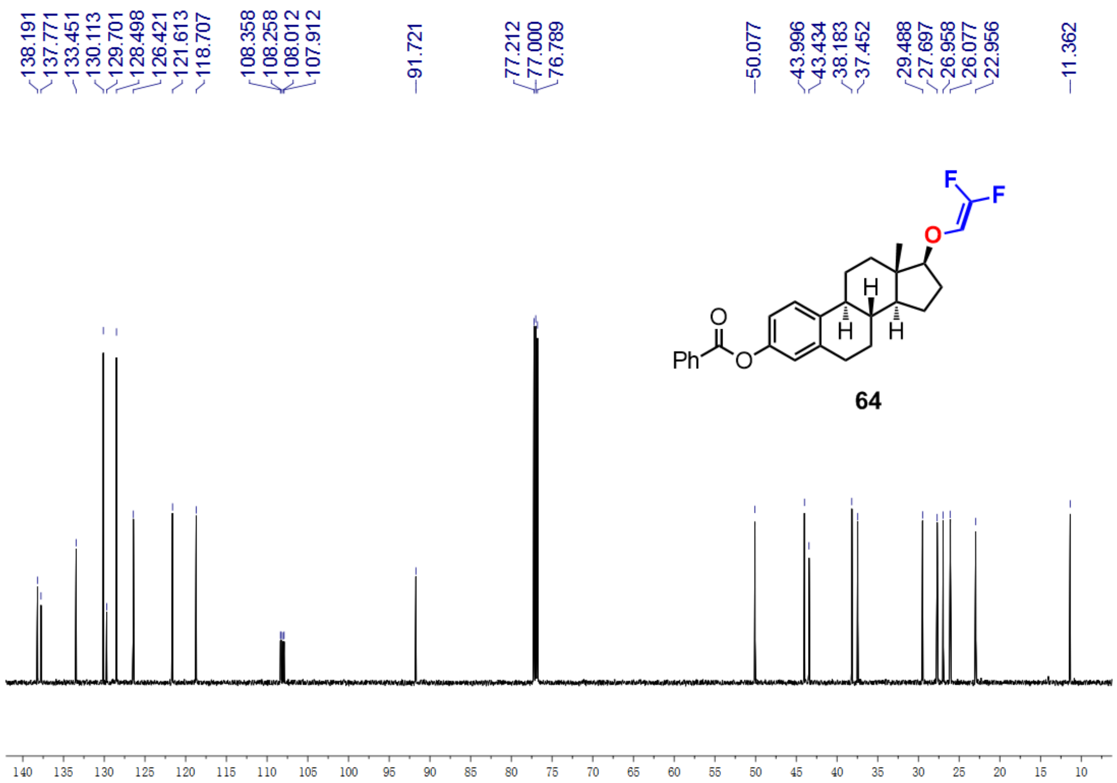

Supplementary Figure 185. ^{13}C NMR of 64

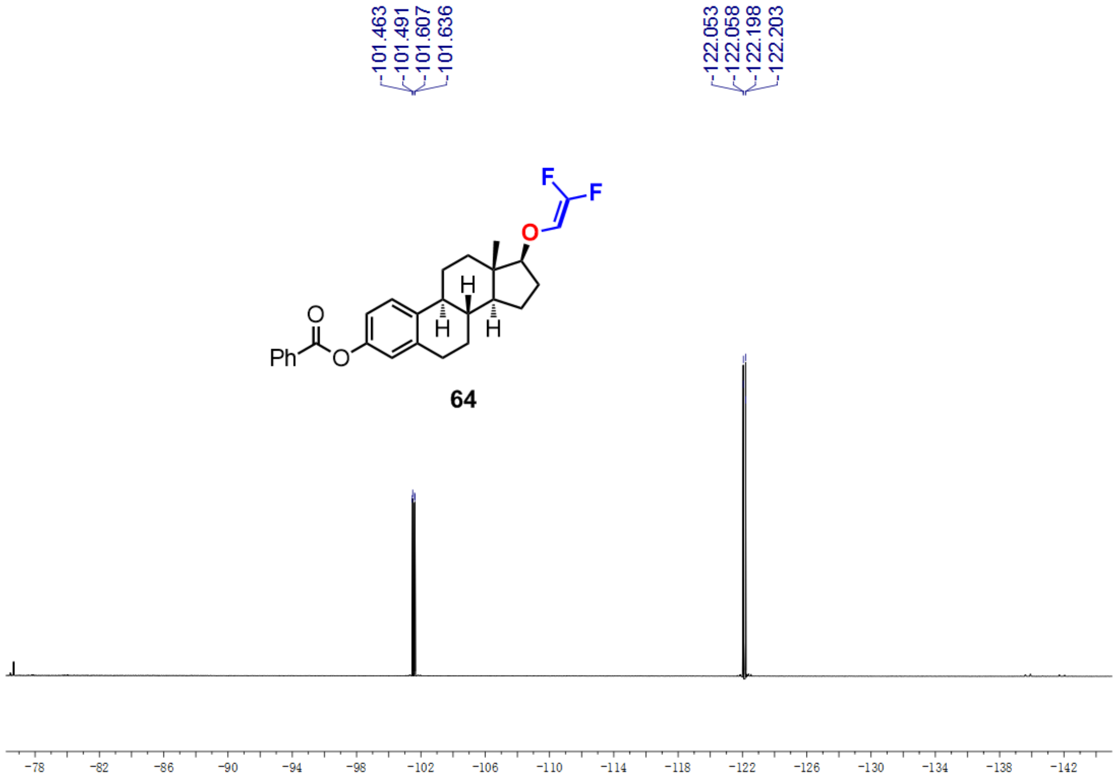

Supplementary Figure 186. ^{19}F NMR of 64

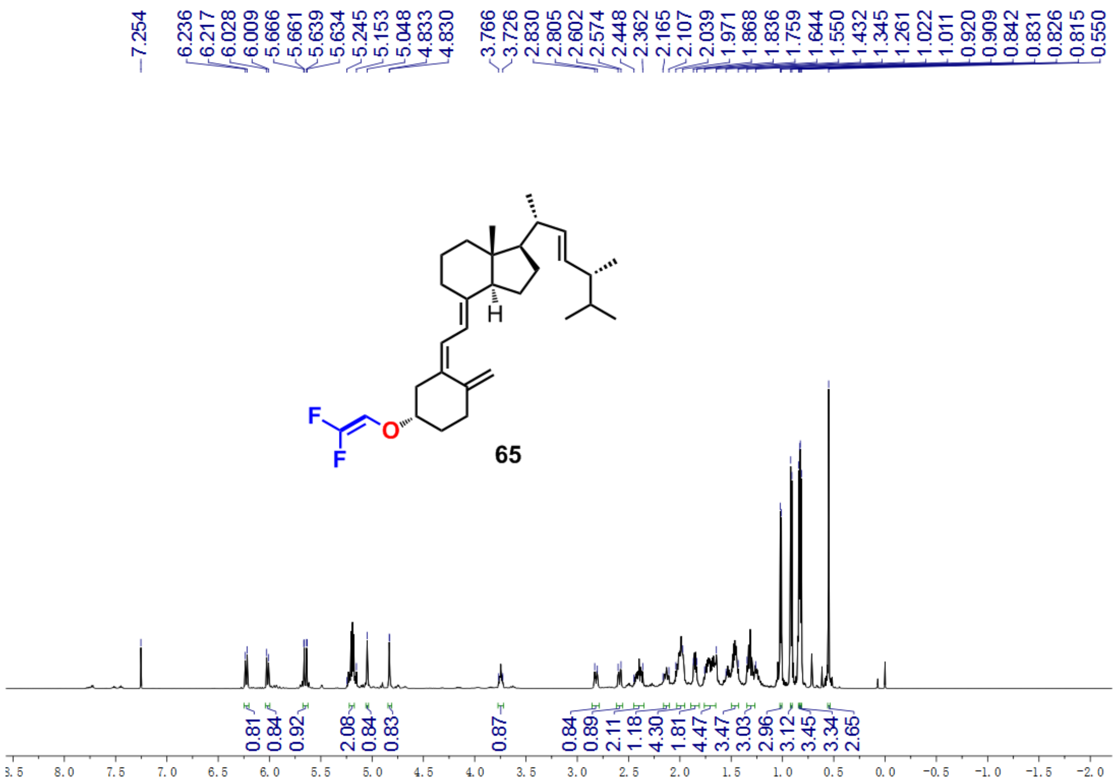

Supplementary Figure 187. ^{1}H NMR of 65

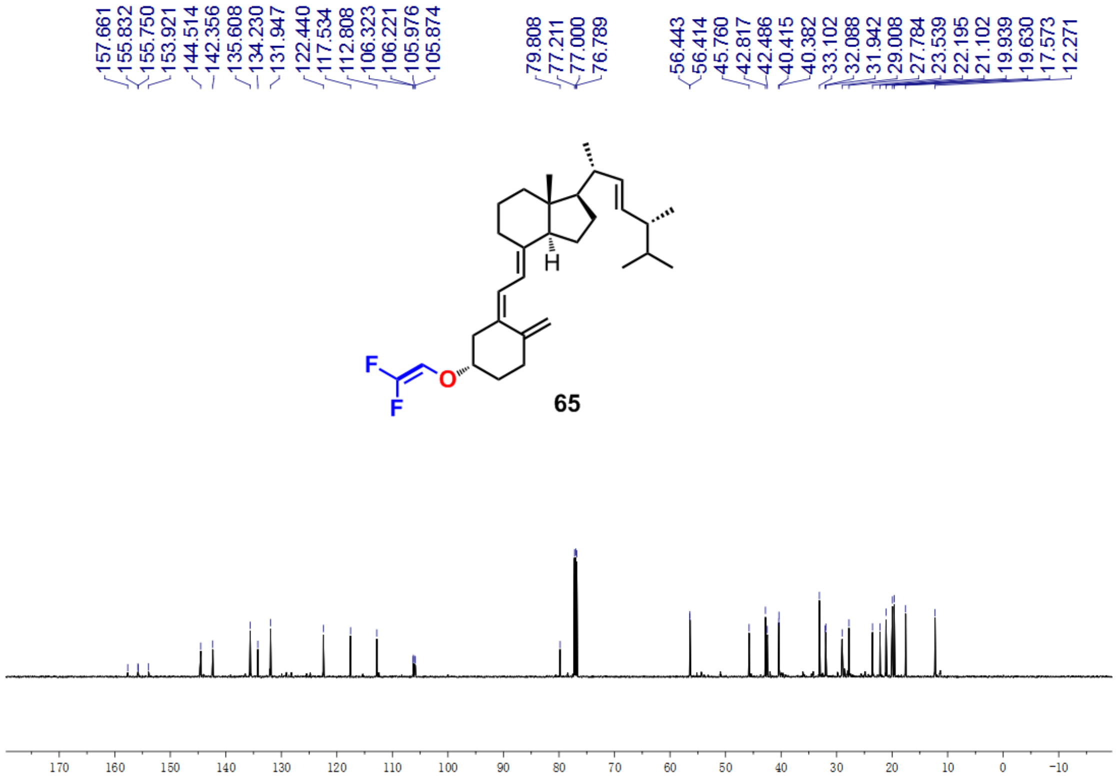

Supplementary Figure 188. ^{13}C NMR of 65

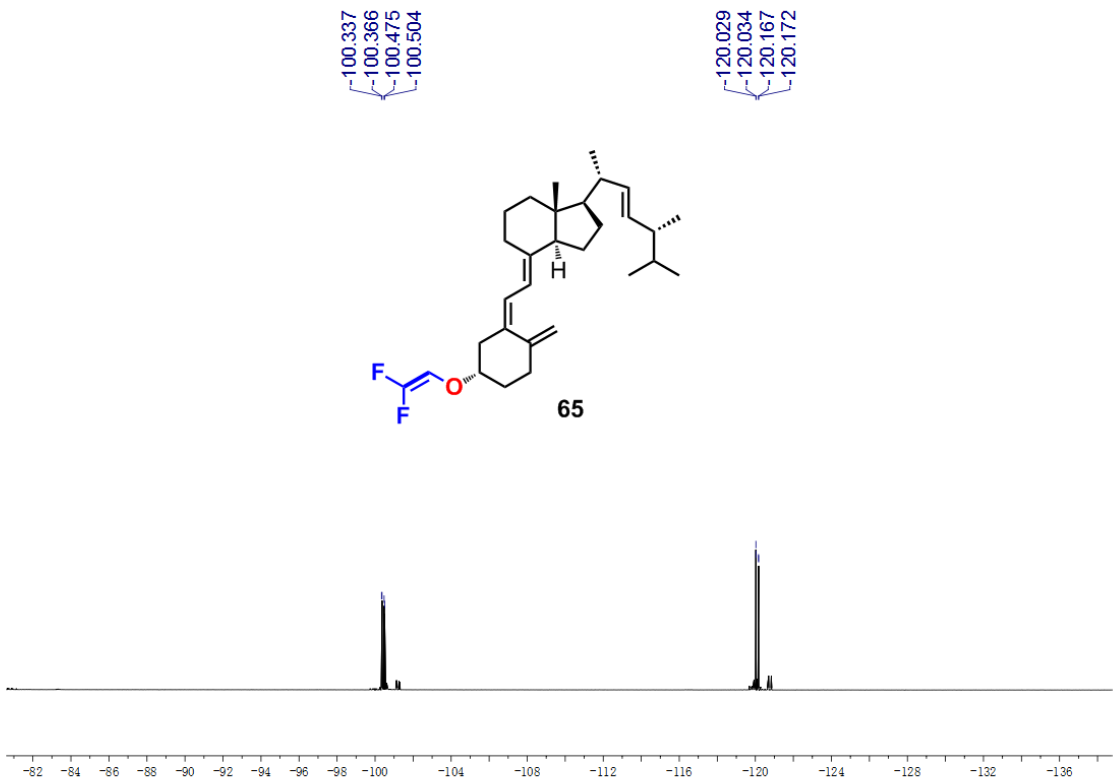

Supplementary Figure 189. ^{19}F NMR of 65

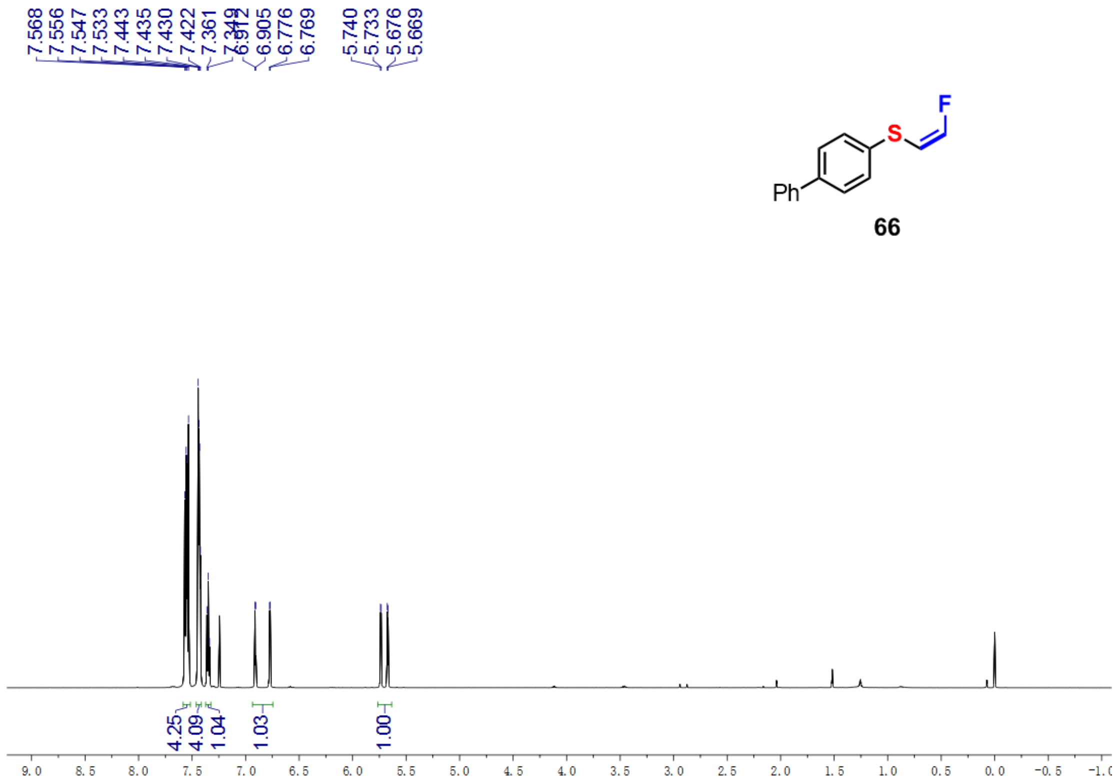

Supplementary Figure 190. ^{1}H NMR of 66

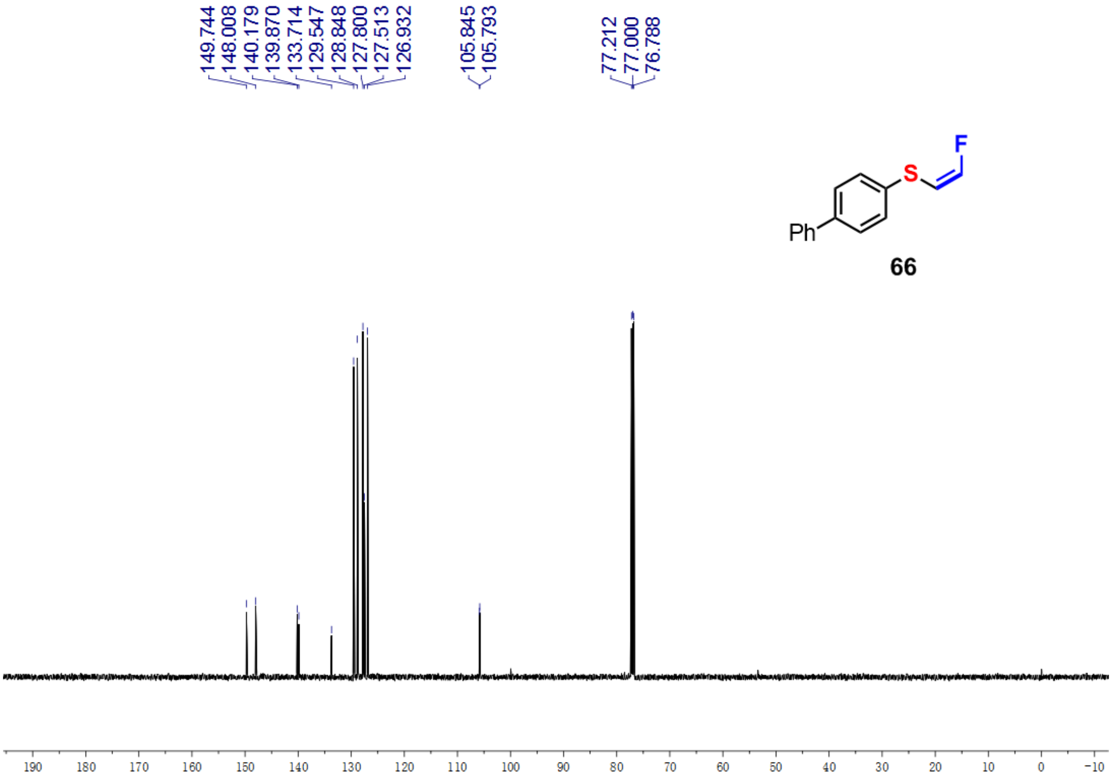

Supplementary Figure 191. ^{13}C NMR of 66

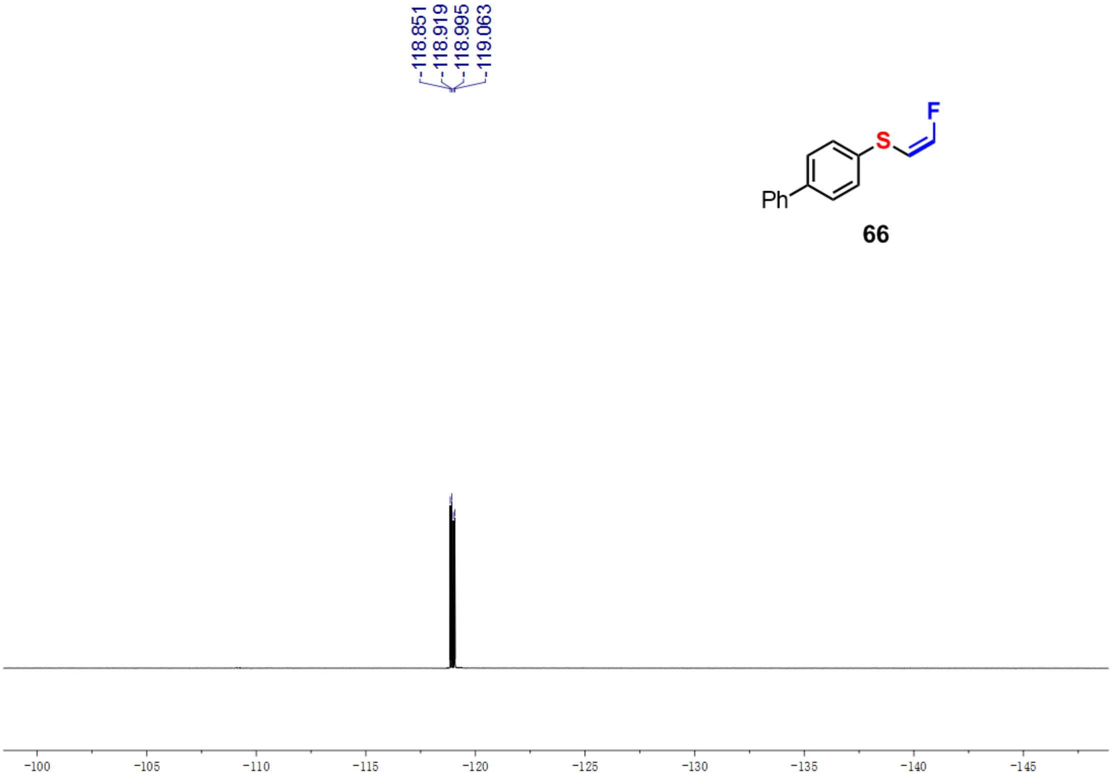

Supplementary Figure 192. ^{19}F NMR of 66

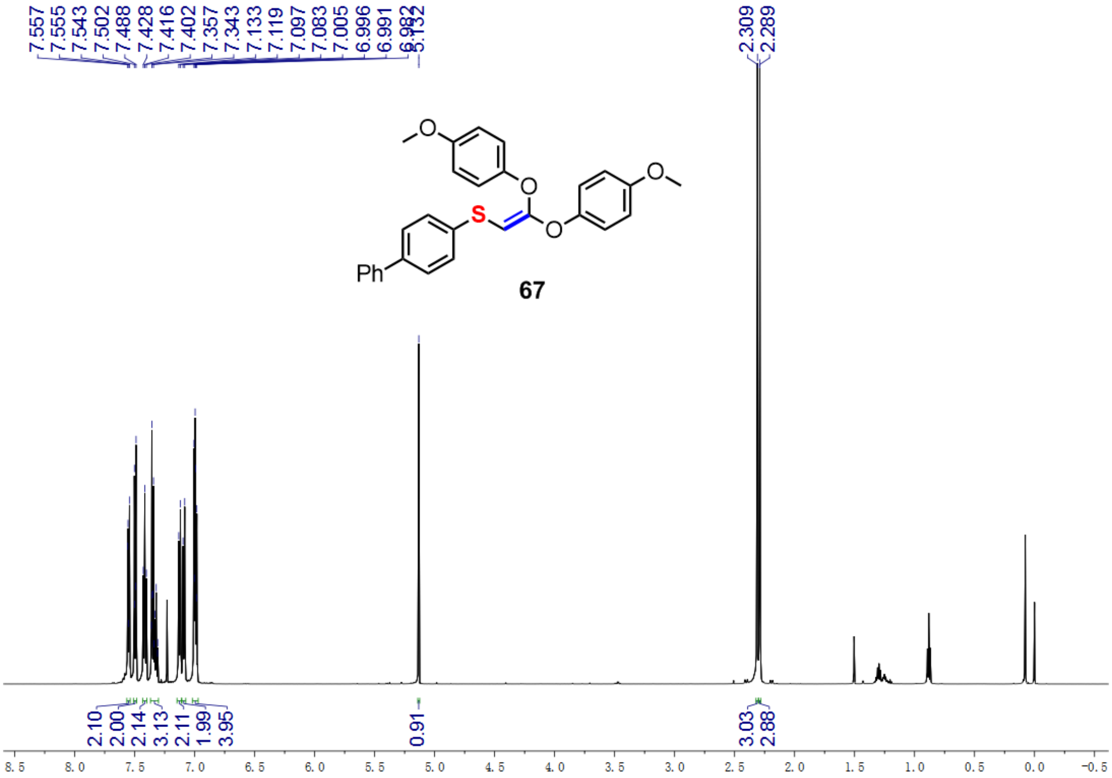

Supplementary Figure 193. ^{1}H NMR of 67

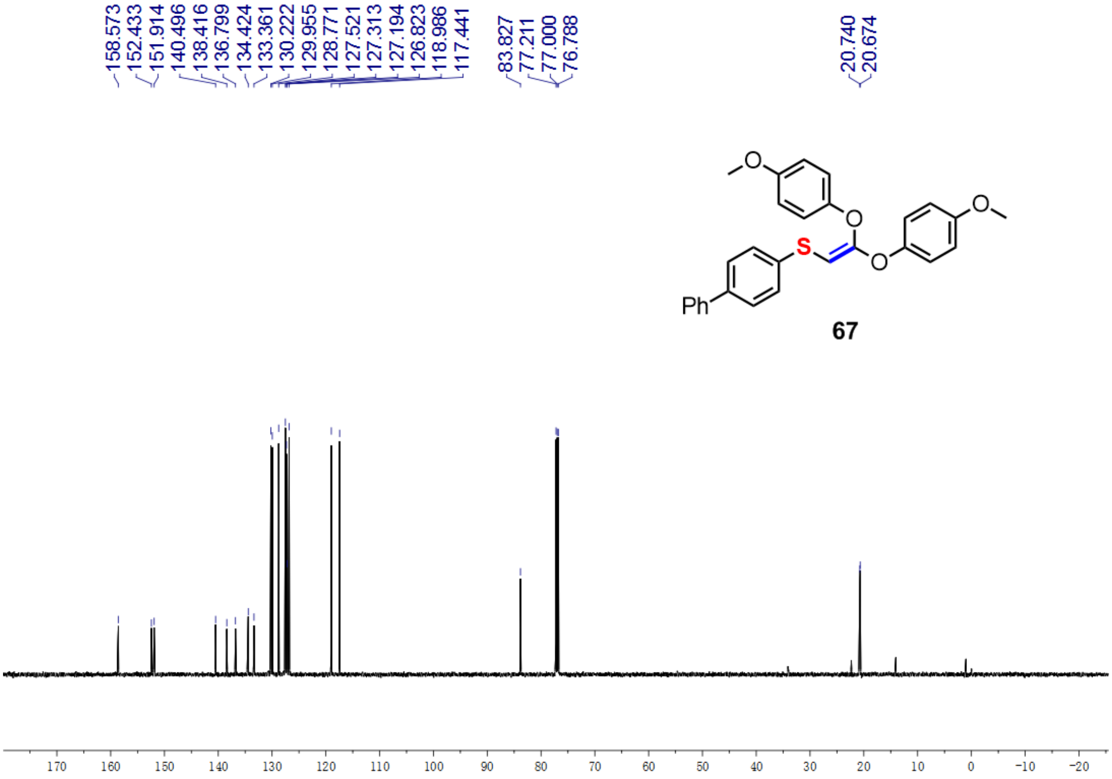

Supplementary Figure 194. ^{13}C NMR of 67

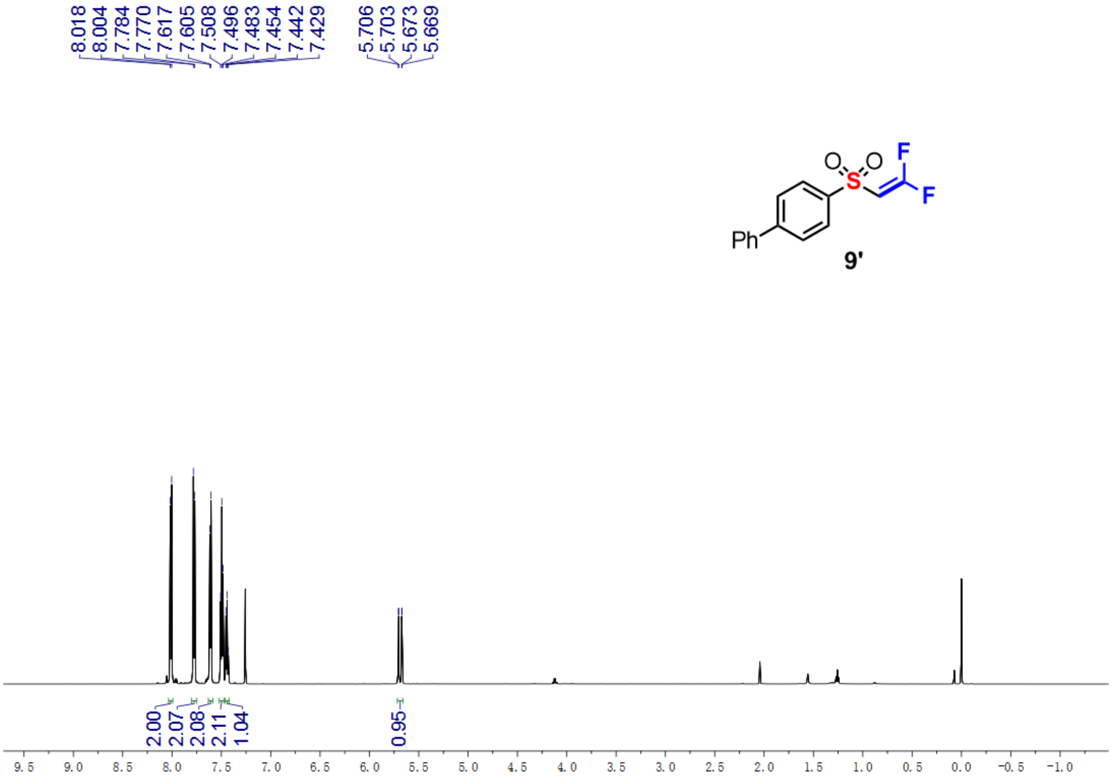

Supplementary Figure 195. ^{1}H NMR of 9’

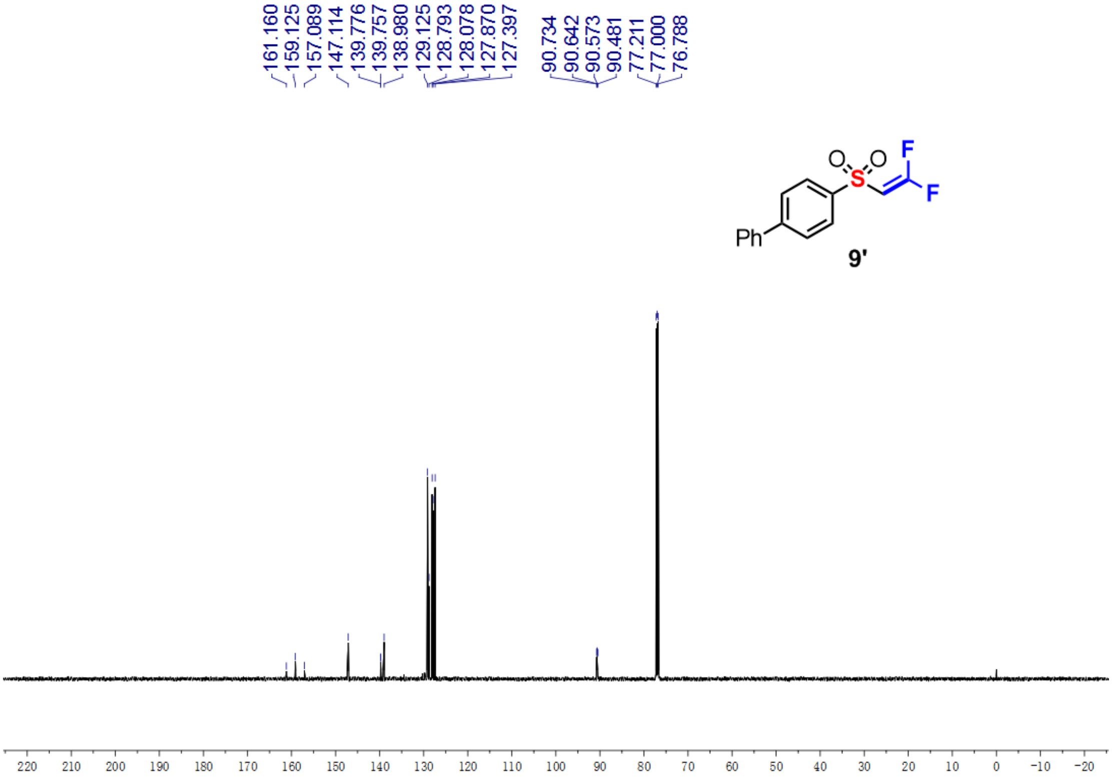

Supplementary Figure 196. ^{13}C NMR of 9’

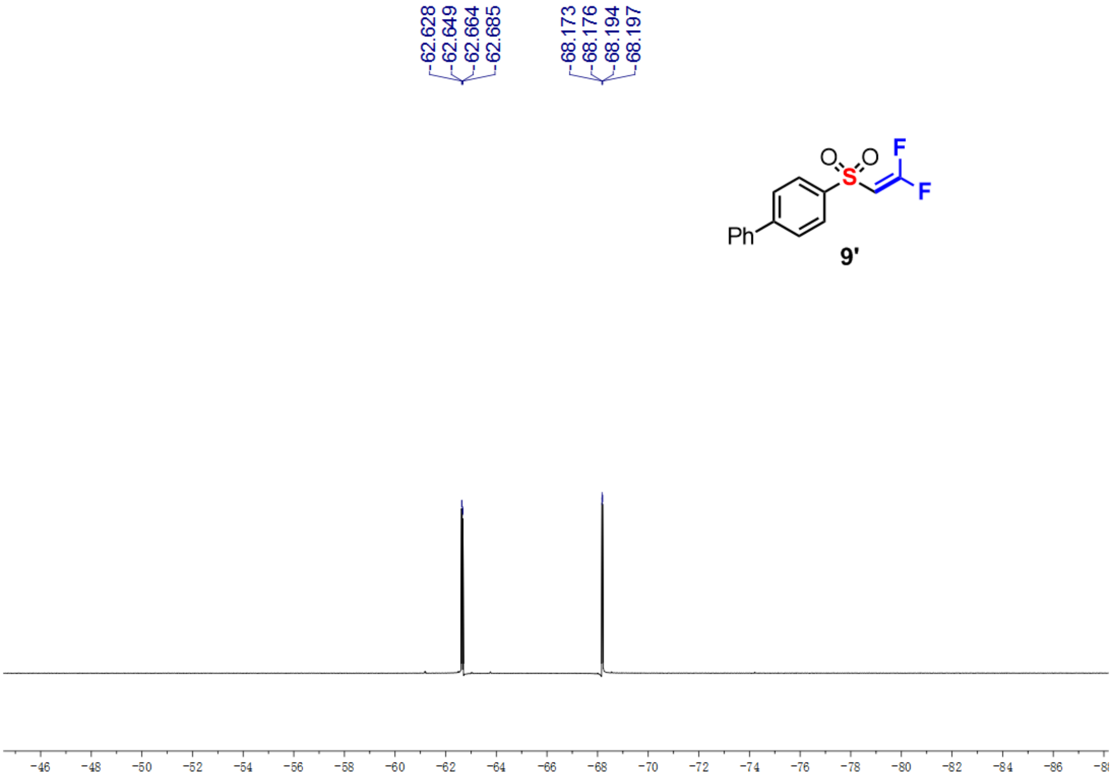

Supplementary Figure 197. ^{19}F NMR of 9’

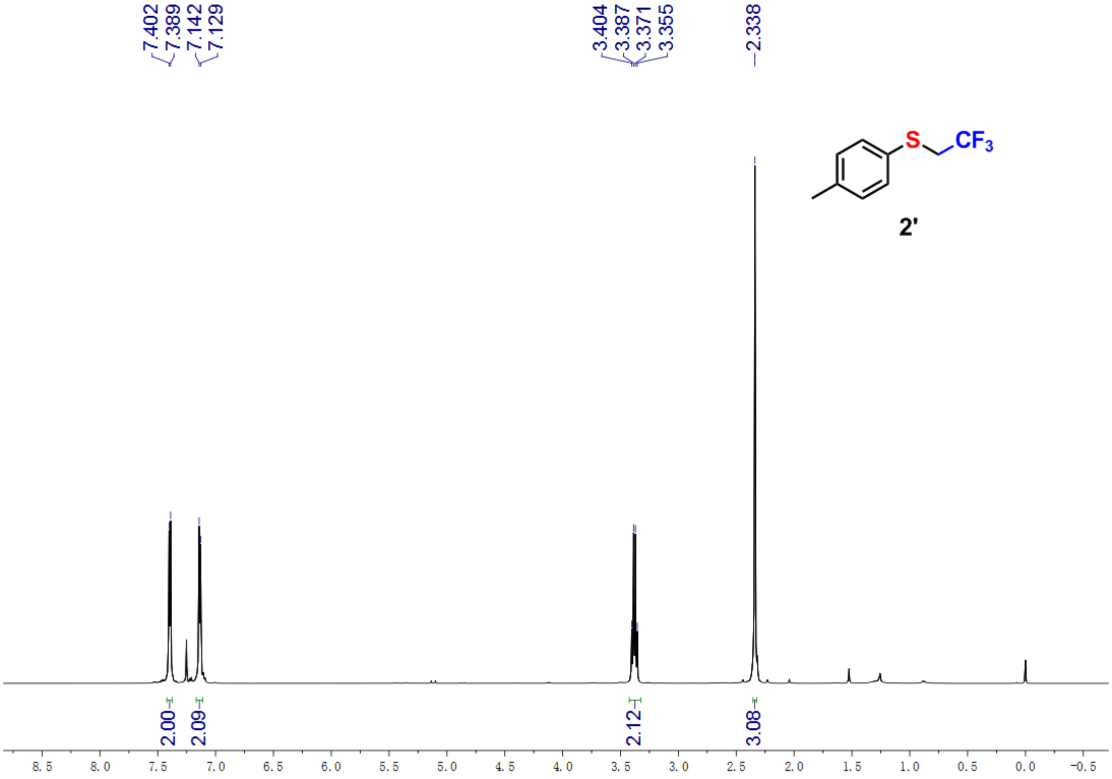

Supplementary Figure 198. ^{1}H NMR of 2’

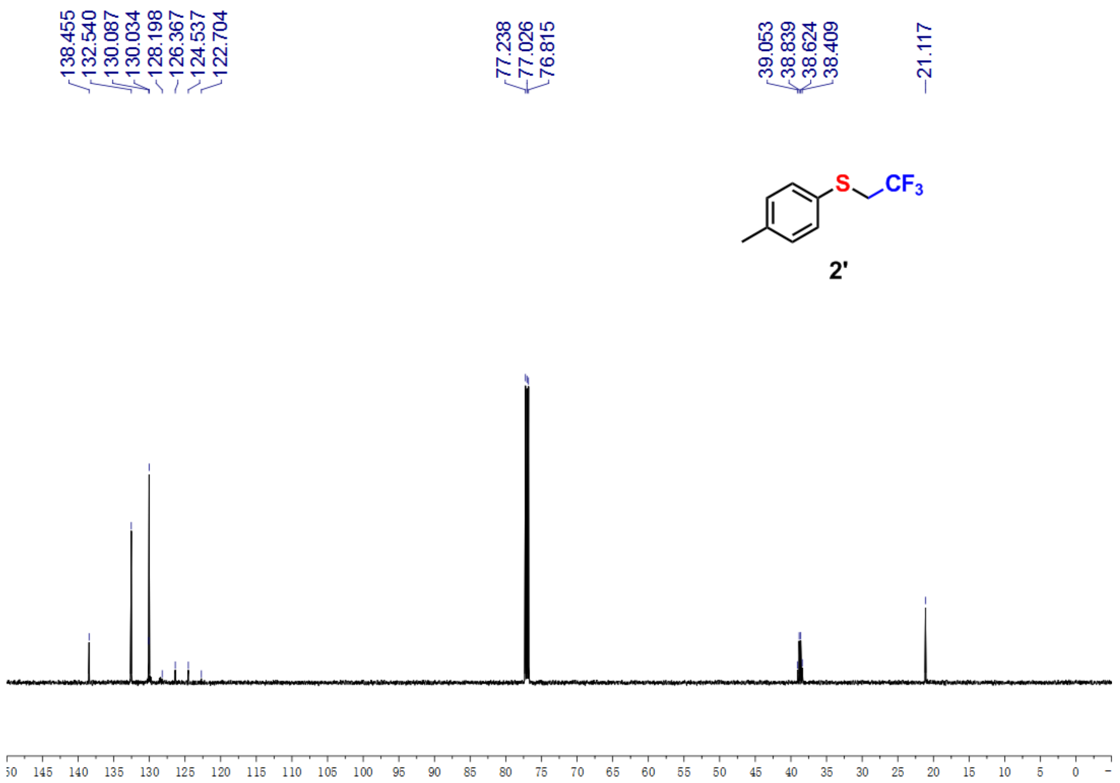

Supplementary Figure 199. ^{13}C NMR of 2’

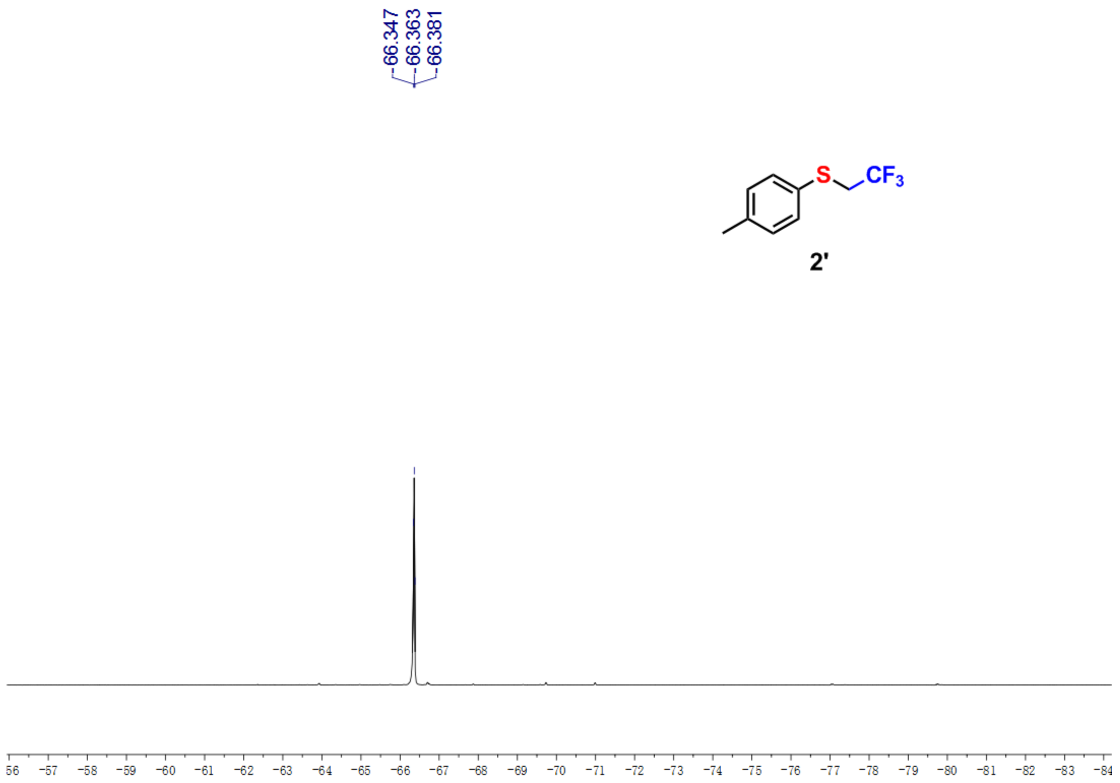

Supplementary Figure 200. ^{19}F NMR of 2’

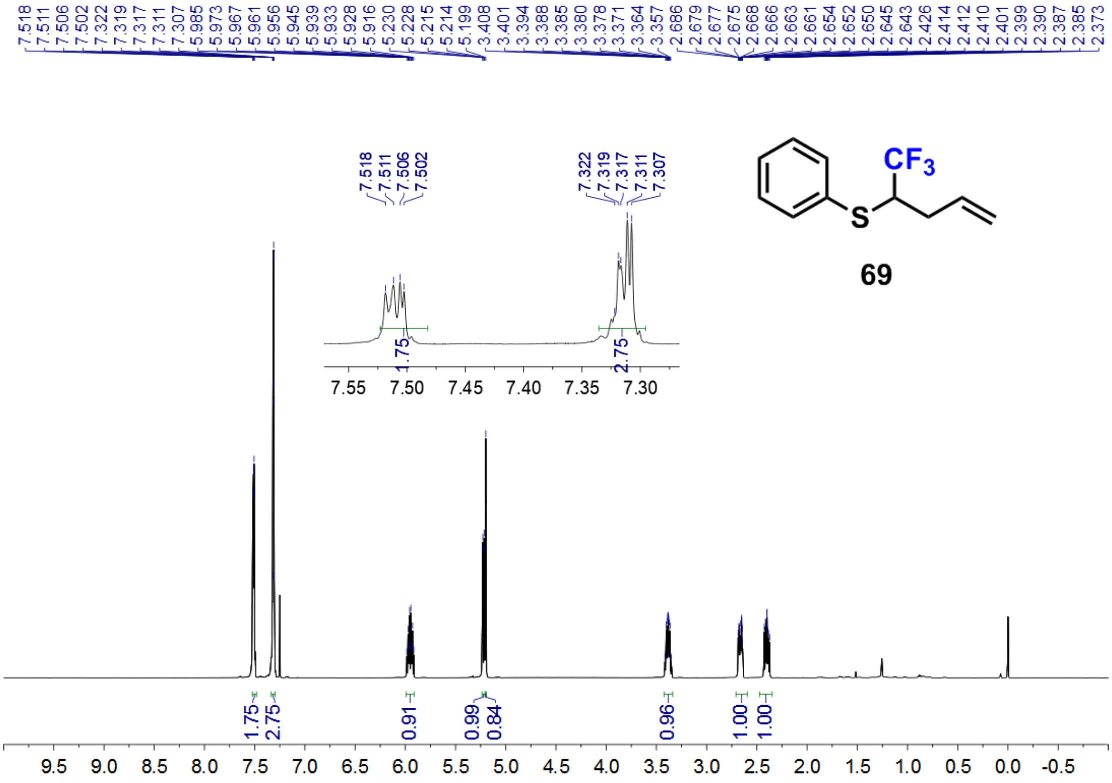

Supplementary Figure 201. ^{1}H NMR of 69

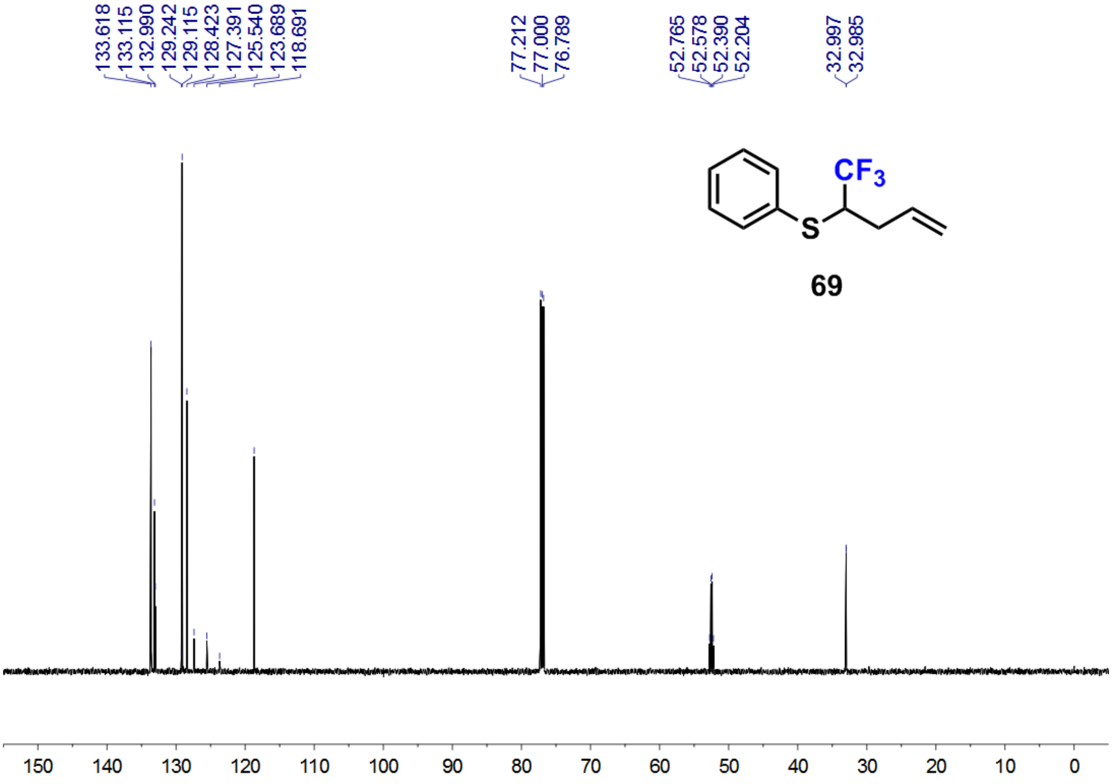

Supplementary Figure 202. ^{13}C NMR of 69

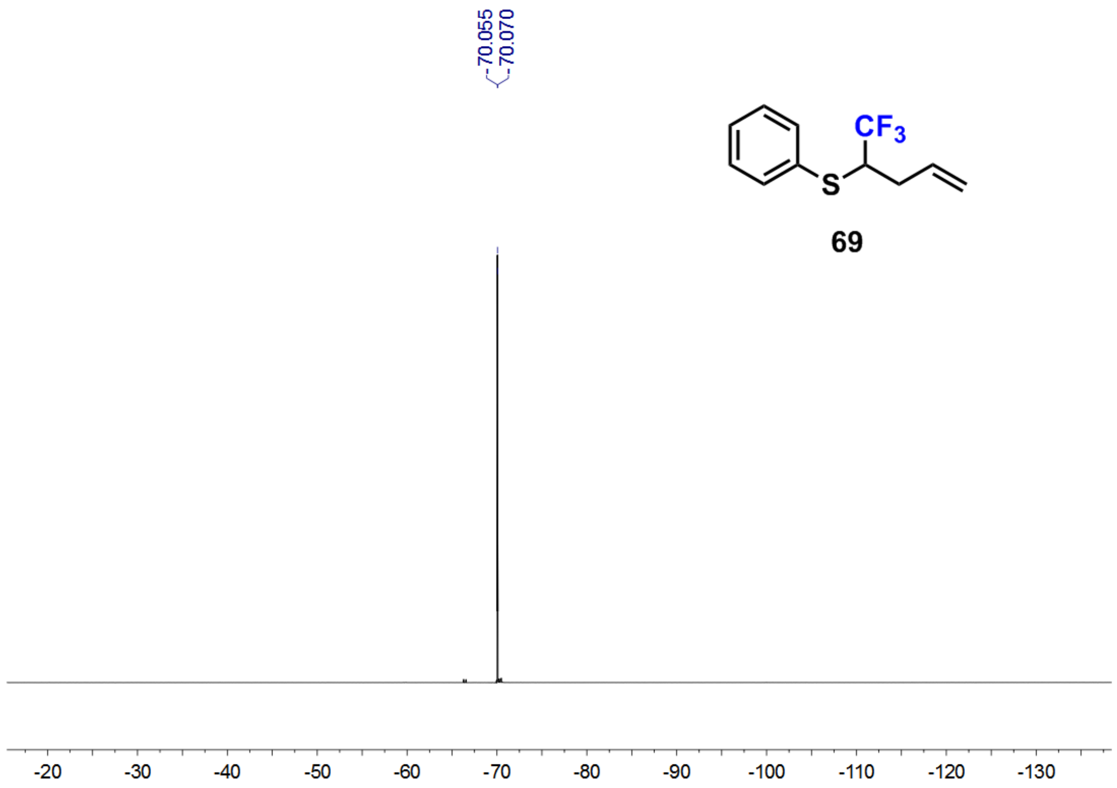

Supplementary Figure 203. ^{19}F NMR of 69

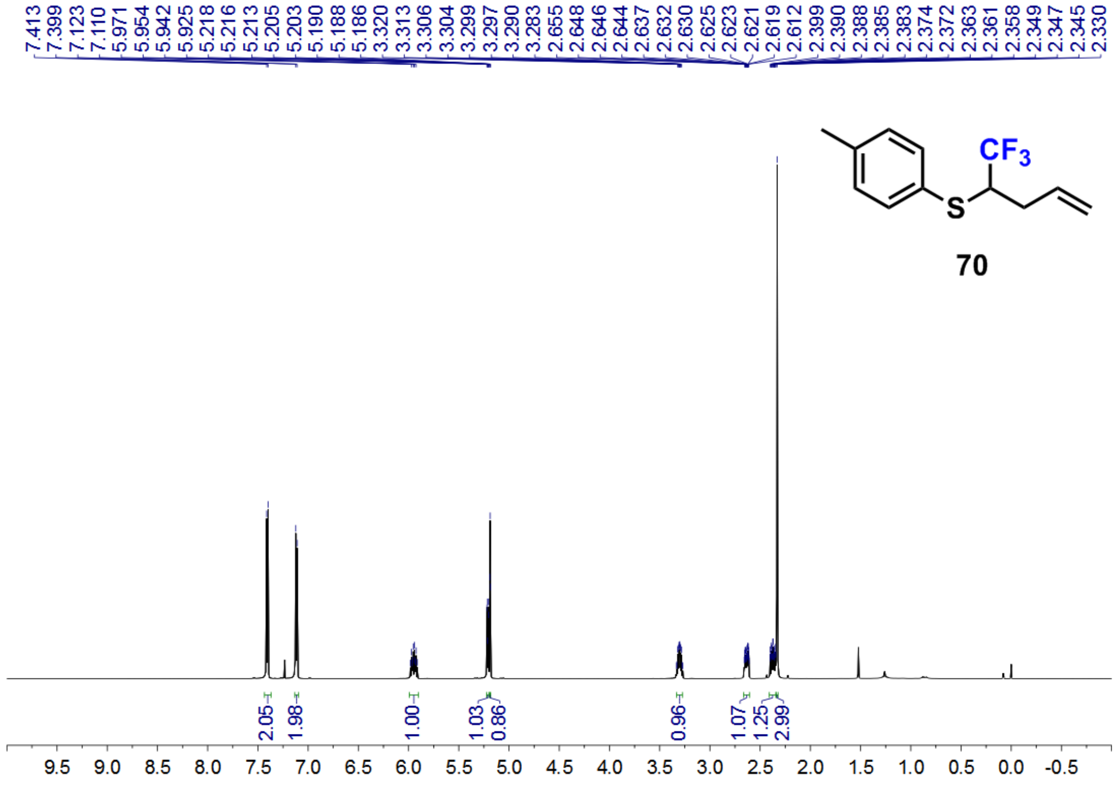

Supplementary Figure 204. ^{1}H NMR of 70

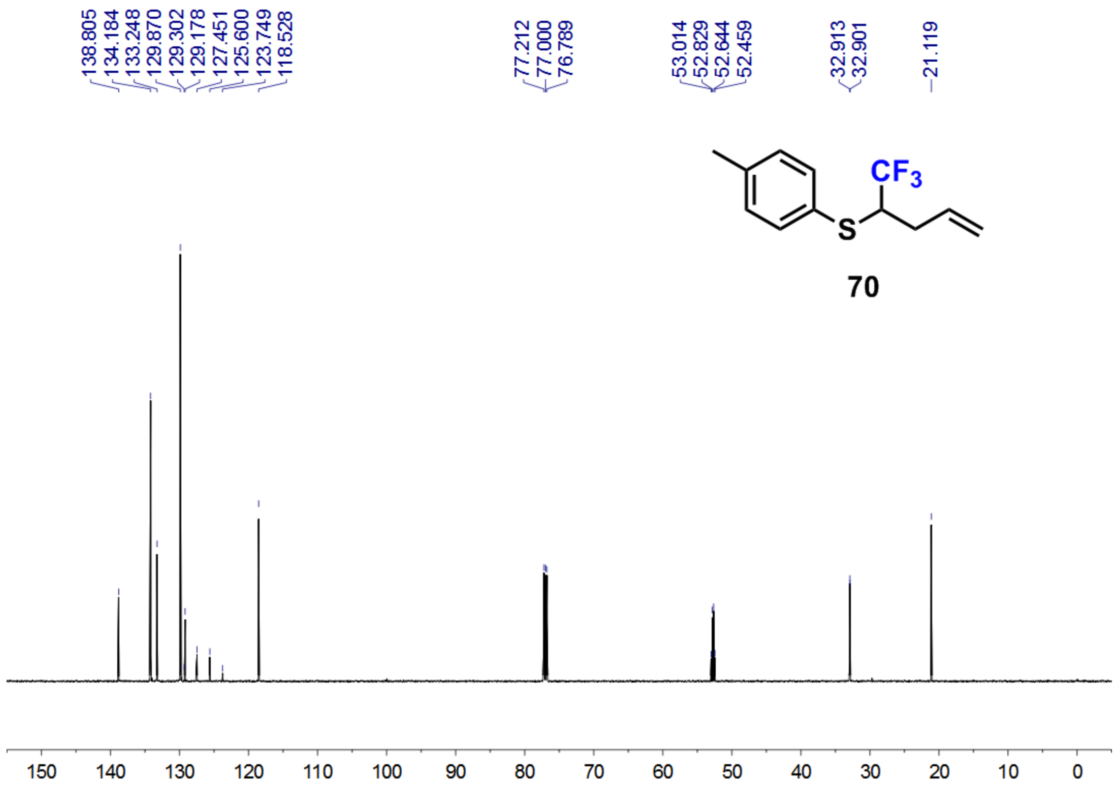

Supplementary Figure 205. ^{13}H NMR of 70

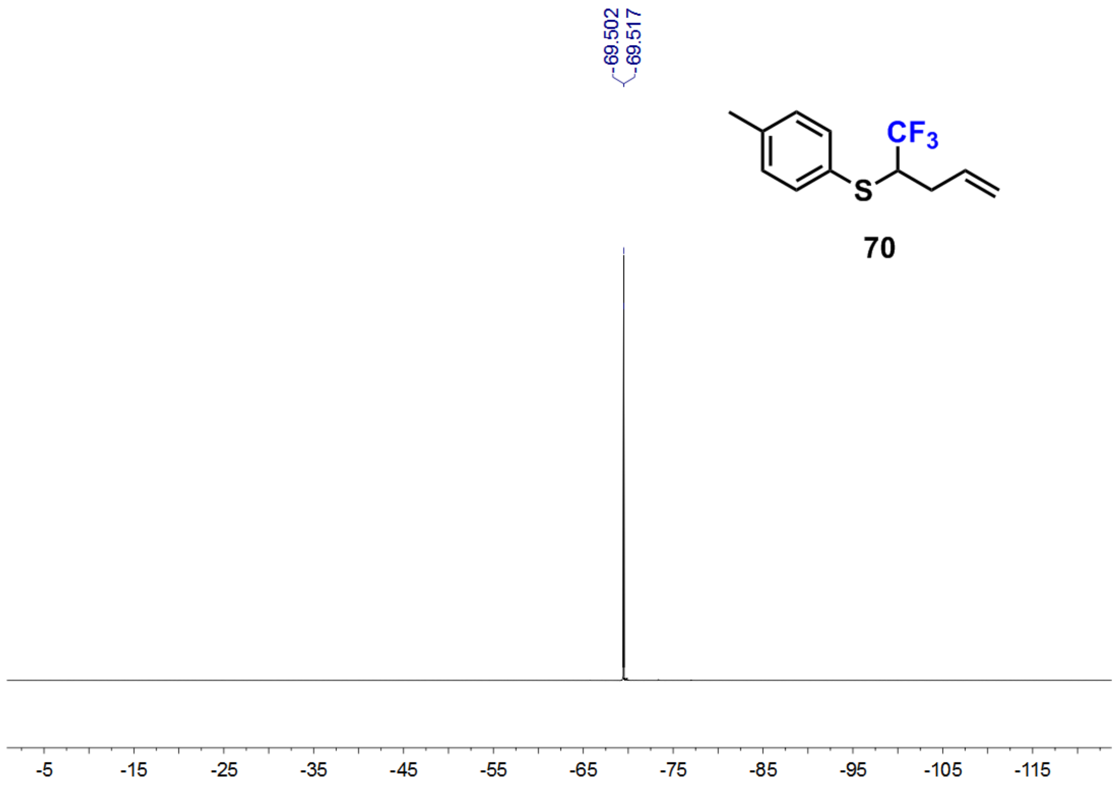

Supplementary Figure 206. ^{19}F NMR of 70

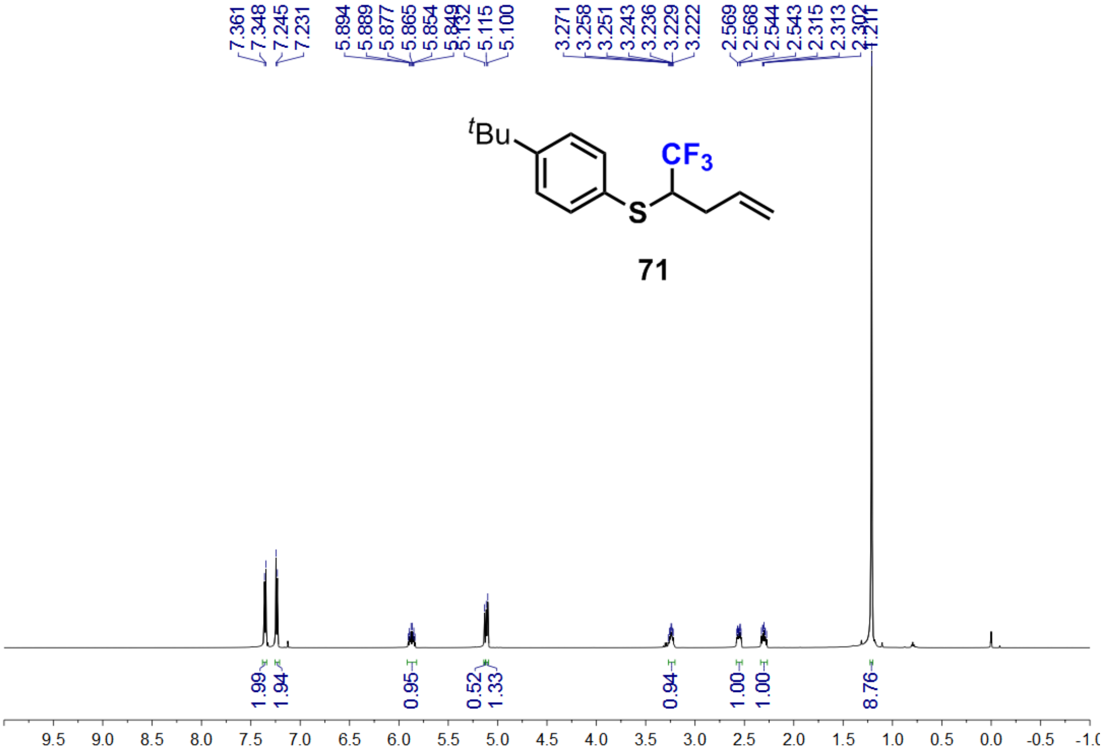

Supplementary Figure 207. ^{1}H NMR of 71

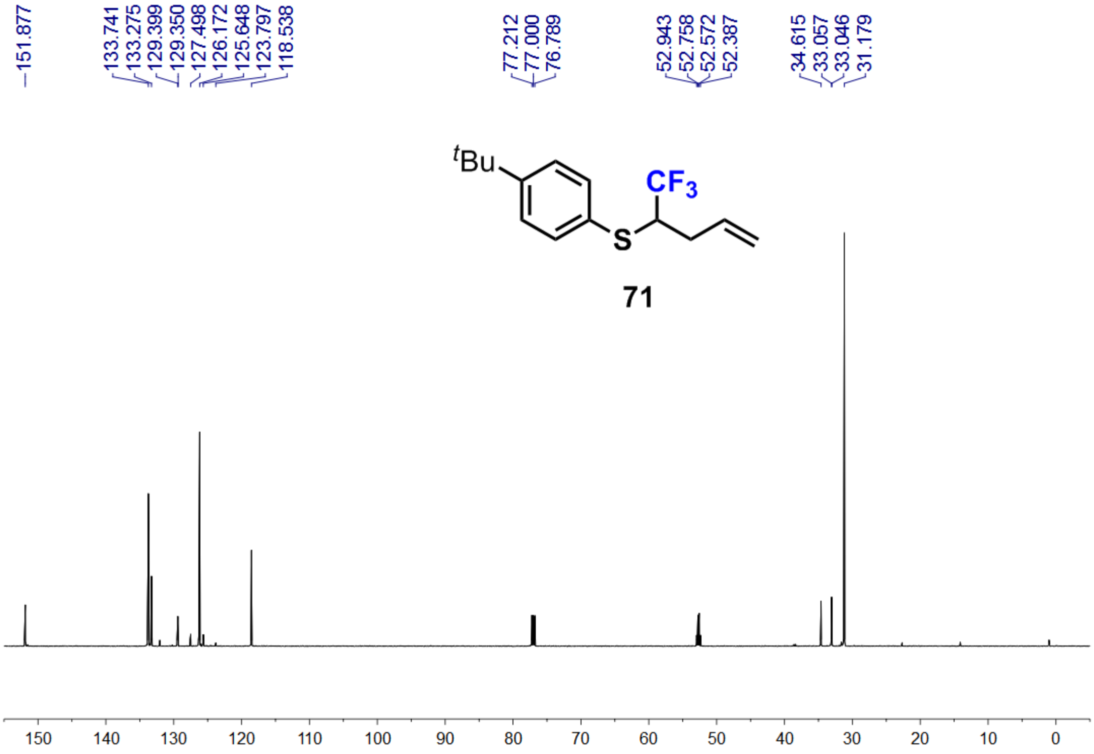

Supplementary Figure 208. ^{13}C NMR of 71

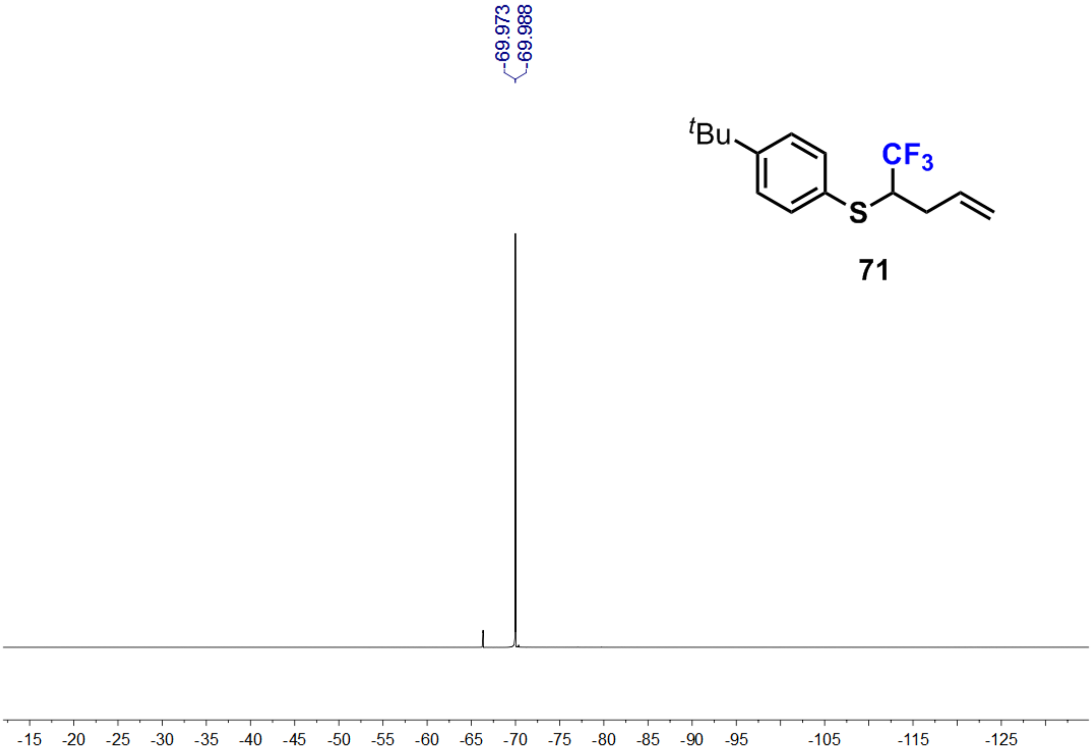

Supplementary Figure 209. ^{19}F NMR of 71

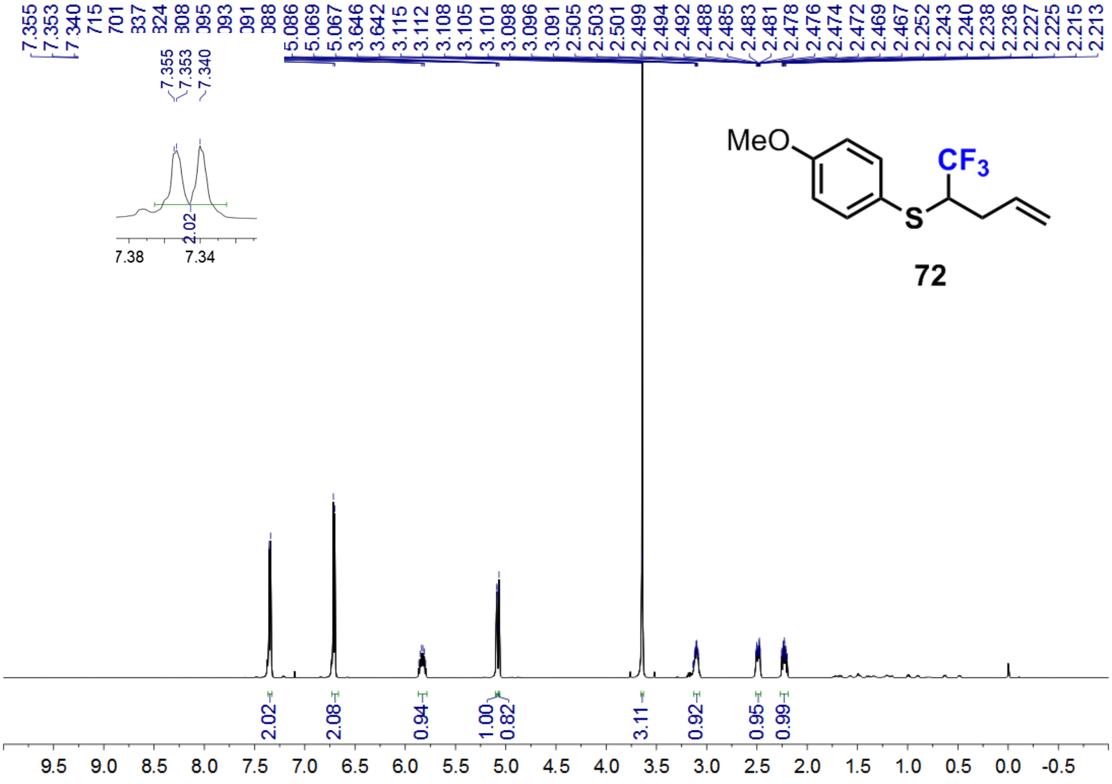

Supplementary Figure 210. ^{1}H NMR of 72

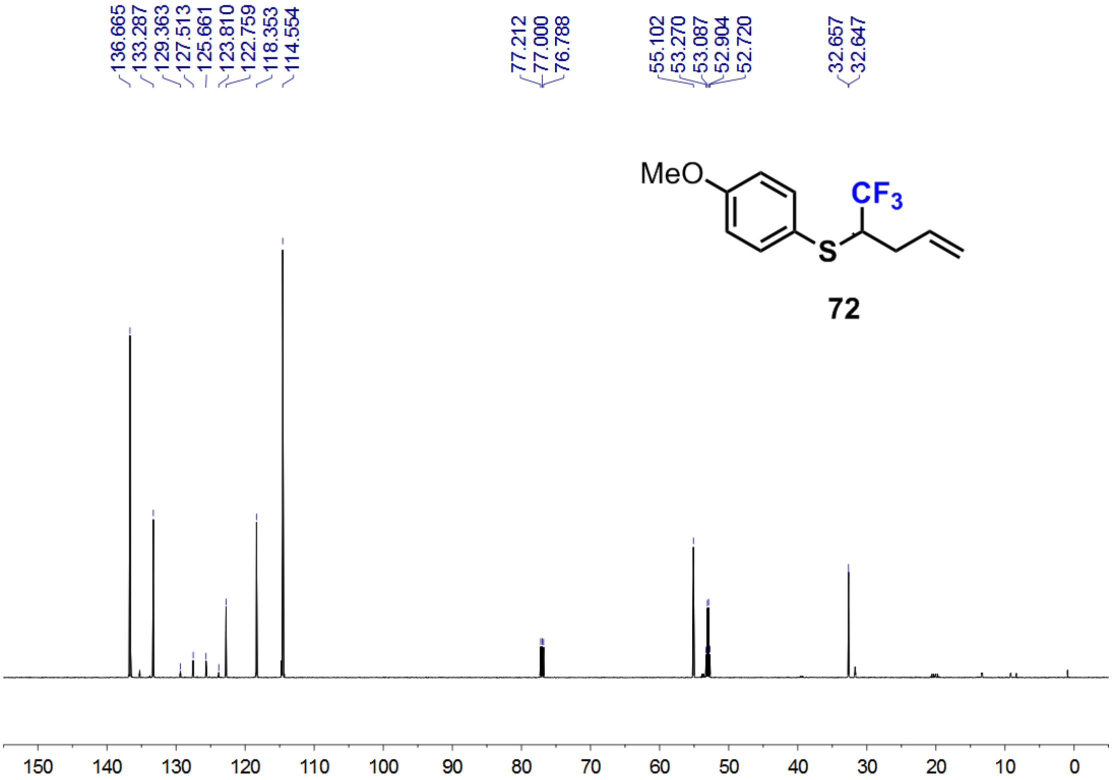

Supplementary Figure 211. ^{13}C NMR of 72

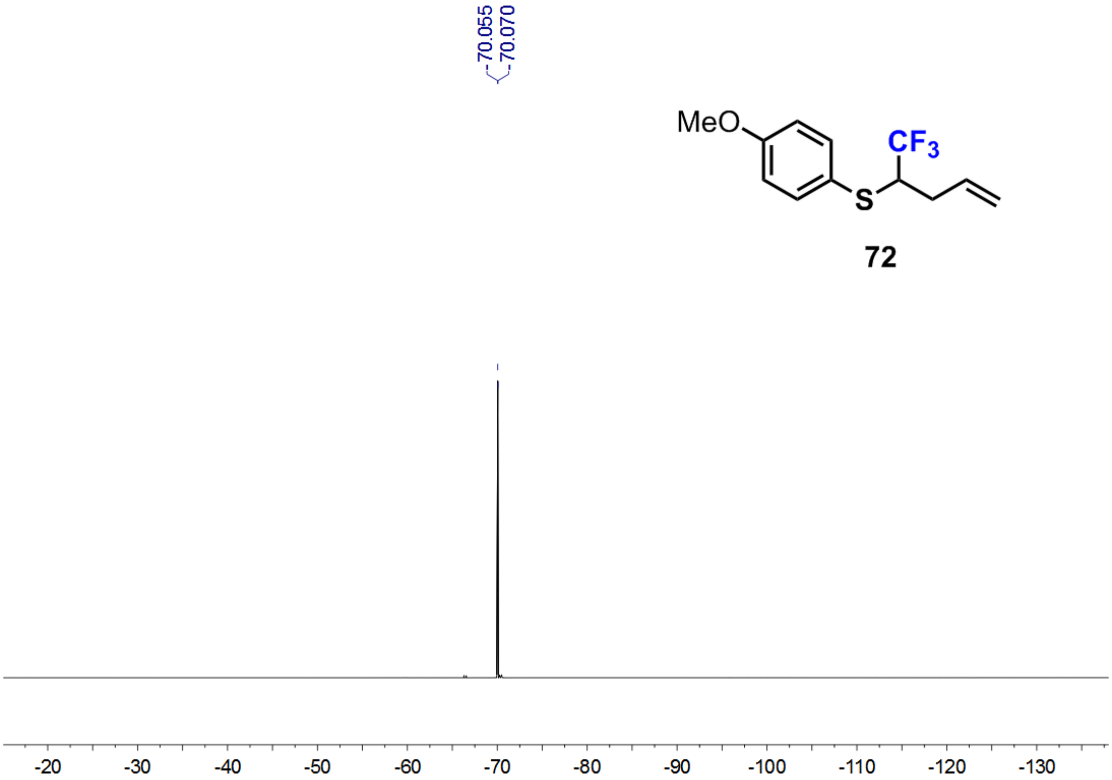

Supplementary Figure 212. ^{19}F NMR of 72

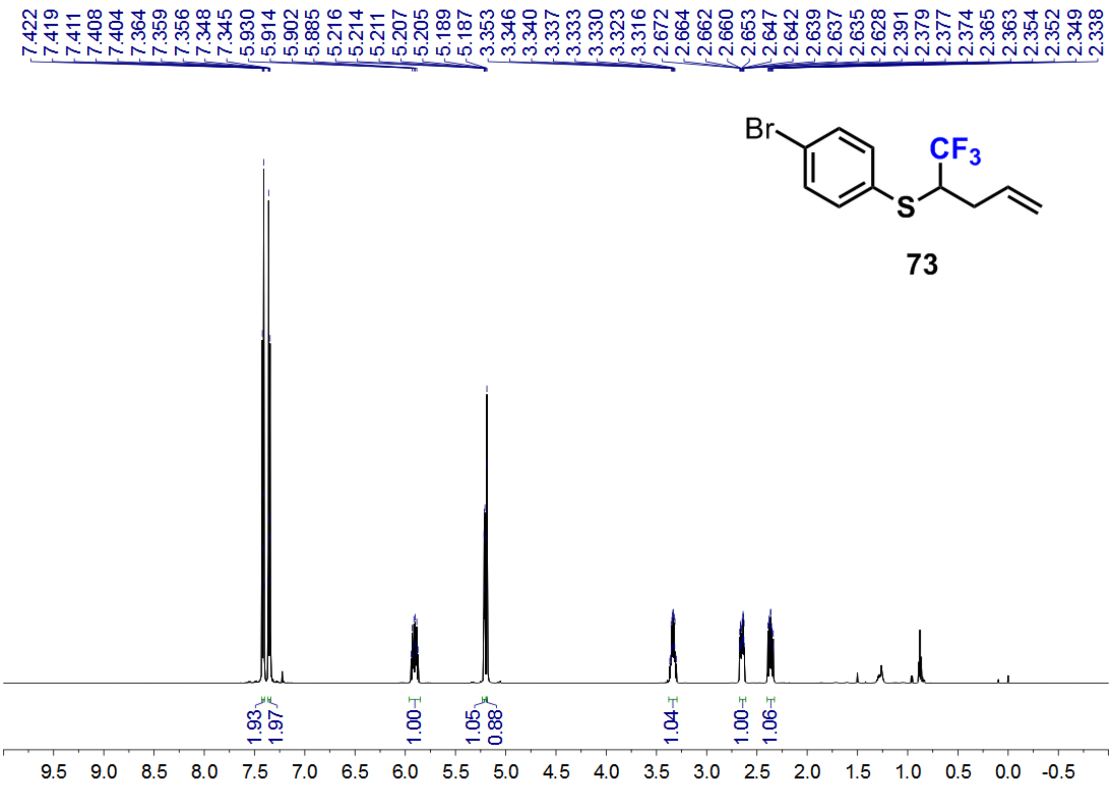

Supplementary Figure 213. ^{1}H NMR of 73

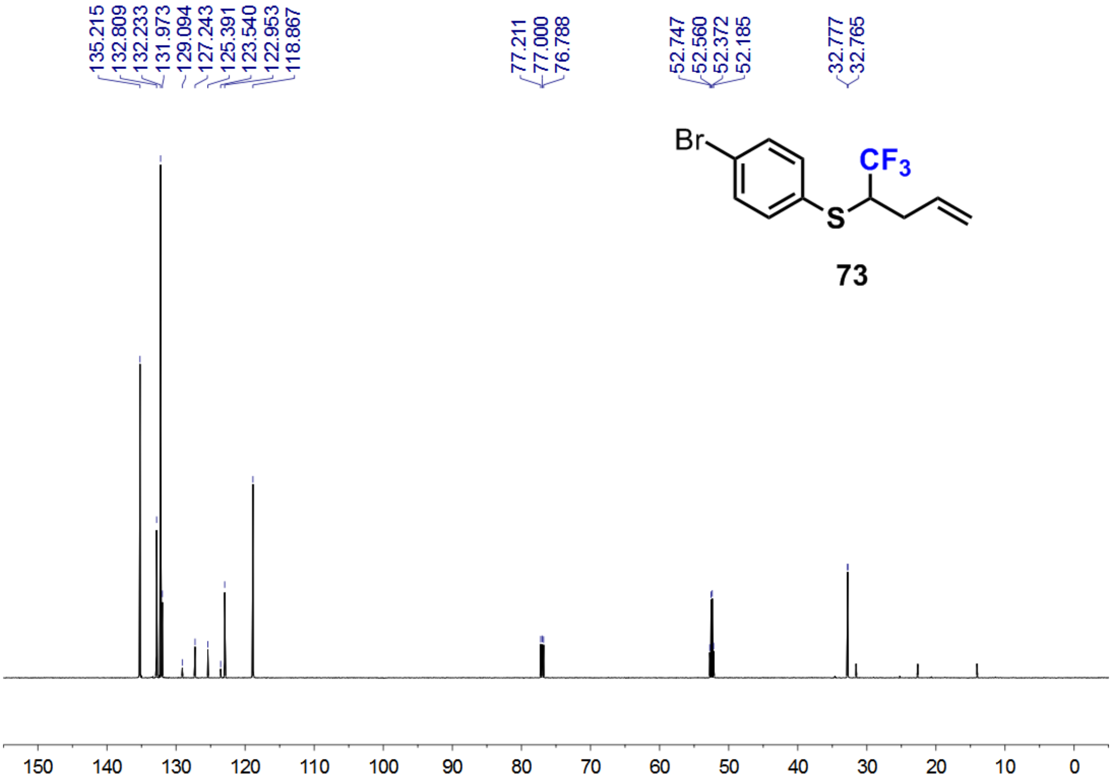

Supplementary Figure 214. ^{13}C NMR of 73

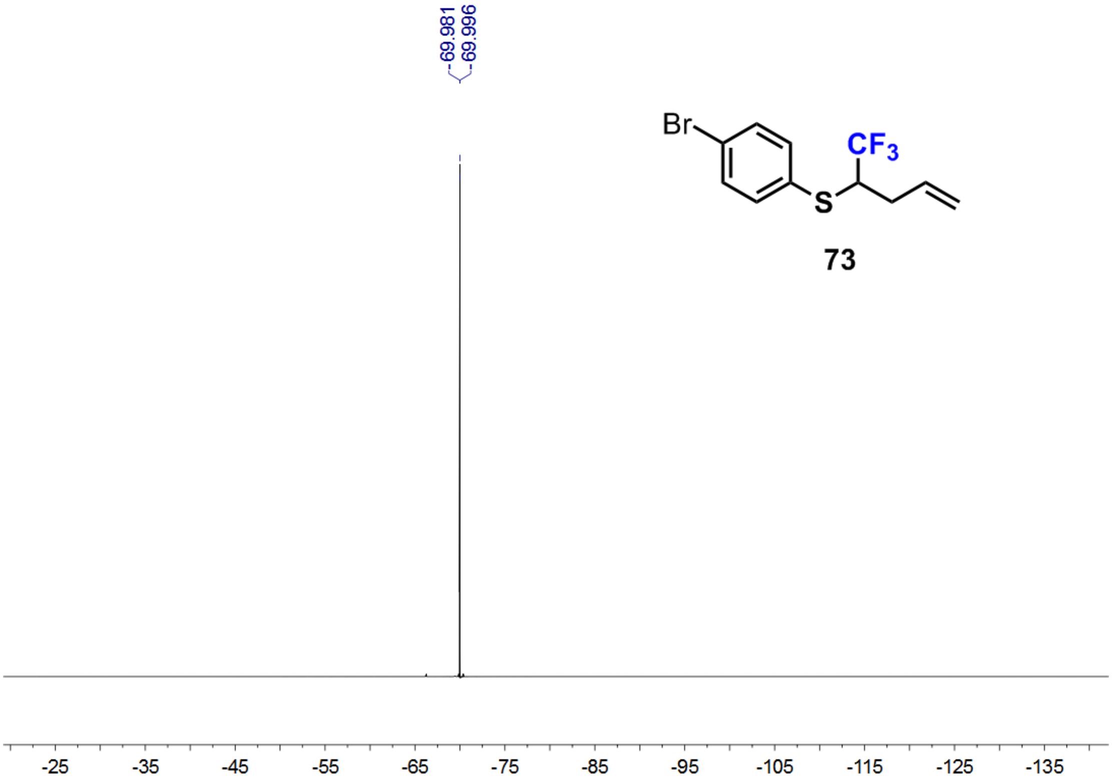

Supplementary Figure 215. ^{19}F NMR of 73

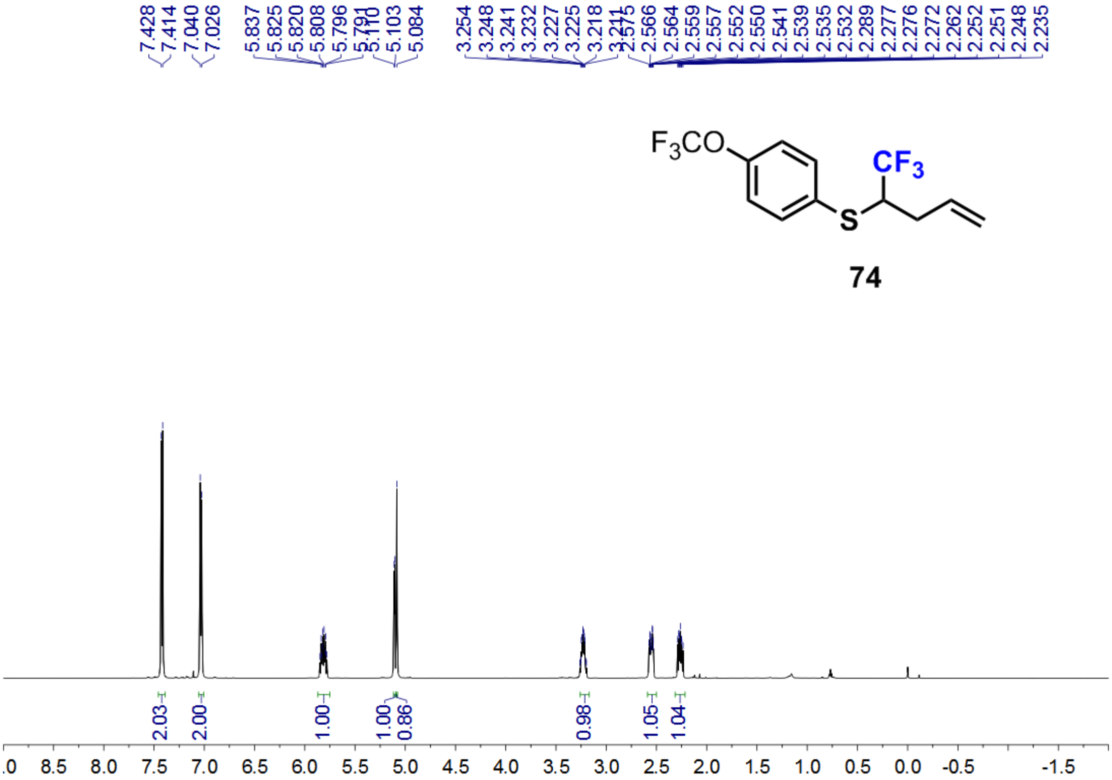

Supplementary Figure 216. ^{1}H NMR of 74

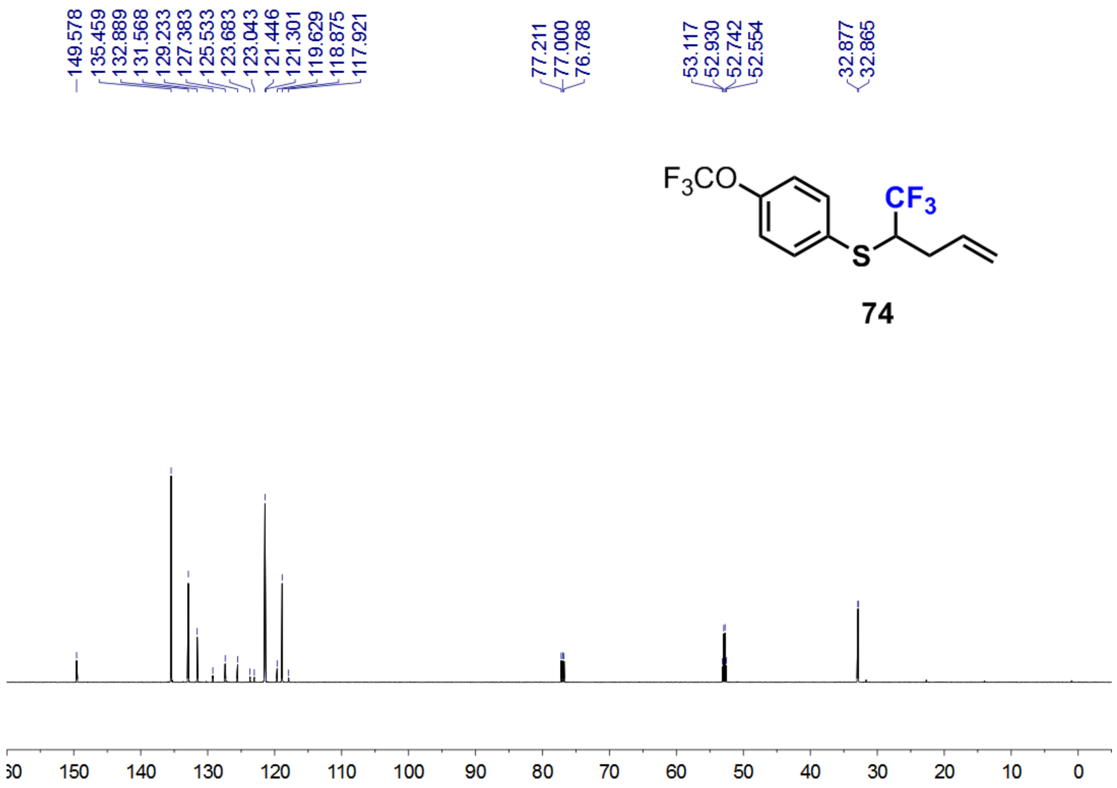

Supplementary Figure 217. ^{13}C NMR of 74

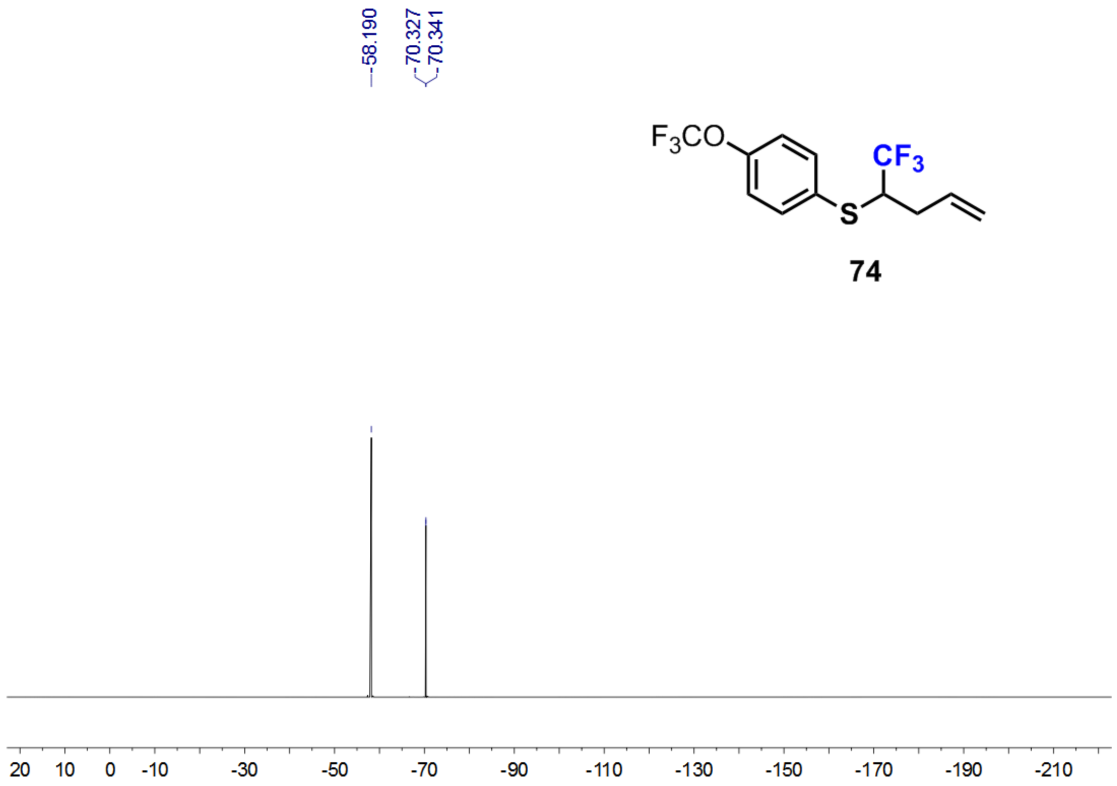

Supplementary Figure 218. ^{19}F NMR of 74

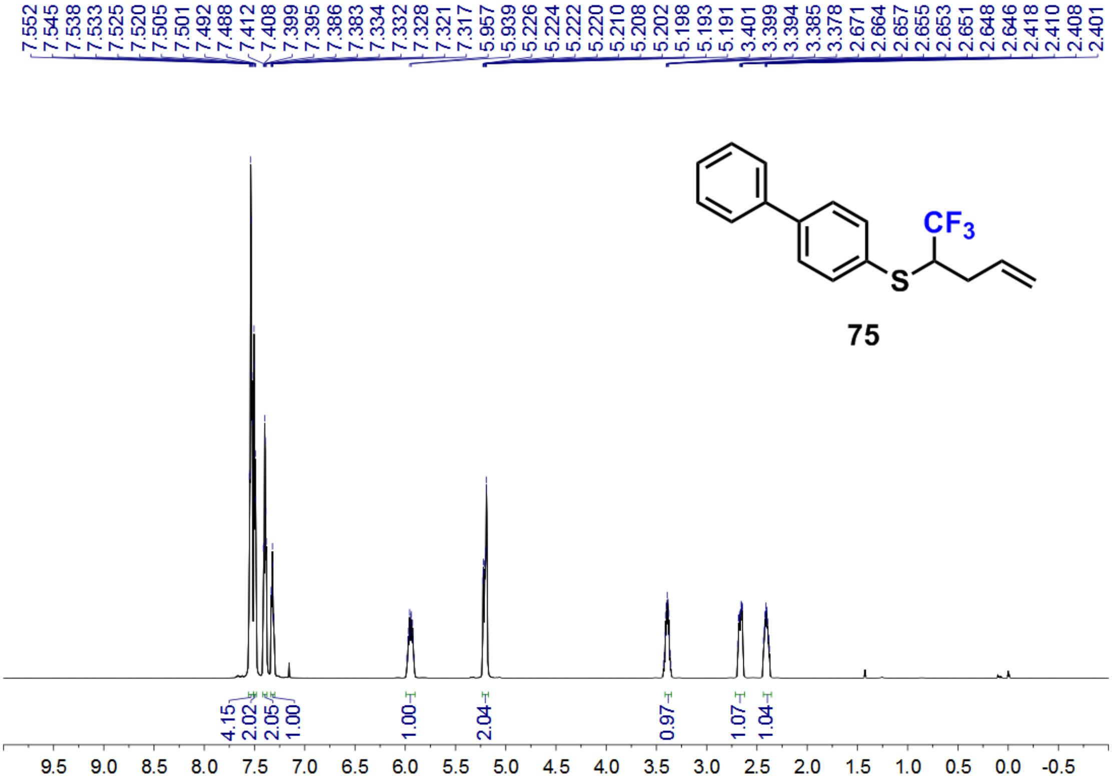

Supplementary Figure 219. ^{1}H NMR of 75

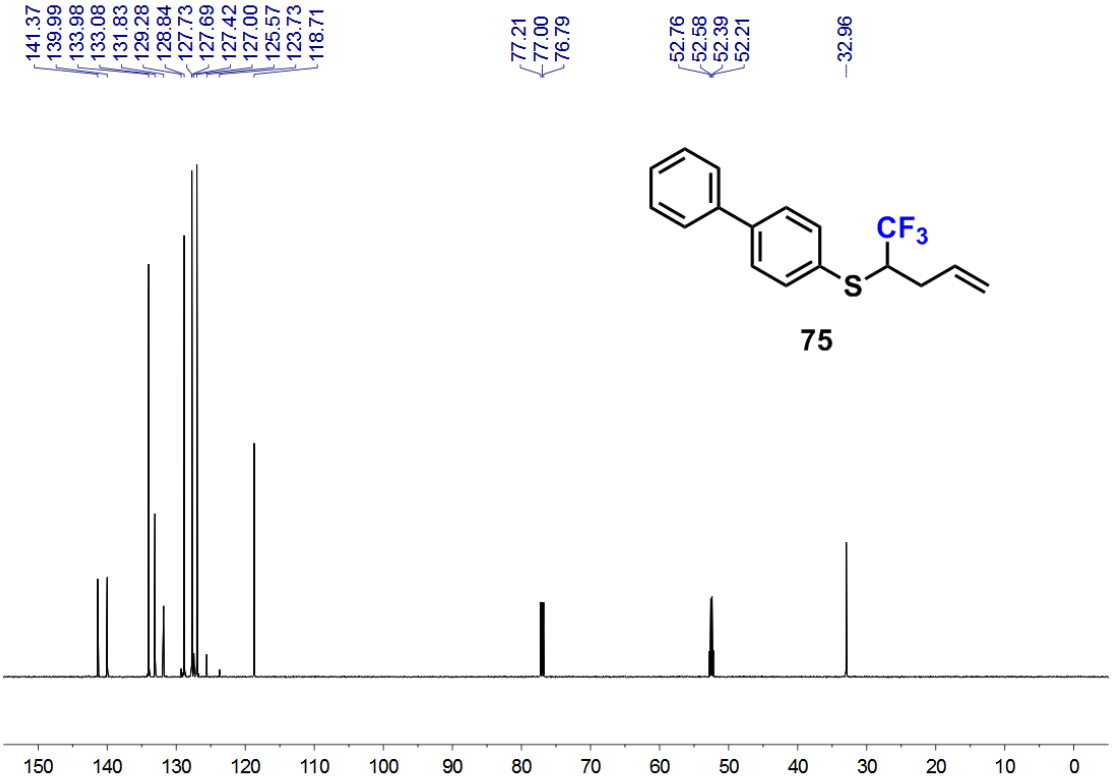

Supplementary Figure 220. ^{13}C NMR of 75

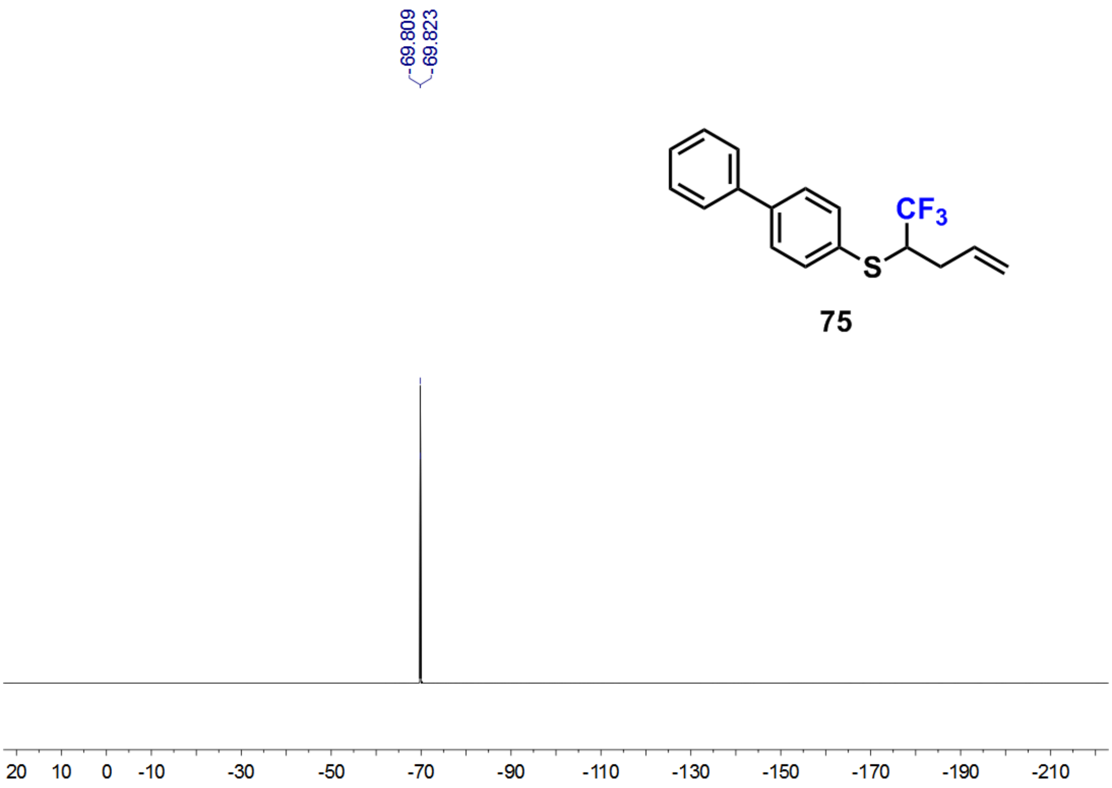

Supplementary Figure 221. ^{19}F NMR of 75

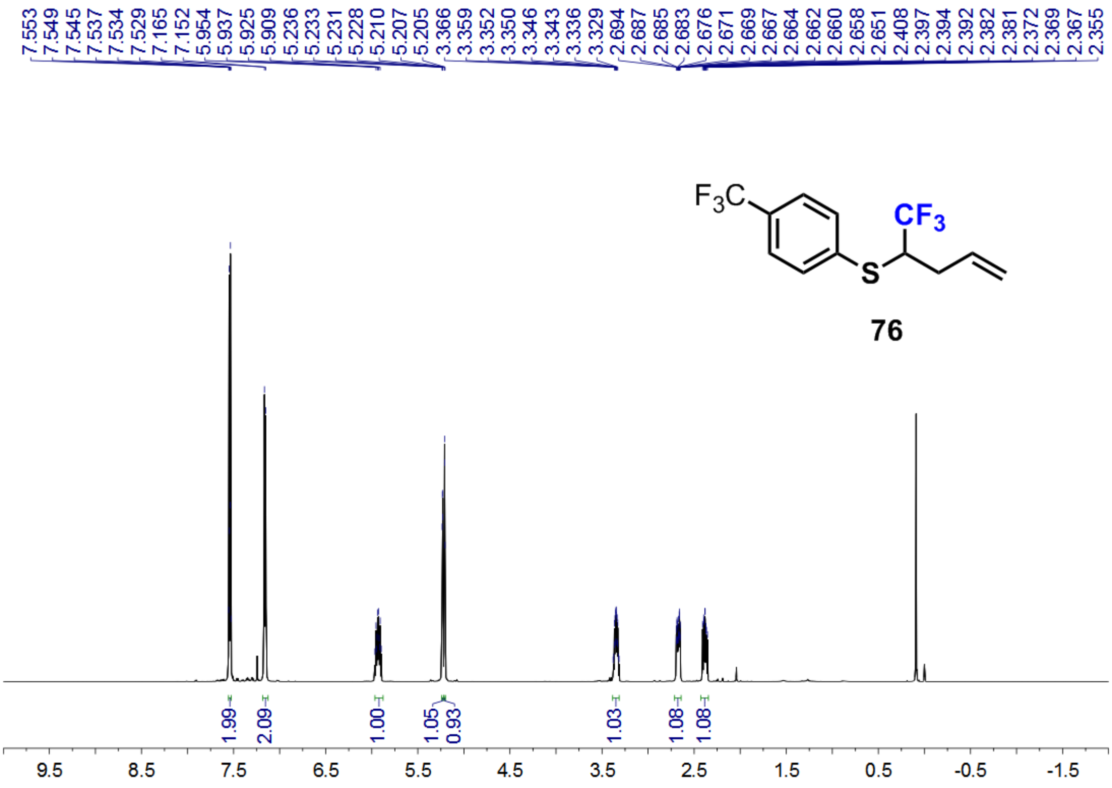

Supplementary Figure 222. ^{1}H NMR of 76

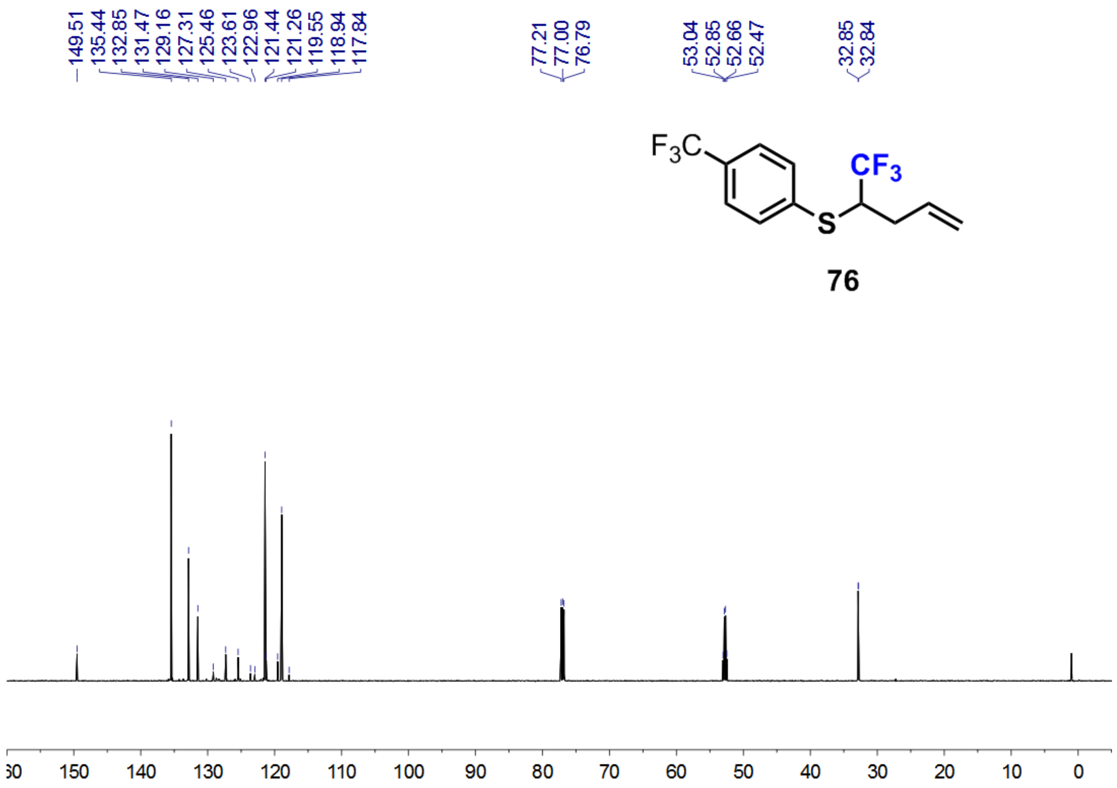

Supplementary Figure 223. ^{13}C NMR of 77

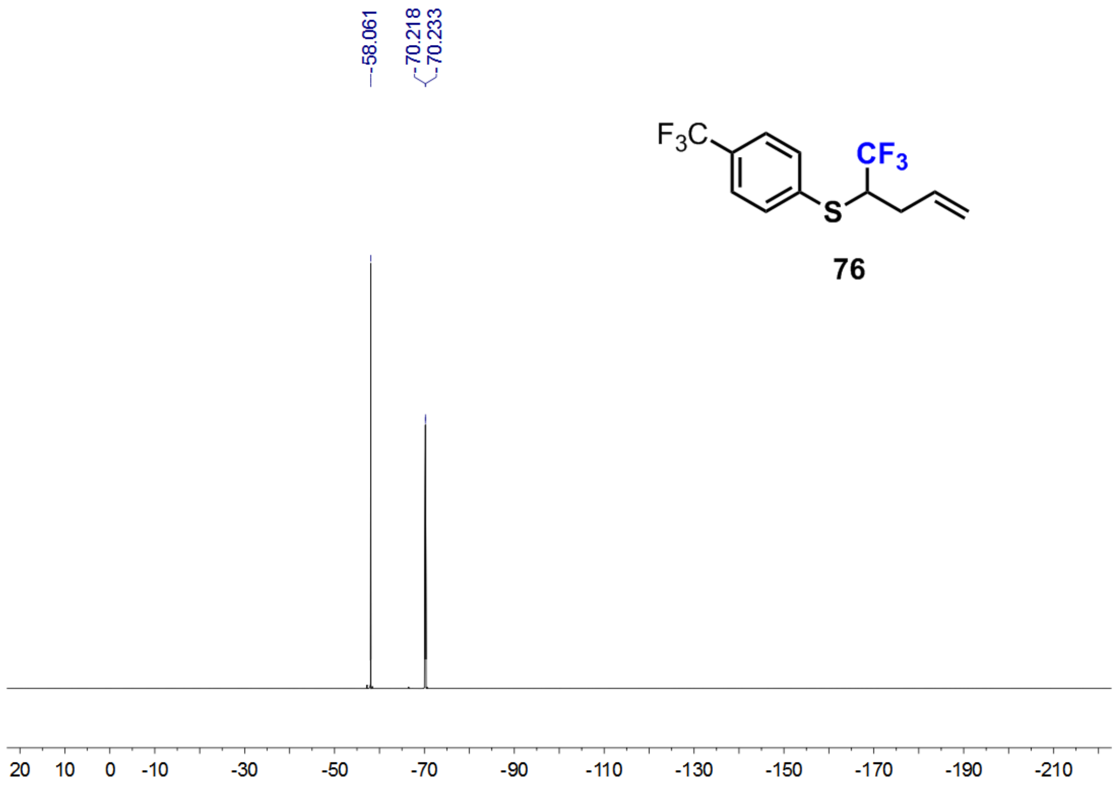

Supplementary Figure 224. ^{19}F NMR of 76

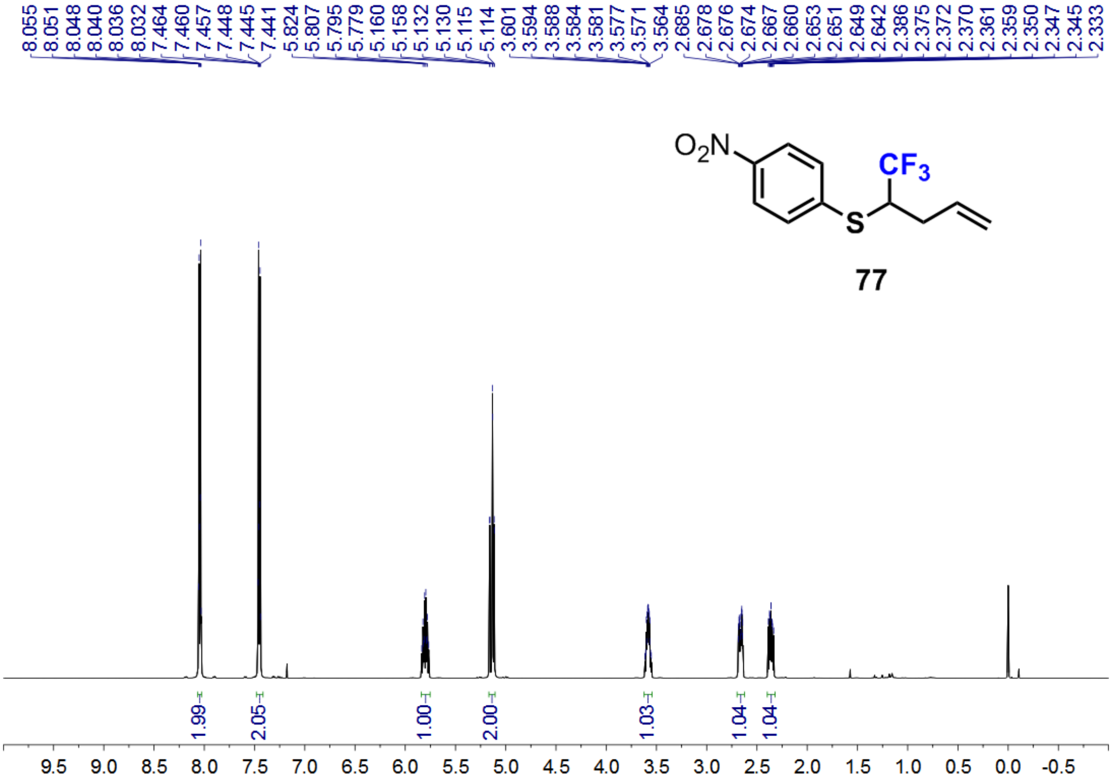

Supplementary Figure 225. ^{1}H NMR of 77

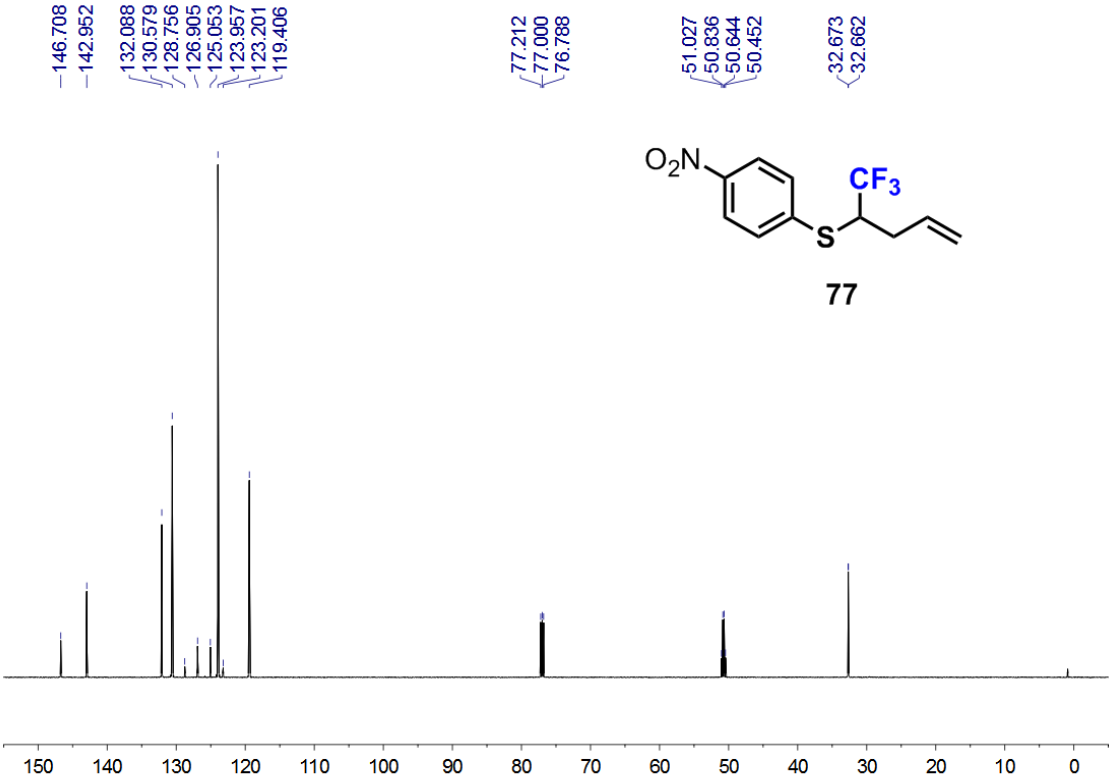

Supplementary Figure 226. ^{13}C NMR of 77

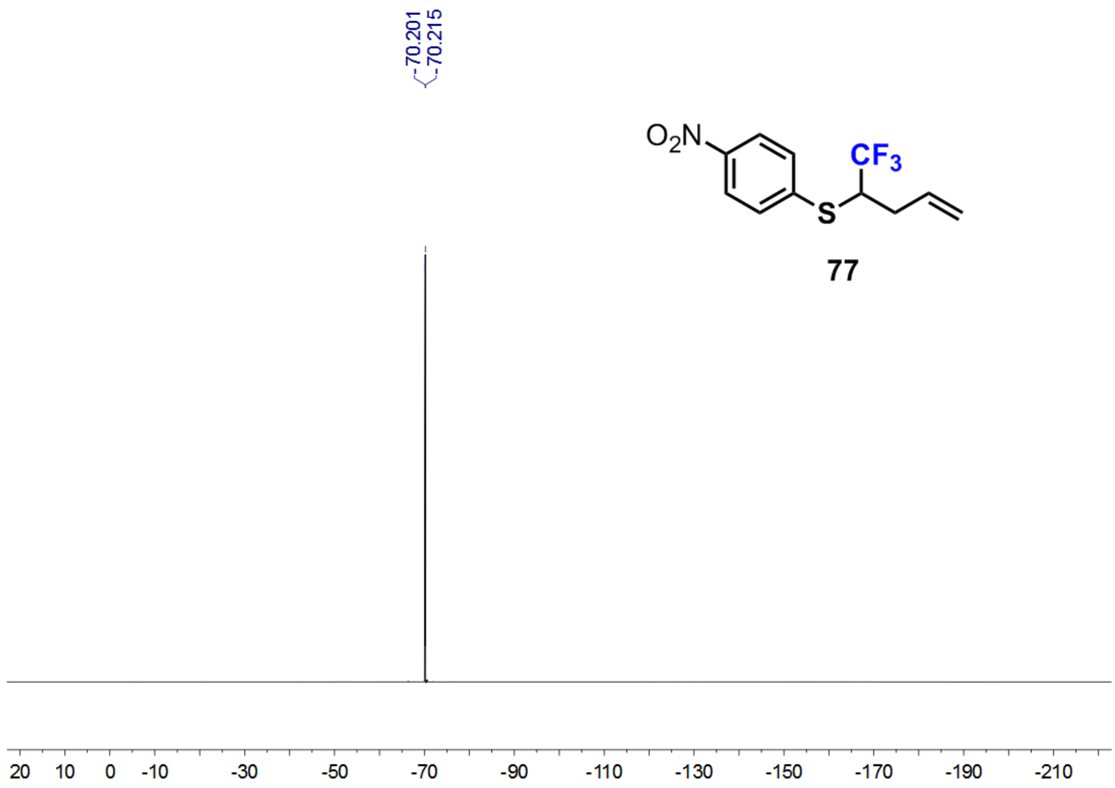

Supplementary Figure 227. ^{19}F NMR of 77

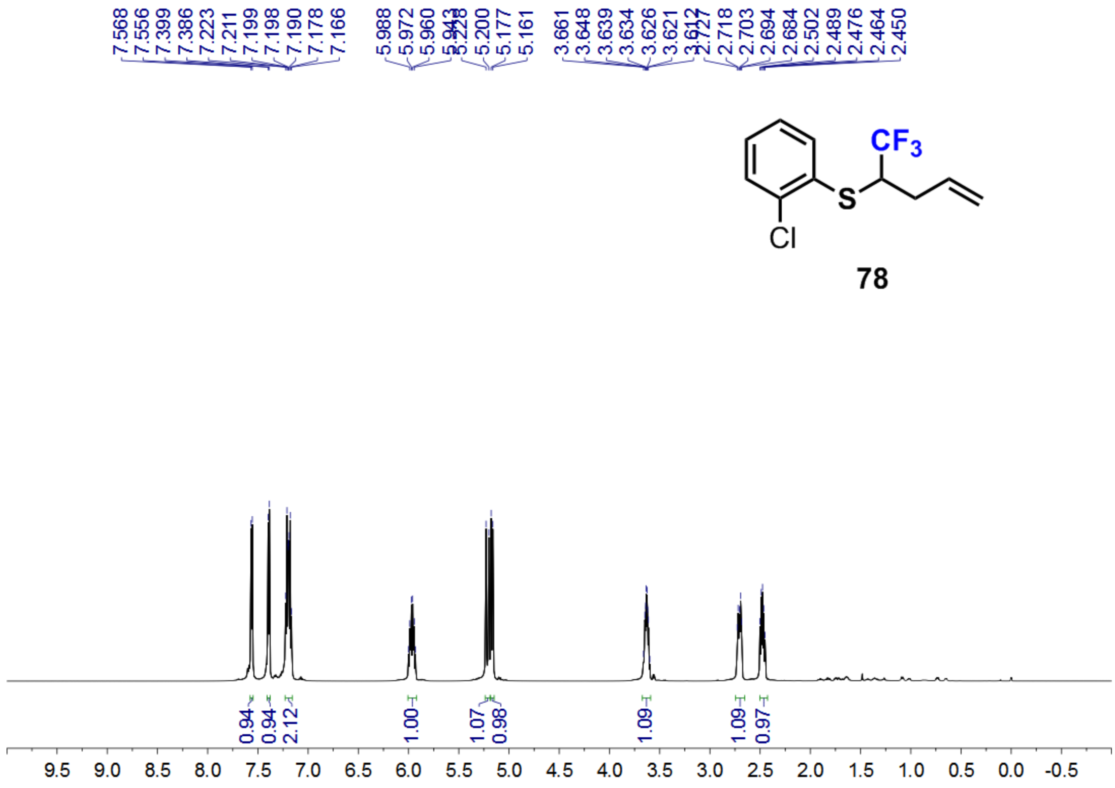

Supplementary Figure 228. ^{1}H NMR of 78

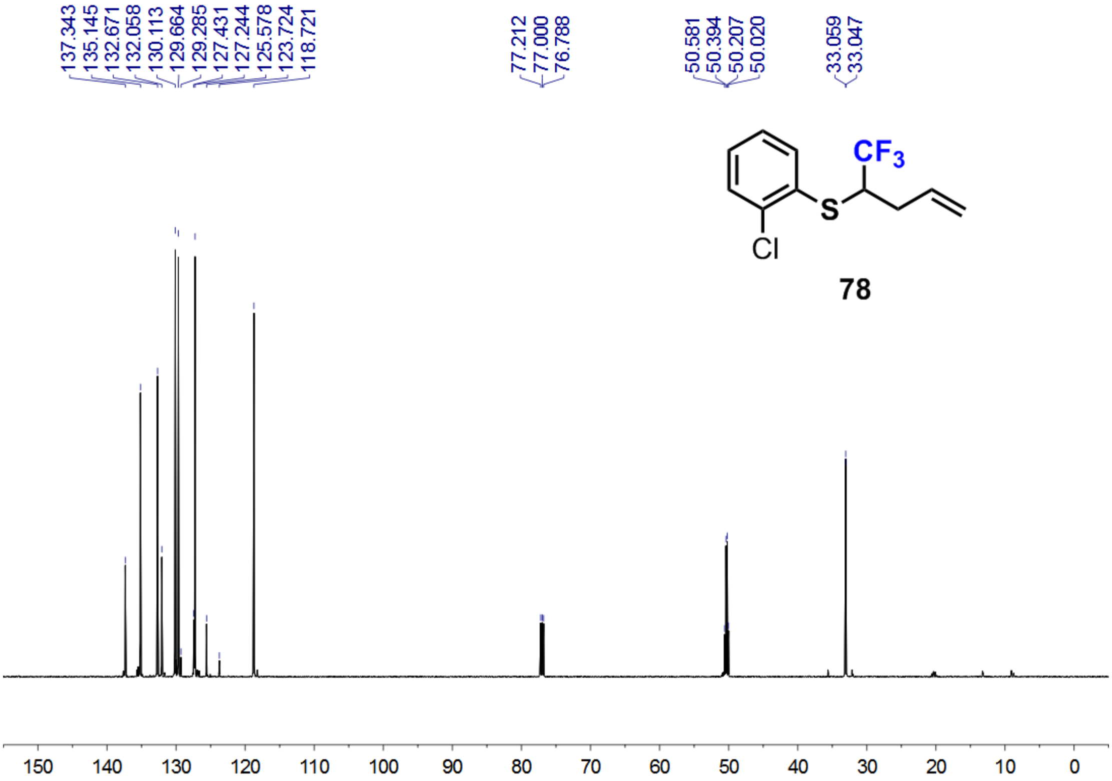

Supplementary Figure 229. ^{13}C NMR of 78

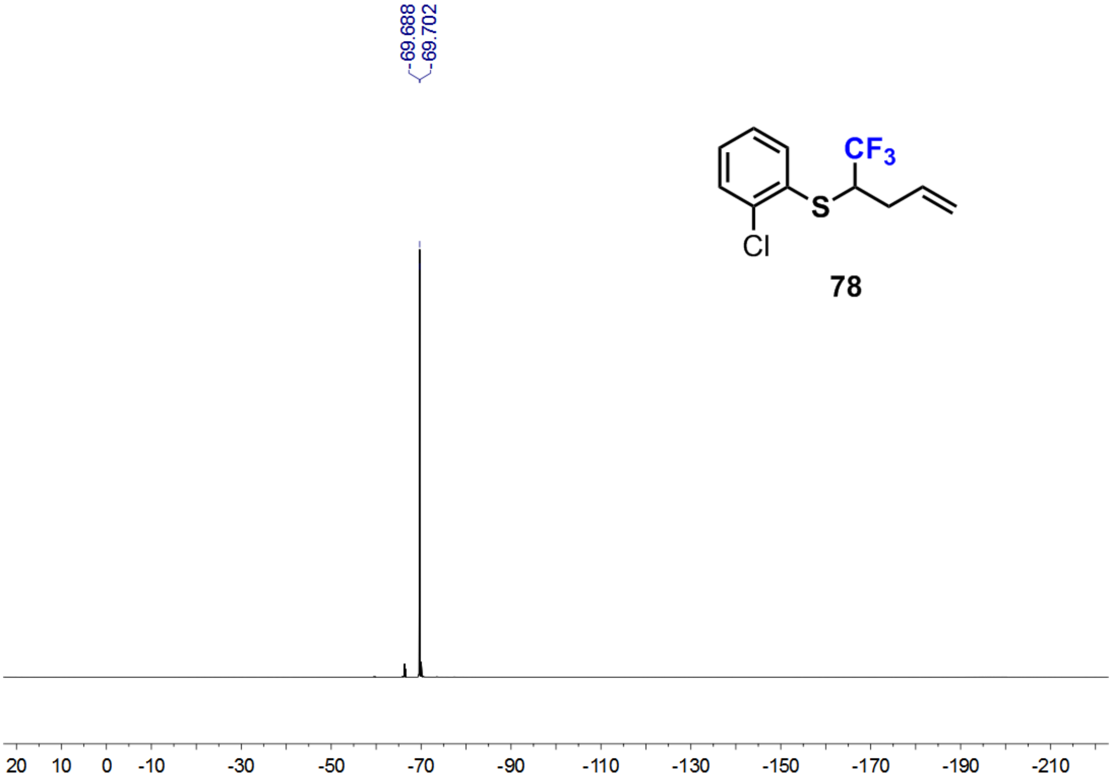

Supplementary Figure 230. ^{19}F NMR of 78

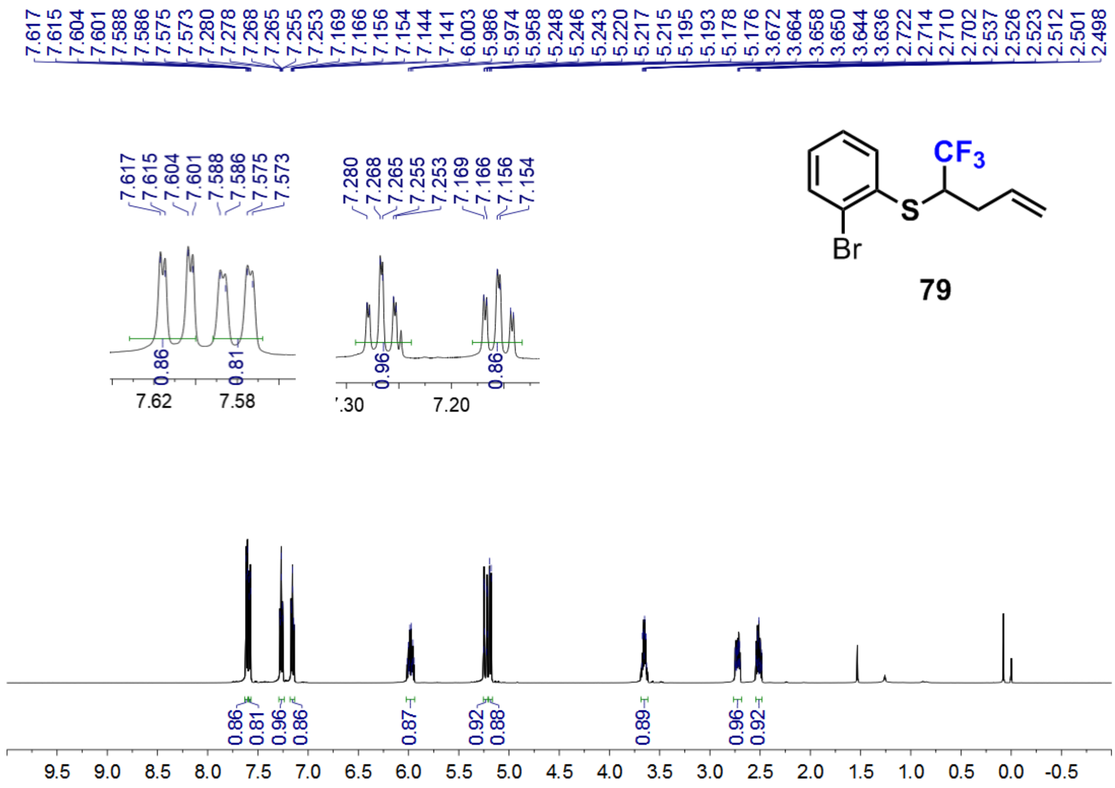

Supplementary Figure 231. ^{1}H NMR of 79

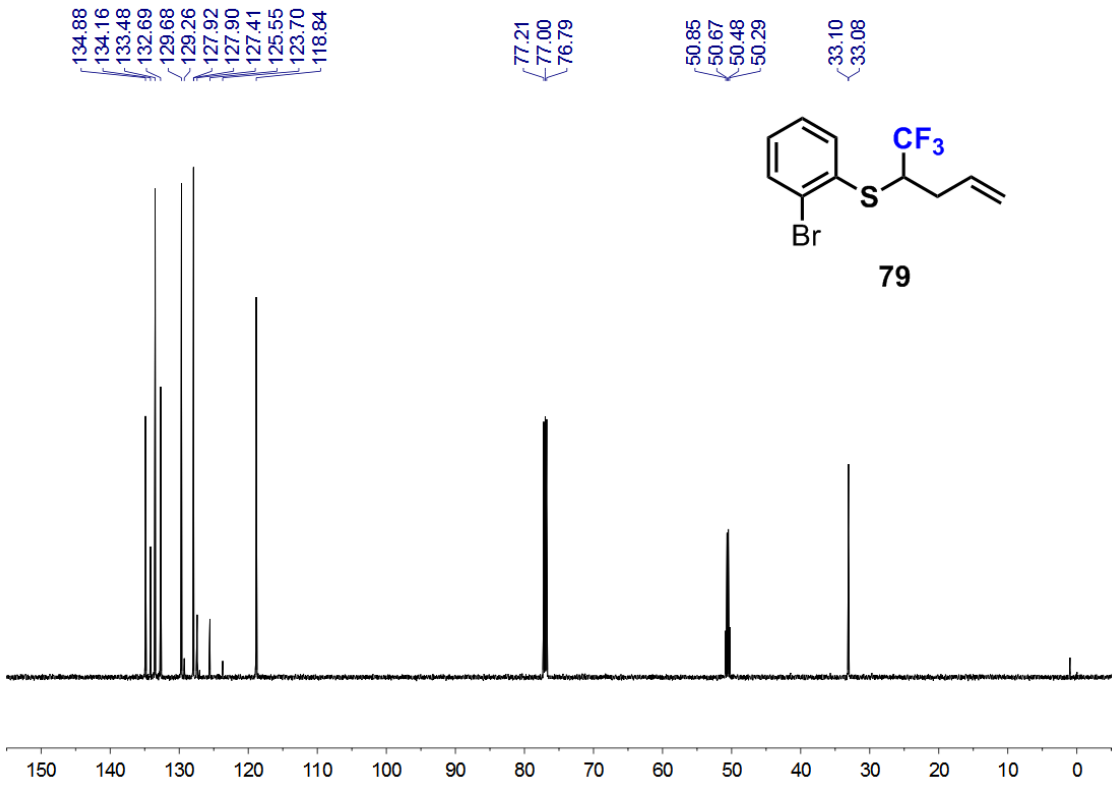

Supplementary Figure 232. ^{13}C NMR of 79

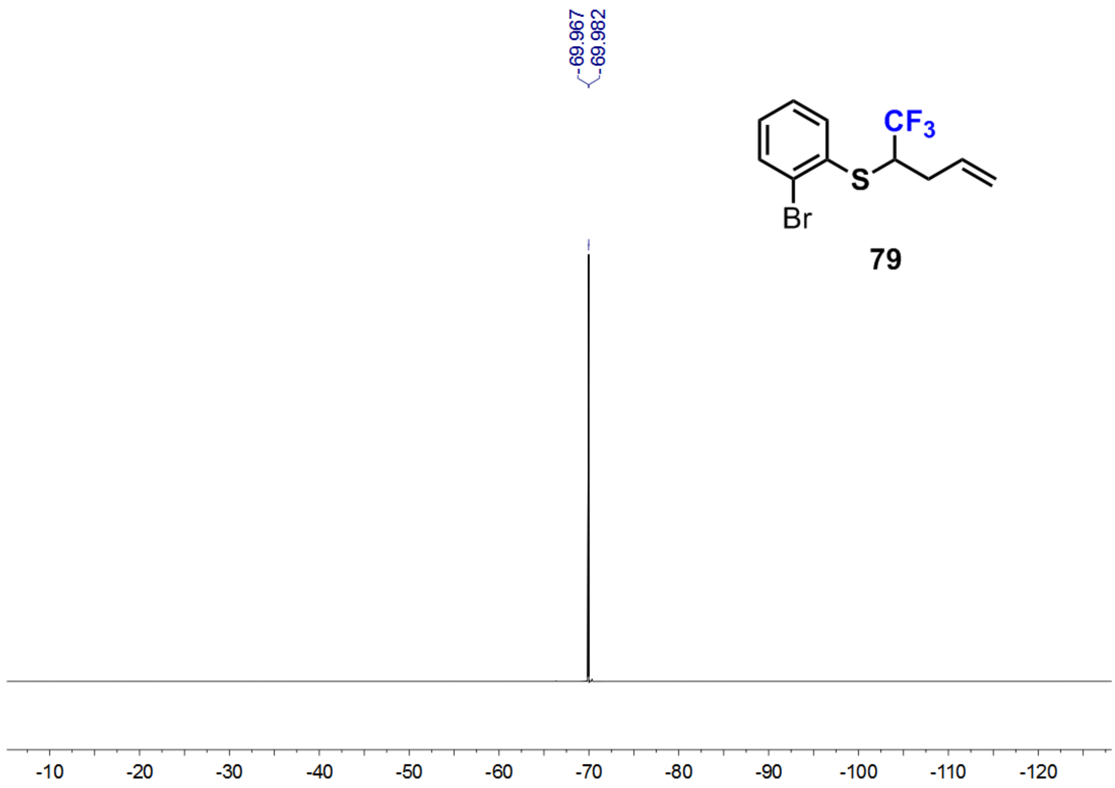

Supplementary Figure 233. ^{19}F NMR of 79

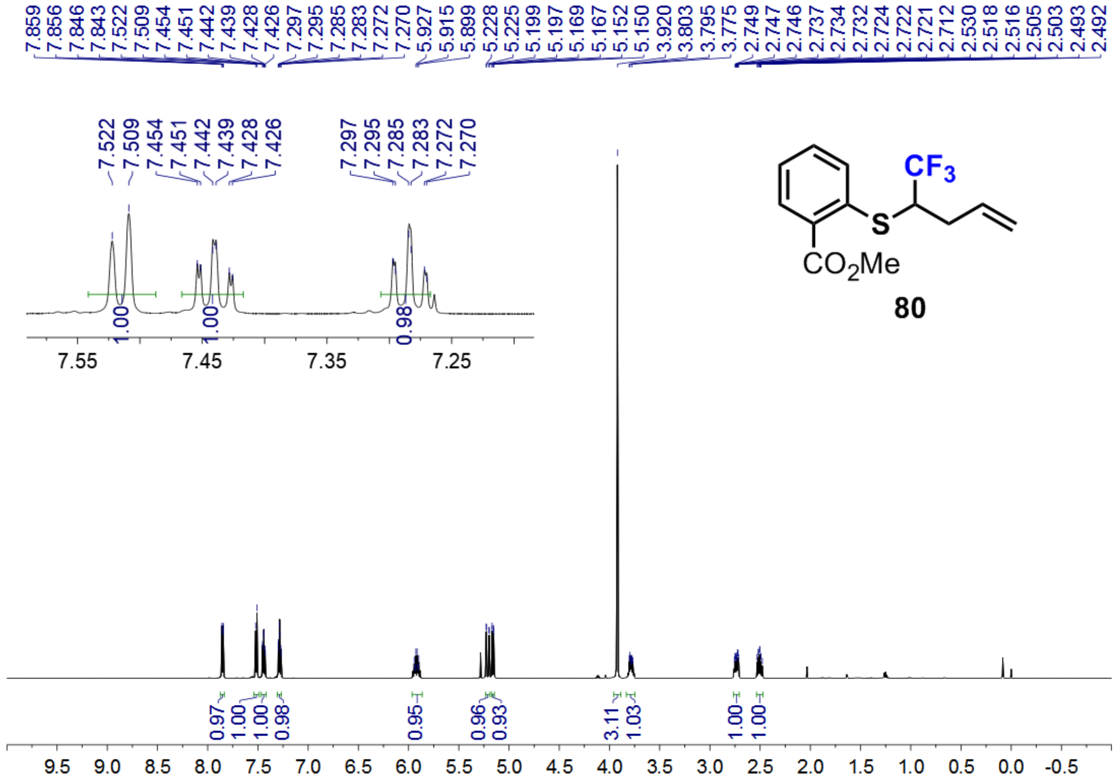

Supplementary Figure 234. ^{1}H NMR of 80

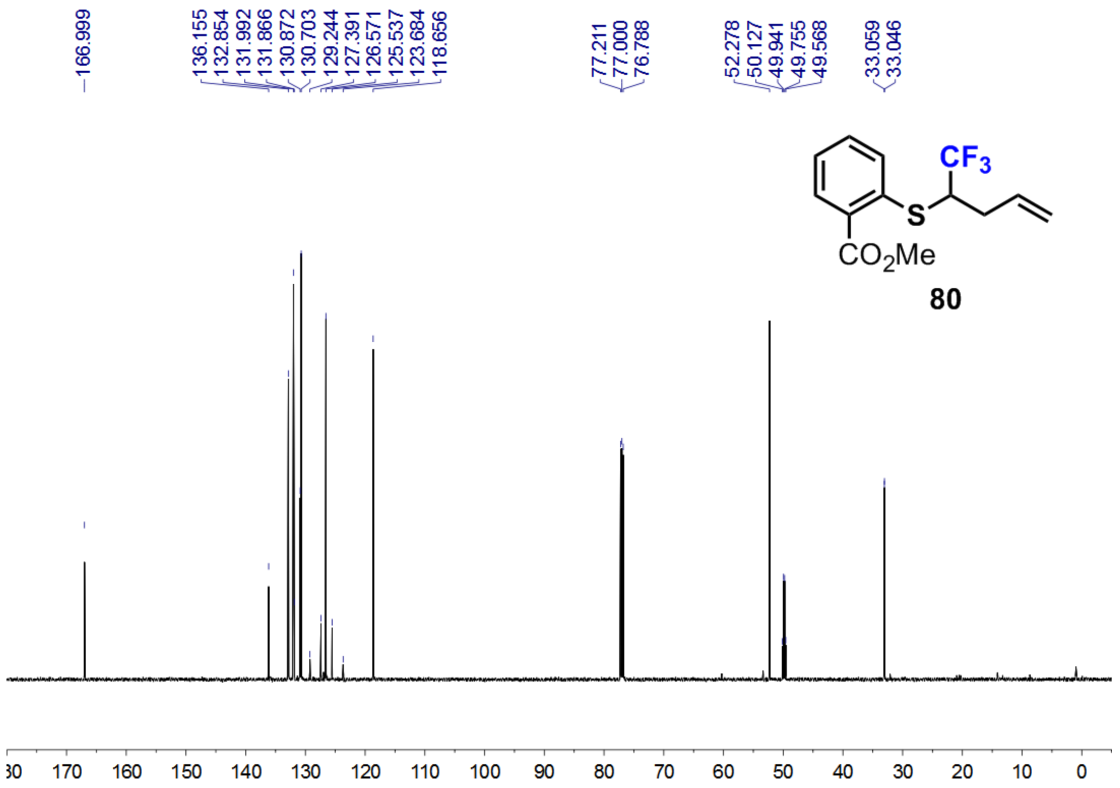

Supplementary Figure 235. ^{13}C NMR of 80

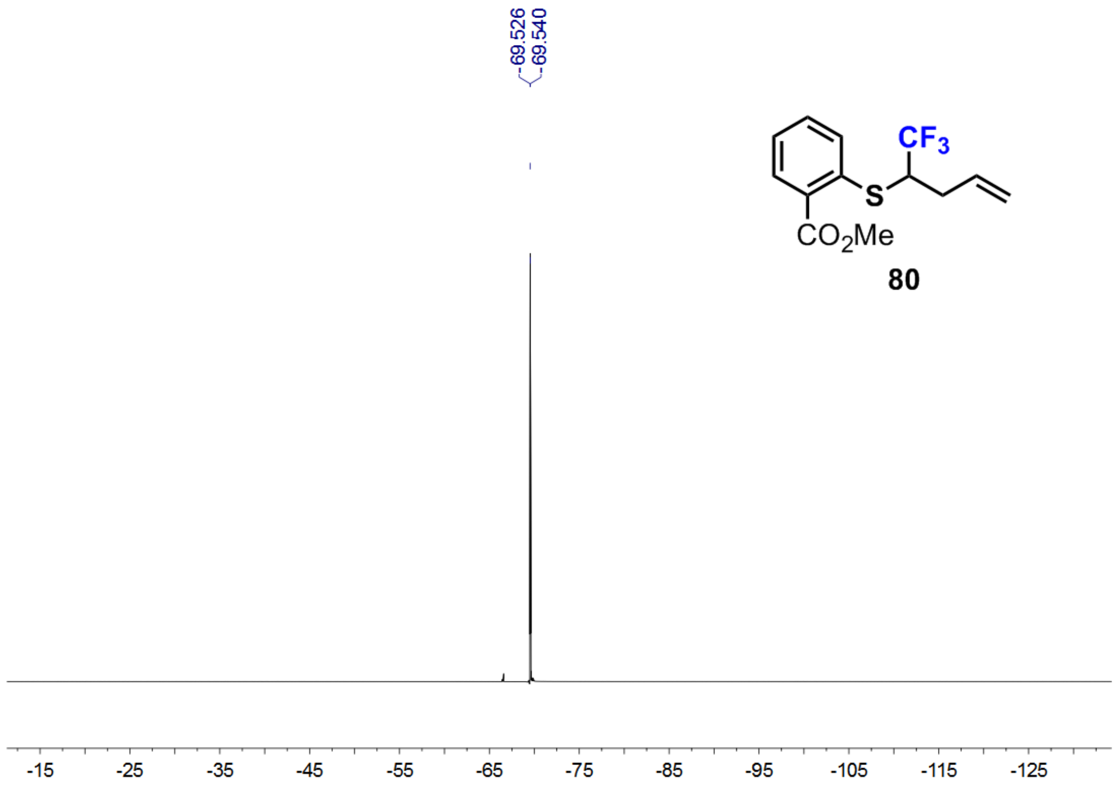

Supplementary Figure 236. ^{19}F NMR of 80

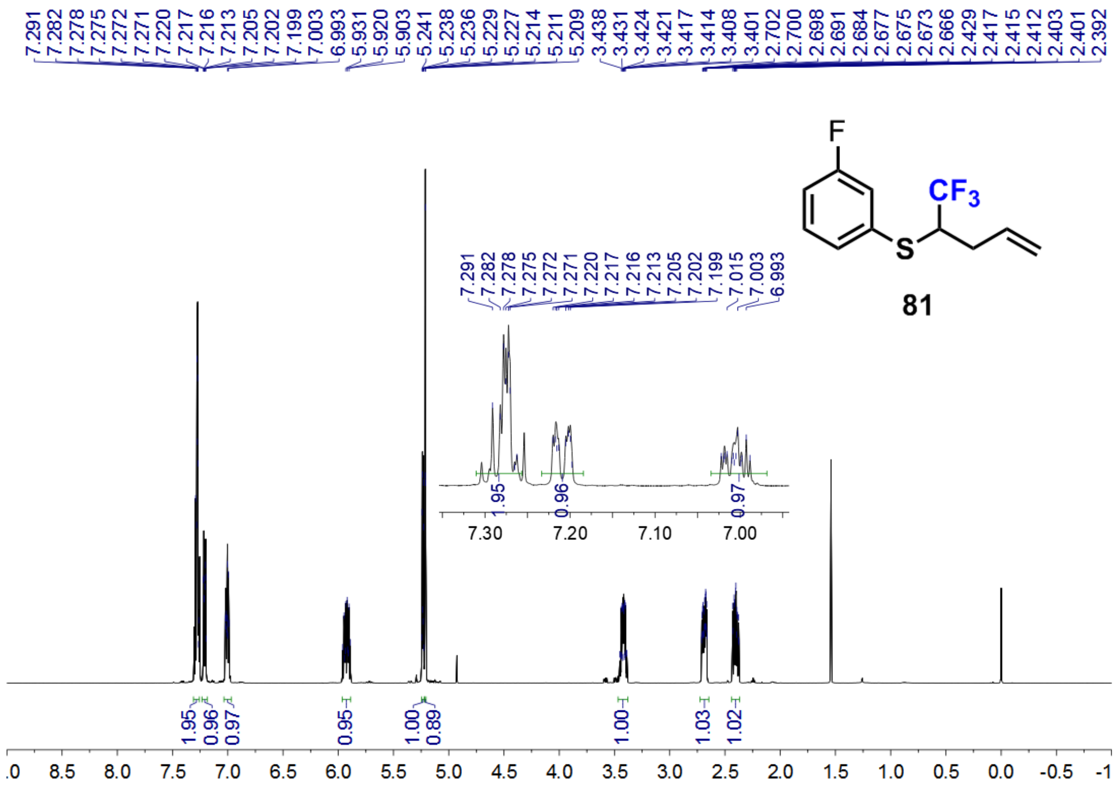

Supplementary Figure 237. ^{1}H NMR of 81

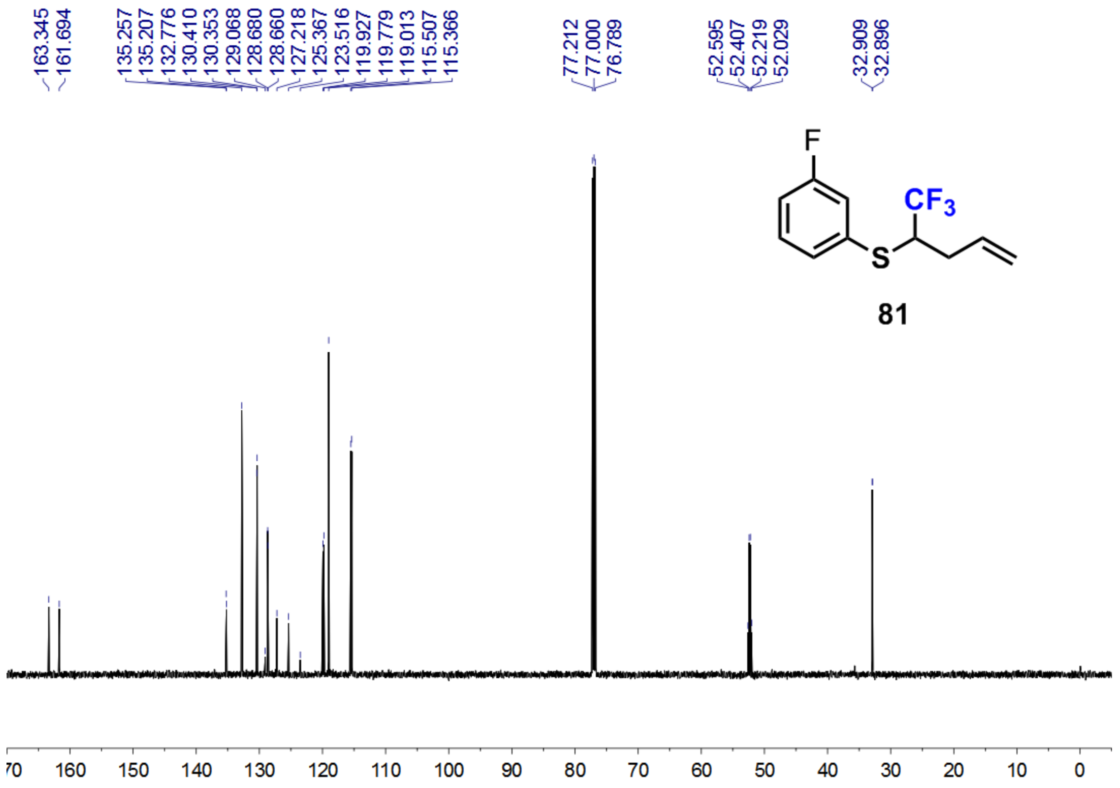

Supplementary Figure 238. ^{13}C NMR of 81

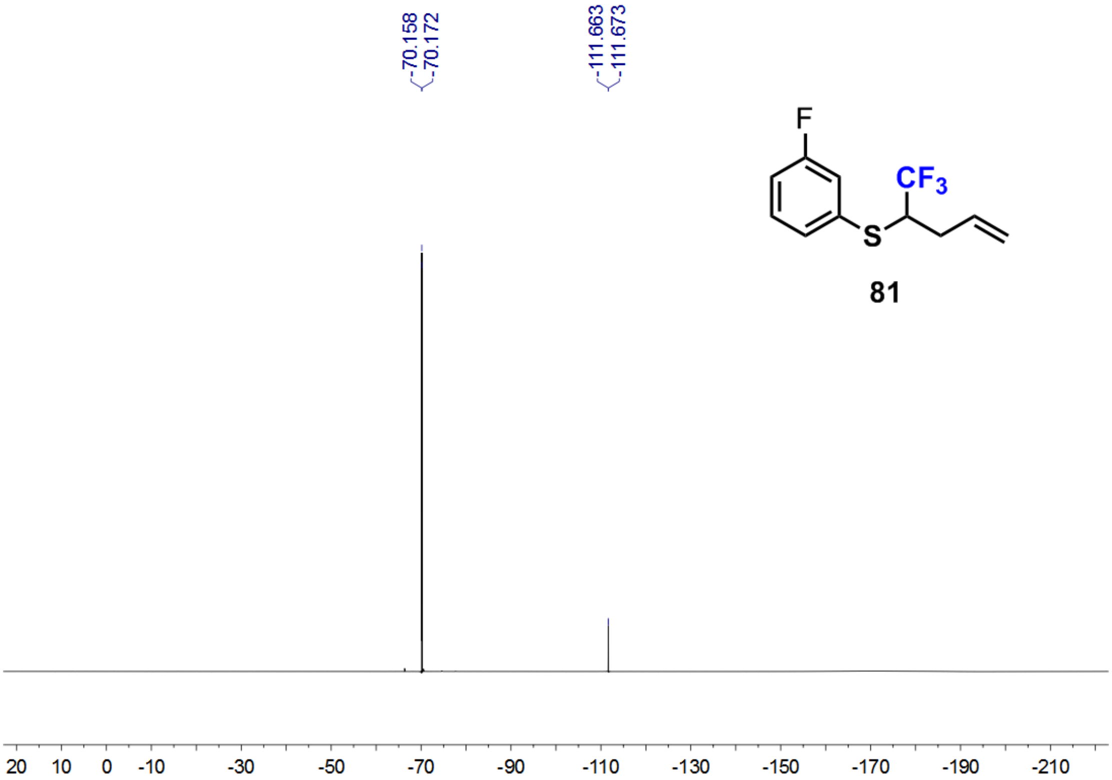

Supplementary Figure 239. ^{19}F NMR of 81

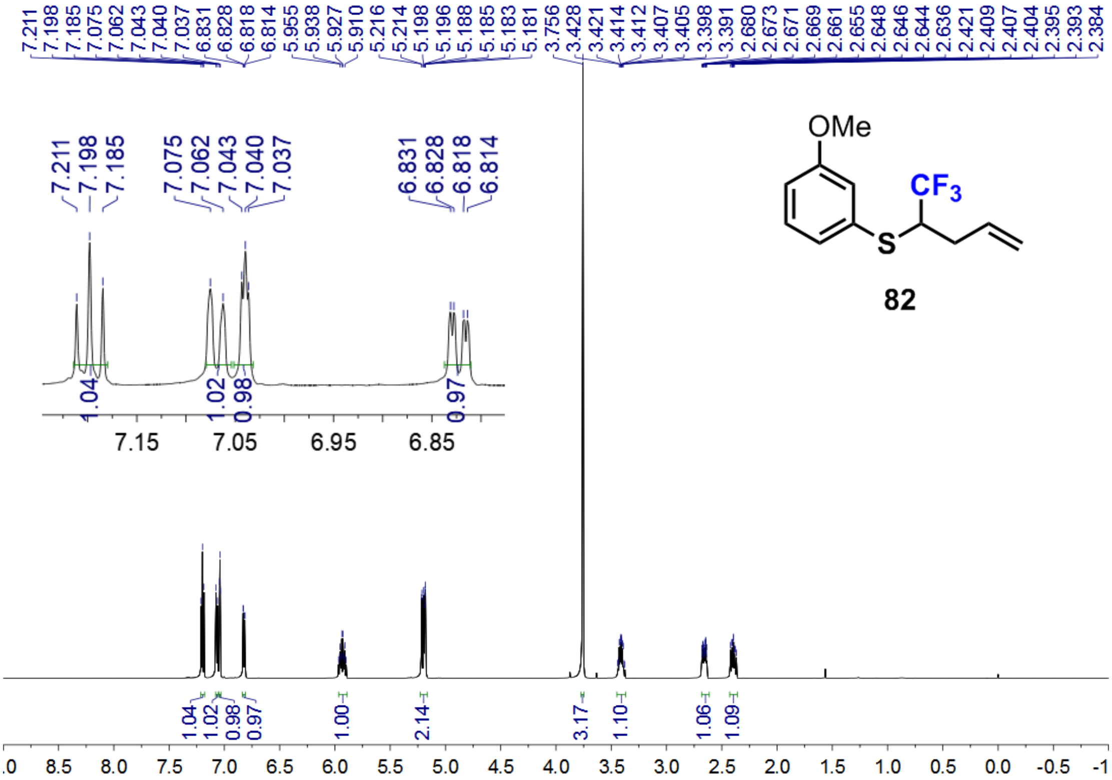

Supplementary Figure 240. ^{1}H NMR of 82

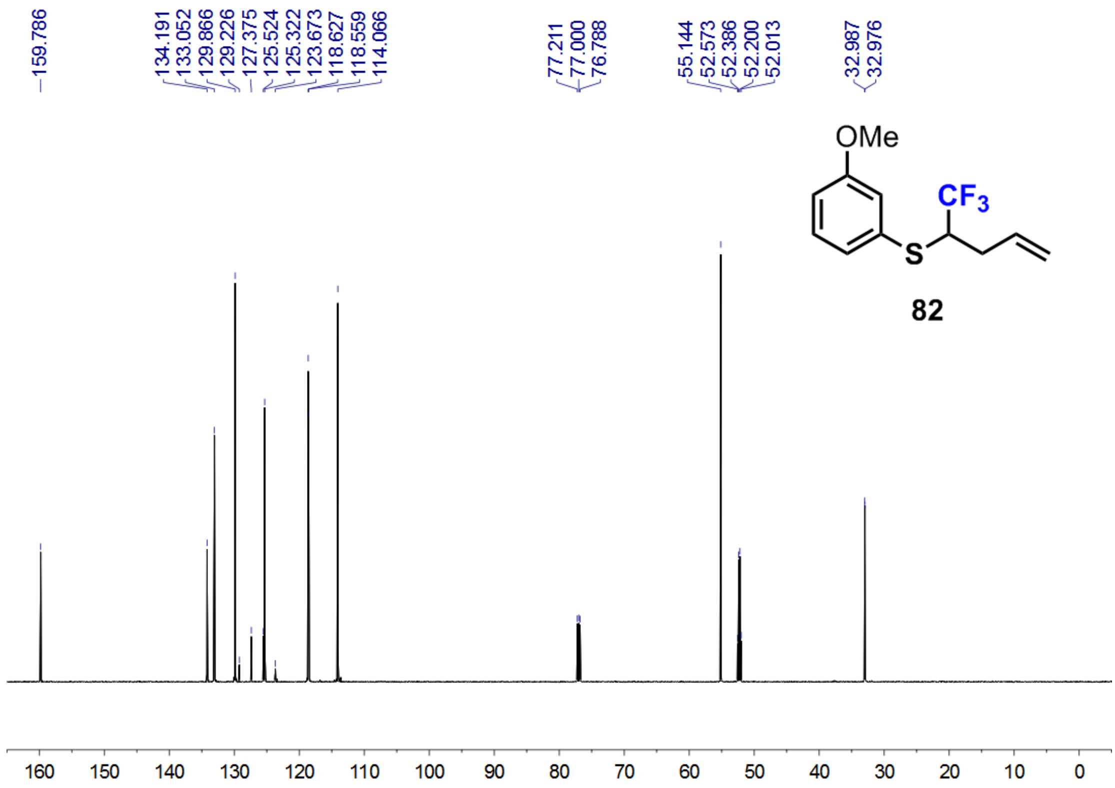

Supplementary Figure 241. ^{13}C NMR of 82

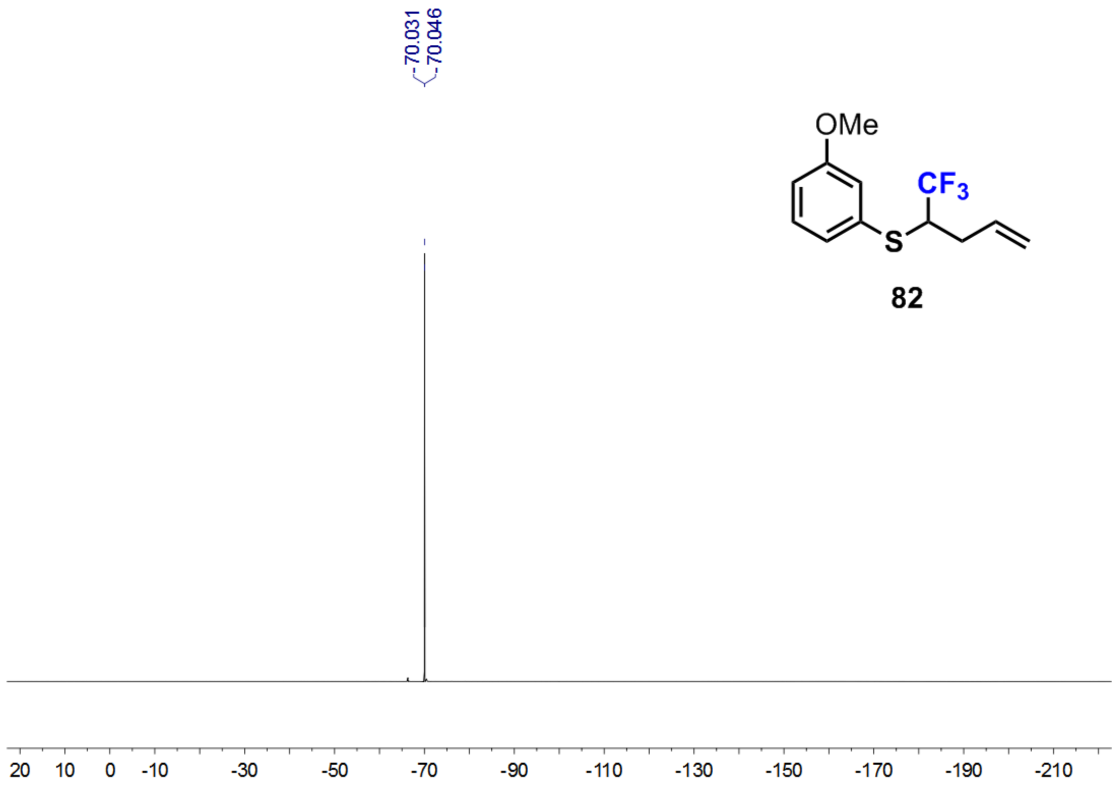

Supplementary Figure 242. ^{19}F NMR of 82

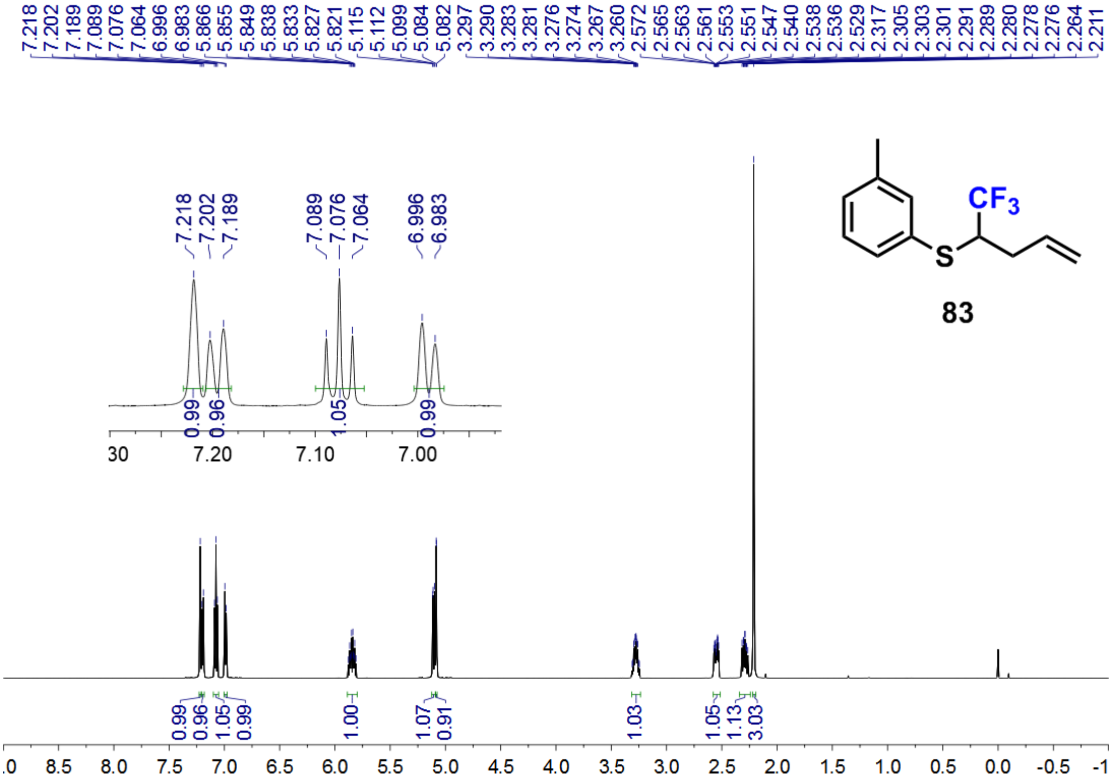

Supplementary Figure 243. ^{1}H NMR of 83

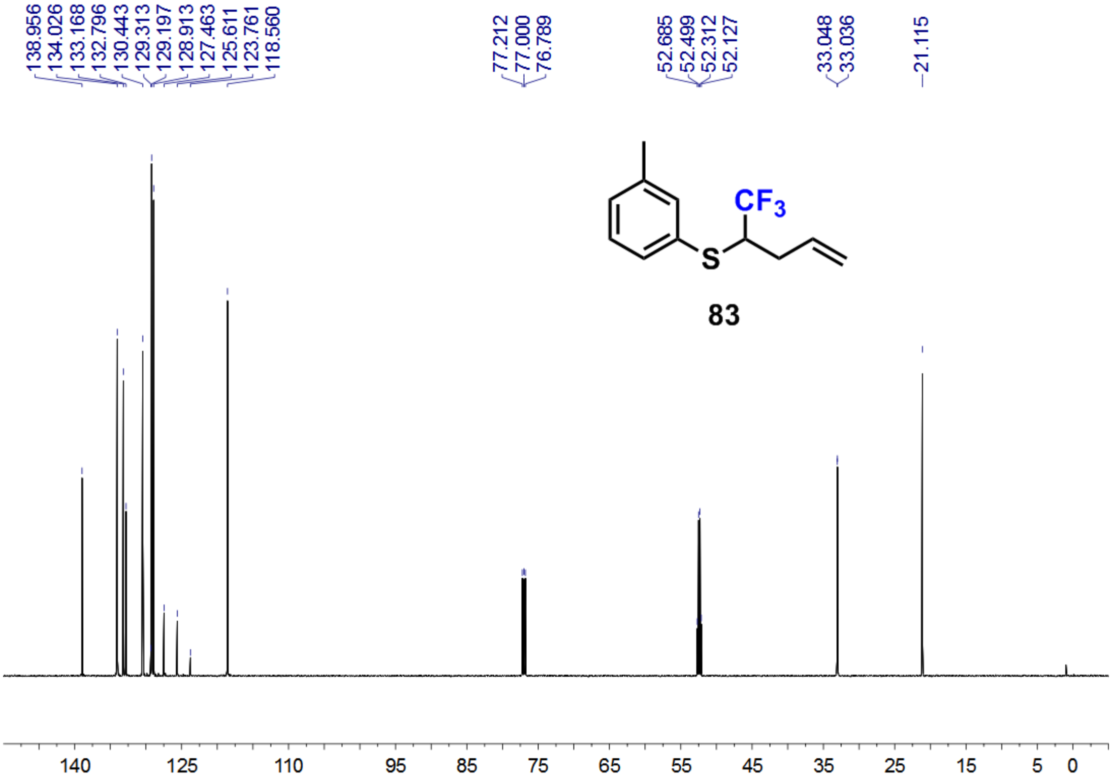

Supplementary Figure 244. ^{13}C NMR of 83

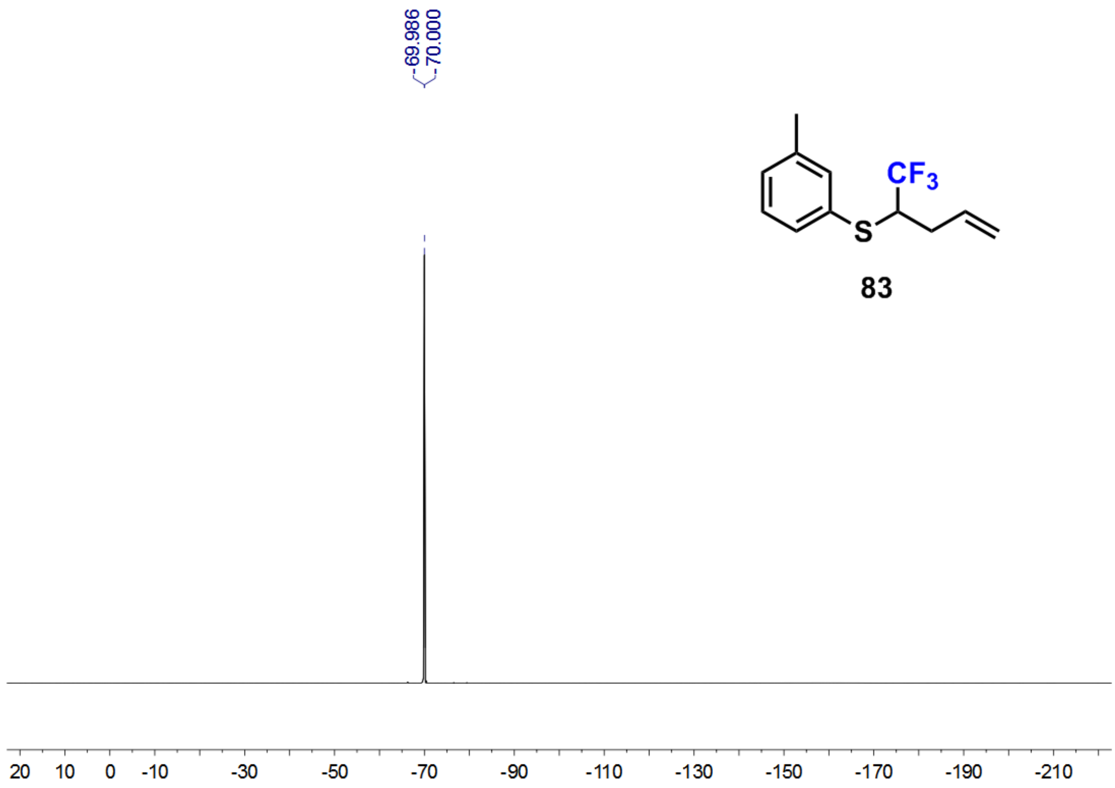

Supplementary Figure 245. ^{19}F NMR of 83

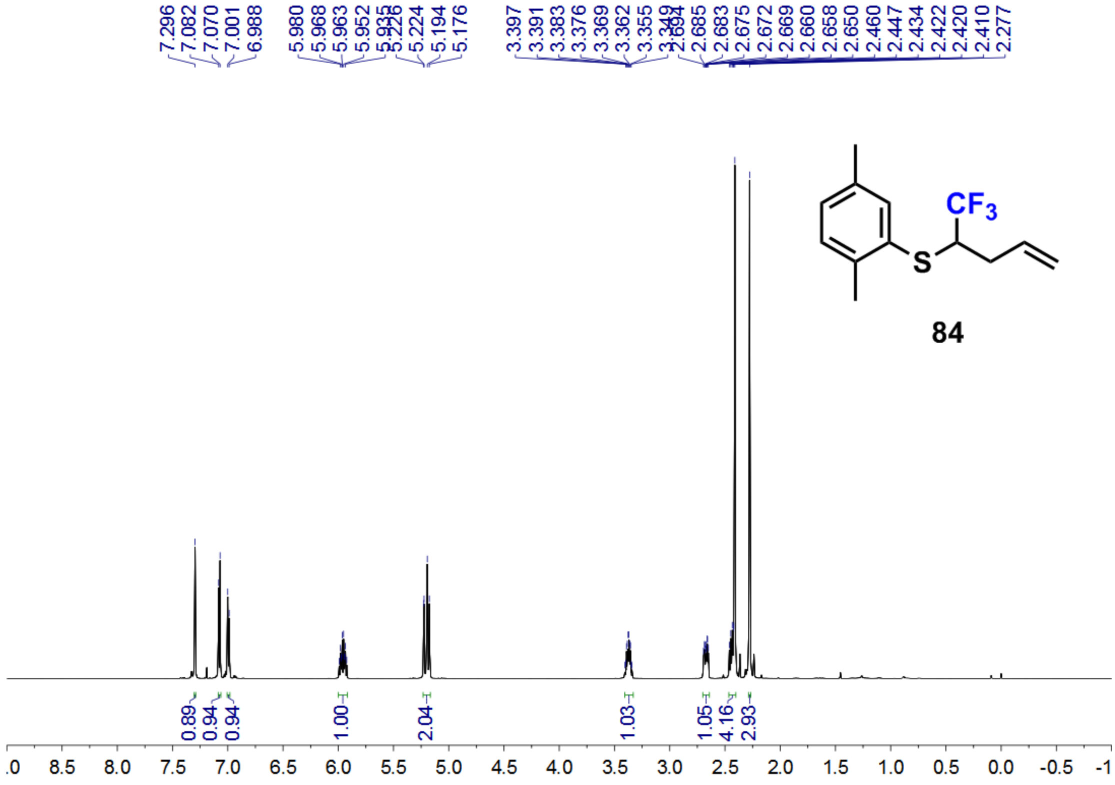

Supplementary Figure 246. ^{1}H NMR of 84

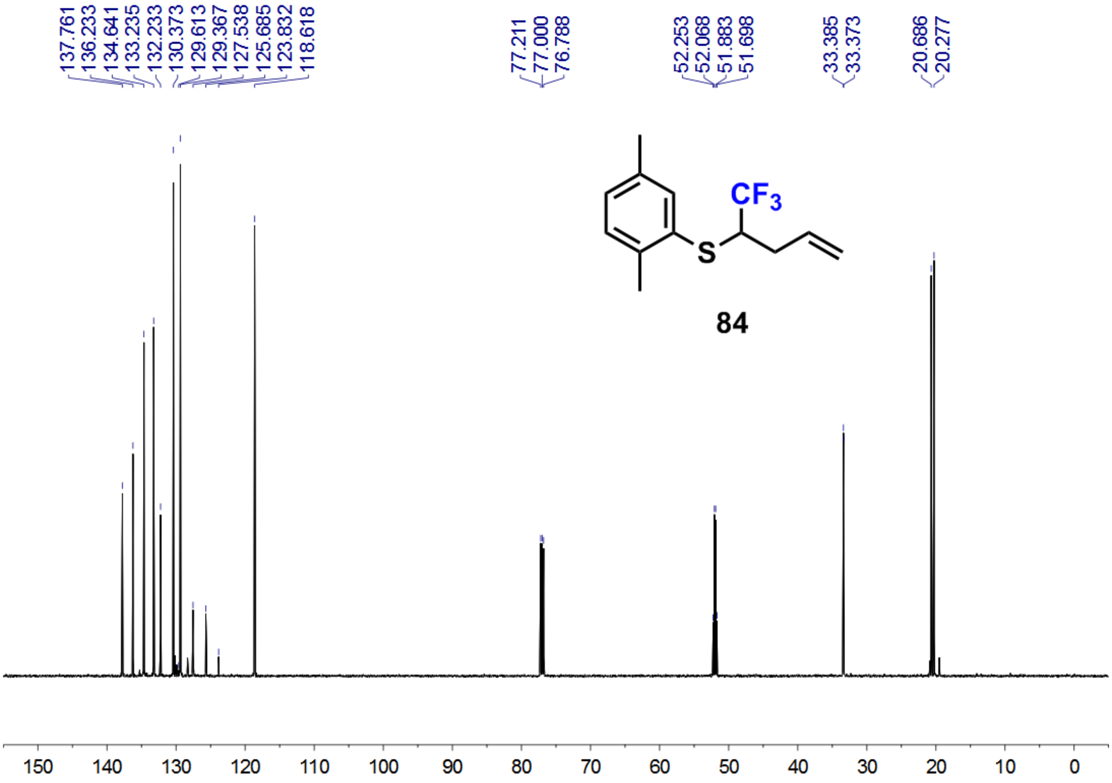

Supplementary Figure 247. ^{13}C NMR of 84

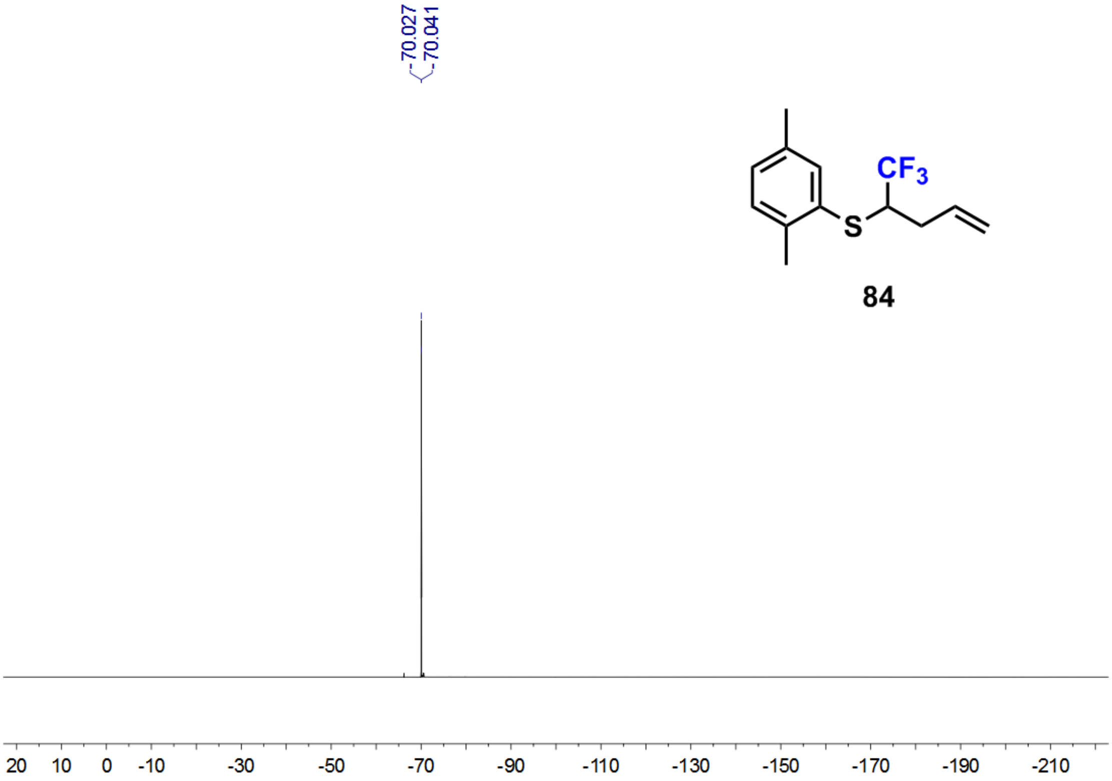

Supplementary Figure 248. ^{19}F NMR of 84

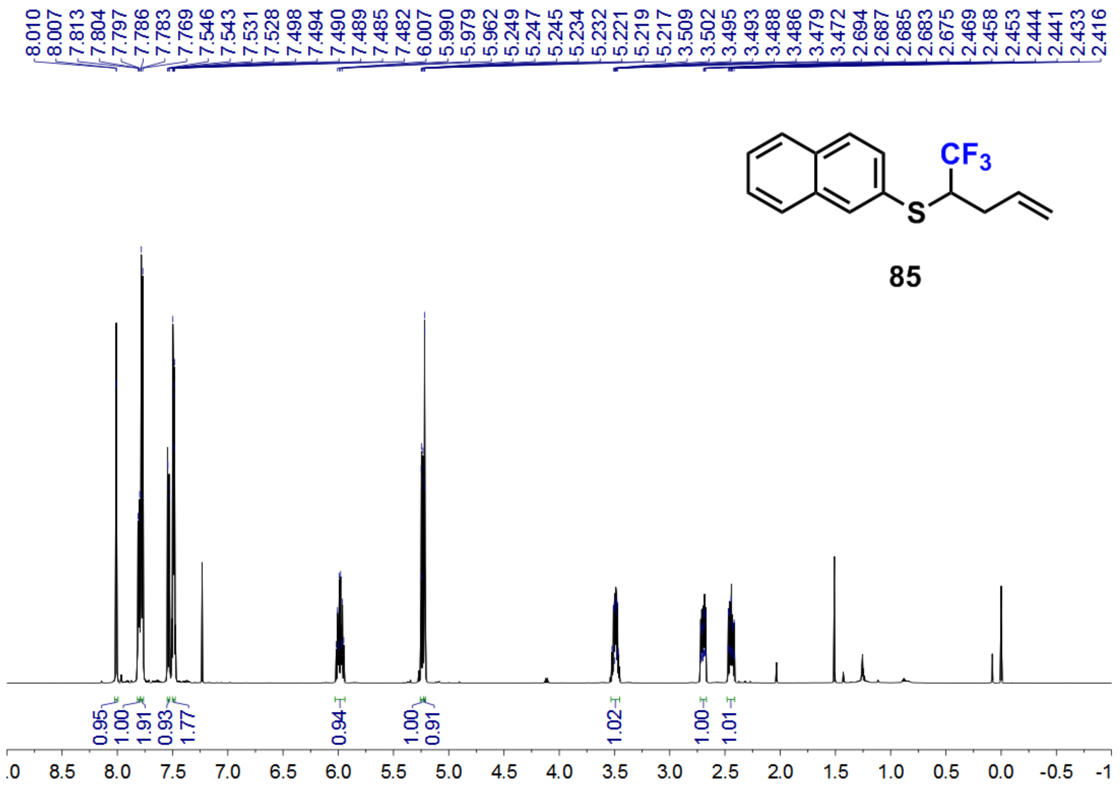

Supplementary Figure 249. ^{1}H NMR of 85

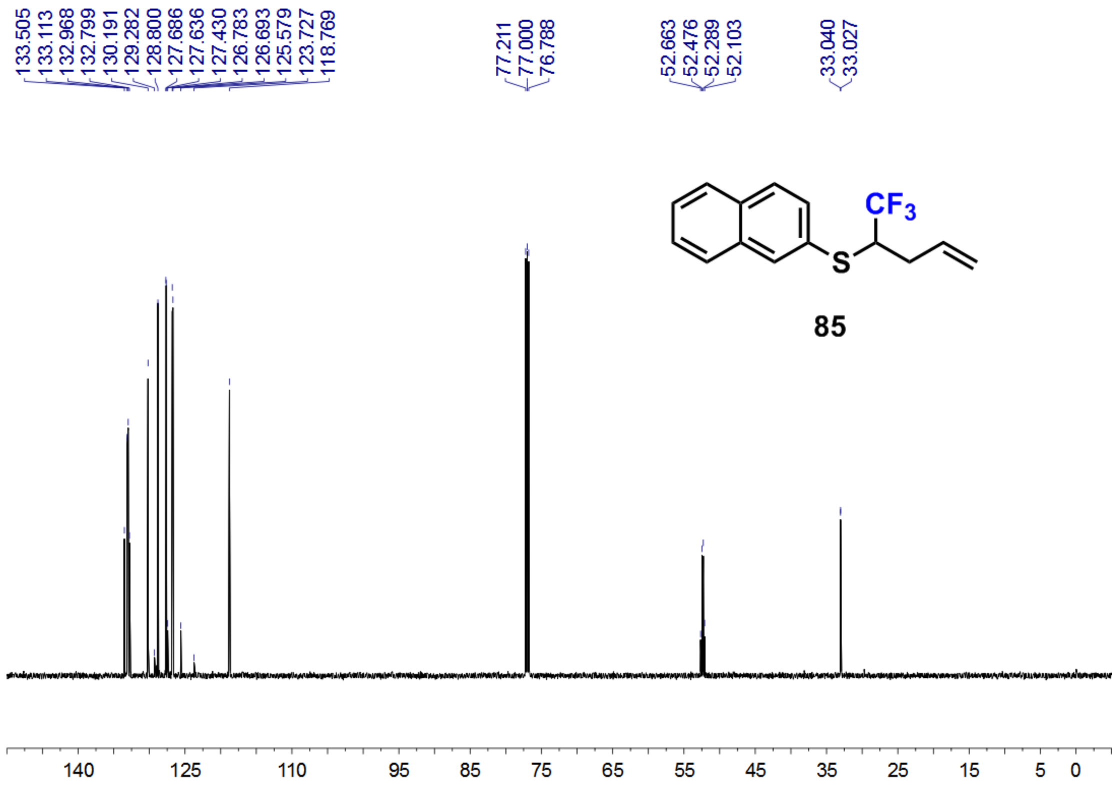

Supplementary Figure 250. ^{13}C NMR of 85

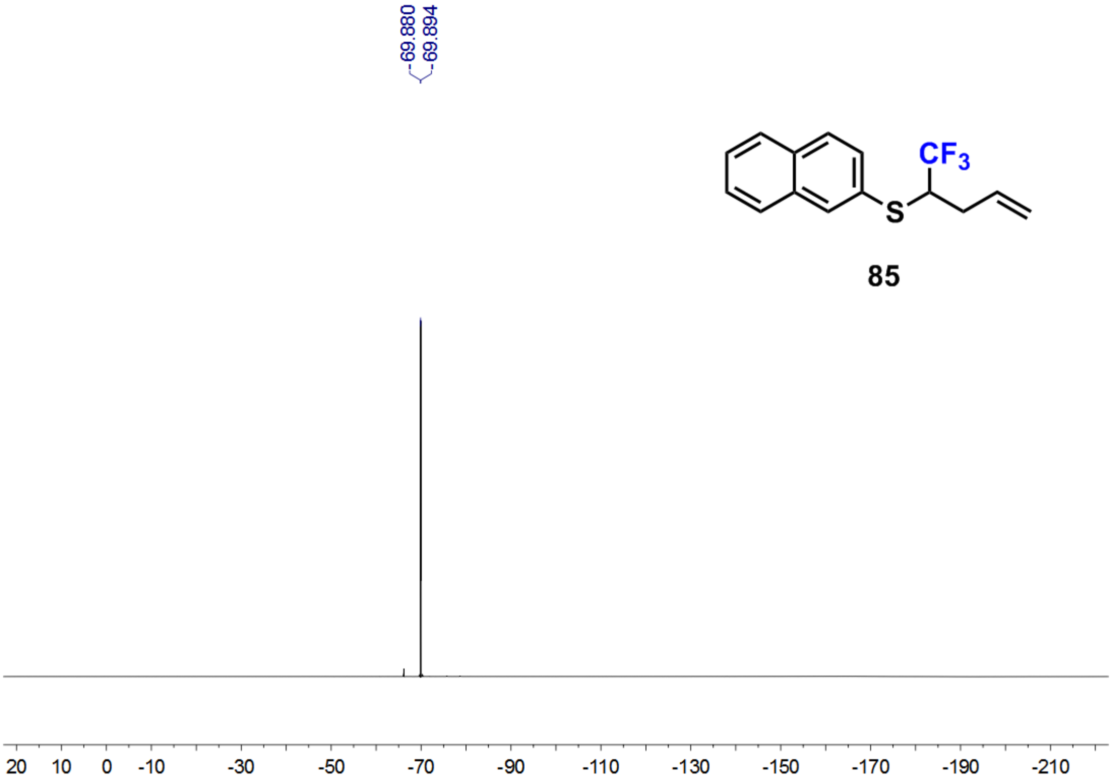

Supplementary Figure 251. ^{19}F NMR of 85

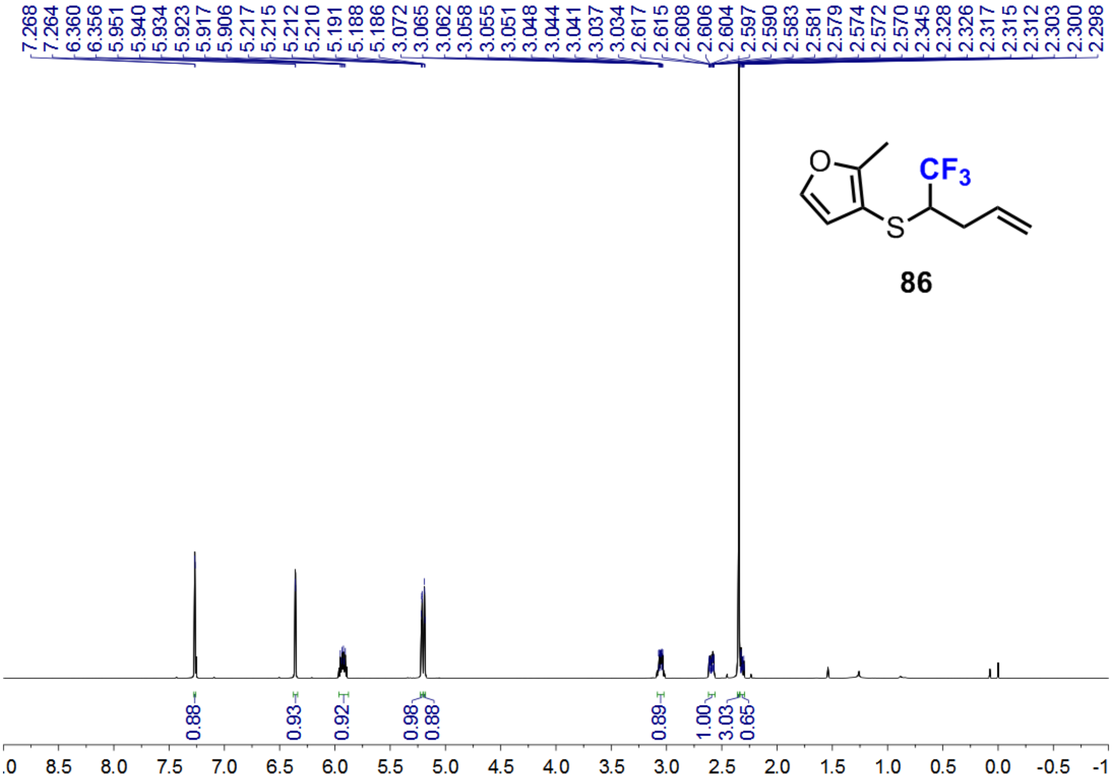

Supplementary Figure 252. ^{1}H NMR of 86

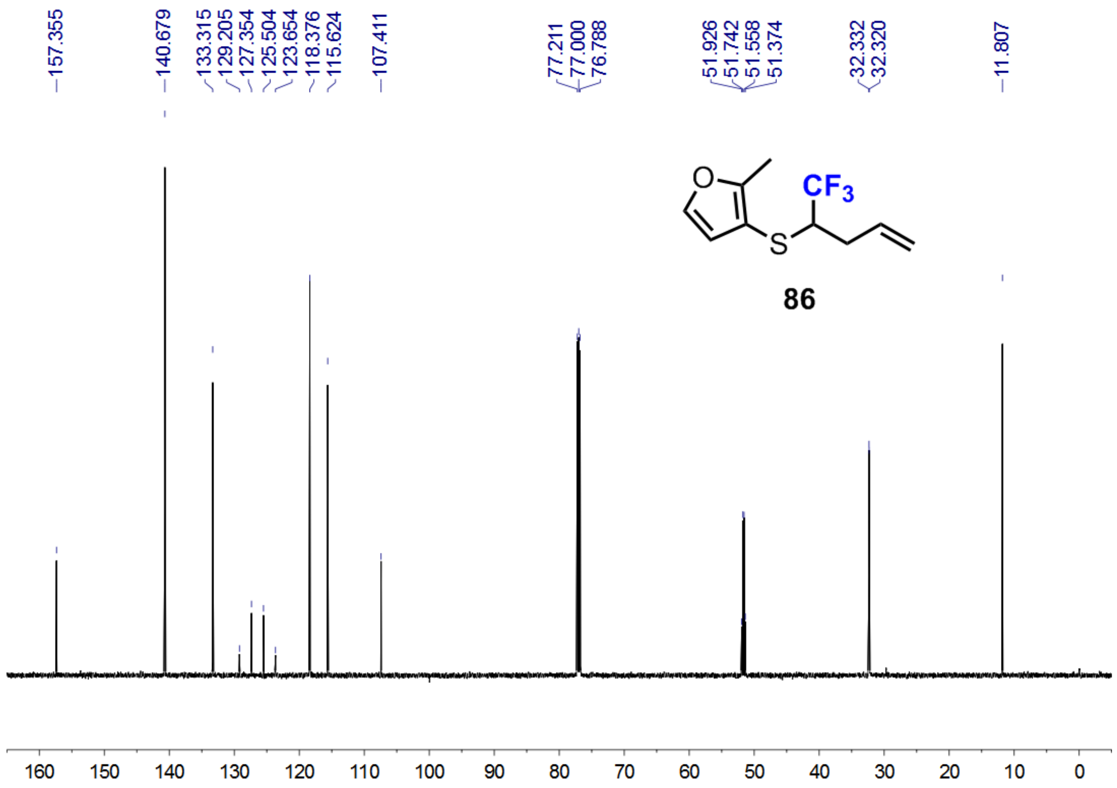

Supplementary Figure 253. ^{13}C NMR of 86

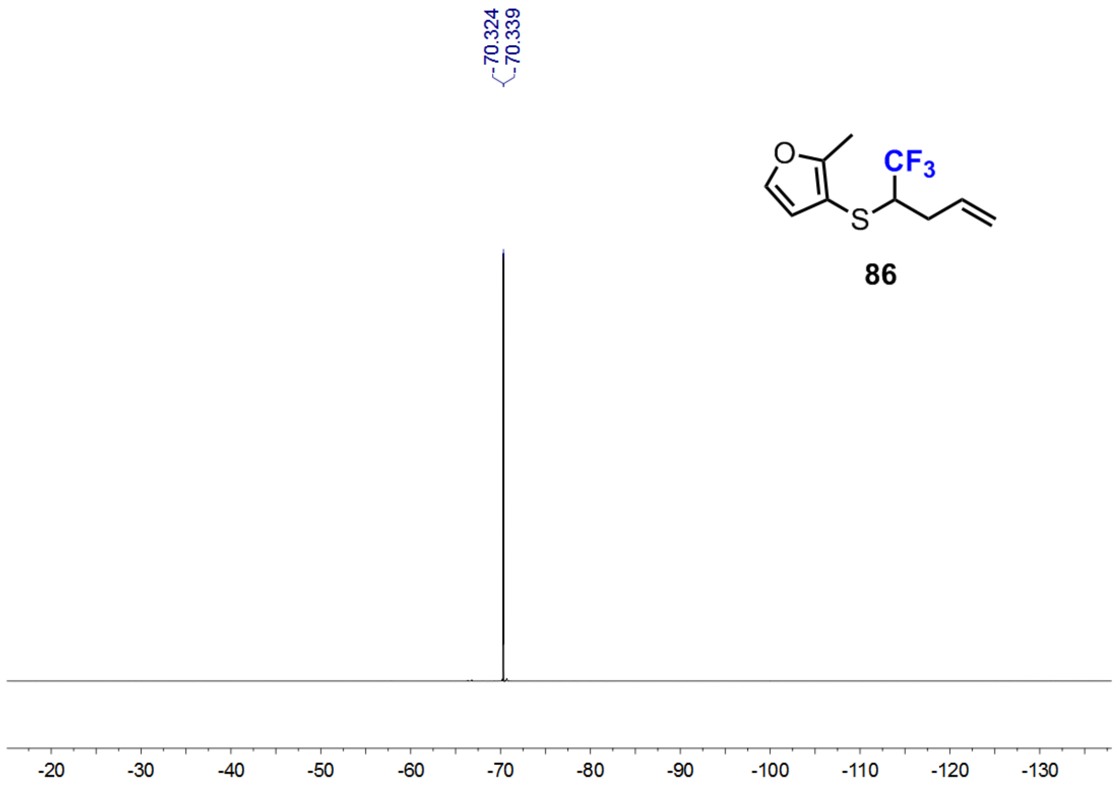

Supplementary Figure 254. ^{19}F NMR of 86

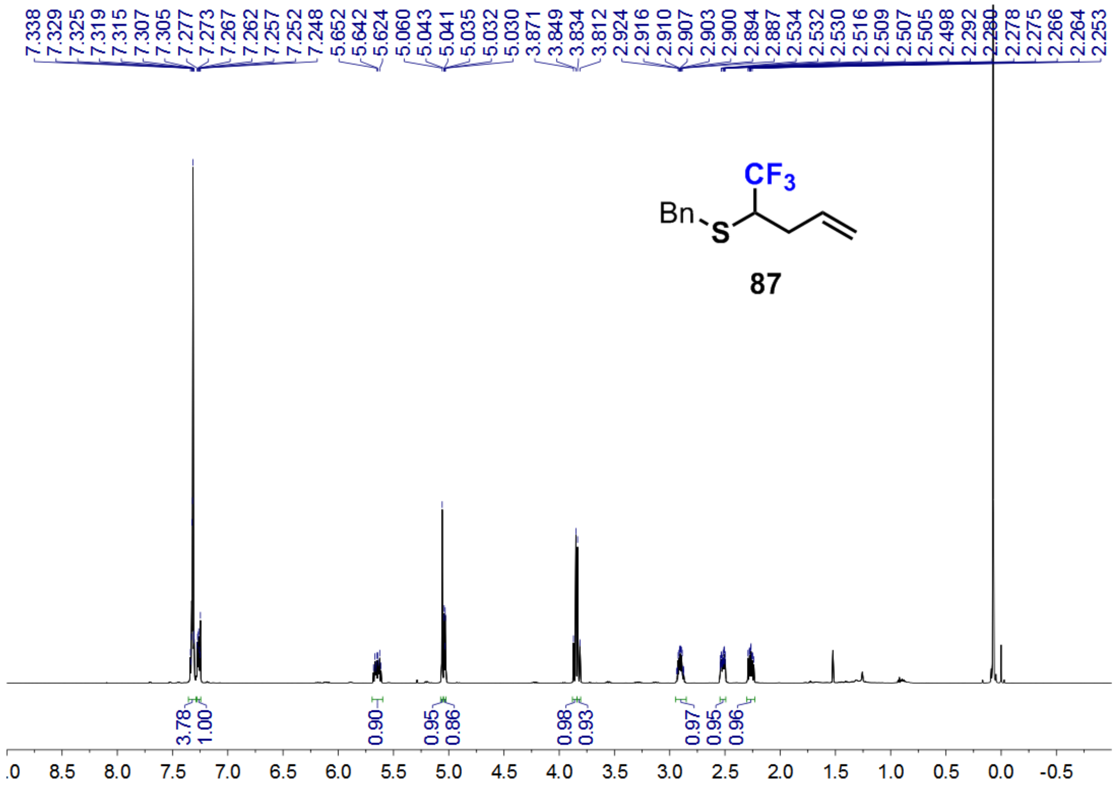

Supplementary Figure 255. ^{1}H NMR of 87

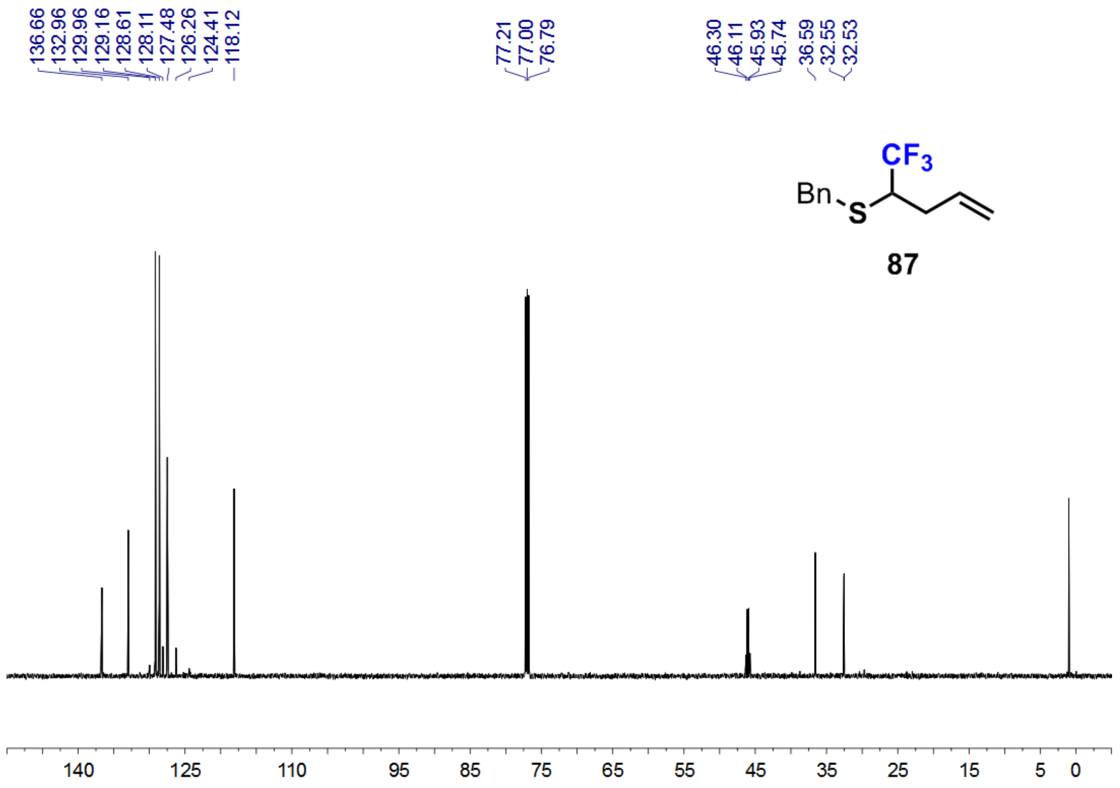

Supplementary Figure 256. ^{13}C NMR of 87

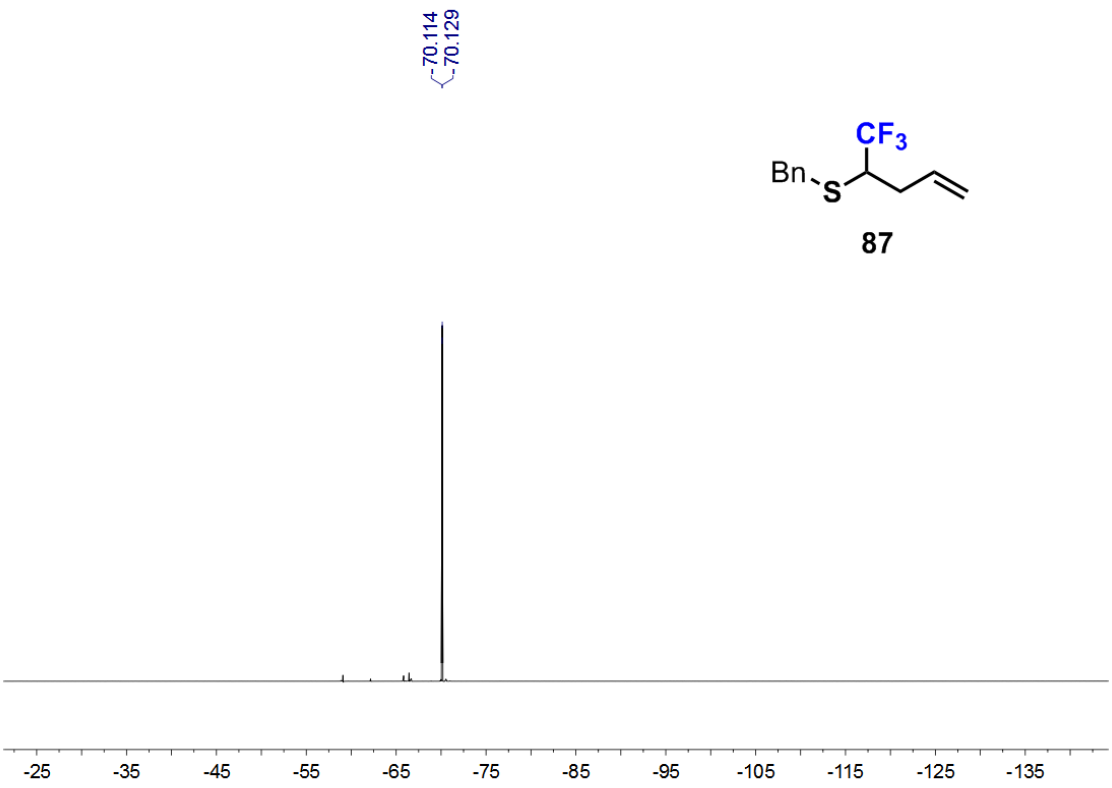

Supplementary Figure 257. ^{19}F NMR of 87

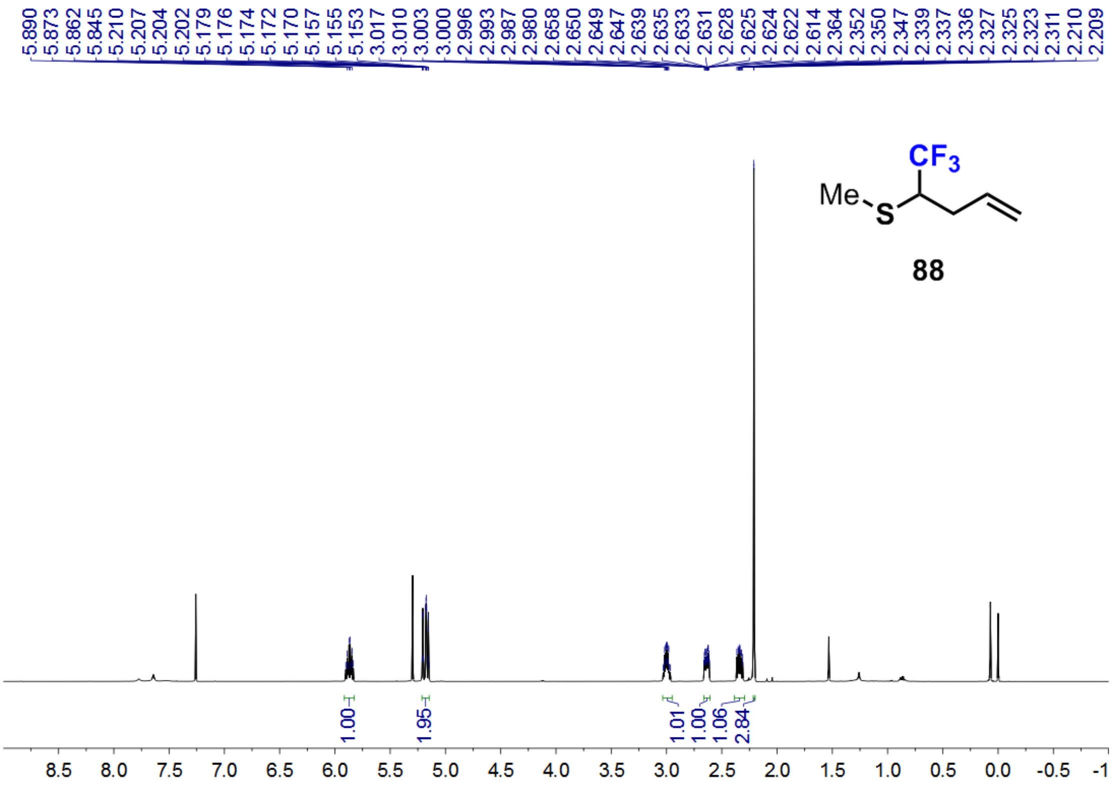

Supplementary Figure 258. ^{1}H NMR of 88

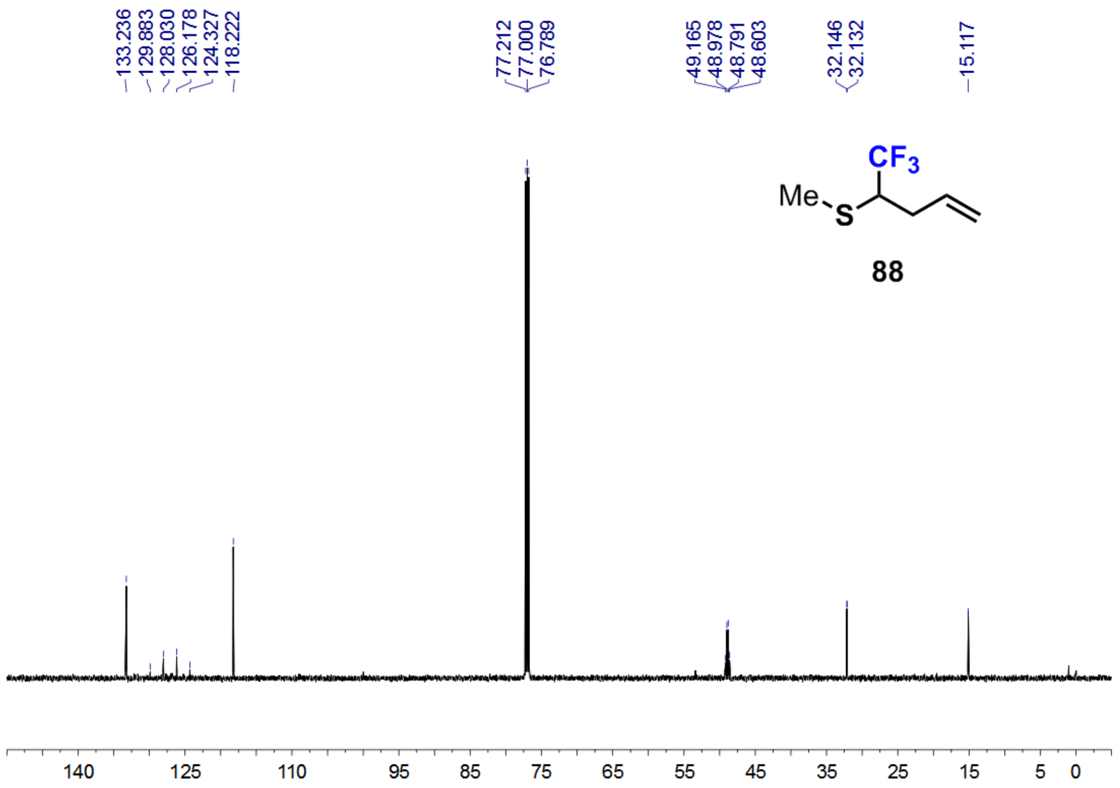

Supplementary Figure 259. ^{13}C NMR of 88

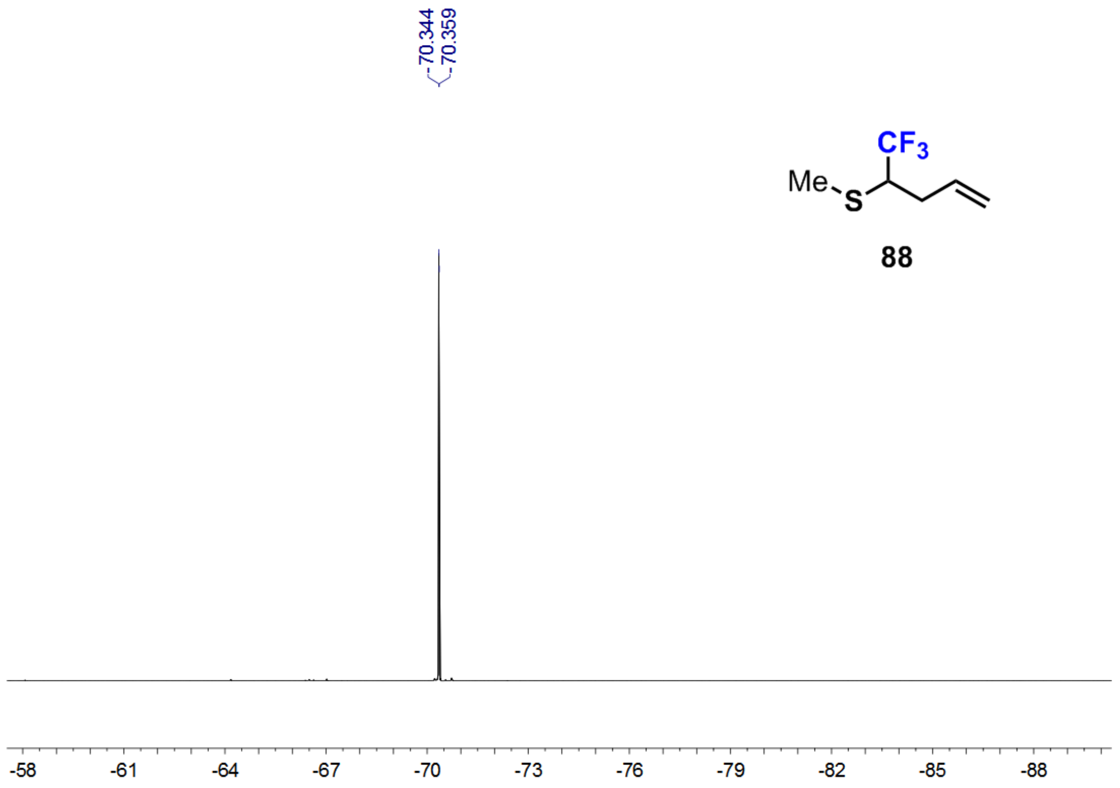

Supplementary Figure 260. ^{19}F NMR of 88

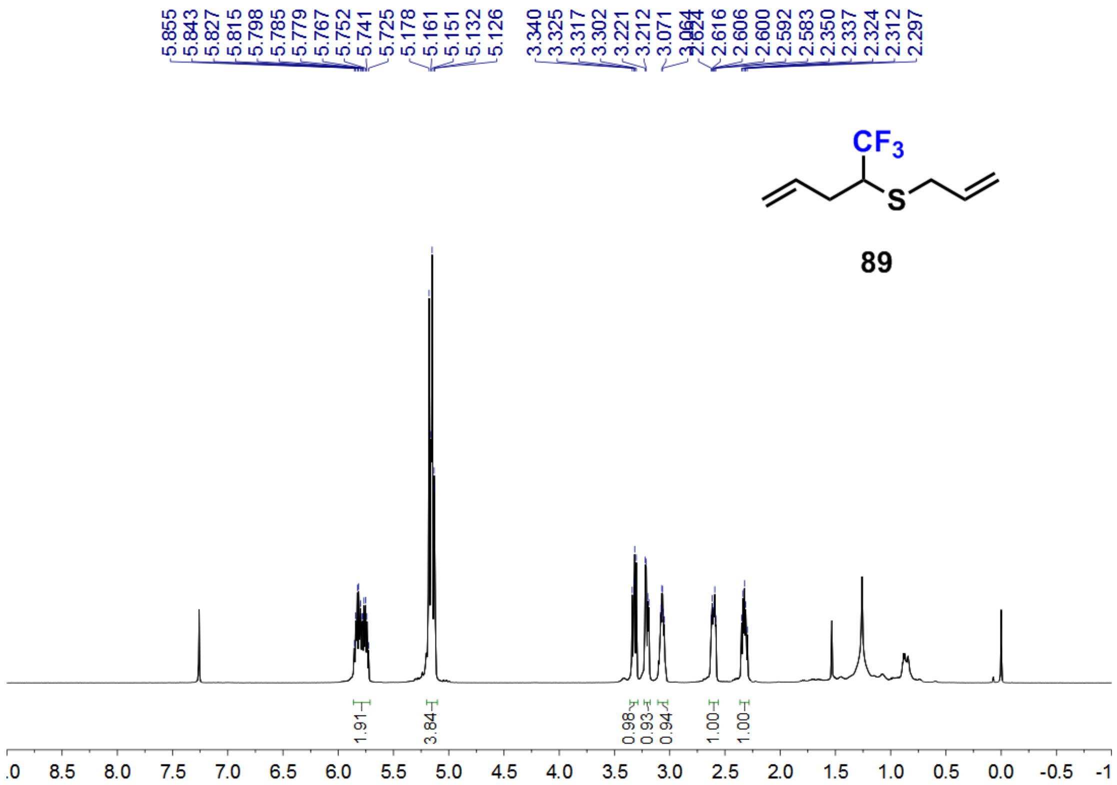

Supplementary Figure 261. ^{1}H NMR of 89

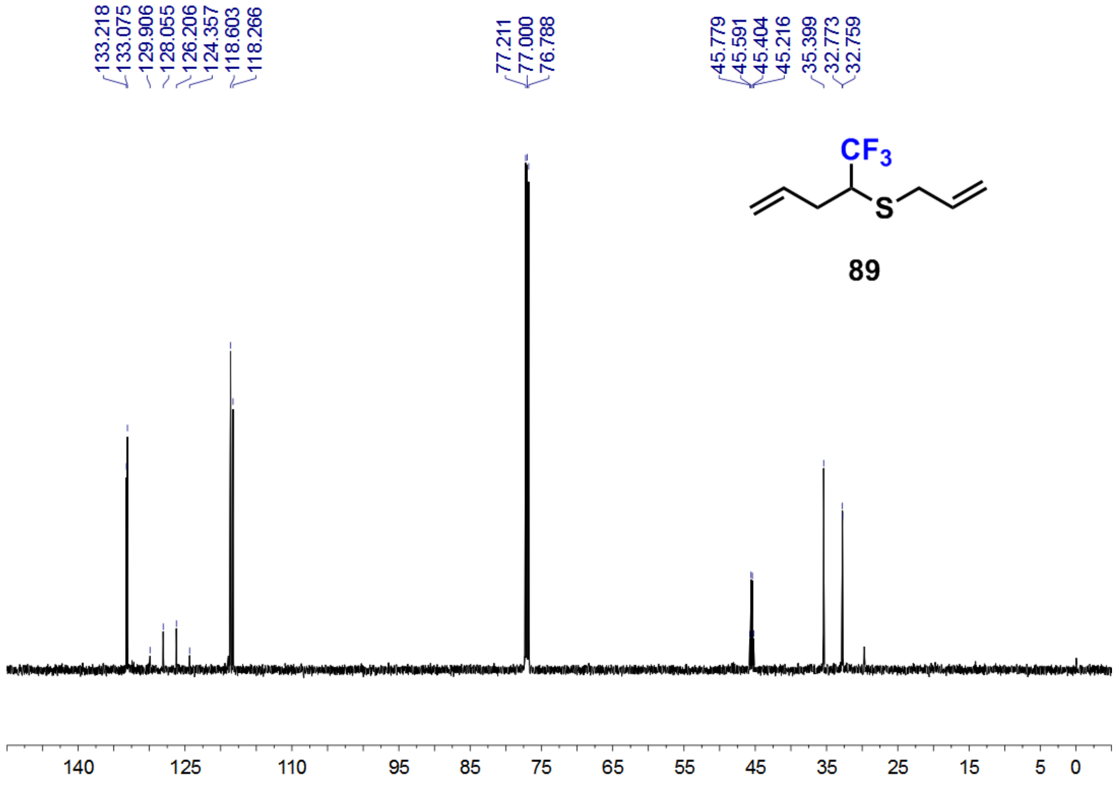

Supplementary Figure 262. ^{13}C NMR of 89

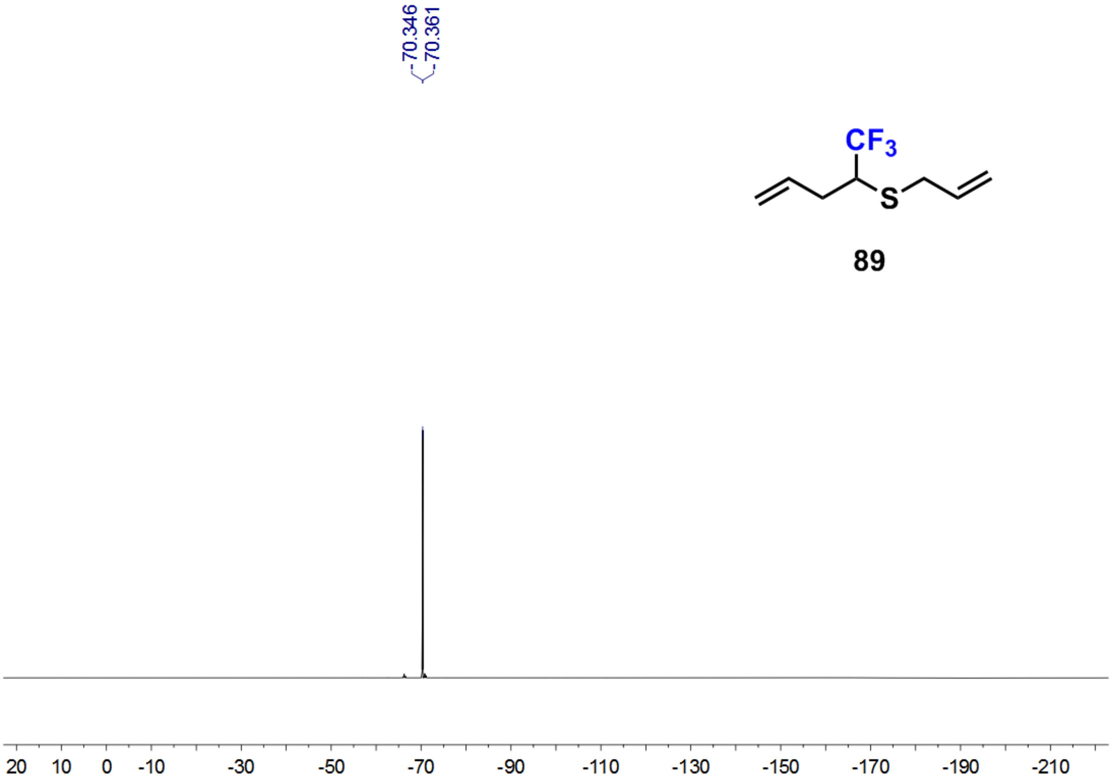

Supplementary Figure 263. ^{19}F NMR of 89

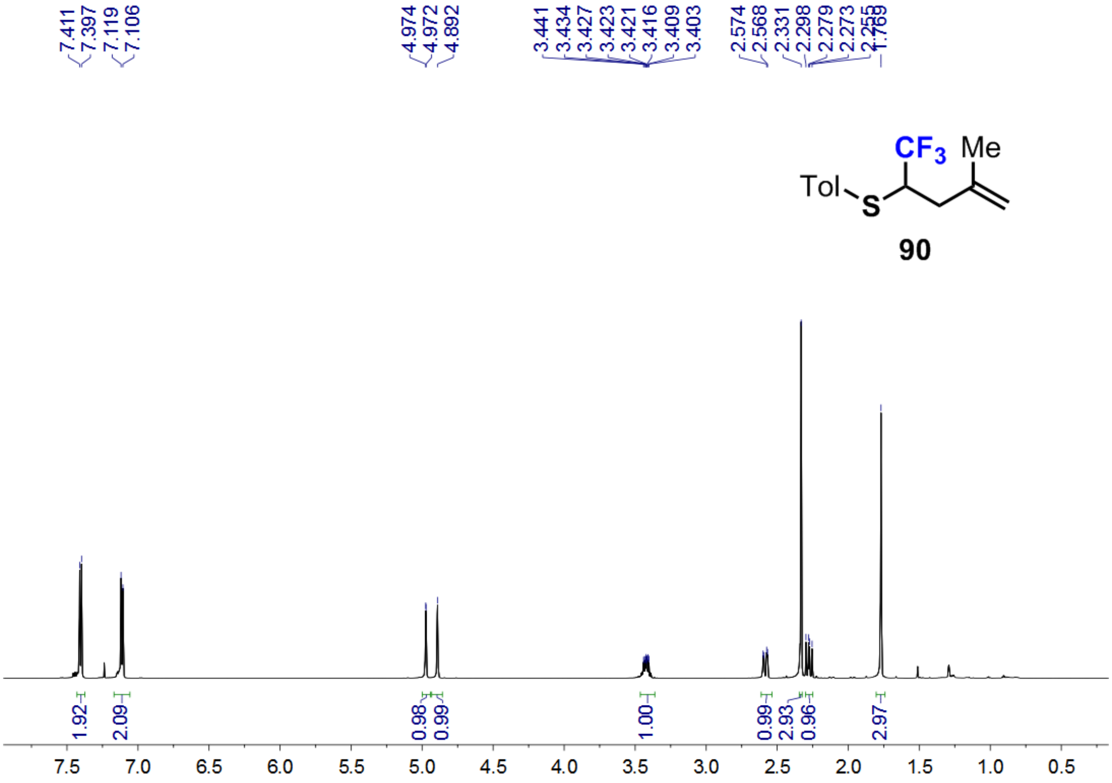

Supplementary Figure 264. ^{1}H NMR of 90

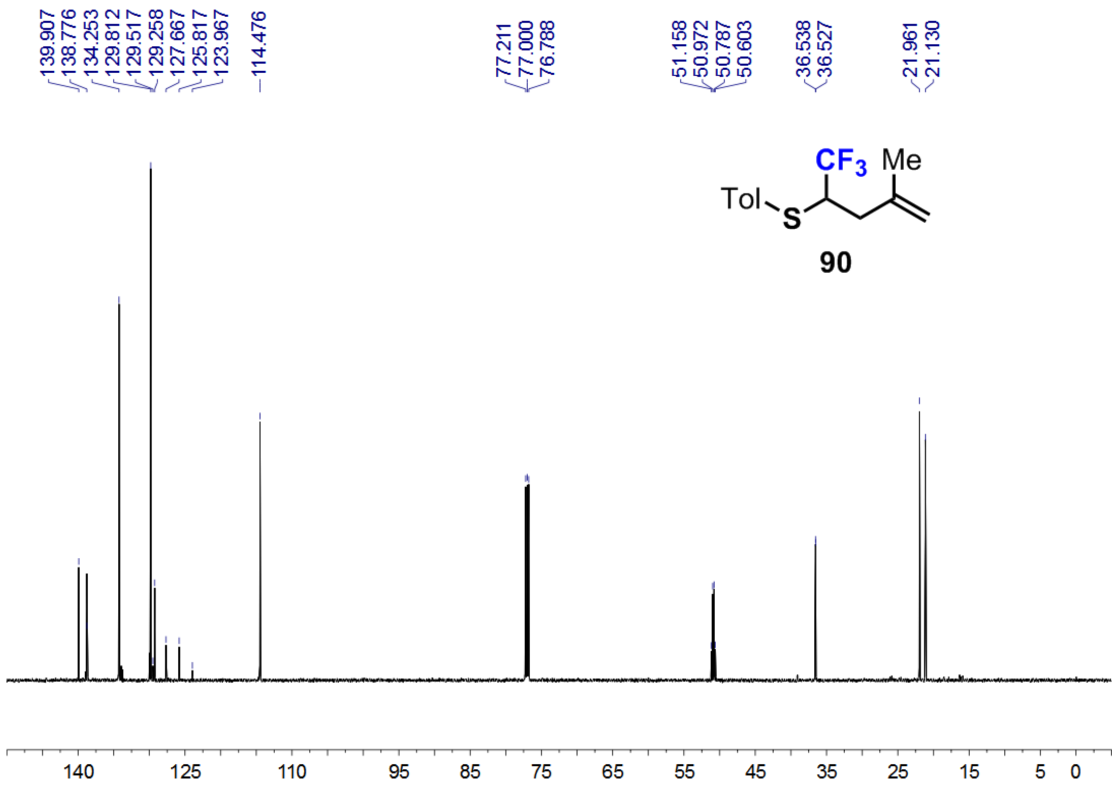

Supplementary Figure 265. ^{13}C NMR of 90

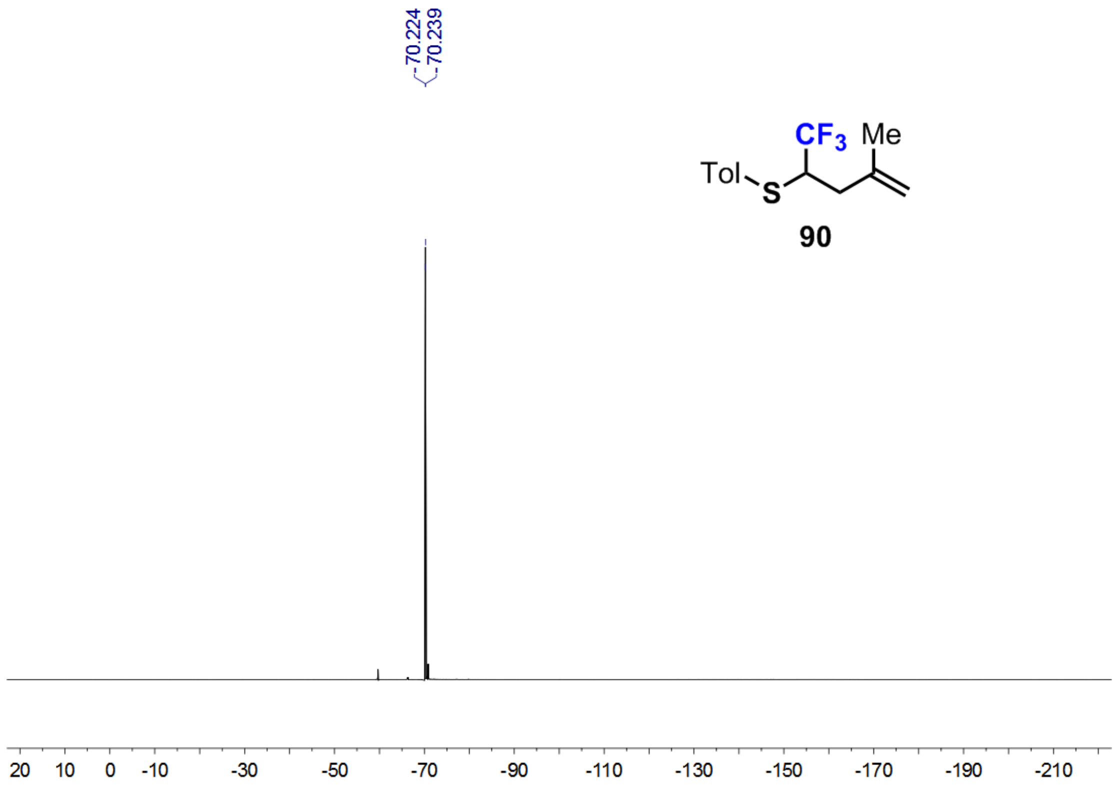

Supplementary Figure 266. ^{19}F NMR of 90

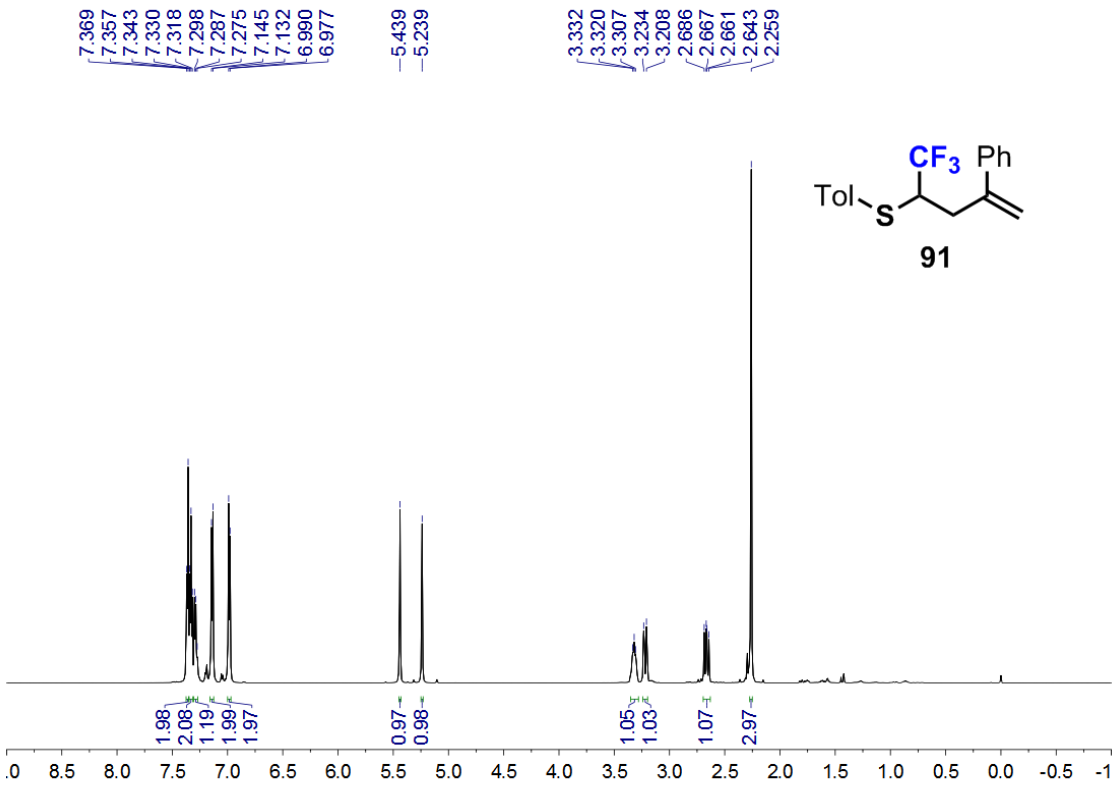

Supplementary Figure 267. ^{1}H NMR of 91

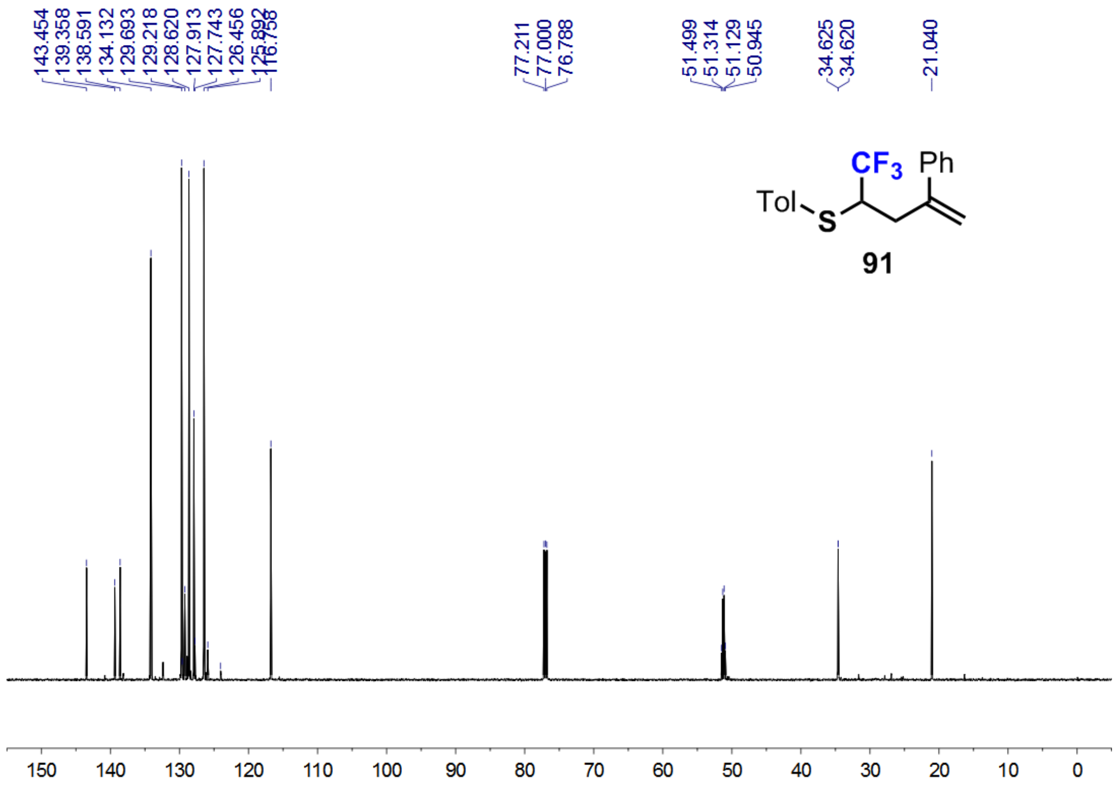

Supplementary Figure 268. ^{13}C NMR of 91

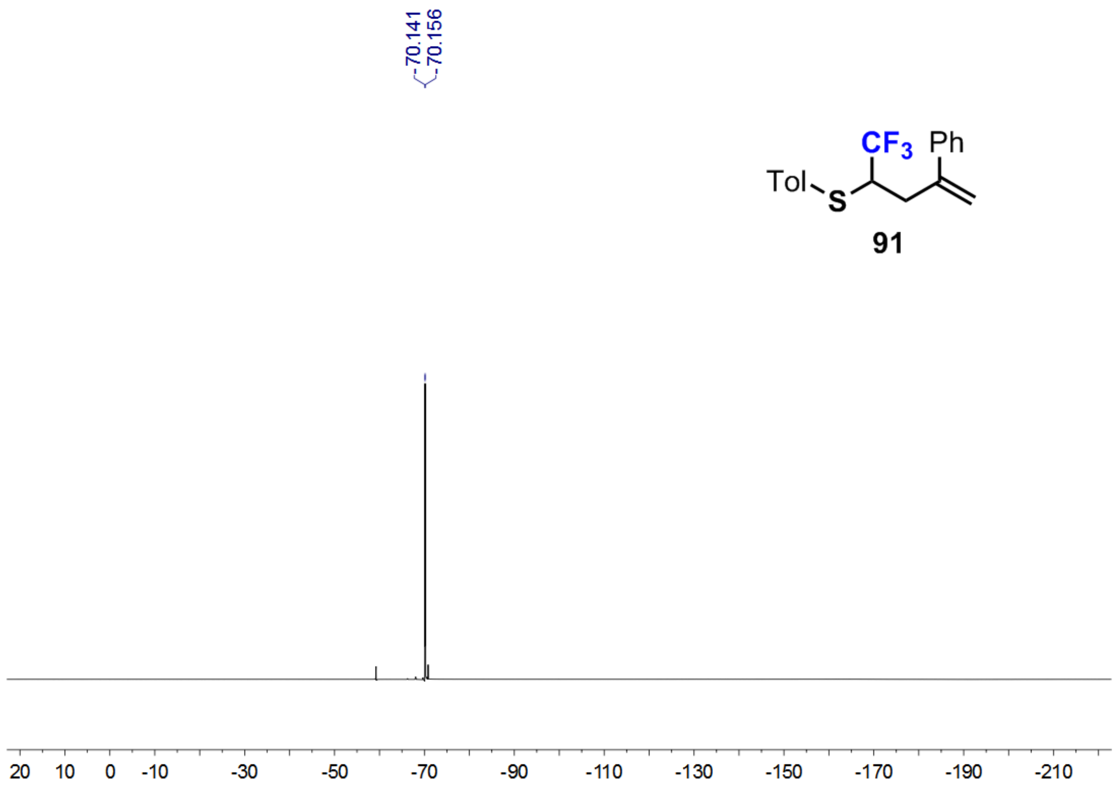

Supplementary Figure 269. [19]C NMR of 91

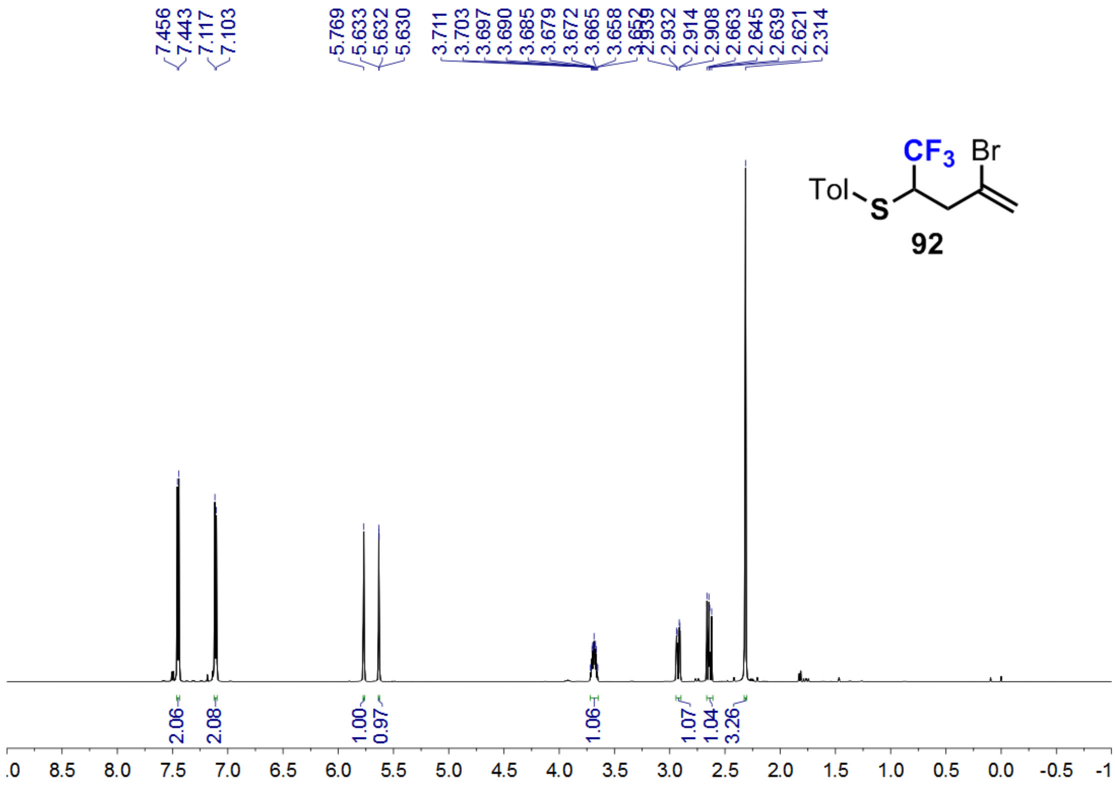

Supplementary Figure 270. ^{1}H NMR of 92

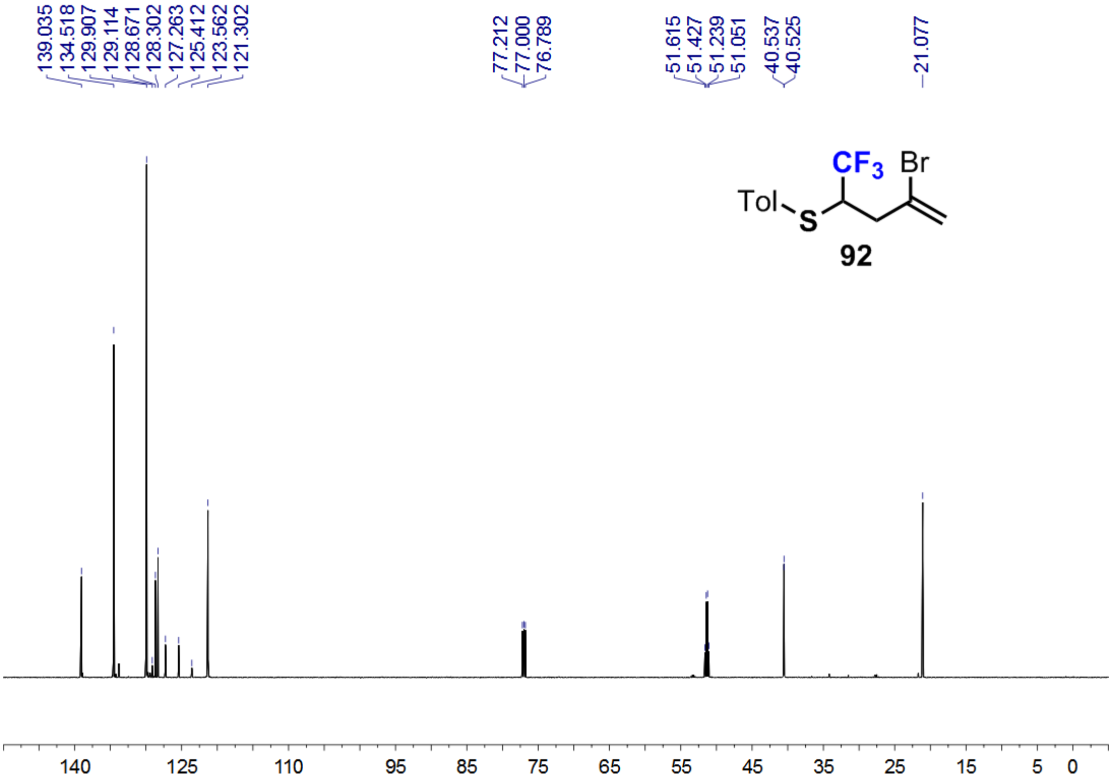

Supplementary Figure 271. ^{13}H NMR of 92

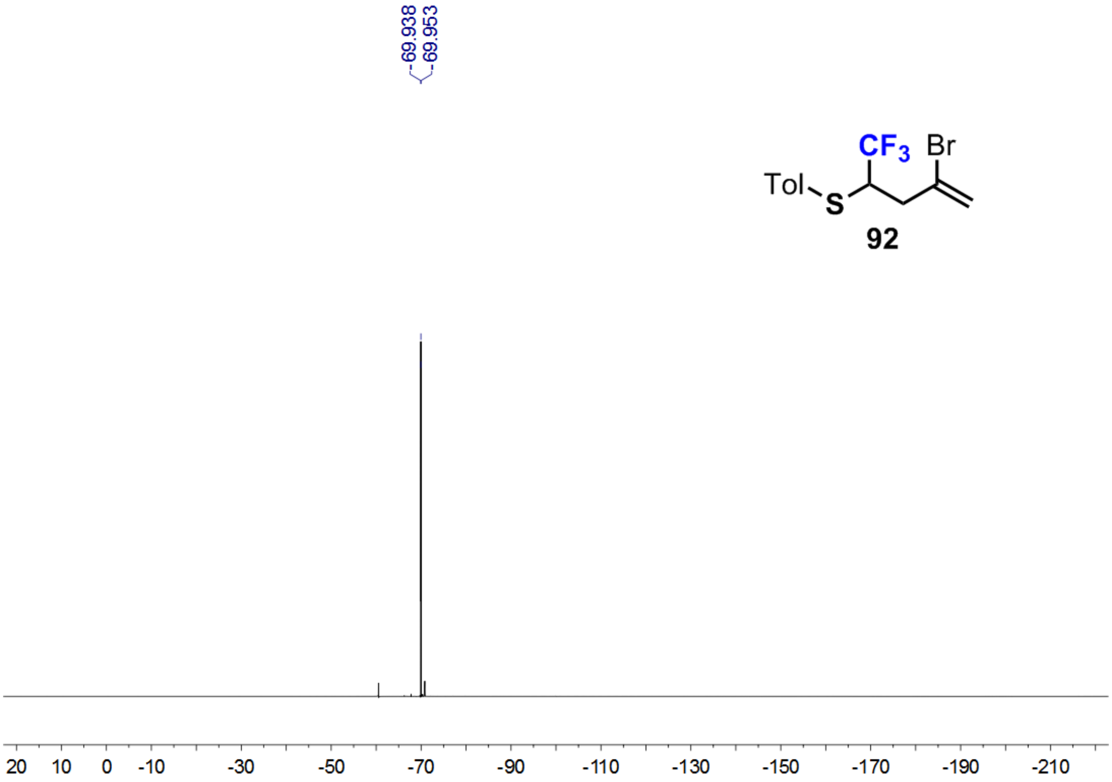

Supplementary Figure 272. ^{19}F NMR of 92

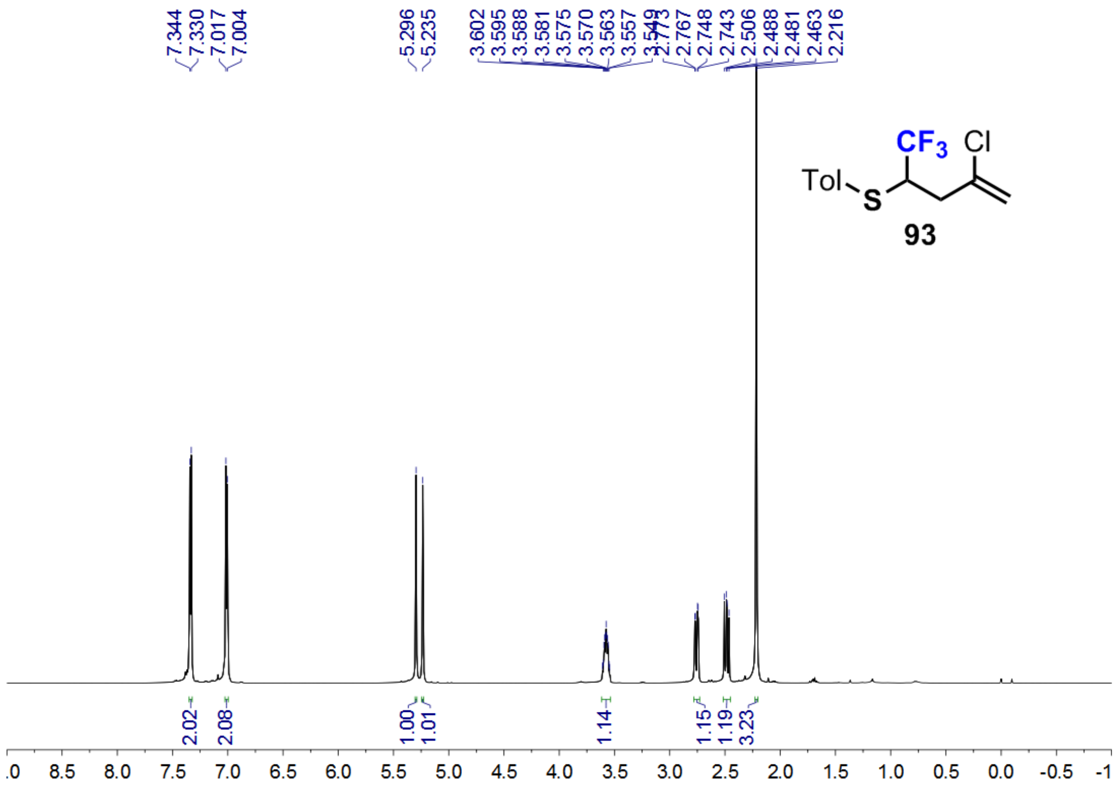

Supplementary Figure 273. ^{1}H NMR of 93

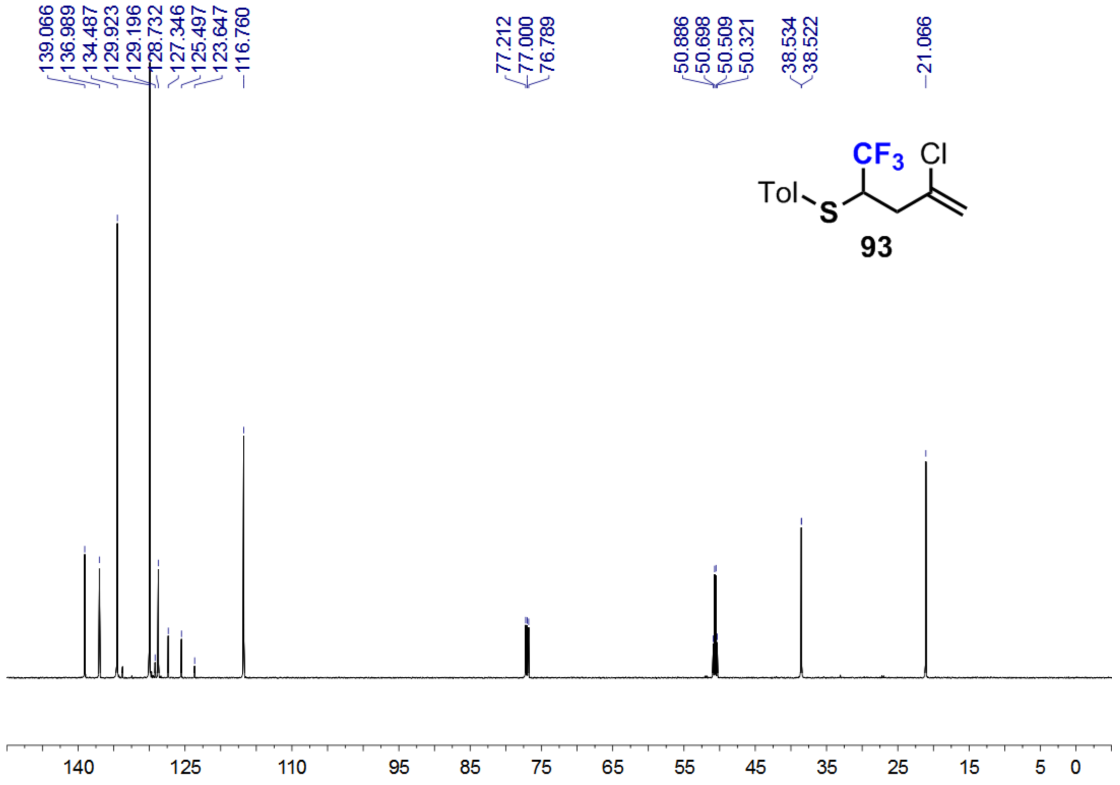

Supplementary Figure 274. ^{13}C NMR of 93

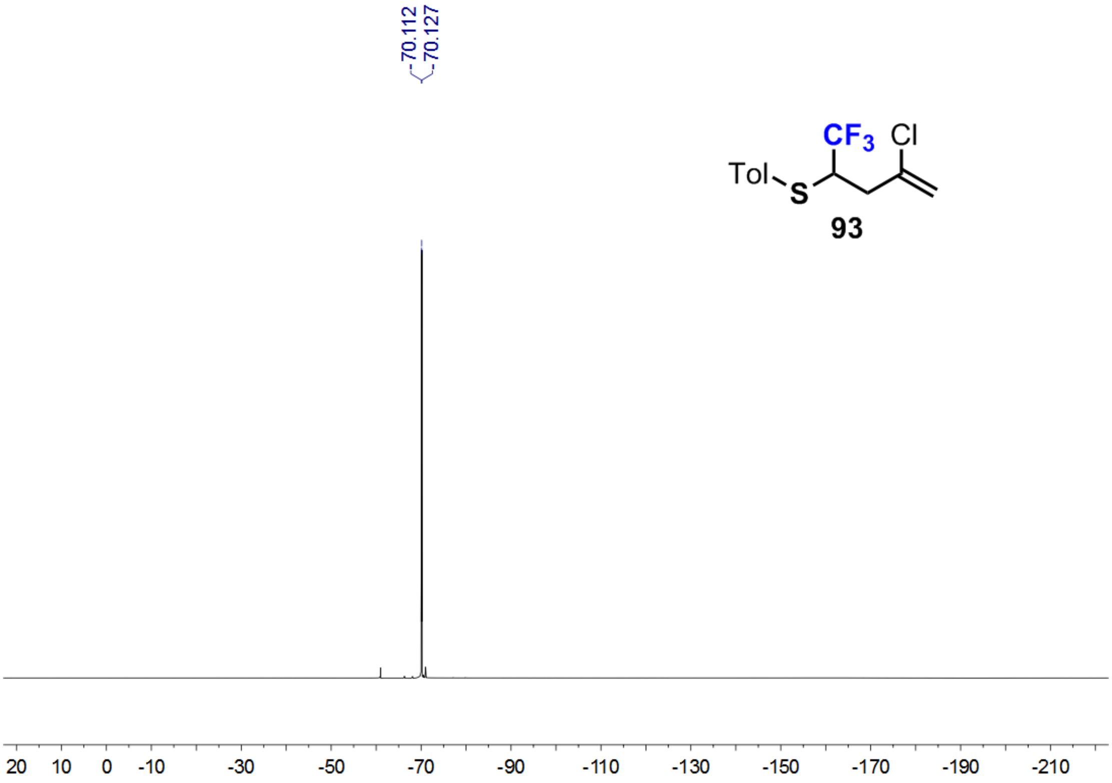

Supplementary Figure 275. ^{19}F NMR of 93

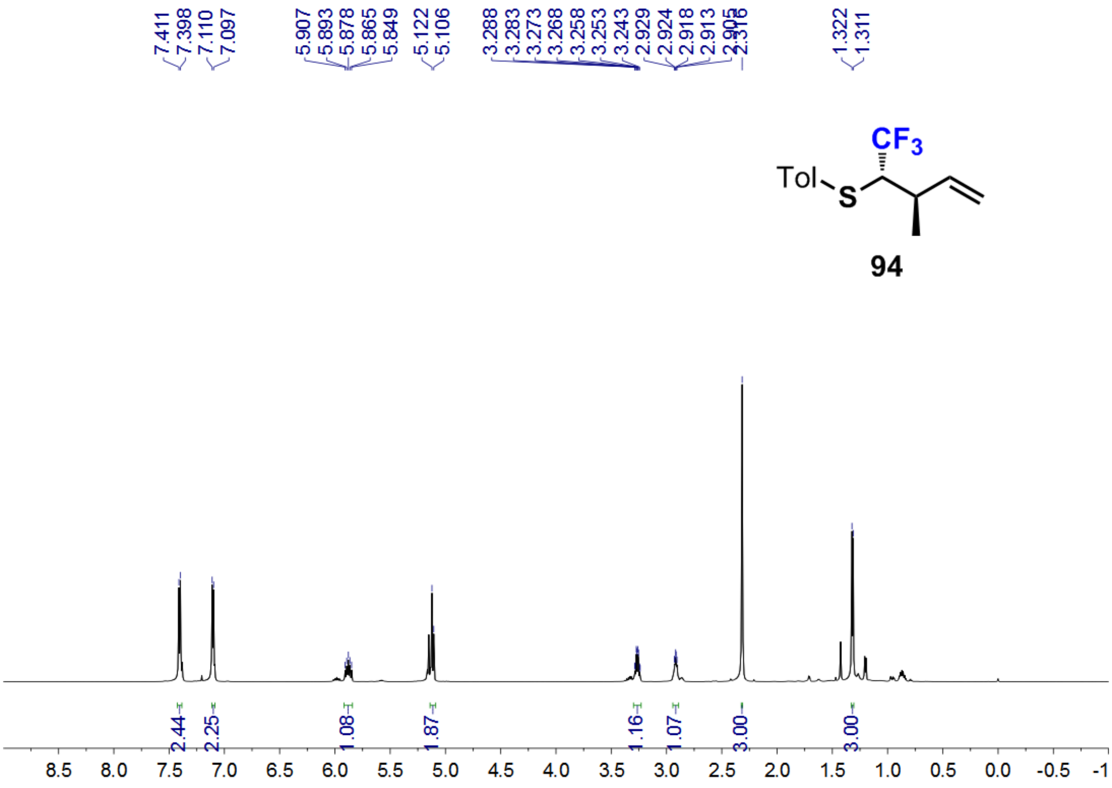

Supplementary Figure 276. ^{1}H NMR of 94

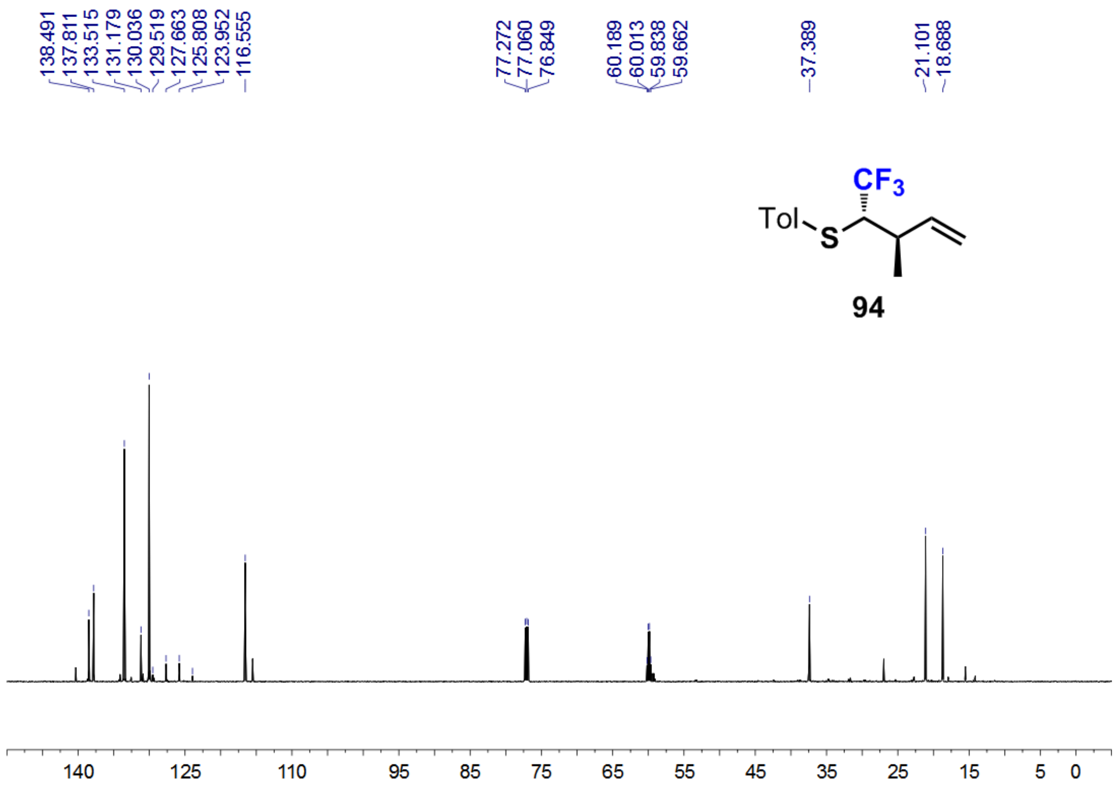

Supplementary Figure 277. ^{13}C NMR of 94

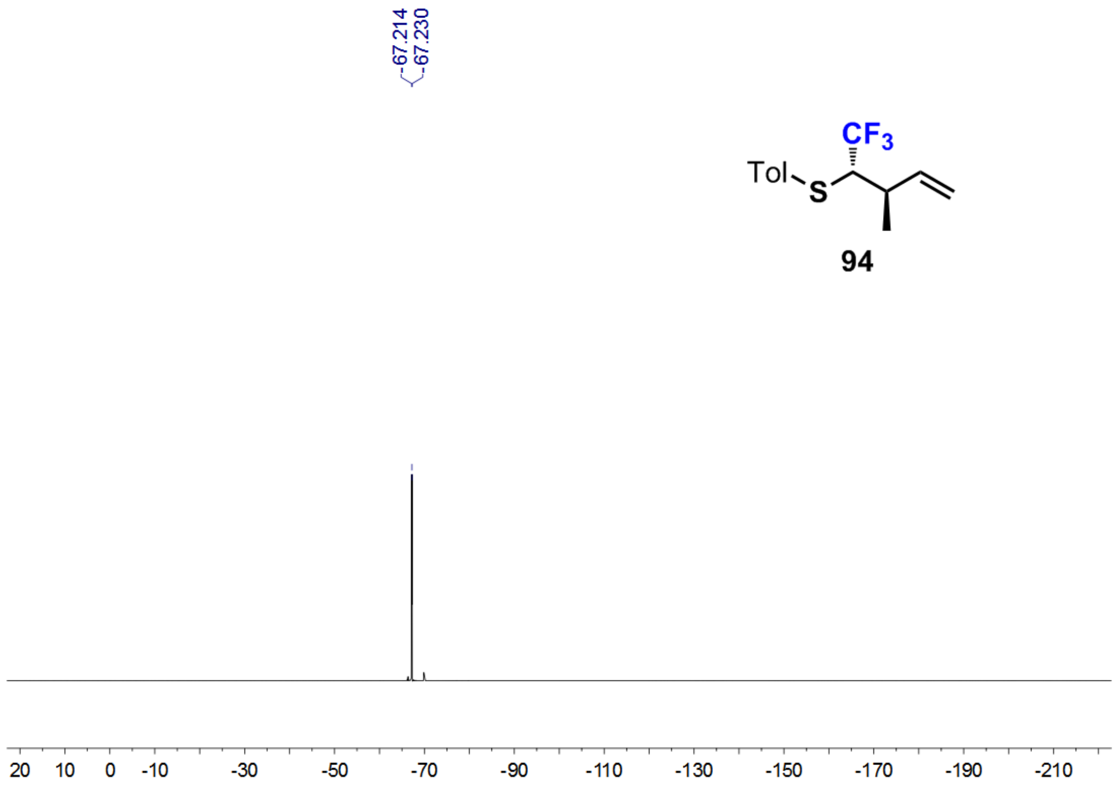

Supplementary Figure 278. ^{19}F NMR of 94

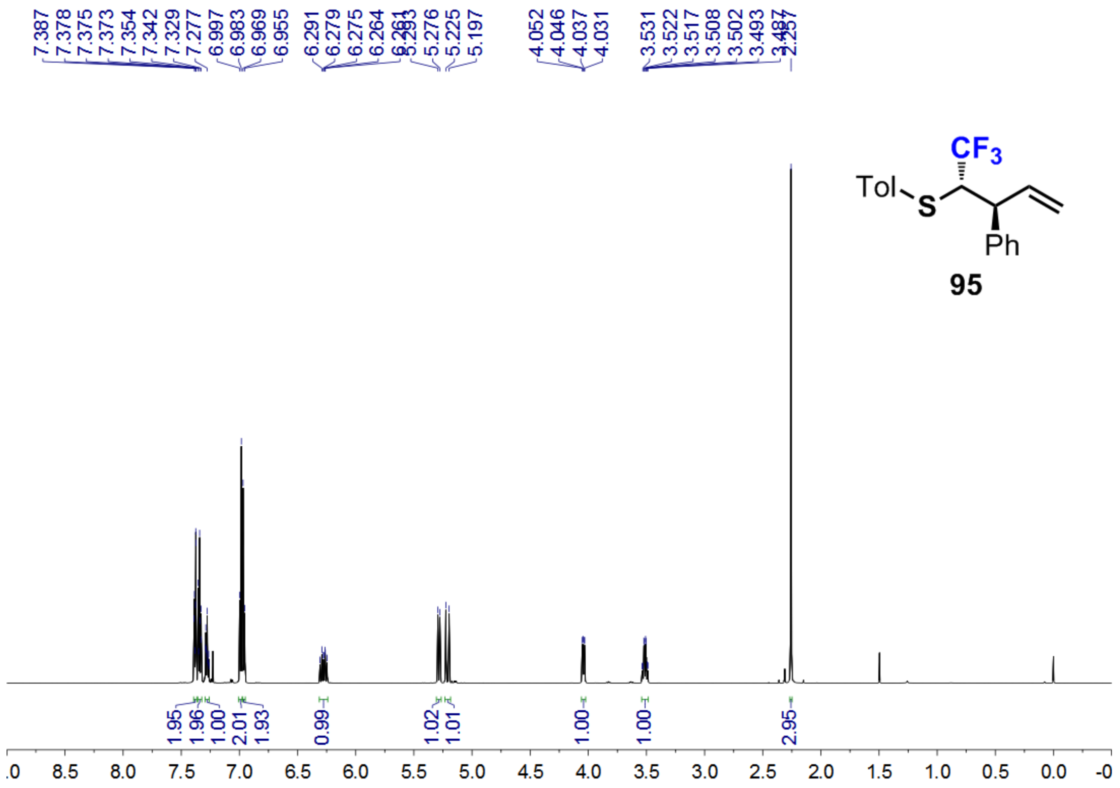

Supplementary Figure 279. ^{1}H NMR of 95

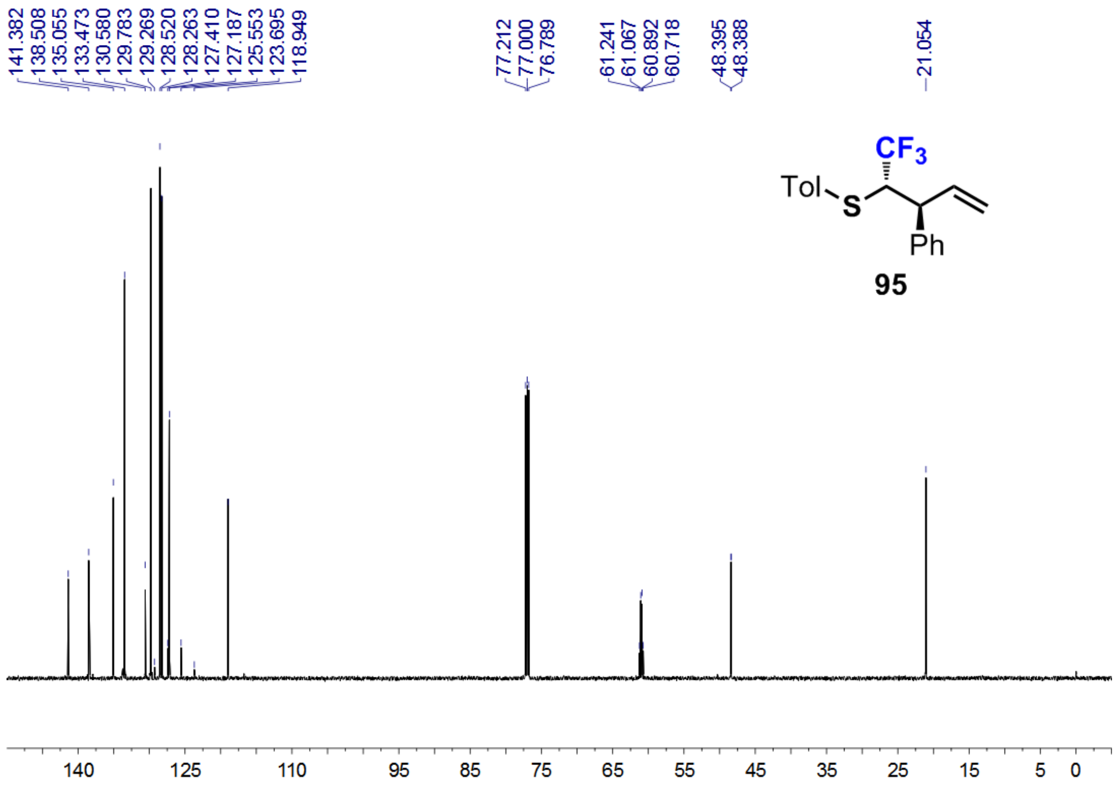

Supplementary Figure 280. ^{13}C NMR of 95

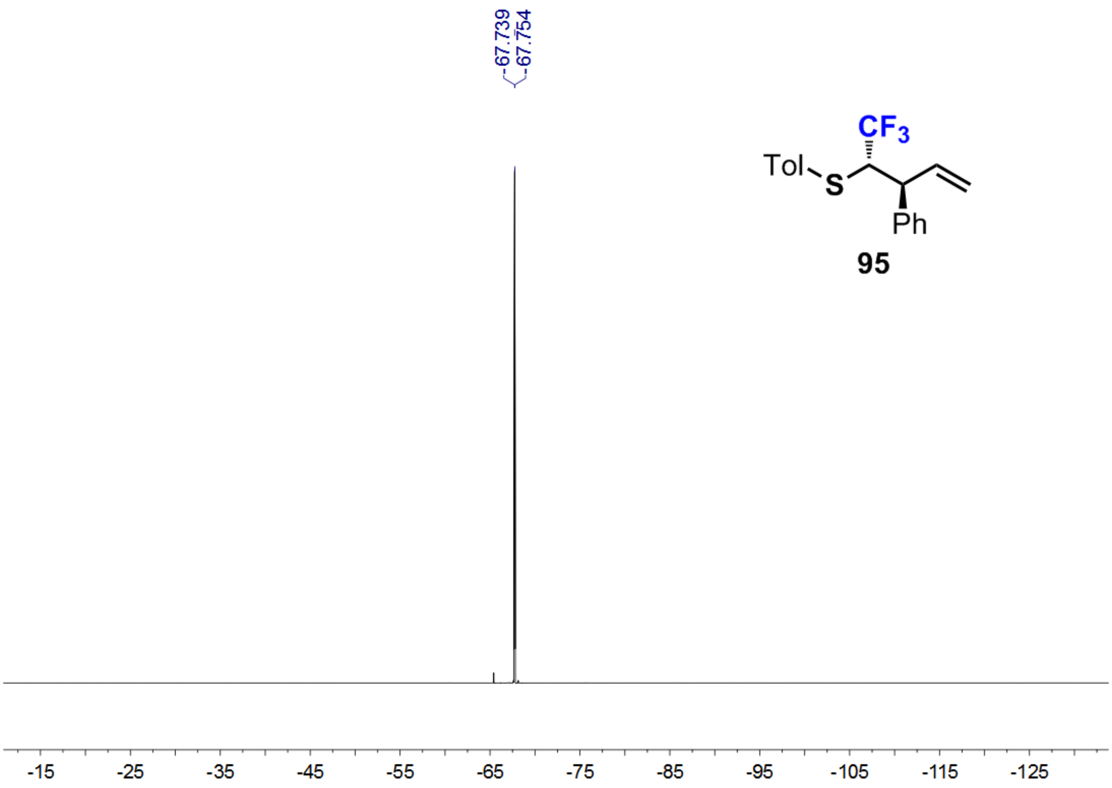

Supplementary Figure 281. ^{19}F NMR of 95

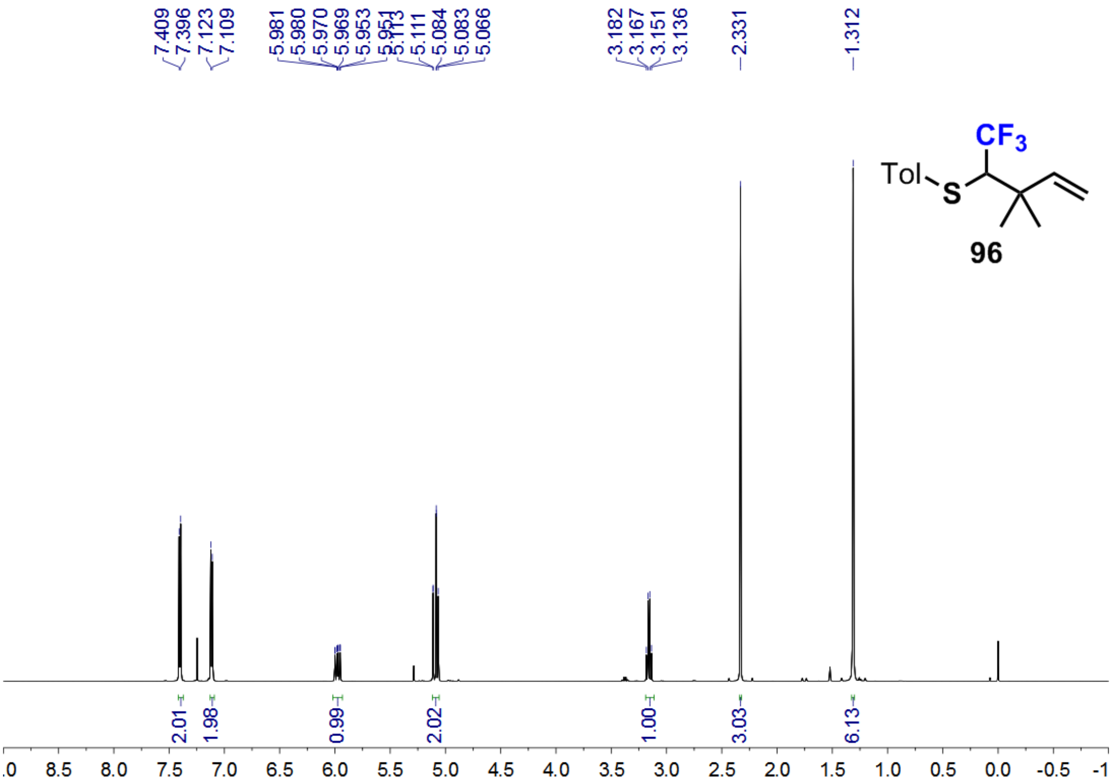

Supplementary Figure 282. ^{1}H NMR of 96

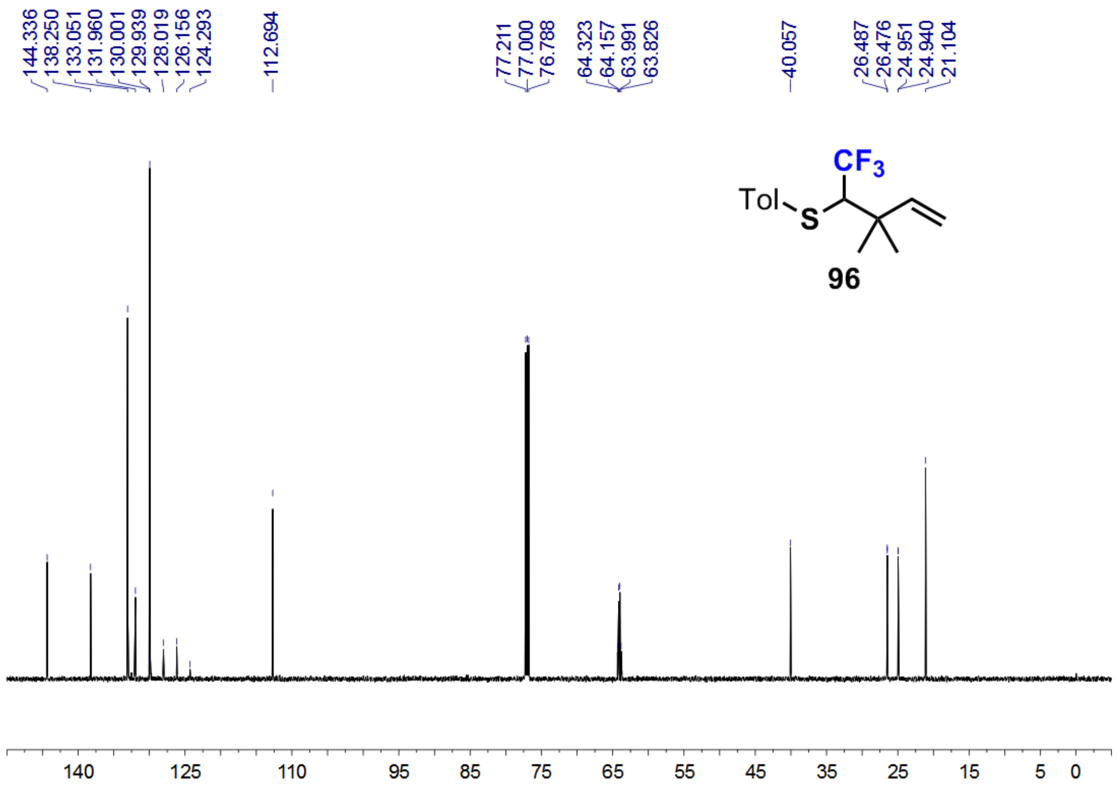

Supplementary Figure 283. ^{13}C NMR of 96

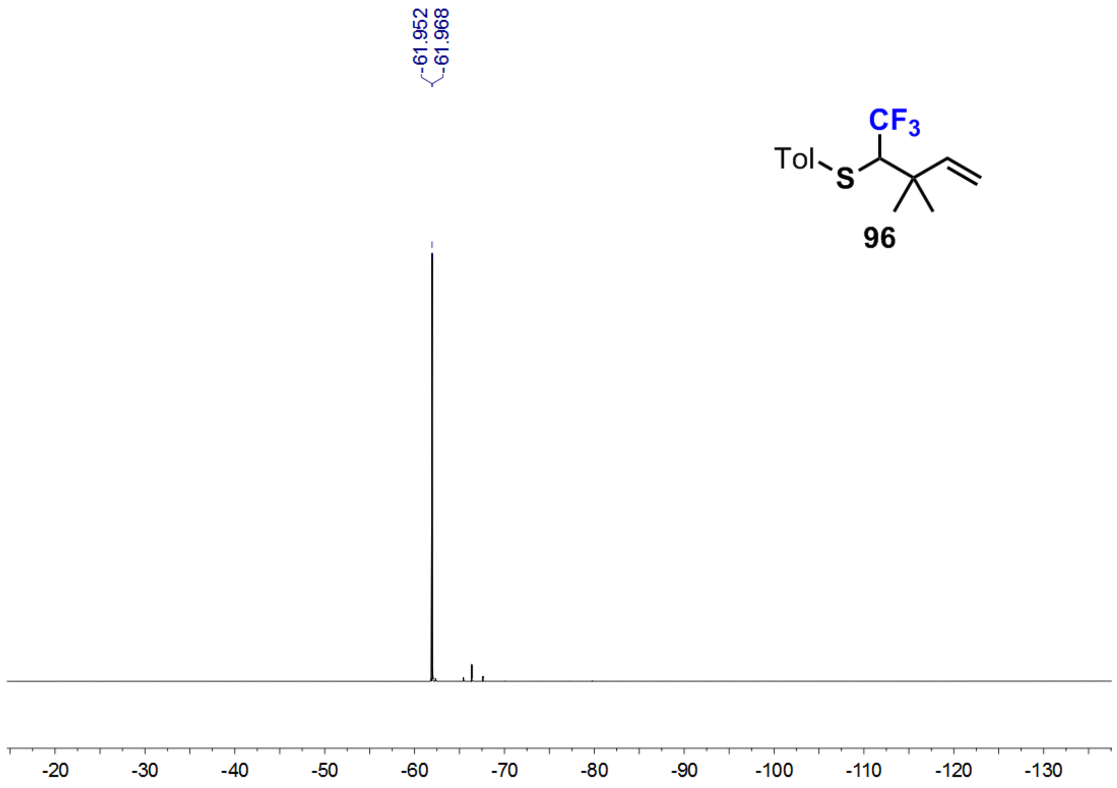

Supplementary Figure 284. ^{19}F NMR of 96

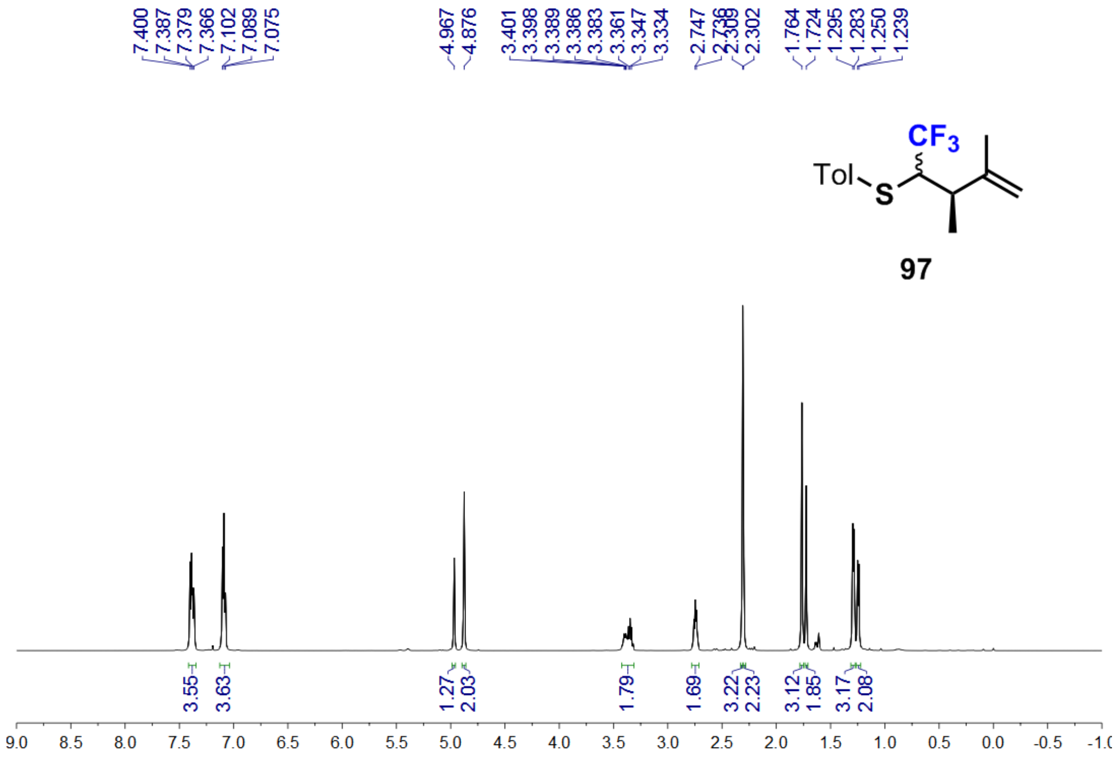

Supplementary Figure 285. ^{1}H NMR of 97

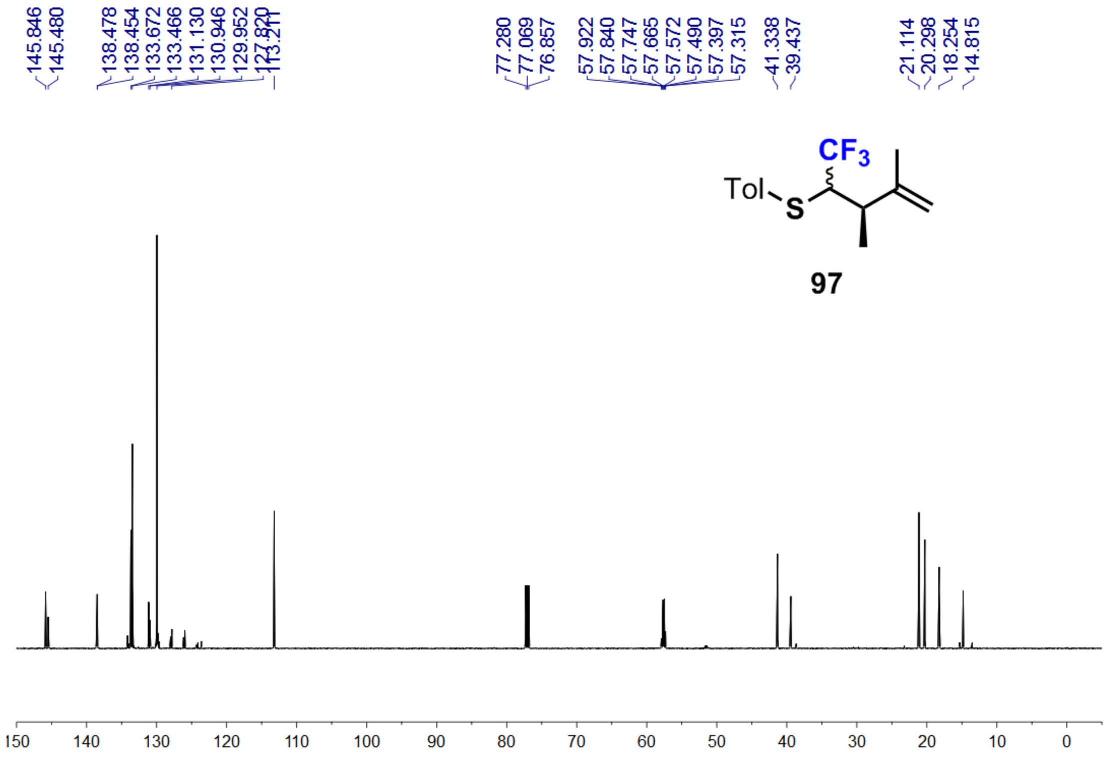

Supplementary Figure 286. ^{13}C NMR of 97

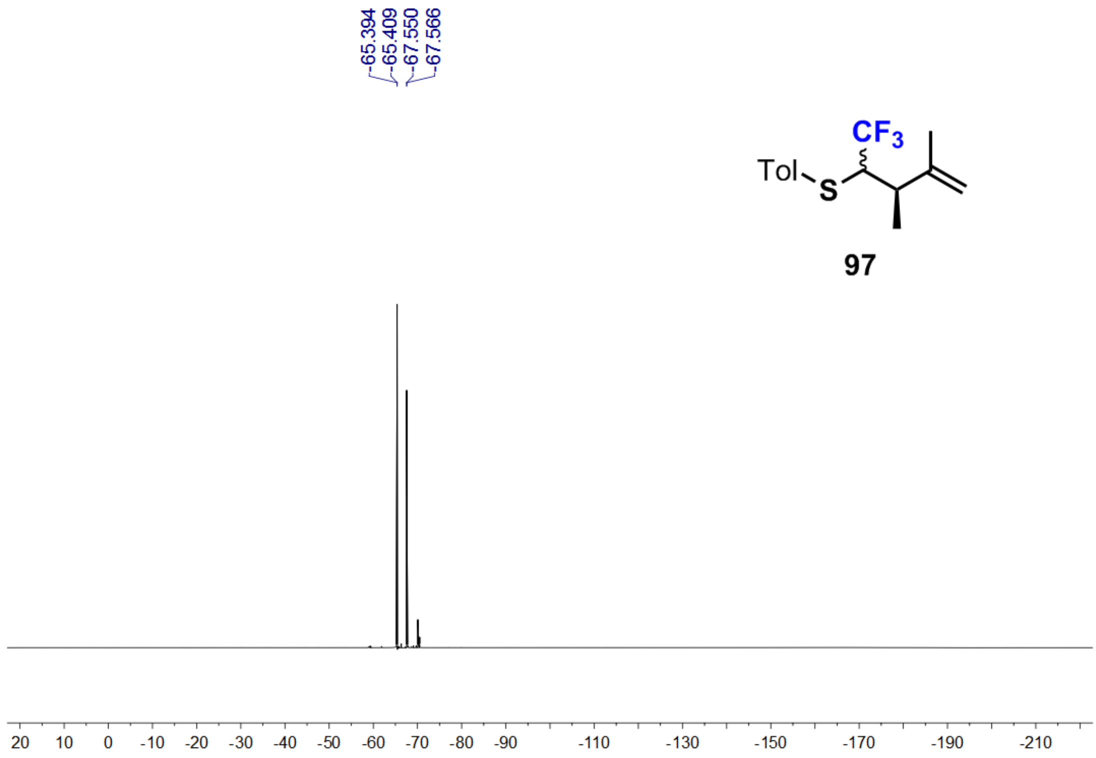

Supplementary Figure 287. ^{19}F NMR of 97

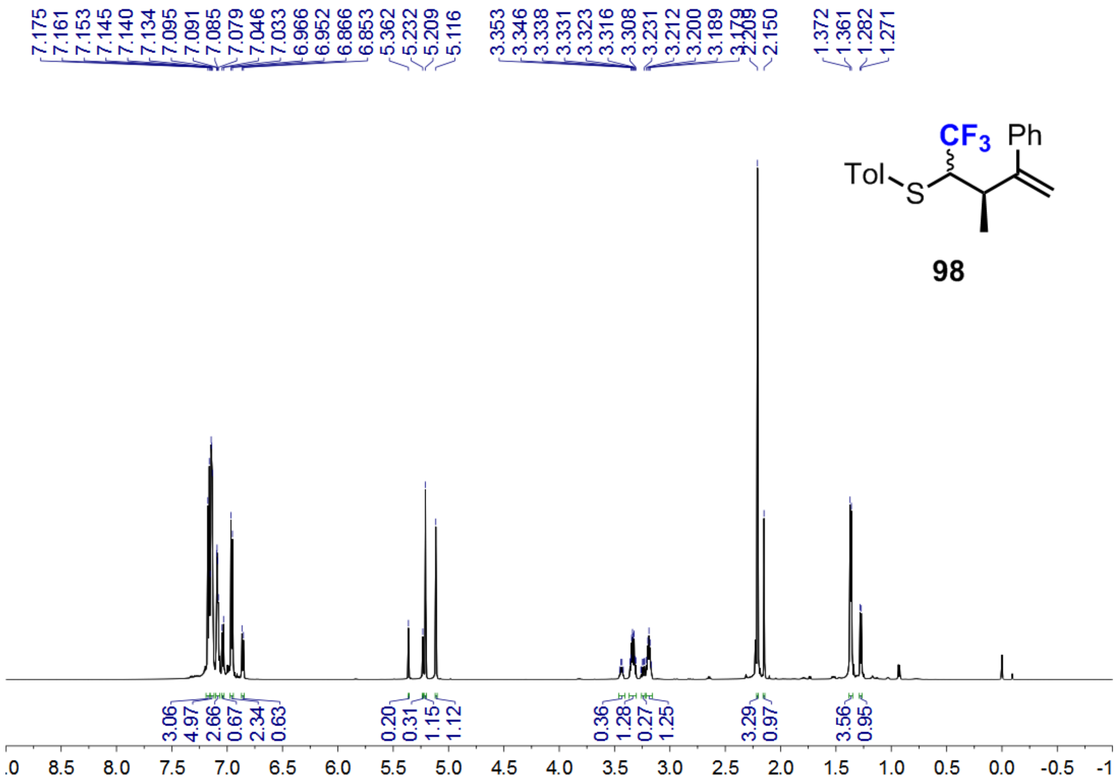

Supplementary Figure 288. ^{1}H NMR of 98

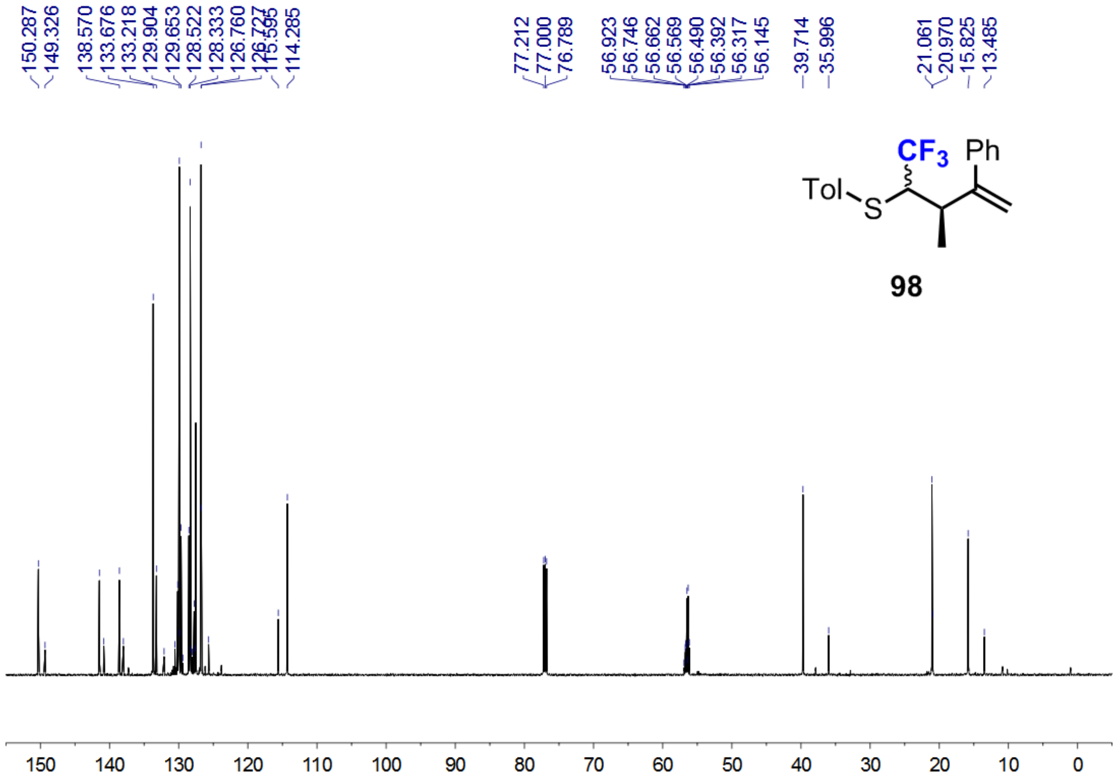

Supplementary Figure 289. ^{13}C NMR of 98

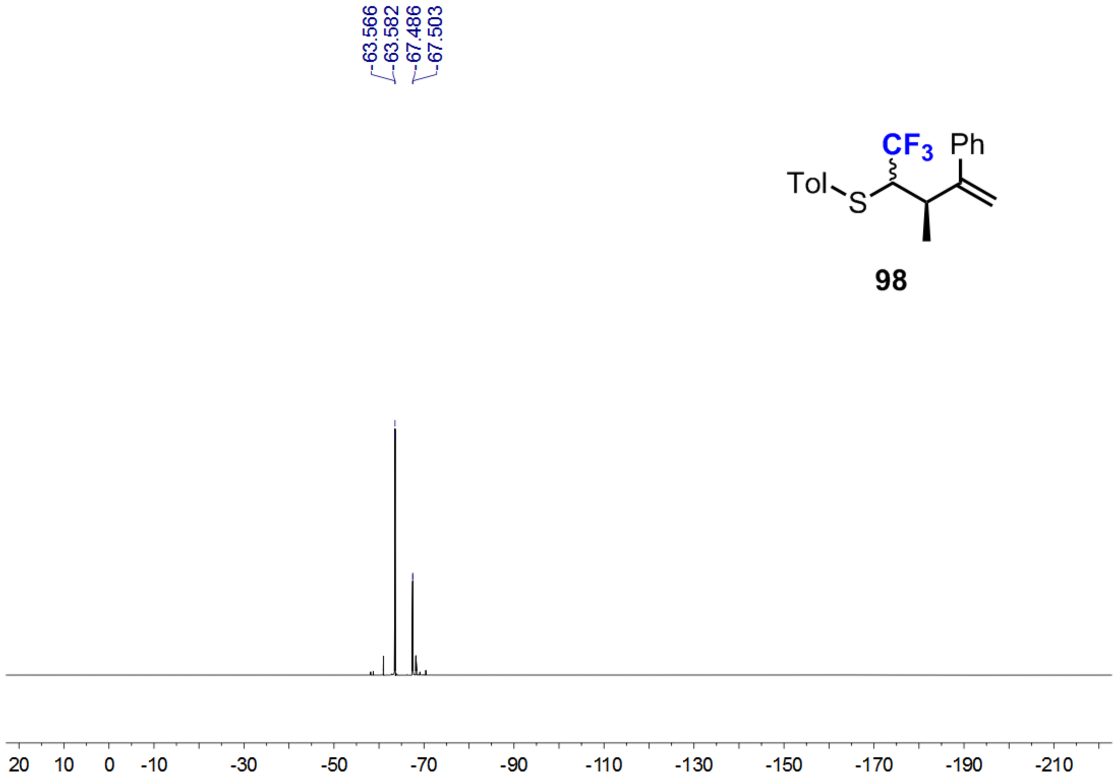

Supplementary Figure 290. ^{19}F NMR of 98

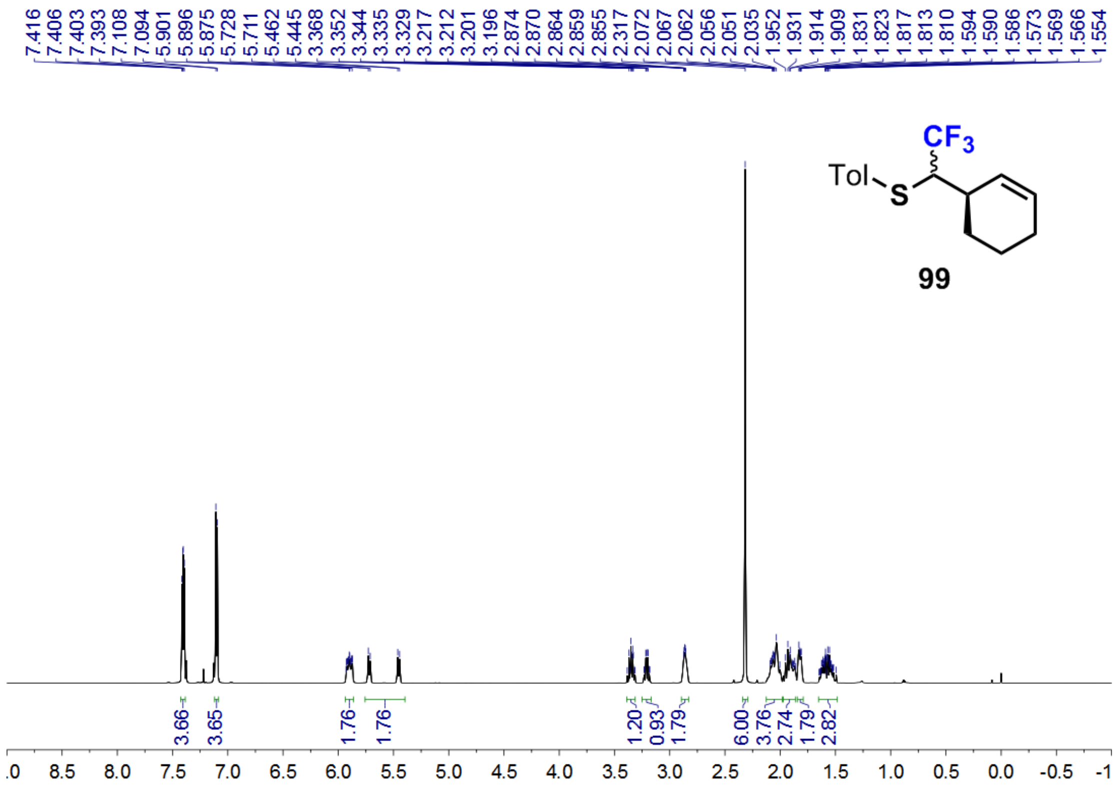

Supplementary Figure 291. ^{1}H NMR of 99

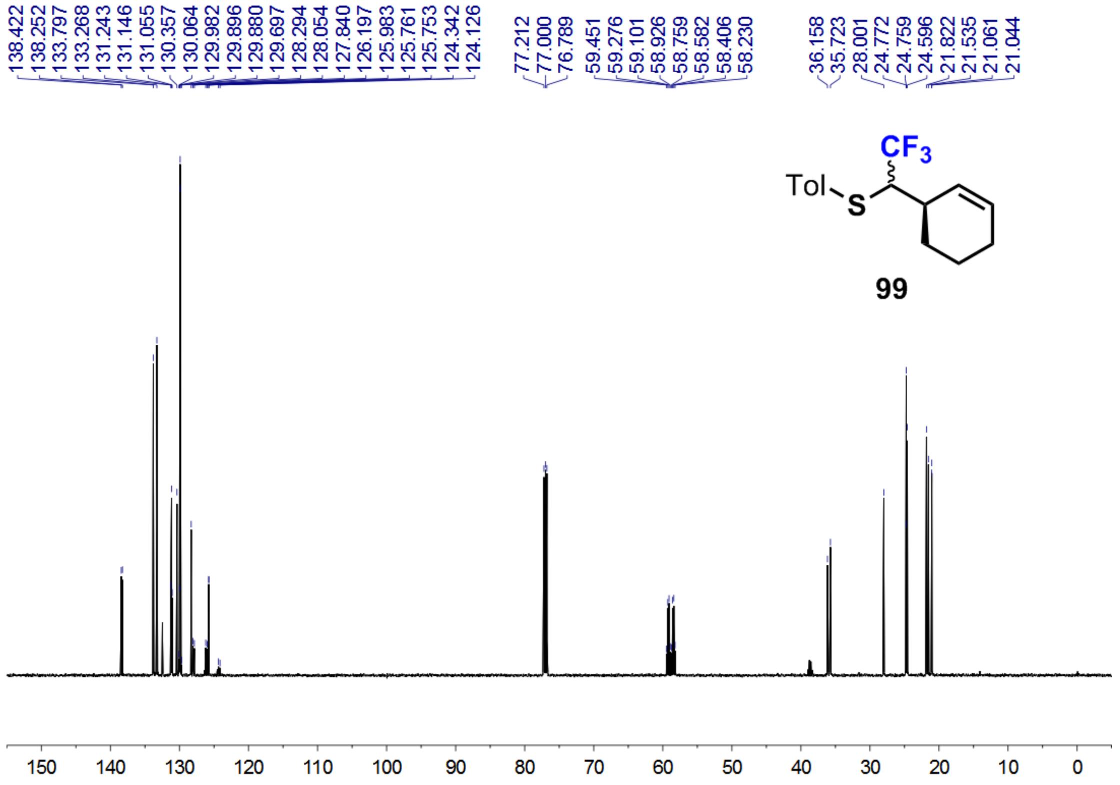

Supplementary Figure 292. ^{13}C NMR of 99

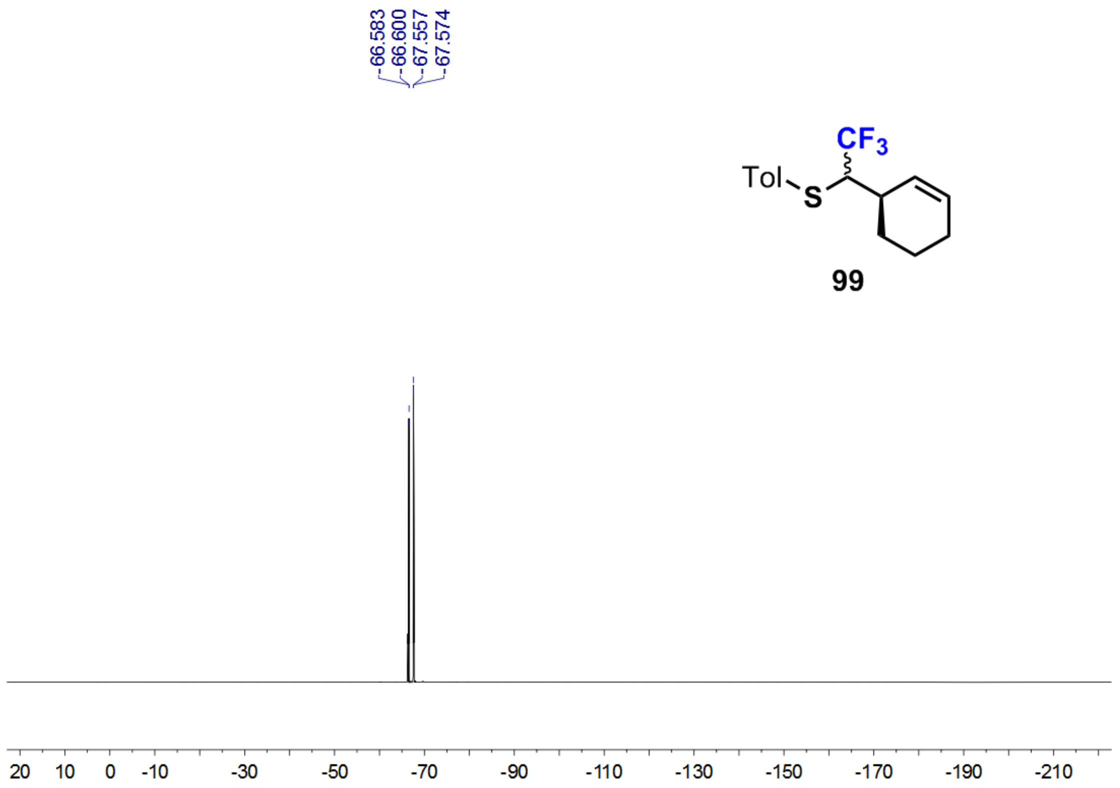

Supplementary Figure 293. ^{19}F NMR of 99

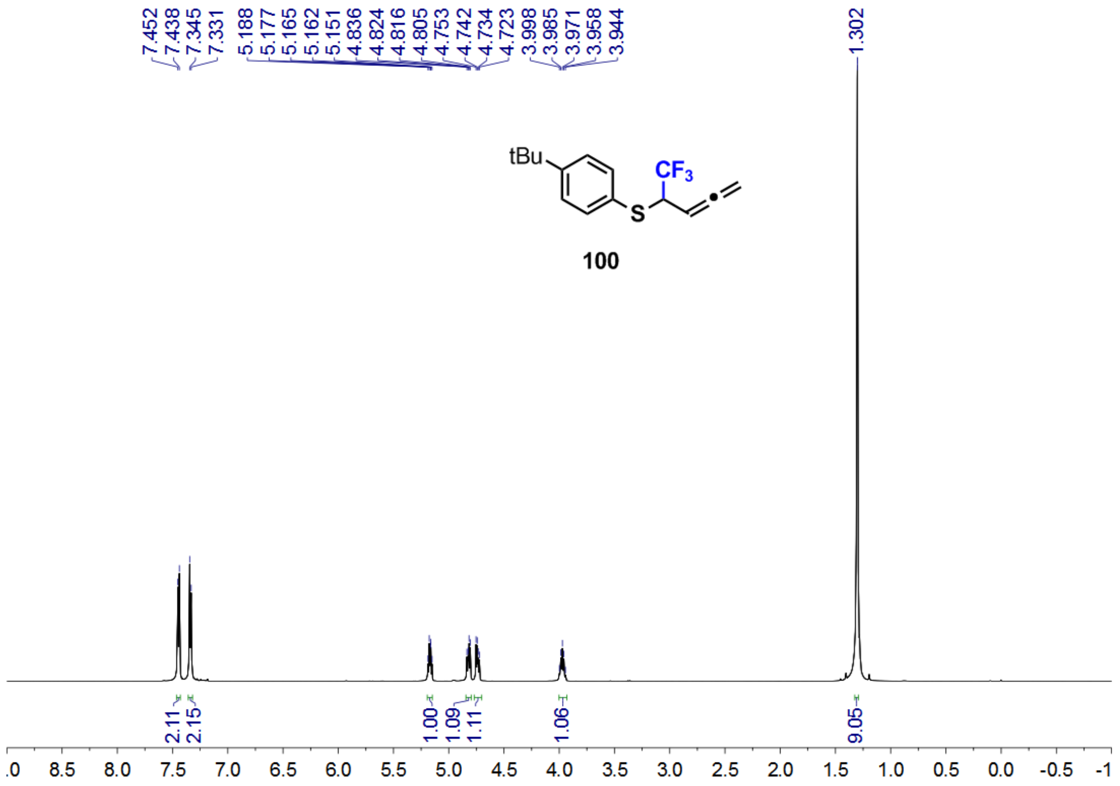

Supplementary Figure 294. ^{1}H NMR of 100

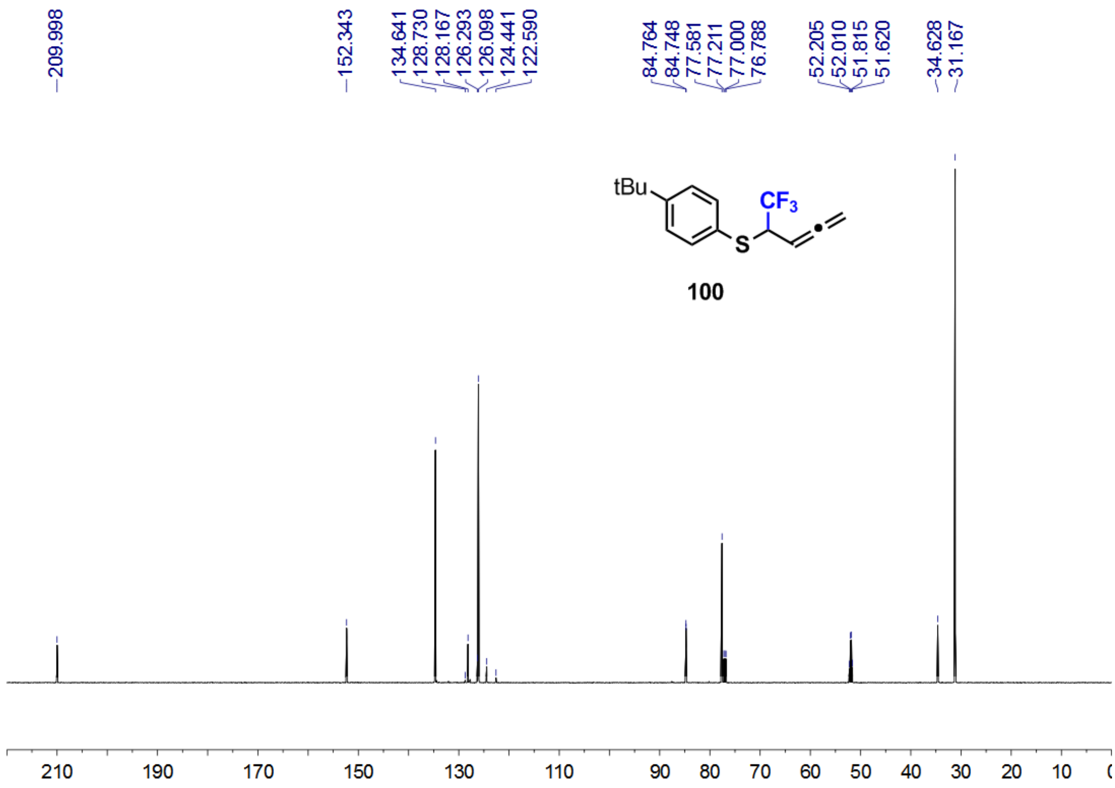

Supplementary Figure 295. ^{13}C NMR of 100

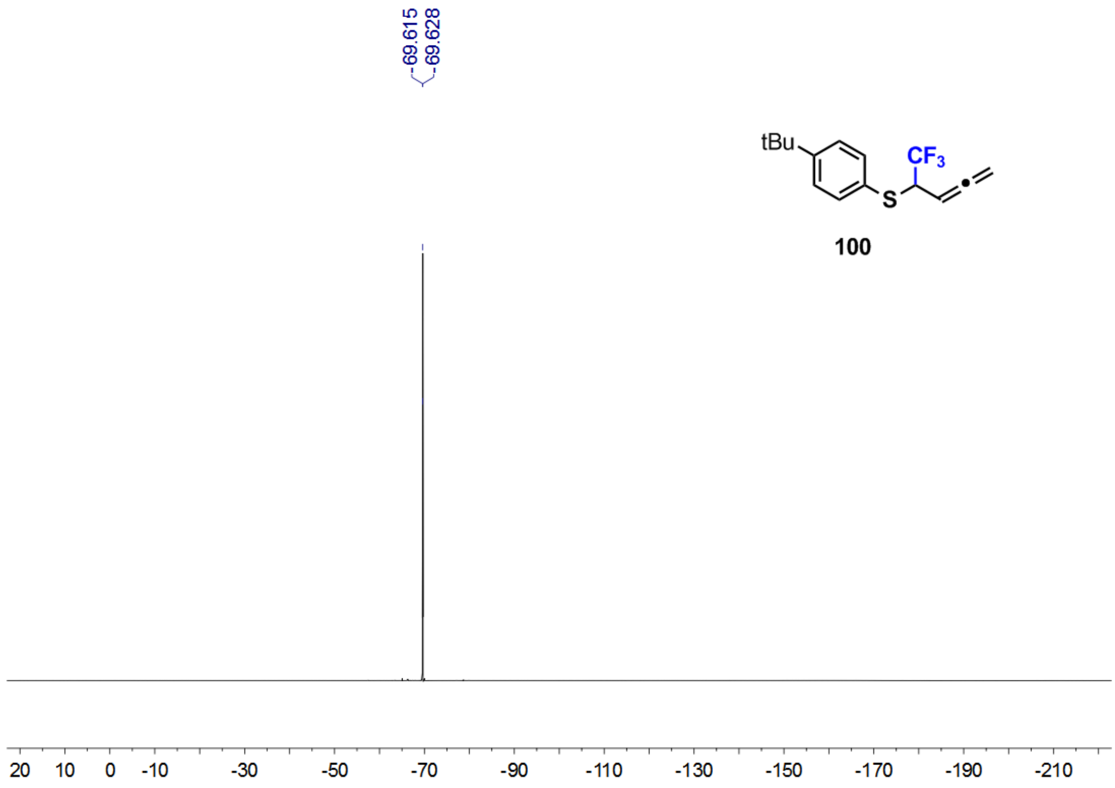

Supplementary Figure 296. ^{19}F NMR of 100

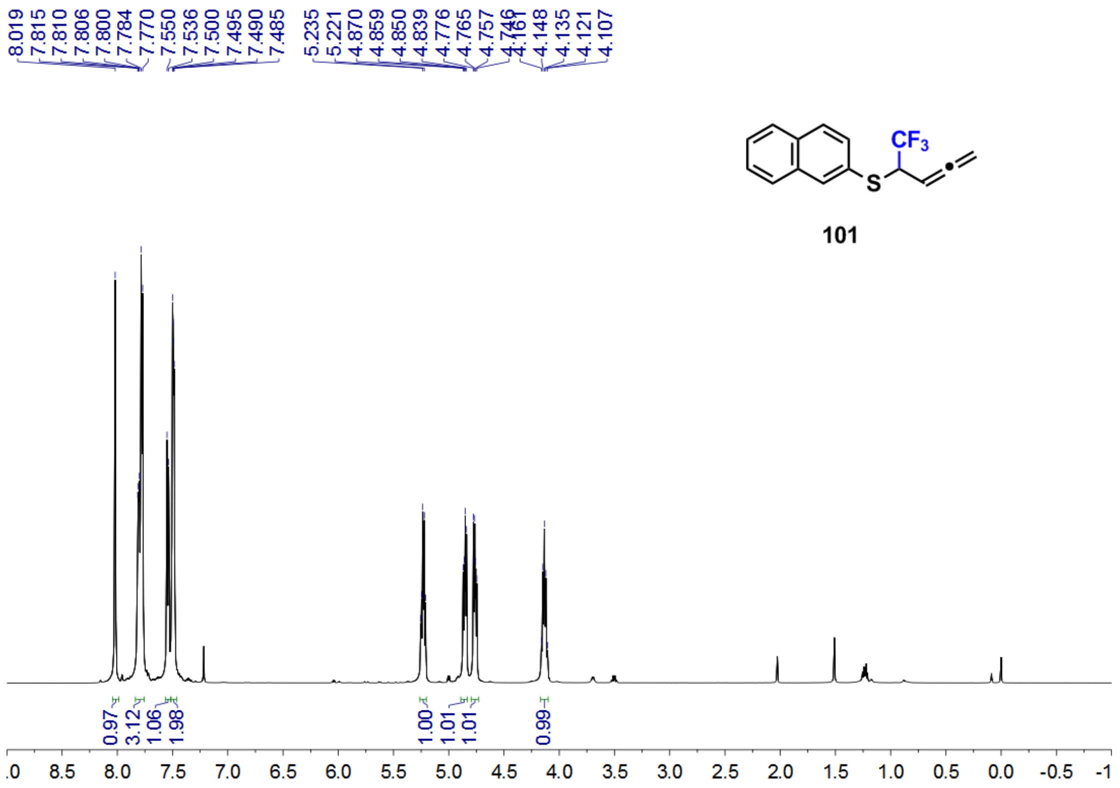

Supplementary Figure 297. ^{1}H NMR of 101

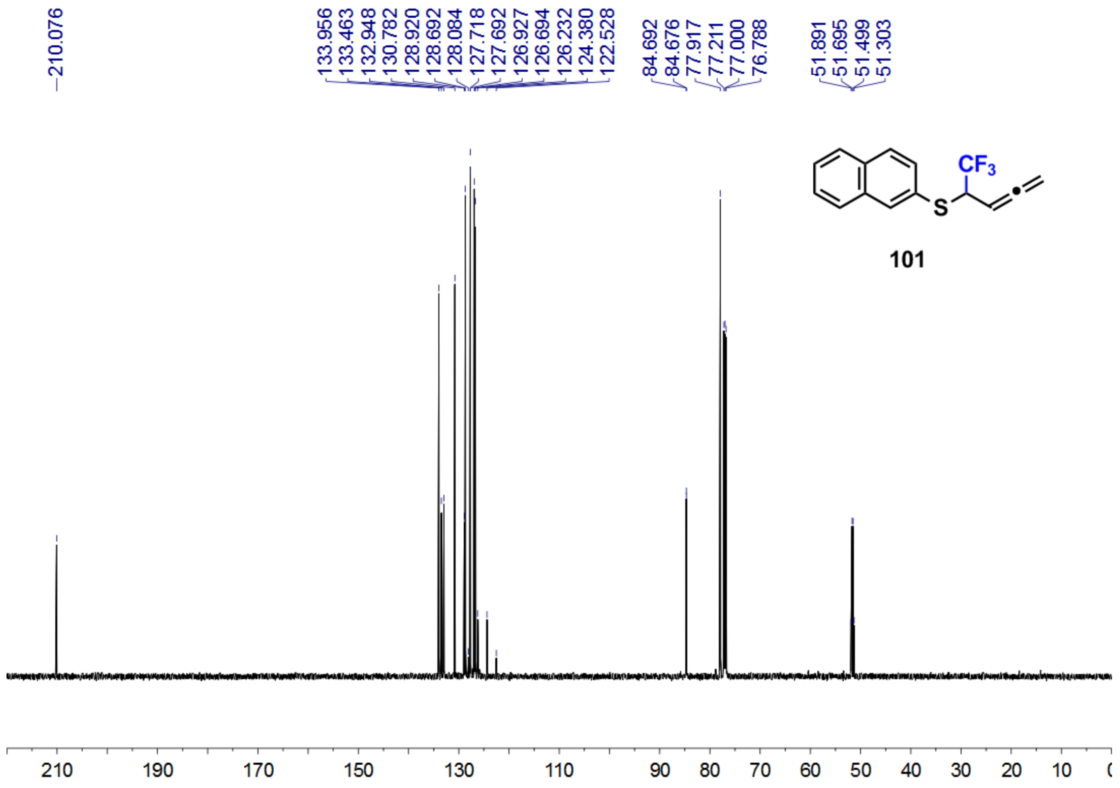

Supplementary Figure 298. ^{13}C NMR of 101

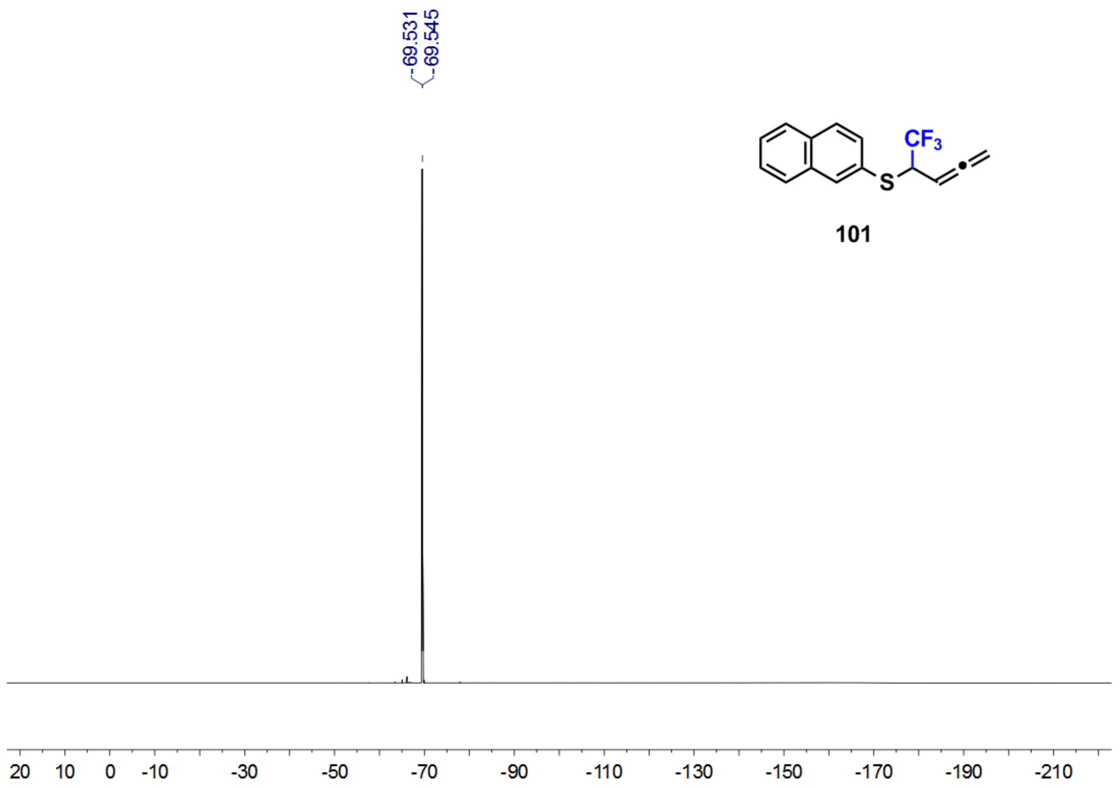

Supplementary Figure 299. ^{19}F NMR of 101

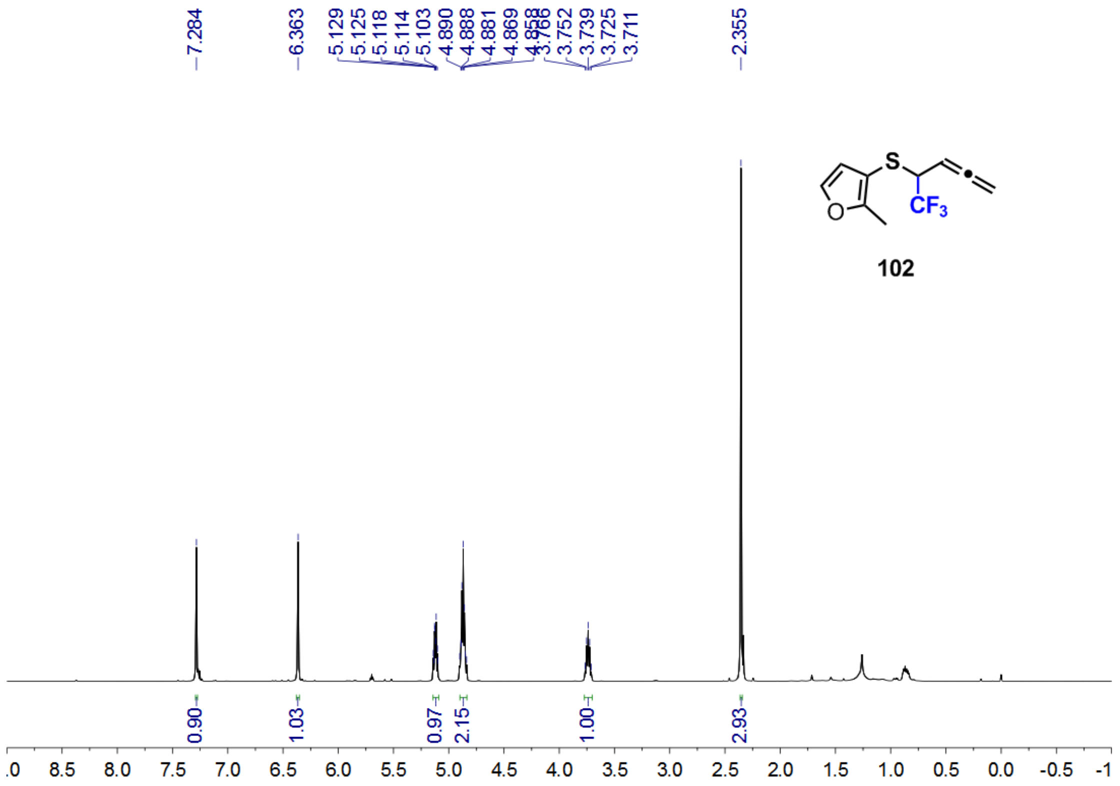

Supplementary Figure 300. ^{1}H NMR of 102

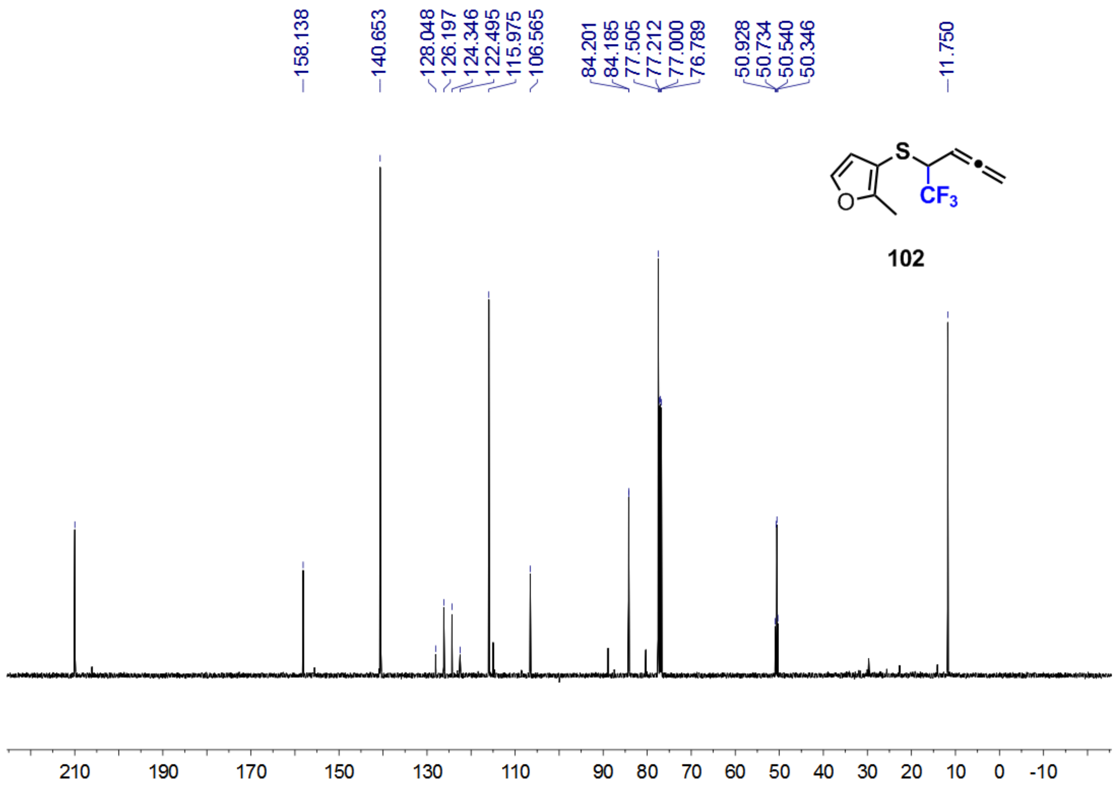

Supplementary Figure 301. ^{13}C NMR of 102

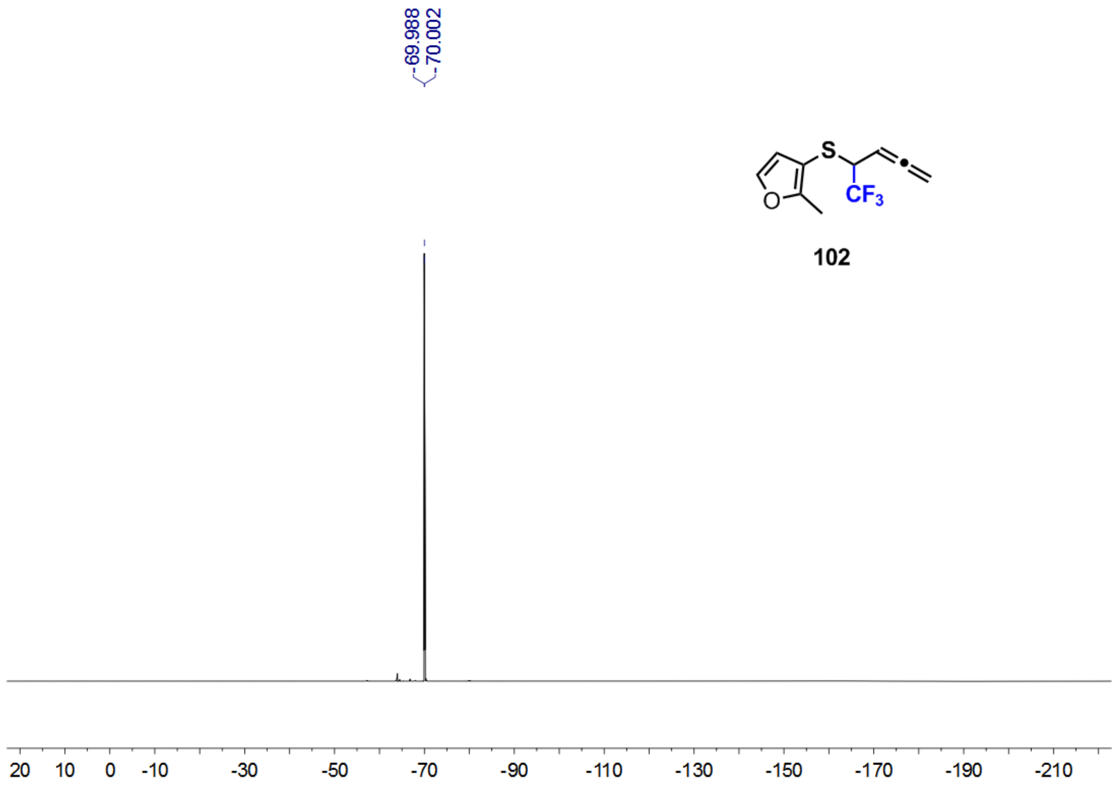

Supplementary Figure 302. ^{19}F NMR of 102

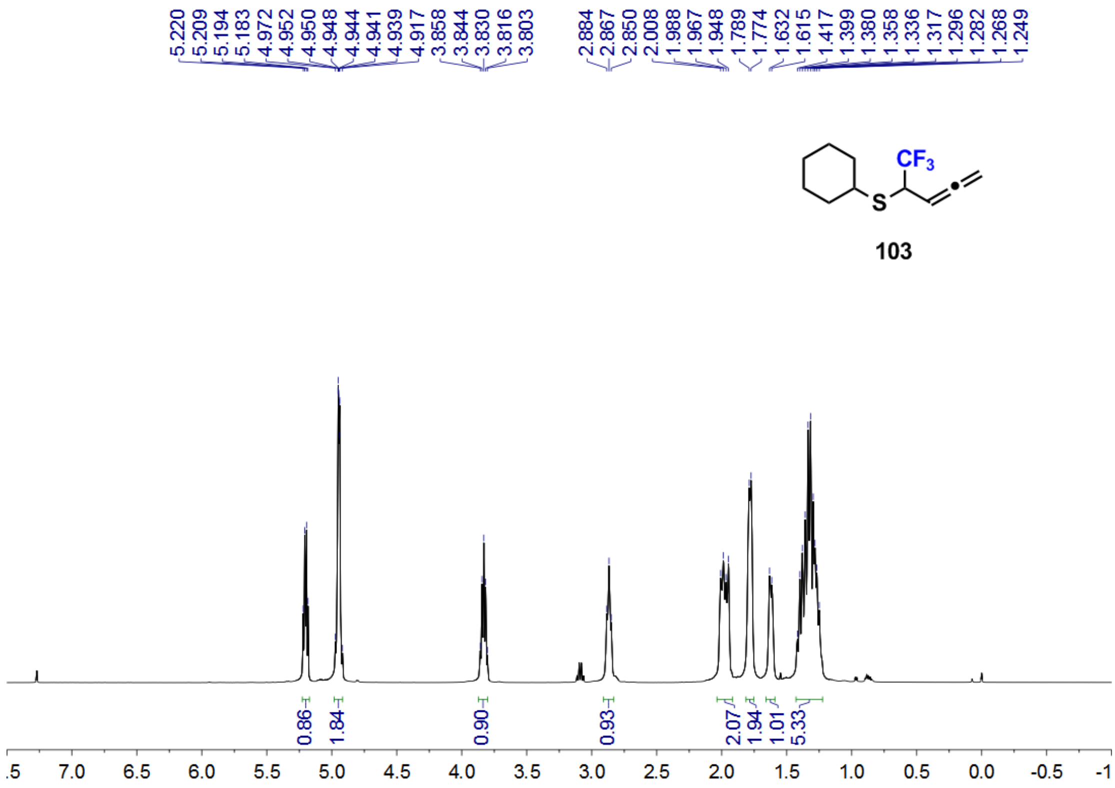

Supplementary Figure 303. ^{1}H NMR of 103

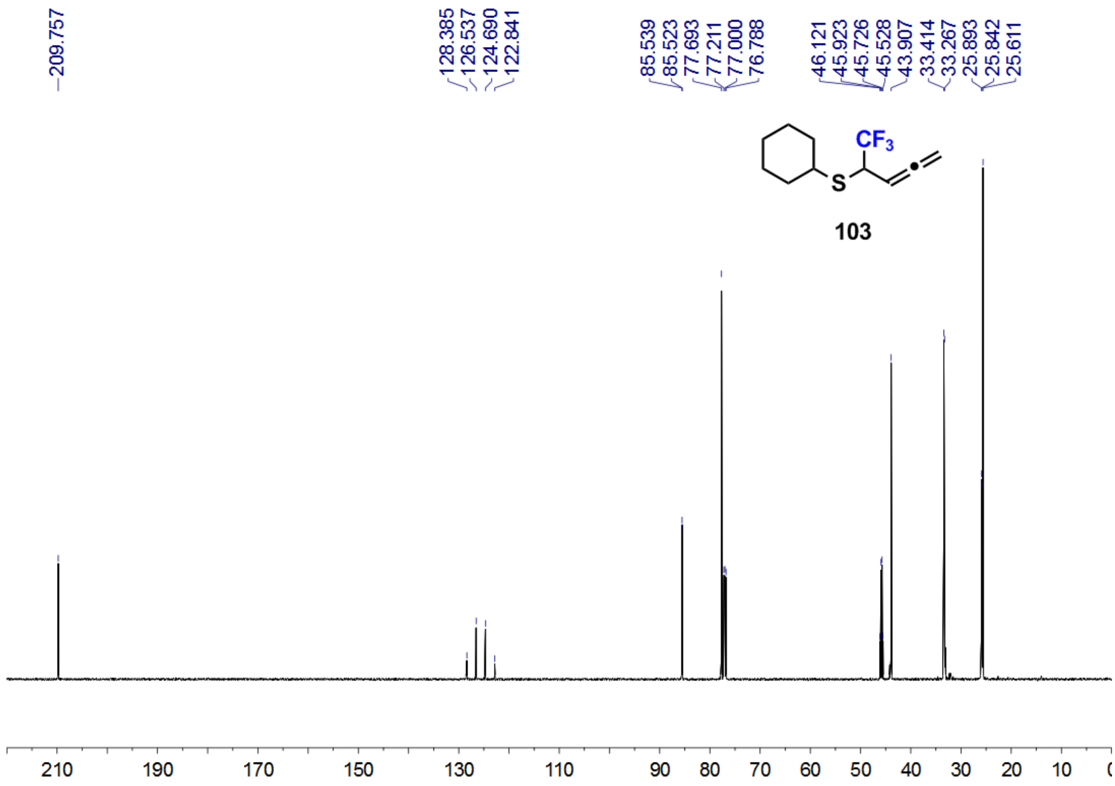

Supplementary Figure 304. ^{13}C NMR of 103

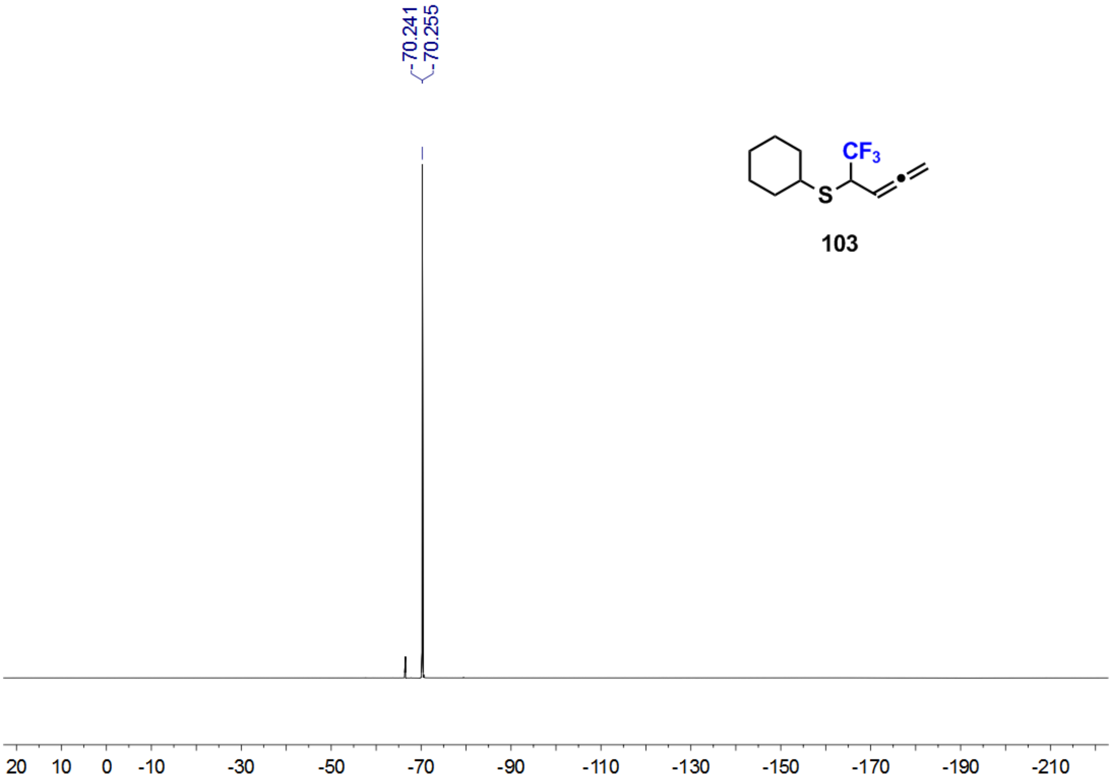

Supplementary Figure 305. ^{19}F NMR of 103

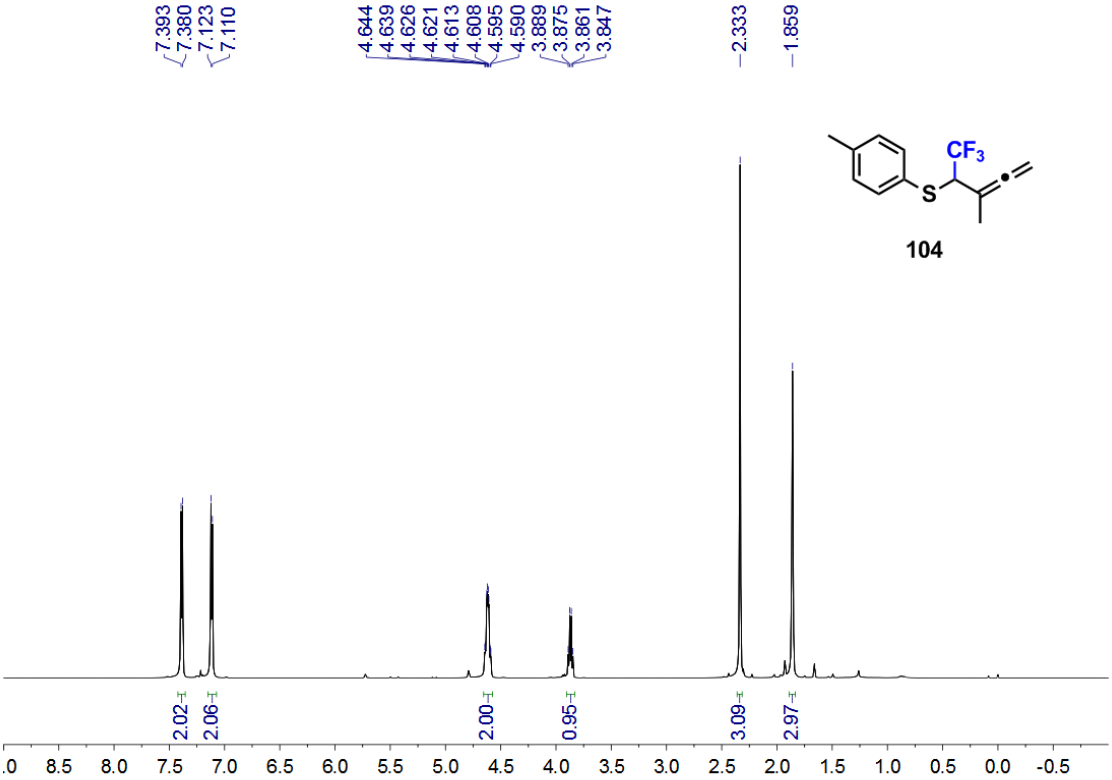

Supplementary Figure 306. ^{1}H NMR of 104

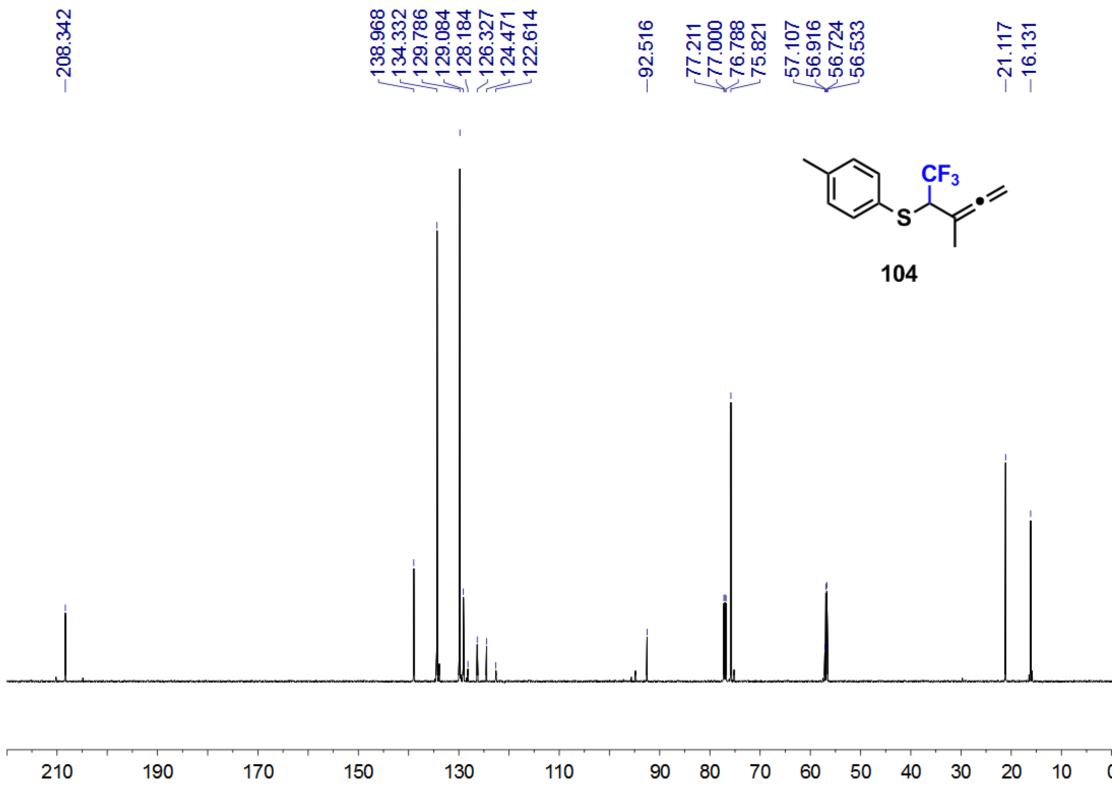

Supplementary Figure 307. ^{13}C NMR of 104

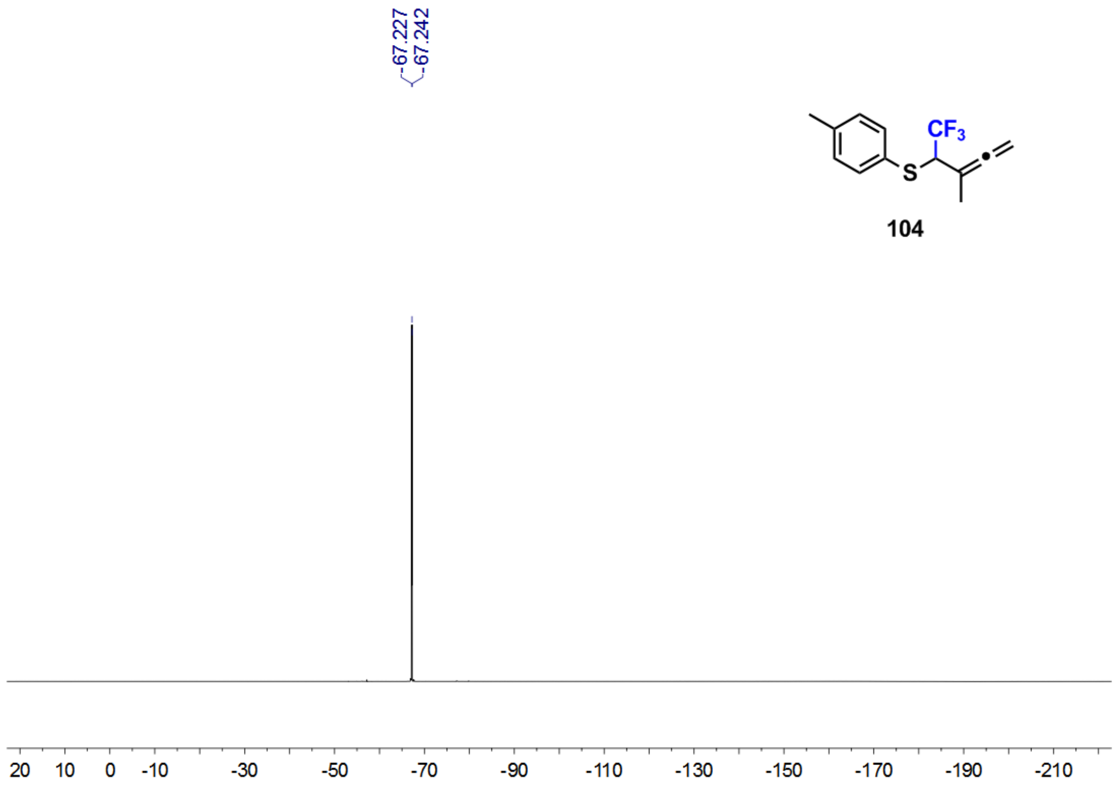

Supplementary Figure 308. ^{19}F NMR of 104

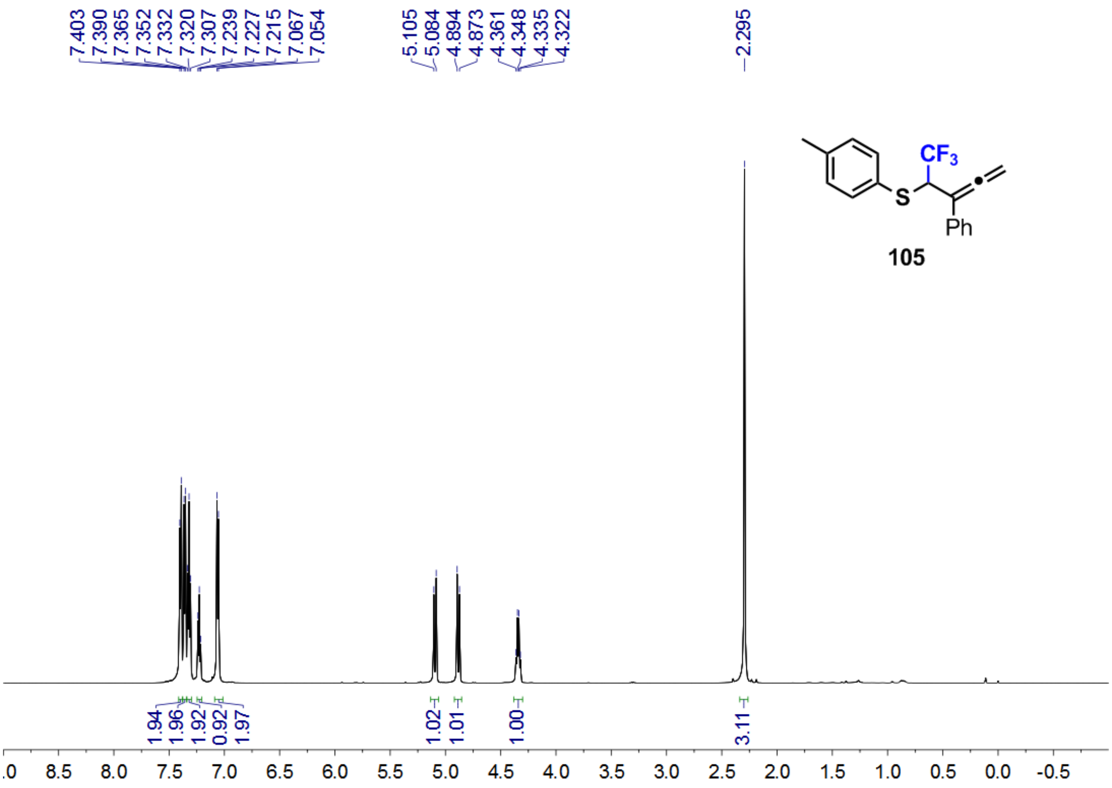

Supplementary Figure 309. ^{1}H NMR of 105

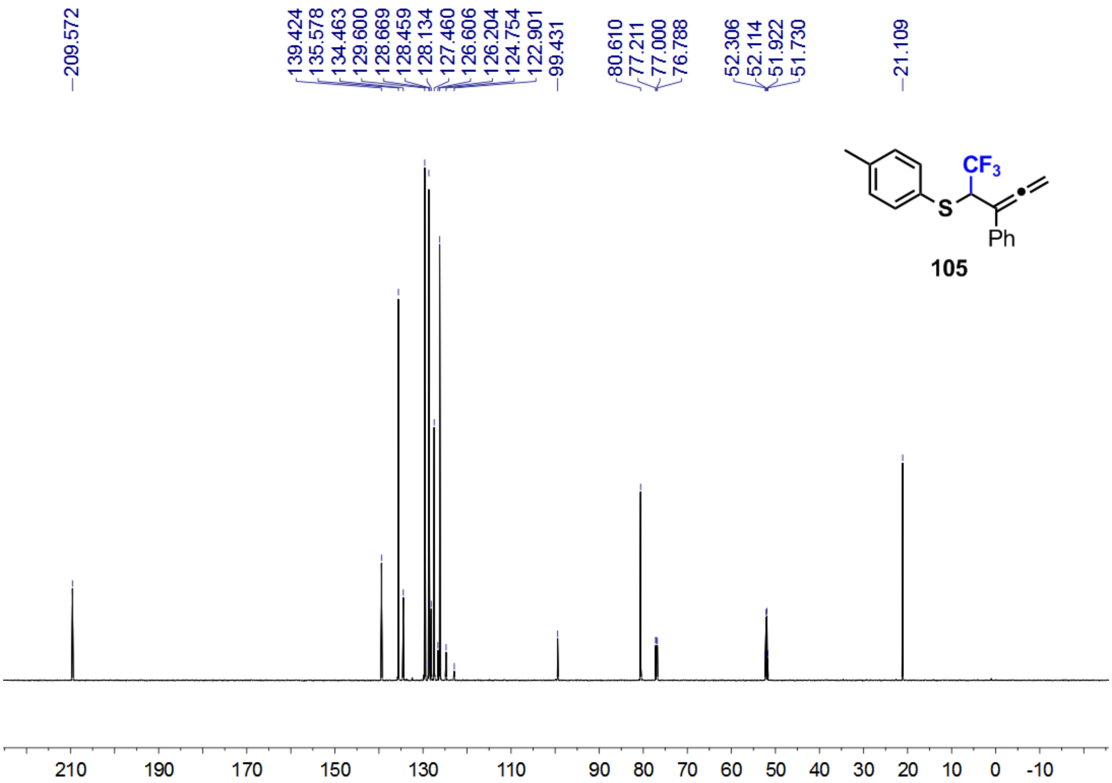

Supplementary Figure 310. ^{13}C NMR of 105

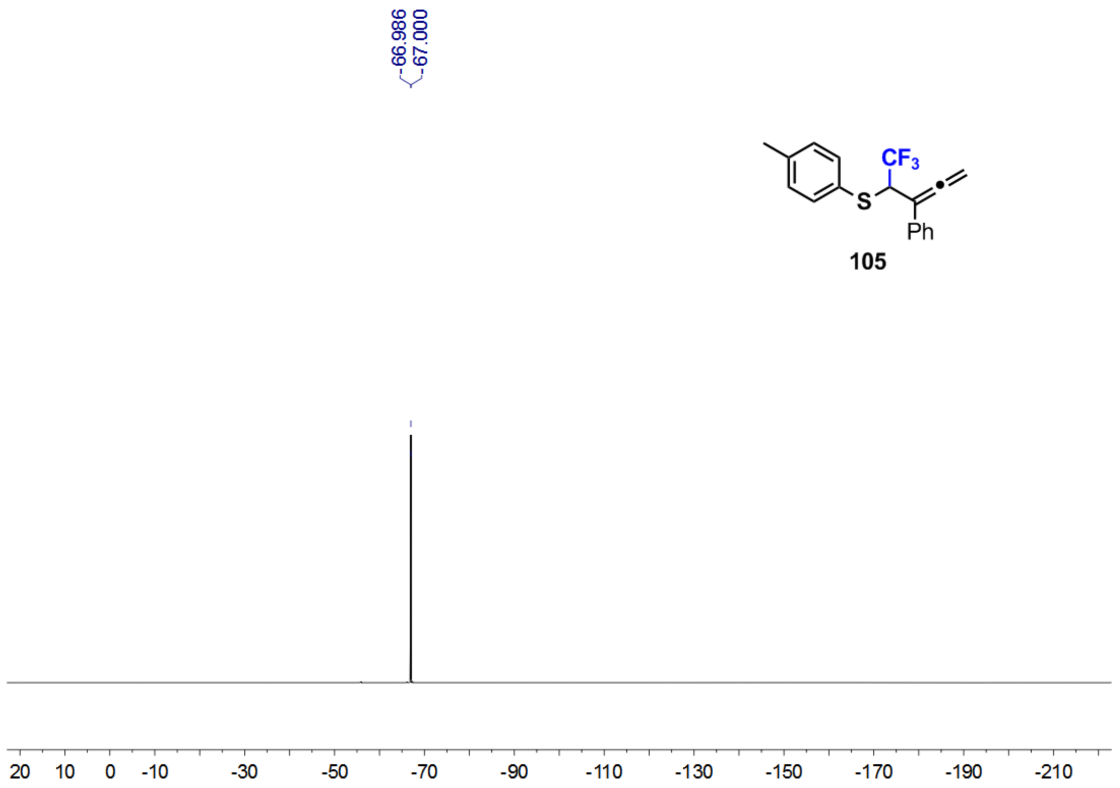

Supplementary Figure 311. ^{19}F NMR of 105

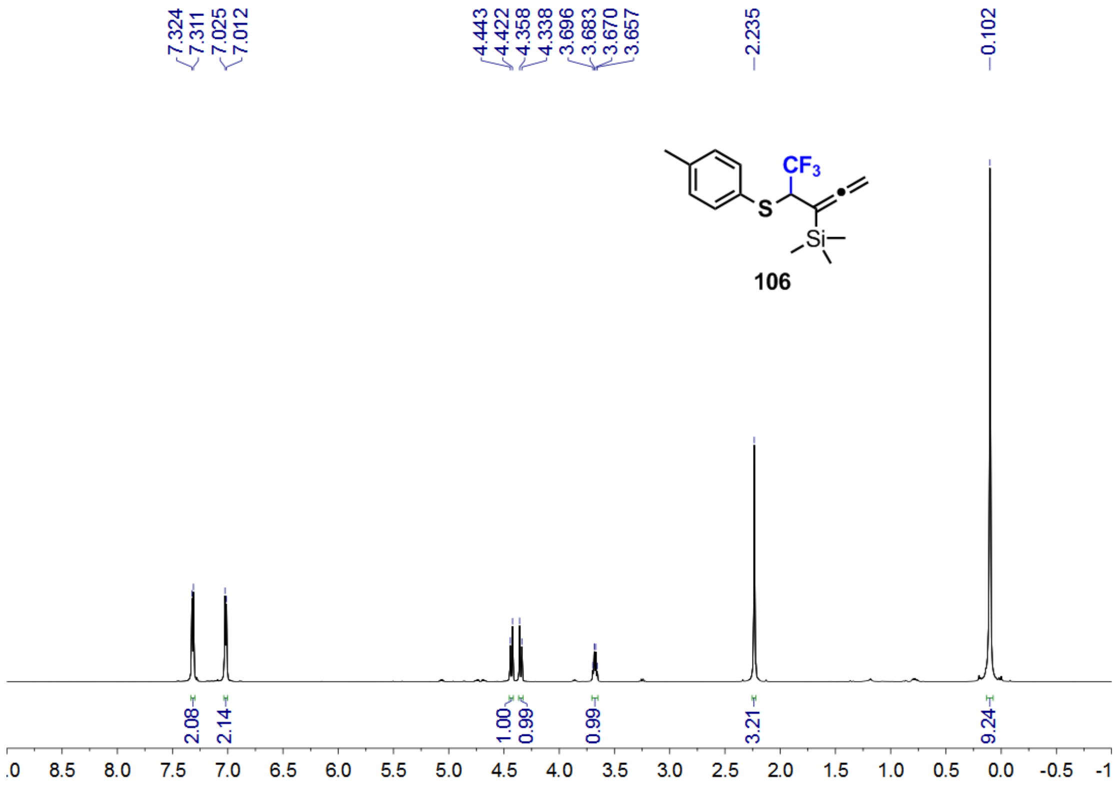

Supplementary Figure 312. ^{1}H NMR of 106

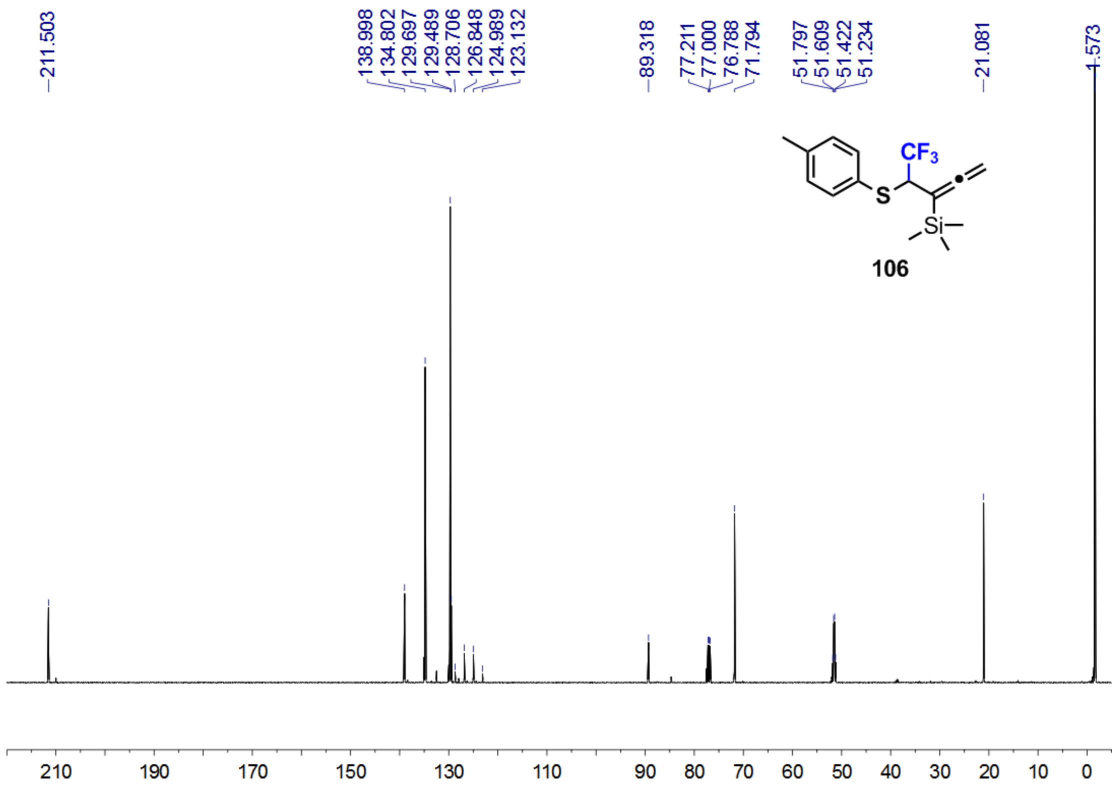

Supplementary Figure 313. ^{13}C NMR of 106

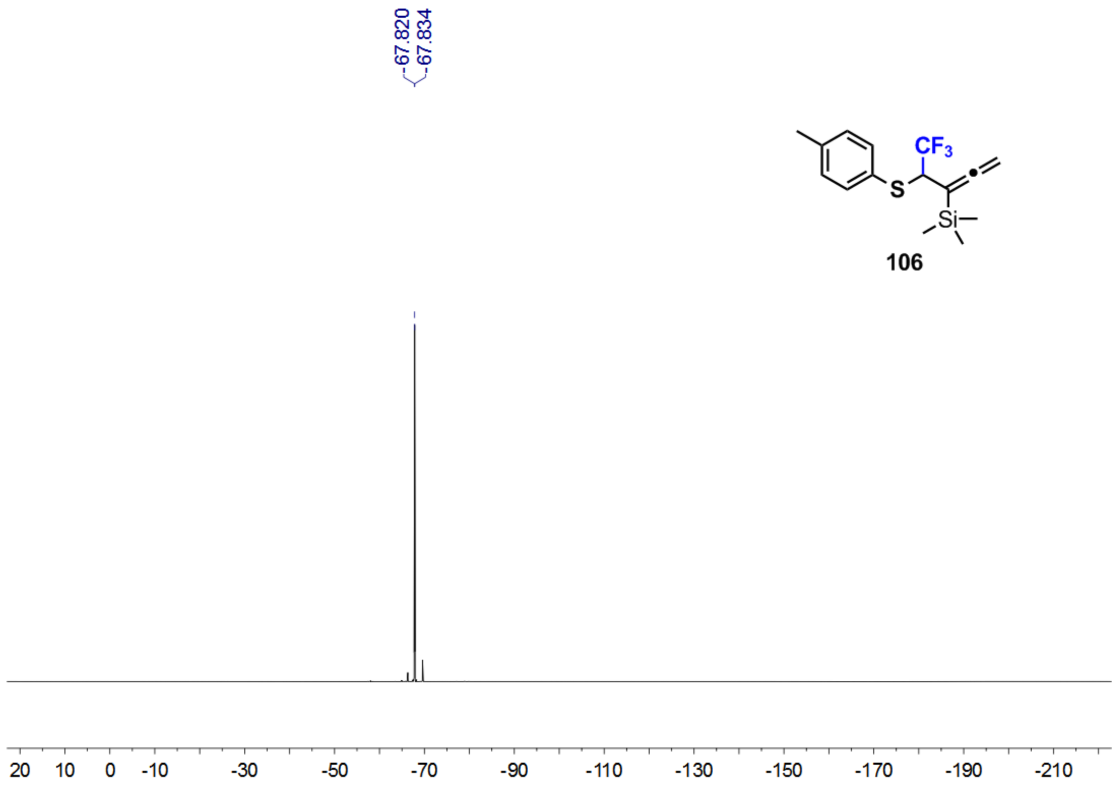

Supplementary Figure 314. ^{19}F NMR of 106

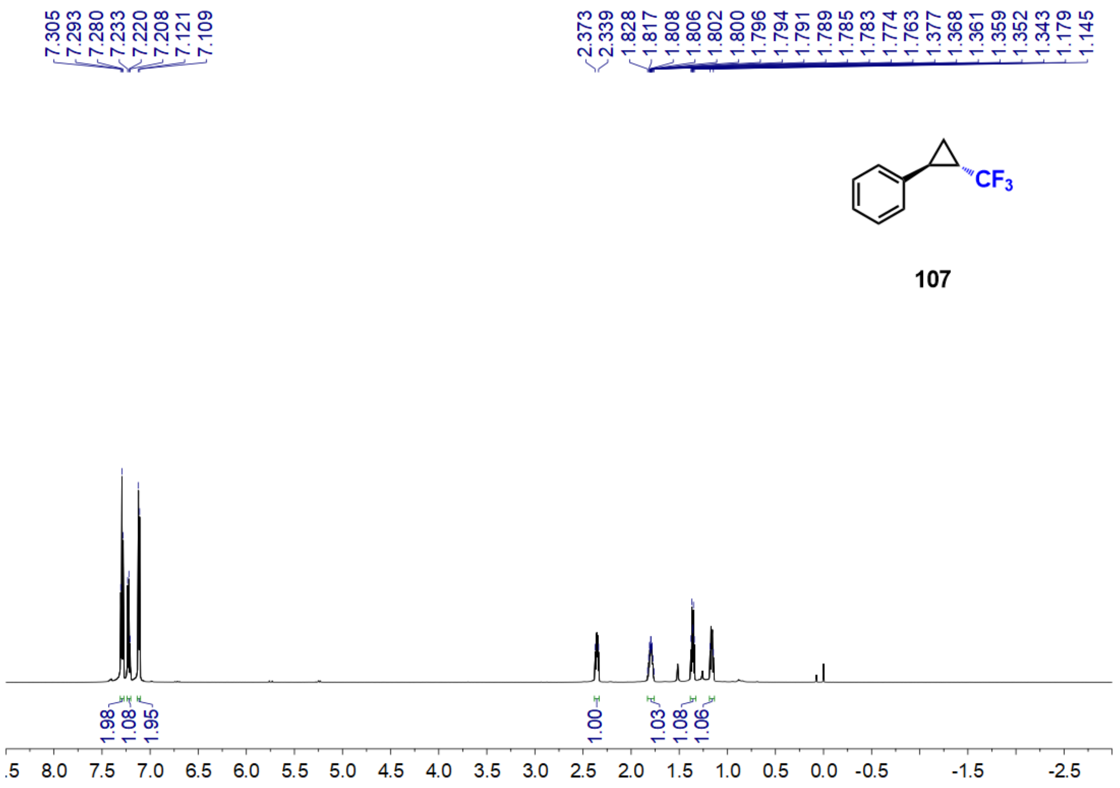

Supplementary Figure 315. ^{1}H NMR of 107

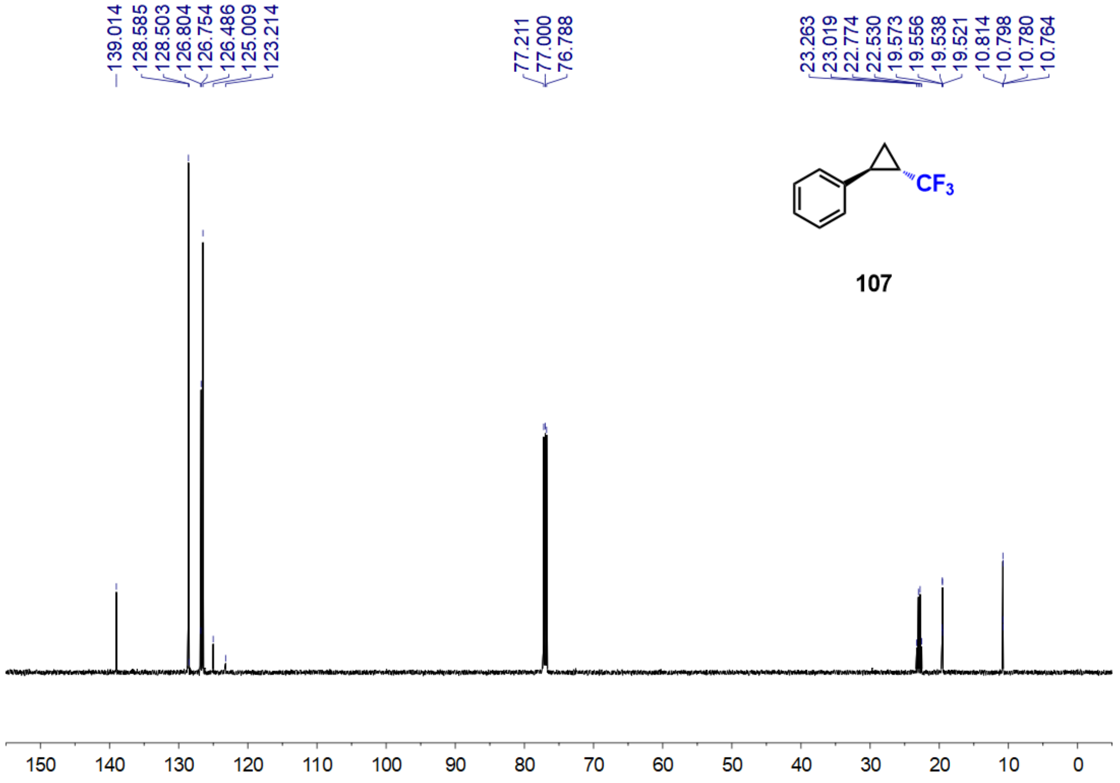

Supplementary Figure 316. ^{13}C NMR of 107

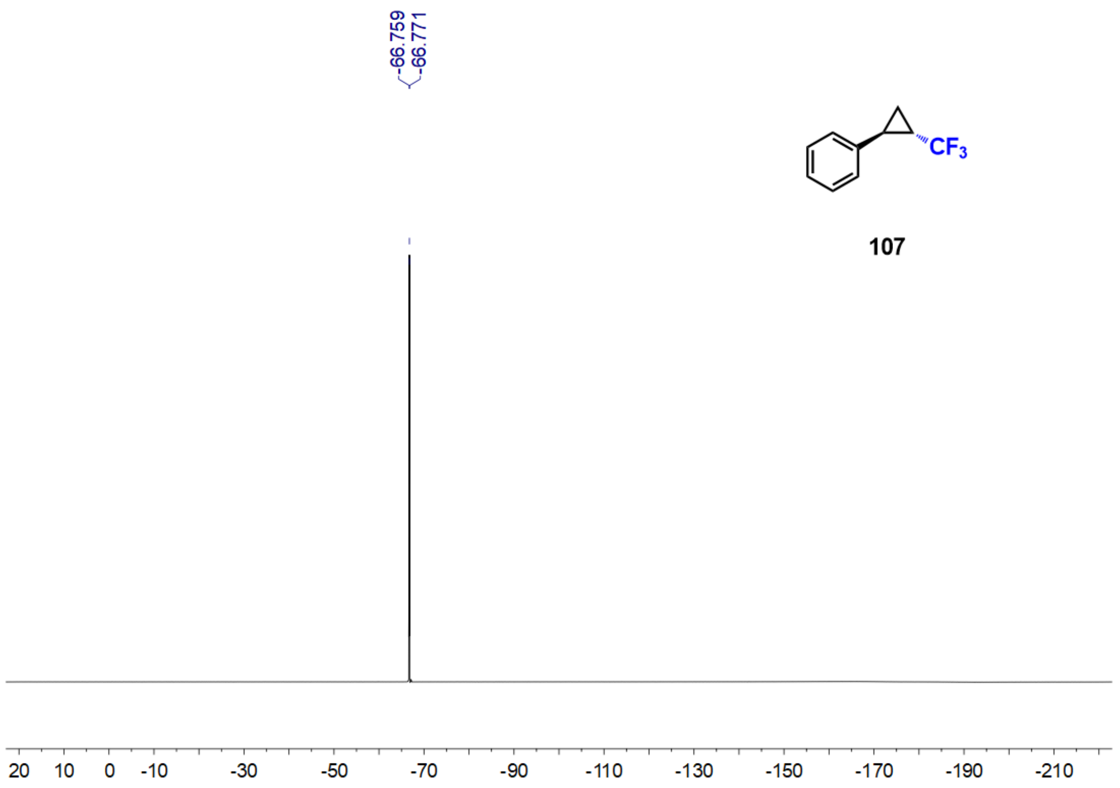

Supplementary Figure 317. ^{19}F NMR of 107

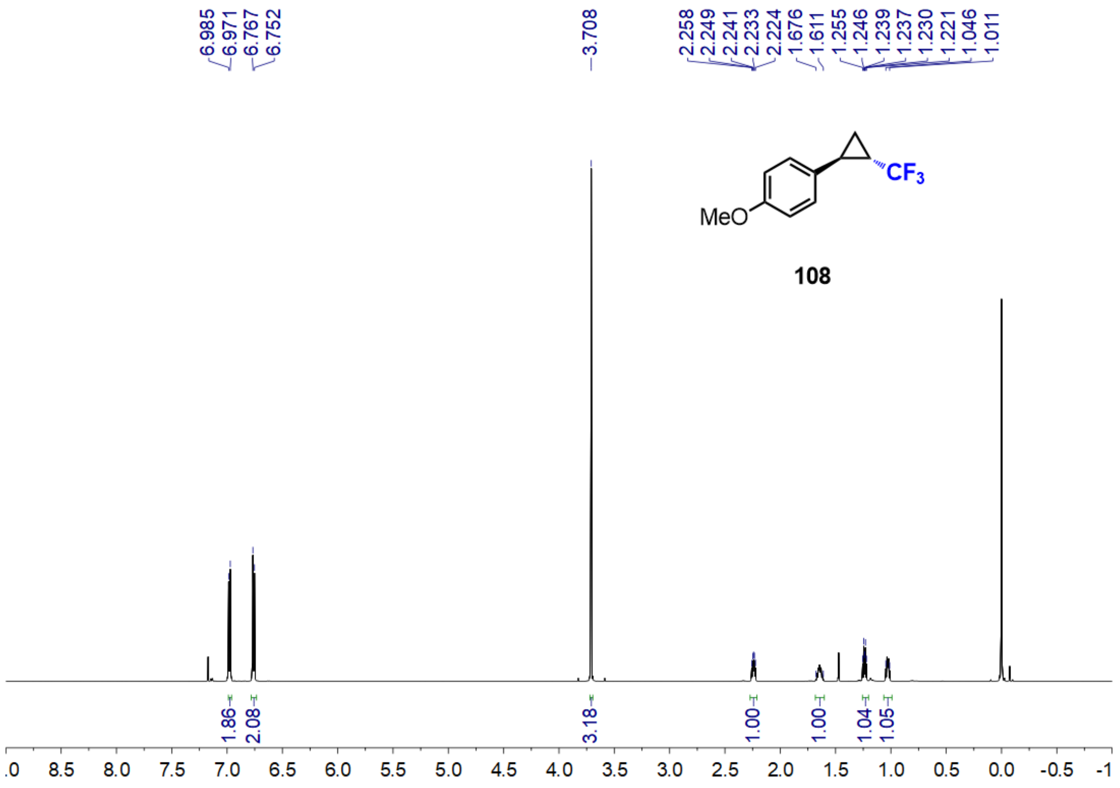

Supplementary Figure 318. ^{1}H NMR of 108

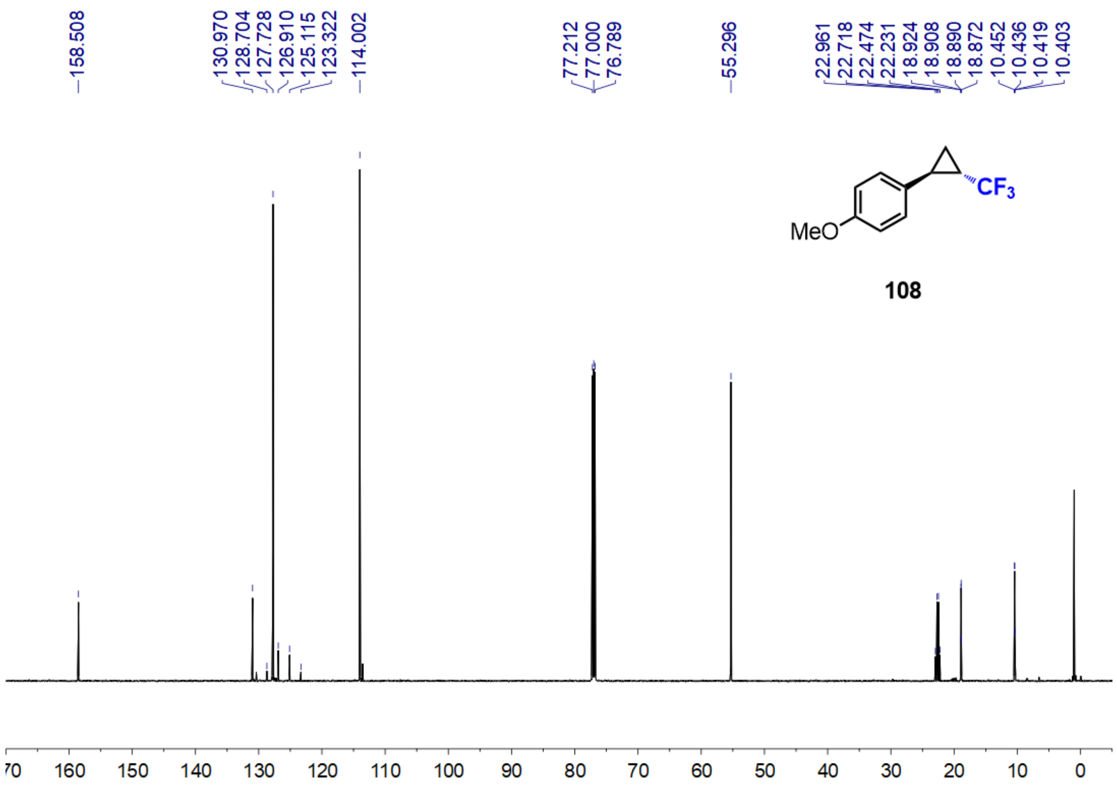

Supplementary Figure 319. ^{13}C NMR of 108

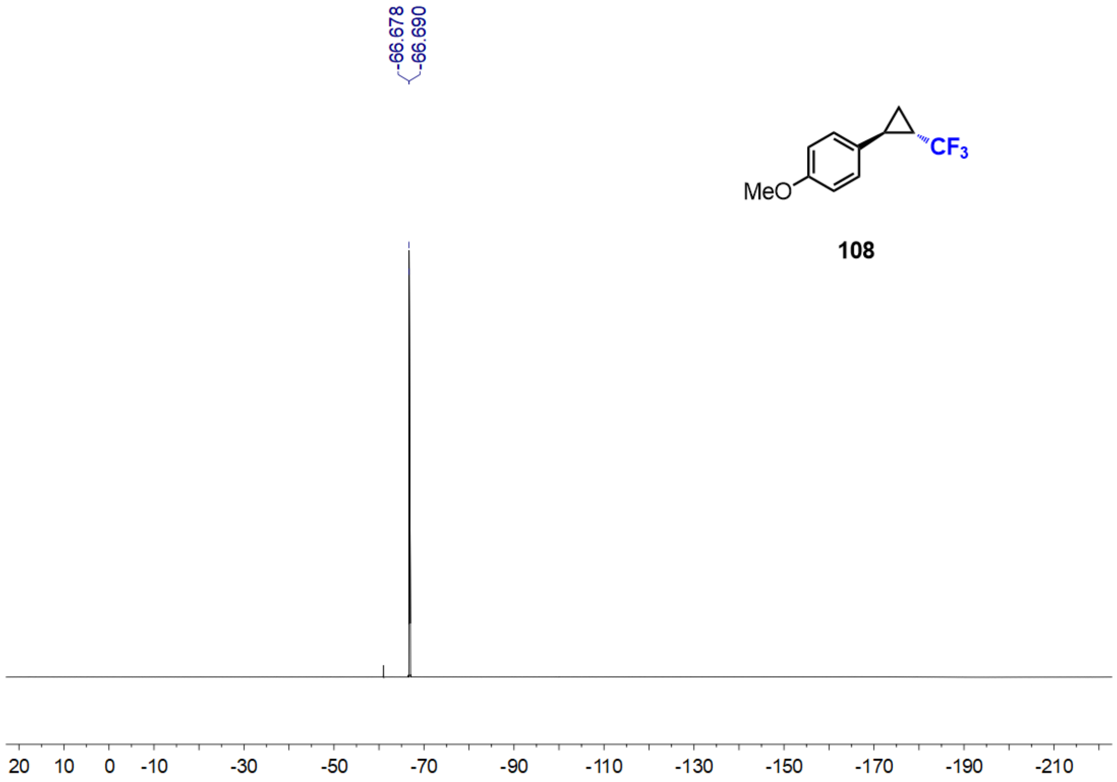

Supplementary Figure 320. ^{19}F NMR of 108

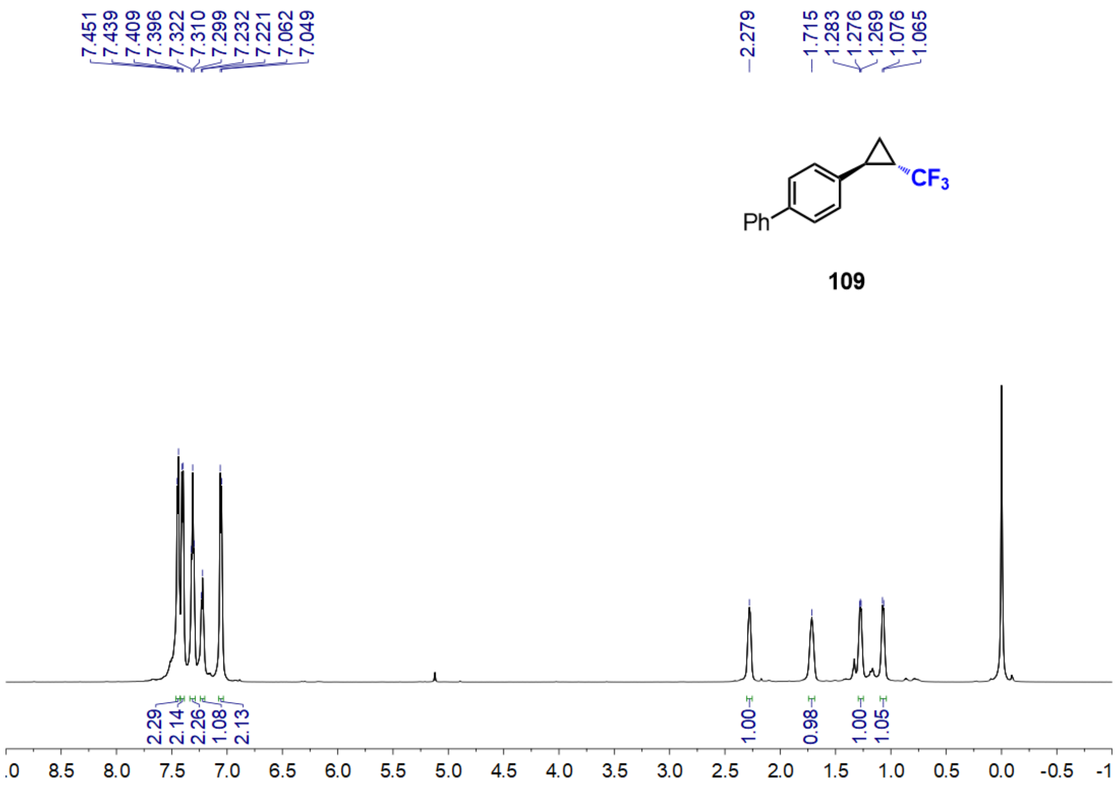

Supplementary Figure 321. ^{1}H NMR of 109

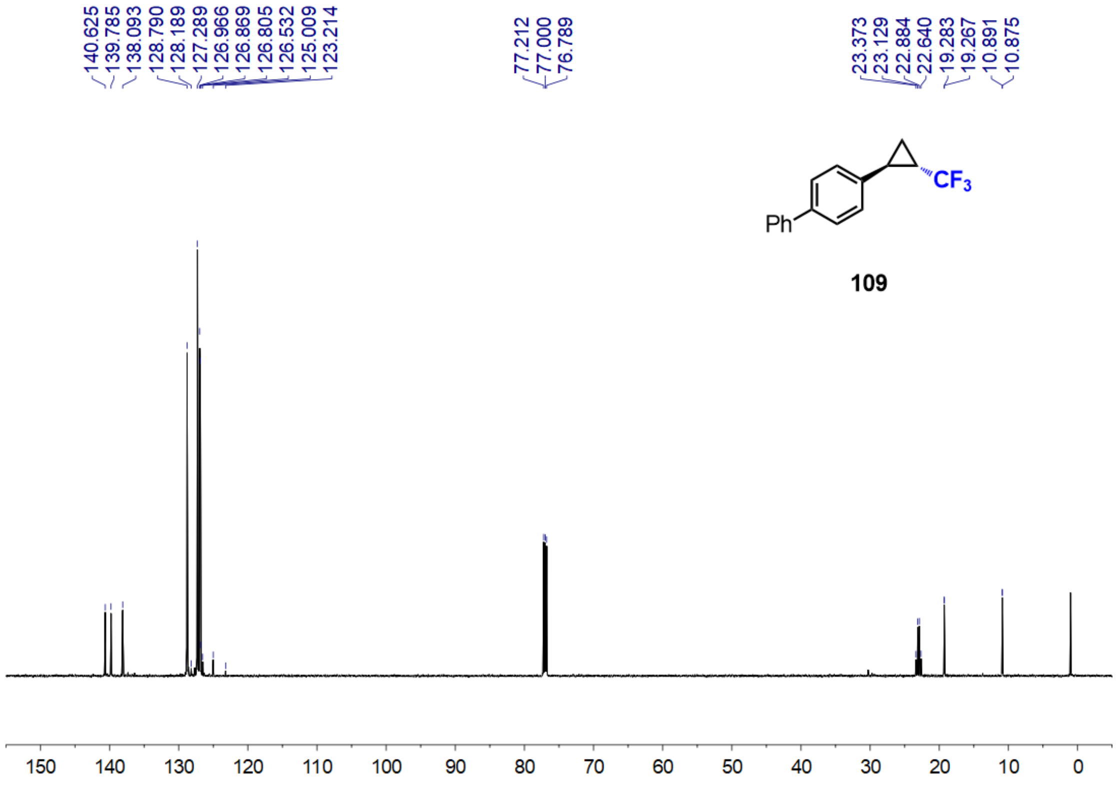

Supplementary Figure 322. ^{13}C NMR of 109

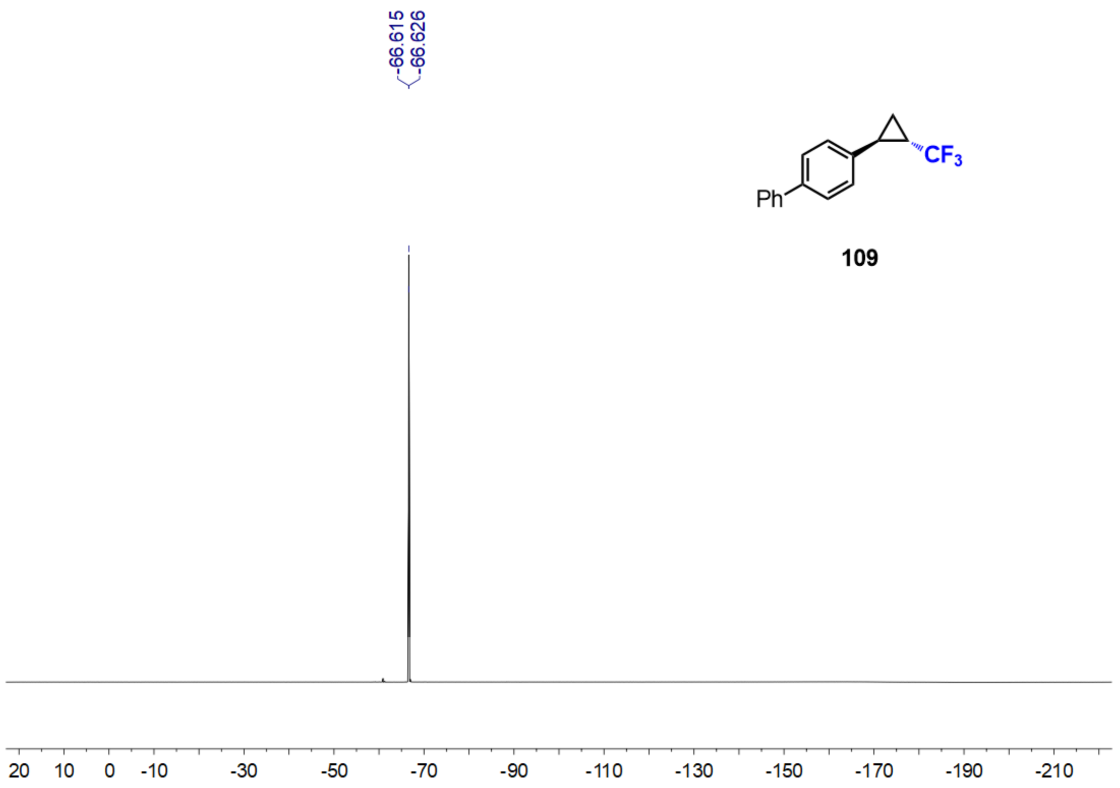

Supplementary Figure 323. ^{19}F NMR of 109

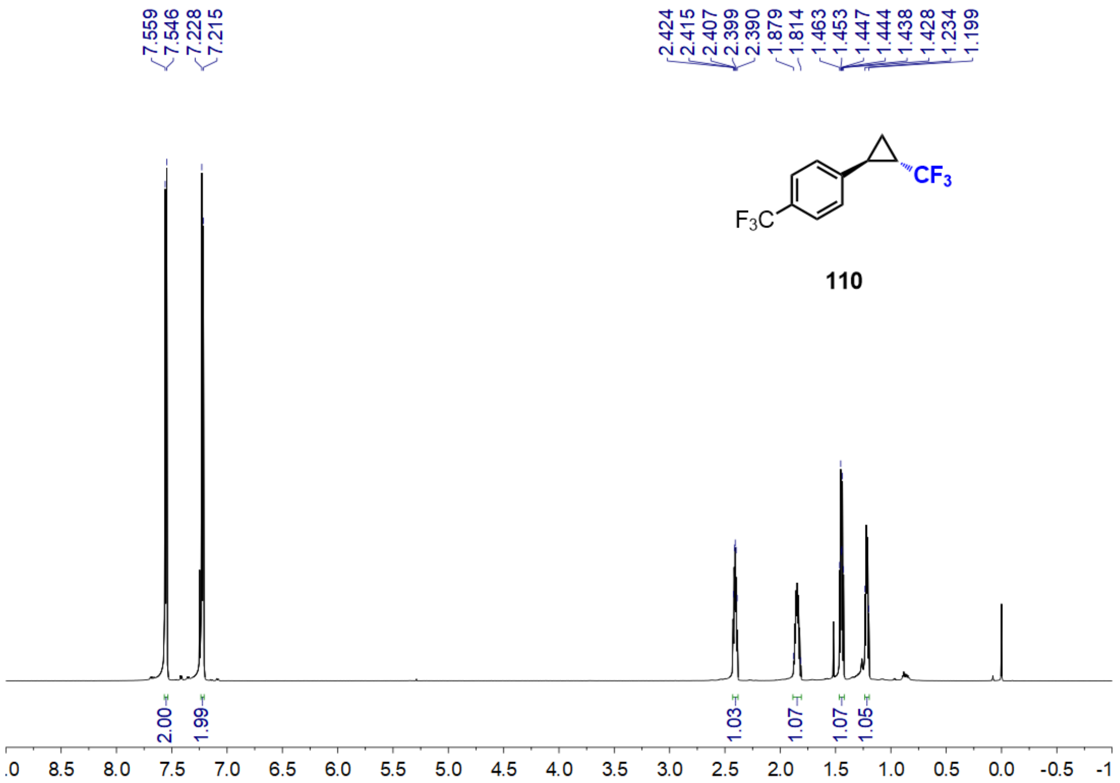

Supplementary Figure 324. ^{1}H NMR of 110

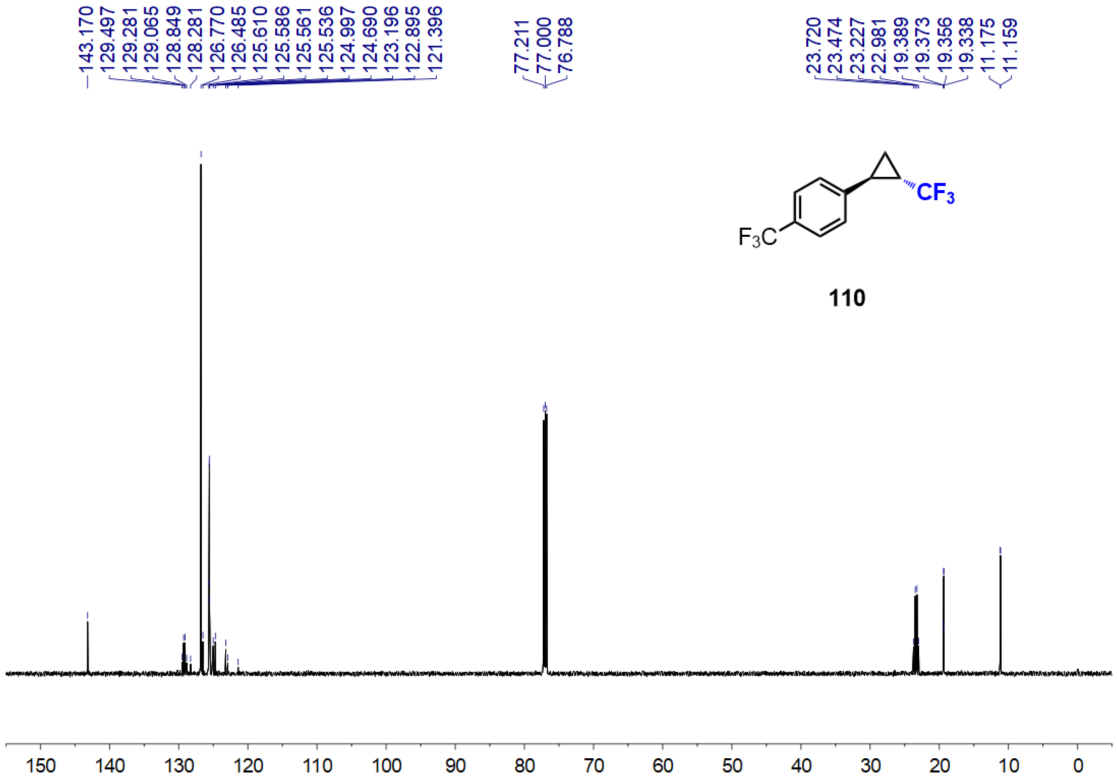

Supplementary Figure 325. ^{13}C NMR of 110

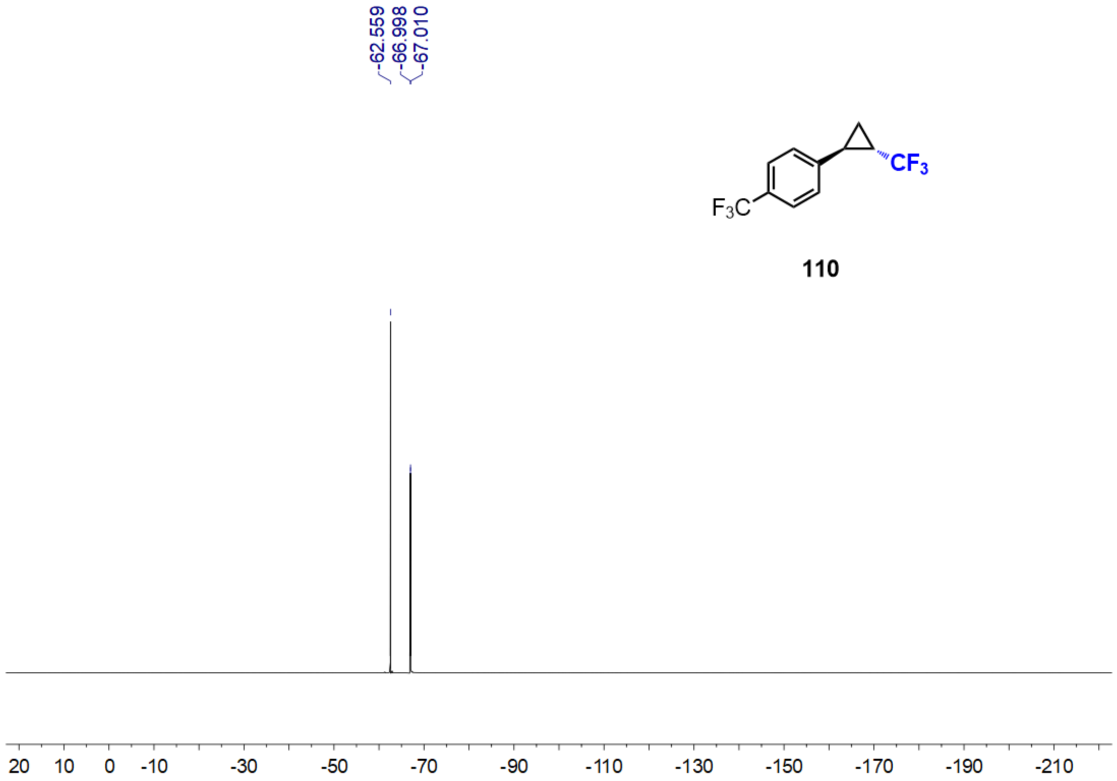

Supplementary Figure 326. ^{19}F NMR of 110

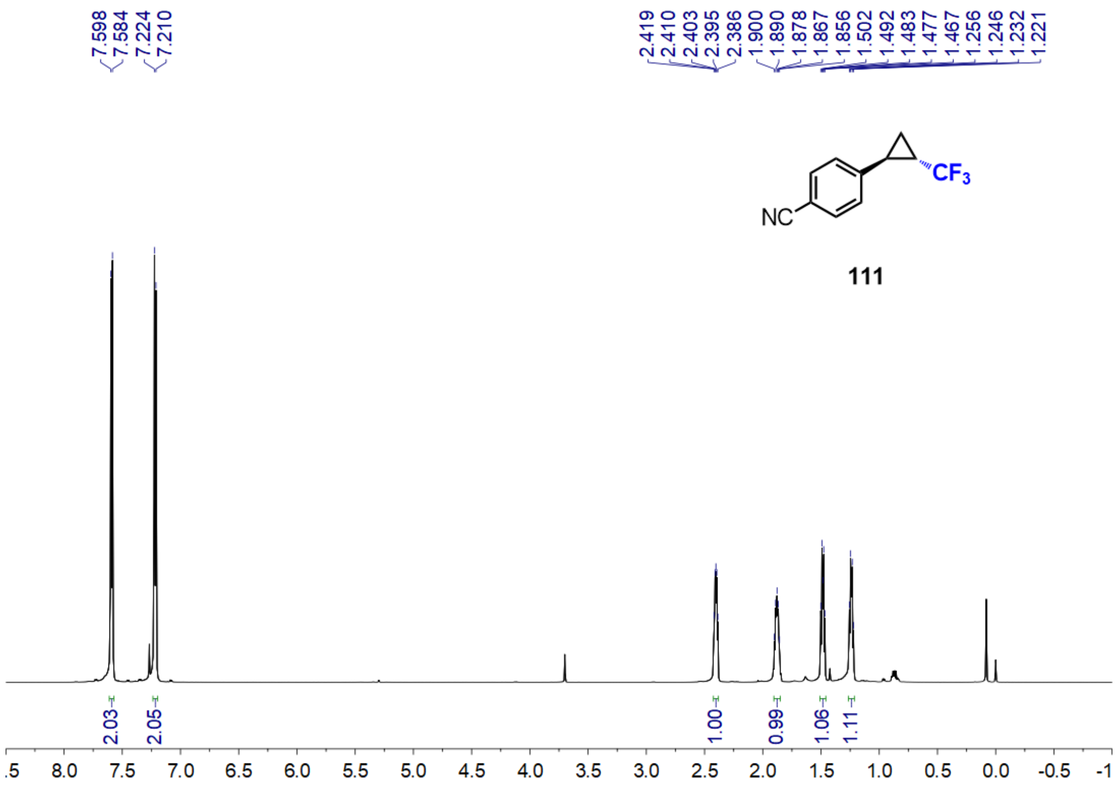

Supplementary Figure 327. ^{1}H NMR of 111

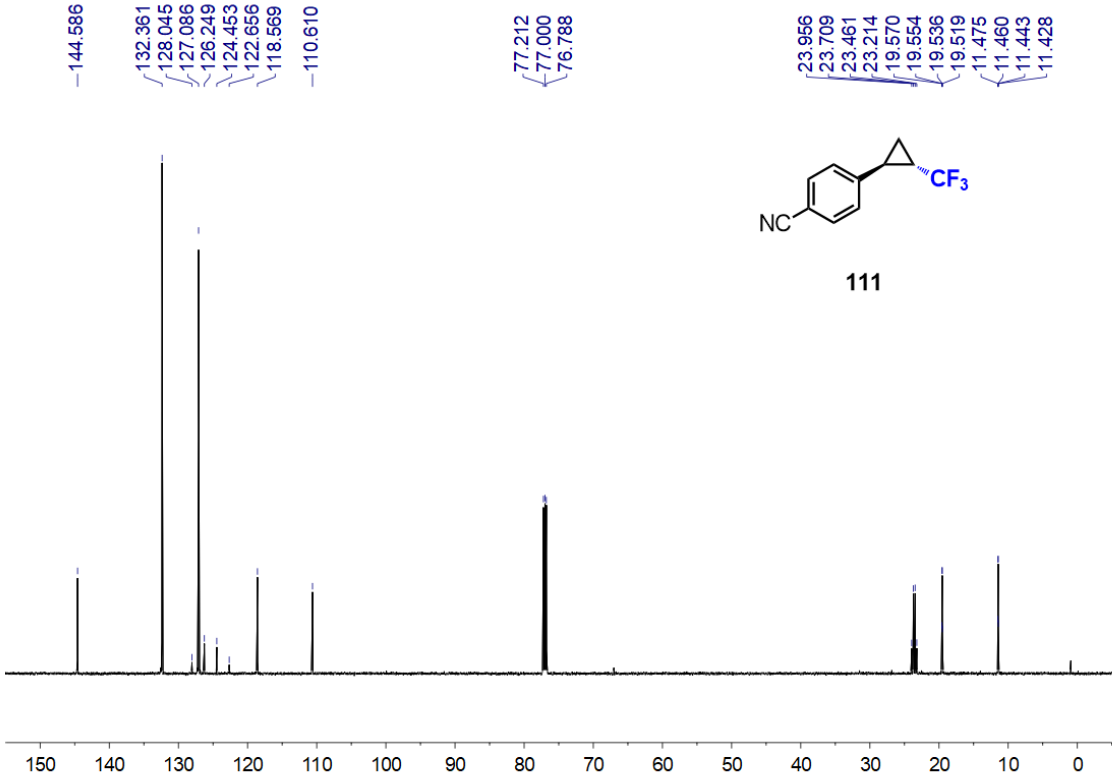

Supplementary Figure 328. ^{13}C NMR of 111

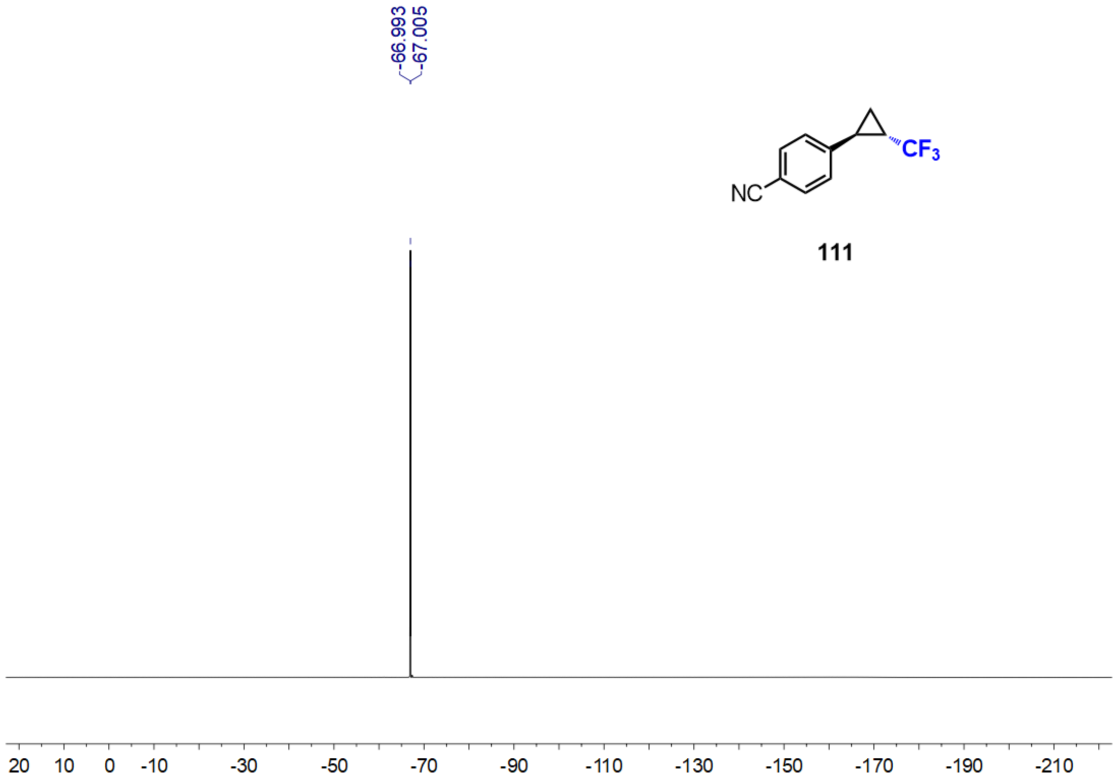

Supplementary Figure 329. ^{19}F NMR of 111

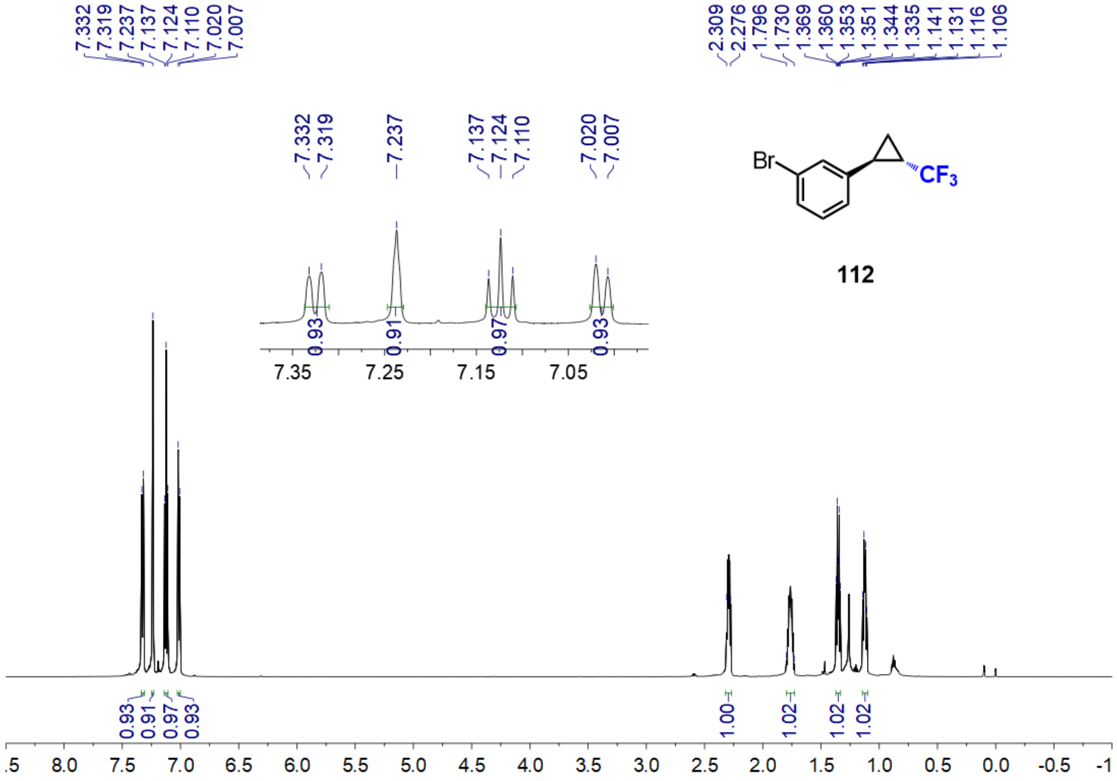

Supplementary Figure 330. ^{1}H NMR of 112

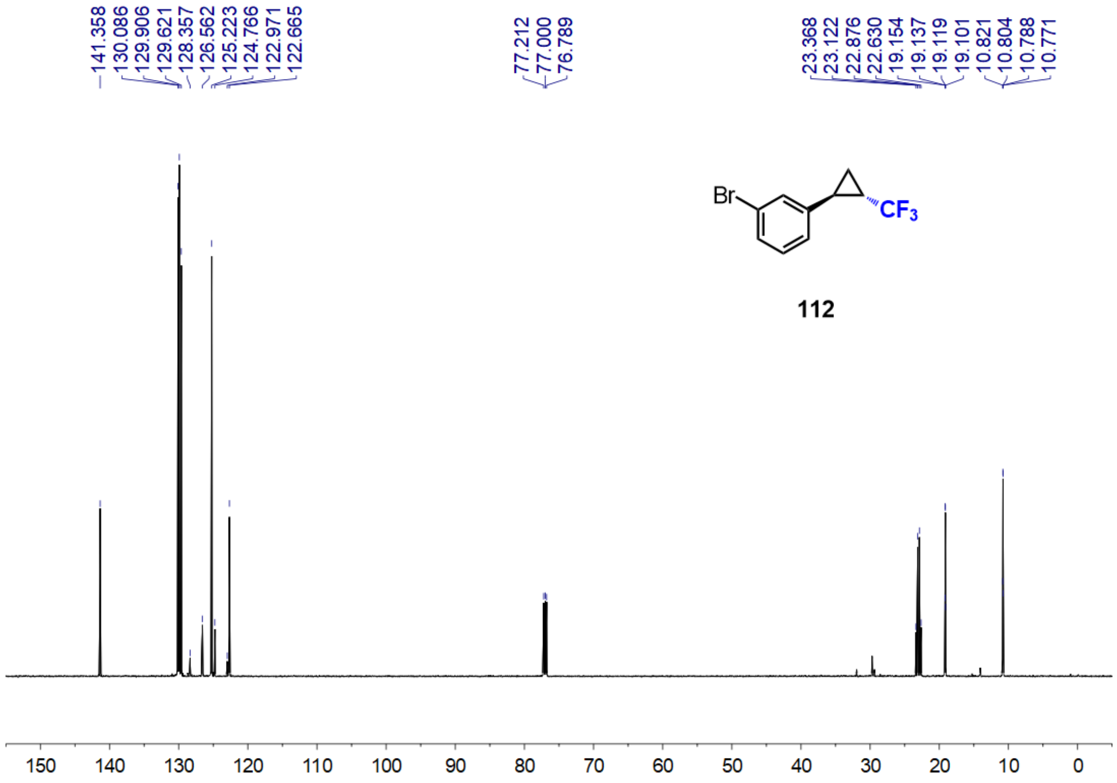

Supplementary Figure 331. ^{13}C NMR of 112

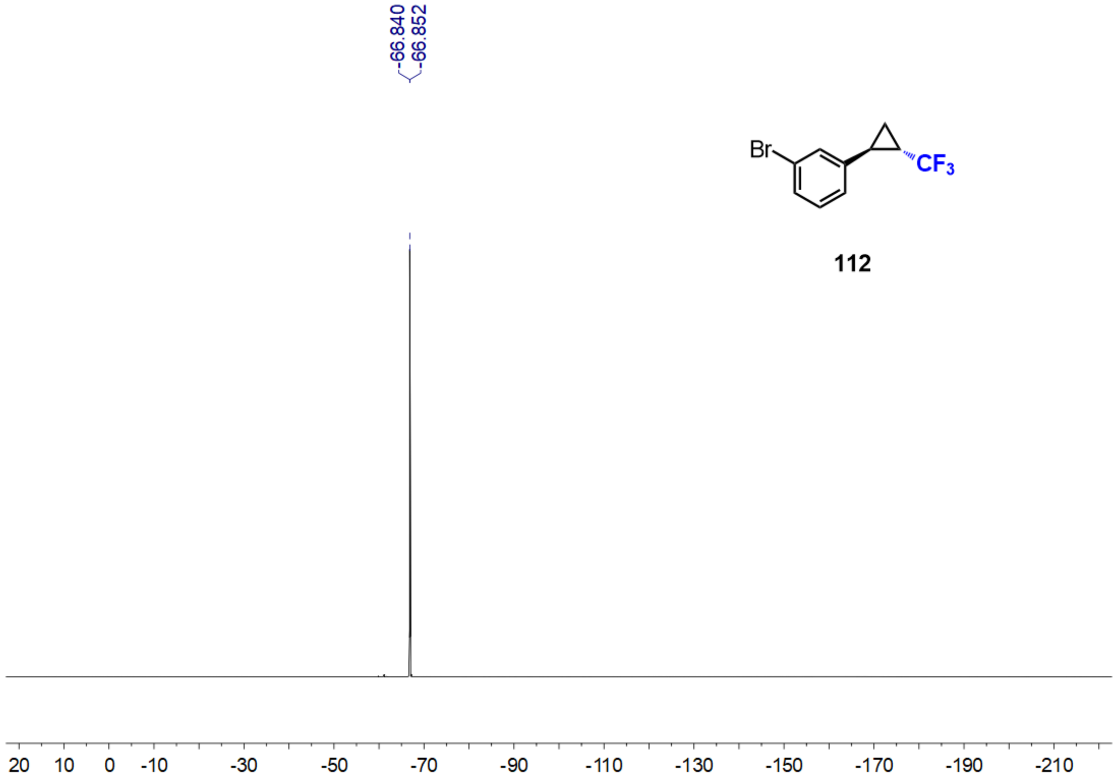

Supplementary Figure 332. ^{19}F NMR of 112

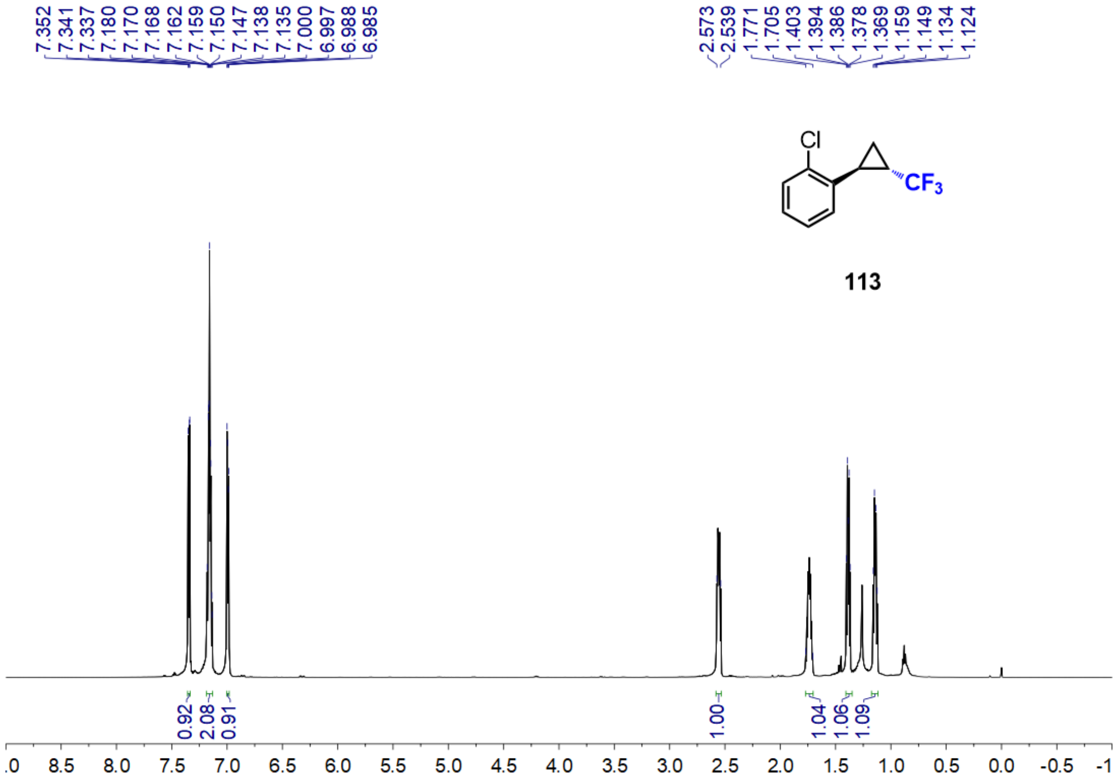

Supplementary Figure 333. ^{1}H NMR of 113

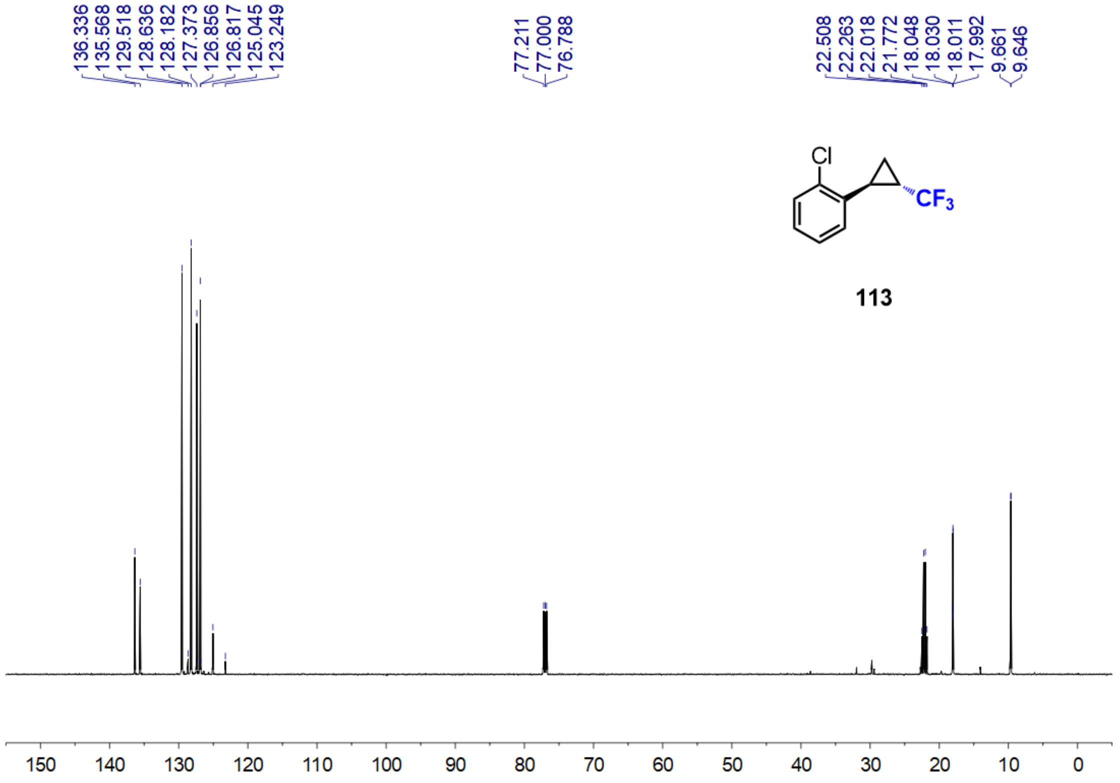

Supplementary Figure 334. ^{13}C NMR of 113

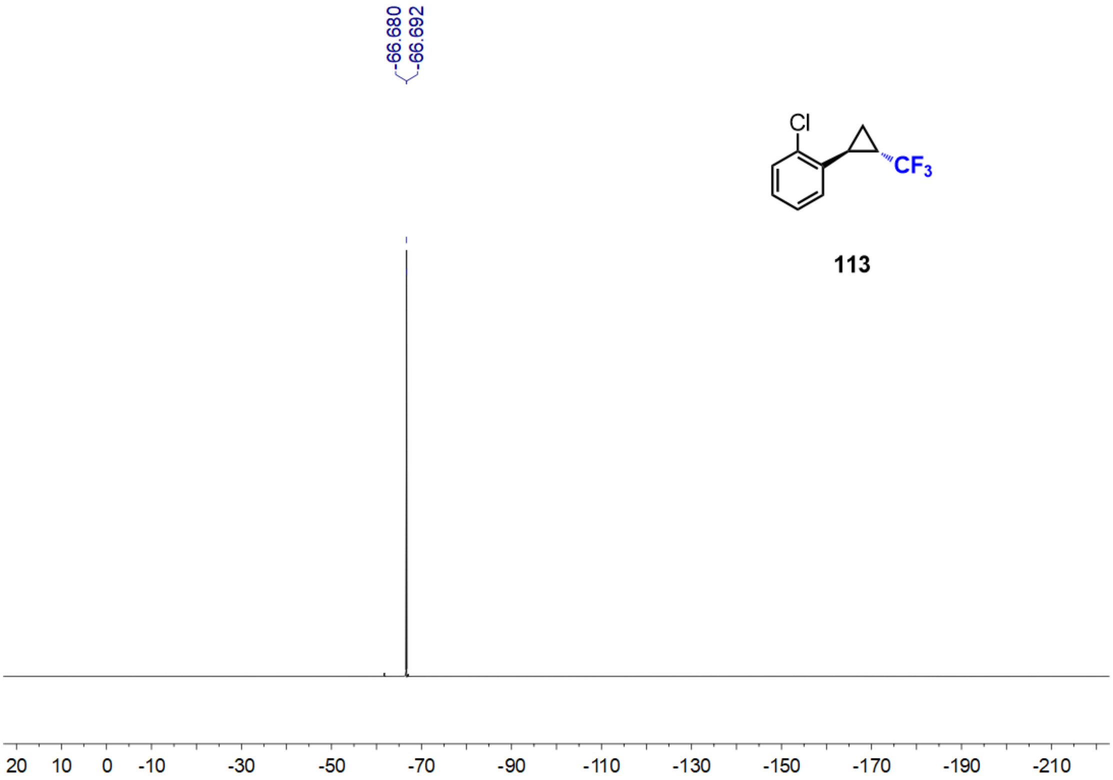

Supplementary Figure 335. ^{19}F NMR of 113

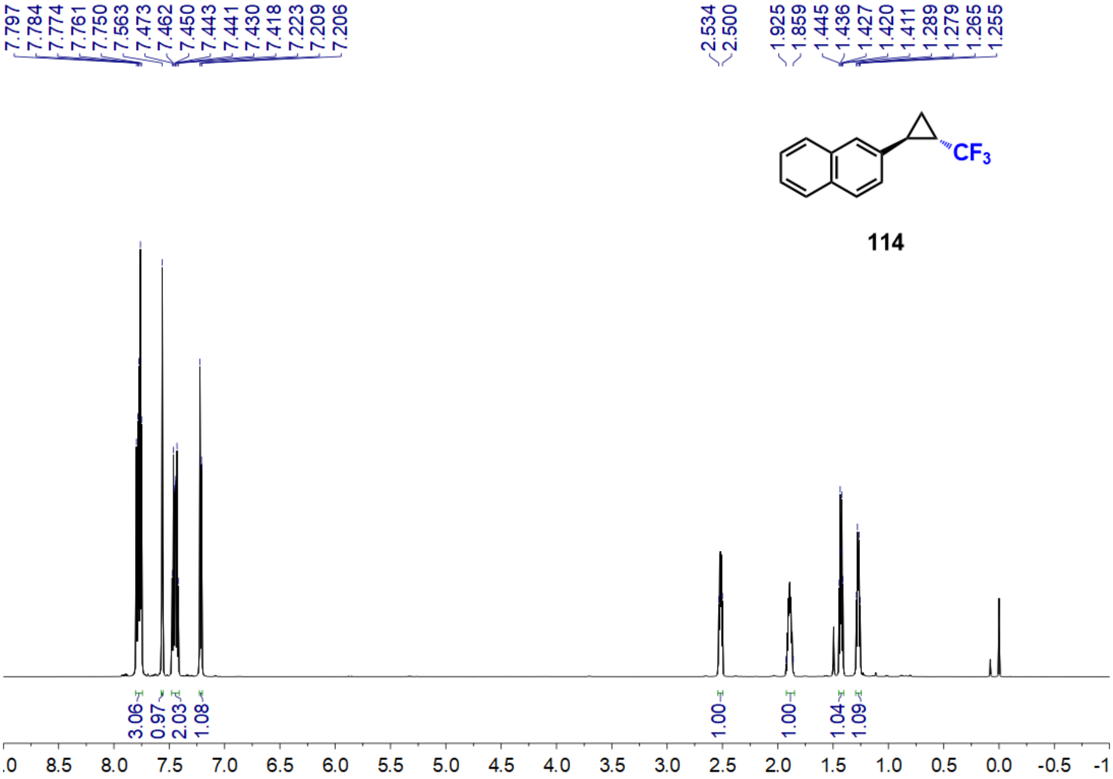

Supplementary Figure 336. ^{1}H NMR of 114

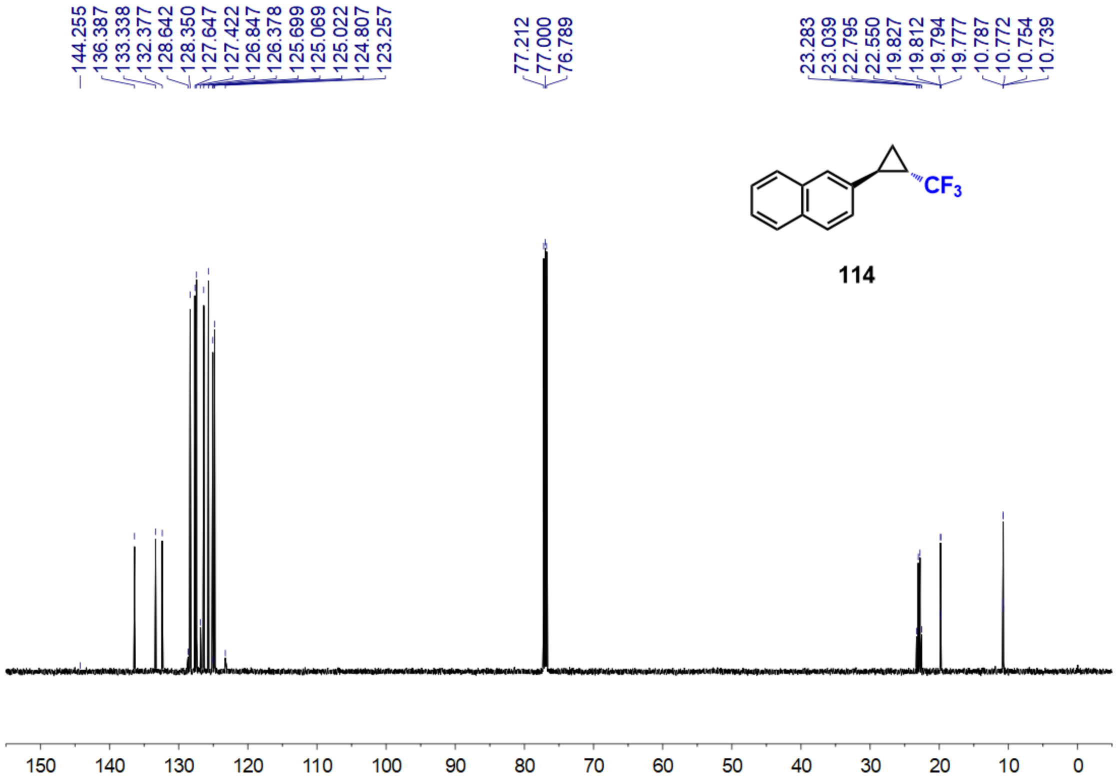

Supplementary Figure 337. ^{13}C NMR of 114

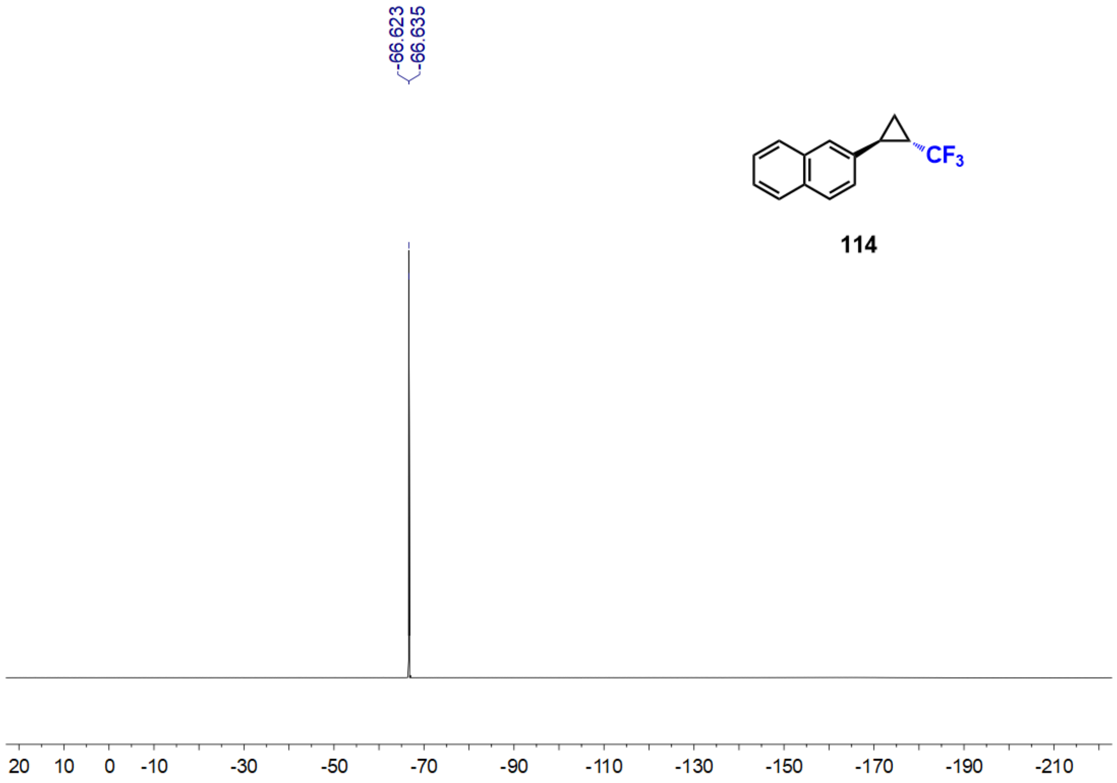

Supplementary Figure 338. ^{19}F NMR of 114

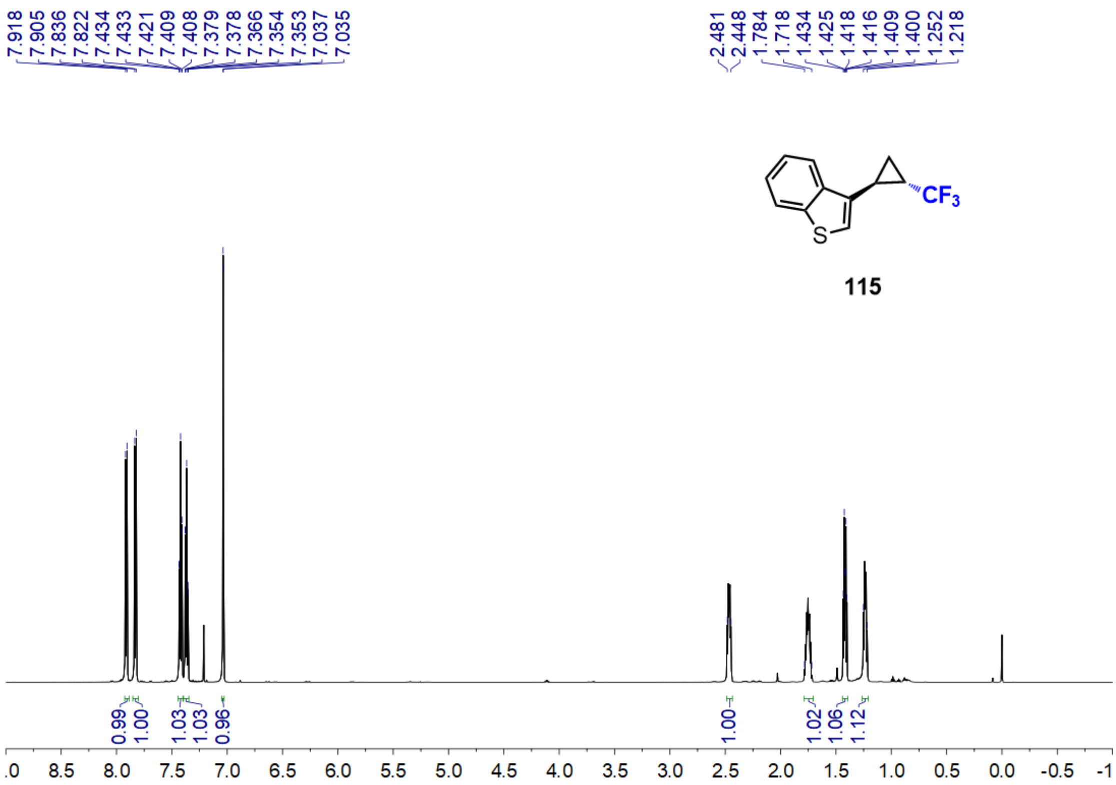

Supplementary Figure 339. ^{1}H NMR of 115

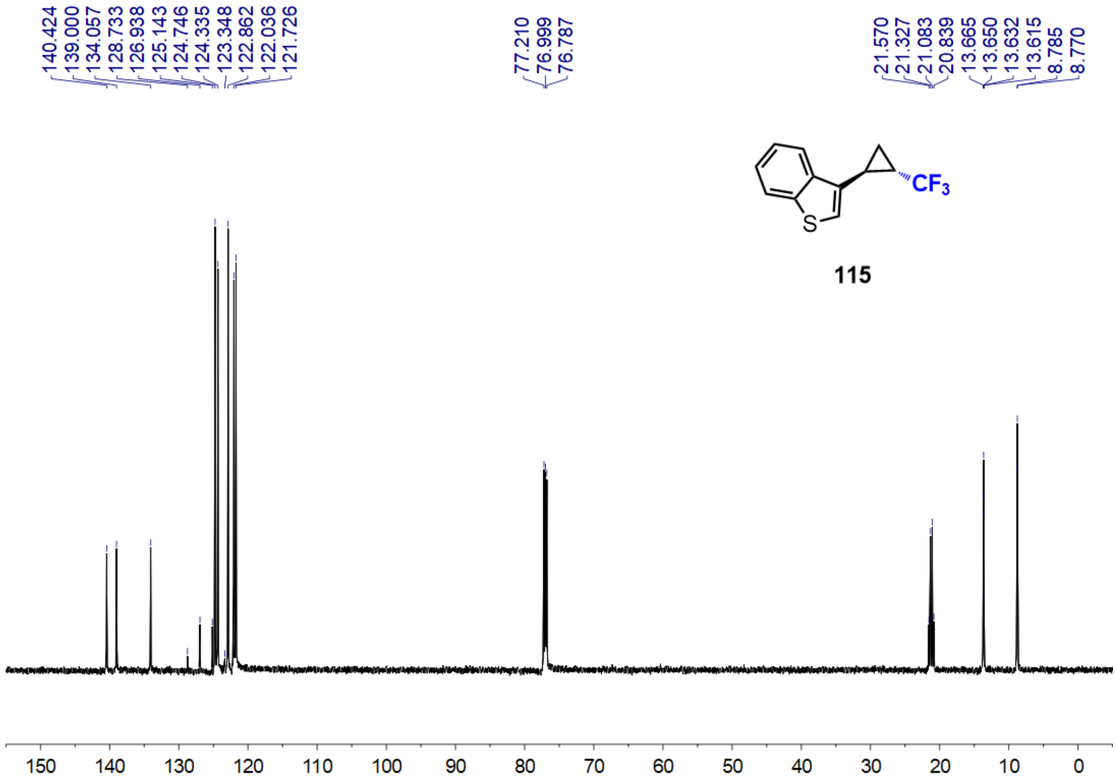

Supplementary Figure 340. ^{13}C NMR of 115

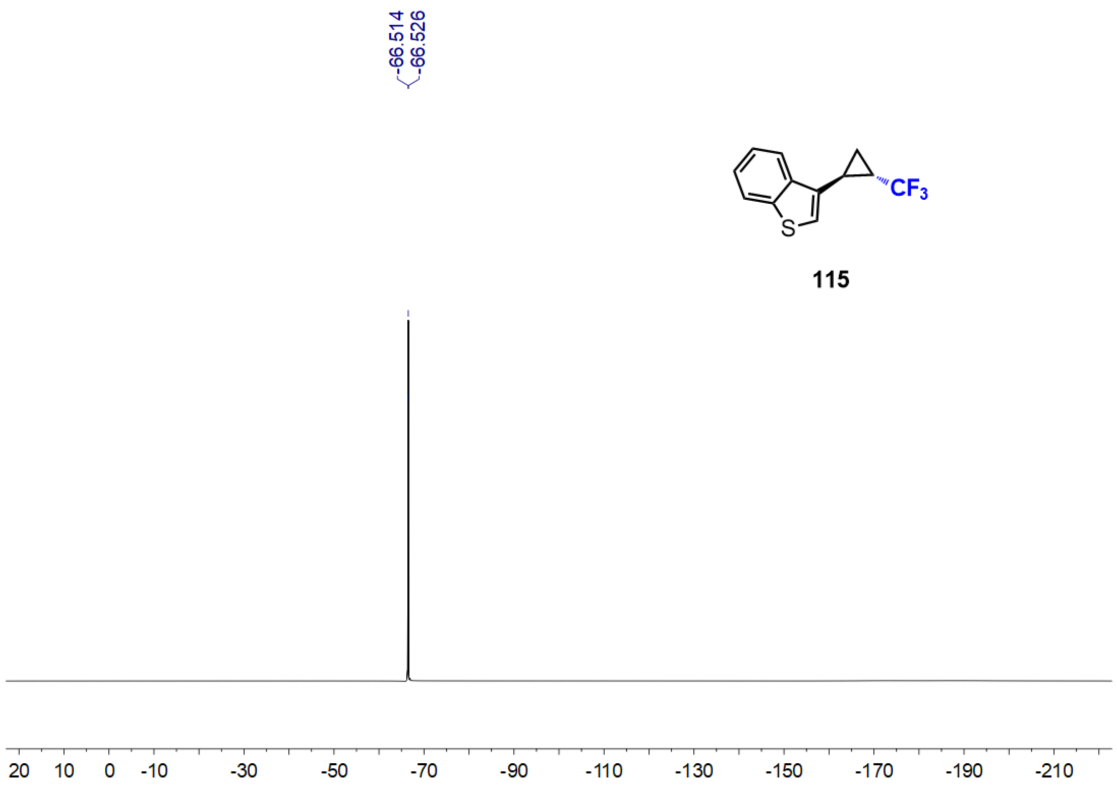

Supplementary Figure 341. ^{19}F NMR of 115

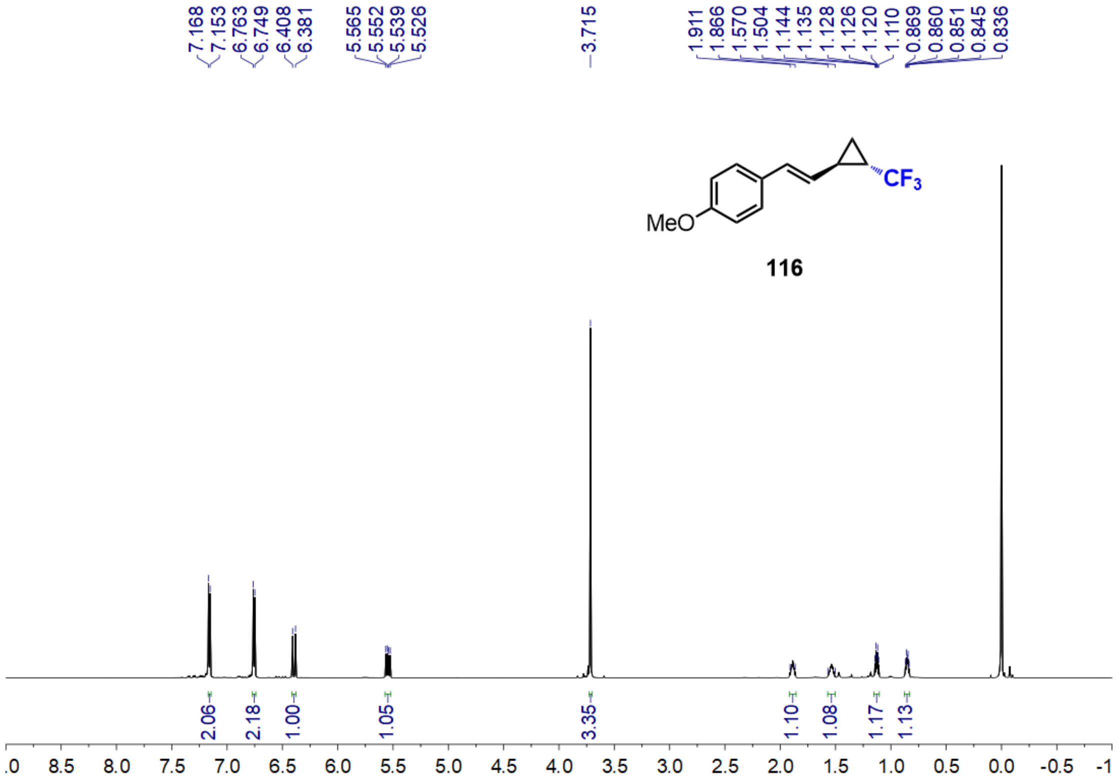

Supplementary Figure 342. ^{1}H NMR of 116

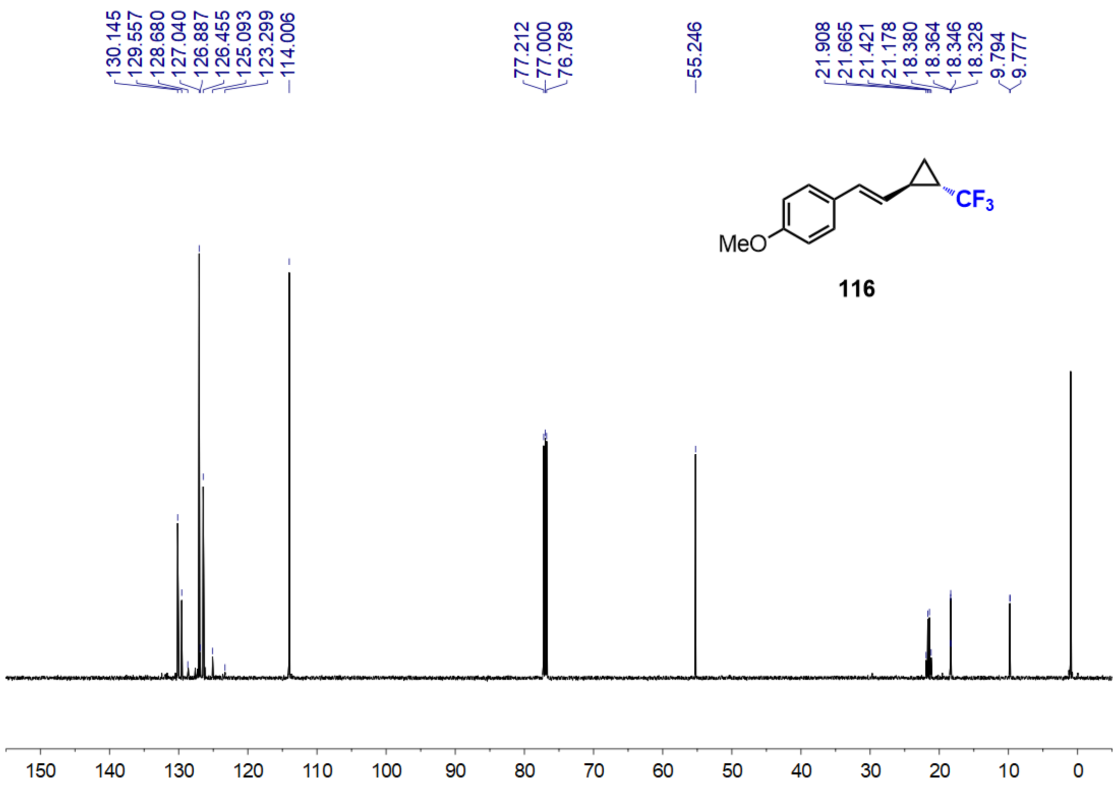

Supplementary Figure 343. ^{13}C NMR of 116

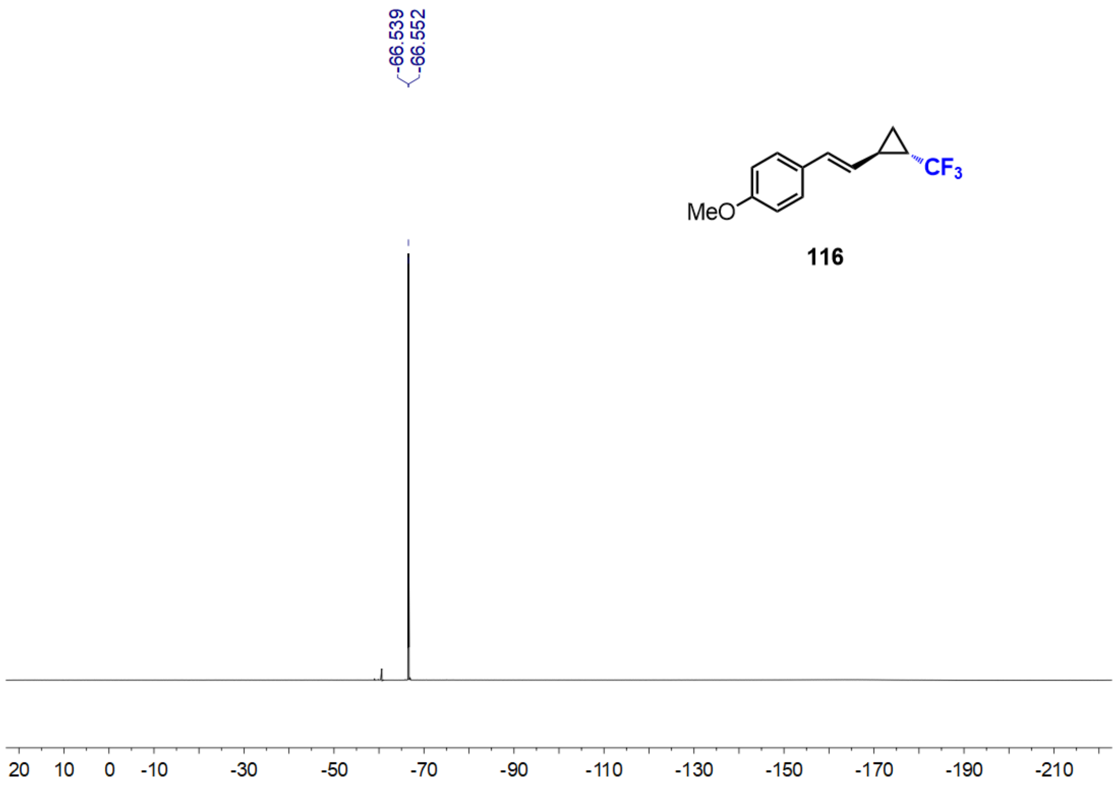

Supplementary Figure 344. ^{19}F NMR of 116

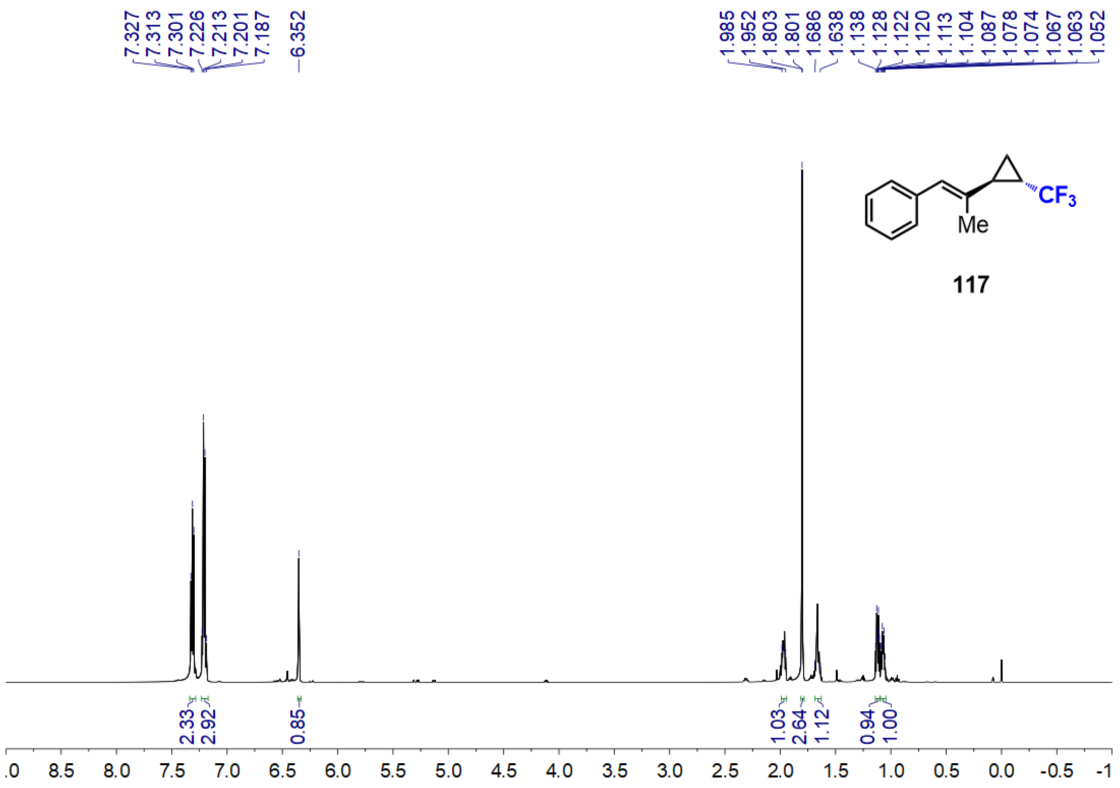

Supplementary Figure 345. ^{1}H NMR of 117

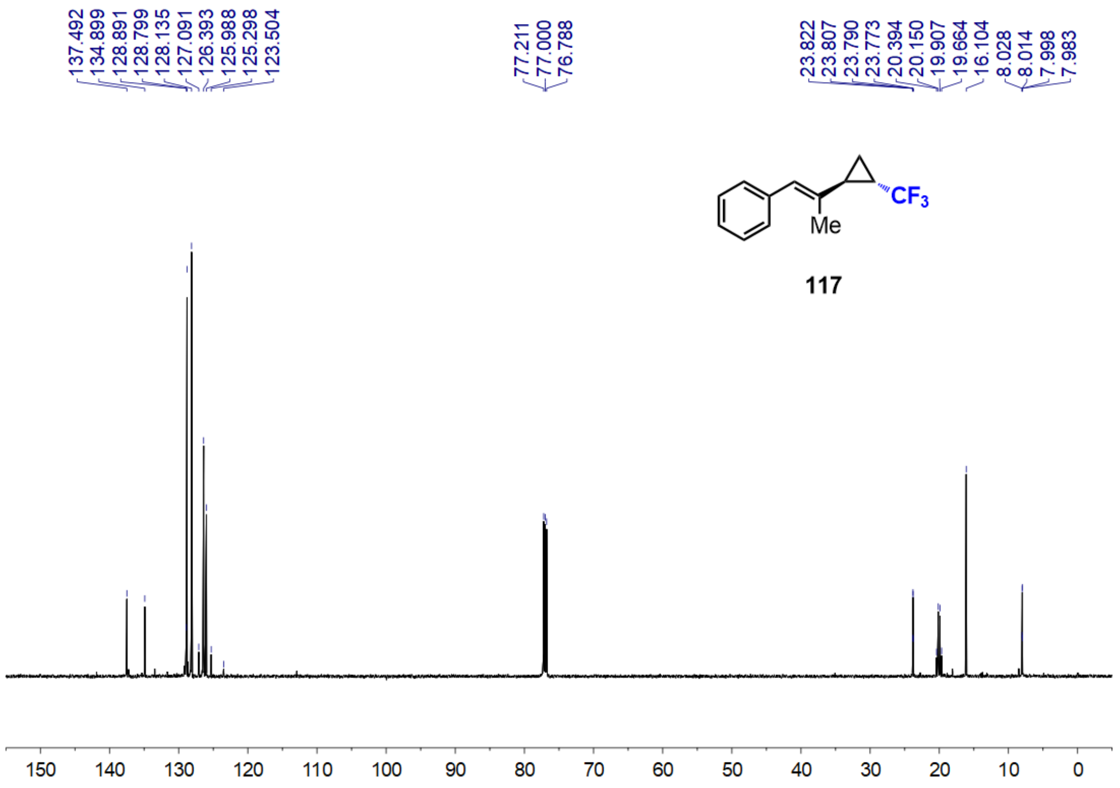

Supplementary Figure 346. ^{13}C NMR of 117

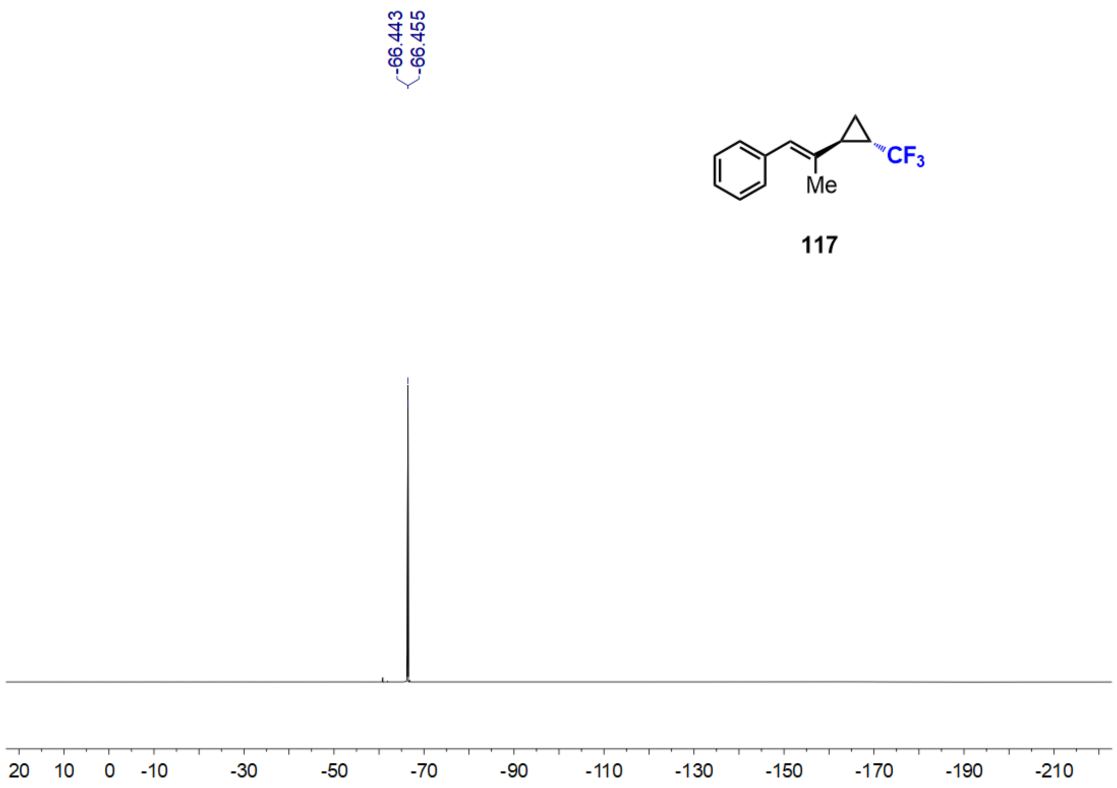

Supplementary Figure 347. ^{19}F NMR of 117

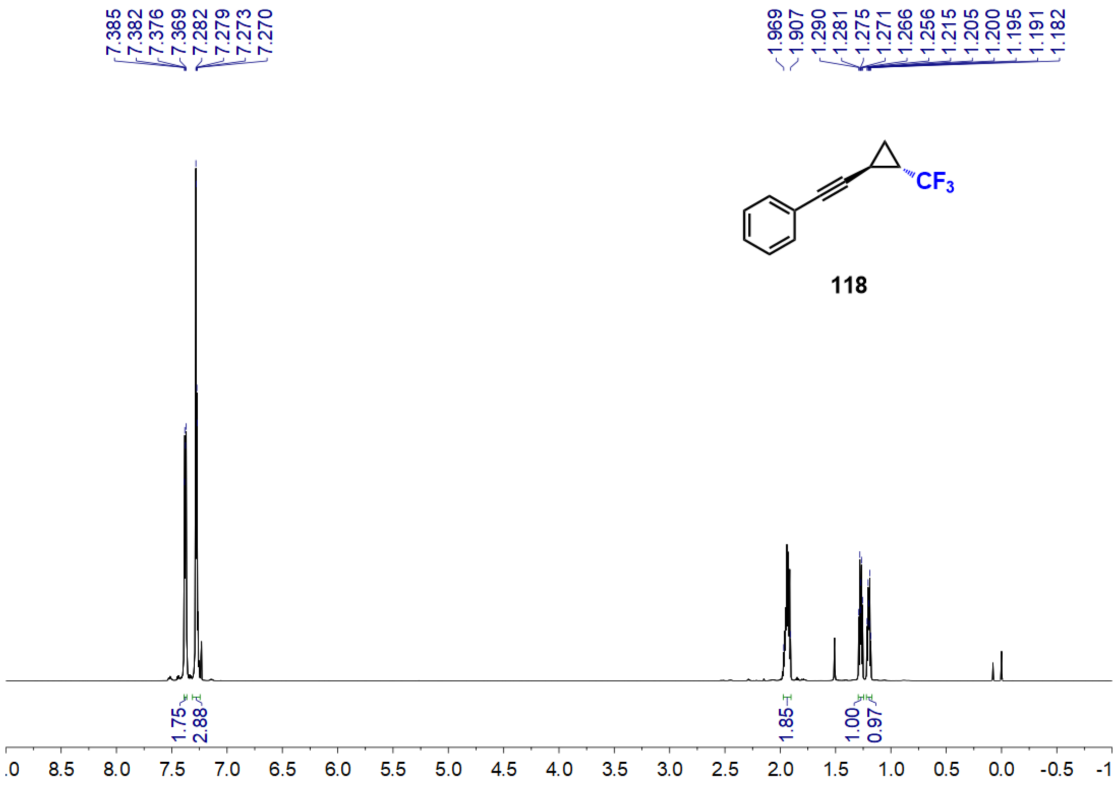

Supplementary Figure 348. ^{1}H NMR of 118

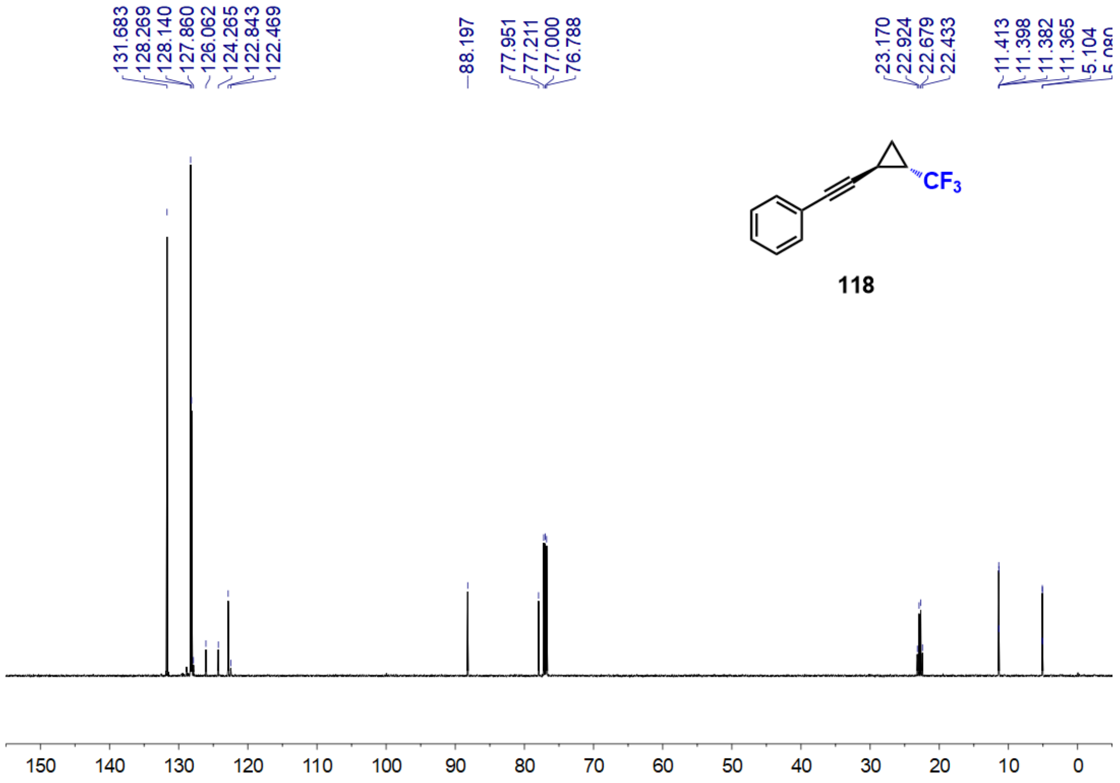

Supplementary Figure 349. ^{13}C NMR of 118

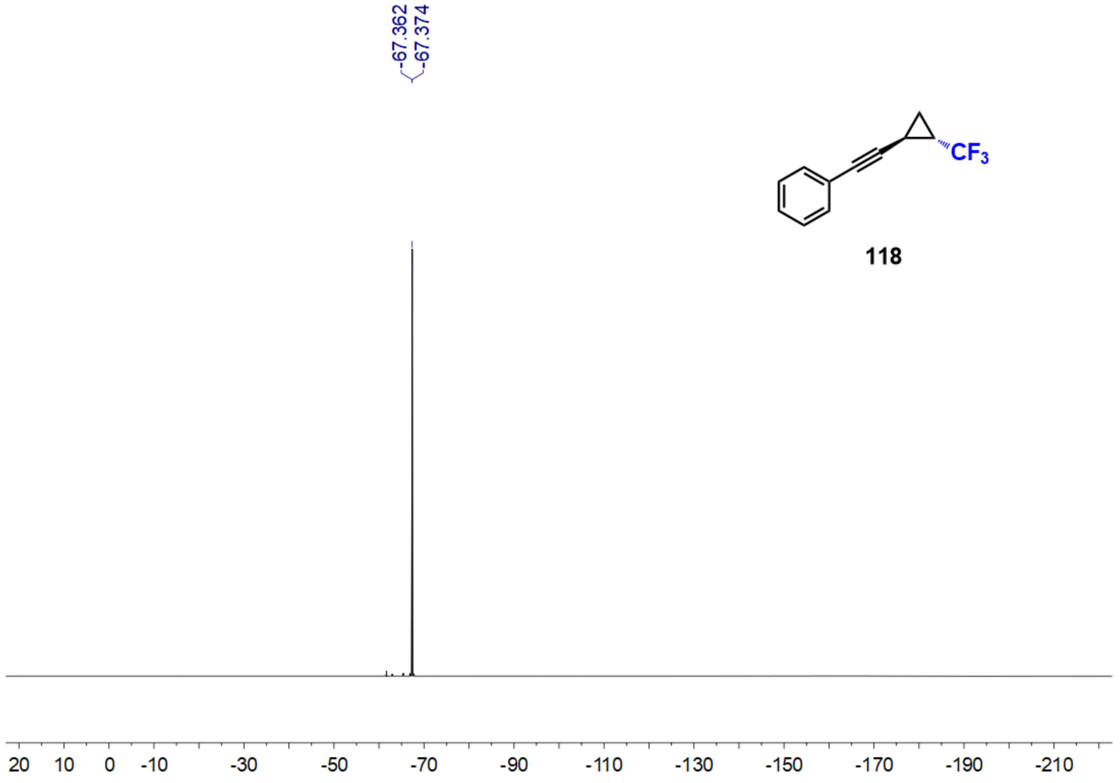

Supplementary Figure 350. ^{19}F NMR of 118

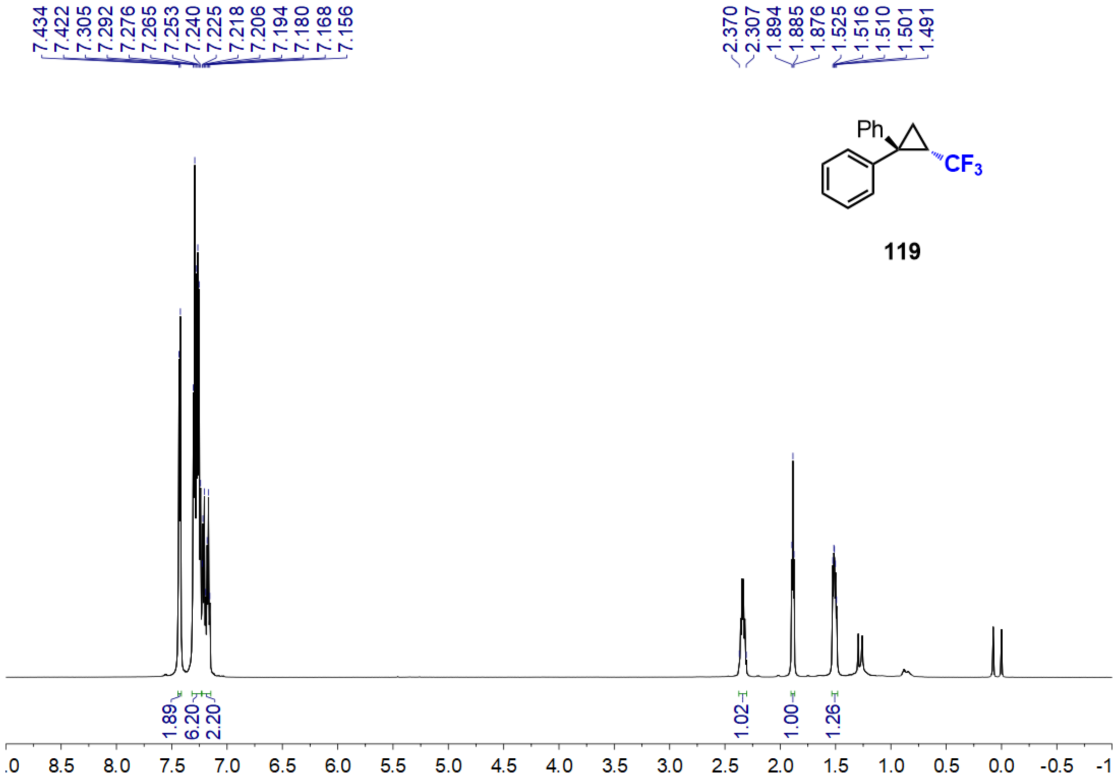

Supplementary Figure 351. ^{1}H NMR of 119

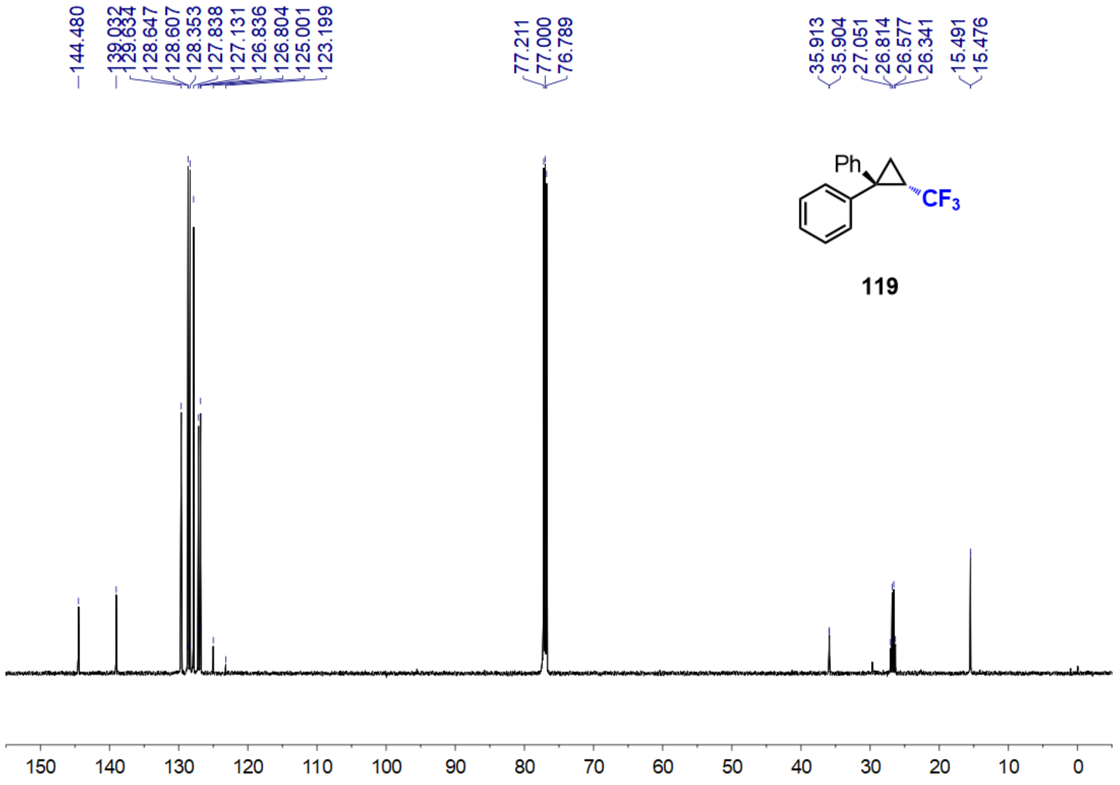

Supplementary Figure 352. ^{13}C NMR of 119

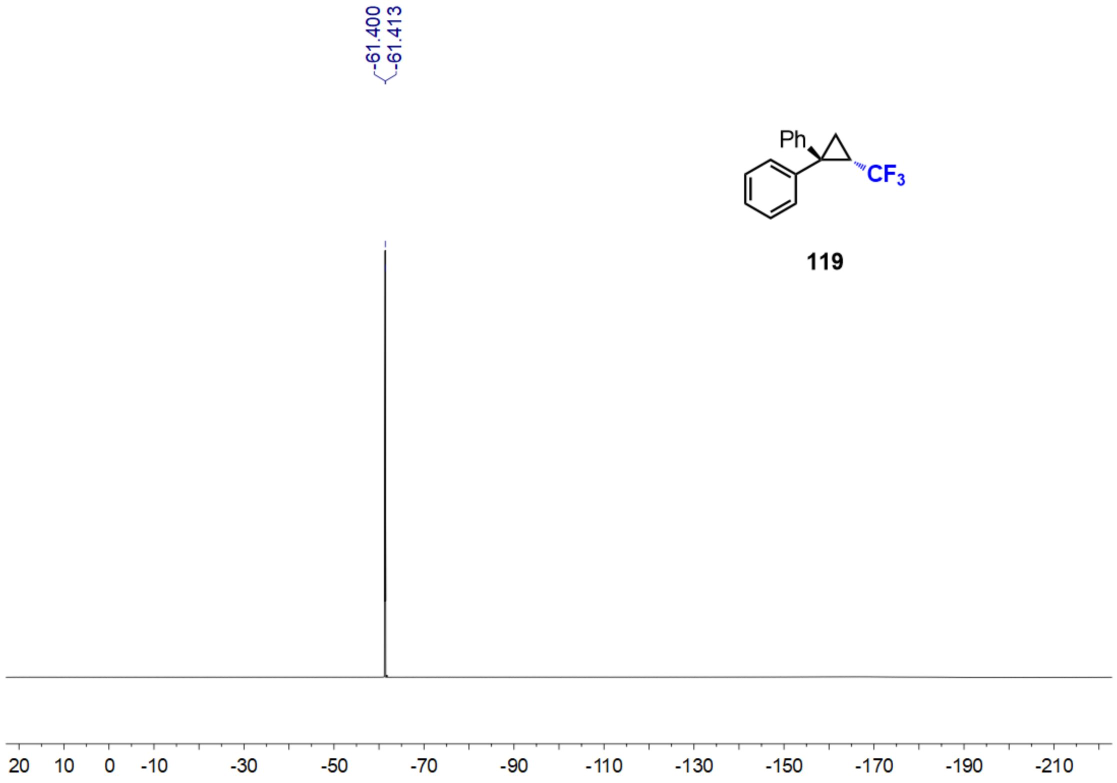

Supplementary Figure 353. ^{19}F NMR of 119

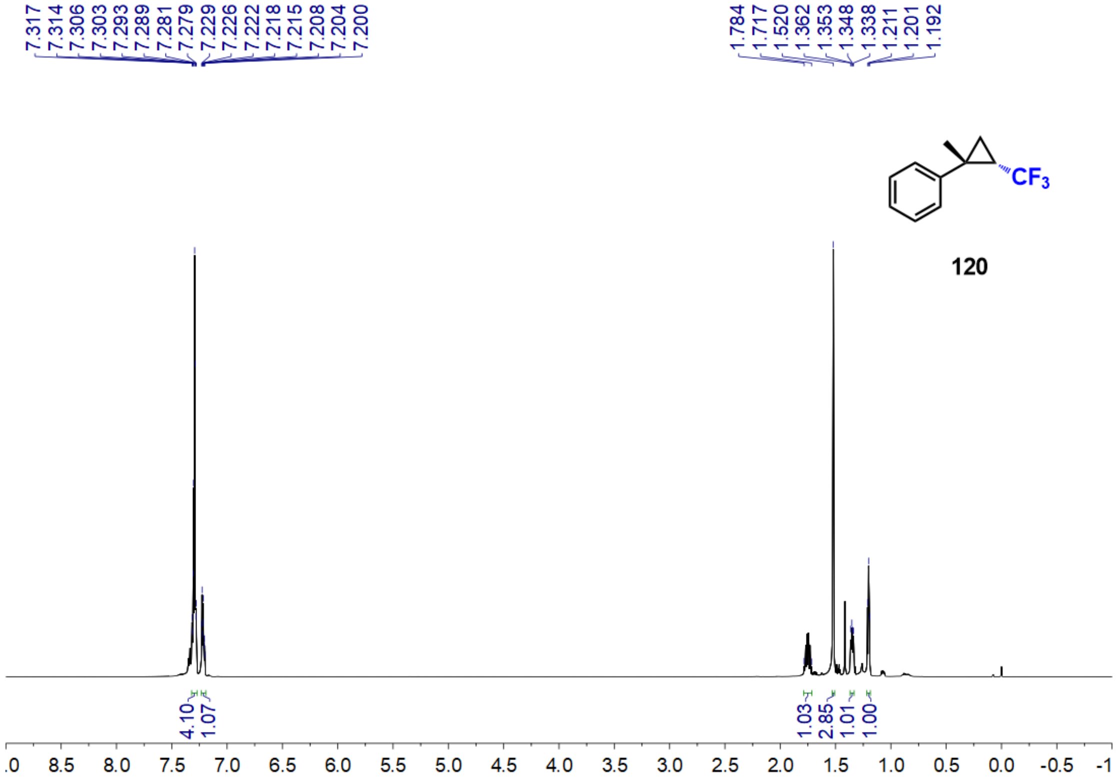

Supplementary Figure 354. ^{1}H NMR of 120

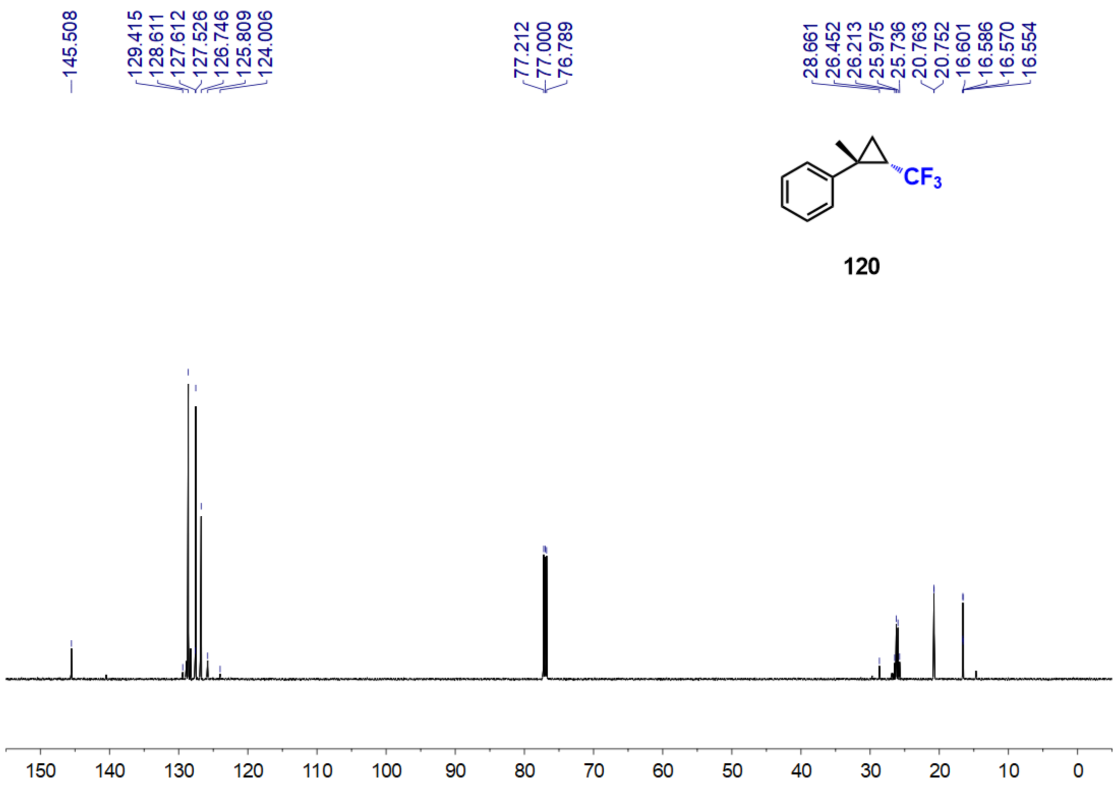

120

Supplementary Figure 355. ^{13}C NMR of 120

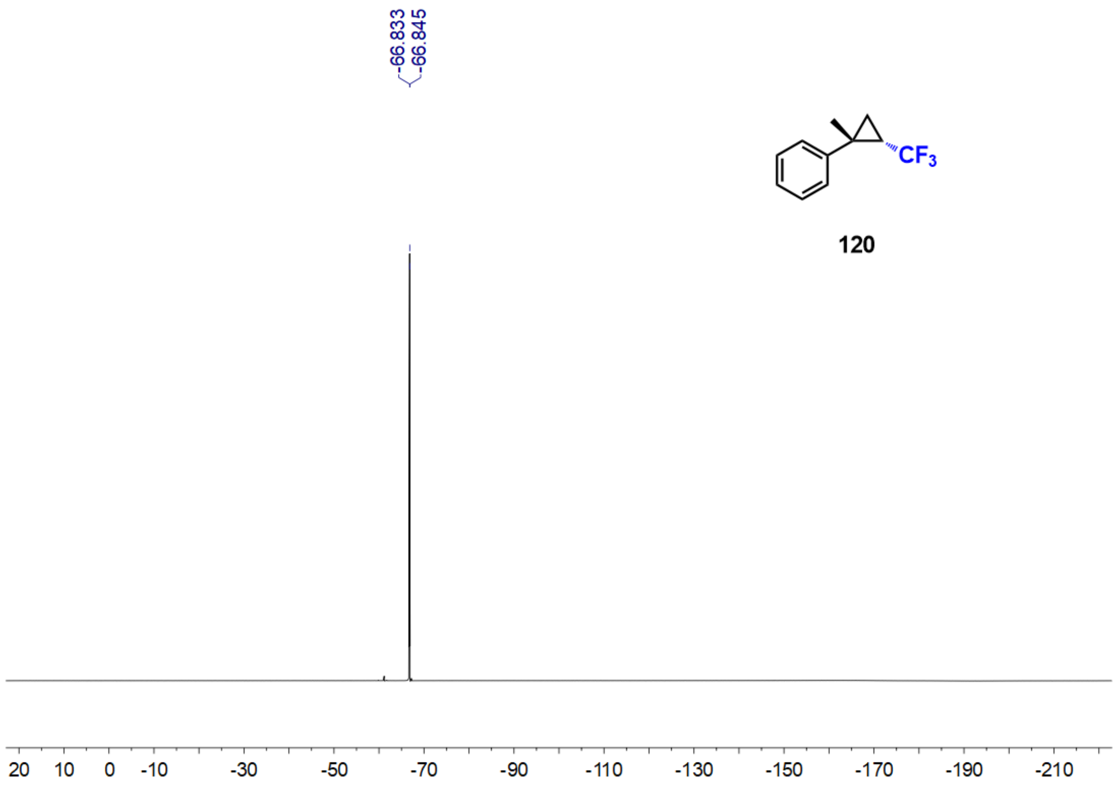

120

Supplementary Figure 356. ^{19}F NMR of 120

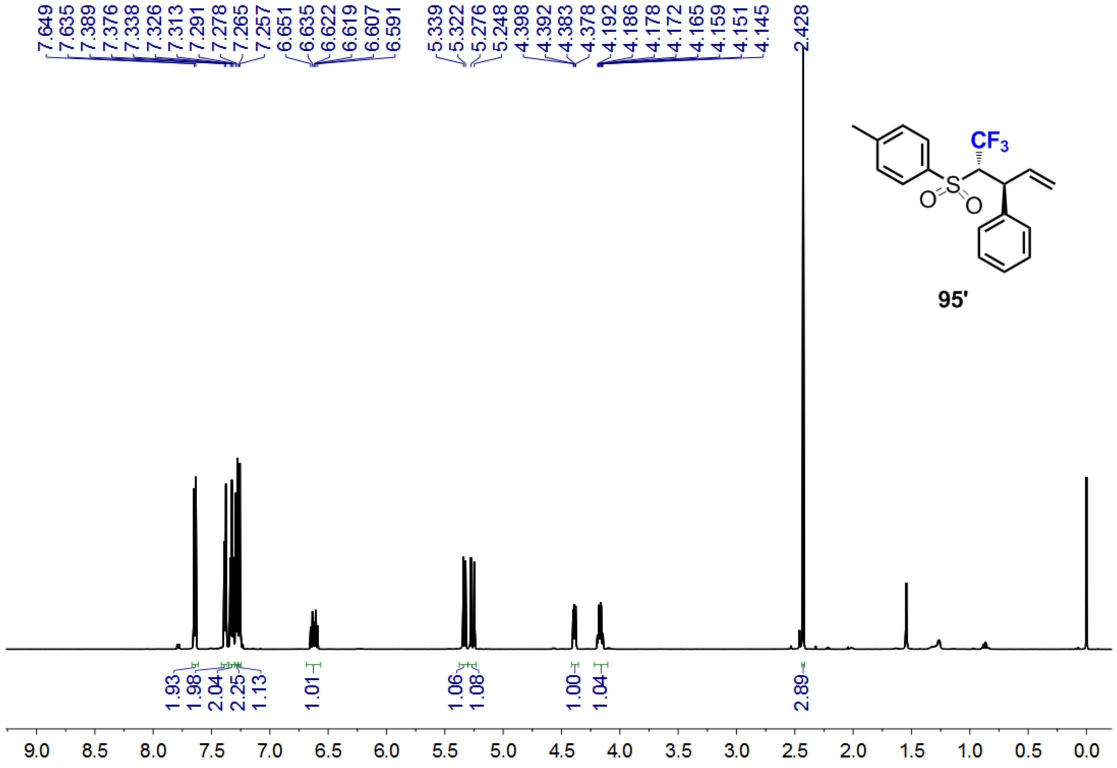

Supplementary Figure 357. ^{1}H NMR of 95’

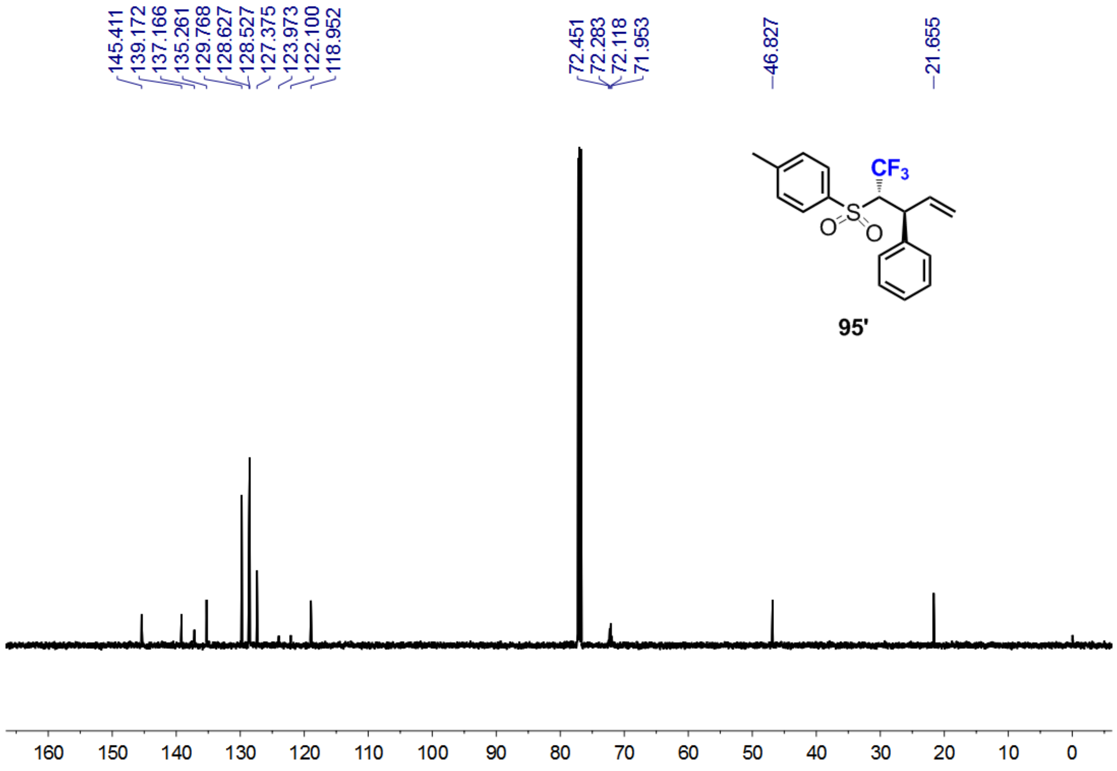

Supplementary Figure 358. ^{13}C NMR of 95’

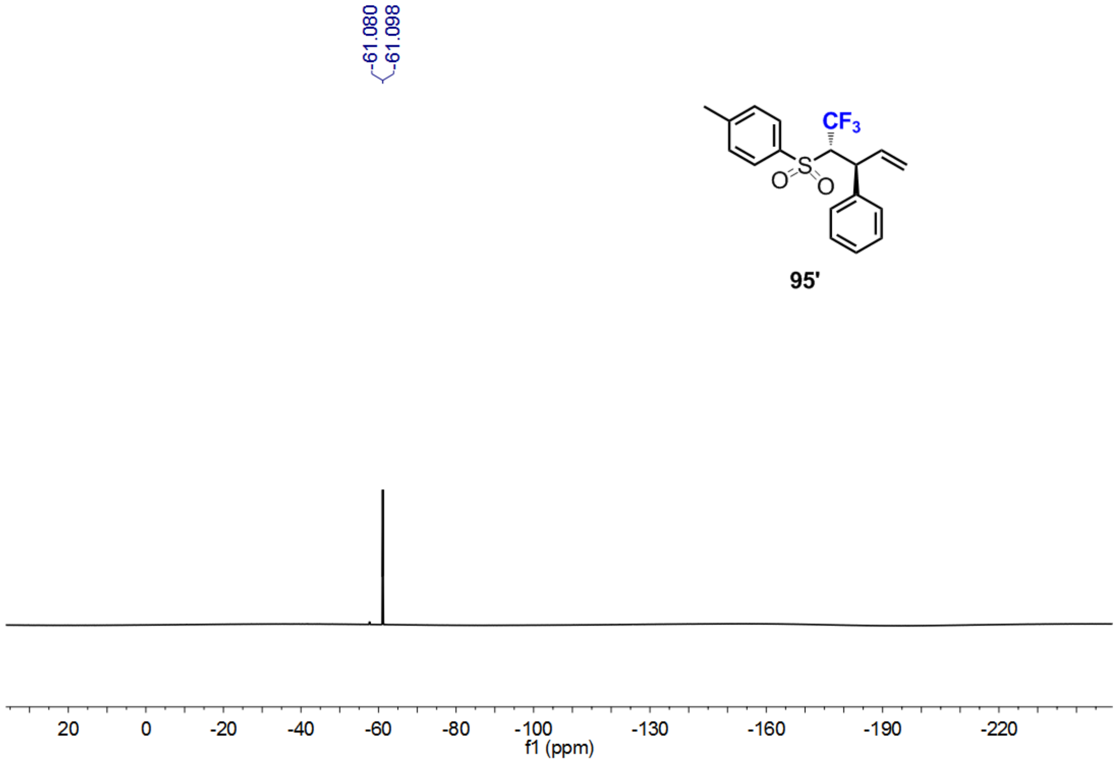

Supplementary Figure 359. ^{19}C NMR of 95’

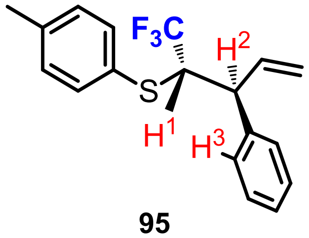

95

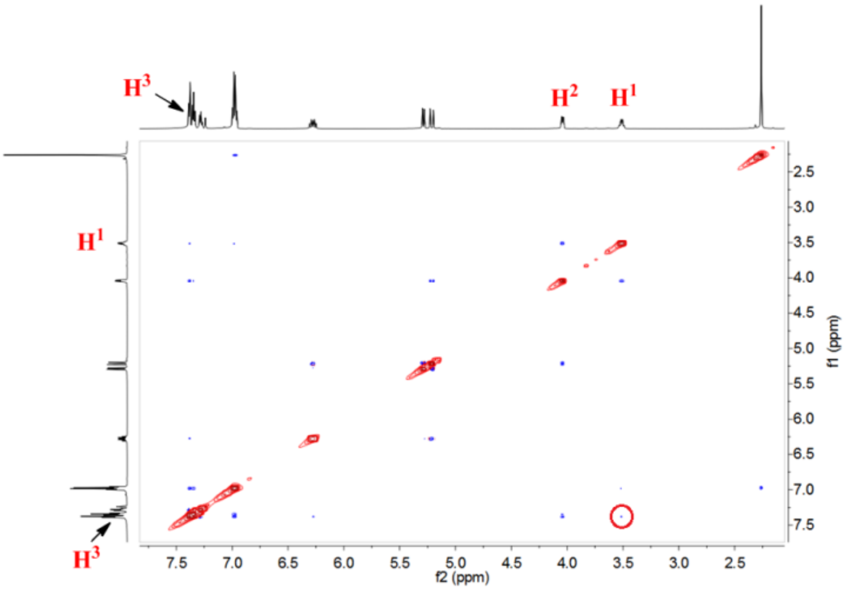

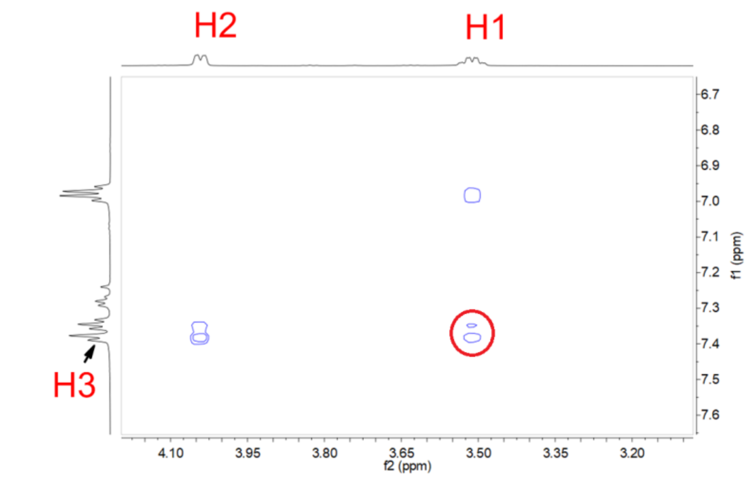

Supplementary Figure 360. NOESY Spectrum of compound 95. From the NOESY experiment of compound 95, we observed the correlation peaks between H^1 proton and H^3 proton in the NOESY spectrum of the compound 95. Thus, indicating the H^1 and H^3 are spatially close. This proves that CF_3 group and H^2 are on the same side, H^1 and phenyl ring are on the same side, and the product is a trans structure.

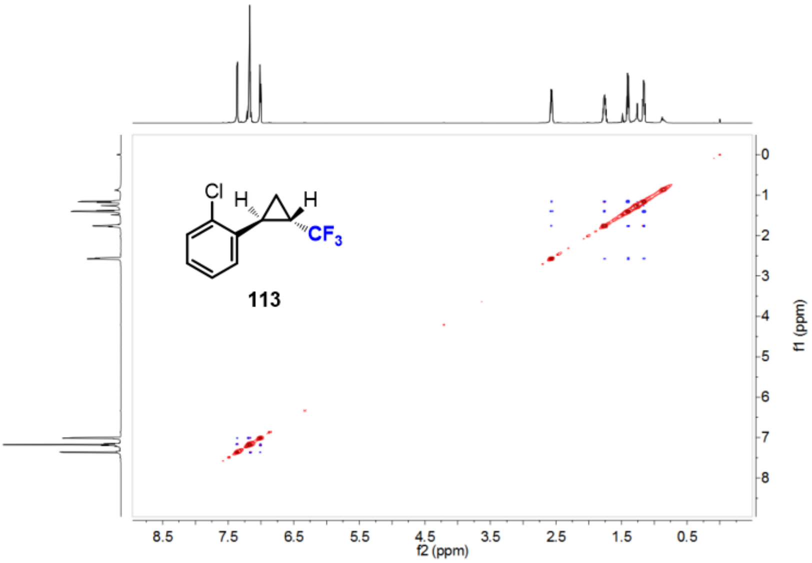

Supplementary Figure 361. NOESY Spectrum of compound 113

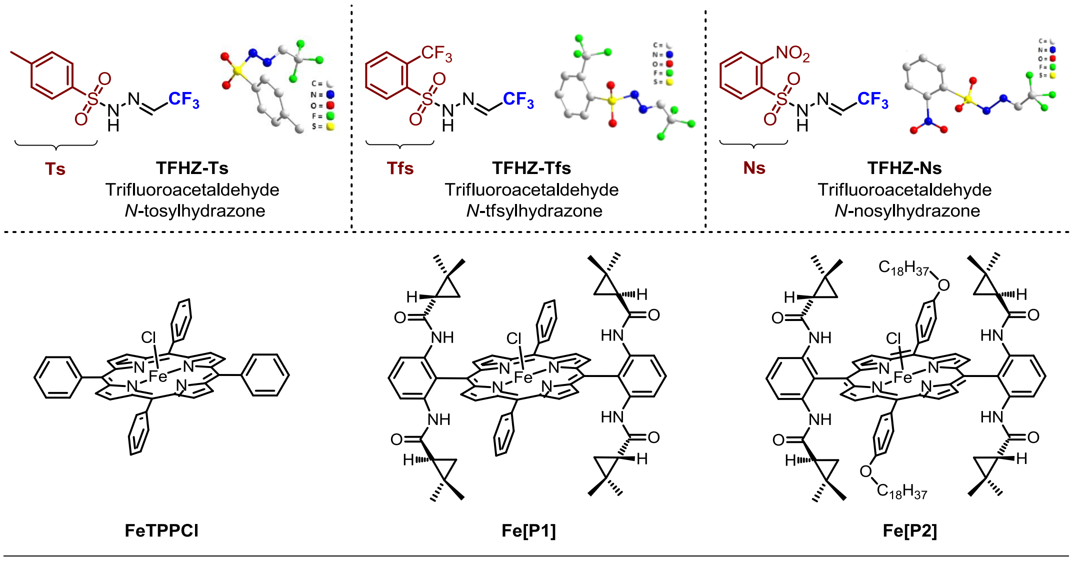

Supplementary Figure 362. trifluoroacetaldehyde sulfonylhydrazones structure and catalysts structure.

Supplementary Table 1. Optimization reaction conditions of *gem*-difluorovinyl sulfide.

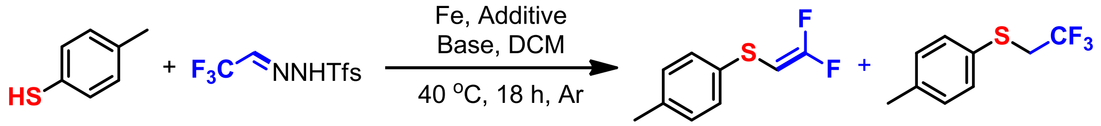

2 2'

Entry	Cat. (mol%)	Base(5 mL)[g]	Additive (30%)	**2** Yield[a]	**2'** Yield[a]
1[b]	FeTPPCl 3%	NaOH 20 wt%	--	6%	N.D.
2	FeTPPCl 3%	NaOH 20 wt%	--	13%	trace
3	FeTPPCl 5%	NaOH 20 wt%	--	39%	11%
4	FeTPPCl 5%	NaOH 20 wt%	$C_{12}H_{25}SO_3Na$	2%	trace
5	FeTPPCl 5%	NaOH 20 wt%	$C_{12}H_{25}PhSO_3Na$	53%	19%
6	FeTPPCl 5%	NaOH 20 wt%	$BnEt_3N^+Cl^-$	24%	16%
7	FeTPPCl 5%	KOH 20 wt%	$C_{12}H_{25}PhSO_3Na$	51%	9%
8	FeTPPCl 5%	NaH 5 eq.	$C_{12}H_{25}PhSO_3Na$	0%	56%
9[c]	Fe[P2] 5%	KOH 20 wt%	$C_{12}H_{25}PhSO_3Na$	75%	5%
10[c]	Fe[P2] 5%	KOH 20 wt%	--	40%	2%
11	Fe[P2] 1%	KOH 20 wt%	$C_{12}H_{25}PhSO_3Na$	82%	5%
12[c]	Fe[P2] 1%	KOH 20 wt%	$C_{12}H_{25}PhSO_3Na$	84%(80%)[f]	5%
13[c]	Fe[P1] 1%	KOH 20 wt%	$C_{12}H_{25}PhSO_3Na$	70%	5%
14[c,d]	Fe[P2] 1%	KOH 20 wt%	$C_{12}H_{25}PhSO_3Na$	32%	6%
15[c,e]	Fe[P2] 1%	KOH 20 wt%	$C_{12}H_{25}PhSO_3Na$	59%	11%

Reaction conditions: thiophenol (0.3 mmol), TFHZ-Tfs (0.6 mmol), DCM (1.0 mL) at 40 °C for 18 h. [a]The yields were determined by ^{1}H-NMR with CH_2Br_2 as an internal standard. [b]The reaction was performed for 5 h. [c]The reactions were carried out under air. [d]Trifluoroacetaldehyde N-nosylhydrazone (TFHZ-Ns) was used. [e]Trifluoroacetaldehyde N-tosylhydrazone (TFHZ-Ts) was used. [f]Yield in parentheses is isolated yeild. [g]NaOH aqueous and KOH aqueous were prepared from DI water.

Supplementary Table 2. Optimization reaction conditions of *gem*-difluorovinyl amine.

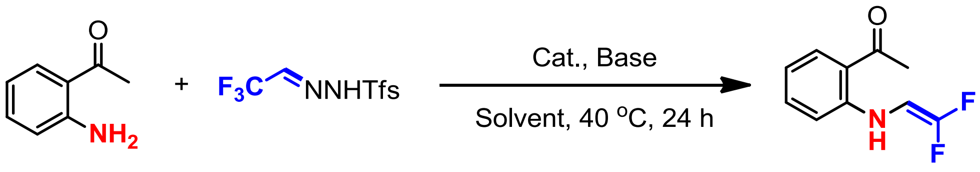

Entry	Cat.	Sol. 4 mL	Base (4 eq.)	**29** Yield[a]
1	$Cu(OTf)_2$ 30%	DCM	NaH	31%
2	$Cu[(CH_3CN)_4PF_6]$ 30%	DCM	NaH	42%
3	CuI 30%	DCM	NaH	17%
4	$Cu(OTf)_2$ 30%	DCM	LiO^tBu	47%
5	$Cu[(CH_3CN)_4PF_6]$ 30%	DCM	LiO^tBu	32%
6	$Cu(acac)_2$ 30%	DCM	LiO^tBu	45%
7	CuBr 30%	DCM	LiO^tBu	42%
8	FeTPPCl 5%	DCM	LiO^tBu	17%
9	$Cu(OTf)_2$ 15%	DCM	LiO^tBu	49%
9	$Cu(OTf)_2$ 20%	DCM	LiO^tBu	51%
10	$Cu(OTf)_2$ 20%	DCE	LiO^tBu	55%
11	$Cu(OTf)_2$ 20%	Chlorobenzene	LiO^tBu	54%
12	$Cu(OTf)_2$ 20%	DCE/toluene 3:1	LiO^tBu	60%(54%)[b]

Reaction conditions: amine (0.3 mmol), TFHZ-Tfs (0.6 mmol), base (1.2 mmol) and catalyst (0.06 mmol) in solvent were stirred at 40 °C for 24 h under Ar. [a]The yields were determined by ^{1}H-NMR with CH_2Br_2 as an internal standard. [b]Yield in parentheses is isolated yeild.

Supplementary Table 3. Optimization reaction conditions of *gem*-difluorovinyl ether.

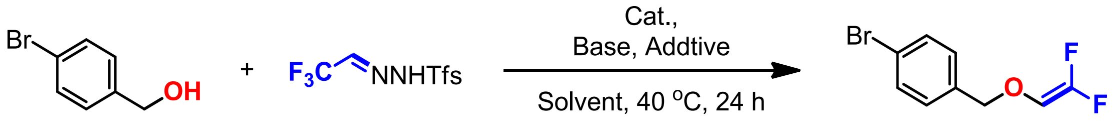

40

Entry	Cat. (30%)	Base (4.0 eq.)	Additive (1.0 eq.)	Sol. (8 mL)	40 Yield[a]
1	CuI	NaH	--	DCM	26 %
2	$Cu(OAc)_2$	NaH	--	DCM	28 %
3	Cu_2O	NaH	--	DCM	trace
4	CuF_2	NaH	--	DCM	trace
5	$CuBr \cdot SMe_2$	NaH	--	DCM	6 %
6	CuBr	NaH	--	DCM	38 %
7	CuBr	LiO^tBu	--	DCM	25 %
8	CuBr	$CsCO_3$	--	DCM	trace
9	CuBr	NaH	LiO^tBu	DCE	64 % (61%)[b]
10	CuBr	NaH	LiOTf	DCE	37 %
11	CuBr	NaH	LiOH	DCE	43 %
12	CuBr	NaH	CH_3OLi	DCE	35 %
13	CuBr	NaH	LiO^tBu	THF	N.D.
14	CuBr	NaH	LiO^tBu	CH_3CN	N.D.
15	CuBr	NaH	LiO^tBu	PhCl	42 %
16	CuBr	NaH	LiO^tBu	toluene	41 %

Reaction condition: TFHZ-Tfs (1 mmol), NaH (2 mmol), and solvent (8.0 mL) were stirred at rt for 1 h, then alcohol (0.5 mmol), additive (0.5 mmol) and catalyst (0.15 mmol) were added and the mixture was stirred at 40 ℃ for 24 h. [a]The yields were determined by ^{1}H-NMR with CH_2Br_2 as an internal standard. [b]Yield in parentheses is isolated yeild.

Supplementary Table 4. Crystal Structure of Trifluoroacetaldehyde N-tfsylhydrazone (TFHZ-Tfs) (CCDC No. 1814685)

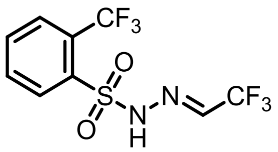

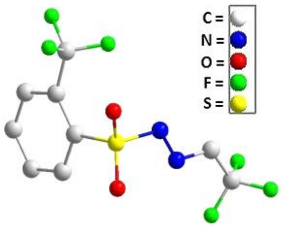

Empirical formula	$C_9H_6F_6N_2O_2S$
Temperature	293(2)
Wavelength	0.71073 Å
Unit cell dimensions	a = 11.325(2) Å<br>b = 5.1625(11) Å<br>c = 21.300(5) Å<br>alpha = 90.0 deg.<br>beta = 102.96(2) deg.<br>gamma = 90.0 deg.
Volume	1213.6(4) $Å^3$
Z	4
Calculated density	1.753 Mg/m^3
Absorption coefficient	0.347 mm^{-1}
F(000)	640
Crystal size	0.38 x 0.30 x 0.28 mm
Theta range for data collection	3.773 to 28.973 deg.
Reflections collected / unique	4789 / 2752 [R(int) = 0.0511]
Data / restraints / parameters	2752 / 0 / 181
Goodness-of-fit on F^2	1.077
Final R indices [I>2sigma(I)]	R1 = 0.0791, wR2 = 0.1953
Rindices (all data)	R1 = 0.1044, wR2 = 0.2324

Supplementary Table 5. Crystal Structure of Trifluoroacetaldehyde N-tosylhydrazone (TFHZ-Ts) (CCDCNo. 1814683)

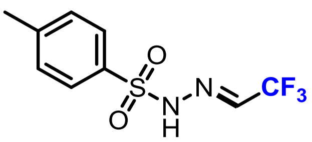

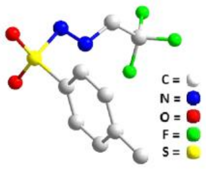

Empirical formula	$C_9H_9F_3N_2O_2S$
Temperature	298(2) K
Wavelength	0.71073 Å
Unit cell dimensions	a = 10.3210(7) Å<br>b = 13.0773(10) Å<br>c = 18.5089(19) Å<br>alpha = 83.295(7) deg.<br>beta = 81.976(7) deg.<br>gamma = 78.658(6) deg.
Volume	2415.1(4) Å ^{3}
Z	8
Calculated density	1.464 Mg/m^3
Absorption coefficient	0.298 mm^{-1}
F(000)	1088
Crystal size	0.37 x 0.30 x 0.28 mm
Theta range for data collection	2.952 to 29.348 deg.
Reflections collected / unique	16727 / 10915 [R(int) = 0.0293]
Data / restraints / parameters	10915 / 1 / 613
Goodness-of-fit on F^2	1.001
Final R indices [I>2sigma(I)]	R1 = 0.0743, wR2 = 0.2307
Rindices (all data)	R1 = 0.1572, wR2 = 0.3278

Supplementary Table 6. Crystal Structure of Trifluoroacetaldehyde N-nosylhydrazone (TFHZ-Ns) (CCDCNo. 1827227)

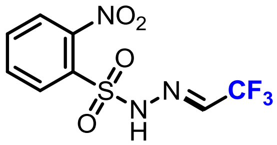

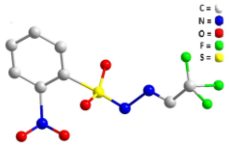

Empirical formula	$C_8H_6F_3N_3O_4S$
Temperature	298(2) K
Wavelength	0.71073 Å
Unit cell dimensions	a = 11.1525(7) Å<br>b = 20.6370(14) Å<br>c = 20.8588(14) Å<br>alpha = 90 deg.<br>beta = 101.305(6) deg.<br>gamma = 90 deg.
Volume	4707.6(5) Å ^{3}
Z	2
Calculated density	1.678 Mg/m^3
Absorption coefficient	0.330 mm^{-1}
F(000)	2402
Crystal size	0.38 x 0.30 x 0.28 mm
Theta range for data collection	3.345 to 29.247 deg.
Reflections collected / unique	21084 / 14829 [R(int) = 0.0858]
Data / restraints / parameters	14829 / 32 / 1370
Goodness-of-fit on F^2	0.949
Final R indices [I>2sigma(I)]	R1 = 0.0913, wR2 = 0.2162
Rindices (all data)	R1 = 0.1960, wR2 = 0.2957

NOTE: We appeared some Alert level A and Alert level B in the check cif file, we still did not solve the alert when we tried to give additional refinement cycles or use new space group. However, we have given sufficient evidence to prove the accuracy of the structure by ^{1}H, ^{13}C and ^{19}F Nuclear Magnetic Resonance (NMR) and High resolution mass spectra.

Supplementary Table 7. Crystal Structure of 9' (CCDC No. 1814506)

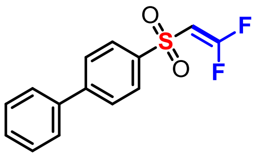

9'

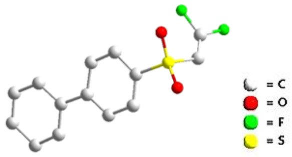

Empirical formula	$C_{14}H_{10}F_2O_2S$
Temperature	298(2) K
Wavelength	0.71073 Å
Unit cell dimensions	a = 10.1120(9) Å<br>b = 5.5299(3) Å<br>c = 23.032(2) Å<br>alpha = 90 deg.<br>beta = 90 deg<br>gamma = 90 deg.
Volume	1287.89(19) $Å^3$
Z	4
Calculated density	1.446 Mg/m^3
Absorption coefficient	0.269 mm^{-1}
F(000)	576
Crystal size	0.35 x 0.20 x 0.18 mm
Theta range for data collection	3.538 to 29.151 deg.
Reflections collected / unique	5008 / 2778 [R(int) = 0.0355]
Data / restraints / parameters	2778 / 1 / 176
Goodness-of-fit on F^2	1.014
Final R indices [I>2sigma(I)]	R1 = 0.0551, wR2 = 0.0942
Rindices (all data)	R1 = 0.1120, wR2 = 0.1171

Supplementary Table 8. Crystal Structure of 95' (oxidation product of 95, CCDC No. 1881268)

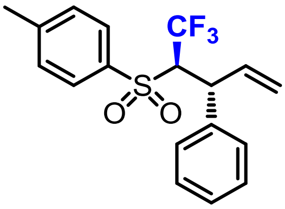

95'

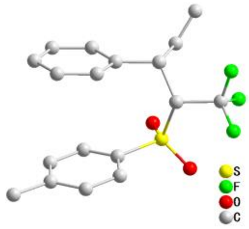

Empirical formula	$C_{18}H_{17}F_3O_2S$
Temperature	293(2) K
Wavelength	0.71073 Å
Unit cell dimensions	a = 10.049(3) Å<br>b = 15.58(3) Å<br>c = 16.736(6) Å<br>alpha = 90 deg.<br>beta = 105.91(3) deg<br>gamma = 90 deg.
Volume	2520(5) $Å^3$
Z	6
Calculated density	1.401 Mg/m^3
Absorption coefficient	0.231 mm^{-1}
F(000)	1104
Crystal size	0.35 x 0.20 x 0.18 mm
Theta range for data collection	3.713 to 29.381 deg.
Reflections collected / unique	11478 / 8866 [R(int) = 0.0417]
Data / restraints / parameters	8866 / 1 / 658
Goodness-of-fit on F^2	0.950
Final R indices [I>2sigma(I)]	R1 = 0.0762, wR2 = 0.2477
Rindices (all data)	R1 = 0.1438, wR2 = 0.3385

Supplementary Methods

General information

All reagents were purchased from commercial sources and used without purification unless otherwise mentioned. The products were purified by column chromatography over silica gel (200-400 size). ^{1}H, ^{13}C and ^{19}F Nuclear Magnetic Resonance (NMR) spectra were recorded at 25 ℃ on a Bruker 600 MHz, 150 MHz and 564 MHz, and TMS was used as internal standard. Mass spectra were recorded on TSQ 8000 Evo by using EI method. High resolution mass spectra (HRMS) were recorded on Bruck microTof by using ESI method and Waters Micromass GCT Premier by using EI method (Shanghai Institute of Organic Chemistry).

Synthesis and characterization data for the hydrazones

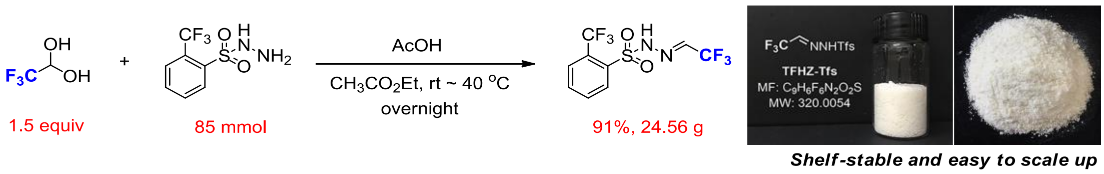

A 500 mL bottom flask was charged with trifluoroacetaldehyde monohydrate (14.9 g, 127.5 mmol), *o*-trifluoromethylbenzenesulfonyl hydrazide (20.4 g, 85.0 mmol) and EA (310.0 mL). Then CH_3COOH (4.3 mL, 7.7 mmol) was added dropwise and stirred at ice water bath for 10 minutes under nitrogen. Then the mixture was transferred to room temperature and stirred for 80 minutes. After that, the mixture was transferred to 40 ℃ and stirred overnight, and monitored by TLC (PE:EA=2:1). After the reaction was complete, the mixture was concentrated under reduced pressure and washed by PE/EE to afford the product as a white solid (24.56 g, 91% yield).

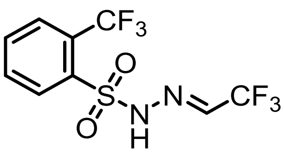

Trifluoroacetaldehyde *N*-tfsylhydrazone (TFHZ-Tfs): White solid, m.p: 125-126 °C; **^{1}H-NMR** (600 MHz, DMSO) δ 13.05 (s, 1H), 8.12 (d, *J* = 7.8 Hz, 1H), 7.99 (d, *J* = 7.8 Hz, 1H), 7.93 (t, *J* = 7.8 Hz, 1H), 7.88 (t, *J* = 7.8 Hz, 1H), 7.58 (q, *J* = 7.8 Hz, *J* = 4.2 Hz, 1H). **^{13}C-NMR** (150 MHz, DMSO) δ 137.46, 134.64, 133.54 (q, *J* = 37.6 Hz), 133.46 (q, *J* = 37.9 Hz), 132.06, 129.14 (q, *J* = 6.3 Hz), 126.98 (q, *J* = 33.0 Hz), 123.13 (q, *J* = 272.4 Hz), 120.39 (q, *J* = 271.5 Hz). ^{19}F-NMR

(565 MHz, DMSO) δ -51.95, -61.95 (d, J = 3.8 Hz). **HRMS** (ESI) m/z calculated for $C_9H_6F_6N_2NaO_2S$ [M+Na]$^+$ 342.9952, found 342.9940.

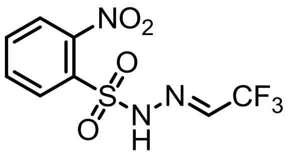

Trifluoroacetaldehyde *N*-nosylhydrazone (TFHZ-Ns): White solid, m.p: 124-125 °C; **^{1}H-NMR** (600 MHz, DMSO) δ 13.00 (s, 1H), 8.06 (dd, J = 7.8 Hz, J = 1.2 Hz, 1H), 8.03 (dd, J = 7.8 Hz, J = 1.8 Hz, 1H), 7.95 (td, J = 7.8 Hz, J = 1.2 Hz, 1H), 7.91 (dd, J = 7.2 Hz, J = 1.2 Hz, 1H), 7.65 (q, J = 4.2 Hz, 1H). **^{13}C-NMR** (150 MHz, DMSO) δ 148.24, 136.10, 134.45 (q, J = 37.8 Hz), 133.65, 131.30, 130.86, 125.65, 120.42 (q, J = 272.0 Hz). **^{19}F-NMR** (565 MHz, DMSO) δ -66.52. **HRMS** (ESI) m/z calculated for $C_8H_8F_3N_3NaO_4S$ [M+Na]$^+$ 319.9925, found 319.9920.

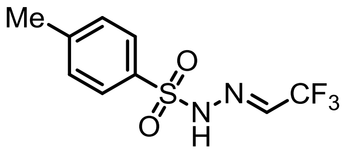

Trifluoroacetaldehyde *N*-tosylhydrazone (TFHZ-Ts): White solid, m.p: 116-117 °C; **^{1}H-NMR** (600 MHz, DMSO) δ 12.48 (s, 1H), 7.72 (d, J = 8.4 Hz, 2H), 7.50 (q, J = 4.0 Hz, 1H), 7.42 (d, J = 8.4 Hz, 2H), 2.32 (s, 3H). **^{13}C-NMR** (150 MHz, DMSO) δ 144.75, 135.86, 133.46 (q, J = 37.6 Hz), 130.42, 127.67, 120.37 (q, J = 272.0 Hz), 21.47. **^{19}F-NMR** (565 MHz, DMSO) δ -66.50 (d, J = 3.4 Hz). HRMS (ESI) m/z calculated for $C_8H_8F_3N_3NaO_4S$ [M+Na]$^+$ 289.0235, found 289.0231.

General procedure for the gram-scale synthesis of 9

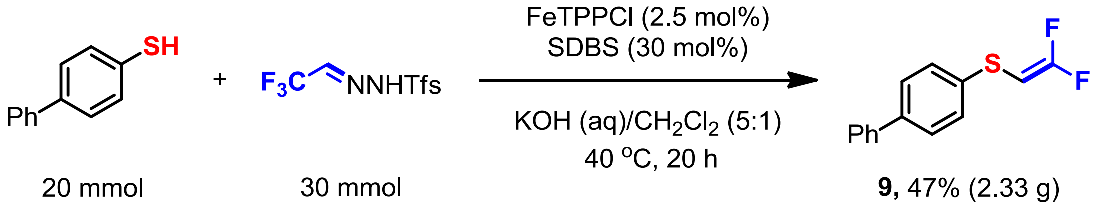

A 500 mL round-bottomed flask was charged with TFHZ-Tfs (9.6 g, 30.0 mmol), *p*-phenylthiophenol (3.7 g, 20.0 mmol), FeTPPCl (352.0 mg, 0.5 mmol) and SDBS (sodium dodecylbenzenesulphonate) (2.09 g, 6.0 mmol) under air, followed by addition of DCM (66.7 mL), KOH aq (333.0 mL, 20 wt%) and install the reflux condenser. The resulting mixture was stirred at 40 ℃ for 20 h. Then water was added to the mixture, which was extracted with DCM. The organic layer was combined and dried with anhydrous $MgSO_4$, then filtered through a silica gel

eluting with DCM. The filtrate was evaporated under reduced pressure to leave a crude mixture, which was separated by flash column chromatography to afford the pure product **9** in 47% as a white solid.

General procedure for the synthesis of *gem*-difluorovinyl Sulfone 9'

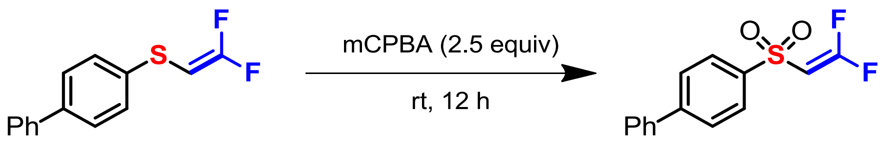

9 **9',** 77%

A screw capped reaction vial was charged with 4-((2,2-difluorovinyl)sulfonyl)-1,1'-biphenyl **9** (124.1 mg, 0.5 mmol), *m*CPBA (253.8 mg, 85 wt%, 1.25 mmol) under air, followed by addition of DCM (5.0 mL) and the mixture was stirred for 12 h at room temperature. The resulting solution was successively washed with aq. $Na_2S_2O_3$ and aq. $NaHCO_3$ solutions. Organic layer was dried over Na_2SO_4, and the solvent was removed under reduced pressure. The residue was chromatographed on a SiO_2 column (1:1 hexane–EtOAc) to give sulfone **9'** in 77% as a white solid.

General procedure for the synthesis of 95'

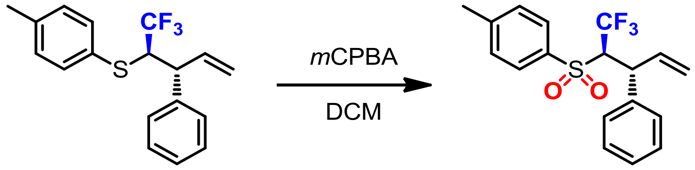

95 **95'**

The synthesis method is the same as 9'.

General procedure for the synthesis of 66

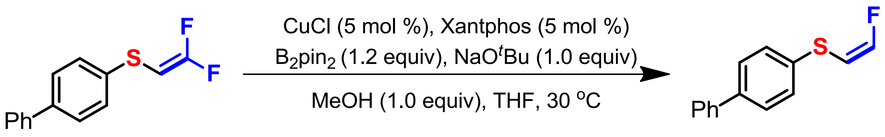

9 **66,** 67%

A screw capped reaction vial was charged with copper chloride (2.70 mg, 0.027 mmol), Xantphos (14.47 mg, 0.025 mmol), bis(pinacolato)diboron (154.9 mg, 0.61 mmol), **9** (124.1 mg, 0.5 mmol) and NaO^{t}Bu (48.0 mg, 0.5 mmol), then evacuated and filled with argon for three times, followed by addition of THF (1.0 mL) and MeOH (22.0 μL) via syringe. Then the mixture was stirred at 30 ℃ overnight. After the reaction was complete, the mixture was passed through a

short silica gel eluting with Et_2O. The crude material was evaporated under reduced pressure to leave a crude mixture, which was purified by column chromatography on silica gel (eluting with petroleum ether/ethyl acetate) to afford **66** in 67% as a colorless oil.

General procedure for the synthesis of 67

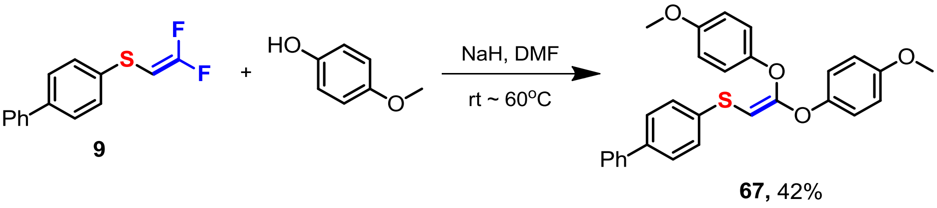

A solution of *p*-methoxyphenol (248.3 mg, 2.0 mmol) dissolved in DMF (5 mL) was added dropwise to a stirred suspension of NaH (320.0 mg, 60 wt%, 8.0 mmol) in DMF (3 mL) at room temperature under Ar. The flask was placed in a preheated oil bath at 60 °C. Then **9** (124.1 mg, 0.5 mmol) was transferred into the solution in a single portion under Ar. After 1 h, the mixture was treated with DI H_2O (25 mL). The organic layer was separated and the aqueous layer was extracted with Et_2O for three times. The combined organic layers were washed with water, dried over $MgSO_4$, filtered over a pad of silica gel. The crude material was evaporated under reduced pressure to leave a crude mixture, which was purified by column chromatography on silica gel (eluting with petroleum ether/ethyl acetate) to afford **67** in 42% as a colorless oil.

General procedure for synthesis of Fe[P2] and Fe[P1]:

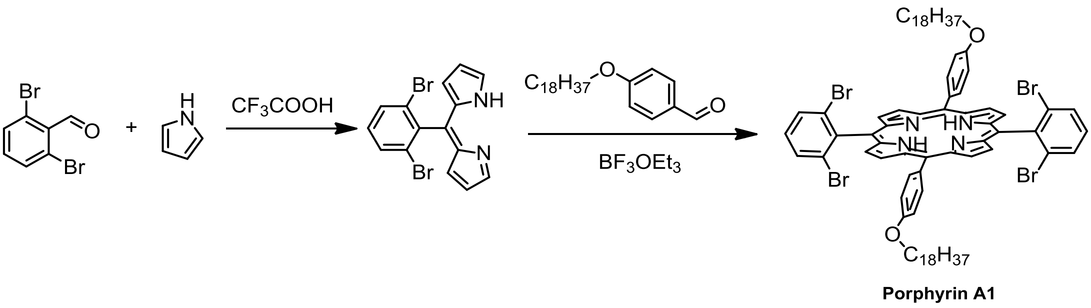

The meso-(2,6-dibromophenyl) dipyrromethane was prepared according to the lterature[1].

The 4-(octadecyloxy)benzaldehyde was prepared according to the lterature[2].

The **Porphyrin A1** was prepared according to the literature[1]. A mixture of meso-(2,6-dibromophenyl)dipyrromethane (1.0 mmol), aldehyde (1.0 mmol), and molecular sieves (4Å, 0.300 g) in chloroform (150 mL) was purged with nitrogen for 10 min. Boron trifluoride diethyl etherate (0.1 mL) was added dropwise via a syringe and the flask was wrapped with aluminum foil

to shield it from light. The solution was stirred under a nitrogen atmosphere at room temperature for 3 h, and 2,3-dichloro-5,6-dicyano-1,4-benzoquinone (DDQ) (1.2 mmol) was added as powder at one time. After 30 min, 1 mL of triethylamine was added. The reaction solution was then directly poured on the top of a silica gel column that was packed with dichloromethane. The column was eluted with dichloromethane to afford the pure compound as a purple solid (0.365g, 50%). **^{1}H NMR** (600 MHz, $CDCl_3$): δ 8.89 (d, *J* = 4.7 Hz, 4H), 8.63 (d, *J* = 4.7 Hz, 4H), 8.12 (d, *J* = 8.3 Hz, 4H), 8.01 (d, *J* = 8.3 Hz, 4H), 7.48 (t, *J* = 8.2 Hz, 2H), 7.25 (t, *J* = 8.5 Hz, 4H), 4.22 (t, *J* = 6.5 Hz, 4H), 1.99-1.94 (m, 4H), 1.64-1.59 (m, 4H), 1.51-1.44 (m, 6H), 1.38-1.24 (m, 50H), 0.88 (t, *J* = 7.0 Hz, 6H), -2.54 (s, 2H).

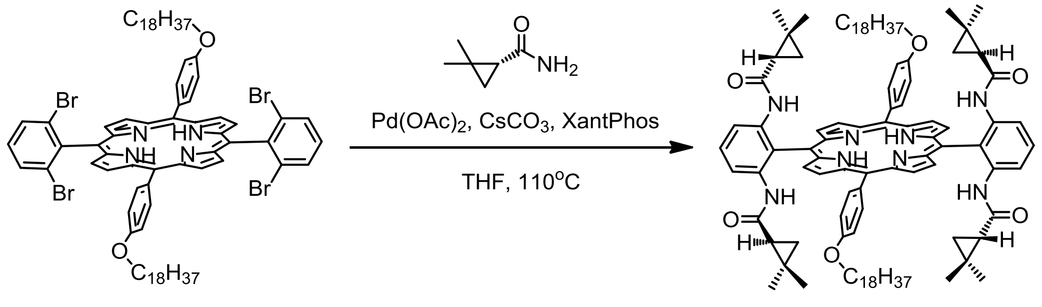

The **Porphyrin B1** was prepared according to the literature[1]. An oven-dried Schlenk tube equipped with a stirring bar was degassed on a vacuum line and purged with nitrogen. The tube was then charged with above obtained **Porphyrin A1** (0.1 mmol, 1 eq.), amide (1.6 mmol, 16 eq.), $Pd(OAc)_2$ (0.04 mmol, 40%), Xantphos (0.08 mmol, 80%), Cs_2CO_3 (1.6 mmol, 16 eq.). The tube was capped with a Teflon screw cap, evacuated, and backfilled with nitrogen. After the Teflon screw cap was replaced with a rubber septum, THF (10 mL) was added via syringe. The tube was purged with nitrogen (1-2 mins) and the septum was then replaced with the Teflon screw cap and sealed. The reaction mixture was heated in an oil bath at 100℃ with stirring for 72 hours. After removed solvent, the resulting reaction mixture was purified by chromatography on silica gel and the pure compound was a purple solid (0.112 g, 70%). **^{1}H NMR** (600 MHz, $CDCl_3$): δ 9.05 (d, *J* = 4.5 Hz, 4H), 8.92 (s, 4H), 8.50 (s, 4H), 8.13 (d, *J* = 7.8 Hz, 4H), 7.86 (t, *J* = 8.8 Hz, 2H), 7.37 (d, *J* = 8.5 Hz, 4H), 6.53 (s, 4H), 4.32 (t, *J* = 6.4 Hz, 4H), 2.07-2.01 (m, 4H), 1.71-1.65 (m, 4H), 1.61-1.50 (m, 5H), 1.48-1.26 (m, 58H), 0.93-0.90 (m, 19H), 0.73, (s, 3H), 0.22-(-0.04) (m, 15H), -2.54 (s, 2H).

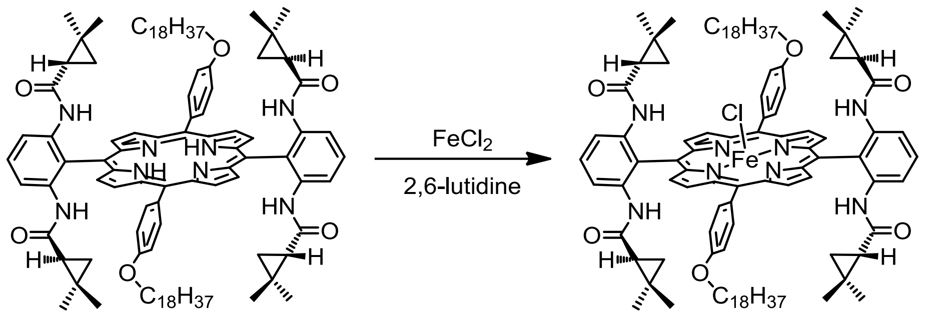

Porphyrin B **Fe[P2]**

The **Porphyrin B1** (0.080 g) and anhydrous $FeCl_2$ (0.030 g) were placed in an oven-dried, resealable Schlenk tube. The tube was capped with a Teflon screwcap, evacuated, and backfilled with nitrogen. The screwcap was replaced with a rubber septum, and 2,6-lutidine (0.030 mL) and dry DMF (6.0 mL) were added via syringe. Then the septum was replaced with the Teflon screwcap. The tube was sealed, and its contents were heated at 160 ℃ with stirring. The resulting mixture was cooled to room temperature, taken up in CH_2Cl_2, and transferred to a separatory funnel. The mixture was washed with 0.1 M HCl, then washed with water 3 times and concentrated in vacuo. The pure compound was obtained as a black solid (67.0 mg, 80%). **HRMS** (ESI) m/z calculated for $C_{104}H_{136}ClFeN_8O_6$ $[M]^+$ 1683.9621, found 1683.9637.

General procedure for the synthesis of **Fe[P1]** is same to **Fe[P2]**.

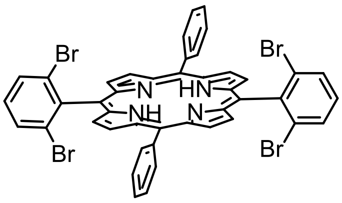

Porphyrin A2

Porphyrin A2: Purple solid. Yield: 47%. ^{1}**H NMR** (600 MHz, $CDCl_3$): δ 8.87 (d, *J* = 4.8 Hz, 4H), 8.66 (d, *J* = 4.8 Hz, 4H), 8.25 (m, 4H), 8.04 (d, *J* = 8.2 Hz, 4H), 7.76 (m, 6H), 7.55 (t, *J* = 8.2 Hz, 2H), −2.58 (s, 2H).

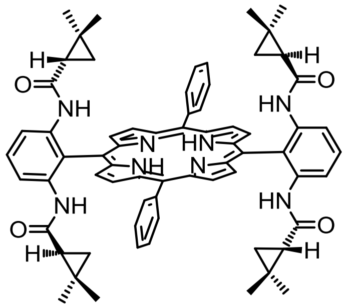

Porphyrin B2

Porphyrin B2: Purple solid. Yield: 78%. **^{1}H NMR** (600 MHz, $CDCl_3$): δ 9.00 (d, J = 4.8 Hz, 4H), 8.92 (d, *J* = 4.8 Hz, 4H), 8.49 (s, 4H), 8.23 (d, *J* = 6.0 Hz, 4H), 7.88 (m, 8H), 6.48 (s, 4H), 0.92 (s, 12H), 0.74 (s, 4H), (−0.03)-(-0.13) (m, 20H), −2.60 (s, 2H).

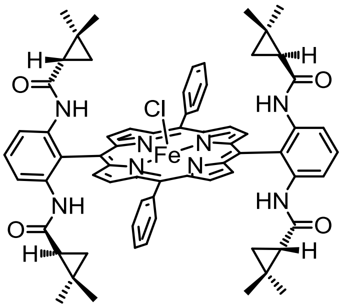

Fe[P1]

Fe[P1]: Purple solid. Yield: 77%. **HRMS** (ESI) m/z calculated for $C_{68}H_{64}ClFeN_8O_4$ $[M]^+$ 1147.4088, found 1147.4098.

Characterization data for the products

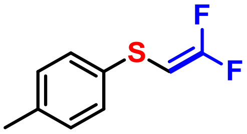

(2,2-difluorovinyl)(p-tolyl)sulfane (2) Colorless oil; **^{1}H-NMR** (600 MHz, $CDCl_3$) δ 7.21 (d, *J* = 7.8 Hz, 2H), 7.11 (d, *J* = 7.8 Hz, 2H), 5.11 (dd, *J* = 21.6 Hz, *J* = 1.0 Hz, 1H), 2.32 (s, 3H). **^{13}C-NMR** (150 MHz, $CDCl_3$) δ 158.7 (dd, *J* = 295.2 Hz, *J* = 290.0 Hz), 136.7, 131.4 (t, *J* = 2.0 Hz), 129.9, 128.4, 74.2 (dd, *J* = 21.0 Hz, *J* = 31.2 Hz), 20.9. **^{19}F NMR** (564 MHz, $CDCl_3$) δ -77.04 (d, *J* = 21.6 Hz), -79.75 (t, *J* = 21.6 Hz). **IR** (KBr): 2954, 1716, 1700, 1653, 1153, 837 cm^{-1}. **MS** (EI, m/z): 186 (M^+, 100.00), 171 (18.80). **HRMS** (EI): exact mass calcd for $C_9H_8F_2S$ (M^+): 186.0315, found: 186.0319.

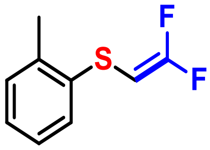

(2,2-difluorovinyl)(o-tolyl)sulfane (3) Colorless oil; **^{1}H-NMR** (600 MHz, $CDCl_3$) δ 7.52 (d, *J* = 7.6 Hz, 1H), 7.17-7.15 (m, 2H), 7.13-7.10 (m, 1H), 5.07 (dd, *J* = 21.0 Hz, *J* = 1.0 Hz, 1H), 2.37 (s, 3H). **^{13}C-NMR** (150 MHz, $CDCl_3$) δ 158.9 (dd, *J* = 297.6 Hz, *J* = 292.6 Hz),136.6, 134.3 (t, *J* = 2.2 Hz), 130.3, 127.3, 126.6, 126.4, 73.0 (dd, *J* = 31.6 Hz, *J* = 21.0 Hz), 19.9. **^{19}F NMR** (564

MHz, $CDCl_3$) δ -75.90 (d, J = 20.3 Hz), -79.45 (t, J = 20.3 Hz). **IR** (KBr): 3065, 2923, 1706, 1650, 1314, 1714, 912, 744 cm^{-1}. **MS** (EI, m/z): 186 (M^+, 90.90), 171 (13.17), 141 (100.00). **HRMS** (EI): exact mass calcd for $C_9H_8F_2S$ (M^+): 186.0315, found: 186.0319.

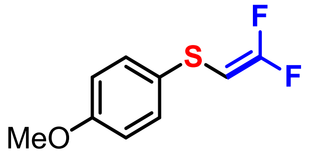

(2,2-difluorovinyl)(4-methoxyphenyl)sulfane (4) Colorless oil; **^{1}H-NMR** (600 MHz, $CDCl_3$) δ 7.31-7.29 (m, 2H), 6.86-6.84 (m, 2H), 5.09 (dd, J = 21.4 Hz, J = 1.0 Hz, 1H), 3.79 (s, 3H). **^{13}C-NMR** (150 MHz, $CDCl_3$) δ 159.2, 158.2 (dd, J = 297.2 Hz, J = 290.8 Hz), 131.4, 125.3 (t, J = 2.0 Hz), 114.8, 75.4 (dd, J = 30.6 Hz, J = 21.0 Hz), 55.4. **^{19}F NMR** (564 MHz, $CDCl_3$) δ -78.18 (d, J = 23.0 Hz), -80.46 (dd, J = 23.0 Hz, J = 21.4 Hz). **IR** (KBr): 3057, 1705, 1650, 1460, 1305, 1169, 1082, 772 cm^{-1}. **MS** (EI, m/z): 202 (M^+, 100.00), 187 (29.74). **HRMS** (EI): exact mass calcd for $C_9H_8F_2OS$ (M^+): 202.0264, found: 202.0269.

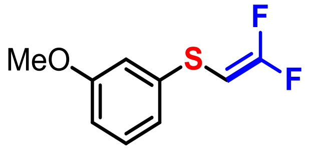

(2,2-difluorovinyl)(3-methoxyphenyl)sulfane (5) Colorless oil; **^{1}H-NMR** (600 MHz, $CDCl_3$) δ 7.21 (t, J = 8.2 Hz, 1H), 6.87 (d, J = 7.8 Hz, 1H), 6.83 (t, J = 2.4 Hz, 1H), 6.74 (dd, J = 8.2 Hz, J = 2.4 Hz, 1H), 5.15 (d, J = 21.0 Hz, 1H), 3.79 (s, 3H). **^{13}C-NMR** (150 MHz, $CDCl_3$) δ 160.1, 159.0 (dd, J = 297.8 Hz, J = 292.7 Hz),136.5 (t, J = 2.2 Hz), 123.0, 119.7, 113.1, 112.0, 73.1 (dd, J = 32.2 Hz, J = 21.0 Hz), 55.3. **^{19}F NMR** (564 MHz, $CDCl_3$) δ -75.84 (d, J = 18.6 Hz), -78.80 (dd, J = 21.0 Hz, J = 18.6 Hz). **IR** (KBr): 3066, 1707, 1653, 1312, 1285, 1249, 1175, 770 cm^{-1}. **MS** (EI, m/z): 202 (M^+, 100.00), 157 (68.43). **HRMS** (EI): exact mass calcd for $C_9H_8F_2OS$ (M^+): 202.0264, found: 202.0268.

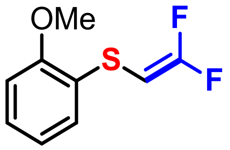

(2,2-difluorovinyl)(2-methoxyphenyl)sulfane (6) Colorless oil; **^{1}H-NMR** (600 MHz, $CDCl_3$) δ 7.22-7.18 (m, 2H), 6.94 (t, J = 7.6 Hz, 1H), 6.86 (d, J = 8.0 Hz, 1H), 5.10 (d, J = 21.0 Hz, 1H), 3.89 (s, 3H). **^{13}C-NMR** (150 MHz, $CDCl_3$) δ 159.1 (dd, J = 297.4 Hz, J = 292.4 Hz), 156.4, 127.7, 127.4, 123.7 (t, J = 2.2 Hz), 121.2, 110.6, 72.1 (dd, J = 31.8 Hz, J = 20.8 Hz), 55.8. **^{19}F NMR**

(564 MHz, $CDCl_3$) δ -75.31 (d, *J* = 19.6 Hz), -79.14 (t, *J* = 20.2 Hz). **IR** (KBr): 3054, 1710, 1653, 1312, 1221, 1175, 750 cm^{-1}. **MS** (EI, m/z): 202 (M^+, 100.00), 187 (36.23). **HRMS** (EI): exact mass calcd for $C_9H_8F_2OS$ (M^+): 202.0264, found: 202.0269.

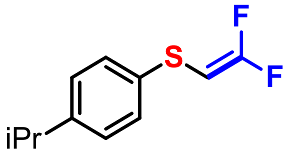

(2,2-difluorovinyl)(4-isopropylphenyl)sulfane (7) Colorless oil; **^{1}H-NMR** (600 MHz, $CDCl_3$) δ 7.25-7.23 (m, 2H), 7.18-7.16 (m, 2H), 5.12 (dd, *J* = 21.0 Hz, *J* = 1.0 Hz, 1H), 2.91-2.84 (m, 1H), 1.23 (d, *J* = 7.0 Hz, 6H). **^{13}C-NMR** (150 MHz, $CDCl_3$) δ 158.7 (dd, *J* = 297.6 Hz, *J* = 291.0 Hz) , 147.7, 131.8 (t, *J* = 2.0 Hz), 128.4, 127.3, 74.1 (dd, *J* = 31.2 Hz, *J* = 21.0 Hz), 33.7, 23.9. **^{19}F NMR** (564 MHz, $CDCl_3$) δ -77.00 (d, *J* = 21.0 Hz), -79.74 (t, *J* = 21.0 Hz). **IR** (KBr): 3069, 2958, 1706, 1316, 1175, 835 cm^{-1}. **MS** (EI, m/z): 214 (M^+, 100), 199 (42.25). **HRMS** (EI): exact mass calcd for $C_{11}H_{12}F_2S$ (M^+): 214.0628, found: 214.0631.

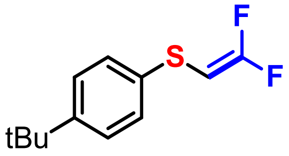

(4-(tert-butyl)phenyl)(2,2-difluorovinyl)sulfane (8) Colorless oil; **^{1}H-NMR** (600 MHz, $CDCl_3$) δ 7.34-7.32 (m, 2H), 7.26-7.24 (m, 2H), 5.12 (dd, *J* = 21.0 Hz, *J* = 1.0 Hz, 1H), 1.30 (s, 9H). **^{13}C-NMR** (150 MHz, $CDCl_3$) δ 158.7 (dd, *J* = 295.2 Hz, *J* = 290.0 Hz), 149.9, 131.6 (t, *J* = 2.0 Hz), 128.0, 126.2, 74.0 (dd, *J* = 31.5 Hz, *J* = 21.0 Hz), 34.5, 31.2. **^{19}F NMR** (564 MHz, $CDCl_3$) δ -77.05 (d, *J* = 21.0 Hz), -79.74 (t, *J* = 21.0 Hz). **IR** (KBr): 3078, 1705, 1650, 1314, 1174, 1117, 821 cm^{-1}. **MS** (EI, m/z): 228 (M^+, 36.23), 213 (100.00). **HRMS** (EI): exact mass calcd for $C_{12}H_{14}F_2S$ (M^+): 228.0784, found: 228.0785.

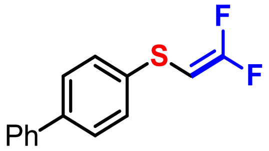

(1,1'-biphenyl)-4-yl(2,2-difluorovinyl)sulfane (9) Write solid, mp: 69-70 ℃; **^{1}H-NMR** (600 MHz, $CDCl_3$) δ 7.56-7.52 (m, 4H), 7.44-7.42 (m, 2H), 7.38-7.33 (m, 3H), 5.17 (dd, *J* = 21.0 Hz, *J* = 1.0 Hz, 1H). **^{13}C-NMR** (150 MHz, $CDCl_3$) δ 159.1 (dd, *J* = 296.0 Hz, *J* = 290.6 Hz), 140.2, 139.5, 134.2 (t, *J* = 2.2 Hz), 128.9, 128.1, 127.8, 127.5, 126.9, 73.4 (dd, *J* = 31.7 Hz, *J* = 20.7 Hz). **^{19}F NMR** (564 MHz, $CDCl_3$) δ -75.87 (d, *J* = 19.0 Hz), -78.78 (dd, *J* = 21.0 Hz, *J* = 19.0 Hz). **IR**

(KBr): 3059, 1711, 1478, 1304, 1175, 1116, 1085, 961, 827, 760, 690 cm^{-1}. **HRMS** (ESI) m/z calculated for $C_{14}H_{10}F_2NaS$ $[M+Na]^+$ 271.0369, found 271.0372.

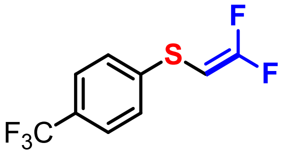

(2,2-difluorovinyl)(4-(trifluoromethyl)phenyl)sulfane (10) Colorless oil; **^{1}H-NMR** (600 MHz, $CDCl_3$) δ 7.54 (d, *J* = 8.4 Hz, 2H), 7.35 (d, *J* = 8.4 Hz, 2H), 5.16 (d, *J* = 21.0 Hz, 1H). **^{13}C-NMR** (150 MHz, $CDCl_3$) δ 159.8 (dd, *J* = 297.6 Hz, *J* = 292.2 Hz), 140.6, 128.2 (q, *J* = 21.6 Hz), 126.6, 125.9 (q, *J* = 3.6 Hz), 124.0 (q, *J* = 271.2 Hz), 71.7 (dd, *J* = 32.8 Hz, *J* = 21.0 Hz). **^{19}F NMR** (564 MHz, $CDCl_3$) δ -62.53, -75.87 (d, *J* = 13.0 Hz), -76.75 (dd, *J* = 21.0 Hz, *J* = 13.0 Hz). **IR** (KBr): 1716, 1180, 1152, 1132, 847 cm^{-1}. **MS** (EI, m/z): 240 (M^+, 100.00), 195 (86.32). **HRMS** (EI): exact mass calcd for $C_9H_5F_5S$ (M^+): 240.0032, found: 240.0041.

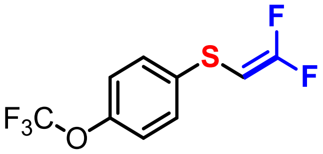

(2,2-difluorovinyl)(4-(trifluoromethoxy)phenyl)sulfane (11) Colorless oil; **^{1}H-NMR** (600 MHz, $CDCl_3$) δ 7.32-7.30 (m, 2H), 7.16 (d, *J* = 8.4 Hz, 2H), 5.13 (d, *J* = 21.0 Hz, 1H). **^{13}C-NMR** (150 MHz, $CDCl_3$) δ 159.4 (dd, *J* = 296.8 Hz, *J* = 291.4 Hz), 147.9 (q, *J* = 1.8 Hz), 134.0 (t, *J* = 2.4 Hz), 129.0, 121.8, 120.4 (q, *J* = 256.0 Hz), 73.0 (dd, *J* = 32.2 Hz, *J* = 20.8 Hz). **^{19}F NMR** (564 MHz, $CDCl_3$) δ -58.12, -75.17 (d, *J* = 16.4 Hz), -78.06 (dd, *J* = 21.0 Hz, *J* = 16.4 Hz). **IR** (KBr): 1708, 1491, 1220, 1178, 1088, 804 cm^{-1}. **MS** (EI, m/z): 256 (M^+, 100), 211 (87.63), 171 (30.19). **HRMS** (EI): exact mass calcd for $C_9H_5F_5OS$ (M^+): 255.9981, found: 255.9983

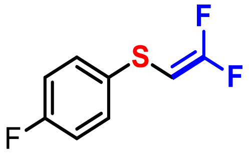

(2,2-difluorovinyl)(4-fluorophenyl)sulfane (12) Colorless oil; **^{1}H-NMR** (600 MHz, $CDCl_3$) δ 7.32-7.29 (m, 2H), 7.03-7.00 (m, 2H), 5.12 (dd, *J* = 21.0 Hz, *J* = 1.0 Hz, 1H). **^{13}C-NMR** (150 MHz, $CDCl_3$) δ 161.9 (d, *J* = 245.2 Hz), 158.9 (dd, *J* = 298.0 Hz, *J* = 290.7 Hz), 130.5 (d, *J* = 8.2 Hz), 130.1 (q, *J* = 2.4 Hz), 116.3 (d, *J* = 22.2 Hz), 74.2 (dd, *J* = 31.6 Hz, *J* = 21.0 Hz). **^{19}F NMR** (564 MHz, $CDCl_3$) δ -76.41 (d, *J* = 19.2 Hz), -79.02 (t, *J* = 21.0 Hz), -(115.13-115.17) (m). **IR**

(KBr): 3065, 1716, 1710, 1650, 1315 cm^{-1}. **MS** (EI, m/z): 190 (M^+, 85.30), 145 (100.00). **HRMS** (EI): exact mass calcd for $C_8H_5F_3S$ (M^+): 190.0064, found: 190.0066.

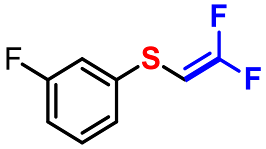

(2,2-difluorovinyl)(3-fluorophenyl)sulfane (13) Colorless oil; **^{1}H-NMR** (600 MHz, $CDCl_3$) δ 7.28-7.24 (m, 1H), 7.50 (d, *J* = 8.0 Hz, 1H), 7.35 (dt, *J* = 9.4 Hz, *J* = 2.0 Hz, 1H), 6.89 (td, *J* = 8.5 Hz, *J* = 2.0 Hz, 1H), 5.15 (d, *J* = 20.5 Hz, 1H). **^{13}C-NMR** (150 MHz, $CDCl_3$) δ 163.0 (d, *J* = 250.2 Hz), 159.5 (dd, *J* = 299.6 Hz, *J* = 294.0 Hz), 137.7 (td, *J* = 8.0 Hz, *J* = 2.6 Hz), 130.4 (d, *J* = 8.4 Hz), 122.7 (d, *J* = 3.0 Hz), 114.1 (d, *J* = 24.0 Hz), 113.3 (d, *J* = 21.4 Hz), 72.4 (dd, *J* = 32.4 Hz, *J* = 20.8 Hz). **^{19}F NMR** (564 MHz, $CDCl_3$) δ -74.55 (d, *J* = 15.2 Hz), -77.61 (dd, *J* = 20.5 Hz, *J* = 15.2 Hz), -(111.69-111.74) (m). **IR** (KBr): 3065, 1717, 1643, 1234, 1171, 902 cm^{-1}. **MS** (EI, m/z): 190 (M^+, 96.24), 145 (100.00). **HRMS** (EI): exact mass calcd for $C_8H_5F_3S$ (M^+): 190.0064, found: 190.0066.

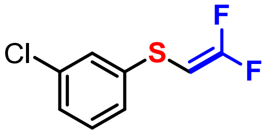

(3-chlorophenyl)(2,2-difluorovinyl)sulfane (14) Colorless oil; **^{1}H-NMR** (600 MHz, $CDCl_3$) δ 7.26 (t, *J* = 1.8 Hz, 1H), 7.22 (t, *J* = 7.8 Hz, 1H), 7.18-7.15 (m, 2H), 5.14 (d, *J* = 21.0 Hz, 1H). **^{13}C-NMR** (150 MHz, $CDCl_3$) δ 159.5 (dd, *J* = 298.4 Hz, *J* = 293.4 Hz), 137.4 (t, *J* = 2.4 Hz), 135.0, 130.1, 126.9, 126.5, 125.4, 72.4 (dd, *J* = 32.4 Hz, *J* = 21.0 Hz). **^{19}F NMR** (564 MHz, $CDCl_3$) δ -74.41 (d, *J* = 15.0 Hz), -77.54 (dd, *J* = 21.0 Hz, *J* = 15.0 Hz). **IR** (KBr): 3056, 1715, 1701, 1384, 1153, 912, 745 cm^{-1}. **MS** (EI, m/z): 206 (M^+, 100.00), 171 (92.07), 161 (86.07). **HRMS** (EI): exact mass calcd for $C_8H_5ClF_2S$ (M^+): 205.9769, found: 205.9771.

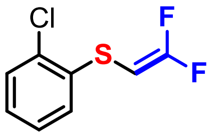

(2-chlorophenyl)(2,2-difluorovinyl)sulfane (15) Colorless oil; **^{1}H-NMR** (600 MHz, $CDCl_3$) δ 7.35 (d, *J* = 8.2 Hz, 1H), 7.25-7.22 (m, 2H), 7.15-7.12 (m, 1H), 5.12 (d, *J* = 21.0 Hz, 1H). **^{13}C-NMR** (150 MHz, $CDCl_3$) δ 159.7 (dd, *J* = 299.5 Hz, *J* = 294.0 Hz), 134.8 (t, *J* = 2.4 Hz), 132.0, 129.8, 127.3, 127.3, 127.0, 71.6 (dd, *J* = 32.4 Hz, *J* = 20.6 Hz). **^{19}F NMR** (564 MHz,

$CDCl_3$) δ -73.37 (d, *J* = 14.0 Hz), -77.19 (dd, *J* = 21.0 Hz, *J* = 14.0 Hz). **IR** (KBr): 3065, 1706, 1316, 1177, 1032, 745 cm^{-1}. **MS** (EI, m/z): 206 (M^+, 100.00), 171 (92.02), 161 (76.43). **HRMS** (EI): exact mass calcd for $C_8H_5ClF_2S$ (M^+): 205.9769, found: 205.9772.

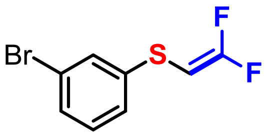

(3-bromophenyl)(2,2-difluorovinyl)sulfane (16) Colorless oil; **^{1}H-NMR** (600 MHz, $CDCl_3$) δ 7.41 (t, *J* = 1.8 Hz, 1H), 7.32 (d, *J* = 8.0 Hz, 1H), 7.21 (d, *J* = 8.0 Hz, 1H), 7.16 (t, *J* = 7.8 Hz, 1H), 5.14 (d, *J* = 21.0 Hz, 1H). **^{13}C-NMR** (150 MHz, $CDCl_3$) δ 159.4 (dd, *J* = 299.4 Hz, *J* = 293.2 Hz), 137.7 (t, *J* = 2.4 Hz), 130.4, 129.8, 129.4, 125.8, 123.1, 72.5 (dd, *J* = 32.8 Hz, *J* = 21.0 Hz). **^{19}F NMR** (564 MHz, $CDCl_3$) δ -74.36 (d, *J* = 15.2 Hz), -77.48 (dd, *J* = 21.0 Hz, *J* = 15.2 Hz). **IR** (KBr): 3065, 1713, 1650, 1261, 1177, 745, 653 cm^{-1}. **MS** (EI, m/z): 250 (M^+, 67.95), 205 (33.95), 171 (100.00). **HRMS** (EI): exact mass calcd for $C_8H_5BrF_2S$ (M^+): 249.9263, found: 249.9259.

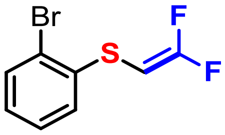

(2-bromophenyl)(2,2-difluorovinyl)sulfane (17) Colorless oil; **^{1}H-NMR** (600 MHz, $CDCl_3$) δ 7.52 (dd, *J* = 8.0 Hz, *J* =1.4 Hz, 1H), 7.29-7.26 (m, 1H), 7.22 (dd, *J* = 8.0 Hz, *J* = 1.6 Hz ,1H), 7.05 (td, *J* = 7.8 Hz, *J* = 1.6 Hz, 1H), 5.12 (d, *J* = 21.0 Hz, 1H). **^{13}C-NMR** (150 MHz, $CDCl_3$) δ 159.7 (dd, *J* = 300.2 Hz, *J* = 294.4 Hz), 136.8 (t, *J* = 2.4 Hz), 133.0, 127.9, 127.0, 121.4, 72.9 (dd, *J* = 32.0 Hz, *J* = 20.6 Hz). **^{19}F NMR** (564 MHz, $CDCl_3$) δ -73.32 (d, *J* = 13.4 Hz), -77.02 (dd, *J* = 21.0 Hz, *J* = 13.4 Hz). **IR** (KBr): 3063, 1704, 1448, 1311, 1171, 744 cm^{-1}. **MS** (EI, m/z): 250 (M^+, 65.65), 205 (35.40), 171 (100.00). **HRMS** (EI): exact mass calcd for $C_8H_5BrF_2S$ (M^+): 249.9263, found: 249.9270.

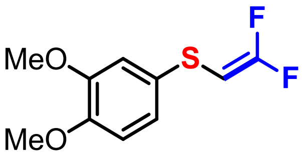

(2,2-difluorovinyl)(3,4-dimethoxyphenyl)sulfane (18) Colorless oil; **^{1}H-NMR** (600 MHz, $CDCl_3$) δ 6.94 (dd, *J* = 8.4 Hz, *J* = 2.0 Hz, 1H), 6.88 (d, *J* = 2.0 Hz, 1H), 6.81 (d, *J* = 8.4 Hz, 1H), 5.12 (dd, *J* = 22.2 Hz, *J* = 1.0 Hz, 1H), 3.88 (s, 3H), 3.86 (s, 3H). **^{13}C-NMR** (150 MHz, $CDCl_3$) δ 158.2 (dd, *J* = 296.4 Hz, *J* = 291.8 Hz) ,149.3, 148.7, 125.6 (t, *J* = 2.2 Hz), 122.3, 113.1, 111.8,

75.2 (dd, J = 30.8Hz, J = 21.0 Hz), 56.0, 55.9. **^{19}F NMR** (564 MHz, $CDCl_3$) δ -77.85 (d, J = 22.2 Hz), -80.18 (t, J = 22.2 Hz). **IR** (KBr): 3065, 1702, 1650, 1314, 1255, 1174, 1026, 745 cm^{-1}. **MS** (EI, m/z): 232 (M^+, 100.00), 217 (33.86), 187 (47.88). **HRMS** (EI): exact mass calcd for $C_{10}H_{10}F_2O_2S$ (M^+): 232.0370, found: 232.0373.

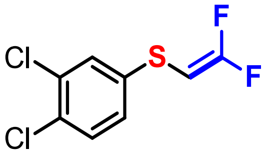

(3,4-dichlorophenyl)(2,2-difluorovinyl)sulfane (19) Colorless oil; **^{1}H-NMR** (600 MHz, $CDCl_3$) δ 7.37-7.35 (m, 2H), 7.11 (dd, J = 8.4 Hz, J =2.2 Hz, 1H), 5.13 (d, J = 20.6 Hz, 1H). **^{13}C-NMR** (150 MHz, $CDCl_3$) δ 159.6 (dd, J = 300.0 Hz, J = 293.4 Hz), 135.6 (t, J = 2.4 Hz), 133.3, 130.8, 130.5, 128.7, 126.6, 72.3 (dd, J = 32.6 Hz, J = 20.8 Hz). **^{19}F NMR** (564 MHz, $CDCl_3$) δ -73.75 (d, J = 13.6 Hz), -76.85 (dd, J = 20.6 Hz, J = 13.6 Hz). **IR** (KBr): 3064, 1705, 1651, 1317, 1178, 809, 743 cm^{-1}. **MS** (EI, m/z): 240 (M^+, 100.00), 205 (80.71), 195 (73.57), 170 (72.22). **HRMS** (EI): exact mass calcd for $C_8H_4Cl_2F_2S$ (M^+): 239.9379, found: 239.9387.

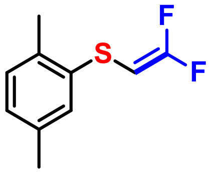

(2,2-difluorovinyl)(2,5-dimethylphenyl)sulfane (20) Brown oil; **^{1}H-NMR** (600 MHz, $CDCl_3$) δ 7.06-7.03 (m, 2H), 6.93 (d, J = 7.6 Hz, 1H), 5.06 (dd, J =21.0 Hz, J = 1.0 Hz, 1H), 2.33 (s, 3H), 2.31 (s, 3H). **^{13}C-NMR** (150 MHz, $CDCl_3$) δ 158.8 (dd, J = 296.8 Hz, J = 291.8 Hz), 136.3, 133.8 (t, J = 2.0 Hz), 133.5, 130.2, 128.0, 127.2, 73.2 (dd, J = 31.2 Hz, J = 20.8 Hz), 20.9, 19.4. **^{19}F NMR** (564 MHz, $CDCl_3$) δ -76.17 (d, J = 21.0 Hz), -79.75 (t, J = 21.0 Hz). **IR** (KBr): 3063, 1707, 1650, 1488, 1310, 1173 cm^{-1}. **MS** (EI, m/z): 200 (M^+, 79.64), 185 (19.41), 155 (100.00). **HRMS** (EI): exact mass calcd for $C_{10}H_{10}F_2S$ (M^+): 200.0471, found: 200.0474.

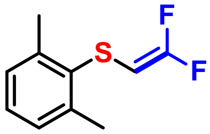

(2,2-difluorovinyl)(2,6-dimethylphenyl)sulfane (21) Colorless oil; **^{1}H-NMR** (600 MHz, $CDCl_3$) δ 7.15-7.14 (m, 1H), 7.09 (d, J = 7.2 Hz, 2H), 4.81 (d, J = 22.0 Hz, 1H), 2.53 (s, 6H). **^{13}C-NMR** (150 MHz, $CDCl_3$) δ 157.3 (dd, J = 295.4 Hz, J = 290.4 Hz), 142.8, 131.4, 128.9, 128.4, 75.7 (dd,

J = 29.6 Hz, J = 21.6 Hz), 21.8. **^{19}F NMR** (564 MHz, $CDCl_3$) δ -80.56 (d, J = 29.7 Hz), -81.46 (dd, J = 29.7 Hz, J = 22.0 Hz). **IR** (KBr): 3057, 1705, 1650, 1460, 1305, 1169, 1082, 772 cm^{-1}. **MS** (EI, m/z): 200 (M^+, 100.00), 185 (24.78), 155 (59.85). **HRMS** (EI): exact mass calcd for $C_{10}H_{10}F_2S$ (M^+): 200.0471, found: 200.0479.

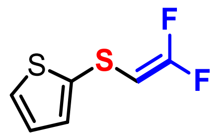

2-((2,2-difluorovinyl)thio)thiophene (22) Colorless oil; **^{1}H-NMR** (600 MHz, $CDCl_3$) δ 7.35 (dd, J = 5.4 Hz, J = 1.2 Hz, 1H), 7.15 (dd, J = 3.6 Hz, J = 1.2 Hz, 1H), 6.97 (dd, J = 5.4 Hz, J = 3.6 Hz, 1H), 5.13 (d, J = 21.0 Hz, 1H). **^{13}C-NMR** (150 MHz, $CDCl_3$) δ 158.0 (dd, J = 298.4 Hz, J = 292.6 Hz), 133.0, 132.6 (t, J = 2.6 Hz), 129.6, 127.6, 77.1 (dd, J = 30.0 Hz, J = 20.8 Hz). **^{19}F NMR** (564 MHz, $CDCl_3$) δ -78.28 (d, J = 21.0 Hz), -79.37 (t, J = 21.0 Hz). **IR** (KBr): 2921, 1715, 1649, 1510, 1224, 1125, 1087, 731 cm^{-1}. **MS** (EI, m/z): 178 (M^+, 79.64), 133 (43.49). **HRMS** (EI): exact mass calcd for $C_6H_4F_2S_2$ (M^+): 177.9722, found: 177.9725.

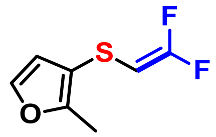

3-((2,2-difluorovinyl)thio)-2-methylfuran (23) Colorless oil; **^{1}H-NMR** (600 MHz, $CDCl_3$) δ 7.27 (d, J = 1.0 Hz, 1H), 6.34 (d, J = 1.0 Hz, 1H), 4.92 (d, J = 21.5 Hz, 1H), 2.35 (s, 3H). **^{13}C-NMR** (150 MHz, $CDCl_3$) δ 157.6 (dd, J = 296.2 Hz, J = 290.6 Hz), 154.8, 140.8, 114.3, 109.2 (t, J = 1.8 Hz), 76.0 (dd, J = 29.0 Hz, J = 21.4 Hz), 11.7. **^{19}F NMR** (564 MHz, $CDCl_3$) δ -79.69 (d, J = 27.3 Hz), -81.15 (dd, J = 27.3 Hz, J = 21.5 Hz). **IR** (KBr): 2922, 2852, 1717, 1650, 1459, 1218, 1150, 700 cm^{-1}. **MS** (EI, m/z): 176 (M+, 100.00), 161 (55.36). **HRMS** (EI): exact mass calcd for $C_7H_6F_2OS$ (M^+): 176.0107, found: 176.0109.

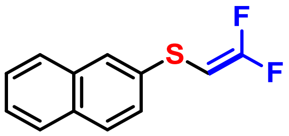

(2,2-difluorovinyl)(naphthalen-2-yl)sulfane (24) Colorless oil; **^{1}H-NMR** (600 MHz, $CDCl_3$) δ 7.79-7.71 (m, 4H), 7.49-7.42 (m, 2H), 7.37 (dd, J = 8.6 Hz, J = 2.0 Hz, 1H), 5.23 (d, J = 21.0 Hz, 1H). **^{13}C-NMR** (150 MHz, $CDCl_3$) δ 159.1 (dd, J = 298.0 Hz, J = 292.6 Hz), 133.7, 132.6 (t, J = 2.0 Hz), 131.9, 128.8, 127.8, 127.1, 126.8, 125.9, 125.7, 125.7, 73.4 (dd, J = 31.8 Hz, J = 20.8 Hz).

^{19}**F NMR** (564 MHz, $CDCl_3$) δ -75.69 (d, *J* = 18.5 Hz), -78.61 (dd, *J* = 21.0 Hz, *J* = 18.5 Hz). **IR** (KBr): 3058, 1706, 1625, 1502, 1312, 1714, 958, 913, 744 cm^{-1}. **MS** (EI, m/z): 222 (M^+, 100.00), 177 (80.22). **HRMS** (EI): exact mass calcd for $C_{12}H_8F_2S$ (M^+): 222.0315, found: 222.0320.

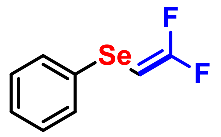

(2,2-difluorovinyl)(phenyl)selane (25) Colorless oil; ^{1}**H-NMR** (600 MHz, $CDCl_3$) δ 7.45-7.43 (m, 2H), 7.30-7.25 (m, 3H), 5.24 (dd, *J* = 23.5 Hz, *J* = 2.4 Hz, 1H). ^{13}**C-NMR** (150 MHz, $CDCl_3$) δ 157.7 (dd, *J* = 298.6 Hz, *J* = 287.8 Hz), 130.9, 130.0 (t, *J* = 1.5 Hz), 129.3, 127.2, 66.0 (t, *J* = 25.8 Hz). ^{19}**F NMR** (564 MHz, $CDCl_3$) δ -73.40 (dd, *J* = 23.5 Hz, *J* = 2.5 Hz), -76.59 (t, *J* = 23.5 Hz). **IR** (KBr): 3076, 1700, 1650, 1293, 1260, 1150 cm^{-1}. **MS** (EI, m/z): 220 (M^+, 100). **HRMS** (EI): exact mass calcd for $C_8H_6F_2Se$ (M^+): 219.9603, found: 219.9607.

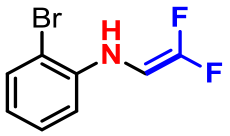

2-bromo-N-(2,2-difluorovinyl)aniline (26) Colorless oil; ^{1}**H-NMR** (600 MHz, $CDCl_3$) δ 7.45 (dd, *J* = 7.9 Hz, *J* = 1.4 Hz, 1H), 7.22-7.19 (m, 1H), 6.72 (dd, *J* = 8.1 Hz, *J* = 1.1 Hz, 1H), 6.69 (td, *J* = 7.6 Hz, *J* = 1.4 Hz, 1H), 5.61-5.56 (m, 1H), 5.30 (s, 1H). ^{13}**C-NMR** (150 MHz, $CDCl_3$) δ 155.0 (dd, *J* = 287.7 Hz, *J* = 280.2 Hz) , 141.3 (d, *J* = 1.5 Hz), 132.7, 128.6, 119.9, 112.2, 110.0 (d, *J* = 1.7 Hz), 86.0 (dd, *J* = 46.9 Hz, *J* = 16.3 Hz). ^{19}**F NMR** (564 MHz, $CDCl_3$) δ -96.55 (dd, *J* = 65.7 Hz, *J* = 18.0 Hz), -108.92 (d, *J* = 65.7 Hz). **IR** (KBr): 3400, 3066, 2926, 1596, 1510, 1310, 1149, 1023, 912, 743 cm^{-1}. **HRMS** (ESI) m/z calculated for $C_8H_6BrF_2NNa$ $[M+Na]^+$ 255.9549, found 255.9553.

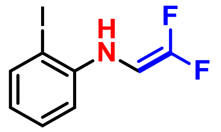

N-(2,2-difluorovinyl)-2-iodoaniline (27) Colorless oil; ^{1}**H-NMR** (600 MHz, $CDCl_3$) δ 7.68 (dd, *J* = 7.8 Hz, *J* = 1.3 Hz, 1H), 7.24-7.22 (m, 1H), 6.67 (d, *J* = 8.2 Hz, 1H), 6.55 (td, *J* = 7.8 Hz, *J* = 1.2 Hz, 1H), 5.59-5.54 (m, 1H), 5.16 (d, *J* = 5.4 Hz, 1H). ^{13}**C-NMR** (150 MHz, $CDCl_3$) δ 155.0

(dd, J = 287.3 Hz, J = 280.5 Hz), 143.7, 139.3, 129.6, 120.8, 111.7, 86.4 (dd, J = 46.8 Hz, J = 16.0 Hz), 85.3 (d, J = 1.7 Hz). **^{19}F NMR** (564 MHz, $CDCl_3$) δ -96.37 (dd, J = 65.2 Hz, J = 17.8 Hz), -108.80 (d, J = 65.0 Hz). **IR** (KBr): 3411, 2925, 1586, 1514, 1299, 1147, 749 cm^{-1}. **HRMS** (ESI) m/z calculated for $C_8H_6F_2INNa$ $[M+Na]^+$ 303.9411, found 303.9417.

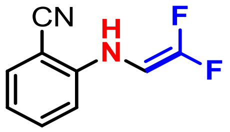

2-((2,2-difluorovinyl)amino)benzonitrile (28) Yellow oil; **^{1}H-NMR** (600 MHz, $CDCl_3$) δ 7.48-7.43 (m, 2H), 6.84 (t, J = 7.6 Hz, 1H), 6.78 (d, J = 8.5 Hz, 1H), 5.61-5.57 (m, 1H), 5.44 (s, 1H). **^{13}C-NMR** (150 MHz, $CDCl_3$) δ 155.5 (dd, J = 289.5 Hz, J = 282.9 Hz), 146.7 (d, J = 1.8 Hz), 134.4, 132.9, 119.0, 117.0, 111.8, 97.2 (d, J = 1.2 Hz), 84.9 (dd, J = 47.4 Hz, J = 16.3 Hz). **^{19}F NMR** (564 MHz, $CDCl_3$) δ -93.59 (dd, J = 59.3 Hz, J = 18.0 Hz), -106.09-(-106.19) (dd, J = 59.3 Hz, J = 2.0 Hz). **IR** (KBr): 3420, 2924, 2853, 2218, 1741, 1606, 1584, 1515, 1271, 1153, 750 cm^{-1}. **HRMS** (ESI) m/z calculated for $C_9H_6F_2NaN_2$ $[M+Na]^+$ 203.0397, found 203.0394.

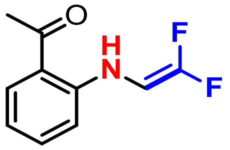

1-(2-((2,2-difluorovinyl)amino)phenyl)ethanone (29) Yellow oil; **^{1}H-NMR** (600 MHz, $CDCl_3$) δ 9.93 (s, 1H), 7.78 (d, J = 7.8 Hz, 1H), 7.40 (t, J = 7.8 Hz, 1H), 6.78 (d, J = 8.4 Hz, 1H), 6.74 (t, J = 7.5 Hz, 1H), 5.69 (dd, J = 18.4 Hz, J = 8.0 Hz, 1H), 2.60 (s, 3H). **^{13}C-NMR** (150 MHz, $CDCl_3$) δ 201.3, 154.7 (dd, J = 288.2 Hz, J = 279.1 Hz) , 147.2 (d, J = 1.9 Hz), 135.1, 132.6, 118.7, 116.5, 112.2, 85.0 (dd, J = 46.9 Hz, J = 16.2 Hz), 27.8. **^{19}F NMR** (564 MHz, $CDCl_3$) δ -95.64 (dd, J = 63.6 Hz, J = 18.4 Hz), -109.18 (d, J = 63.6 Hz). **IR** (KBr): 1740, 1715, 1700, 1311, 1205, 1034, 769 cm^{-1}. **HRMS** (ESI) m/z calculated for $C_{10}H_9F_2NaNO$ $[M+Na]^+$ 220.0550, found 220.0557.

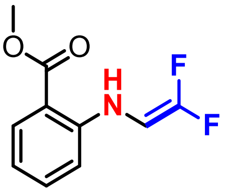

Methyl 2-((2,2-difluorovinyl)amino)benzoate (30) Yellow oil; **^{1}H-NMR** (600 MHz, $CDCl_3$) δ 8.83 (d, J = 8.0 Hz, 1H), 7.92 (d, J = 8.0 Hz, 1H), 7.39 (t, J = 7.8 Hz, 1H), 6.76 (d, J = 8.4 Hz, 1H), 6.72 (t, J = 7.5 Hz, 1H), 5.68 (dd, J = 18.4 Hz, J = 8.0 Hz, 1H), 3.88 (s, 3H). **^{13}C-NMR** (150 MHz,

$CDCl_3$) δ 168.9, 154.7 (dd, *J* = 288.5 Hz, *J* = 279.8 Hz), 147.3 (d, *J* = 1.8 Hz), 134.7, 131.6, 116.9, 111.8, 111.4, 85.2 (dd, *J* = 46.9 Hz, *J* = 16.0 Hz), 51.8. **^{19}F NMR** (564 MHz, $CDCl_3$) δ -96.55 (dd, *J* = 65.6 Hz, *J* = 18.5 Hz), -109.75 (d, *J* = 65.6 Hz). **IR** (KBr): 3489, 3315, 2954, 1763, 1691, 1587, 1518, 1439, 1249, 1163, 932, 750, 700 cm^{-1}. **HRMS** (ESI) m/z calculated for $C_{10}H_9F_2NaNO_2$ $[M+Na]^+$ 236.0499, found 236.0494.

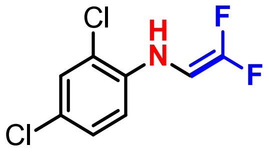

2,4-dichloro-N-(2,2-difluorovinyl)aniline (31) Colorless oil; **^{1}H-NMR** (600 MHz, $CDCl_3$) δ 7.29 (d, *J* = 2.4 Hz, 1H), 7.13 (dd, *J* = 9.0 Hz, *J* = 2.4 Hz, 1H), 6.65 (d, *J* = 9.0 Hz 1H), 5.54 (ddd, *J* = 18.0 Hz, *J* = 7.8 Hz, *J* = 1.8 Hz, 1H), 5.23 (d, *J* = 7.8 Hz, 1H). **^{13}C-NMR** (150 MHz, $CDCl_3$) δ 155.2 (dd, *J* = 287.2 Hz, *J* = 281.2 Hz), 139.2 (d, *J* = 1.7 Hz), 129.1, 127.9, 123.6, 120.0, 112.7, 85.6 (dd, *J* = 47.2 Hz, *J* = 16.4 Hz). **^{19}F NMR** (564 MHz, $CDCl_3$) δ -95.68 (dd, *J* = 63.2 Hz, *J* = 18.0 Hz), -107.91 (d, *J* = 63.2 Hz). **IR** (KBr): 3414, 2926, 1700, 1598, 1507, 1394, 1314, 1187, 868, 800 cm^{-1}. **HRMS** (ESI) m/z calculated for $C_8H_5Cl_2F_2NaN$ $[M+Na]^+$ 245.9665, found 245.9670.

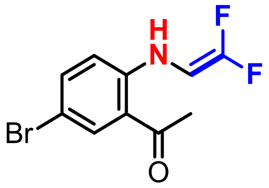

1-(5-bromo-2-((2,2-difluorovinyl)amino)phenyl)ethanone (32) Yellow oil; **^{1}H-NMR** (600 MHz, $CDCl_3$) δ 9.86 (d, *J* = 5.3 Hz, 1H), 7.85 (s. 1H), 7.45 (d, *J* = 8.4 Hz, 1H), 6.68 (dd, *J* = 8.4 Hz, *J* = 2.4 Hz, 1H), 5.63 (dd, *J* = 18.2 Hz, *J* = 7.8 Hz, 1H), 2.58 (d, *J* = 1.4 Hz, 3H). **^{13}C-NMR** (150 MHz, $CDCl_3$) δ 200.2, 154.8 (dd, *J* = 289.6 Hz, *J* = 280.4 Hz), 146.1, 137.6, 134.7, 119.9, 114.2, 107.9, 84.7 (dd, *J* = 47.3 Hz, *J* = 16.4 Hz), 27.8. **^{19}F NMR** (564 MHz, $CDCl_3$) δ -94.42 (dd, *J* = 60.0 Hz, *J* = 18.2 Hz), -107.79 (d, *J* = 60.0 Hz). **IR** (KBr): 1739, 1716, 1699, 1309, 1153, 1033 cm^{-1}. **HRMS** (ESI) m/z calculated for $C_{10}H_8BrF_2NaNO$ $[M+Na]^+$ 297.9655, found 297.9659.

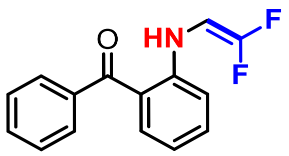

(2-((2,2-difluorovinyl)amino)phenyl)(phenyl)methanone (33) Yellow oil; **^{1}H-NMR** (600 MHz, $CDCl_3$) δ 9.54 (d, *J* = 8.0 Hz, 1H), 7.62 (d, *J* = 7.2 Hz, 2H), 7.53 (t, *J* = 7.2 Hz, 1H), 7.47-7.41 (m, 3H), 6.86 (d, *J* = 8.4 Hz, 1H), 6.70 (d, *J* = 7.8 Hz, 1H), 5.74 (ddd, *J* = 18.4 Hz, *J* = 8.0 Hz, *J* = 1.2 Hz, 1H). **^{13}C-NMR** (150 MHz, $CDCl_3$) δ 199.4, 154.8 (dd, *J* = 288.9 Hz, *J* = 279.4 Hz), 147.8 (d, *J* = 1.9 Hz), 139.7, 135.3, 135.0, 131.3, 129.2, 128.1, 118.6, 116.3, 112.3, 85.2 (dd, *J* = 46.9 Hz, *J* = 16.2 Hz). **^{19}F NMR** (564 MHz, $CDCl_3$) δ -95.47 (dd, *J* = 63.2 Hz, *J* = 18.4 Hz), -109.01 (d, *J* = 63.2 Hz). **IR** (KBr): 3272, 3080, 2925, 1759, 1625, 1579, 1517, 1454, 1260, 1166, 937, 749, 700 cm^{-1}. **HRMS** (ESI) m/z calculated for $C_{15}H_{11}F_2NaNO$ $[M+Na]^+$ 282.0706 found 282.0714.

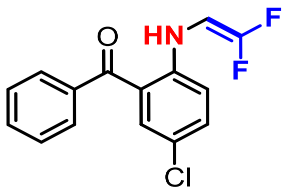

(5-chloro-2-((2,2-difluorovinyl)amino)phenyl)(phenyl)methanone (34) Yellow oil; **^{1}H-NMR** (600 MHz, $CDCl_3$) δ 9.42 (d, *J* = 7.8 Hz, 1H), 7.62 (d, *J* = 7.4 Hz, 2H), 7.56 (t, *J* = 7.4 Hz, 1H), 7.50-7.47 (m, 3H), 7.67 (dd, *J* = 9.0 Hz, *J* = 2.4 Hz, 1H), 6.82 (d, *J* = 9.0 Hz, 1H), 5.68 (dd, *J* = 18.2 Hz, *J* = 7.8 Hz, 1H). **^{13}C-NMR** (150 MHz, $CDCl_3$) δ 198.3, 154.9 (dd, *J* = 289.3 Hz, *J* = 281.1 Hz), 146.3 (d, *J* = 2.2 Hz), 138.9, 134.8, 134.0, 131.7, 129.2, 128.4, 121.1, 119.4, 114.0, 85.0 (dd, *J* = 47.1 Hz, *J* = 16.4 Hz). **^{19}F NMR** (564 MHz, $CDCl_3$) δ -94.41 (dd, *J* = 60.6 Hz, *J* = 18.2 Hz), -107.83 (d, *J* = 60.6 Hz). **IR** (KBr): 1740, 1610, 1560, 1261, 1170, 933, 750 cm^{-1}. **HRMS** (ESI) m/z calculated for $C_{15}H_{10}ClF_2NaNO$ $[M+Na]^+$ 316.0317, found 316.0321.

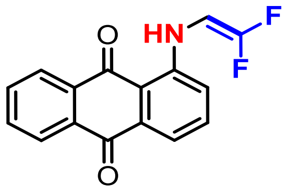

1-((2,2-difluorovinyl)amino)anthracene-9,10-dione (35) Red solid, mp: 104-105 ℃; **^{1}H-NMR** (600 MHz, $CDCl_3$) δ 10.71 (s, 1H), 8.31-8.21 (m, 2H), 7.79-7.70 (m, 3H), 7.59 (t, *J* = 8.0 Hz 1H), 7.12 (d, *J* = 8.0 Hz, 1H), 5.80 (dd, *J* = 18.0 Hz, *J* = 7.9 Hz, 1H). **^{13}C-NMR** (150 MHz, $CDCl_3$) δ 185.7, 183.2, 154.8 (dd, *J* = 290.2 Hz, *J* = 281.6 Hz), 147.8, 135.5, 134.7, 134.5, 134.1, 133.5, 132.9, 126.9, 126.9, 118.0, 117.7, 114.4, 84.7 (dd, *J* = 47.4 Hz, *J* = 16.3 Hz). **^{19}F NMR** (564 MHz, $CDCl_3$) δ -93.55 (dd, *J* = 58.0 Hz, *J* = 18.0 Hz), -107.02 (d, *J* = 58.0 Hz); **IR** (KBr): 3066, 1625,

1500, 1246, 1162, 1088, 931, 751 cm^{-1}. **HRMS** (ESI) m/z calculated for $C_{29}H_{34}Na$ $[M+Na]^+$ 308.0494, found 308.0476.

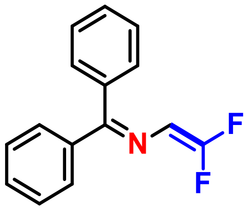

N-(diphenylmethylene)-2,2-difluoroethenamine (36) Yellow oil; ^{1}**H-NMR** (600 MHz, $CDCl_3$) δ7.68 (d, *J* = 7.8 Hz, 2H), 7.50-7.45 (m, 3H), 7.39 (t, *J* = 7.2 Hz 1H), 7.34 (t, *J* = 7.6 Hz, 2H), 7.19 (d, *J* = 7.2 Hz, 2H), 5.98 (d, *J* = 17.4 Hz, 1H). ^{13}**C-NMR** (150 MHz, $CDCl_3$) δ 165.9 (dd, *J* = 12.4 Hz, *J* = 3.5 Hz), 158.2 (dd, *J* = 304.3 Hz, *J* = 286.1 Hz), 139.0, 135.4, 130.4, 129.0, 128.7, 128.7, 128.4, 128.2, 96.6 (dd, *J* = 44.3 Hz, *J* = 10.0 Hz). ^{19}**F NMR** (564 MHz, $CDCl_3$) δ -85.48 (dd, *J* = 19.6 Hz, *J* = 17.4 Hz), -95.46 (d, *J* = 19.6 Hz). The spectroscopic data match with those reported in the literature[3].

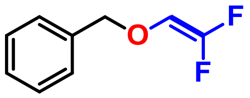

(((2,2-difluorovinyl)oxy)methyl)benzene (38) Colorless oil; ^{1}**H-NMR** (600 MHz, $CDCl_3$) δ 7.30-7.28 (m, 2H), 7.26-7.24 (m, 3H), 5.59 (dd, *J* = 16.2 Hz, *J* = 2.8 Hz, 1H), 4.64 (s, 2H). ^{13}**C-NMR** (150 MHz, $CDCl_3$) δ 155.5 (dd, *J* = 288.4 Hz, *J* = 276.2 Hz) , 136.2, 128.6, 128.4, 127.9, 107.5 (dd, *J* = 53.0 Hz, *J* = 15.2 Hz), 75.1 (t, *J* = 1.4 Hz). ^{19}**F NMR** (564 MHz, $CDCl_3$) δ -99.97 (dd, *J* = 78.6 Hz, *J* = 16.2 Hz), -120.34 (dd, *J* = 78.6 Hz, *J* = 2.8 Hz). **IR** (KBr): 2925, 2854, 1622, 1311, 1244, 1170 cm^{-1}. **HRMS** (EI) m/z calculated for $C_9H_8F_2O$ (M^+) 170.0543, found 170.0546.

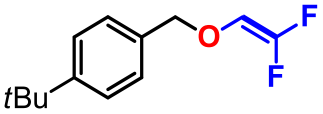

1-(tert-butyl)-4-(((2,2-difluorovinyl)oxy)methyl)benzene (39) Colorless oil; ^{1}**H-NMR** (600 MHz, $CDCl_3$) δ 7.40 (d, *J* = 8.2 Hz, 2H), 7.27 (d, *J* = 8.2 Hz, 2H), 5.68 (dd, *J* = 16.2 Hz, *J* = 3.0 Hz, 1H), 4.70 (s, 2H), 1.32 (s, 9H). ^{13}**C-NMR** (150 MHz, $CDCl_3$) δ 155.4 (dd, *J* = 288.8 Hz, *J* = 275.4 Hz) ,151.5, 133.2, 127.8, 125.5, 107.6 (dd, *J* = 52.8 Hz, *J* = 15.2 Hz), 74.9 (t, *J* = 1.5 Hz),

34.6, 31.3. ^{19}**F NMR** (564 MHz, $CDCl_3$) δ -100.12 (dd, *J* = 79.0 Hz, *J* = 16.2 Hz), -120.56 (dd, *J* = 79.0 Hz, *J* = 3.0 Hz). **IR** (KBr): 2961, 2926, 1768, 1618, 1270, 1241, 1171, 835 cm^{-1}. **MS** (EI, m/z): 226 (M^+, 0.04), 147 (100.00). **HRMS** (EI) m/z calculated for $C_{13}H_{16}F_2O$ (M^+) 226.1169, found 226.1172.

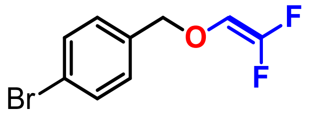

1-bromo-4-(((2,2-difluorovinyl)oxy)methyl)benzene (40) Colorless oil; ^{1}**H-NMR** (600 MHz, $CDCl_3$) δ 7.42 (d, *J* = 8.4 Hz, 2H), 7.13 (d, *J* = 8.4 Hz, 2H), 5.58 (dd, *J* = 16.0 Hz, *J* = 3.0 Hz, 1H), 4.60 (s, 2H). ^{13}**C-NMR** (150 MHz, $CDCl_3$) δ 155.5 (dd, *J* = 288.2 Hz, *J* = 276.8 Hz), 135.2, 131.8, 129.5, 122.4, 107.5 (dd, *J* = 52.8 Hz, *J* = 15.4 Hz), 74.3 (t, *J* = 1.4 Hz). ^{19}**F NMR** (564 MHz, $CDCl_3$) δ -99.40 (dd, *J* = 77.2 Hz, *J* = 16.0 Hz), -119.70 (dd, *J* = 77.0 Hz, *J* = 3.0 Hz). **IR** (KBr): 2925, 2856, 1625, 1341, 1310, 1244, 1171, 797, 580 cm^{-1}. **MS** (EI, m/z): 248 (M^+, 0.29), 169 (100.00). **HRMS** (EI) m/z calculated for $C_9H_7BrF_2O$ (M^+) 247.9648, found 247.9644.

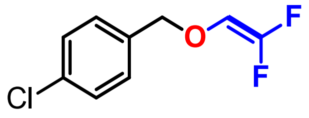

1-chloro-4-(((2,2-difluorovinyl)oxy)methyl)benzene (41) Colorless oil; ^{1}**H-NMR** (600 MHz, $CDCl_3$) δ 7.27 (d, *J* = 8.2 Hz, 2H), 7.19 (d, *J* = 8.2 Hz, 2H), 5.58 (dd, *J* = 16.0 Hz, *J* = 3.0 Hz, 1H), 4.62 (s, 2H). ^{13}**C-NMR** (150 MHz, $CDCl_3$) δ 155.5 (dd, *J* = 289.3 Hz, *J* = 276.9 Hz), 134.7, 134.3, 129.2, 128.8, 107.5 (dd, *J* = 53.2 Hz, *J* = 15.4 Hz), 74.3 (t, *J* = 1.5 Hz). ^{19}**F NMR** (564 MHz, $CDCl_3$) δ -99.46 (dd, *J* = 77.8 Hz, *J* = 16.0 Hz), -119.76 (dd, *J* = 77.8 Hz, *J* = 3.0 Hz). **IR** (KBr): 2924, 2855, 1626, 1310, 1244, 1711, 1150, 802 cm^{-1}. **MS** (EI, m/z): 204 (M^+, 0.20), 125 (100.00). **HRMS** (EI) m/z calculated for $C_9H_7ClF_2O$ (M^+) 204.0153, found 204.0160.

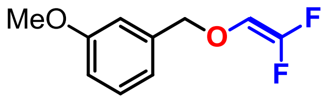

1-(((2,2-difluorovinyl)oxy)methyl)-3-methoxybenzene (42) Colorless oil; ^{1}**H-NMR** (600 MHz, $CDCl_3$) δ 7.20 (t, *J* = 7.8 Hz, 1H), 6.83-6.77 (m, 3H), 5.59 (dd, *J* = 16.2 Hz, *J* = 3.0 Hz, 1H), 4.62 (s, 2H), 3.72 (s, 3H). ^{13}**C-NMR** (150 MHz, $CDCl_3$) δ 159.9, 155.4 (dd, *J* = 289.0 Hz, *J* = 275.8 Hz) , 137.8, 129.6, 120.0, 114.0, 113.1, 107.6 (dd, *J* = 53.0 Hz, *J* = 15.2 Hz), 74.9 (t, *J* = 1.4 Hz),

55.2. **^{19}F NMR** (564 MHz, $CDCl_3$) δ -99.96 (dd, *J* = 78.6 Hz, 16.2 Hz), -120.31 (dd, *J* = 78.6 Hz, *J* = 3.0 Hz). **IR** (KBr): 2924, 2855, 1647, 1313, 1269, 1241, 1170, 1044, 783, 692 cm^{-1}. **MS** (EI, m/z): 200 (M^+, 3.56), 121 (100.00). **HRMS** (EI): exact mass calcd for $C_{10}H_{10}F_2O_2$ (M^+): 200.0649, found: 200.0650.

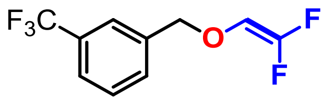

1-(((2,2-difluorovinyl)oxy)methyl)-3-(Trifluoroacetaldehyde)benzene (43) Colorless oil; **^{1}H-NMR** (600 MHz, $CDCl_3$) δ 7.51 (d, *J* = 8.8 Hz, 2H), 7.45-7.40 (m, 2H), 5.62 (dd, *J* = 16.0 Hz, *J* = 3.0 Hz, 1H), 4.69 (s, 2H). **^{13}C-NMR** (150 MHz, $CDCl_3$) δ 155.7 (dd, *J* = 288.8 Hz, *J* = 277.0 Hz), 137.3, 131.1 (q, *J* = 32.4 Hz), 131.0, 129.1, 125.2 (q, *J* = 3.8 Hz), 124.4 (q, *J* = 3.6 Hz), 124.0 (q, *J* = 272.9 Hz), 107.6 (dd, *J* = 53.1 Hz, *J* = 15.2 Hz), 74.3 (t, *J* = 1.5 Hz). **^{19}F NMR** (564 MHz, $CDCl_3$) δ -62.80, -99.16 (dd, *J* = 76.5 Hz, *J* = 16.0 Hz), -119.53 (dd, *J* = 76.5 Hz, *J* = 3.0 Hz). **IR** (KBr): 2924, 2855, 1647, 1635, 1310, 1241, 1171, 1033, 768, 698 cm^{-1}. **MS** (EI, m/z): 238 (M^+, 0.20), 159 (100.00). **HRMS** (EI) m/z calculated for $C_{10}H_7F_5O$ (M^+) 238.0417, found 238.0424.

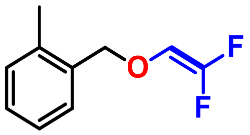

1-(((2,2-difluorovinyl)oxy)methyl)-2-methylbenzene (44) Colorless oil; **^{1}H-NMR** (600 MHz, $CDCl_3$) δ 7.28-7.23 (m, 2H), 7.20-7.17 (m, 2H), 5.68 (dd, *J* = 16.4 Hz, *J* = 3.0 Hz, 1H), 4.74 (s, 2H), 2.35 (s, 3H). **^{13}C-NMR** (150 MHz, $CDCl_3$) δ 155.5 (dd, *J* = 288.4 Hz, *J* = 276.8 Hz), 137.2, 134.1, 130.5, 129.1, 128.7, 125.9, 107.4 (dd, *J* = 52.8 Hz, *J* = 15.8 Hz), 73.7 (t, *J* = 1.2 Hz), 18.7. **^{19}F NMR** (564 MHz, $CDCl_3$) δ -100.10 (dd, *J* = 78.6 Hz, *J* = 16.4 Hz), -120.28 (dd, *J* = 78.6 Hz, *J* = 3.0 Hz). **IR** (KBr): 2924, 2855, 1635, 1623, 1340, 1310, 1242, 1171, 1150, 743 cm^{-1}. **MS** (EI, m/z): 184 (M^+, 0.16), 105 (100.00). **HRMS** (EI) m/z calculated for $C_{10}H_{10}F_2O$ (M^+) 184.0700, found 184.0702.

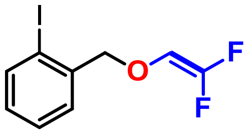

1-(((2,2-difluorovinyl)oxy)methyl)-2-iodobenzene (45) Colorless oil; **^{1}H-NMR** (600 MHz, $CDCl_3$) δ 7.85 (d, *J* = 8.0 Hz, 1H), 7.42 (d, *J* = 7.6 Hz, 1H), 7.38 (t, *J* = 7.6 Hz, 1H), 7.05-7.02 (m,

1H), 5.77 (dd, J = 16.0 Hz, J = 3.0 Hz, 1H), 4.76 (s, 2H). **^{13}C-NMR** (150 MHz, $CDCl_3$) δ 155.5 (dd, J = 289.6 Hz, J = 277.0 Hz) , 139.4, 138.6, 129.9, 129.0, 128.4, 107.9 (dd, J = 53.6 Hz, J = 15.4 Hz), 97.6, 78.7 (t, J = 1.4 Hz). **^{19}F NMR** (564 MHz, $CDCl_3$) δ -99.14 (dd, J = 77.0 Hz, J = 16.0 Hz), -119.86 (dd, J = 77.0 Hz, J = 3.0 Hz). **IR** (KBr): 2923, 2854, 1647, 1637, 1243, 1176, 1013, 747 cm^{-1}. **MS** (EI, m/z): 296 (M^+, 1.28), 217 (100.00). **HRMS** (EI): exact mass calcd for $C_9H_7F_2IO$ (M^+): 295.9510, found: 295.9508.

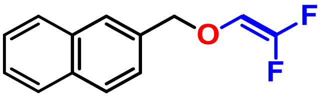

2-(((2,2-difluorovinyl)oxy)methyl)naphthalene (46) Colorless oil; **^{1}H-NMR** (600 MHz, $CDCl_3$) δ 7.71-7.68 (m, 3H), 7.62, (s, 1H), 7.36-7.34 (m, 2H), 7.30 (dd, J = 8.4 Hz, J = 1.5 Hz, 1H), 5.57 (dd, J = 16.2 Hz, J = 3.0 Hz, 1H), 4.71 (s, 2H). **^{13}C-NMR** (150 MHz, $CDCl_3$) δ 155.5 (dd, J = 288.8 Hz, J = 275.8 Hz), 133.6, 133.2, 133.1, 128.5, 128.0, 127.7, 127.0, 126.3, 126.3, 125.5, 107.6 (dd, J = 52.8 Hz, J = 15.2 Hz), 75.1 (t, J = 1.1 Hz). **^{19}F NMR** (564 MHz, $CDCl_3$) δ -99.78 (dd, J = 78.4 Hz, J = 16.2 Hz), -120.11 (dd, J = 78.4 Hz, J = 3.0 Hz). **IR** (KBr): 2925, 2983, 1637, 1334, 1213, 1069 cm^{-1}. **HRMS** (EI): exact mass calcd for $C_{13}H_{10}F_2O$ (M^+): 220.0700, found: 220.0703.

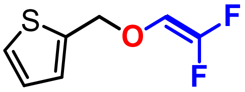

2-(((2,2-difluorovinyl)oxy)methyl)thiophene (47) Yellow oil; **^{1}H-NMR** (600 MHz, $CDCl_3$) δ 7.35 (d, J = 5.0 Hz, 1H), 7.06, (d, J = 3.9 Hz, 1H), 7.00 (t, J = 4.4 Hz, 1H), 5.69 (dd, J = 16.0 Hz, J = 2.8 Hz, 1H), 4.88 (s, 2H). **^{13}C-NMR** (150 MHz, $CDCl_3$) δ 155.6 (dd, J = 289.6 Hz, J = 277.2 Hz),138.3, 127.9, 127.0, 126.9, 106.8 (dd, J = 53.0 Hz, J = 15.0 Hz), 69.2 (t, J = 1.5 Hz). **^{19}F NMR** (564 MHz, $CDCl_3$) δ -99.23 (dd, J = 76.4 Hz, J = 16.0 Hz), -119.32 (dd, J = 76.4 Hz, J = 2.8 Hz). **IR** (KBr): 1437, 1244, 1220, 1105 cm^{-1}. **MS** (EI, m/z): 176 (M^+, 0.14), 97 (100.00). **HRMS** (EI): exact mass calcd for $C_7H_6F_2OS$ (M^+): 176.0107, found: 176.0111.

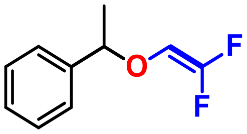

(1-((2,2-difluorovinyl)oxy)ethyl)benzene (48) Colorless oil; **^{1}H-NMR** (600 MHz, $CDCl_3$) δ 7.29-7.26 (m, 2H), 7.24-7.21 (m, 3H), 5.44 (dd, J = 16.3 Hz, J = 3.0 Hz, 1H), 4.58 (q, J = 6.6 Hz,

1H), 1.46 (d, *J* = 6.6 Hz, 3H). ^{13}**C-NMR** (150 MHz, $CDCl_3$) δ 155.4 (dd, *J* = 288.8 Hz, *J* = 275.8 Hz), 141.7, 128.6, 128.1, 126.1, 106.5 (dd, *J* = 52.6 Hz, *J* = 15.3 Hz), 81.0 (t, *J* = 1.5 Hz), 23.2. ^{19}**F NMR** (564 MHz, $CDCl_3$) δ -100.47 (dd, *J* = 78.5 Hz, *J* = 16.3 Hz), -120.48 (dd, *J* = 78.5 Hz, *J* = 3.0 Hz). **IR** (KBr): 2960, 2890, 1643, 1310, 1254, 1171 cm^{-1}. **HRMS** (ESI) m/z calculated for $C_{10}H_{10}F_2NaO$ [M+Na]$^+$ 207.0597, found 207.0608.

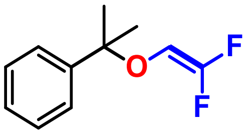

(2-((2,2-difluorovinyl)oxy)propan-2-yl)benzene (49) Colorless oil; ^{1}**H-NMR** (600 MHz, $CDCl_3$) δ 7.33-7.31 (m, 2H), 7.28 (t, *J* = 7.8 Hz, 2H), 7.21 (t, *J* = 7.4 Hz, 1H), 5.29 (dd, *J* = 16.6 Hz, *J* = 2.8 Hz, 1H), 1.54 (s, 6H). ^{13}**C-NMR** (150 MHz, $CDCl_3$) δ 155.8 (dd, *J* = 287.2 Hz, *J* = 276.7 Hz), 144.3, 128.4, 127.6, 125.6, 102.8 (dd, *J* = 53.1 Hz, *J* = 14.8 Hz), 80.7, 28.1. ^{19}**F NMR** (564 MHz, $CDCl_3$) δ -100.84 (dd, *J* = 77.2 Hz, *J* = 16.5 Hz), -119.06 (dd, *J* = 77.2 Hz, *J* = 2.8 Hz). **IR** (KBr): 2964, 2890, 1650, 1450, 1317, 1173 cm^{-1}. **HRMS** (ESI) m/z calculated for $C_{11}H_{12}F_2NaO$ [M+Na]$^+$ 221.0754, found 221.0762.

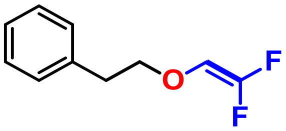

(2-((2,2-difluorovinyl)oxy)ethyl)benzene (50) Colorless oil; ^{1}**H-NMR** (600 MHz, $CDCl_3$) δ 7.30 (t, *J* = 7.2 Hz, 2H), 7.23-7.20 (m, 3H), 5.62 (dd, *J* = 16.2 Hz, *J* = 2.4 Hz, 1H), 3.89 (t, *J* = 7.2 Hz, 2H), 2.94 (t, *J* = 7.2 Hz, 2H). ^{13}**C-NMR** (150 MHz, $CDCl_3$) δ 155.4 (dd, *J* = 286.5 Hz, *J* = 274.0 Hz), 137.7, 128.9, 128.5, 126.6, 108.0 (dd, *J* = 52.2 Hz, *J* = 15.0 Hz), 74.2, 35.9. ^{19}**F NMR** (564 MHz, $CDCl_3$) δ -100.54 (dd, *J* = 79.5 Hz, *J* = 15.8 Hz), -(120.97-121.11) (m). **IR** (KBr): 2924, 2855, 1674, 1636, 1310, 1244, 1173 cm^{-1}. **MS** (EI, m/z): 184 (M$^+$, 0.20), 105 (100.00). **HRMS** (EI): exact mass calcd for $C_{10}H_{10}F_2O$ (M$^+$): 184.0700, found: 184.0705.

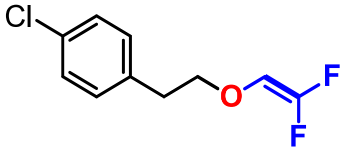

1-chloro-4-(2-((2,2-difluorovinyl)oxy)ethyl)benzene (51) Colorless oil; ^{1}**H-NMR** (600 MHz, $CDCl_3$) δ 7.21-7.18 (m, 2H), 7.07 (d, *J* = 8.5 Hz, 2H), 5.55 (dd, *J* = 16.4 Hz, *J* = 3.0 Hz, 1H), 3.80 (t, *J* = 6.8 Hz, 2H), 2.83 (t, *J* = 6.8 Hz, 2H). ^{13}**C-NMR** (150 MHz, $CDCl_3$) δ 155.4 (dd, *J* = 289.2

Hz, J = 276.7 Hz) , 136.3, 132.4, 130.2, 128.6, 108.0 (dd, J = 53.0 Hz, J = 15.2 Hz), 73.8, 35.2. **^{19}F NMR** (564 MHz, $CDCl_3$) δ -100.23 (dd, J = 79.2 Hz, J = 16.4 Hz), -120.68 (dd, J = 79.2 Hz, J = 3.0 Hz). **IR** (KBr): 2925, 2855, 1625, 1342, 1310, 1243, 1173, 816 cm^{-1}. **HRMS** (EI): exact mass calcd for $C_{10}H_9ClF_2O$ (M^+): 218.0310, found: 218.0315.

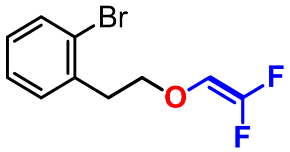

1-bromo-2-(2-((2,2-difluorovinyl)oxy)ethyl)benzene (52) Colorless oil; **^{1}H-NMR** (600 MHz, $CDCl_3$) δ 7.54 (d, J = 7.8 Hz, 1H), 7.26-7.23 (m, 2H), 7.10-7.08 (m, 1H), 5.65 (dd, J = 16.2 Hz, J = 1.8 Hz, 1H), 3.91 (t, J = 7.2 Hz, 2H), 3.09 (t, J = 7.2 Hz, 2H). **^{13}C-NMR** (150 MHz, $CDCl_3$) δ 155.3 (dd, J = 286.4 Hz, J = 273.9 Hz), 136.9, 132.9, 131.3, 128.4, 127.5, 124.5, 108.0 (dd, J = 52.4 Hz, J = 15.2 Hz), 72.2, 36.1. **^{19}F NMR** (564 MHz, $CDCl_3$) δ -100.42 (dd, J = 79.52 Hz, J = 15.8 Hz), -120.89 (dd, J = 79.52 Hz, J = 2.3 Hz). **IR** (KBr): 2924, 2855, 1647, 1625, 1341, 1310, 1243, 1174, 1034, 750 cm^{-1}. **MS** (EI, m/z): 262 (M^+, 1.04), 183 (80.99), 104 (100.00). **HRMS** (EI): exact mass calcd for $C_{10}H_9BrF_2O$ (M^+): 261.9805, found: 261.9812.

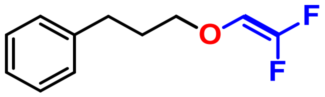

(3-((2,2-difluorovinyl)oxy)propyl)benzene (53) Colorless oil; **^{1}H-NMR** (600 MHz, $CDCl_3$) δ 7.28 (t, J = 7.2 Hz, 2H), 7.21-7.17 (m, 3H), 5.60 (dd, J = 16.2 Hz, J = 2.4 Hz, 1H), 3.67 (t, J = 6.0 Hz, 2H), 3.09 (t, J = 7.8 Hz, 2H), 1.96-1.92 (m, 2H). **^{13}C-NMR** (150 MHz, $CDCl_3$) δ 155.5 (dd, J = 286.2 Hz, J = 273.8 Hz), 141.3, 128.5, 128.5, 126.0, 108.1 (dd, J = 51.9 Hz, J = 15.0 Hz), 72.6, 31.8, 30.9. **^{19}F NMR** (564 MHz, $CDCl_3$) δ -101.04 (dd, J = 80.09 Hz, J = 16.4 Hz), -(121.18-121.32) (m). **IR** (KBr): 2924, 2855, 1635, 1456, 1310, 1273, 1241, 1171 cm^{-1}. **MS** (EI, m/z): 198 (M^+, 3.83), 119 (14.79), 91 (100.00). **HRMS** (EI): exact mass calcd for $C_{11}H_{12}F_2O$ (M^+): 198.0856, found: 198.0855.

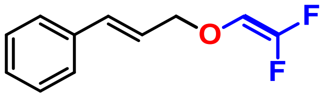

(E)-(3-((2,2-difluorovinyl)oxy)prop-1-en-1-yl)benzene (54) Colorless oil; **^{1}H-NMR** (600 MHz, $CDCl_3$) δ 7.31 (d, J = 7.3 Hz, 2H), 7.26-7.23 (m, 2H), 7.20-7.17 (m, 1H), 6.55 (d, J = 15.7 Hz, 1H), 6.18 (td, J = 15.7 Hz, J = 6.3 Hz, 1H), 5.63 (dd, J = 16.2 Hz, J = 2.8 Hz, 1H), 4.28 (d, J = 6.3

Hz, 2H). **^{13}C-NMR** (150 MHz, $CDCl_3$) δ 155.4 (dd, J = 288.6 Hz, J = 275.1 Hz), 136.1, 134.3, 128.6, 128.2, 126.6, 123.8, 107.4 (dd, J = 52.9 Hz, J = 15.4 Hz), 73.7 (t, J = 1.5 Hz). **^{19}F NMR** (564 MHz, $CDCl_3$) δ -100.19 (dd, J = 79.2 Hz, J = 16.2 Hz), -120.53 (dd, J = 79.2 Hz, J = 2.8 Hz). **IR** (KBr): 2925, 2854, 1666, 1645, 1313, 1274, 1218, 1171 cm^{-1}. **MS** (EI, m/z): 196 (M^+, 2.34), 117 (100.00). **HRMS** (EI): exact mass calcd for $C_{11}H_{10}F_2O$ (M^+): 196.0700, found: 196.0704.

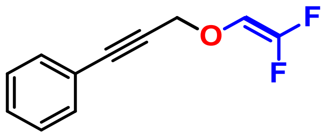

(3-((2,2-difluorovinyl)oxy)prop-1-yn-1-yl)benzene (55) Colorless oil; **^{1}H-NMR** (600 MHz, $CDCl_3$) δ 7.46-7.44 (m, 2H), 7.35-7.30 (m, 3H), 5.88 (dd, J = 16.0 Hz, J = 3.0 Hz, 1H), 4.57 (s , 2H). **^{13}C-NMR** (150 MHz, $CDCl_3$) δ 155.8 (dd, J = 287.9 Hz, J = 275.1 Hz), 131.8, 128.9, 128.4, 122.0, 106.8 (dd, J = 53.7 Hz, J = 15.5 Hz), 88.0, 82.9, 61.2 (t, J = 2.0 Hz). **^{19}F NMR** (564 MHz, $CDCl_3$) δ -98.70 (dd, J = 75.2 Hz, J = 16.0 Hz), -118.65 (dd, J = 75.2 Hz, J = 3.0 Hz). **IR** (KBr): 2927, 2853, 1642, 1244, 1170, 694 cm^{-1}. **HRMS** (EI): exact mass calcd for $C_{11}H_8F_2O$ (M^+): 194.0543, found: 194.0546.

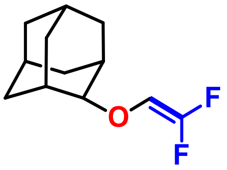

2-((2,2-difluorovinyl)oxy)adamantane (56) Colorless oil; **^{1}H-NMR** (600 MHz, $CDCl_3$) δ 5.55 (dd, J = 16.3 Hz, J = 3.0 Hz, 1H), 3.61 (t, J = 3.5 Hz 1H), 2.01-1.95 (m, 4H), 1.81-1.78 (m, 2H), 1.77-1.71 (m, 2H), 1.65 (s, 2H), 1.58 (d, J = 12.1 Hz, 2H), 1.44 (d, J = 12.1 Hz, 2H). **^{13}C-NMR** (150 MHz, $CDCl_3$) δ 155.7 (dd, J = 287.8 Hz, J = 275.2 Hz), 106.2 (dd, J = 52.1 Hz, J = 15.5 Hz), 84.9, 37.4, 36.3, 31.9, 31.1, 27.2, 27.1. **^{19}F NMR** (564 MHz, $CDCl_3$) δ -101.88 (dd, J = 81.1 Hz, J = 16.3 Hz), -121.11 (dd, J = 81.1 Hz, J = 3.0 Hz). **IR** (KBr): 3217, 3205, 3193, 2655, 1631, 1310, 1274, 1173, 810 cm^{-1}. **MS** (EI, m/z): 214 (M^+, 9.12), 135 (100.00). **HRMS** (EI): exact mass calcd for $C_{12}H_{16}F_2O$ (M^+): 214.1169, found: 214.1173.

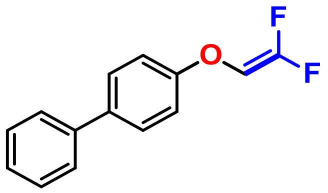

4-((2,2-difluorovinyl)oxy)-1,1'-biphenyl (57) Colorless oil; **^{1}H-NMR** (600 MHz, $CDCl_3$) δ 7.56-7.54 (m, 4H), 7.43 (t, *J* = 7.6 Hz, 2H), 7.33 (t, *J* = 7.2 Hz, 1H), 7.08 (d, *J* = 8.4 Hz, 2H), 6.11 (dd, *J* = 15.3 Hz, *J* = 3.3 Hz, 1H). **^{13}C-NMR** (150 MHz, $CDCl_3$) δ 156.7 (dd, *J* = 291.3 Hz, *J* = 279.9 Hz), 156.6, 140.3, 136.2, 128.8, 128.4, 127.1, 126.9, 115.6, 104.5 (dd, *J* = 56.1 Hz, *J* = 15.6 Hz). **^{19}F NMR** (564 MHz, $CDCl_3$) δ -97.13 (dd, *J* = 68.0 Hz, *J* = 15.3 Hz), -115.55 (dd, *J* = 68.0 Hz, *J* = 3.3 Hz). **IR** (KBr): 1715, 1632, 1337, 1148, 1046, 697 cm^{-1}. **MS** (EI, m/z): 232 (M^+, 47.23), 153 (100.00). **HRMS** (EI): exact mass calcd for $C_{14}H_{10}F_2O$ (M^+): 232.0700, found: 232.0703.

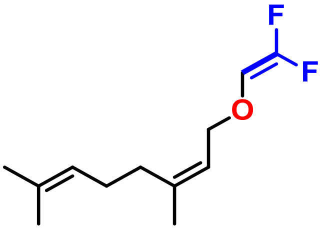

Nerol (58) Colorless oil; **^{1}H-NMR** (600 MHz, $CDCl_3$) δ 5.57 (dd, *J* = 16.2 Hz, *J* = 2.4 Hz, 1H), 5.29 (t, *J* = 7.2 Hz, 1H), 5.01 (t, *J* = 7.2 Hz, 1H), 4.13 (d, *J* = 7.2 Hz, 2H), 2.05-1.98 (m, 4H), 1.70 (s, 3H), 1.61 (s, 3H), 1.53 (s, 3H). **^{13}C-NMR** (150 MHz, $CDCl_3$) δ 155.3 (dd, *J* = 288.0 Hz, *J* = 275.3 Hz), 142.8, 132.3, 123.5, 120.0, 107.4 (dd, *J* = 52.5 Hz, *J* = 15.5 Hz), 69.2, 32.2, 26.6, 25.6, 23.4, 17.6. **^{19}F NMR** (564 MHz, $CDCl_3$) δ -101.21 (dd, *J* = 80.5 Hz, *J* = 16.2 Hz), -121.33 (dd, *J* = 80.5 Hz, *J* = 2.4 Hz). **IR** (KBr): 3329, 2966, 2923, 2867, 1717, 1670, 1631, 1447, 1377, 1084, 1001 cm^{-1}. **MS** (EI, m/z): 216 (M^+, 100.00), 201 (50.88). **HRMS** (EI): exact mass calcd for $C_{12}H_{18}F_2O$ (M^+): 216.1326, found: 216.1329.

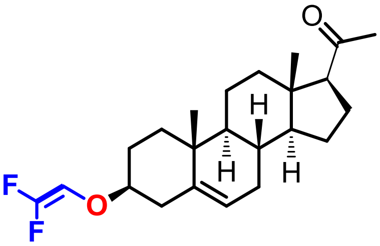

Pregnenolone (59) Colorless oil; **^{1}H-NMR** (600 MHz, $CDCl_3$) δ 5.65 (dd, *J* = 16.2 Hz, *J* = 3.0 Hz, 1H), 5.39-5.37 (m, 1H), 3.44-3.38 (m, 1H), 2.53 (t, *J* = 9.0 Hz, 1H), 2.41-2.37 (m, 1H), 2.34-2.28 (m, 1H), 2.23-2.15 (m, 1H), 2.13 (s, 3H), 2.07-1.99 (m, 2H), 1.96-1.88 (m, 2H), 1.72-1.63 (m, 2H), 1.63-1.53 (m, 3H), 1.53-1.42 (m, 3H), 1.27-1.12 (m, 2H), 1.07 (td, *J* = 13.8 Hz, *J* = 3.6 Hz ,1H), 1.02-0.96 (m, 4H), 0.64 (s, 3H). **^{13}C-NMR** (150 MHz, $CDCl_3$) δ 209.4, 155.6 (dd, *J* = 288.2 Hz, *J* = 275.5 Hz), 139.9, 122.2, 106.1 (dd, *J* = 52.6 Hz, *J* = 15.2 Hz), 81.9, 63.7, 56.9, 49.9, 44.0, 38.8,

38.6, 36.9, 36.7, 31.8, 31.8, 31.5, 28.1, 24.5, 22.8, 21.1, 19.3, 13.2. **^{19}F NMR** (564 MHz, $CDCl_3$) δ -101.21 (dd, J = 80.0 Hz, J = 16.2 Hz), -120.63 (dd, J = 80.0 Hz, J = 3.0 Hz). **IR** (KBr): 3607, 2968, 2932, 2895, 1700, 1683, 1631, 1360, 1058 cm^{-1}. **HRMS** (ESI) m/z calculated for $C_{23}H_{32}F_2NaO_2$ $[M+Na]^+$ 401.2263, found 401.2274.

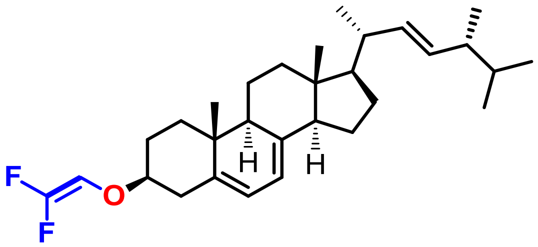

Ergosterol (60) Colorless oil; **^{1}H-NMR** (600 MHz, $CDCl_3$) δ 5.66 (dd, J = 16.2 Hz, J = 2.4 Hz, 1H), 5.59-5.58 (m, 1H), 5.39-5.38 (m, 1H), 5.25-5.15 (m, 2H), 3.54-3.49 (m, 1H), 2.56-2.53 (m, 1H), 2.36 (t, J = 12.6 Hz, 1H), 2.08-2.01 (m, 2H), 1.99-1.95 (m, 2H), 1.94-1.83 (m, 3H), 1.79-1.64 (m, 3H), 1.60-1.53 (m, 2H), 1.50-1.45 (m, 1H), 1.41-1.31 (m, 2H), 1.29-1.24 (m, 3H), 1.04 (d, J = 6.6 Hz, 3H), 0.95 (s, 3H), 0.92 (d, J = 7.2 Hz, 3H), 0.84 (d, J = 6.6 Hz, 3H), 0.83 (d, J = 6.6 Hz, 3H), 0.63 (s, 3H). **^{13}C-NMR** (150 MHz, $CDCl_3$) δ 155.6 (dd, J = 287.8 Hz, J = 277.0 Hz), 139.9, 138.3, 129.3, 122.5, 106.2 (dd, J = 52.2 Hz, J = 15.4 Hz), 82.1, 56.9, 56.0, 51.3, 50.1, 42.2, 40.5, 39.7, 38.7, 37.0, 36.7, 31.9, 31.9, 31.9, 28.9, 28.1, 25.4, 24.4, 21.2, 21.1, 19.3, 19.0, 12.2, 12.0. **^{19}F NMR** (564 MHz, $CDCl_3$) δ -101.30 (dd, J = 79.5 Hz, J = 16.2 Hz), -120.76 (dd, J = 79.5 Hz, J = 2.4 Hz). **IR** (KBr): 3430, 2956, 2871, 1651, 1643, 1460, 1377, 1054 cm^{-1}. **HRMS** (ESI) m/z calculated for $C_{30}H_{44}F_2NaO$ $[M+Na]^+$ 481.3258, found 481.3259.

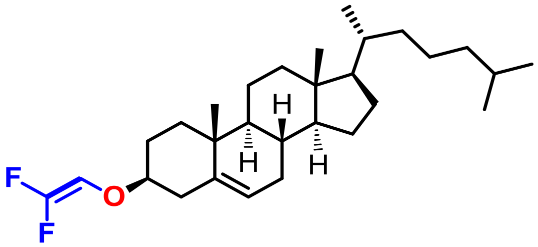

Cholesterol (61) Colorless oil; **^{1}H-NMR** (600 MHz, $CDCl_3$) δ 5.64 (dd, J = 16.2 Hz, J = 3.0 Hz, 1H), 5.38-5.36 (m, 1H), 3.43-3.37 (m, 1H), 2.39-2.35 (m, 1H), 2.34-2.28 (m, 1H), 2.03-1.95 (m, 2H), 1.94-1.80 (m, 3H), 1.61-1.42 (m, 7H), 1.39-1.30 (m, 3H), 1.30-1.19 (m, 1H), 1.18-1.04 (m, 7H), 1.01-0.95 (m, 5H), 0.95-0.89 (m, 4H), 0.87 (d, J = 6.6 Hz, 3H), 0.86 (d, J = 6.6 Hz, 3H), 0.68 (s, 3H). **^{13}C-NMR** (150 MHz, $CDCl_3$) δ 155.6 (dd, J = 288.0 Hz, J = 275.8 Hz), 139.9, 122.5, 106.2 (dd, J = 52.5 Hz, J = 15.8 Hz), 82.1, 56.8, 56.2, 50.1, 42.3, 39.8, 39.5, 38.7, 37.0, 36.7, 36.2, 35.8, 31.9, 31.9, 28.2, 28.1, 28.0, 24.3, 23.9, 22.8, 22.6, 21.1, 19.3, 18.7, 11.9. **^{19}F NMR** (564

MHz, $CDCl_3$) δ -101.32 (dd, *J* = 80.0 Hz, *J* = 16.2 Hz), -120.76 (dd, *J* = 80.0 Hz, *J* = 3.0 Hz). **IR** (KBr): 2877, 1650, 1647, 1464, 1371, 1053 cm^{-1}. **HRMS** (ESI) m/z calculated for $C_{29}H_{46}F_2NaO[M+Na]^+$ 471.3414, found 471.3419.

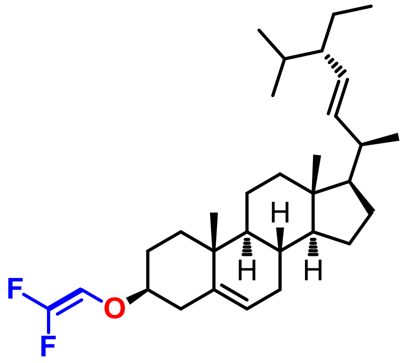

Stigmasterol (62) Colorless oil; **^{1}H-NMR** (600 MHz, $CDCl_3$) δ 5.64 (dd, *J* = 16.2 Hz, *J* = 2.4 Hz, 1H), 5.37 (d, *J* = 4.2 Hz, 1H), 5.15 (dd, *J* = 15.0 Hz, *J* = 8.4 Hz, 1H), 5.02 (dd, *J* = 15.0 Hz, *J* = 8.4 Hz, 1H), 3.43-3.37 (m, 1H), 2.38-2.25 (m, 2H), 2.07-1.94 (m, 3H), 1.94-1.83 (m, 2H), 1.74-1.68 (m, 1H), 1.61-1.39 (m, 9H), 1.29-1.22 (m, 2H), 1.21-1.11 (m, 3H), 1.10-1.00 (m, 8H), 0.99-0.91 (m, 1H), 0.85 (d, *J* = 8.4 Hz, 3H), 0.83-0.79 (m, 6H), 0.70 (s, 3H). **^{13}C-NMR** (150 MHz, $CDCl_3$) δ 155.6 (dd, *J* = 287.8 Hz, *J* = 277.0 Hz), 139.9, 138.3, 129.3, 122.5, 106.2 (dd, *J* = 52.2 Hz, *J* = 15.4 Hz), 82.1, 56.9, 56.0, 51.3, 50.1, 42.2, 40.5, 39.7, 38.7, 37.0, 36.7, 31.9, 31.89, 31.87, 28.9, 28.1, 25.4, 24.4, 21.2, 21.1, 19.3, 19.0, 12.2, 12.0. **^{19}F NMR** (564 MHz, $CDCl_3$) δ -101.30 (dd, *J* = 79.5 Hz, *J* = 16.2 Hz), -120.76 (dd, *J* = 79.5 Hz, *J* = 2.4 Hz). **IR** (KBr): 2956, 2936, 2903, 2866, 1644, 1637, 1461, 1055, 971 cm^{-1}. **HRMS** (ESI) m/z calculated for $C_{31}H_{48}F_2NaO$ $[M+Na]^+$ 497.3571, found 497.3578.

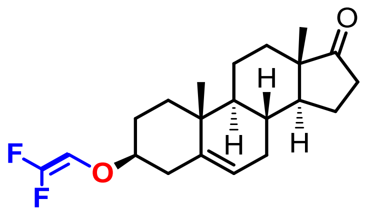

Prasterone (63) Colorless oil; **^{1}H-NMR** (600 MHz, $CDCl_3$) δ 5.65 (dd, *J* = 16.2 Hz, *J* = 3.0 Hz, 1H), 5.42-5.40 (m, 1H), 3.45-3.39 (m, 1H), 2.47 (dd, *J* = 19.2 Hz, *J* = 8.4 Hz, 1H), 2.42-2.39 (m, 1H), 2.35-2.30 (m, 1H), 2.15-2.05 (m, 2H), 1.98-1.83 (m, 4H), 1.71-1.61 (m, 3H), 1.61-1.46 (m, 3H), 1.31-1.26 (m, 2H), 1.09-0.98 (m, 5H), 0.89 (s, 3H). **^{13}C-NMR** (150 MHz, $CDCl_3$) δ 221.0, 155.6 (dd, *J* = 287.8 Hz, *J* = 276.0 Hz), 140.1, 121.7, 106.1 (dd, *J* = 52.4 Hz, *J* = 15.7 Hz), 81.8, 51.7, 50.2, 47.5, 38.7, 36.9, 36.8, 35.8, 31.5, 31.4, 30.8, 28.0, 21.9, 20.4, 19.3, 13.5. **^{19}F NMR**

(564 MHz, $CDCl_3$) δ -101.15 (dd, *J* = 79.5 Hz, *J* = 16.2 Hz), -120.53 (dd, *J* = 79.5 Hz, *J* = 2.2 Hz). **IR** (KBr): 2931, 2894, 2850, 1737, 1641, 1631, 1451, 1059, 1029 cm^{-1}. **HRMS** (ESI) m/z calculated for $C_{21}H_{28}F_2NaO_2$ $[M+Na]^+$ 373.1950, found 373.1941.

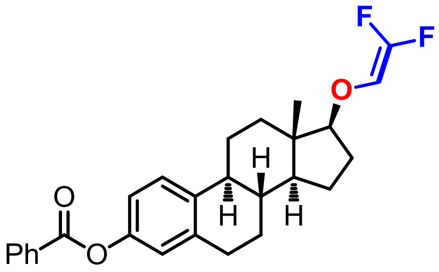

Estradiol benzoate (64) Write solid, mp: 118-119 ℃; **^{1}H-NMR** (600 MHz, $CDCl_3$) δ 8.20-8.18 (m, 2H), 7.62 (t, *J* = 7.8 Hz, 1H), 7.50 (t, *J* = 7.8 Hz, 2H), 7.32 (d, *J* = 8.4 Hz, 1H), 6.97 (dd, *J* = 8.4 Hz, *J* = 2.4 Hz, 1H), 6.92 (d, *J* = 2.4 Hz, 1H), 5.68 (dd, *J* = 16.2 Hz, *J* = 3.0 Hz, 1H), 3.63 (d, *J* = 8.4 Hz, 1H), 2.94-2.85 (m, 2H), 2.34-2.29 (m, 1H), 2.29-2.22 (m, 1H), 2.13-2.04 (m, 2H), 1.91-1.87 (m, 1H), 1.74-1.65 (m, 2H), 1.59-1.45 (m, 2H), 1.45-1.32 (m, 3H), 1.27-1.19 (m, 1H), 0.85 (s, 3H). **^{13}C-NMR** (150 MHz, $CDCl_3$) δ 165.4, 154.9 (dd, *J* = 286.8 Hz, *J* = 275.0 Hz), 148.7, 138.2, 137.8, 133.5, 130.1, 129.7, 128.5, 126.4, 121.6, 118.7, 108.1 (dd, *J* = 52.2 Hz, *J* = 15.1 Hz), 91.7, 50.1, 44.0, 43.4, 38.2, 37.5, 29.5, 27.7, 27.0, 26.1, 23.0, 11.4. **^{19}F NMR** (564 MHz, $CDCl_3$) δ -101.55 (dd, *J* = 81.7 Hz, *J* = 16.2 Hz), -122.13 (dd, *J* = 81.7 Hz, *J* = 3.0 Hz). **IR** (KBr): 3667, 2950, 1730, 1647, 1631, 1497, 1266, 1223, 1026 cm^{-1}. **HRMS** (ESI) m/z calculated for $C_{27}H_{28}F_2NaO_3$ $[M+Na]^+$ 461.1899, found 461.1901.

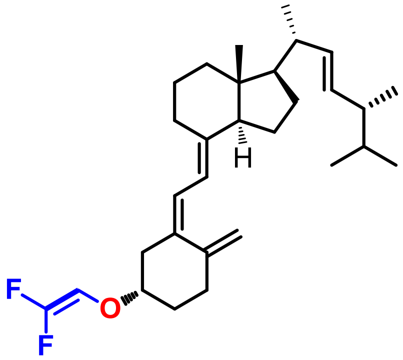

Vitamin D_2 (65) Colorless oil; **^{1}H-NMR** (600 MHz, $CDCl_3$) δ 6.23 (d, *J* = 11.4 Hz, 1H), 6.02 (d, *J* = 11.4 Hz, 1H), 5.65 (dd, *J* = 16.2 Hz, *J* = 3.0 Hz, 1H), 5.24-5.15 (m, 2H), 5.05 (s, 1H), 4.83 (d, *J* = 1.8 Hz, 1H), 3.77-3.72 (m, 1H), 2.83-2.80 (m, 1H), 2.61-2.57 (m, 1H), 2.45-2.36 (m, 2H), 2.17-2.11 (m, 1H), 2.05-1.95 (m, 4H), 1.87-1.83 (m, 2H), 1.76-1.64 (m, 4H), 1.55-1.43 (m, 3H), 1.35-1.26 (m, 3H), 1.02 (d, *J* = 7.0 Hz, 3H), 0.91 (d, *J* = 7.0 Hz, 3H), 0.84 (d, *J* = 6.6 Hz, 3H),

0.82 (d, J = 6.6 Hz, 3H), 0.55 (s, 3H). **^{13}C-NMR** (150 MHz, $CDCl_3$) δ 155.8 (dd, J = 288.2 Hz, J = 276.5 Hz), 144.5, 142.4, 135.6, 134.2, 132.0, 122.4, 117.5, 112.81, 106.1 (dd, J = 52.8 Hz, J = 15.4 Hz), 79.8, 56.4, 56.4, 45.8, 42.8, 42.5, 40.4, 40.4, 33.1, 32.1, 31.9, 29.0, 27.8, 23.5, 22.2, 21.1, 19.9, 19.6, 17.6, 12.3. **^{19}F-NMR** (564 MHz, $CDCl_3$) δ -100.42 (dd, J = 78.0 Hz, J = 16.2 Hz), -120.10 (dd, J = 78.0 Hz, J = 3.0 Hz). **IR** (KBr): 3286, 2955, 2933, 2872,1650, 1644, 1639, 1442, 1068 cm^{-1}. **HRMS** (ESI) m/z calculated for $C_{30}H_{44}F_2NaO$ $[M^+Na]^+$ 481.3252, found 481.3266.

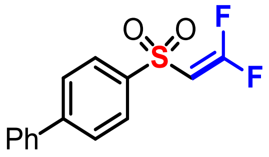

4-((2,2-difluorovinyl)sulfonyl)-1,1'-biphenyl (9') Write solid, mp: 108-109 ℃; **^{1}H-NMR** (600 MHz, $CDCl_3$) δ 8.01 (d, J = 8.4 Hz, 2H), 7.78 (d, J = 8.4 Hz, 2H), 7.61 (d, J = 7.2 Hz, 2H), 7.50 (t, J = 7.2 Hz, 2H), 7.44 (t, J = 7.2 Hz, 1H), 5.69 (dd, J = 20.2 Hz, J = 2.2 Hz, 1H). **^{13}C-NMR** (150 MHz, $CDCl_3$) δ 159.1 (t, J = 307.2 Hz), 147.1, 139.8 (d, J = 3.0 Hz), 139.0, 129.1, 128.8, 128.1, 127.9, 127.4, 90.6 (dd, J = 24.5 Hz, J = 13.8 Hz). **^{19}F-NMR** (564 MHz, $CDCl_3$) δ -62.66 (dd, J = 20.2 Hz, J = 11.8 Hz), -68.19 (dd, J = 11.8 Hz, J = 1.8 Hz). **IR** (KBr): 3084, 3031, 1769, 1712, 1589, 1396, 1328, 1145, 1087, 977, 844 cm^{-1}. **HRMS** (ESI) m/z calculated for $C_{14}H_{10}F_2NaO_2S$ $[M^+Na]^+$ 303.0267, found 303.0261.

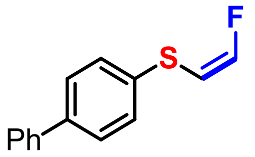

(Z)-[1,1'-biphenyl]-4-yl(2-fluorovinyl)sulfane (66) Colorless oil; **^{1}H-NMR** (600 MHz, $CDCl_3$) δ 7.57-7.53 (m, 4H), 7.45-7.42 (m, 4H), 7.35 (t, J = 7.2 Hz, 1H), 6.84 (dd, J = 81.6 Hz, J = 4.2 Hz, 1H), 5.70 (dd, J = 38.4 Hz, J = 4.2 Hz, 1H). **^{13}C-NMR** (150 MHz, $CDCl_3$) δ 148.9 (d, J = 262.6 Hz), 140.2, 139.9, 133.7, 129.6, 128.9, 127.8, 127.5, 126.9, 105.8 (d, J = 7.8 Hz). **^{19}F-NMR** (564 MHz, $CDCl_3$) δ -118.96 (dd, J = 81.6 Hz, J = 38.4 Hz). **IR** (KBr): 1714, 1481, 1314, 1172, 1131, 1061, 824, 756, 690 cm^{-1}. **MS** (EI, m/z): 230 (M^+, 100.00). **HRMS** (EI): exact mass calcd for $C_{14}H_{11}FS$ (M^+): 230.0565, found: 230.0567.

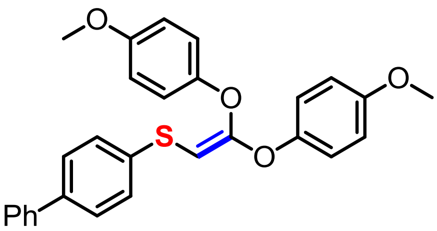

[1,1'-biphenyl]-4-yl(2,2-bis(4-methoxyphenoxy)vinyl)sulfane (67) Colorless oil; **^{1}H-NMR** (600 MHz, $CDCl_3$) δ 7.56-7.54 (m, 2H), 7.51-7.49 (m, 2H), 7.42 (t, *J* = 7.2 Hz, 2H), 7.36-7.31 (m, 3H), 7.13 (d, *J* = 8.4 Hz, 2H), 7.09 (d, *J* = 8.4 Hz, 2H), 7.01-6.98 (m, 4H), 5.13 (s, 1H), 2.31 (s, 3H), 2.29 (s, 3H). **^{13}C-NMR** (150 MHz, $CDCl_3$) δ 158.6, 152.4, 151.9, 140.5, 138.4, 136.8, 134.4, 133.4, 130.2, 130.0, 128.8, 127.5, 127.3, 127.2, 126.8, 119.0, 117.4, 83.8, 20.7, 20.7. **IR** (KBr): 3063, 2245, 1650, 1148, 1046, 855, 835 cm^{-1}. **HRMS** (ESI) m/z calculated for $C_{28}H_{24}O_4NaS$ $[M+Na]^+$479.1293, found 479.1298.

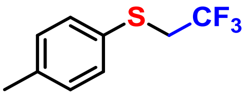

p-tolyl(2,2,2-trifluoroethyl)sulfane (2') Colorless oil; **^{1}H-NMR** (600 MHz, $CDCl_3$) δ 7.40 (d, *J* = 7.8 Hz, 2H), 7.14 (d, *J* = 7.8 Hz 2H), 3.38 (q, *J* = 9.8 Hz, 2H), 2.34 (s, 3H). **^{13}C-NMR** (150 MHz, $CDCl_3$) δ 138.5, 132.5, 130.1, 130.0, 125.4 (q, *J* = 277.1 Hz), 38.7 (q, *J* = 32.3 Hz), 21.1. **^{19}F-NMR** (564 MHz, $CDCl_3$) δ -66.36 (t, *J* = 9.3 Hz). **IR** (KBr): 2925, 1652, 1384, 1307, 1243, 1124, 1081, 806 cm^{-1}. **HRMS** (ESI) m/z calculated for $C_9H_9F_3NaS$ $[M+Na]^+$ 229.0275, found 229.0279.

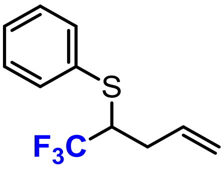

Phenyl(1,1,1-trifluoropent-4-en-2-yl)sulfane (69) Colourless oil; **^{1}H-NMR** (600 MHz, $CDCl_3$) δ 7.53-7.49 (m, 2H), 7.34-7.30 (m, 3H), 5.99-5.91 (m, 1H), 5.24-5.21 (m, 1H), 5.21-5.19 (m, 1H), 3.43-3.34 (m, 1H), 2.68-2.63 (m, 1H), 2.44-2.36 (m, 1H); **^{13}C-NMR** (150 MHz, $CDCl_3$) δ 133.6, 133.1, 132.9, 129.1, 128.4, 126.5 (q, *J* = 277.7 Hz), 118.7, 52.5 (q, *J* = 28.2 Hz), 33.0 (q, *J* = 1.8 Hz); **^{19}F-NMR** (564 MHz, $CDCl_3$) δ -70.06 (d, *J* = 8.5 Hz). **IR** (KBr): 3080, 2957, 2923, 2850, 1650, 1253, 1165, 1103, 920, 800 cm^{-1}. **MS** (EI, m/z): 232 (M^+, 30.32), 191 (100.00). **HRMS** (EI): exact mass calcd for $C_{11}H_{11}F_3S$ (M^+): 232.0534, found: 232.0532.

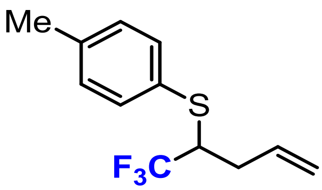

P-tolyl(1,1,1-trifluoropent-4-en-2-yl)sulfane (70) Colourless oil; **^{1}H-NMR** (600 MHz, $CDCl_3$) δ 7.41 (d, *J* = 7.8 Hz, 2H), 7.12 (d, *J* = 7.8 Hz, 2H), 5.99-5.90 (m, 1H), 5.21 (dq, *J* = 7.8, 1.8 Hz, 1H), 5.19 (t, *J* = 1.2 Hz, 1H), 3.34-3.27 (m, 1H), 2.67-2.60 (m, 1H), 2.41-2.34 (m, 1H), 2.33 (s, 3H); **^{13}C-NMR** (150 MHz, $CDCl_3$) δ 138.8, 134.2, 133.3, 129.9, 129.2, 126.5 (q, *J* = 277.7 Hz) , 118.5, 52.7 (q, *J* = 27.8 Hz), 32.9 (q, *J* = 1.8 Hz), 21.1; **^{19}F-NMR** (564 MHz, $CDCl_3$) δ -69.51 (d, *J* = 8.5 Hz). **IR** (KBr): 3082, 2924, 1650, 1252, 1163, 1105, 829 cm^{-1}. **MS** (EI, m/z): 246 (M^+, 94.14), 205 (100.00), 185 (35.24). **HRMS** (EI): exact mass calcd for $C_{12}H_{13}F_3S$ (M^+): 246.0690, found: 246.0693.

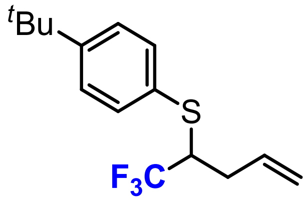

(4-(tert-butyl)phenyl)(1,1,1-trifluoropent-4-en-2-yl)sulfane (71) Colourless oil; **^{1}H-NMR** (600 MHz, $CDCl_3$) δ 7.35 (d, *J* = 8.4 Hz, 2H), 7.24 (d, *J* = 8.4 Hz, 2H), 5.92-5.85 (m, 1H), 5.14-5.11 (m, 1H), 5.11-5.09 (m, 1H), 3.27-3.21 (m, 1H), 2.58-2.52 (m, 1H), 2.34-2.27 (m,, 1H), 1.21 (s, 9H); **^{13}C-NMR** (150 MHz, $CDCl_3$) δ 151.9, 133.7, 133.3, 129.4, 126.6 (q, *J* = 277.5 Hz), 126.2, 118.5, 52.7 (q, *J* = 28.5 Hz), 34.6, 33.1 (q, *J* = 1.5 Hz), 31.2; **^{19}F-NMR** (564 MHz, $CDCl_3$) δ -69.98 (d, *J* = 8.5 Hz). **IR** (KBr): 3081, 2964, 1645, 1490, 1348, 1252, 1164, 1103, 919, 829 cm^{-1}. **MS** (EI, m/z): 288 (M^+, 30.31), 273 (100.00). **HRMS** (EI): exact mass calcd for $C_{15}H_{19}F_3S$ (M^+): 288.1160, found: 288.1170.

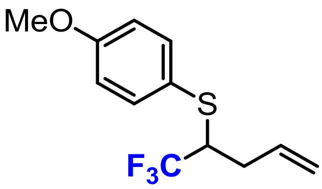

(4-methoxyphenyl)(1,1,1-trifluoropent-4-en-2-yl)sulfane (72) Colourless oil; **^{1}H-NMR** (600 MHz, $CDCl_3$) δ 7.35 (d, *J* = 8.4 Hz, 2H), 6.71 (d, *J* = 8.4 Hz, 2H), 5.87-5.79 (m, 1H), 5.11-5.08 (m, 1H), 5.08-5.05 (m, 1H), 3.64 (s, 3H), 3.13-3.07 (m, 1H), 2.52-2.46 (m, 1H), 2.26-2.19 (m,

1H); **^{13}C-NMR** (150 MHz, $CDCl_3$) δ 160.4, 136.7, 133.3, 126.6 (q, *J* = 277.8 Hz), 122.8, 118.4, 114.6, 55.1, 52.5 (q, *J* = 27.5 Hz), 32.7 (q, *J* = 1.5 Hz); **^{19}F-NMR** (564 MHz, $CDCl_3$) δ -70.06 (d, *J* = 8.5 Hz). **IR** (KBr): 3082, 2944, 1645, 1594, 1494, 1288, 1248, 1167, 1101, 918, 829 cm^{-1}. **MS** (EI, m/z): 262 (M^+, 100), 221 (9.33), 165 (85.62). **HRMS** (EI): exact mass calcd for $C_{12}H_{13}F_3OS$ (M^+): 262.0639, found: 262.0638.

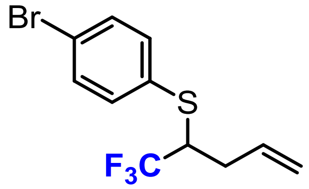

(4-bromophenyl)(1,1,1-trifluoropent-4-en-2-yl)sulfane (73) Colourless oil; **^{1}H-NMR** (600 MHz, $CDCl_3$) δ 7.43-7.39 (m, 2H), 7.37-7.34 (m, 2H), 5.95-5.86 (m, 1H), 5.23-5.20 (m, 1H), 5.20-5.18 (m, 1H), 3.38-3.29 (m, 1H), 2.68-2.62 (m, 1H), 2.40-2.33 (m, 1H); **^{13}C-NMR** (150 MHz, $CDCl_3$) δ 135.2, 132.8, 132.2, 132.0, 126.3 (q, *J* = 277.8 Hz), 123.0, 118.9, 52.5 (q, *J* = 28.2 Hz), 32.8 (q, *J* = 1.8 Hz); **^{19}F-NMR** (564 MHz, CDCl3) δ -70.00 (d, *J* = 8.5 Hz). **IR** (KBr): 3083, 1645, 1593, 1244, 1169, 1101, 827 cm^{-1}. **MS** (EI, m/z): 310 (M^+, 28.6), 271 (15.75), 190 (100.00). **HRMS** (EI): exact mass calcd for $C_{11}H_{10}BrF_3S$ (M^+): 309.9639, found: 309.9631

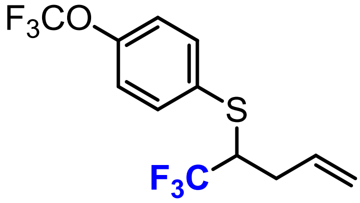

(4-(trifluoromethoxy)phenyl)(1,1,1-trifluoropent-4-en-2-yl)sulfane (74) Colourless oil; **^{1}H-NMR** (600 MHz, $CDCl_3$) δ 7.42 (d, *J* = 8.4 Hz, 2H), 7.03 (d, *J* = 8.4 Hz, 2H), 5.86-5.77 (m, 1H), 5.11 (d, *J* = 4.2 Hz, 1H), 5.08 (s, 1H), 3.27-3.17 (m, 1H), 2.59-2.52 (m, 1H), 2.40-2.23 (m, 1H); **^{13}C-NMR** (150 MHz, $CDCl_3$) δ 149.6, 135.5, 132.9, 131.6, 126.5 (q, *J* = 277.5 Hz), 121.4, 120.5 (q, *J* = 250.8 Hz), 118.9, 52.8 (q, *J* = 28.2 Hz), 32.9 (q, *J* = 1.8 Hz); **^{19}F-NMR** (564 MHz, $CDCl_3$) δ -58.19, -70.33 (d, *J* = 7.9 Hz). **IR** (KBr): 3080, 3025, 2925, 1652, 1492, 1448, 1348, 1294, 1254, 1174, 1103, 810 cm^{-1}. **MS** (EI, m/z): 316 (M^+, 100.00), 275 (97.85). **HRMS** (EI): exact mass calcd for $C_{12}H_{10}F_6OS$ (M^+): 316.0357, found: 316.0353.

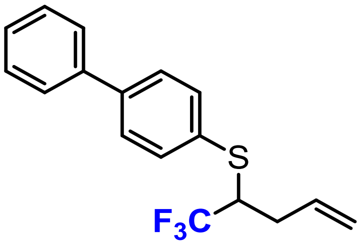

[1,1'-biphenyl]-4-yl(1,1,1-trifluoropent-4-en-2-yl)sulfane (75) White solid, m.p: 52-53 ℃; **^{1}H-NMR** (600 MHz, $CDCl_3$) δ 7.57-7.51 (m, 4H), 7.51-7.47 (m, 2H), 7.40 (t, *J* = 7.8, 2H), 7.32 (t, *J* = 7.8 Hz, 1H), 5.99-5.90 (m, 1H), 5.24-5.18 (m, 2H), 3.44-3.35 (m, 1H), 2.68-2.64 (m, 1H), 2.45-2.37 (m, 1H); **^{13}C-NMR** (150 MHz, $CDCl_3$) δ 141.4, 140.0, 134.0, 133.1, 131.8, 128.8, 127.73, 127.69, 127.0, 126.5 (q, *J* = 277.8 Hz), 118.7, 52.5 (q, *J* = 27.9 Hz), 33.0; **^{19}F-NMR** (564 MHz, $CDCl_3$) δ -69.82 (d, *J* = 7.9 Hz). **IR** (KBr): 2923, 1650, 1314, 1225, 1167, 1131, 800, 709 cm^{-1}. **HRMS** (ESI) m/z calculated for $C_{17}H_{15}F_3NaS$ $[M+Na]^+$ 331.0744, found 331.0748.

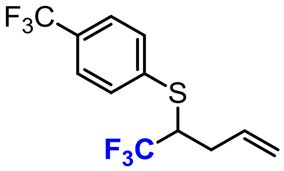

(4-(trifluoromethyl)phenyl)(1,1,1-trifluoropent-4-en-2-yl)sulfane (76) Colourless oil; **^{1}H-NMR** (600 MHz, $CDCl_3$) δ 7.56-7.52 (m, 2H), 7.16 (d, *J* = 7.8 Hz, 2H), 5.98-5.89 (m, 1H), 5.25-5.22 (m, 1H), 5.22-5.20 (m, 1H), 3.39-3.31 (m, 1H), 2.70-2.64 (m, 1H), 2.42-2.36 (m, 1H); **^{13}C-NMR** (150 MHz, $CDCl_3$) δ 149.5, 135.4, 132.9, 131.5, 126.4 (q, *J* = 277.5 Hz), 121.4, 120.4 (q, *J* = 256.2 Hz), 118.9, 52.8 (q, *J* = 28.2 Hz), 32.8 (q, *J* = 1.8 Hz); **^{19}F-NMR** (564 MHz, $CDCl_3$) δ -58.06, -70.23 (d, *J* = 8.5 Hz). **IR** (KBr): 3085, 2924, 1645, 1491, 1348, 1167, 1106, 916, 807 cm^{-1}. **HRMS** (EI): exact mass calcd for $C_{12}H_{10}F_6S$ (M^+): 300.0407, found: 300.0407.

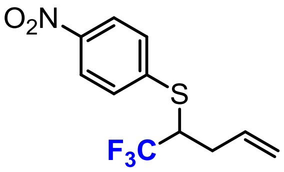

(4-nitrophenyl)(1,1,1-trifluoropent-4-en-2-yl)sulfane (77) Colourless oil; **^{1}H-NMR** (600 MHz, $CDCl_3$) δ 8.07-8.02 (m, 2H), 7.47-7.43 (m, 2H), 5.85-5.76 (m, 1H), 5.17-5.10 (m, 2H), 3.62-3.54 (m, 1H), 2.70-2.63 (m, 1H), 2.40-2.32 (m, 1H); **^{13}C-NMR** (150 MHz, $CDCl_3$) δ 146.7, 143.0, 132.1, 130.6, 126.0 (q, *J* = 277.8 Hz), 124.0, 119.4, 50.7 (q, *J* = 28.8 Hz), 32.7 (q, *J* = 1.7 Hz);

^{19}**F-NMR** (564 MHz, $CDCl_3$) δ -70.21 (d, *J* = 7.9 Hz). **IR** (KBr): 1645, 1521, 1479, 1171, 1147, 807 cm^{-1}. **MS** (EI, m/z): 277 (M^+, 50.96), 190 (100.00). **HRMS** (EI): exact mass calcd for $C_{11}H_{10}F_3NO_2S$ (M^+): 277.0384, found: 277.0381.

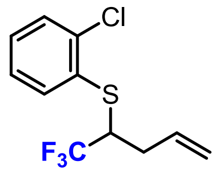

(2-chlorophenyl)(1,1,1-trifluoropent-4-en-2-yl)sulfane (78) Colourless oil; ^{1}**H-NMR** (600 MHz, $CDCl_3$) δ 7.56 (d, *J* = 7.2 Hz, 1H), 7.39 (d, *J* = 7.8 Hz, 1H), 7.23-7.16 (m, 2H), 6.01-5.92 (m, 1H), 5.21 (d, *J* = 16.8 Hz, 1H), 5.17 (d, *J* = 9.6 Hz, 1H), 3.67-3.59 (m, 1H), 2.74-2.67 (m, 1H), 2.51-2.44 (m, 1H); ^{13}**C-NMR** (150 MHz, $CDCl_3$) δ 137.3, 135.2, 132.7, 132.1, 130.1, 129.7, 127.2, 126.5 (q, *J* = 278.0 Hz), 118.7, 50.3 (q, *J* = 28.1 Hz), 33.1 (q, *J* = 1.8 Hz); ^{19}**F-NMR** (564 MHz, $CDCl_3$) δ -69.70 (d, *J* = 7.9 Hz). **IR** (KBr): 3082, 2924, 1645, 1454, 1253, 1165, 1110, 922 cm^{-1}. **MS** (EI, m/z): 266 (M^+, 70.32), 225 (100.00). **HRMS** (EI): exact mass calcd for $C_{11}H_{10}ClF_3S$ (M^+): 266.0144, found: 266.0150.

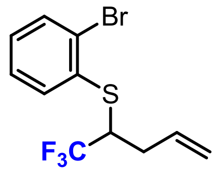

(2-bromophenyl)(1,1,1-trifluoropent-4-en-2-yl)sulfane (79) Colourless oil; ^{1}**H-NMR** (600 MHz, $CDCl_3$) δ 7.61 (dd, *J* = 7.8, 1.2 Hz, 1H), 7.58 (dd, *J* = 7.8, 1.2 Hz, 1H), 7.27 (td, *J* = 7.2, 1.2 Hz, 1H), 7.15 (td, *J* = 7.8, 1.8 Hz, 1H), 6.02-5.94 (m, 1H), 5.23 (dq, *J* = 17.4, 1.8 Hz, 1H), 5.19 (dq, *J* = 10.2, 1.2 Hz, 1H), 3.69-3.62 (m, 1H), 2.76-2.69 (m, 1H), 2.55-2.47 (m, 1H); ^{13}**C-NMR** (150 MHz, $CDCl_3$) δ 134.9, 134.2, 133.5, 132.7, 129.7, 127.92, 127.90, 125.5 (q, *J* = 278.0 Hz), 118.8, 50.6 (q, *J* = 28.2 Hz), 33.1 (q, *J* = 2.1 Hz); ^{19}**F-NMR** (564 MHz, $CDCl_3$) δ -69.97 (d, *J* = 8.5 Hz). **IR** (KBr): 3080, 2923, 2853, 1645, 1447, 1347, 1252, 1165, 1108, 1021, 922, 750 cm^{-1}. **MS** (EI, m/z): 310 (M^+, 15.96), 190 (100.00). **HRMS** (EI): exact mass calcd for $C_{11}H_{10}BrF_3S$ (M^+): 309.9639, found: 309.9644.

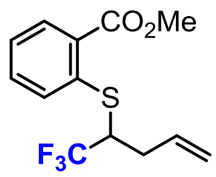

Ethyl 2-((1,1,1-trifluoropent-4-en-2-yl)thio)benzoate (80) Colourless oil; **^{1}H-NMR** (600 MHz, $CDCl_3$) δ 7.85 (dd, *J* = 7.8, 1.8 Hz, 1H), 7.52 (d, *J* = 7.8 Hz, 1H), 7.44 (td, *J* = 7.2, 1.8 Hz, 1H), 7.28 (td, *J* = 7.2, 1.2 Hz, 1H), 5.94-5.90 (m, 1H), 5.21 (dq, *J* = 17.4, 1.8 Hz, 1H), 5.16 (dq, *J* = 10.2, 1.2 Hz, 1H), 3.92 (s, 3H), 3.81 -3.77 (m, 1H), 2.79-2.70 (m, 1H), 2.54-2.47 (m, 1H); **^{13}C-NMR** (150 MHz, $CDCl_3$) δ 167.0, 136.2, 132.9, 132.0, 131.9, 130.9, 130.7, 126.6, 126.5 (q, *J* = 277.5 Hz), 118.7, 52.3, 50.6 (q, *J* = 27.9 Hz), 33.1 (q, *J* = 2.0 Hz); **^{19}F-NMR** (564 MHz, $CDCl_3$) δ -69.53 (d, *J* = 7.9 Hz). **IR** (KBr): 3084, 2923, 2852, 1733, 1636, 1153, 670 cm^{-1}. **HRMS** (EI): exact mass calcd for $C_{13}H_{13}F_3O_2S$ (M^+): 290.0588, found: 290.0585.

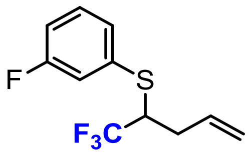

(3-fluorophenyl)(1,1,1-trifluoropent-4-en-2-yl)sulfane (81) Colourless oil; **^{1}H-NMR** (600 MHz, $CDCl_3$) δ 7.30-7.25 (m, 2H), 7.23-7.19 (m, 1H), 7.03-6.98 (m, 1H), 5.97-5.88 (m, 1H), 5.23 (dq, *J* = 6.6, 1.2 Hz, 1H), 5.21 (t, *J* = 1.8 Hz, 1H), 3.46-3.38 (m, 1H), 2.72-2.65 (m, 1H), 2.44-2.36 (m, 1H); **^{13}C-NMR** (150 MHz, $CDCl_3$) δ 162.5 (d, *J* = 249.3 Hz), 135.2 (d, *J* = 7.8 Hz), 132.8, 130.4 (d, *J* = 8.6 Hz), 128.7 (d, *J* = 3.2 Hz), 126.3 (q, J = 277.7 Hz), 119.8 (d, *J* = 22.3 Hz), 119.0, 115.4 (d, *J* = 21.3 Hz), 52.3 (q, *J* = 28.2 Hz), 32.9 (q, *J* = 2.0 Hz); **^{19}F-NMR** (564 MHz, $CDCl_3$) δ -70.16 (d, *J* = 7.9 Hz), -111.67 (d, *J* = 5.6 Hz). **IR** (KBr): 2945, 2840, 1645, 1344,. 1310, 1175, 900 cm^{-1}. **MS** (EI, m/z): 250 (M^+, 92.06), 209 (100.00). **HRMS** (EI): exact mass calcd for $C_{11}H_{10}F_4S$ (M^+): 250.0439, found: 250.0438.

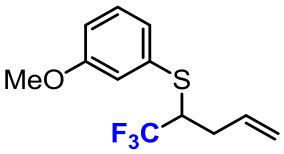

(3-methoxyphenyl)(1,1,1-trifluoropent-4-en-2-yl)sulfane (82) Colourless oil; **^{1}H-NMR** (600 MHz, $CDCl_3$) δ 7.20 (t, *J* = 7.8 Hz, 1H), 7.07 (d, *J* = 7.8 Hz, 1H), 7.04 (t, *J* = 1.8 Hz, 1H), 6.82 (dd, *J* = 7.8, 1.8 Hz, 1H), 5.98-5.89 (m, 1H), 5.23-5.17 (m, 2H), 3.76 (s, 3H), 3.45-3.37 (m, 1H), 2.69-2.63 (m, 1H), 2.43-2.36 (m, 1H); **^{13}C-NMR** (150 MHz, $CDCl_3$) δ 159.8, 134.2, 133.1, 129.9,

126.5 (q, J = 277.7 Hz), 125.3, 118.6, 118.5, 114.1, 55.1, 52.3 (q, J = 27.9 Hz), 33.0 (q, J = 1.7 Hz); **^{19}F-NMR** (564 MHz, $CDCl_3$) δ -70.04 (d, J = 8.5 Hz). **IR** (KBr): 3082, 2945, 2840, 1645, 1594, 1494, 1348,. 1288, 1248, 1166, 1101, 919, 829 cm^{-1}. **MS** (EI, m/z): 262 (M^+, 100), 221 (11.08), 165 (79.42). **HRMS** (EI): exact mass calcd for $C_{12}H_{13}F_3OS$ (M^+): 262.0639, found: 262.0641.

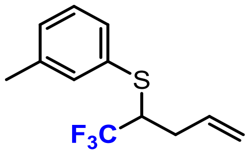

M-tolyl(1,1,1-trifluoropent-4-en-2-yl)sulfane (83) Colourless oil; **^{1}H-NMR** (600 MHz, $CDCl_3$) δ 7.22 (s, 1H), 7.20 (d, J = 7.8 Hz, 1H), 7.08 (t, J = 7.8 Hz, 1H), 6.99 (d, J = 7.8 Hz, 1H), 5.89-5.80 (m, 1H), 5.12-5.09 (m, 1H), 5.09-5.07 (m, 1H), 3.32-3.24 (m, 1H), 2.58-2.52 (m, 1H), 2.33-2.25 (m, 1H), 2.21 (s, 3H); **^{13}C-NMR** (150 MHz, $CDCl_3$) δ 140.0, 134.0, 133.2, 132.8, 130.4, 129.2, 128.9, 126.5 (q, J = 277.8 Hz), 118.6, 52.4 (q, J = 28.1 Hz), 33.1 (q, J = 1.8 Hz), 21.1; **^{19}F-NMR** (564 MHz, $CDCl_3$) δ -69.99 (d, J = 7.9 Hz). **IR** (KBr): 3082, 2924, 1644, 1475, 1349, 1252, 1163, 1105, 920, 779, 688 cm^{-1}. **MS** (EI, m/z): 246 (M^+, 96.25), 205 (100.00), 185 (42.12). **HRMS** (EI): exact mass calcd for $C_{12}H_{13}F_3S$ (M^+): 246.0690, found: 246.0693.

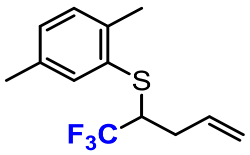

(2,5-dimethylphenyl)(1,1,1-trifluoropent-4-en-2-yl)sulfane (84) Colourless oil; **^{1}H-NMR** (600 MHz, $CDCl_3$) δ 7.30 (s, 1H), 7.08 (d, J = 7.2 Hz, 1H), 6.99 (d, J = 7.8 Hz, 1H), 5.99-5.91 (m, 1H), 5.23-5.16 (m, 2H), 3.42-3.33 (m, 1H), 2.71-2.64 (m, 1H), 2.47-2.40 (m, 4H), 2.28 (s, 3H); **^{13}C-NMR** (150 MHz, $CDCl_3$) δ 137.8, 136.2, 134.6, 133.2, 132.2, 130.4, 129.4, 126.6 (q, J = 278.0 Hz), 118.6, 52.0 (q, J = 27.8 Hz), 33.4 (q, J = 1.8 Hz), 20.7, 20.3; **^{19}F-NMR** (564 MHz, $CDCl_3$) δ -70.03 (d, J = 7.9 Hz). **IR** (KBr): 3028, 2923, 1644, 1490, 1453, 1322, 1253, 1150, 1101, 924, 812, 700 cm^{-1}. **MS** (EI, m/z): 260 (M^+, 95.11), 219 (100.00). **HRMS** (EI): exact mass calcd for $C_{13}H_{15}F_3S$ (M^+): 260.0847, found: 260.0845.

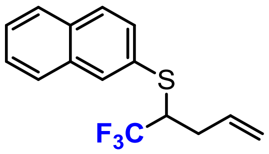

Naphthalen-2-yl(1,1,1-trifluoropent-4-en-2-yl)sulfane (85) Colourless oil; **^{1}H-NMR** (600 MHz, $CDCl_3$) δ 8.01 (d, *J* = 1.8 Hz, 1H), 7.83-7.79 (m, 1H), 7.79-7.76 (m, 2H), 7.54 (dd, *J* = 9.0, 1.8 Hz, 1H), 7.51-7.47 (m, 2H), 6.03-5.94 (m, 1H), 5.26-5.23 (m, 1H), 5.22-5.21 (m, 1H), 3.53-3.46 (m, 1H), 2.73-2.66 (m, 1H), 2.48-2.40 (m, 1H); **^{13}C-NMR** (150 MHz, $CDCl_3$) δ 133.5, 133.1, 133.0, 132.8, 130.2, 128.8, 127.7, 127.6, 126.8, 126.7, 126.5 (q, *J* = 277.7 Hz), 118.8, 52.4 (q, *J* = 28.1 Hz), 33.0 (q, *J* = 2.0 Hz); **^{19}F-NMR** (564 MHz, $CDCl_3$) δ -69.89 (d, *J* = 7.9 Hz). **IR** (KBr): 3859, 3057, 3002, 2321, 2109, 1750, 1438, 1366, 1213, 1022, 918 cm^{-1}. **MS** (EI, m/z): 282 (M^+, 89.23), 241 (100.00). **HRMS** (EI): exact mass calcd for $C_{15}H_{13}F_3S$ (M^+): 282.0690, found: 282.0692.

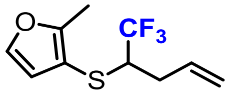

2-methyl-3-((1,1,1-trifluoropent-4-en-2-yl)thio)furan (86) Colourless oil; **^{1}H-NMR** (600 MHz, $CDCl_3$) δ 7.27 (d, *J* = 2.4 Hz, 1H), 6.36 (d, *J* = 2.4 Hz, 1H), 5.95-5.91 (m, 1H), 5.23-5.20 (m, 1H), 5.20-5.18 (m, 1H), 3.08-3.02 (m, 1H), 2.63-2.56 (m, 1H), 2.35 (s, 3H), 2.34-2.29 (m, 1H); **^{13}C-NMR** (150 MHz, $CDCl_3$) δ 157.4, 140.7, 133.3, 126.4 (q, *J* = 277.5 Hz), 118.4, 115.6, 107.4, 51.7 (q, *J* = 27.6 Hz), 32.3 (q, *J* = 1.8 Hz), 11.8; **^{19}F-NMR** (564 MHz, $CDCl_3$) δ -70.33 (d, *J* = 8.5 Hz). **IR** (KBr): 3081, 2922, 1444, 1254, 1223, 1125, 1088, 917 cm^{-1}. **MS** (EI, m/z): 236 (M^+, 54.31), 195 (100.00). **HRMS** (EI): exact mass calcd for $C_{10}H_{11}F_3OS$ (M^+): 236.0483, found: 236.0485.

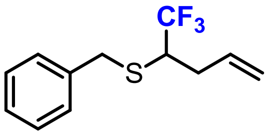

Benzyl(1,1,1-trifluoropent-4-en-2-yl)sulfane (87) Colourless oil; **^{1}H-NMR** (600 MHz, $CDCl_3$) δ 7.34-7.29 (m, 4H), 7.28-7.24 (m, 1H), 5.68-5.61 (m, 1H), 5.06 (t, *J* = 6.0 Hz, 1H), 5.05-5.02 (m, 1H), 3.86 (d, *J* = 13.2 Hz, 1H), 3.82 (d, *J* = 13.2 Hz, 1H), 2.94-2.87 (m, 1H), 2.54-2.50 (m, 1H), 2.29-2.24 (m, 1H); **^{13}C-NMR** (150 MHz, $CDCl_3$) δ 136.7, 133.0, 129.2, 128.6, 127.5, 127.2 (q, *J* = 277.5 Hz), 118.1, 46.0 (q, *J* = 28.2 Hz), 36.6, 32.6 (q, *J* = 2.1 Hz); **^{19}F-NMR** (564 MHz, $CDCl_3$) δ -70.12 (d, *J* = 8.5 Hz). **IR** (KBr): 3083, 3031, 2961, 2926, 1645, 1515, 1455, 1346, 1260, 1102, 1019, 920, 801, 700 cm^{-1}. **MS** (EI, m/z): 246 (M^+, 11.79), 155 (6.39), 91 (100.00). **HRMS** (EI): exact mass calcd for $C_{12}H_{13}F_3S$ (M^+): 246.0690, found: 246.0692.

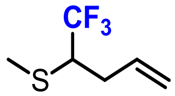

Methyl(1,1,1-trifluoropent-4-en-2-yl)sulfane (88) Colourless oil; ^{1}**H-NMR** (600 MHz, $CDCl_3$) δ 5.91-5.82 (m, 1H), 5.21-5.15 (m, 2H), 3.03-2.97 (m, 1H), 2.66-2.61 (m, 1H), 2.37-2.31 (m, 1H), 2.21 (d, J = 0.6 Hz, 3H); ^{13}**C-NMR** (150 MHz, $CDCl_3$) δ 133.2, 127.1 (q, J = 277.8 Hz), 118.2, 48.9 (q, J = 28.1 Hz), 32.2 (q, J = 2.1 Hz), 15.1; ^{19}**F-NMR** (564 MHz, $CDCl_3$) δ -70.35 (d, J = 8.5 Hz). **IR** (KBr): 2984, 1441, 1371, 1214, 1153, 920 cm^{-1}. **HRMS** (EI): exact mass calcd for $C_6H_9F_3S$ (M^+): 170.0377, found: 170.0375.

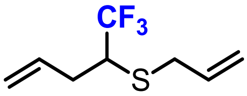

Allyl(1,1,1-trifluoropent-4-en-2-yl)sulfane (89) Colourless oil; ^{1}**H-NMR** (600 MHz, $CDCl_3$) δ 5.87-5.71 (m, 2H), 5.23-5.11 (m, 4H), 3.32 (dd, J = 13.2, 9.0 Hz, 1H), 3.21 (dd, J = 13.2, 5.4 Hz, 1H), 3.11-3.04 (m, 1H), 2.63-2.57 (m, 1H), 2.36-2.29 (m, 1H); ^{13}**C-NMR** (150 MHz, $CDCl_3$) δ 133.2, 133.1, 127.1 (q, J = 277.4 Hz), 118.6, 118.3, 45.5 (q, J = 28.1 Hz), 35.4, 32.8 (q, J = 2.1 Hz); ^{19}**F-NMR** (564 MHz, $CDCl_3$) δ -70.35 (d, J = 8.5 Hz). **IR** (KBr): 2981, 1367, 1341, 1258, 1231, 1155, 1031, 990, 917 cm^{-1}. **HRMS** (EI): exact mass calcd for $C_8H_{11}F_3S$ (M^+): 196.0534, found: 196.0536.

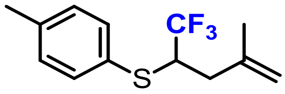

P-tolyl(1,1,1-trifluoro-4-methylpent-4-en-2-yl)sulfane (90) Colourless oil; ^{1}**H-NMR** (600 MHz, $CDCl_3$) δ 7.40 (d, J = 8.0 Hz, 2H), 7.11 (d, J = 8.0 Hz, 2H), 4.98-4.96 (m, 1H), 4.89 (s, 1H), 3.45 -3.40 (m, 1H), 2.58 (dd, J = 15.0, 3.6 Hz, 1H), 2.33 (s, 3H), 2.28 (dd, J = 15.0, 11.4 Hz, 1H), 1.77 (s, 3H); ^{13}**C-NMR** (150 MHz, $CDCl_3$) δ 139.9, 138.8, 134.3, 129.8, 129.3, 126.7 (q, J = 277.5 Hz), 114.5, 50.9 (q, J = 27.8 Hz), 36.5 (q, J = 1.7 Hz), 22.0, 21.1; ^{19}**F-NMR** (564 MHz, $CDCl_3$) δ -70.23 (d, J = 8.5 Hz). **IR** (KBr): 2922, 1467, 1245, 1171, 1105, 830 cm^{-1}. **MS** (EI, m/z): 260 (M^+, 100.00), 240 (23.54). **HRMS** (EI): exact mass calcd for $C_{13}H_{15}F_3S$ (M^+): 260.0847, found: 260.0849.

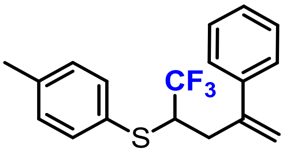

P-tolyl(1,1,1-trifluoro-4-phenylpent-4-en-2-yl)sulfane (91) Colourless oil; **^{1}H-NMR** (600 MHz, $CDCl_3$) δ 7.36 (d, *J* = 7.2 Hz, 2H), 7.33 (t, *J* = 7.8 Hz, 2H), 7.31-7.26 (m, 1H), 7.14 (d, *J* = 7.8 Hz, 2H), 6.98 (d, *J* = 7.8 Hz, 2H), 5.44 (s, 1H), 5.24 (s, 1H), 3.37-3.27 (m, 1H), 3.22 (d, *J* = 15.6 Hz, 1H), 2.66 (dd, *J* = 15.0, 11.4 Hz, 1H), 2.26 (s, 3H); **^{13}C-NMR** (150 MHz, $CDCl_3$) δ 143.5, 139.4, 138.6, 134.1, 129.7, 129.2, 128.6, 127.9, 126.8 (q, *J* = 277.7 Hz), 126.5, 116.8, 51.2 (q, *J* = 27.8 Hz), 34.6 (d, *J* = 0.8 Hz), 21.0; **^{19}F-NMR** (564 MHz, $CDCl_3$) δ -70.15 (d, *J* = 8.5 Hz). **IR** (KBr): 2924, 1443, 1241, 1153, 829 cm^{-1}. **HRMS** (ESI) m/z calculated for $C_{18}H_{17}F_3NaS$ $[M+Na]^+$ 345.0901, found 345.0908.

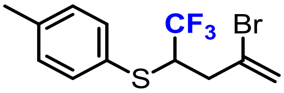

(4-bromo-1,1,1-trifluoropent-4-en-2-yl)(p-tolyl)sulfane (92) Colourless oil; **^{1}H-NMR** (600 MHz, $CDCl_3$) δ 7.45 (d, *J* = 7.8 Hz, 2H), 7.11 (d, *J* = 7.8 Hz, 2H), 5.77 (t, *J* = 2.0 Hz, 1H), 5.65-5.62 (m, 1H), 3.74-3.63 (m, 1H), 2.92 (dd, *J* = 15.0, 3.6 Hz, 1H), 2.64 (dd, *J* = 15.0, 10.8 Hz, 1H), 2.31 (s, 3H); **^{13}C-NMR** (150 MHz, $CDCl_3$) δ 139.0, 134.5, 129.9, 128.7, 128.3, 126.3 (q, *J* = 277.7 Hz), 121.3, 51.3 (q, *J* = 28.2 Hz), 40.5 (q, *J* = 1.8 Hz), 21.1; **^{19}F-NMR** (564 MHz, $CDCl_3$) δ -69.95 (d, *J* = 8.5 Hz). **IR** (KBr): 2923, 1433, 1235, 1170, 1131, 671 cm^{-1}. **MS** (EI, m/z): 324 (M^+, 41.03), 205 (100.00). **HRMS** (EI): exact mass calcd for $C_{12}H_{12}BrF_3S$ (M^+): 323.9795, found: 323.9797.

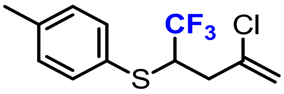

(4-chloro-1,1,1-trifluoropent-4-en-2-yl)(p-tolyl)sulfane (93) Colourless oil; **^{1}H-NMR** (600 MHz, $CDCl_3$) δ 7.34 (d, *J* = 7.8 Hz, 2H), 7.01 (d, *J* = 7.8 Hz, 2H), 5.30 (s, 1H), 5.24 (s, 1H), 3.61-3.54 (m, 1H), 2.76 (dd, *J* = 14.8, 3.4 Hz, 1H), 2.48 (dd, *J* = 14.8, 10.8 Hz, 1H), 2.22 (s, 3H); **^{13}C-NMR** (150 MHz, $CDCl_3$) δ 139.1, 137.0, 134.5, 129.9, 128.7, 126.4 (q, *J* = 277.4 Hz), 116.8, 50.6 (q, *J* = 28.4 Hz), 38.5 (q, *J* = 1.8 Hz), 21.1; **^{19}F-NMR** (564 MHz, $CDCl_3$) δ -70.12 (d, *J* = 8.5 Hz). **IR**

(KBr): 2925, 1248, 1157, 1104, 743 cm^{-1}. **MS** (EI, m/z): 280 (M$^+$, 59.56), 205 (100.00). **HRMS** (EI): exact mass calcd for $C_{12}H_{12}ClF_3S$ (M$^+$): 280.0300, found: 280.0303.

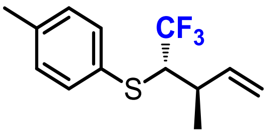

P-tolyl(1,1,1-trifluoro-3-methylpent-4-en-2-yl)sulfane (94), dr = 5:1, Colourless oil; **^{1}H-NMR** (600 MHz, $CDCl_3$) δ 7.40 (d, *J* = 7.8 Hz, 2H), 7.10 (d, *J* = 7.8 Hz, 2H), 5.91-5.85 (m, 1H), 5.17-5.10 (m, 2H), 3.26 (qd, *J* = 9.0, 3.0 Hz, 1H), 2.94-2.90 (m, 1H), 2.32 (s, 3H), 1.32 (d, *J* = 6.6 Hz, 3H); **^{13}C-NMR** (150 MHz, $CDCl_3$) δ 138.5, 137.8, 133.5, 131.2, 130.0, 126.7 (q, *J* = 278.3 Hz), 116.6, 57.5 (q, *J* = 26.3 Hz), 37.4, 21.1, 18.7; **^{19}F-NMR** (564 MHz, $CDCl_3$) δ -67.22 (d, *J* = 9.0 Hz). **IR** (KBr): 2920, 1460, 1368, 1215, 1171, 829 cm^{-1}. **HRMS** (EI): exact mass calcd for $C_{13}H_{15}F_3S$ (M$^+$): 260.0847, found: 260.0849.

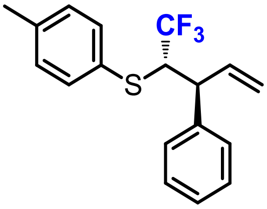

P-tolyl(1,1,1-trifluoro-3-phenylpent-4-en-2-yl)sulfane (95), dr = 10:1, Colourless oil; **^{1}H-NMR** (600 MHz, $CDCl_3$) δ 7.40-7.37 (m, 2H), 7.36-7.33 (m, 2H), 7.30-7.25 (m, 1H), 6.99 (d, *J* = 8.4 Hz, 2H), 6.96 (d, *J* = 8.4 Hz, 2H), 6.32-6.24 (m, 1H), 5.28 (d, *J* = 10.2 Hz, 1H), 5.21 (d, *J* = 16.8 Hz, 1H), 4.04 (dd, *J* = 9.0, 3.6 Hz, 1H), 3.51 (qd, *J* = 9.0, 3.6 Hz, 1H), 2.26 (s, 3H); **^{13}C-NMR** (150 MHz, $CDCl_3$) δ 141.4, 138.5, 135.1, 133.5, 130.6, 129.8, 128.5, 128.3, 127.2, 126.5 (q, *J* = 278.6 Hz), 118.9, 61.0 (q, *J* = 26.3 Hz), 48.4 (q, *J* = 1.7 Hz), 21.1; **^{19}F-NMR** (564 MHz, $CDCl_3$) δ -67.75 (d, *J* = 8.5 Hz). **IR** (KBr): 2979, 2954, 1644, 1219, 1131, 918, 836 cm^{-1}. **HRMS** (ESI) m/z calculated for $C_{18}H_{17}F_3NaS$ [M+Na]$^+$ 345.0901, found 345.0904.

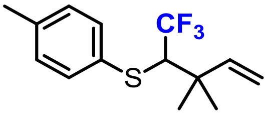

P-tolyl(1,1,1-trifluoro-3,3-dimethylpent-4-en-2-yl)sulfane (96) Colourless oil; **^{1}H-NMR** (600 MHz, $CDCl_3$) δ 7.40 (d, *J* = 7.8 Hz, 2H), 7.12 (d, *J* = 7.8 Hz, 2H), 6.00-5.94 (m, 1H), 5.12-5.06 (m, 2H), 3.16 (q, *J* = 9.0 Hz, 1H), 2.33 (s, 3H), 1.31 (s, 6H); **^{13}C-NMR** (150 MHz, $CDCl_3$) δ 144.3,

138.3, 133.1, 132.0, 129.9, 127.1 (q, *J* = 279.5 Hz), 112.7, 64.1 (q, *J* = 24.9 Hz), 40.1, 26.5 (q, *J* = 2.3 Hz), 25.0 (q, *J* = 1.7 Hz), 21.1; **^{19}F-NMR** (564 MHz, $CDCl_3$) δ -61.96 (d, *J* = 9.0 Hz). **IR** (KBr): 3088, 2954, 1261, 1153, 917, 836 cm^{-1}. **HRMS** (EI): exact mass calcd for $C_{14}H_{17}F_3S$ (M^+): 274.1003, found: 274.1005.

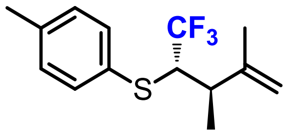

P-tolyl(1,1,1-trifluoro-3,4-dimethylpent-4-en-2-yl)sulfane (Stereoisomers 97), dr = 4:1, Colorless oil; **^{1}H-NMR** (600 MHz, $CDCl_3$) δ 7.37-7.40 (m, 4H, major+minor), 7.09 (m, 4H, major+minor), 4.97 (s, 2H, minor), 4.88 (s, 2H, major), 3.42-3.31 (m, 2H, major+minor), 2.74-2.77 (m, 2H, major+minor), 2.31 (s, 3H, major), 2.30 (s, 3H, minor), 1.76 (s, 3H, major), 1.72 (s, 3H, minor), 1.29 (d, *J* = 7.2 Hz, 3H, major), 1.24 (d, *J* = 7.2 Hz, 3H, minor); **^{13}C-NMR** (150 MHz, $CDCl_3$) δ 145.8, 145.5, 138.48, 138.45, 133.7, 133.5, 131.1, 130.9, 130.0, 129.8, 127.1 (d, *J* = 280.3 Hz), 126.9 (q, *J* = 278.7 Hz), 113.21, 113.20, 57.6 (q, *J* = 26.3 Hz), 57.5 (d, *J* = 26.3 Hz), 41.3, 39.4, 21.18, 21.11, 20.3, 18.3, 14.8; **^{19}F-NMR** (564 MHz, CDCl3) δ -65.39 (d, *J* = 8.5 Hz, major), -67.56 (d, *J* = 9.0 Hz, minor). **IR** (KBr): 2924, 1331, 1220, 1147, 836 cm^{-1}. **HRMS** (EI): exact mass calcd for $C_{14}H_{17}F_3S$ (M^+): 274.1003, found: 274.1004.

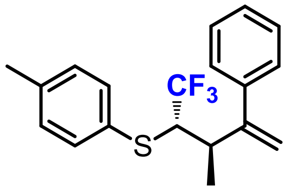

P-tolyl(1,1,1-trifluoro-3-methyl-4-phenylpent-4-en-2-yl)sulfane (Stereoisomers 98), dr = 4:1, Colorless oil; **^{1}H-NMR** (600 MHz, $CDCl_3$) δ 7.18-7.16 (m, 3H, major+minor), 7.16-7.13 (m, 5H, major+minor), 7.10-7.08 (m, 3H, major), 7.04 (d, *J* = 7.8 Hz, minor, 3H), 6.96 (d, *J* = 8.4 Hz, 2H, major), 6.86 (d, *J* = 7.8 Hz, 2H, minor), 5.36 (s, 1H, minor), 5.23 (s, 1H, minor,), 5.21 (s, 1H, major), 5.12 (s, 1H, major,), 3.44 (q, *J* = 6.0 Hz, 1H, minor), 3.36-3.31 (m, 1H, major), 3.26-3.24 (m, 1H, minor), 3.21-3.17 (m, 1H, major), 2.21 (s, 3H, major), 2.15 (s, 3H, minor), 1.37 (d, *J* = 6.6 Hz, 3H, major,), 1.28 (d, *J* = 6.6 Hz, 3H, minor); **^{13}C-NMR** (150 MHz, $CDCl_3$) δ 150.3, 149.3, 141.5, 140.8, 138.6, 138.0, 133.7, 133.2, 130.5, 130.1, 129.9, 129.7, 128.52, 128.49 (d, *J* = 274.5 Hz), 128.3, 127.8, 127.5, 126.8, 126.7, 126.6 (q, *J* = 287.4 Hz), 115.6, 114.3, 56.7 (q, *J* = 26.7 Hz),

56.4 (d, J = 26.0 Hz), 39.7, 36.0, 21.1, 21.0, 15.8, 13.5; **^{19}F-NMR** (565 MHz, CDCl3) δ -63.57 (d, J = 9.0 Hz, major), -67.49 (d, J = 9.6 Hz, minor). **IR** (KBr): 2920, 1653, 1217, 1113 cm^{-1}. **MS** (EI, m/z): 336 (M$^+$, 16.21), 117 (100.00). **HRMS** (EI): exact mass calcd for $C_{19}H_{19}F_3S$ (M$^+$): 336.1160, found: 336.1170.

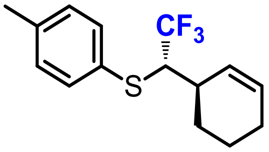

(1-cyclohex-2-en-1-yl)-2,2,2-trifluoroethyl)(p-tolyl)sulfane (Stereoisomers 99), dr = 7:1, Colorless oil; **^{1}H-NMR** (600 MHz, $CDCl_3$) δ 7.43-7.38 (m, 4H), 7.10 (d, J = 7.8 Hz, 4H), 5.93-5.87 (m, 2H), 5.72 (d, J = 10.2 Hz, 1H), 5.46 (d, J = 10.2 Hz, 1H), 3.39-3.31 (m, 1H), 3.23-3.18 (qd, J = 9.0, 3.0 Hz, 1H), 2.91-2.82 (m, 2H), 2.32 (s, 6H), 2.09-2.00 (m, 4H), 1.95-1.86 (m, 3H), 1.84-1.81 (m, 2H), 1.65-1.49 (m, 3H); **^{13}C-NMR** (150 MHz, $CDCl_3$) δ 138.4, 138.3, 133.8, 133.3, 132.5, 131.2, 131.1, 131.0, 130.4, 130.0, 129.90, 129.88, 128.3, 127.1 (d, J = 278.6 Hz), 126.9 (d, J = 278.6 Hz), 125.8 (d, J = 1.1 Hz), 59.2 (q, J = 26.3 Hz), 58.5 (q, J = 26.4 Hz), 36.2, 35.7, 28.0, 24.8, 24.77, 24.76, 21.8, 21.5, 21.06, 21.04; **^{19}F-NMR** (565 MHz, CDCl3) δ -66.59 (d, J = 9.6 Hz), -67.57 (d, J = 9.6 Hz). **IR** (KBr): 2933, 2856, 1650, 1451, 793 cm^{-1}. **HRMS** (EI): exact mass calcd for $C_{15}H_{17}F_3S$ (M$^+$): 286.1003, found: 286.1005.

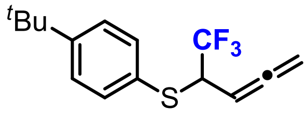

(4-(tert-butyl)phenyl)(1,1,1-trifluoropenta-3,4-dien-2-yl)sulfane (100) Colourless oil; **^{1}H-NMR** (600 MHz, $CDCl_3$) δ 7.44 (d, J = 8.4 Hz, 2H), 7.34 (d, J = 8.4 Hz, 2H), 5.19-5.15 (m, 1H), 4.82 (dd, J = 12.0, 7.2 Hz, 1H), 4.74 (dd, J = 12.0, 7.2 Hz, 1H), 3.97 (p, J = 7.8 Hz, 1H), 1.30 (s, 9H); **^{13}C-NMR** (150 MHz, $CDCl_3$) δ 210.0, 152.3, 134.6, 128.2, 126.1, 125.4 (q, J = 277.8 Hz), 84.8 (q, J = 2.4 Hz), 77.6, 51.9 (q, J = 29.3 Hz), 34.6, 31.2; **^{19}F-NMR** (564 MHz, $CDCl_3$) δ -69.62 (d, J = 7.3 Hz). **IR** (KBr): 2923, 2858, 1943, 1490, 1348, 1131, 1103, 924, 829 cm^{-1}. **HRMS** (ESI) m/z calculated for $C_{15}H_{18}F_3S$ [M+H]$^+$ 287.1081, found 287.1086.

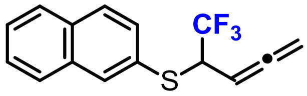

Naphthalen-2-yl(1,1,1-trifluoropenta-3,4-dien-2-yl)sulfane (101) Colourless oil; **^{1}H-NMR** (600 MHz, $CDCl_3$) δ 8.02 (s, 1H), 7.78-7.75 (m, 3H), 7.54 (d, *J* = 8.4 Hz, 1H), 7.50-7.48 (m, 2H), 5.26 -5.20 (m, 1H), 4.85 (dd, *J* = 12.0, 6.6 Hz, 1H), 4.76 (dd, *J* = 12.0, 6.6 Hz, 1H), 4.17-4.10 (m, 1H); **^{13}C-NMR** (150 MHz, $CDCl_3$) δ 210.1, 134.0, 133.5, 132.9, 130.8, 128.9, 128.7, 127.72, 127.69, 126.9, 126.7, 125.3 (q, *J* = 277.8 Hz), 84.7 (q, *J* = 2.4 Hz), 77.9, 51.6 (q, *J* = 29.4 Hz); **^{19}F-NMR** (564 MHz, $CDCl_3$) δ -69.54 (d, *J* = 7.9 Hz). **IR** (KBr): 3051, 2924, 2850, 1650, 1506, 1271, 1157, 1506, 1125, 869, 819, 749 cm^{-1}. **HRMS** (ESI) m/z calculated for $C_{15}H_{12}F_3S$ $[M+H]^+$ 281.0612, found 281.0619.

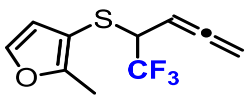

2-methyl-3-((1,1,1-trifluoropenta-3,4-dien-2-yl)thio)furan (102) Colourless oil; **^{1}H-NMR** (600 MHz, $CDCl_3$) δ 7.28 (s, 1H), 6.36 (s, 1H), 5.14-5.10 (m, 1H), 4.91-4.83 (m, 2H), 3.78-3.70 (m, 1H), 2.36 (s, 3H); **^{13}C-NMR** (150 MHz, $CDCl_3$) δ 210.0, 158.1, 140.7, 125.3 (q, *J* = 277.7 Hz), 116.0, 106.6, 84.2 (q, *J* = 2.4 Hz), 77.5, 50.6 (q, *J* = 29.1 Hz), 11.8; **^{19}F-NMR** (564 MHz, $CDCl_3$) δ -70.00 (d, *J* = 7.9 Hz). **IR** (KBr): 2924, 1471, 1434, 1271, 1254, 1214, 1173, 1125, 920 cm^{-1}. **HRMS** (ESI) m/z calculated for $C_{10}H_{10}F_3OS$ $[M+H]^+$ 235.0404, found 235.0409.

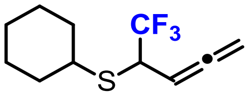

Cyclohexyl(1,1,1-trifluoropenta-3,4-dien-2-yl)sulfane (103) Colourless oil; **^{1}H-NMR** (600 MHz, $CDCl_3$) δ 5.20 (dd, *J* = 15.6, 6.6 Hz, 1H), 4.98-4.92 (m, 2H), 3.83 (p, *J* = 8.4 Hz, 1H), 2.90-2.83 (m, 1H), 2.04-1.92 (m, 2H), 1.83-1.73 (m, 2H), 1.65-1.60 (m, 1H), 1.43-1.24 (m, 5H); **^{13}C-NMR** (150 MHz, $CDCl_3$) δ 209.8, 125.6 (q, *J* = 277.1 Hz), 85.5 (q, *J* = 2.4 Hz), 77.7, 45.8 (q, *J* = 30.0 Hz), 43.9, 33.4, 33.3, 25.89, 25.84, 25.6; **^{19}F-NMR** (564 MHz, $CDCl_3$) δ -70.25 (d, *J* = 7.9 Hz). **IR** (KBr): 2933, 2856, 1957, 1451, 1295, 1250, 1161, 1103, 854, 693 cm^{-1}. **MS** (EI, m/z): 236 (M^+, 100.00), 216 (17.15). **HRMS** (EI): exact mass calcd for $C_{11}H_{15}F_3S$ (M^+): 236.0847, found: 236.0845.

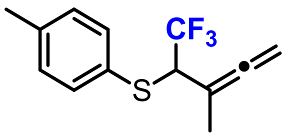

P-tolyl(1,1,1-trifluoro-3-methylpenta-3,4-dien-2-yl)sulfane (104) Colourless oil; **^{1}H-NMR** (600 MHz, $CDCl_3$) δ 7.39 (d, *J* = 7.8 Hz, 2H), 7.12 (d, *J* = 7.8 Hz, 2H), 4.66-4.57 (m, 2H), 3.87 (q, *J* = 8.4 Hz, 1H), 2.33 (s, 3H), 1.86 (s, 3H); **^{13}C-NMR** (150 MHz, $CDCl_3$) δ 208.3, 139.0, 134.3, 129.8, 129.1, 125.4 (q, *J* = 278.4 Hz), 92.5, 75.8, 56.8 (q, *J* = 28.8 Hz), 21.1, 16.1; **^{19}F-NMR** (564 MHz, $CDCl_3$) δ -67.23 (d, *J* = 8.5 Hz). **IR** (KBr): 2920, 1595, 1491, 1427, 1304, 1266, 1016, 857, 810 cm^{-1}. **HRMS** (ESI) m/z calculated for $C_{13}H_{14}F_3S$ $[M+H]^+$ 259.0768, found 259.0774.

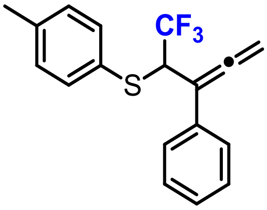

P-tolyl(1,1,1-trifluoro-3-phenylpenta-3,4-dien-2-yl)sulfane (105) Colourless oil; **^{1}H-NMR** (600 MHz, $CDCl_3$) δ 7.40 (d, *J* = 7.8 Hz, 2H), 7.36 (d, *J* = 7.8 Hz, 2H), 7.32 (t, *J* = 7.2 Hz, 2H), 7.23 (t, *J* = 7.2 Hz, 1H), 7.06 (d, *J* = 7.8 Hz, 2H), 5.09 (d, *J* = 12.6 Hz, 1H), 4.88 (d, *J* = 12.6 Hz, 1H), 4.34 (q, *J* = 7.8 Hz, 1H), 2.29 (s, 3H); **^{13}C-NMR** (150 MHz, $CDCl_3$) δ 209.6, 139.4, 135.6, 134.5, 129.6, 128.7, 128.1, 127.5, 126.2, 125.7 (q, *J* = 277.8 Hz), 99.4, 80.6, 52.0 (q, *J* = 28.8 Hz), 21.1; **^{19}F-NMR** (564 MHz, $CDCl_3$) δ -66.99 (d, *J* = 7.9 Hz). **IR** (KBr): 2920, 1487, 1433, 1311, 1276, 855 cm^{-1}. **HRMS** (ESI) m/z calculated for $C_{18}H_{16}F_3S$ $[M+H]^+$ 321.0925, found 321.0928.

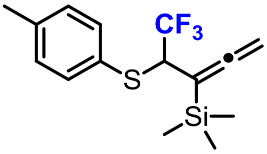

Trimethyl(5,5,5-trifluoro-4-(p-tolylthio)penta-1,2-dien-3-yl)silane (106) Colourless oil; **^{1}H-NMR** (600 MHz, $CDCl_3$) δ 7.32 (d, *J* = 7.8 Hz, 2H), 7.02 (d, *J* = 7.8 Hz, 2H), 4.43 (d, *J* = 12.6 Hz, 1H), 4.35 (d, *J* = 12.6 Hz, 1H), 3.68 (q, *J* = 7.8 Hz, 1H), 2.23 (s, 3H), 0.10 (s, 9H); **^{13}C-NMR** (150 MHz, $CDCl_3$) δ 211.5, 139.0, 134.8, 129.7, 129.5, 125.9 (q, *J* = 278.9 Hz), 89.3, 71.8, 51.5 (q, *J* = 28.1 Hz), 21.1, -1.6; **^{19}F-NMR** (564 MHz, $CDCl_3$) δ -67.83 (d, *J* = 7.9 Hz). **IR** (KBr): 2923, 2858, 1351, 1141, 1105 cm^{-1}. **HRMS** (ESI) m/z calculated for $C_{15}H_{20}F_3SSi$ $[M+H]^+$ 317.1007, found 317.1014.

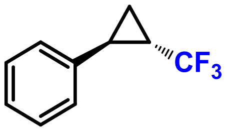

(2-(trifluoromethyl)cyclopropyl)benzene (107) Colourless oil; ^{1}**H-NMR** (600 MHz, $CDCl_3$) δ 7.29 (t, *J* = 7.2 Hz, 2H), 7.22 (t, *J* = 7.8 Hz, 1H), 7.12 (d, *J* = 7.2 Hz, 2H), 2.38-2.33 (m, 1H), 1.83 -1.76 (m, 1H), 1.36 (dt, *J* = 9.6, 5.4 Hz, 1H), 1.19-1.14 (m, 1H); ^{13}**C-NMR** (150 MHz, $CDCl_3$) δ 139.0, 128.6, 126.8, 126.5, 125.9 (q, *J* = 269.3 Hz), 22.9 (q, *J* = 36.8 Hz) , 19.6 (q, *J* = 2.7 Hz), 10.8 (q, *J* = 2.7 Hz); ^{19}**F-NMR** (564 MHz, $CDCl_3$) δ -66.76 (d, *J* = 6.8 Hz), The spectroscopic data match with those reported in the literature[4].

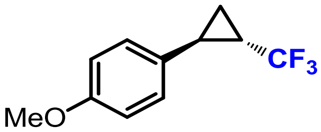

1-methoxy-4-(2-(trifluoromethyl)cyclopropyl)benzene (108) Colourless oil; ^{1}**H-NMR** (600 MHz, $CDCl_3$) δ 6.98 (d, *J* = 8.4 Hz, 2H), 6.76 (d, *J* = 8.4 Hz, 2H), 3.71 (s, 3H), 2.27-2.21 (m, 1H), 1.68-1.62 (m, 1H), 1.24 (dt, *J* = 9.6, 5.4 Hz, 1H), 1.06-1.01 (m, 1H); ^{13}**C-NMR** (150 MHz, $CDCl_3$) δ 158.5, 131.0, 127.7, 126.0 (q, *J* = 269.3 Hz), 114.0, 55.3, 22.6 (q, *J* = 36.6 Hz), 18.9 (q, *J* = 2.7 Hz), 10.4 (q, *J* = 2.6 Hz); ^{19}**F-NMR** (564 MHz, $CDCl_3$) δ -66.68 (d, *J* = 6.8 Hz). The spectroscopic data match with those reported in the literature[4].

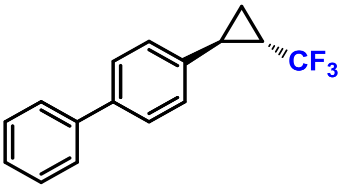

4-(2-(trifluoromethyl)cyclopropyl)-1,1'-biphenyl (109) Colourless oil; ^{1}**H-NMR** (600 MHz, $CDCl_3$) δ 7.44 (d, *J* = 7.2 Hz, 2H), 7.40 (d, *J* = 7.8 Hz, 2H), 7.31 (t, *J* = 7.2 Hz, 2H), 7.23 (d, *J* = 6.6 Hz, 1H), 7.06 (d, *J* = 7.8 Hz, 2H), 2.31-2.24 (m, 1H), 1.76-1.66 (m, 1H), 1.31-1.23 (m, 1H), 1.10-1.03 (m, 1H); ^{13}**C-NMR** (150 MHz, $CDCl_3$) δ 140.6, 139.8, 138.1, 128.8, 127.3, 127.0, 126.9, 126.5, 125.9 (q, *J* = 269.4 Hz), 23.0 (q, *J* = 36.8 Hz), 19.3 (q, *J* = 2.4 Hz), 10.9 (q, *J* = 2.4 Hz); ^{19}**F-NMR** (564 MHz, $CDCl_3$) δ -66.62 (d, *J* = 6.2 Hz). The spectroscopic data match with those reported in the literature[5].

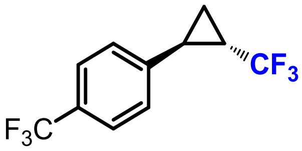

1-(trifluoromethyl)-4-(2-(trifluoromethyl)cyclopropyl)benzene (110) Colourless oil; ^{1}**H-NMR** (600 MHz, $CDCl_3$) δ 7.55 (d, *J* = 7.8 Hz, 2H), 7.22 (d, *J* = 7.8 Hz, 2H), 2.43-2.38 (m, 1H), 1.88-

1.82 (m, 1H), 1.45 (dt, *J* = 9.6, 6.0 Hz, 1H), 1.24-1.18 (m, 1H); **^{13}C-NMR** (150 MHz, $CDCl_3$) δ 143.2, 129.2 (q, *J* = 32.4 Hz), 126.8, 125.6 (q, *J* = 3.8 Hz), 125.5 (q, *J* = 269.3 Hz), 124.1 (q, *J* = 270.2 Hz), 23.4 (q, *J* = 37.1 Hz), 19.4 (q, *J* = 2.6 Hz), 11.2 (q, *J* = 2.4 Hz); **^{19}F-NMR** (564 MHz, $CDCl_3$) δ -62.56, -67.00 (d, *J* = 6.8 Hz). The spectroscopic data match with those reported in the literature[4].

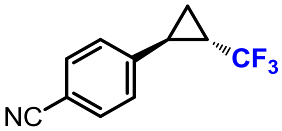

4-(2-(trifluoromethyl)cyclopropyl)benzonitrile (111) Colourless oil; **^{1}H-NMR** (600 MHz, $CDCl_3$) δ 7.59 (d, *J* = 8.4 Hz, 2H), 7.22 (d, *J* = 8.4 Hz, 2H), 2.43-2.38 (m, 1H), 1.91-1.85 (m, 1H), 1.48 (dt, *J* = 11.4, 6.0 Hz, 1H), 1.27-1.21 (m, 1H); **^{13}C-NMR** (150 MHz, $CDCl_3$) δ 144.6, 132.4, 127.1, 125.4 (q, *J* = 269.4 Hz), 118.6, 110.6, 23.6 (q, *J* = 37.2 Hz), 19.6 (q, *J* = 2.7 Hz), 11.5 (q, *J* = 2.6 Hz); **^{19}F-NMR** (564 MHz, $CDCl_3$) δ -67.00 (d, *J* = 6.8 Hz). The spectroscopic data match with those reported in the literature[5].

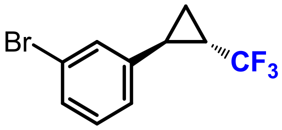

1-bromo-3-(2-(trifluoromethyl)cyclopropyl)benzene (112) Colourless oil; **^{1}H-NMR** (600 MHz, $CDCl_3$) δ 7.33 (d, *J* = 7.8 Hz, 1H), 7.24 (s, 1H), 7.12 (t, *J* = 7.8 Hz, 1H), 7.01 (d, *J* = 7.8 Hz, 1H), 2.32-2.27 (m, 1H), 1.80-1.73 (m, 1H), 1.35 (dt, *J* = 9.6, 5.4 Hz, 1H), 1.15-1.10 (m, 1H); **^{13}C-NMR** (150 MHz, $CDCl_3$) δ 141.4, 130.1, 129.9, 129.6, 125.7 (q, *J* = 269.4 Hz), 125.2, 122.7, 23.0 (q, *J* = 36.9 Hz), 19.1 (q, *J* = 2.7 Hz), 10.8 (q, *J* = 2.4 Hz); **^{19}F-NMR** (564 MHz, $CDCl_3$) δ -66.85 (d, *J* = 6.8 Hz). **IR** (neat): 3065, 1464, 1424, 1337, 1107, 1024 cm^{-1}. **HRMS** (EI): exact mass calcd for $C_{10}H_8BrF_3$ (M^+): 263.9761, found: 263.9760.

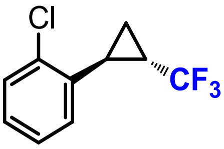

1-chloro-2-(2-(trifluoromethyl)cyclopropyl)benzene (113) Colourless oil; **^{1}H-NMR** (600 MHz, $CDCl_3$) δ 7.36-7.32 (m, 1H), 7.19-7.13 (m, 2H), 6.99 (dd, *J* = 7.2, 1.8 Hz, 1H), 2.58-2.53 (m, 1H), 1.77-1.70 (m, 1H), 1.39 (dt, *J* = 10.2, 5.4 Hz, 1H), 1.17-1.12 (m, 1H); **^{13}C-NMR** (150 MHz, $CDCl_3$) δ 136.3, 135.6, 129.5, 128.2, 127.4, 126.9, 125.9 (q, *J* = 269.4 Hz), 22.1 (q, *J* = 36.8 Hz),

18.0 (q, J = 2.9 Hz), 9.7 (q, J = 2.3 Hz); ^{19}**F-NMR** (564 MHz, $CDCl_3$) δ -66.69 (d, J = 6.8 Hz). **IR** (neat): 3067, 1574, 1465, 1277, 1134, 741 cm^{-1}. **MS** (EI, m/z): 220 (M^+, 66.24), 115 (100.00). **HRMS** (EI): exact mass calcd for $C_{10}H_8ClF_3$ (M^+): 220.0263, found: 220.0262.

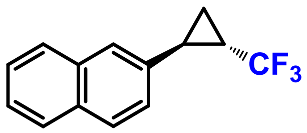

2-(2-(trifluoromethyl)cyclopropyl)naphthalene (114) Colourless oil; ^{1}**H-NMR** (600 MHz, $CDCl_3$) δ 7.80-7.74 (m, 3H), 7.56 (s, 1H), 7.48-7.41 (m, 2H), 7.23-7.20 (m, 1H), 2.54-2.50 (m, 1H), 1.93-1.86 (m, 1H), 1.45-1.40 (m, 1H), 1.30-1.25 (m, 1H); ^{13}**C-NMR** (150 MHz, $CDCl_3$) δ 136.4, 133.3, 132.4, 128.4, 127.7, 127.4, 126.4, 125.9 (q, J = 270.0 Hz), 125.7, 125.1, 124.8, 22.9 (q, J = 36.6 Hz), 19.8 (q, J = 2.6 Hz), 10.8 (q, J = 2.3 Hz); ^{19}**F-NMR** (564 MHz, $CDCl_3$) δ -66.63 (d, J = 6.8 Hz). The spectroscopic data match with those reported in the literature[6].

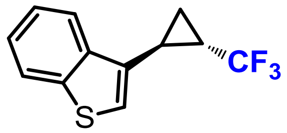

3-(2-(trifluoromethyl)cyclopropyl)benzo[b]thiophene (115) Colourless oil; ^{1}**H-NMR** (600 MHz, $CDCl_3$) δ 7.91 (d, J = 7.8 Hz, 1H), 7.83 (d, J = 7.8 Hz, 1H), 7.44-7.40 (m, 1H), 7.39-7.35 (m, 1H), 7.04 (d, J = 1.2 Hz, 1H), 2.49-2.44 (m, 1H), 1.79-1.71 (m, 1H), 1.42 (dt, J = 9.6, 5.4 Hz, 1H), 1.26 -1.21 (m, 1H); ^{13}**C-NMR** (150 MHz, $CDCl_3$) δ 140.4, 139.0, 134.1, 126.0 (q, J = 269.3 Hz), 124.8, 124.3, 122.9, 122.0, 121.7, 21.2 (q, J = 36.6 Hz), 13.6 (q, J = 2.7 Hz), 8.8 (q, J = 2.3 Hz); ^{19}**F-NMR** (564 MHz, $CDCl_3$) δ -66.52 (d, J = 6.8 Hz). The spectroscopic data match with those reported in the literature[5].

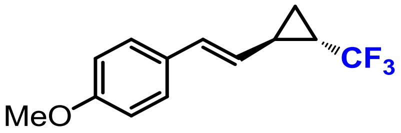

1-methoxy-4-((*E*)-2-(2-(trifluoromethyl)cyclopropyl)vinyl)benzene (116) Colourless oil; ^{1}**H-NMR** (600 MHz, $CDCl_3$) δ 7.16 (d, J = 9.0 Hz, 2H), 6.76 (d, J = 9.0 Hz, 2H), 6.39 (d, J = 15.6 Hz, 1H), 5.55 (dd, J = 15.6, 7.8 Hz, 1H), 3.72 (s, 3H), 1.92-1.86 (m, 1H), 1.57-1.50 (m, 1H), 1.13 (dt, J = 9.6, 5.4 Hz, 1H), 0.88-0.83 (m, 1H); ^{13}**C-NMR** (150 MHz, $CDCl_3$) δ 159.1, 130.1, 129.6, 127.0, 126.5, 126.0 (q, J = 269.1 Hz), 114.0, 55.3, 21.5 (q, J = 36.6 Hz), 18.4 (q, J = 2.7 Hz), 9.8

(q, J = 2.6 Hz); **^{19}F-NMR** (564 MHz, $CDCl_3$) δ -66.55 (d, J = 7.3 Hz). The spectroscopic data match with those reported in the literature[4].

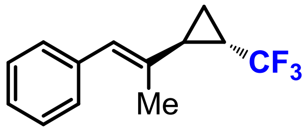

((*E*)-2-(2-(trifluoromethyl)cyclopropyl)prop-1-en-1-yl)benzene (117) Colourless oil; **^{1}H-NMR** (600 MHz, $CDCl_3$) δ 7.31 (t, J = 8.4 Hz, 2H), 7.23-7.17 (m, 3H), 6.35 (s, 1H), 1.99-1.94 (m, 1H), 1.80 (d, J = 1.2 Hz, 3H), 1.69-1.63 (m, 1H), 1.12 (dt, J = 9.6, 6.0 Hz, 1H), 1.09-1.05 (m, 1H); **^{13}C-NMR** (150 MHz, $CDCl_3$) δ 137.5, 134.9, 128.8, 128.1, 126.4, 126.2 (q, J = 267.0 Hz) 126.0, 23.8 (q, J = 2.4 Hz), 20.1 (q, J = 36.5 Hz), 16.1, 8.0 (q, J = 2.4 Hz); **^{19}F-NMR** (564 MHz, $CDCl_3$) δ -66.45 (d, J = 6.8 Hz). The spectroscopic data match with those reported in the literature[7].

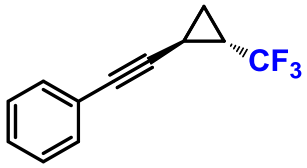

((2-(trifluoromethyl)cyclopropyl)ethynyl)benzene (118) Colourless oil; **^{1}H-NMR** (600 MHz, $CDCl_3$) δ 7.40-7.36 (m, 2H), 7.29-7.26 (m, 3H), 1.97-1.90 (m, 2H), 1.27 (dt, J = 9.0, 5.4 Hz, 1H), 1.20 (dt, J = 9.0, 6.0 Hz, 1H); **^{13}C-NMR** (150 MHz, $CDCl_3$) δ 131.7, 128.3, 128.1, 125.2 (q, J = 269.6 Hz), 122.5, 88.2, 78.0, 22.8 (q, J = 36.8 Hz), 11.4 (q, J = 2.4 Hz), 5.1 (q, J = 3.6 Hz); **^{19}F-NMR** (564 MHz, $CDCl_3$) δ -67.37 (d, J = 6.8 Hz). The spectroscopic data match with those reported in the literature[7].

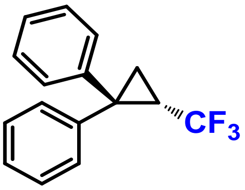

(2-(trifluoromethyl)cyclopropane-1,1-diyl)dibenzene (119) Colourless oil; **^{1}H-NMR** (600 MHz, $CDCl_3$) δ 7.43 (d, J = 7.2 Hz, 2H), 7.31-7.24 (m, 6H), 7.23-7.15 (m, 2H), 2.38-2.29 (m, 1H), 1.89 (t, J = 5.4 Hz, 1H), 1.53-1.48 (m, 1H); **^{13}C-NMR** (150 MHz, $CDCl_3$) δ 144.5, 139.0, 129.6, 128.7, 128.6, 127.8, 127.1, 126.8, 125.9 (q, J = 270.5 Hz), 35.9 (q, J = 1.4 Hz), 26.7 (q, J = 35.6 Hz), 15.5 (q, J = 3.6 Hz); **^{19}F-NMR** (564 MHz, $CDCl_3$) δ -61.41 (d, J = 7.3 Hz). The spectroscopic data match with those reported in the literature[5].

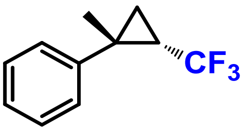

(1-methyl-2-(trifluoromethyl)cyclopropyl)benzene (120), dr > 95:5, Colourless oil; **^{1}H-NMR** (600 MHz, $CDCl_3$) δ 7.32-7.27 (m, 4H), 7.23-7.20 (m, 1H), 1.79-1.71 (m, 1H), 1.52 (s, 3H), 1.35 (dd, *J* = 8.4, 5.4 Hz, 1H), 1.20 (t, *J* = 6.0 Hz, 1H); **^{13}C-NMR** (150 MHz, $CDCl_3$) δ 145.5, 128.6, 127.5, 126.8, 126.7 (q, *J* = 270.5 Hz), 28.7, 26.1 (q, *J* = 35.7 Hz), 20.8 (q, *J* = 6.6 Hz), 16.6 (q, *J* = 2.4 Hz); **^{19}F-NMR** (564 MHz, $CDCl_3$) δ -66.84 (d, *J* = 6.8 Hz). The spectroscopic data match with those reported in the literature[4].

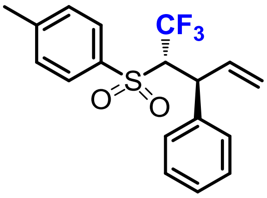

95'

1-methyl-4-((1,1,1-trifluoro-3-phenylpent-4-en-2-yl)sulfonyl)benzene (95'), White solid; **^{1}H-NMR** (600 MHz, $CDCl_3$) δ 7.64 (d, *J* = 8.4 Hz, 2H), 7.38 (d, *J* = 7.8 Hz, 2H), 7.33 (t, *J* = 7.6 Hz, 2H), 7.29-7.26 (m, 3H), 6.65-6.59 (m, 1H), 5.33 (d, *J* = 10.2 Hz, 1H), 5.26 (d, *J* = 16.8 Hz, 1H), 4.39 (dd, *J* = 9.0, 3.6 Hz, 1H), 4.17 (qd, *J* = 8.4, 3.6 Hz, 1H), 2.43 (s, 3H); **^{13}C-NMR** (150 MHz, $CDCl_3$) δ 145.4, 139.2, 137.2, 135.3, 129.8, 128.6, 128.5, 127.4, 124.0, 122.1, 119.0, 72.2 (q, *J* = 25.0 Hz), 46.8, 21.7; **^{19}F-NMR** (470 MHz, $CDCl_3$) δ -61.09 (d, *J* = 8.5 Hz). **IR** (KBr): 3037, 2954, 1911, 1598, 1452, 1332, 1252, 1167, 1146, 1107, 953, 714 cm^{-1}. **HRMS** (ESI) m/z calculated for $C_{18}H_{17}F_3NaO_2S$ $[M+Na]^+$ 377.0799, found 377.0802.

Supplementary Discussion

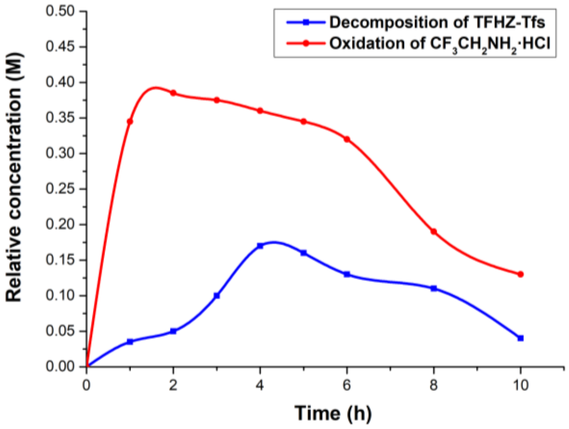

Supplementary Figure 363. **Comparison of the methods for generation of trifluorodiazoethane by acidic oxidation and basic decomposition. Red curve**: $CF_3CH_2NH_2$•HCl (1.2 mmol), $NaNO_2$ (1.5 mmol) and 53 μL H_2O at 0 ℃ in $CDCl_3$ (2 mL). **Blue curve**: TFHZ-Tfs (1.2 mmol) and NaH (2.4 mmol) at 40 ℃ in $CDCl_3$ (2 mL).

The benefit of this CF_3CHN_2-generating method was established by comparing the concentration profile of trifluorodiazoethane released from trifluoroethylamine oxidation with TFHZ-Tfs decomposition. A rapid release and significant accumulation of CF_3CHN_2 was observed in the oxidation of $CF_3CH_2NH_2$ HCl, as had been described in previous reports (red curve);[1] in contrast, decomposition of TFHZ-Tfs led to a slow release of CF_3CHN_2 with a peak around 4-5 h, and overall a significantly lower concentration of CF_3CHN_2 throughout the reaction. These results indicate that the use of TFHZ-Tfs, without special precautions, can avoid excessive accumulation of CF_3CHN_2 in solution.

General procedure for the NMR timepoint experiments of TFHZ-Tfs

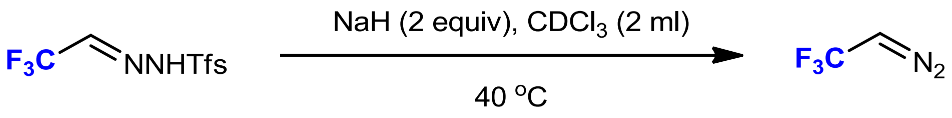

In a screw capped reaction vial was charged with TFHZ-Tfs (384.0 mg, 1.2 mmol), and NaH (96.0 mg, 60 wt%, 2.4 mmol), and then added dry $CDCl_3$ (2 mL) via a syringe. The vessel was sealed with a cap and the mixture was stirred at 40 ℃ for specified time. After completion, the resulting mixture was cooled to room temperature, and added 0.3 mmol of CH_2Br_2. Then the **^{1}H NMR** was recorded to calibrate the yield.

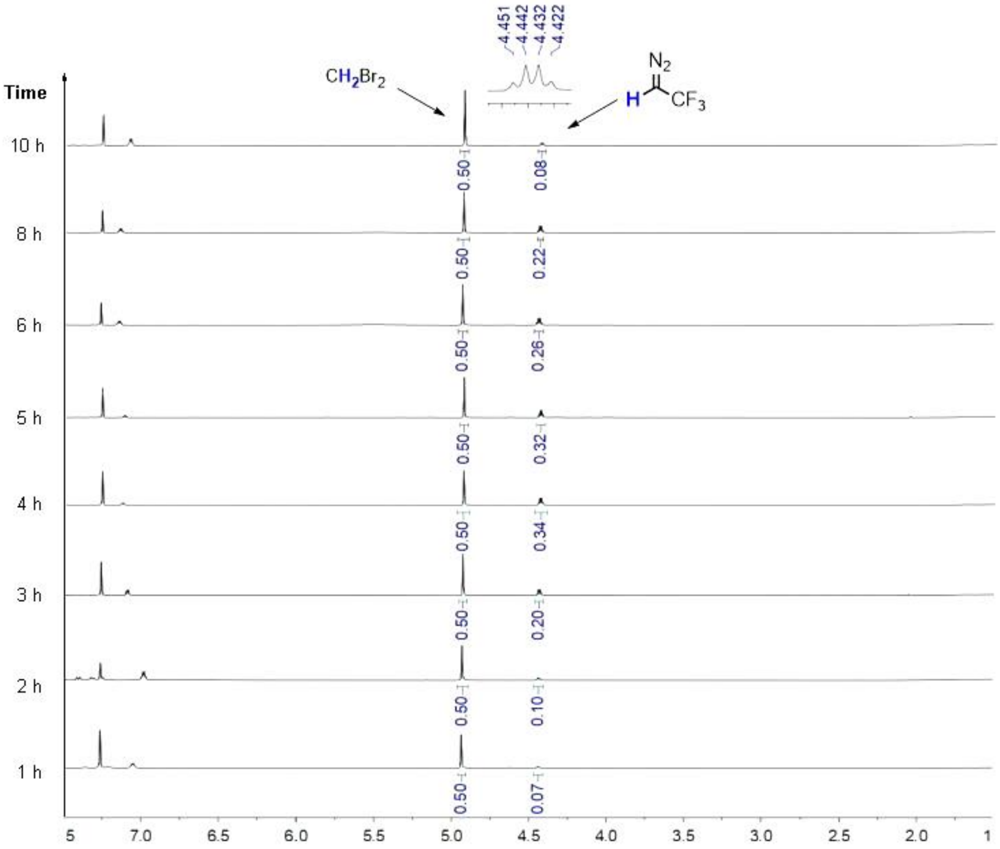

Supplementary Figure 364. NMR timepoint experiments of TFHZ-Tfs.

General procedure for the NMR timepoint experiments of $CF_3CH_2NH_2$·HCl

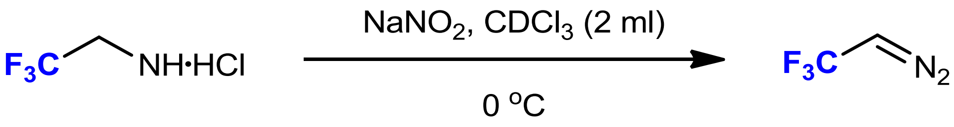

In a screw capped reaction vial was charged with $CF_3CH_2NH_2$•HCl (162.6 mg, 1.2 mmol) and $NaNO_2$ (103.5 mg, 1.5 mmol) was added 2 mL of $CDCl_3$ and 53 μL of distilled H_2O. The vessel was sealed with a cap and the mixture was stirred at 0 ℃. After completion, the resulting

mixture was cooled to room temperature, and added 0.3 mmol of CH_2Br_2. Then the ^{1}**H NMR** was recorded to calibrate the yield.

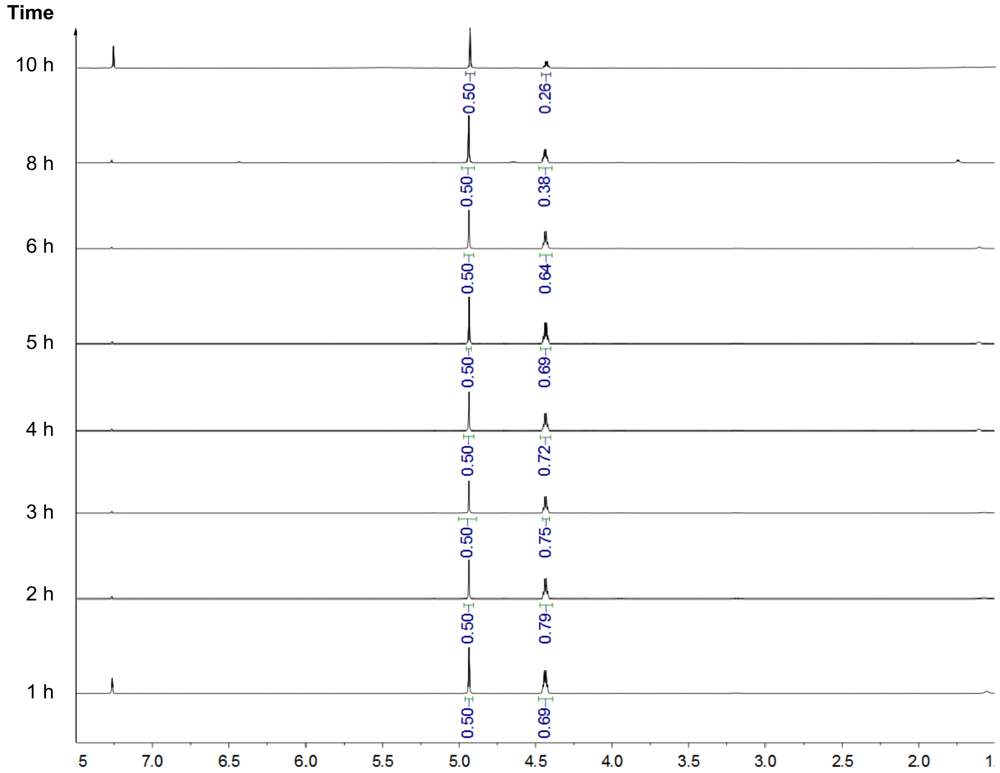

Supplementary Figure 365. NMR timepoint experiments of $CF_3CH_2NH_2$ ·HCl

Mechanismtic investigations

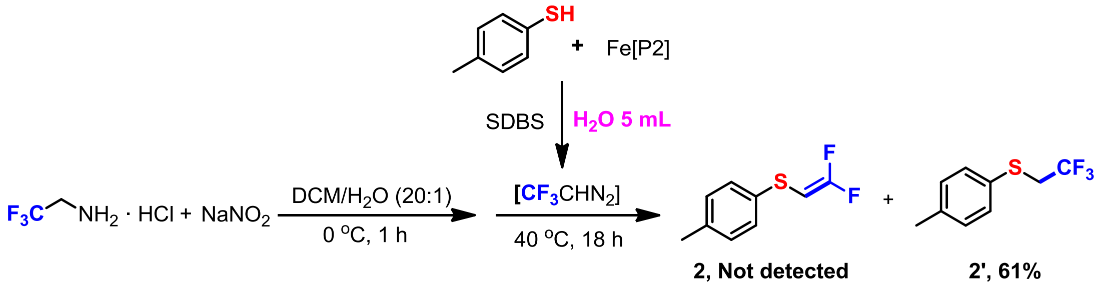

Supplementary Figure 366. Mechanismtic investigation without base.

To a dry 10-mL Schlenk charged with $CF_3CH_2NH_2$ ·HCl (162.6 mg, 1.2 mmol) and $NaNO_2$ (103.5 mg, 1.5 mmol) was added 1.2 mL of DCM and 53 μL of distilled H_2O, and the mixture was stirred at 0 °C for 1 h. Then *p*-toluenethiol (37.3 mg, 0.3 mmol), **Fe[P2]** (5.06 mg, 0.003 mmol), SDBS (sodium dodecylbenzenesulphonate) (31.4 mg, 0.09 mmol) and DI H_2O (5.0 mL) transferred into the solution in a single portion under air and the mixture was placed in a 40 ℃ oil bath for 18 h. Then 10 mL water was added to the mixture, which was extracted with DCM (3 ×

10 mL). The organic layer was combined and dried with anhydrous $MgSO_4$, then filtered through a short silica gel eluting with DCM and the solvent was removed under reduced pressure. Finally, we got **2'** in 61% yield (^{1}H NMR).

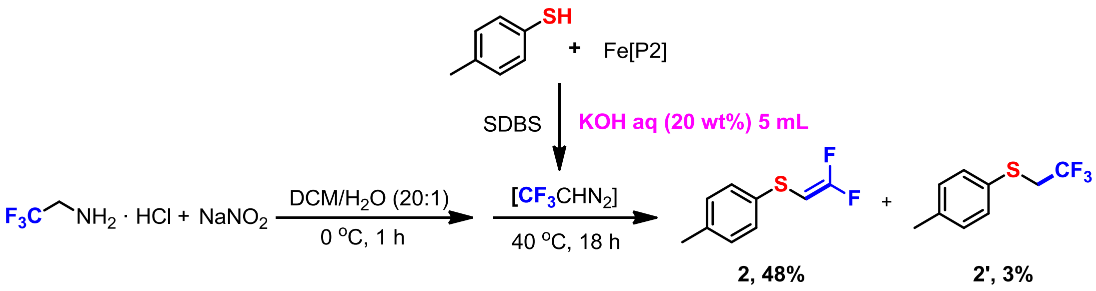

Supplementary Figure 367. Mechanismtic investigation with base.

To a dry 10-mL Schlenk charged with $CF_3CH_2NH_2$ HCl (162.6 mg, 1.2 mmol) and $NaNO_2$ (103.5 mg, 1.5 mmol) was added 1.2 mL of toluene and 53 µL of distilled H_2O, and the mixture was stirred at 0 °C for 1 h. Then *p*-toluenethiol (37.3 mg, 0.3 mmol), **Fe[P2]** (5.06 mg, 0.003 mmol), SDBS (sodium dodecylbenzenesulphonate) (31.4 mg, 0.09 mmol) and KOH aq (20 wt%) (5 mL) transferred into the solution in a single portion under air and the mixture was placed in a 40 ℃ oil bath for 18 h. Finally, we got **2** in 48% and **2'** in 3% yield (^{1}H NMR).

Supplementary References

1 Lu, H., Li, C., Jiang, H., Lizardi, C. L. & Zhang, X. P. Chemoselective Amination of Propargylic C(sp^3)-H Bonds by Cobalt(II)-Based Metalloradical Catalysis. *Angew. Chem. Int. Ed.* **53**, 7028-7032 (2014).

2 Gotfredsen, H., Jevric, M., Kadziola, A. & Nielsen, M. B. Acetylenic Scaffolding with Subphthalocyanines. *Eur. J. Org. Chem.* **2016**, 17-21 (2016).

3 Zhang, D., Liu, H., Zhu, P., Meng, W. & Huang, Y. One-pot syntheses of N-(α-fluorovinyl)azole derivatives from N-(diphenylmethylene)-2,2,2-trifluoroethanamine. *RSC Adv.* **6**, 73683-73691 (2016).

4 Morandi, B., Mariampillai, B. & Carreira, E. M. Enantioselective cobalt-catalyzed preparation of trifluoromethyl-substituted cyclopropanes. *Angew. Chem. Int. Ed.* **50**, 1101-1104 (2011).

5 Duan, Y., Lin, J.-H., Xiao, J.-C. & Gu, Y.-C. A Trifluoromethylcarbene Source. *Org. Lett.* **18**, 2471-2474 (2016).

6 Kotozaki, M., Chanthamath, S., Fujii, T., Shibatomi, K. & Iwasa, S. Highly enantioselective synthesis of trifluoromethyl cyclopropanes by using Ru(II)–Pheox catalysts. *Chem.Commun.* **54**, 5110-5113 (2018).

7 Morandi, B., Cheang, J. & Carreira, E. M. Iron-Catalyzed Preparation of Trifluoromethyl Substituted Vinyl- and Alkynylcyclopropanes. *Org. Lett.* **13**, 3080-3081 (2011).
